# Palladium-Catalyzed Access to Benzocyclobutenone-Derived Ketonitrones via C(sp^2^)–H Functionalization

**DOI:** 10.1021/acs.orglett.2c01317

**Published:** 2022-05-25

**Authors:** Jakub Brześkiewicz, Rafał Loska

**Affiliations:** Institute of Organic Chemistry, Polish Academy of Sciences, Kasprzaka 44/52, 01-224 Warsaw, Poland

## Abstract

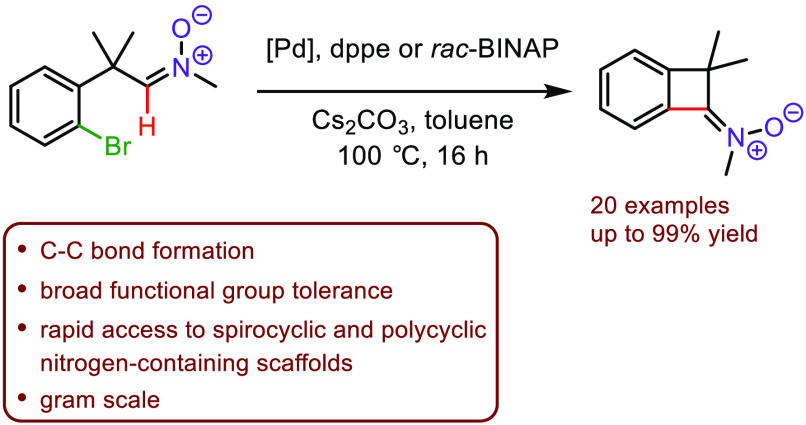

The palladium-catalyzed C(sp^2^)–H functionalization of bromoaryl aldonitrones leading to benzocyclobutenone-derived ketonitrones is described. This method allows for the preparation of a wide range of strained, four-membered ketonitrones with broad functional group tolerance. Downstream transformations of the formed products were readily demonstrated, illustrating the synthetic utility of the obtained benzocyclobutenone-derived nitrones for the construction of polycyclic nitrogen-containing scaffolds.

The strain inherent in four-membered rings renders them versatile building blocks in organic synthesis.^1^ In particular, benzocyclobutenes (BCBs) have been recognized as valuable synthons with numerous synthetic applications disclosed in recent decades,^2^ often relying upon the four-membered ring opening to *o*-quinodimethane derivatives, followed by cycloaddition restoring the aromaticity of the benzene ring.^3^ In the past few years, new modes of BCBs transformations were actively developed, initiated by cleavage of the proximal^4^ or distal^5^ C–C bond or involving C–H functionalization^6^ or ring expansion upon addition of nucleophiles.^7^ The high synthetic potential of the BCB framework, as well as its occurrence in polymer precursors, complex natural compounds, and drugs such as ivabradine,^8^ is directly reflected in a variety of strategies developed for their preparation, including [2 + 2] cycloaddition and Pd-catalyzed or photocatalyzed cyclization.^9^

We envisioned that the repertoire of useful transformations of BCBs could be significantly expanded by incorporating a nitrone moiety into the four-membered ring. In fact, while the synthesis and synthetic utility of heterocyclic four-membered nitrones have been recently highlighted by Anderson’s group,^10^ only few reports on cyclobutanone-derived nitrones are available,^11^ but none about nitrones derived from cyclobutenone or benzocyclobutenone. Nitrones exhibit very rich chemistry^12^ and readily participate in 1,3-cycloaddition,^13^ reductive coupling^14^ or addition of nucleophiles, including Pictet–Spengler type cyclizations;^15^ therefore, they are versatile substrates in the synthesis of a variety of nitrogen-containing compounds. In this context, special emphasis is placed upon the synthesis of ketonitrones, as they are excellent precursors of quaternary carbon centers (e.g., preparation of C^α^-tetrasubstituted α-amino acids).^16^ However, the availability of ketonitrones, compared to aldonitrones, is still limited. In recent years, new methods for accessing highly functionalized ketonitrones were investigated, such as hydromagnesation, oxime functionalization, or nucleophilic addition to amide derivatives.^17^

Transition metal catalyzed C–H activation reactions proceed in an elegant atom- and step-economic fashion.^18^ Their efficiency in the formation of four-membered rings of BCBs via C(sp^3^)–H activation is well-documented.^9c,9d,19^ Concerning coupling of two C(sp^2^) carbon atoms, the 2010 seminal report of Martin et al. indicated that benzocyclobutenones could be obtained by simple palladium-catalyzed cyclization of haloaryl-containing aldehydes (Scheme 1B).^20^ We have recently disclosed a Pd-catalyzed reaction for the C–H activation of aldonitrones bearing an ester group to access various aryl ketonitrones (Scheme 1A).^21^ In this approach, a reaction pathway has been proposed in which the nitrone oxygen atom serves as the directing group, facilitating the cross-coupling process. Accordingly, our envisioned strategy toward benzocyclobutenone-derived nitrones, which we term benzocyclobutenitrones (BCBn), is based upon palladium-catalyzed cyclization of bromoaryl-substituted aldonitrones (Scheme 1C). The target strained ketonitrones are interesting by themselves in terms of their reactivity and preparation of nitrogen-containing compounds. Even more importantly, as we demonstrate herein, facile preparation of BCBn opens opportunities in the development of cascade or tandem reactions that combine the peculiar reactivity of BCBs with that of ketonitrones and allow for expeditious preparation of polycyclic nitrogen-containing scaffolds.

**Scheme 1:**
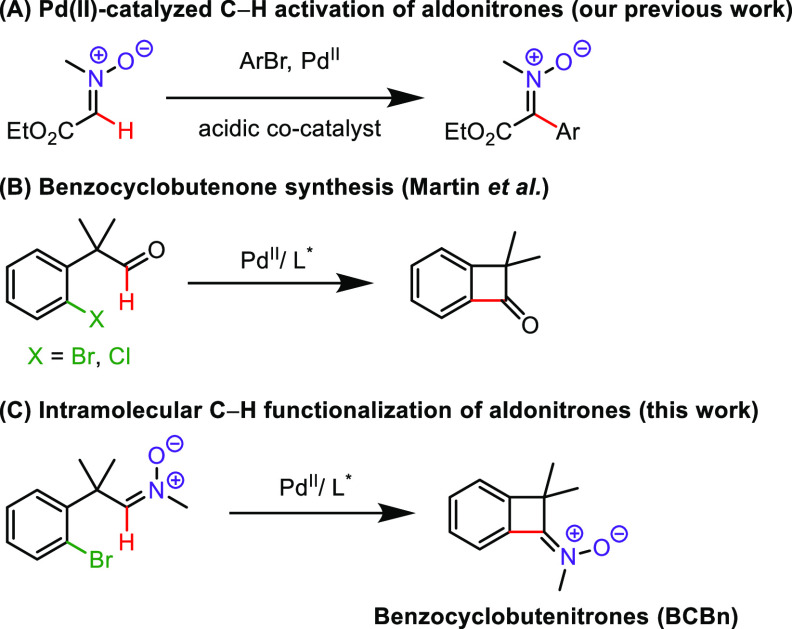
C(sp^2^)–H Functionalization of Aldonitrones and Aldehydes

At the outset of the project, we searched for the optimal reaction conditions using aldonitrone **1a** as the model substrate (Table 1). In the initial experiment, we used the conditions similar to those reported by Martin et al.^20^ for cyclobutenone synthesis (entry 1), but unexpectedly, no coupling product was detected. Next, we examined different ligands and solvent (toluene), but the results remained unsatisfactory (Table 1, entries 2 and 3). A breakthrough came upon increasing the reaction temperature to 120 °C which led to the formation of trace amounts of the desired ketonitrone **2a** (entry 4). Upon replacing 1,4-dioxane with toluene, the yield of the C–H functionalization process was dramatically improved to 74% (Table 1, entries 5 and 6). Finally, it was found that, with toluene as a solvent and Cs_2_CO_3_ as a base, the cyclization reaction temperature could be lowered back to 100 °C, which led to an excellent yield of **2a** particularly with the dppe ligand (94%; entry 7). This result indicates that the BCBn **2a** is characterized by moderate stability at elevated temperature (120 °C), probably due to the strained nature of the ring present in its structure. A reduced amount of phosphine was found to be detrimental for this transformation (Table 1, entry 10). Notably, no decarbonylation products were observed, unlike the palladium-catalyzed functionalization of aldehydes.^22^

**Table 1:**
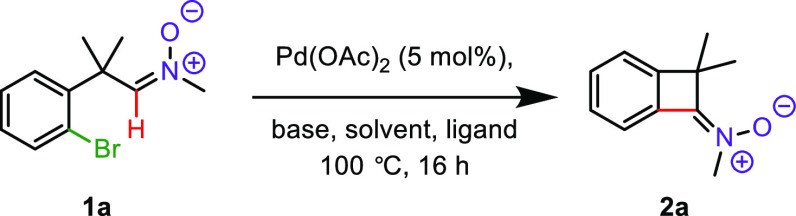
Optimizations Studies^a^

entry	ligand	solvent	base	yield (%)^b^
1	*rac*-BINAP	1,4-dioxane	Cs_2_CO_3_	N. R.
2	dppe	1,4-dioxane	Cs_2_CO_3_	N. R.
3^c^	PPh_3_	toluene	K_2_CO_3_	N. R.
4^d^	dppe	1,4-dioxane	Cs_2_CO_3_	traces
5^d^	dppe	toluene	Cs_2_CO_3_	57
6^d^	PPh_3_	toluene	Cs_2_CO_3_	74
**7**	**dppe**	**toluene**	**Cs**_**2**_**CO**_**3**_	**94**
8	PPh_3_	toluene	Cs_2_CO_3_	80
9	*rac*-BINAP	toluene	Cs_2_CO_3_	77
10^e^	dppe	toluene	Cs_2_CO_3_	85

a Reaction conditions: **1a** (0.5 mmol), Pd(OAc)_2_ (5 mol %), ligand (12 mol %), base (1 mmol), solvent (2.0 mL), 100 °C, 16 h.

b Isolated yields.

c PivOH as an additive (30 mol %).

d Reaction at 120 °C.

e Ligand (6 mol %) was used.

With the optimized conditions in hand, we then examined the efficiency of the four-membered ketonitrone formation process on a range of aldonitrones **1** which could be readily obtained from (2-bromophenyl)acetonitriles (see the Supporting Information). It is worth noting that several substrates (**2e**–**f**, **2j**, **2n**, **2r**) underwent a more efficient coupling reaction with *rac*-BINAP as a ligand rather than with dppe (Scheme 2). A broad range of electron-withdrawing and -donating substituents in the benzene ring, at positions *para*- and *meta*- with respect to the bromine atom, were tolerated in this reaction (54–99% yields). Products with both CO_2_Me (**2d**) and NEt_2_ (**2g**) groups were obtained in high yields (99% and 81%, respectively).

**Scheme 2:**
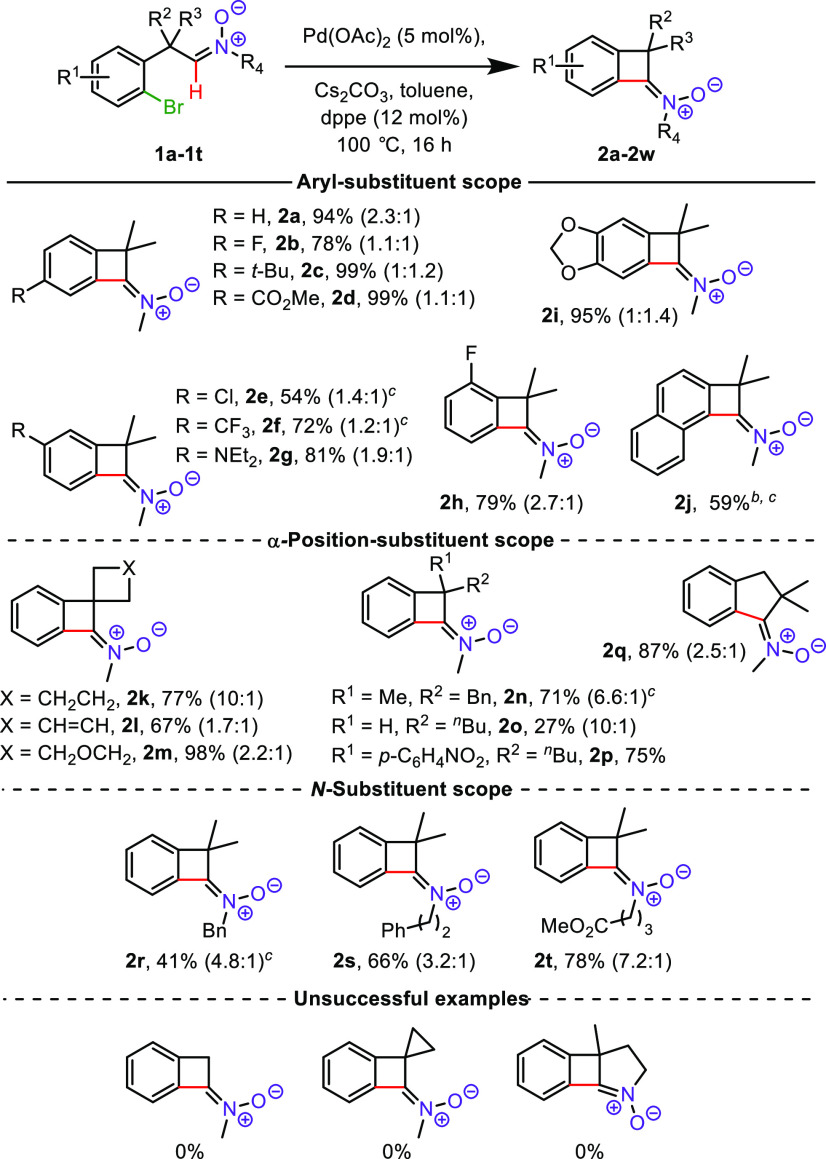
Scope of the Reaction Reaction conditions: aldonitrone (0.5 mmol), Pd(OAc)_2_ (5 mol %), dppe (12 mol %), Cs_2_CO_3_ (1 mmol), toluene (2.0 mL), 100 °C, 16 h, under an argon atmosphere. Reaction performed at 120 °C. *rac*-BINAP (12 mol %) instead of dppe.

The coupling process for **2e**, where the chlorine atom is present in aryl moiety, also proceeded smoothly (54% yields). Next, substituents in position α to the nitrone moiety were investigated. Good to excellent yields were obtained for spirocyclic ketonitrones (**2k**–**m**, from 67% to 98%) as well as for BCBn **2n** and **2p** with an asymmetric quaternary carbon center at the α-position, 71% and 75%, respectively. Noteworthy, in the case of **2n** and **2p** no competitive coupling between the bromoaryl ring and the aromatic α substituents was observed. The cyclization process was considerably less efficient for the mono α-substituted nitrone **2o** (27%). Aldonitrone bearing no α-substituents or a cyclopropyl ring failed to react, presumably due to a lack of the Thorpe–Ingold effect. Aldonitrone **1q** with an extra −CH_2_– group furnished the desired Indane-derived ketonitrone **2q** in excellent yield (87%). An *N*-benzyl, *N*-homobenzyl, and an *N*-alkyl containing an ester group with acidic α hydrogens were all compatible with the coupling process, delivering the corresponding BCBn from moderate (**2r**, 41%) to good (**2s**, 66%; **2t**, 78%) yields.

Moderate yields of some ketonitrones **2** resulted from incomplete conversion of substrate, with exception of **2l**, **2o**, **2r**, and **2s**. These nitrones (or the respective starting aldonitrones) partially decomposed to unidentified tarry products under the cross-coupling conditions.

To showcase the utility of the prepared BCBn, we attempted their further transformations that exploit the presence of the nitrone functionality within the strained cyclobutene ring (Scheme 3). Ketonitrone **2a** could be readily engaged in 1,3-dipolar cycloaddition with *N*-methylmaleimide furnishing polycyclic isoxazolidine **3** in 81% yield as a single diastereoisomer. Its structure was confirmed by X-ray diffraction analysis, providing also a confirmation of the structure of ketonitrones **2**.^23^ Nitrone **2a** reacted efficiently with aryne generated from 2-(trimethylsilyl)phenyl trifluoro-methanesulfonate, giving fused isoxazolidine **4** in excellent yield (88%) or the four-membered ring opening product **5** at elevated temperature.

**Scheme 3:**
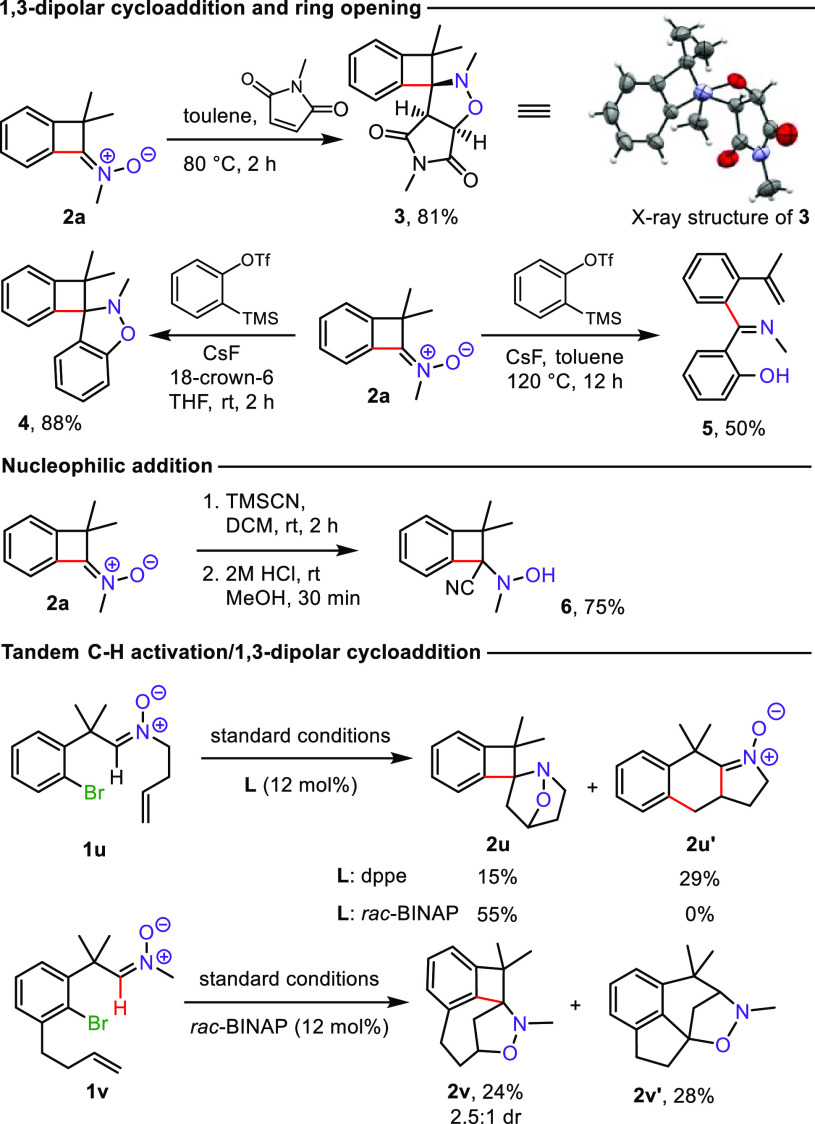
Further Transformations of BCBn **2**

Treatment of BCBn **2a** with TMSCN, followed by acidic deprotection, afforded the α-cyanated *N*-methylhydroxylamine **6**, a potential precursor of α-amino acid derivatives containing a benzocyclobutene ring (Scheme 3).^9a,24^

To demonstrate the utility of our protocol for the construction of polycyclic scaffolds containing nitrogen, we examined tandem C–H functionalization/1,3-dipolar cycloaddition processes with aldonitrones **1u**, **1v** bearing a homoallyl substituent. In the Pd-catalyzed reaction of nitrone **1u** in the presence of dppe, we observed formation of two isomeric products—a bridged isoxazolidine **2u** resulting from BCBn formation followed by its intramolecular cycloaddition and ketonitrone **2u′**, the formation of which can be explained by insertion of palladium into the C–Br bond, migratory insertion into the double bond of the homoallyl substituent, and finally coupling with the nitrone moiety. Interestingly, by changing the ligand to *rac*-BINAP, the BCB-type product **2u** formed exclusively in 55% yield. Apparently, after the initial oxidative insertion into the C–Br bond, the reaction course could be controlled by the selection of the catalytic system. A similar aldonitrone **1v** with a homoallyl substituent in the benzene ring in the presence of *rac*-BINAP underwent a tandem process with the formation of isoxazolidine **2v** in 24% yield and a Heck/cycloaddition product **2v′** in 28% yield. The structures of compounds **2u**, **2u′**, **2v**, **2v′** were confirmed by 2D NMR spectroscopy.

Nitrones are also excellent precursors of β-lactams.^25^ In particular, cyclobutenone-derived ketonitrones could serve as substrates for the straightforward preparation of the azaspiro[3.3]heptane skeleton, which is an emerging privileged structural motif in medicinal chemistry.^26^ Indeed, 1,3-dipolar cycloaddition between BCBn **2n** and 2*H*-pentafluoropropene (PFP) afforded isoxazolidine **7** in 72% yield (Scheme 4). Subsequent hydrogenation of isoxazolidine **7** led to fluorinated, spirocyclic β-lactams **8, 8′** (85% 7.5:1) which could be readily separated by chromatography.

**Scheme 4:**
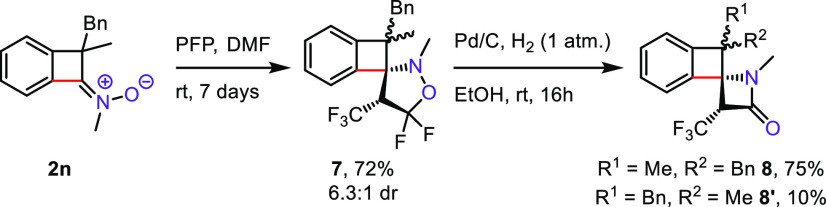
Synthesis of Spirocyclic β-Lactams

To highlight the practicality of the BCBn synthesis protocol, we performed the gram-scale experiment. Gratifyingly, this transformation was successfully scaled up to 2.56 g of **1a** with a lower loading of a Pd(II) catalyst (2 mol %) to deliver **2a** in 91% yield.

A kinetic isotope effect (KIE) experiment was conducted to gain mechanistic insight into the process of the intramolecular coupling of aldonitrones **1** (see the Supporting Information). The intermolecular competition reaction between **1a** and **1a**-*d*_1_ (deuterated at the nitrone carbon atom) resulted in determination of the KIE value of 1.06, suggesting that the C–H cleavage might not be involved in the turnover-limiting step, in contrast to benzocyclobutenone formation examined by Martin. Therefore, we hypothesize that, after oxidative insertion into the aryl C–Br bond, a Heck-type reaction with the double C=N bond occurs, followed by β-hydride elimination to restore the nitrone group.

In conclusion, we developed a method to access previously unknown benzocyclobutenitrones via an intramolecular, four-membered ring forming C–H functionalization process. To our knowledge, this is the first protocol for the synthesis of benzocyclobutenone-derived ketonitrones, and it allows for their preparation in a highly atom-economical manner and in good or excellent yields. Given the broad substrate scope and the high synthetic potential of benzocyclobutenitrones for the synthesis of nitrogen-substituted benzocyclobutenes, including spirocyclic β-lactams, as well as nitrogen-containing polycyclic compounds, we believe this protocol will find broad applicability in nitrone chemistry. Further studies toward applications of BCBn in other complex transformations are currently underway in our laboratory.

## Supporting Information Available

Experimental procedures, characterization data, and reproductions of NMR spectra of the synthesized compounds (PDF)
