# Supplementary material for: Palladium-Catalyzed Access to Benzocyclobutenone-Derived Ketonitrones via C(sp2)–H Functionalization
Source: Org Lett. 2022 May 25;24(22):3960–4. doi: 10.1021/acs.orglett.2c01317 (PMC9278523; doi:10.1021/acs.orglett.2c01317)

Supporting Information

Palladium-Catalyzed Access to Benzocyclobutenone-derived Ketonitrones *via* C(sp^2)–H Functionalization

Jakub Brześkiewicz and Rafał Loska*

Institute of Organic Chemistry, Polish Academy of Sciences, Kasprzaka 44/52, 01-224 Warsaw, Poland

Corresponding author e-mail: rloska@icho.edu.pl

Table of contents

1. General information S2
2. Optimization details S3
3. Experimental procedures S3
    3.1. Synthesis of starting materials S3
    3.2. Palladium catalyzed nitrone cyclization S20
    3.3. Scale-up procedure S30
    3.4. Application of ketonitrones S30
4. Unsuccessful and low-yielding substrates S33
5. Mechanistic considerations S34
6. Crystallographic data S35
7. References S42
8. NMR Spectra S42

1. General information

Reagents and solvents

Analytical grade solvents were used as received. Hexanes and dichloromethane (DCM) used for extraction and chromatography were distilled before use. All anhydrous solvents were distilled from appropriate drying agents prior to use and stored over 3Å molecular sieves or directly taken from commercial sealed bottles under an atmosphere of argon. All commercially available reagents were used as received, including palladium catalysts (Sigma-Aldrich, Fluorochem), phosphines (Fluorochem), *N*-methylhydroxylamine hydrochloride (J&K), *N*-benzylhydroxylamine hydrochloride (J&K), 2-bromophenylacetonitrile (Alfa Aesar), cesium carbonate (Acros Organics), methyl iodide (Sigma-Aldrich) and DIBAL-H solution (1M in hexanes, Sigma-Aldrich). Potassium *tert*-butoxide (J&K) was stored and weighed in a glovebox under nitrogen atmosphere.

Characterization

NMR spectra were recorded at 298 K in $CDCl_3$ solutions using a Bruker 400 MHz or Varian-Agilent 500 MHz spectrometer. The ^{1}H (400 MHz or 500 MHz) and ^{13}C NMR (101 MHz or 125 MHz) chemical shifts are given relative to TMS (0.0 ppm) and relative to $CFCl_3$ for ^{19}F spectra (376 MHz). Mass spectra and High-resolution mass spectra (HRMS) were recorded on a Waters AutoSpec Premier instrument using electron ionization (EI) or a Waters SYNAPT G2-S HDMS instrument using electrospray ionization (ESI, APCI). Spectrometers are equipped with q-TOF type mass analyzer (ESI, APCI) or EBE double focusing geometry mass analyzer (EI). IR spectra were obtained using a JASCO 6200 or Shimadzu IRTRACER-100 FT-IR spectrometer. Elemental analysis (C, H, N) were performed using an Elementar UNICUBE elemental analyzer. Melting point temperatures were measured at a heating rate of 3 °C/min. Column chromatography was performed using silica gel 60 (0.040−0.063 mm). Analytical thin layer chromatography (TLC) was performed using pre-coated silica gel plates (0.20 mm thickness) and visualized under a UV lamp.

2. Optimization details

Table S1. Optimization studies

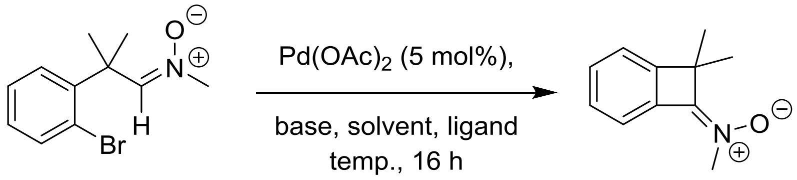

1a **2a**

entry	ligand	solvent	temp. [°C]	base	yield (%)
1[a]	PPh_3	toluene	100	K_2CO_3	N. R
2[a]	dppe	1,4-dioxane	100	K_2CO_3	N. R
3	dppe	1,4-dioxane	100	Cs_2CO_3	N. R
4	rac-BINAP	1,4-dioxane	100	Cs_2CO_3	N.R
5	dppe	1,4-dioxane	120	Cs_2CO_3	traces
6	dppe	toluene	120	Cs_2CO_3	57
7	PPh_3	toluene	120	Cs_2CO_3	74
8	dppe	toluene	120	K_2CO_3	traces
**9**	**dppe**	**toluene**	**100**	**Cs_2CO_3**	**94**
10	rac-BINAP	toluene	100	Cs_2CO_3	77
11	PPh_3	toluene	100	Cs_2CO_3	80
12[b]	dppe	toluene	100	Cs_2CO_3	85

Conditions: **1a** (0.5 mmol), base (1 mmol), solvent (2 mL), $Pd(OAc)_2$ (5 mol%), ligand (12 mol%), 16h, under an Ar atmosphere. [a]PivOH (30 mol%) as an additive. [b]Ligand (6 mol%).

3. Experimental procedures

3.1 Synthesis of the starting materials

Synthesis of nitriles

All known 2-bromophenylacetonitrile derivatives were synthesized from corresponding 2-bromobenzyl bromides according to **GP1** and their ^1H NMR spectra were consistent with those reported in the literature. [1,2]

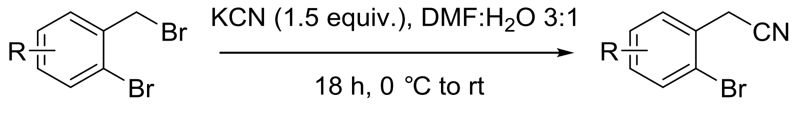

S1

General procedure for the cyanation of 2-bromobenzyl bromide derivatives (GP1):

A 100 mL round-bottom flask was charged with the benzyl bromide derivative (10 mmol, 1 equiv.), DMF (15 mL) and distilled H_2O (5 mL). Then the resulting suspension was cooled down to 0 °C and KCN (0.977 g, 15 mmol, 1.5 equiv.) was added. The mixture was allowed to warm to room temperature and stirred for 18 h. After that time, the solution was diluted with H_2O (ca. 50 mL), transferred into a separatory funnel and extracted with EtOAc (3 x 50 mL). The combined organic phases were washed with brine (3 x 50 mL) and dried over anhydrous Na_2SO_4. The solution was

filtered, concentrated under reduced pressure and purified by column chromatography (silica gel, hexanes/EtOAc).

2-(2-Bromo-4-*tert*-butylphenyl)acetonitrile (S1c)

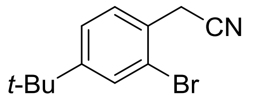

Following **GP1** from 2-bromo-1-bromomethyl-4-*tert*-butylbenzene.[3] The crude was purified by column chromatography to afford the title compound as a white solid in 76% yield (1.92 g, 7.6 mmol).

mp 31-33 °C. ***Rf:*** 0.22 (Hexane). **^{1}H NMR (CDCl$_3$, 400 MHz):** δ 7.60 (d, 1H, J = 1.9 Hz), 7.44 (d, 1H, J = 8.1 Hz), 7.36 (dd, 1H, J = 8.1 Hz, 1.9 Hz), 3.80 (s, 2H), 1.31 (s, 9H). **^{13}C NMR (CDCl$_3$, 100 MHz):** δ 153.6, 130.1, 129.3, 126.8, 125.2, 123.4, 117.0, 34.6, 31.0, 24.3. **IR (CH$_2$Cl$_2$, cm^{-1})** ν_{max}: 2964, 2906, 2871, 2251, 1604, 1553, 1495, 1389, 1261, 1038, 814. **MS (EI 70 eV, *m/z*, %)** 251 (M$^+$, 25), 236 (100), 208 (26), 157 (21), 115 (17), 69 (21); **HRMS (EI$^+$)** *m/z*: [M]$^+$ Calcd for $C_{12}H_{14}NBr$ 251.0310; Found 251.0298.

2-(2-Bromo-3-(but-3-en-1-yl)phenyl)acetonitrile (S1v)

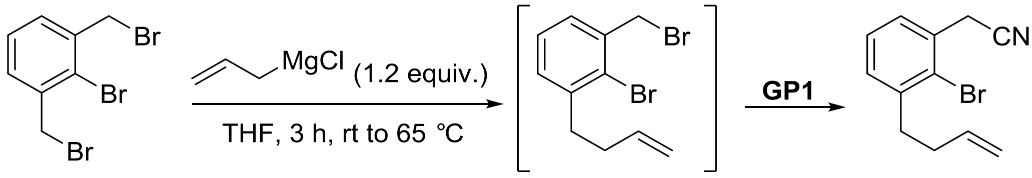

S1v

Under argon atmosphere, a flame-dried Schlenk flask was loaded with commercially available 2-bromo-1,3-bis(bromomethyl)benzene (6.857 g, 20 mmol, 1 equiv.) and anhydrous THF (20 mL). Solution of allylmagnesium chloride (1.7M in THF, 14.1 mL, 24 mmol, 1.2 equiv.) was added dropwise and the reaction mixture was stirred at 65 °C for 3 h (oil bath). Then, the reaction mixture was cooled down and quenched with saturated $NH_4Cl_{(aq)}$ solution (50 mL). The aqueous phase was extracted with EtOAc (2 x 10 mL) and the combined organic phases were dried over Na_2SO_4. The volatiles were removed under reduced pressure to provide **2-bromo-1-(bromomethyl)-3-(but-3-en-1-yl)benzene** (5.53 g) which was used without further purification to prepare compound **S1v** following **GP1**. The crude was purified by column chromatography to afford the title compound **S1v** as a colorless oil in 49% yield after two steps (2.45 g, 9.8 mmol).

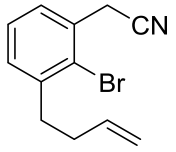

Rf: 0.27 (Hexane/EtOAc 100:1). **^{1}H NMR (CDCl$_3$, 400 MHz):** δ 7.37 (dd, 1H, J = 7.4 Hz, 0.9 Hz), 7.27 (t, 1H, J = 7.6 Hz), 7.21 (dd, 1H, J = 7.6 Hz, 1.2 Hz), 5.86 (ddt, 1H, J = 17.0 Hz, 10.3 Hz, 6.7 Hz), 5.06 (dm, 1H, J = 17.1 Hz), 5.01 (dm, 1H, J = 10.2 Hz), 3.86 (s, 2H), 2.88 (m, 2H), 2.37 (m, 2H). **^{13}C NMR (CDCl$_3$, 100 MHz):** δ 142.5, 137.2, 130.5, 130.2, 127.6, 127.4, 125.6, 117.1, 115.4, 36.2, 33.5, 25.7. **IR (CH$_2$Cl$_2$, cm^{-1})** ν_{max}: 3076, 2997, 2933, 2251, 1640, 1588, 1430, 1025, 915, 780, 719. **MS (EI 70 eV, *m/z*, %)** 249 (M$^+$, 1), 223 (22), 208 (100), 170 (47), 129 (32), 102 (33); **HRMS (EI$^+$)** *m/z*: [M]$^+$ Calcd for $C_{12}H_{12}NBr$ 249.0153; Found 249.0156.

Synthesis of α-alkyl- and α,α-bis(alkyl)nitriles

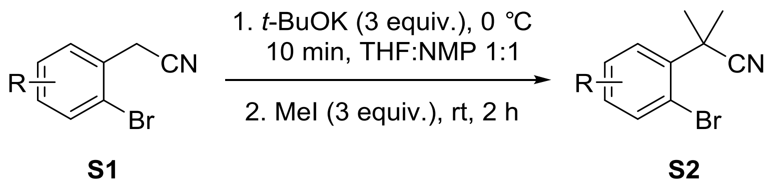

General procedure for the alkylation (or arylation) of 2-bromophenylacetonitrile derivatives (**GP2**): A flame-dried Schlenk flask was loaded with anhydrous THF (20 mL) and anhydrous NMP (20 mL) under an argon atmosphere. The appropriate 2-bromophenylacetonitrile derivative **S1** (10 mmol, 1 equiv.) was dissolved and the solution was cooled down to 0 °C. Then *t*-BuOK (3.366 g, 30 mmol, 3 equiv.) was added to the reaction mixture which was allowed to stir at 0 °C for 10 min (note: Sometimes the reaction mixture could solidify but it did not affect the final yield). Then alkyl halide (30 mmol, 3 equiv.) was slowly added. The resulting mixture was warmed to room temperature and stirred for another 2 h. Then, it was quenched by the addition of saturated aqueous solution of NH_4Cl (50 mL) and the resulting mixture was transferred into a separatory funnel and extracted with EtOAc (3 x 50 mL). The combined organic phases were washed with brine (3 x 50 mL) and dried over anhydrous Na_2SO_4. The solution was filtered, concentrated under reduced pressure and the product **S2** purified by column chromatography (silica gel, hexanes/EtOAc).

2-(2-Bromo-4-fluorophenyl)-2-methylpropanenitrile (S2b)

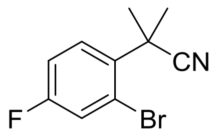

Following **GP2** from 2-(2-bromo-4-fluorophenyl)acetonitrile.[4] The crude was purified by column chromatography to afford the title compound as a white solid in 76% yield (1.84 g, 7.6 mmol).

mp 83-85 °C. ***Rf:*** 0.43 (Hexane/EtOAc 10:1). **^{1}H NMR (CDCl$_3$, 400 MHz):** δ 7.45 (dd, 1H, $^3J_{HH}$ = 8.9 Hz, $^4J_{HF}$ = 5.7 Hz), 7.41 (dd, 1H, $^3J_{HF}$ = 8.0 Hz, $^4J_{HH}$ = 2.7 Hz), 7.05 (ddd, 1H, $^3J_{HH}$ = 8.9 Hz, $^3J_{HF}$ = 7.4 Hz, $^4J_{HH}$ = 2.7 Hz), 1.87 (s, 6H). **^{13}C NMR (CDCl$_3$, 100 MHz):** δ 161.6 (d, $^1J_{CF}$ = 252.5 Hz), 134.3 (d, $^4J_{CF}$ = 3.7 Hz), 128.3 (d, $^3J_{CF}$ = 8.6 Hz), 123.1, 122.8 (d, $^3J_{CF}$ = 8.8 Hz), 122.7 (d, $^2J_{CF}$ = 24.9 Hz), 114.7 (d, $^2J_{CF}$ = 20.7 Hz), 36.9, 27.6. **^{19}F NMR (CDCl$_3$, 376 MHz):** δ -112.38 (m). **IR (CH$_2$Cl$_2$, cm^{-1})** ν_{max}: 2986, 2882, 2234, 1591, 1479, 1389, 1242, 1111, 1026, 860, 816. **MS (EI 70 eV, *m/z*, %)** 241 (M^+, 67), 226 (100), 199 (97), 182 (19), 147 (31), 120 (66); **HRMS (EI$^+$)** *m/z*: $[M]^+$ Calcd for $C_{10}H_9NFBr$ 240.9902; Found 240.9905.

2-(2-Bromo-4-*tert*-butylphenyl)-2-methylpropanenitrile (S2c)

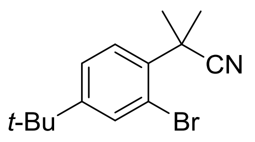

Following **GP2** from 2-(2-bromo-4-*tert*-butylphenyl)acetonitrile **(S1a)**. The crude was purified by column chromatography to afford the title compound as a pale-yellow solid in 88% yield (1.88 g, 6.7 mmol).

mp 62-64 °C. ***Rf:*** 0.50 (Hexane/EtOAc 10:1). **^{1}H NMR (CDCl$_3$, 400 MHz):** δ 7.64 (d, 1H, J = 2.1 Hz), 7.40 (d, 1H, J = 8.4 Hz), 7.33 (dd, 1H, J = 8.4 Hz, 2.1 Hz), 1.88 (s, 6H), 1.30 (s, 9H). **^{13}C NMR (CDCl$_3$, 100 MHz):** δ 152.9, 135.0, 132.6, 126.8, 124.8, 123.4, 122.2, 36.9, 34.3, 30.9, 27.4. **IR (CH$_2$Cl$_2$, cm^{-1})** ν_{max}: 2964, 2905, 2871, 2234, 1601, 1485, 1383, 1239, 1032, 826, 640. **MS (EI 70 eV, *m/z*, %)** 279 (M^+, 30), 264 (100), 186 (20), 115 (18), 42 (21); **HRMS (EI$^+$)** *m/z*: $[M]^+$ Calcd for $C_{14}H_{18}NBr$ 279.0623; Found 279.0620.

Methyl 3-bromo-4-(2-cyanopropan-2-yl)benzoate (S2d)

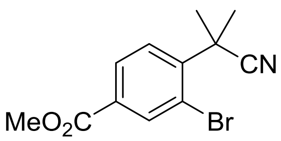

Following **GP2** from methyl 3-bromo-4-(cyanomethyl)benzoate.[5] The crude was purified by column chromatography to afford the title compound as a white solid in 45% yield (1.23 g, 4.5 mmol).

mp 87-89 °C. ***Rf:*** 0.33 (Hexane/EtOAc 5:1). **^{1}H NMR ($CDCl_3$, 400 MHz):** δ 8.30 (d, 1H, J = 1.8 Hz), 7.97 (dd, 1H, J = 8.3 Hz, 1.8 Hz), 7.55 (d, 1H, J = 8.3 Hz), 3.92 (s, 6H), 1.90 (s, 6H). **^{13}C NMR ($CDCl_3$, 100 MHz):** δ 165.0, 142.8, 136.5, 131.4, 128.8, 127.3, 122.7, 122.4, 52.5, 37.6, 27.3. **IR (CH_2Cl_2, cm^{-1})** ν_{max}: 2987, 2952, 2236, 1728, 1436, 1382, 1296, 1260, 1123, 1035, 766. **MS (EI 70 eV, *m/z*, %)** 281 (M^+, 88), 266 (100), 250 (80), 239 (82), 143 (35), 115 (50); **HRMS (EI$^+$)** *m/z*: $[M]^+$ Calcd for $C_{12}H_{12}NO_2Br$ 281.0051; Found 281.0050.

2-(2-Bromo-5-trifluoromethylphenyl)-2-methylpropanenitrile (S2f)

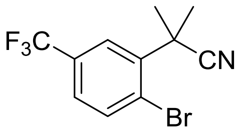

Following **GP2** from 2-(2-bromo-5-(trifluoromethyl)phenyl)acetonitrile.[6] The crude was purified by column chromatography to afford the title compound as a yellow oil in 73% yield (2.13 g, 7.3 mmol).

Rf: 0.38 (Hexane/EtOAc 20:1). **^{1}H NMR ($CDCl_3$, 400 MHz):** δ 7.81 (dm, 1H, J = 8.3 Hz), 7.68 (m, 1H), 7.45 (dm, 1H, J = 8.3 Hz), 1.92 (s, 6H). **^{13}C NMR ($CDCl_3$, 100 MHz):** δ 139.4, 136.2, 130.4 (q, $^2J_{CF}$ = 33.2 Hz), 126.6, 126.2 (q, $^3J_{CF}$ = 3.7 Hz), 124.0 (q, $^3J_{CF}$ = 3.9 Hz), 123.4 (q, $^1J_{CF}$ = 272.5 Hz), 122.4, 37.2, 27.3. **^{19}F NMR ($CDCl_3$, 376 MHz):** δ -62.79 (s). **IR (CH_2Cl_2, cm^{-1})** ν_{max}: 2988, 2943, 2235, 1609, 1470, 1406, 1329, 1175, 1128, 1028, 831. **MS (EI 70 eV, *m/z*, %)** 291 (M^+, 77), 276 (100), 249 (93), 176 (30), 170 (59), 115 (24); **HRMS (EI$^+$)** *m/z*: $[M]^+$ Calcd for $C_{11}H_9NF_3Br$ 290.9870; Found 290.9874.

2-(2-Bromo-6-fluorophenyl)-2-methylpropanenitrile (S2h)

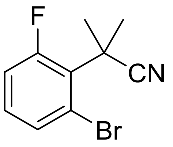

Following **GP2** from 2-(2-bromo-6-fluorophenyl)acetonitrile.[7] The crude was purified by column chromatography to afford the title compound as a colorless oil in 92% yield (2.23 g, 9.2 mmol).

Rf: 0.43 (Hexane/EtOAc 20:1). **^{1}H NMR ($CDCl_3$, 400 MHz):** δ 7.45 (m, 1H), 7.12 (td, 1H, $^3J_{HH}$ = 8.1 Hz, $^4J_{HF}$ = 5.4 Hz), 7.02 (ddm, 1H, $^3J_{HF}$ = 13.1 Hz, $^3J_{HH}$ = 8.3 Hz), 1.96 (d, 6H, $^5J_{HF}$ = 4.2 Hz). **^{13}C NMR ($CDCl_3$, 100 MHz):** δ 160.6 (d, $^1J_{CF}$ = 254.1 Hz), 131.5 (d, $^4J_{CF}$ = 3.8 Hz), 130.0 (d, $^3J_{CF}$ = 11.0 Hz), 125.7 (d, $^2J_{CF}$ = 12.0 Hz), 123.5 (d, $^3J_{CF}$ = 4.9 Hz), 123.3, 116.9 (d, $^2J_{CF}$ = 27.1 Hz), 37.1 (d, $^3J_{CF}$ = 3.7 Hz), 28.7 (d, $^4J_{CF}$ = 8.1 Hz). **^{19}F NMR ($CDCl_3$, 376 MHz):** δ -104.28 (m). **IR (CH_2Cl_2, cm^{-1})** ν_{max}: 3082, 2988, 2950, 2234, 1598, 1566, 1443, 1271, 1235, 865, 785. **HRMS (ESI$^+$)** *m/z*: $[M + Na]^+$ Calcd for $C_{10}H_9NFNaBr$ 263.9800; Found 263.9796.

2-(2-Bromophenyl)-2-methyl-3-phenylpropanenitrile (S2n)

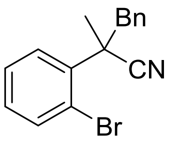

Following slightly modified **GP2** from 2-(2-bromophenyl)propanenitrile[8] and benzyl bromide (1.2 equiv.) as substrates. The crude was purified by column chromatography to afford the title compound as a colorless solid in 88% yield (2.63 g, 8.8 mmol).

mp 82-84 °C. ***Rf:*** 0.42 (Hexane/EtOAc 50:1). **^{1}H NMR ($CDCl_3$, 400 MHz):** δ 7.71 (dd, 1H, J = 7.8 Hz, 1.5 Hz), 7.34 (dd, 1H, J = 7.8 Hz, 1.8 Hz), 7.23 (m, 4H), 7.18 (td, 1H, J = 7.5 Hz, 1.8 Hz), 7.11 (m, 2H), 3.70 (d, 1H, J = 13.6 Hz), 3.33 (d, 1H, J = 13.6 Hz), 1.92 (s, 3H). **^{13}C NMR ($CDCl_3$, 100 MHz):** δ 136.5, 135.7, 135.0, 130.3, 129.6, 129.3, 128.1, 127.7, 127.3, 122.4, 121.9, 44.1, 43.3, 24.9. **IR (CH_2Cl_2, cm^{-1})** ν_{max}: 3062, 3030, 2985, 2936, 2234, 1809, 1496, 1468, 1021, 765, 704. **MS (EI 70 eV, *m/z*, %)** 299 (M^+, 17),

181 (8), 102 (12), 91 (100), 65 (17); **HRMS (EI$^+$)** *m/z*: $[M]^+$ Calcd for $C_{16}H_{14}NBr$ 299.0310; Found 299.0308.

2-(2-Bromophenyl)hexanenitrile (S2o)

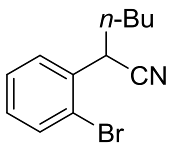

Following slightly modified **GP2** from (2-bromophenyl)acetonitrile and *n*-butyl iodide (1 equiv.) as substrates. The crude was purified by column chromatography to afford the title compound as a colorless oil in 72% yield (1.82 g, 7.2 mmol).

Rf: 0.39 (Hexane/EtOAc 100:1). **^{1}H NMR (CDCl$_3$, 400 MHz):** δ 7.59 (m, 2H), 7.38 (t, 1H, J = 7.5 Hz), 7.19 (m, 1H), 4.29 (dd, 1H, J = 9.1 Hz, 5.4 Hz), 1.88 (m, 2H), 1.56 (m, 2H), 1.41 (m, 2H), 0.95 (t, 3H, J = 7.3 Hz). **^{13}C NMR (CDCl$_3$, 100 MHz):** δ 135.5, 133.2, 129.5, 128.8, 128.1, 122.8, 120.2, 37.0, 34.0, 29.0, 21.9, 13.6. **IR (CH_2Cl_2, cm^{-1})** ν_{max}: 3060, 2958, 2931, 2862, 2242, 1570, 1472, 1440, 1025, 755, 682. **MS (EI 70 eV, *m/z*, %)** 253 (32), 251 (M^+, 33), 197 (99), 195 (100), 115 (40), 58 (44); **HRMS (EI$^+$)** *m/z*: $[M]^+$ Calcd for $C_{12}H_{14}NBr$ 251.0310; Found 251.0314.

2-(2-Bromophenyl)-2-(4-nitrophenyl)hexanenitrile (S2p)

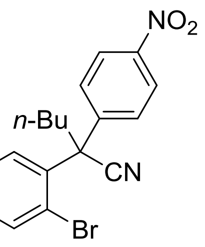

Following slightly modified **GP2** from 2-(2-bromophenyl)hexanenitrile (**S2o**) and 4-fluoronitrobenzene (1.2 equiv.) as substrates. The crude was purified by column chromatography to afford the title compound as a orange solid in 67% yield (1.79 g, 4.8 mmol).

mp 116-118 °C. ***Rf:*** 0.35 (Hexane/EtOAc 10:1). **^{1}H NMR (CDCl$_3$, 400 MHz):** δ 8.16 (m, 2H), 7.75 (m, 1H), 7.59 (m, 1H), 7.45 (m, 3H), 7.28 (m, 1H), 2.66 (m, 1H), 2.26 (m, 1H), 1.53 (m, 1H), 1.38 (m, 2H), 1.23 (m, 1H), 0.89 (m, 3H). **^{13}C NMR (CDCl$_3$, 100 MHz):** δ 147.1, 146.7, 136.4, 135.9, 130.5, 128.9, 128.1, 127.8, 124.2, 123.7, 119.6, 51.8, 39.7, 27.3, 22.5, 13.7. **IR (CH_2Cl_2, cm^{-1})** ν_{max}: 3078, 2958, 2932, 2871, 2237, 1596, 1522, 1346, 849, 753, 699. **MS (EI 70 eV, *m/z*, %)** 372 (M^+, 15), 316 (100), 190 (63), 58 (43), 42 (40); **HRMS (EI$^+$)** *m/z*: $[M]^+$ Calcd for $C_{18}H_{17}N_2O_2Br$ 372.0473; Found 372.0476.

2-(2-bromo-3-(but-3-en-1-yl)phenyl)-2-methylpropanenitrile (S2v)

Following **GP2** from nitrile **S1v**. The crude was purified by column chromatography to afford the title compound as a colorless oil in 76% yield (1.84 g, 7.6 mmol).

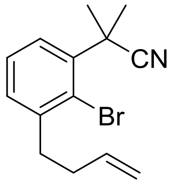

Rf: 0.24 (Hexane/EtOAc 50:1). **^{1}H NMR (CDCl$_3$, 400 MHz):** δ 7.29 (dd, 1H, J = 7.6 Hz, 2.2 Hz), 7.25 (t, 1H, J = 7.5 Hz), 7.22 (dd, 1H, J = 7.3 Hz, 2.2 Hz), 5.88 (ddt, 1H, J = 17.0 Hz, 10.3 Hz, 6.6 Hz), 5.07 (dm, 1H, J = 17.1 Hz), 5.00 (dm, 1H, J = 10.2 Hz), 2.92 (m, 2H), 2.39 (m, 2H), 1.89 (s, 6H). **^{13}C NMR (CDCl$_3$, 100 MHz):** δ 143.6, 138.8, 137.3, 130.2, 127.3, 125.0, 124.8, 123.6, 115.2, 37.6, 36.7, 33.6, 28.0. **IR (CH_2Cl_2, cm^{-1})** ν_{max}: 3075, 2981, 2936, 2873, 2233, 1640, 1459, 1411, 1021, 914, 791. **MS (EI 70 eV, *m/z*, %)** 277 (M^+, 1), 236 (100), 198 (32), 171 (54), 156 (78), 115 (37); **HRMS (EI$^+$)** *m/z*: $[M]^+$ Calcd for $C_{14}H_{16}NBr$ 277.0466; Found 277.0469.

2-(2-Bromo-5-(diethylamino)phenyl)-2-methylpropanenitrile (S2g)

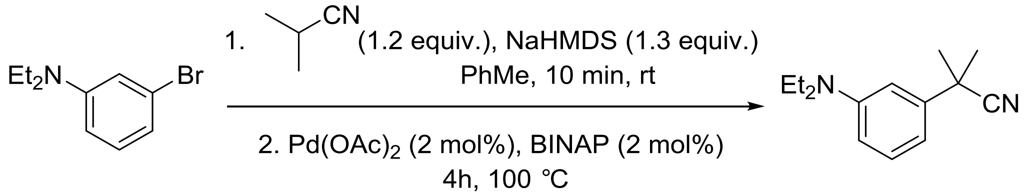

Synthesis of 2-(3-(diethylamino)phenyl)-2-methylpropanenitrile:

The nitrile was synthesized according to a literature procedure.[9] Under Ar atmosphere, a flame-dried Schlenk flask was charged with a solution of NaHMDS (0.6 M in toluene, 21.7 mL, 13 mmol, 1.3 equiv.). Isobutyronitrile (0.829 g, 12 mmol, 1.2 equiv.) was added to the solution and the resulting mixture was stirred for 10 min. Then it was transferred to a flame-dried Schlenk flask containing a stirred suspension of $Pd(OAc)_2$ (44.9 mg, 0.2 mmol, 0.02 equiv.), BINAP (124.5 mg, 0.2 mmol, 0.02 equiv.), and 3-bromo-*N*,*N*-diethylaniline (2.281 g, 10 mmol, 1 equiv.) in toluene (50 mL). The reaction mixture was stirred for 4 h at 100 °C and cooled down to room temperature before it was quenched with aqueous NH_4Cl solution (20 mL). The reaction mixture was poured into water and extracted with EtOAc (2 x 50 mL). The organic extracts were dried over anhydrous Na_2SO_4, filtered and concentrated.

The crude was purified by column chromatography to afford **2-(3-(diethylamino)phenyl)-2-methylpropanenitrile** as a pale-yellow oil in 82% yield (1.770 g, 8.2 mmol).

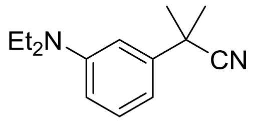

Rf: 0.33 (Hexane/EtOAc 20:1). **^{1}H NMR ($CDCl_3$, 400 MHz):** δ 7.22 (t, 1H, J = 8.0 Hz), 6.81 (t, 1H, J = 2.2 Hz), 6.69 (m, 1H), 6.63 (dd, 1H, J = 8.4 Hz, 2.5 Hz), 3.39 (q, 4H, J = 7.1 Hz), 1.73 (s, 6H), 1.19 (t, 6H, J = 7.1 Hz). **^{13}C NMR ($CDCl_3$, 100 MHz):** δ 148.0, 142.4, 129.7, 124.8, 111.4, 111.0, 108.6, 44.3, 37.4, 29.1, 12.5. **IR (CH_2Cl_2, cm^{-1})** ν_{max}: 2975, 2933, 2872, 2235, 1602, 1502, 1357, 1275, 1020, 772, 699. **MS (EI 70 eV, *m/z*, %)** 216 (M^+, 49), 201 (100), 173 (18), 157 (15), 146 (13), 77 (10); **HRMS (EI$^+$)** *m/z*: $[M]^+$ Calcd for $C_{14}H_{20}N_2$ 216.1626; Found 216.1631.

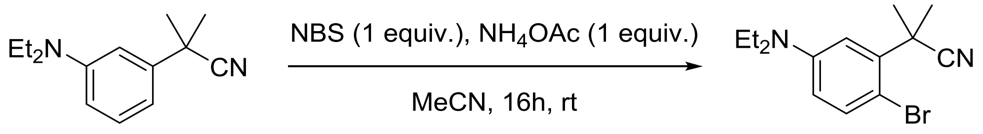

S2g

Synthesis of 2-(2-Bromo-5-(diethylamino)phenyl)-2-methylpropanenitrile (S2g)

A 100 mL round-bottom flask was charged with 2-(3-(diethylamino)phenyl)-2-methylpropanenitrile (1.770 g, 8.2 mmol, 1 equiv.), MeCN (41 mL) and NH_4OAc (632 mg, 8.2 mmol, 1 equiv.). To the resulting suspension was added NBS (1.459 g, 8.2 mmol, 1 equiv.) at room temperature and the resulting mixture was stirred for 18 h. After that time, the solution was transferred into a separatory funnel, diluted with H_2O (ca. 100 mL) and extracted with EtOAc (2 x 50 mL). The combined organic phases were dried over anhydrous Na_2SO_4. The solution was filtered and concentrated under reduced pressure.

The crude was purified by column chromatography to afford the title compound as a white solid in 95% yield (2.290 g, 7.8 mmol).

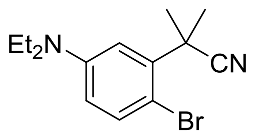

mp 66-68 °C. ***Rf:*** 0.22 (Hexane/EtOAc 20:1). **^{1}H NMR ($CDCl_3$, 400 MHz):** δ 7.38 (d, 1H, J = 8.9 Hz), 6.82 (d, 1H, J = 3.0 Hz), 6.47 (dd, 1H, J = 8.9 Hz, 3.0 Hz), 3.34 (q, 4H, J = 7.1 Hz), 1.89 (s, 6H), 1.17 (t, 6H, J = 7.1 Hz). **^{13}C NMR ($CDCl_3$, 100 MHz):** δ 147.1, 138.2, 135.9, 123.9, 112.5, 110.8, 105.9, 44.4, 38.1, 27.2, 12.3. **IR (CH_2Cl_2, cm^{-1})** ν_{max}: 2973, 2933, 2873, 2233, 1596, 1491, 1356, 1266, 1200, 1076, 799. **MS (EI 70 eV, *m/z*, %)** 294 (M^+, 40), 279 (100), 251 (16), 201 (23), 143 (8), 115 (9); **HRMS (EI^+)** *m/z*: $[M]^+$ Calcd for $C_{14}H_{19}N_2Br$ 294.0732; Found 294.0725.

Synthesis of aldehydes

All known aldehydes were synthesized from the corresponding nitriles according to **GP3** and their ^{1}H NMR spectra were consistent with those reported in the literature.[1]

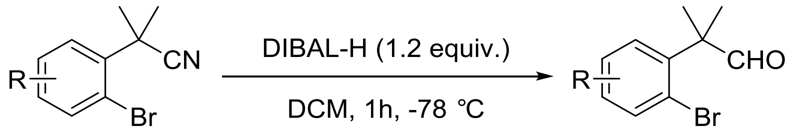

S2 **S3**

General procedure for the reduction of nitrile derivatives (GP3):

A flame-dried Schlenk flask was loaded with anhydrous DCM (20 mL) and the nitrile substrate (5 mmol, 1 equiv.) under argon atmosphere. The solution was cooled down to -78 °C and DIBAL-H (1M solution in hexanes, 6 mL, 6 mmol, 1.2 equiv.) was added dropwise. The reaction mixture was stirred at -78 °C for 1 h and slowly quenched with MeOH (5 mL) and 2M HCl (10 mL). Resulting mixture was warmed to room temperature, transferred into a separatory funnel and diluted with DCM (10 mL) and water (10 mL). The aqueous phase was extracted with DCM (2 x 15 mL). The combined organic phases were washed with brine (3 x 50 mL) and dried over anhydrous Na_2SO_4. The solution was filtered and volatiles were removed under reduced pressure. The obtained crude product was purified by column chromatography (silica gel, hexanes/EtOAc).

2-(2-Bromo-4-fluorophenyl)-2-methylpropanal (S3b)

Following **GP3** from nitrile **S2b**. The crude was purified by column chromatography to afford the title compound as a colorless oil in 50% yield (613 mg, 2.5 mmol).

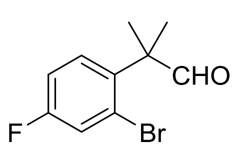

Rf: 0.36 (Hexane/EtOAc 20:1). **^{1}H NMR ($CDCl_3$, 400 MHz):** δ 9.74 (s, 1H), 7.36 (m, 2H), 7.08 (m, 1H), 1.49 (s, 6H). **^{13}C NMR ($CDCl_3$, 100 MHz):** δ 202.7, 161.5 (d, $^1J_{CF}$ = 250.1 Hz), 138.3 (d, $^4J_{CF}$ = 4.0 Hz), 129.6 (d, $^3J_{CF}$ = 8.5 Hz), 123.3 (d, $^3J_{CF}$ = 9.4 Hz), 121.6 (d, $^2J_{CF}$ = 24.2 Hz), 114.7 (d, $^2J_{CF}$ = 20.4 Hz), 51.3, 23.3. **^{19}F NMR ($CDCl_3$, 376 MHz):** δ -113.33 (m). **IR (CH_2Cl_2, cm^{-1})** ν_{max}: 3414, 2984, 2810, 1721, 1595, 1483, 1358, 1244, 1207, 864, 824. **HRMS ($APCI^+$)** *m/z*: $[M + H]^+$ Calcd for $C_{10}H_{11}OFBr$ 244.9977; Found 244.9978.

2-(2-Bromo-4-*tert*-butylphenyl)-2-methylpropanal (S3c)

Following **GP3** from nitrile **S2c**. The crude was purified by column chromatography to afford the title compound as a colorless oil in 56% yield (793 mg, 2.8 mmol).

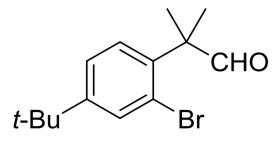

Rf: 0.33 (Hexane/EtOAc 50:1). **^{1}H NMR ($CDCl_3$, 400 MHz):** δ 9.77 (s, 1H), 7.58 (d, 1H, J = 2.0 Hz), 7.37 (dd, 1H, J = 8.3 Hz, 2.0 Hz), 7.33 (d, 1H, J = 8.3 Hz), 1.50 (s, 6H), 1.31 (s, 9H). **^{13}C NMR ($CDCl_3$, 100 MHz):** δ 203.3, 152.5, 139.1, 131.5, 128.1, 124.9, 123.3, 51.3, 34.4, 31.1, 23.2. **IR (CH_2Cl_2, cm^{-1})** ν_{max}: 3434,

2965, 2871, 2801, 2703, 1726, 1485, 1385, 1032, 835, 622. **HRMS (ESI^+)** *m/z*: $[M + Na]^+$ Calcd for $C_{14}H_{19}ONaBr$ 305.0517; Found 305.0507.

2-(2-Bromo-5-chlorophenyl)-2-methylpropanal (S3e)

Following **GP3** from 2-(2-bromo-5-chlorophenyl)-2-methylpropanenitrile.[2] The crude was purified by column chromatography to afford the title compound as a yellow oil in 73% yield (968 mg, 3.7 mmol).

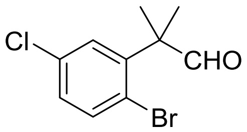

Rf: 0.51 (Hexane/EtOAc 20:1). **^{1}H NMR ($CDCl_3$, 400 MHz):** δ 9.74 (s, 1H), 7.50 (d, 1H, J = 8.5 Hz), 7.36 (d, 1H, J = 2.5 Hz), 7.15 (dd, 1H, J = 8.5 Hz, 2.5 Hz), 1.49 (s, 6H). **^{13}C NMR ($CDCl_3$, 100 MHz):** δ 202.0, 144.2, 135.4, 134.0, 129.0, 129.0, 121.1, 51.8, 23.0 **IR (CH_2Cl_2, cm^{-1})** ν_{max}: 3433, 2979, 2937, 2807, 1728, 1454, 1382, 1234, 1107, 1022, 813. **HRMS ($APCI^+$)** *m/z*: $[M + H]^+$ Calcd for $C_{10}H_{11}OClBr$ 260.9682; Found 260.9688.

2-(2-Bromo-5-trifluoromethylphenyl)-2-methylpropanal (S3f)

Following **GP3** from nitrile **S2f**. The crude was purified by column chromatography to afford the title compound as a colorless oil in 75% yield (1.11 g, 3.75 mmol).

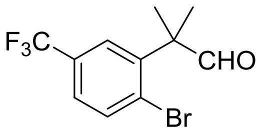

Rf: 0.38 (Hexane/EtOAc 20:1). **^{1}H NMR ($CDCl_3$, 400 MHz):** δ 9.78 (s, 1H), 7.73 (dm, 1H, J = 8.3 Hz), 7.64 (m, 1H), 7.44 (dm, 1H, J = 8.3 Hz), 1.55 (s, 6H). **^{13}C NMR ($CDCl_3$, 100 MHz):** δ 201.9, 143.6, 135.1, 130.3 (q, $^2J_{CF}$ = 32.7 Hz), 127.3 (q, $^4J_{CF}$ = 1.5 Hz), 125.8 (q, $^3J_{CF}$ = 3.7 Hz), 125.5 (q, $^3J_{CF}$ = 3.7 Hz), 123.7 (q, $^1J_{CF}$ = 272.3 Hz), 51.9, 23.1. **^{19}F NMR ($CDCl_3$, 376 MHz):** δ -62.72 (s). **IR (CH_2Cl_2, cm^{-1})** ν_{max}: 3424, 2988, 2940, 1720, 1607, 1332, 1329, 1173, 1130, 1023, 827. **HRMS ($APCI^+$)** *m/z*: $[M + H]^+$ Calcd for $C_{11}H_{11}OF_3Br$ 294.9945; Found 294.9941.

2-(2-Bromo-5-(diethylamino)phenyl)-2-methylpropanal (S3g)

Following **GP3** from nitrile **S2g**. The crude was purified by column chromatography to afford the title compound as a yellow oil in 66% yield (786 mg, 3.3 mmol).

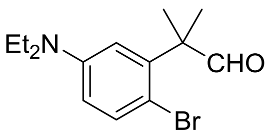

Rf: 0.30 (Hexane/EtOAc 20:1). **^{1}H NMR ($CDCl_3$, 400 MHz):** δ 9.79 (s, 1H), 7.34 (d, 1H, J = 8.9 Hz), 6.67 (d, 1H, J = 3.1 Hz), 6.49 (dd, 1H, J = 8.9 Hz, 3.1 Hz), 3.35 (q, 4H, J = 7.1 Hz), 1.49 (s, 6H), 1.17 (t, 6H, J = 7.1 Hz). **^{13}C NMR ($CDCl_3$, 100 MHz):** δ 203.7, 147.4, 142.4, 134.7, 112.5, 111.8, 107.8, 51.8, 44.6, 23.2, 12.4. **IR (CH_2Cl_2, cm^{-1})** ν_{max}: 2971, 2932, 2871, 2702, 1725, 1593, 1487, 1357, 1265, 1200, 797. **MS (EI 70 eV, *m/z*, %)** 297 (M^+, 72), 282 (100), 218 (36), 190 (50), 174 (62), 146 (27); **HRMS (EI^+)** *m/z*: $[M]^+$ Calcd for $C_{14}H_{20}NOBr$ 297.0728; Found 297.0716.

2-(2-Bromo-6-fluorophenyl)-2-methylpropanal (S3h)

Following **GP3** from nitrile **S2h**. The crude was purified by column chromatography to afford the title compound as a colorless oil in 79% yield (980 mg, 4.0 mmol).

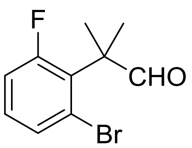

Rf: 0.46 (Hexane/EtOAc 20:1). **^{1}H NMR ($CDCl_3$, 400 MHz):** δ 9.78 (d, 1H, $^5J_{HF}$ = 2.5 Hz), 7.39 (dm, 1H, $^3J_{HH}$ = 7.9 Hz), 7.11 (td, 1H, $^3J_{HH}$ = 8.1 Hz, $^4J_{HF}$ = 5.6 Hz), 7.03 (ddd, 1H, $^3J_{HF}$ = 12.5 Hz, $^3J_{HH}$ = 8.3 Hz, $^4J_{HH}$ = 1.5 Hz), 1.58 (d, 6H, $^5J_{HF}$ = 3.3 Hz). **^{13}C NMR ($CDCl_3$, 100 MHz):** δ 200.9 (d, $^4J_{CF}$ = 1.1 Hz), 162.3 (d, $^1J_{CF}$ = 250.6 Hz), 130.7 (d, $^4J_{CF}$ = 3.1 Hz), 129.9 (d, $^2J_{CF}$ = 13.3 Hz), 129.5 (d, $^3J_{CF}$ = 10.4 Hz), 123.6 (d, $^3J_{CF}$ = 6.1 Hz), 116.5 (d, $^2J_{CF}$ = 26.5

Hz), 51.9 (d, $^3J_{CF}$ = 3.4 Hz), 23.1 (d, $^4J_{CF}$ = 7.4). **^{19}F NMR (CDCl$_3$, 376 MHz):** δ -104.75 (m). **IR (CH_2Cl_2, cm^{-1})** ν_{max}: 3433, 2984, 2810, 1721, 1599, 1564, 1439, 1267, 1238, 860, 783. **HRMS (APCI$^+$)** *m/z*: [M + H]$^+$ Calcd for $C_{10}H_{11}OFBr$ 244.9977; Found 244.9981.

2-(6-Bromobenzo[*d*][1,3]dioxol-5-yl)-2-methylpropanal (S3i)

Following **GP3** from 2-(6-bromobenzo[*d*][1,3]dioxol-5-yl)-2-methylpropanenitrile.[2] The crude was purified by column chromatography to afford the title compound as a white solid in 69% yield (949 mg, 3.5 mmol).

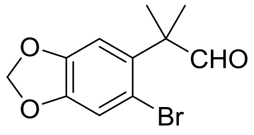

mp 76-78 °C. ***Rf:*** 0.36 (Hexane/EtOAc 10:1). **^{1}H NMR (CDCl$_3$, 400 MHz):** δ 9.70 (s, 1H), 7.01 (s, 1H), 6.89 (s, 1H), 5.97 (s, 2H), 1.44 (s, 6H). **^{13}C NMR (CDCl$_3$, 100 MHz):** δ 202.9, 147.8, 147.5, 135.6, 114.1, 113.7, 108.4, 101.9, 51.4, 23.4. **IR (CH_2Cl_2, cm^{-1})** ν_{max}: 2977, 2906, 2804, 2703, 1728, 1503, 1481, 1242, 1038, 929, 841. **MS (EI 70 eV, *m/z*, %)** 270 (M$^+$, 39), 241 (100), 213 (63), 191 (43), 162 (74); **HRMS (EI$^+$)** *m/z*: [M]$^+$ Calcd for $C_{11}H_{11}O_3Br$ 269.9892; Found 269.9884.

4-(2-Bromophenyl)tetrahydro-2*H*-pyran-4-carbaldehyde (S3m)

Following **GP3** from 4-(2-bromophenyl)tetrahydro-2*H*-pyran-4-carbonitrile.[2] The crude was purified by column chromatography to afford the title compound as a white solid in 44% yield (592 mg, 2.2 mmol).

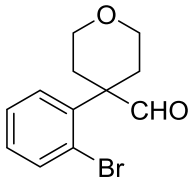

mp 54-56 °C. ***Rf:*** 0.33 (Hexane/EtOAc 5:1). **^{1}H NMR (CDCl$_3$, 400 MHz):** δ 9.80 (s, 1H), 7.60 (m, 1H), 7.44 (m, 1H), 7.38 (m, 1H), 7.18 (m, 1H), 3.88 (m, 2H), 3.80 (m, 2H), 2.40 (m, 2H), 2.18 (m, 2H). **^{13}C NMR (CDCl$_3$, 100 MHz):** δ 202.0, 140.2, 135.1, 129.3, 129.3, 127.8, 123.4, 64.0, 52.6, 30.9. **IR (CH_2Cl_2, cm^{-1})** ν_{max}: 3422, 2955, 2862, 2708, 1722, 1469, 1242, 1105, 1017, 758, 574. **HRMS (APCI$^+$)** *m/z*: [M + H]$^+$ Calcd for $C_{12}H_{14}O_2Br$ 269.0177; Found 269.0170.

2-(2-Bromophenyl)-2-methyl-3-phenylpropanal (S3n)

Following **GP3** from nitrile **S2n**. The crude was purified by column chromatography to afford the title compound as a colorless oil in 64% yield (970 mg, 3.2 mmol).

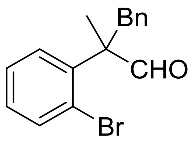

Rf: 0.29 (Hexane/EtOAc 50:1). **^{1}H NMR (CDCl$_3$, 400 MHz):** δ 9.91 (s, 1H), 7.67 (m, 1H), 7.19 (m, 2H), 7.10 (m, 3H), 6.87 (m, 1H), 6.68 (m, 2H), 3.66 (d, 1H, J = 13.6 Hz), 3.25 (d, 1H, J = 13.6 Hz), 1.33 (s, 3H). **^{13}C NMR (CDCl$_3$, 100 MHz):** δ 202.7, 139.7, 136.5, 134.3, 130.57, 130.61, 129.3, 127.6, 127.3, 126.3, 123.5, 56.0, 39.2, 20.7. **IR (CH_2Cl_2, cm^{-1})** ν_{max}: 3425, 3028, 2937, 2803, 1723, 1465, 1382, 1268, 1020, 766, 702. **MS (EI 70 eV, *m/z*, %)** 302 (M$^+$, 9), 223 (17), 183 (29), 178 (15), 91 (100), 44 (16); **HRMS (EI$^+$)** *m/z*: [M]$^+$ Calcd for $C_{16}H_{15}OBr$ 302.0306; Found 302.0306.

2-(2-Bromophenyl)hexanal (S3o)

Following **GP3** from nitrile **S2o**. The crude was purified by column chromatography to afford the title compound as a colorless oil in 72% yield (916 mg, 3.6 mmol).

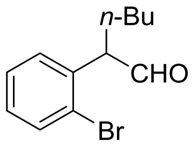

Rf: 0.24 (Hexane/EtOAc 100:1). **^{1}H NMR (CDCl$_3$, 400 MHz):** δ 9.70 (s, 1H), 7.63 (dd, 1H, J = 7.9 Hz, 0.9 Hz), 7.33 (m, 1H), 7.16 (td, 1H, J = 7.5 Hz, 1.7 Hz), 7.13 (dd, 1H, J = 7.7 Hz, 1.6 Hz), 4.13 (m, 1H), 2.12 (m, 1H), 1.73 (m, 1H), 1.29 (m, 4H), 0.88 (t, 3H, J = 7.1). **^{13}C NMR (CDCl$_3$, 100 MHz):** δ 200.1, 136.6, 133.4, 129.7, 129.0, 127.9,

125.8, 57.5, 29.15, 29.13, 22.6, 13.8. **IR (CH_2Cl_2, cm^{-1})** ν_{max}: 3398, 3065, 2956, 2931, 2871, 1707, 1468, 1439, 1025, 754, 676. **HRMS (APCI$^-$)** *m/z*: $[M + H]^-$ Calcd for $C_{12}H_{14}OBr$ 253.0228; Found 253.0227.

2-(2-Bromophenyl)-2-(4-nitrophenyl)hexanal (S3p)

Following **GP3** from nitrile **S2p**. The crude was purified by column chromatography to afford the title compound as a yellow oil in 81% yield (1.54 g, 4.1 mmol).

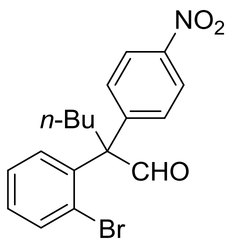

Rf: 0.40 (Hexane/EtOAc 10:1). **^{1}H NMR ($CDCl_3$, 400 MHz):** δ 9.97 (s, 1H), 8.15 (d, 2H, J = 9.0 Hz), 7.61 (dd, 1H, J = 7.9 Hz, 1.2 Hz), 7.44 (m, 3H), 7.37 (dd, 1H, J = 7.9 Hz, 1.7 Hz), 7.26 (td, 1H, J = 7.6 Hz, 1.7 Hz), 2.41 (m, 2H), 1.32 (m, 2H), 1.11 (m, 1H), 1.01 (m, 1H), 0.85 (t, 3H, J = 7.3 Hz). **^{13}C NMR ($CDCl_3$, 100 MHz):** δ 197.8, 147.5, 146.7, 139.5, 135.4, 130.9, 129.7, 129.0, 127.7, 124.4, 123.5, 63.6, 34.6, 26.9, 23.0, 13.7. **IR (CH_2Cl_2, cm^{-1})** ν_{max}: 3074, 2957, 2932, 2871, 1725, 1603, 1519, 1468, 1348, 854, 753. **HRMS (ESI$^+$)** *m/z*: $[M + Na]^+$ Calcd for $C_{18}H_{18}NO_3NaBr$ 398.0368; Found 398.0366.

2-(2-bromo-3-(but-3-en-1-yl)phenyl)-2-methylpropanal (S3v)

Following **GP3** from nitrile **S2v**. The crude was purified by column chromatography to afford the title compound as a colorless oil in 71% yield (1,00 g, 3.6 mmol).

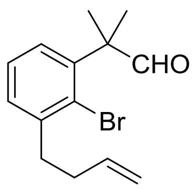

Rf: 0.38 (Hexane/EtOAc 20:1). **^{1}H NMR ($CDCl_3$, 400 MHz):** δ 9.80 (s, 1H), 7.27 (m, 2H), 7.20 (dd, 1H, J = 6.8 Hz, 2.4 Hz), 5.87 (ddt, 1H, J = 17.0 Hz, 10.3 Hz, 6.6 Hz), 5.06 (dm, 1H, J = 17.1 Hz), 5.00 (dm, 1H, J = 10.2 Hz), 2.87 (m, 2H), 2.37 (m, 2H), 1.52 (s, 6H). **^{13}C NMR ($CDCl_3$, 100 MHz):** δ 203.2, 143.2, 142.8, 137.5, 129.7, 127.5, 126.4, 125.7, 115.2, 52.3, 36.7, 33.7, 23.7. **IR (CH_2Cl_2, cm^{-1})** ν_{max}: 3075, 2981, 2936, 2873, 2233, 1640, 1459, 1411, 1021, 914, 791. **HRMS (APCI$^+$)** *m/z*: $[M + H]^+$ Calcd for $C_{14}H_{18}OBr$ 281.0541; Found 281.0540.

Methyl 3-bromo-4-(2-methyl-1-oxopropan-2-yl)benzoate (S3d)

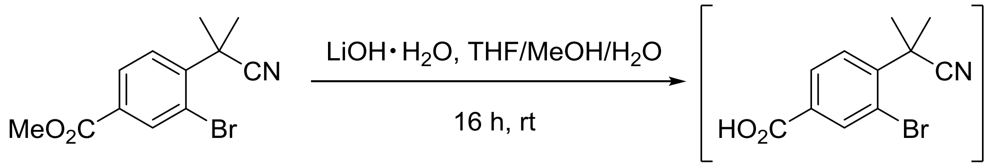

Methyl 3-bromo-4-(2-cyanopropan-2-yl)benzoate **S2d** (1.411 g, 5 mmol, 1 equiv.) was dissolved in the mixture of THF (15 mL), MeOH (5 mL) and H_2O (5 mL) followed by addition of LiOH · H_2O (629 mg, 15 mmol, 3 equiv.). The reaction mixture was stirred for 16 h at room temperature and acidified with 2M HCl (20 mL), then it was transferred into a separatory funnel and extracted with EtOAc (2 x 20 mL). Combined organic phases were dried over anhydrous Na_2SO_4, filtered and concentrated under reduced pressure. The resulting crude 3-bromo-4-(2-cyanopropan-2-yl)benzoic acid (1.193 g, 4.45 mmol, 89% yield) was used in the next step without further purification.

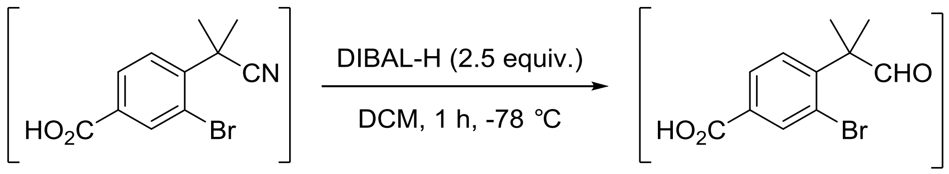

The crude 3-bromo-4-(2-cyanopropan-2-yl)benzoic acid (1.212 g, 4.45 mmol, 1 equiv.) was reduced following the slightly modified **GP3** using 2.5 equiv. of DIBAL-H (1M solution in hexanes, 11.1 mL, 11.1 mmol) instead of 1.2 equiv. The obtained crude 3-bromo-4-(2-methyl-1-oxopropan-2-yl)benzoic acid (0.965 g, 3.56 mmol, 80% yield) was used directly in the next step.

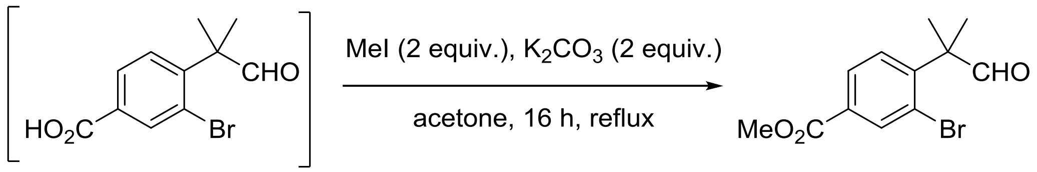

The crude 3-bromo-4-(2-methyl-1-oxopropan-2-yl)benzoic acid (0.965 g, 3.56 mmol) was dissolved in acetone (20 mL), followed by addition of methyl iodide (0.554 mL, 8.9 mmol, 2.5 equiv.) and K_2CO_3 (1.230 g, 8.9 mmol, 2.5 equiv.). The reaction mixture was stirred for 16 h at reflux (heating mantle). After cooling to room temperature the volatiles were removed under reduced pressure and the crude material was dissolved in EtOAc (30 mL) and washed with water (20 mL). The organic phase was dried over anhydrous Na_2SO_4, filtered, concentrated and purified by column chromatography to afford the title compound **S3d** as a colorless oil in 79% yield (798 mg, 2.8 mmol).

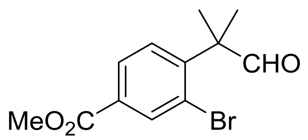

Rf: 0.40 (Hexane/EtOAc 5:1). **^{1}H NMR ($CDCl_3$, 400 MHz):** δ 9.75 (s, 1H), 8.21 (d, 1H, J = 1.8 Hz), 7.97 (dd, 1H, J = 8.2 Hz, 1.8 Hz), 7.46 (d, 1H, J = 8.2 Hz), 3.89 (s, 6H), 1.50 (s, 6H). **^{13}C NMR ($CDCl_3$, 100 MHz):** δ 201.9, 165.2, 147.2, 135.3, 130.9, 128.7, 128.6, 123.2, 52.3, 51.9, 23.0. **IR (CH_2Cl_2, cm^{-1})** ν_{max}: 2979, 2952, 2806, 1725, 1435, 1385, 1295, 1261, 1120, 1034, 767. **HRMS (ESI$^+$)** *m/z*: $[M + Na]^+$ Calcd for $C_{12}H_{13}O_3NaBr$ 306.9946; Found 306.9934.

Synthesis of aldonitrones

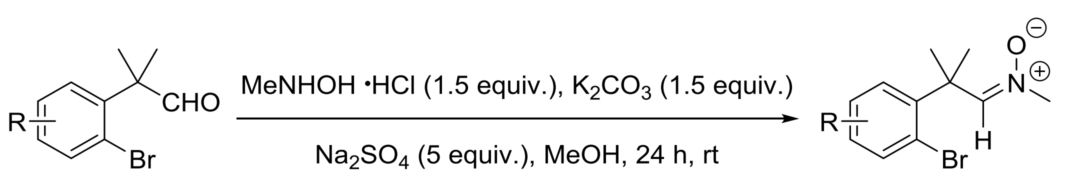

General procedure for the condensation of aldehyde derivatives with *N*-methylhydroxylamine hydrochloride (GP4):

A 50 mL round-bottom flask was charged with aldehyde (2 mmol, 1 equiv.), *N*-methylhydroxylamine hydrochloride (251 mg, 3 mmol, 1.5 equiv.), K_2CO_3 (414 mg, 3 mmol, 1.5 equiv), Na_2SO_4 (1.420 g, 10 mmol, 5 equiv.) and methanol (10 mL). Then resulting suspension was stirred at room temperature for 24 h. After that time, the reaction mixture was filtered and concentrated under reduced pressure. The crude material was diluted with DCM (ca. 30 mL), filtered through a pad of celite, evaporated under vacuum and purified by column chromatography (silica gel, hexanes/EtOAc).

(*Z*)-2-(2-Bromophenyl)-*N*,2-dimethylpropan-1-imine oxide (1a)

Following **GP4** from 2-(2-bromophenyl)-2-methylpropanal.[1] The crude was purified by column chromatography to afford the title compound as a white solid in 90% yield (461 mg, 1.8 mmol).

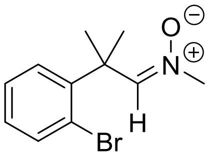

mp 75-77 °C. ***Rf:*** 0.41 (EtOAc/MeOH 5:1). **^{1}H NMR ($CDCl_3$, 400 MHz):** δ 7.50 (dd, 1H, J = 7.9 Hz, 1.2 Hz), 7.45 (dd, 1H, J = 8.0 Hz, 1.6 Hz), 7.29 (m, 1H), 7.04 (m, 1H), 6.92 (d, 1H, J = 0.6 Hz), 3.61 (d, 3H, J = 0.6 Hz), 1.66 (s, 6H). **^{13}C NMR ($CDCl_3$, 100 MHz):** δ 144.6, 144.4, 134.2, 127.8, 127.8, 127.6, 120.9, 52.6, 41.1, 24.5. **IR (CH_2Cl_2, cm^{-1})** ν_{max}: 3396, 3061, 2971, 2874, 1606, 1468, 1410, 1185, 1019, 971, 758. **HRMS (ESI$^+$)** *m/z*: $[M + H]^+$ Calcd for $C_{11}H_{15}NOBr$ 256.0337; Found 256.0339.

(*Z*)-2-(2-Bromo-4-fluorophenyl)-*N*,2-dimethylpropan-1-imine oxide (1b)

Following **GP4** from aldehyde **S3b**. The crude was purified by column chromatography to afford the title compound as a white solid in 66% yield (362 mg, 1.32 mmol).

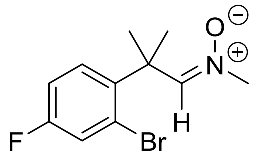

mp 97-99 °C. ***Rf:*** 0.27 (EtOAc/MeOH 10:1). **^{1}H NMR ($CDCl_3$, 400 MHz):** δ 7.40 (dd, 1H, $^3J_{HH}$ = 8.9 Hz, $^4J_{HF}$ = 6.0 Hz), 7.23 (dd, $^3J_{HF}$ = 8.2 Hz, $^4J_{HH}$ = 2.7 Hz), 7.00 (ddd, 1H, $^3J_{HH}$ = 8.9 Hz, $^3J_{HF}$ = 7.7 Hz, $^4J_{HH}$ = 2.7 Hz), 3.60 (s, 3H), 1.63 (s, 6H). **^{13}C NMR ($CDCl_3$, 100 MHz):** δ 160.5 (d, $^1J_{CF}$ = 249.3 Hz), 144.0, 140.5 (d, $^4J_{CF}$ = 3.6 Hz), 128.6 (d, $^3J_{CF}$ = 8.2 Hz), 121.1 (d, $^2J_{CF}$ = 24.35 Hz), 120.4 (d, $^3J_{CF}$ = 9.0 Hz), 114.3 (d, $^2J_{CF}$ = 20.3 Hz), 52.5, 40.5, 24.6. **^{19}F NMR ($CDCl_3$, 376 MHz):** δ -115.74 (m). **IR (CH_2Cl_2, cm^{-1})** ν_{max}: 3040, 2972, 2876, 1591, 1481, 1410, 1250, 1180, 1115, 1030, 862. **HRMS (ESI$^+$)** *m/z*: $[M + H]^+$ Calcd for $C_{11}H_{14}NOFBr$ 274.0243; Found 274.0241.

(*Z*)-2-(2-Bromo-4-*tert*-butylphenyl)-*N*,2-dimethylpropan-1-imine oxide (1c)

Following **GP4** from aldehyde **S3c**. The crude was purified by column chromatography to afford the title compound as a white solid in 86% yield (537 mg, 1.72 mmol).

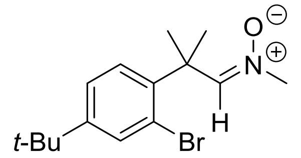

mp 142-144 °C. ***Rf:*** 0.40 (EtOAc/MeOH 10:1). **^{1}H NMR ($CDCl_3$, 400 MHz):** δ 7.47 (s, 1H), 7.35 (m, 1H), 7.28 (m, 1H), 6.91 (s, 1H), 3.60 (s, 3H), 1.65 (s, 6H), 1.26 (s, 9H). **^{13}C NMR ($CDCl_3$, 100 MHz):** δ 150.7, 144.4, 141.3, 131.1, 127.2, 124.6, 120.8, 52.6, 40.5, 34.1, 31.0, 24.5. **IR (CH_2Cl_2, cm^{-1})** ν_{max}**:** 3047, 2963, 2869, 1601, 1481, 1411, 1252, 1192, 1123, 1029, 818. **HRMS (ESI$^+$)** *m/z*: $[M + H]^+$ Calcd for $C_{15}H_{23}NOBr$ 312.0963; Found 312.0959.

(*Z*)-2-(2-Bromo-4-(methoxycarbonyl)phenyl)-*N*,2-dimethylpropan-1-imine oxide (1d)

Following **GP4** from aldehyde **S3d**. The crude was purified by column chromatography to afford the title compound as a white solid in 86% yield (540 mg, 1.72 mmol).

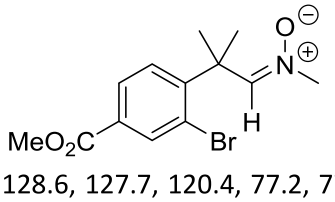

mp 126-128 °C. ***Rf:*** 0.23 (EtOAc/MeOH 10:1). **^{1}H NMR ($CDCl_3$, 500 MHz):** δ 8.17 (d, 1H, J = 1.9 Hz), 7.95 (dd, 1H, J = 8.3 Hz, 1.9 Hz, 1H), 7.53 (d, 1H, J = 8.3 Hz, 1H), 6.95 (s, 1H), 3.88 (s, 3H), 3.61 (s, 3H), 1.67 (s, 6H). **^{13}C NMR ($CDCl_3$, 125 MHz):** δ 165.6, 149.7, 143.5, 135.2, 129.5, 128.6, 127.7, 120.4, 77.2, 77.0, 76.8, 52.3, 52.1, 41.2, 24.4. **IR (CH_2Cl_2, cm^{-1})** ν_{max}: 3419, 2951, 2877, 1723, 1600, 1435, 1295, 1240, 1119, 972, 768. **MS (EI 70 eV, *m/z*, %)** 314 (M^+, 3), 234 (100), 193 (67), 165 (36), 115 (44), 43 (96); **HRMS (ESI$^+$)** *m/z*: $[M]^+$ Calcd for $C_{13}H_{17}NO_3Br$ 314.0392; Found 314.0386.

(*Z*)-2-(2-Bromo-5-chlorophenyl)-*N*,2-dimethylpropan-1-imine oxide (1e)

Following **GP4** from aldehyde **S3e**. The crude was purified by column chromatography to afford the title compound as a white solid in 78% yield (453 mg, 1.56 mmol).

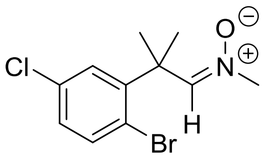

mp 138-140 °C. ***Rf:*** 0.30 (EtOAc/MeOH 10:1). **^{1}H NMR ($CDCl_3$, 400 MHz):** δ 7.40 (m, 2H), 7.02 (dd, 1H, J = 8.5 Hz, 2.4 Hz), 6.89 (s, 1H), 3.60 (s, 3H), 1.62 (s, 6H). **^{13}C NMR ($CDCl_3$, 100 MHz):** δ 146.4, 143.3, 135.0, 133.7, 128.1, 127.7, 118.4, 52.4, 41.0, 24.4. **IR (CH_2Cl_2, cm^{-1})** ν_{max}: 3401, 2971, 2874, 1601, 1461, 1410, 1188, 1107, 1019, 973, 808. **MS (EI 70 eV, *m/z*, %)** 290 (M^+, 2), 210 (79), 195 (33), 141 (34), 115 (36), 43 (100); **HRMS (ESI^+)** *m/z*: $[M + H]^+$ Calcd for $C_{11}H_{14}NOClBr$ 289.9947; Found 289.9949.

(*Z*)-2-(2-Bromo-5-trifluoromethylphenyl)-*N*,2-dimethylpropan-1-imine oxide (1f)

Following **GP4** from aldehyde **S3f**. The crude was purified by column chromatography to afford the title compound as a white solid in 90% yield (583 mg, 1.8 mmol).

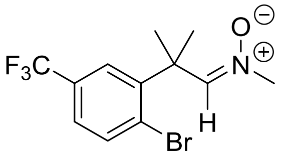

mp 144-146 °C. ***Rf:*** 0.30 (EtOAc/MeOH 10:1). **^{1}H NMR ($CDCl_3$, 500 MHz):** δ 7.69 (s, 1H), 7.63 (d, 1H, J = 7.8 Hz), 7.31 (d, 1H, J = 7.5 Hz), 6.95 (s, 1H), 3.62 (s, 3H), 1.69 (s, 6H). **^{13}C NMR ($CDCl_3$, 100 MHz):** δ 145.8, 143.3, 134.6, 130.2 (q, $^{2}J_{CF}$ = 27.6 Hz), 129.9 (q, $^{2}J_{CF}$ = 32.6 Hz), 124.6 (q, $^{3}J_{CF}$ = 4.6 Hz), 124.3 (q, $^{3}J_{CF}$ = 3.6 Hz), 123.9 (q, $^{1}J_{CF}$ = 271.4 Hz), 52.3, 41.1, 24.4. **^{19}F NMR ($CDCl_3$, 376 MHz):** δ -62.56 (s). **IR (CH_2Cl_2, cm^{-1})** ν_{max}: 3041, 2993, 2969, 1604, 1467, 1402, 1326, 1161, 1125, 1021, 824. **HRMS (ESI^+)** *m/z*: $[M + H]^+$ Calcd for $C_{12}H_{14}NOF_3Br$ 324.0211; Found 324.0196.

(*Z*)-2-(2-Bromo-5-(diethylamino)phenyl)-*N*,2-dimethylpropan-1-imine oxide (1g)

Following **GP4** from aldehyde **S3g**. The crude was purified by column chromatography to afford the title compound as a pale-yellow solid in 73% yield (478 mg, 1.46 mmol).

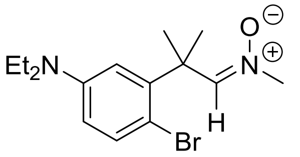

mp 100-102 °C. ***Rf:*** 0.30 (EtOAc/MeOH 10:1). **^{1}H NMR ($CDCl_3$, 400 MHz):** δ 7.27 (d, 1H, J = 8.8 Hz), 6.92 (s, 1H), 6.74 (d, 1H, J = 3.0 Hz), 6.38 (dd, 1H, J = 8.8 Hz, 3.0 Hz), 3.62 (s, 3H), 3.32 (q, 4H, J = 7.1 Hz), 1.68 (s, 6H), 1.14 (t, 6H, J = 7.1 Hz). **^{13}C NMR ($CDCl_3$, 100 MHz):** δ 147.1, 144.78, 144.75, 134.5, 111.6, 111.4, 106.1, 52.7, 44.5, 41.1, 24.2, 12.4. **IR (CH_2Cl_2, cm^{-1})** ν_{max}: 3403, 2969, 2872, 1594, 1482, 1413, 1355, 1266, 1191, 1076, 795. **MS (EI 70 eV, *m/z*, %)** 326 (M^+, 1), 247 (99), 206 (100), 190 (46), 57 (64), 43 (37); **HRMS (EI^+)** *m/z*: $[M]^+$ Calcd for $C_{15}H_{23}N_2OBr$ 326.0994; Found 326.0992.

(*Z*)-2-(2-Bromo-6-fluorophenyl)-*N*,2-dimethylpropan-1-imine oxide (1h)

Following **GP4** from aldehyde **S3h**. The crude was purified by column chromatography to afford the title compound as a colorless solid in 65% yield (356 mg, 1.3 mmol).

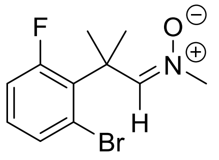

mp 82-84 °C. ***Rf:*** 0.38 (EtOAc/MeOH 20:1). **^{1}H NMR ($CDCl_3$, 400 MHz):** δ 7.30 (m, 1H), 6.97 (m, 2H), 6.90 (s, 1H), 3.60 (s, 3H), 1.74 (d, 6H, $^{5}J_{HF}$ = 4.5 Hz). **^{13}C NMR ($CDCl_3$, 100 MHz):** δ 161.9 (d, J_{CF} = 251.1), 145.2, 131.5 (d, J_{CF} = 12.5), 130.3 (d, J_{CF} = 2.9 Hz), 128.0 (d, J_{CF} = 10.2 Hz), 121.6 (d, J_{CF} = 5.9 Hz), 116.6 (d, J_{CF} = 27.1 Hz), 52.2, 41.5 (d, J_{CF} = 3.7 Hz), 25.3 (d, J_{CF} = 8.7 Hz). **^{19}F NMR ($CDCl_3$, 376 MHz):** δ -105.61 (m). **IR (CH_2Cl_2, cm^{-1})** ν_{max}: 3399, 3081, 2984, 2945, 2880, 1601, 1442, 1236, 1187, 859, 781. **MS (EI 70 eV, *m/z*, %)** 274 (M^+, 3), 194 (90), 134 (46), 125 (45), 84 (51), 43 (100); **HRMS (EI^+)** *m/z*: $[M]^+$ Calcd for $C_{11}H_{14}NOFBr$ 274.0243; Found 274.0253.

(*Z*)-2-(6-Bromobenzo[*d*][1,3]dioxol-5-yl)-*N*,2-dimethylpropan-1-imine oxide (1i)

Following **GP4** from aldehyde **S3i**. The crude was purified by column chromatography to afford the title compound as a white solid in 92% yield (552 mg, 1.84 mmol).

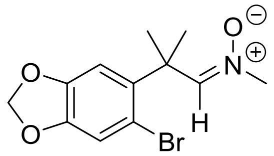

mp 76-78 °C. ***Rf:*** 0.36 (EtOAc/MeOH 10:1). **^{1}H NMR ($CDCl_3$, 400 MHz):** δ 6.95 (s, 1H), 6.93 (s, 1H), 6.89 (s, 1H), 5.91 (s, 2H), 3.59 (s, 3H), 1.60 (s, 6H). **^{13}C NMR ($CDCl_3$, 100 MHz):** δ 147.5, 146.5, 144.5, 138.2, 113.9, 110.9, 108.0, 101.7, 52.5, 40.7, 24.7. **IR (CH_2Cl_2, cm^{-1})** ν_{max}: 3387, 2970, 2906, 1601, 1504, 1480, 1237, 1135, 1037, 928, 836. **HRMS (ESI$^+$)** *m/z*: [M + H]$^+$ Calcd for $C_{12}H_{15}NO_3Br$ 300.0235; Found 300.0219.

(*Z*)-2-(1-Bromonaphthalen-2-yl)-*N*,2-dimethylpropan-1-imine oxide (1j)

Following **GP4** from 2-(1-bromonaphthalen-2-yl)-2-methylpropanal.[1] The crude was purified by column chromatography to afford the title compound as a pale-yellow solid in quantitative yield (612 mg, 2 mmol).

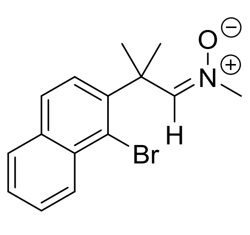

mp 134-136 °C. ***Rf:*** 0.28 (EtOAc/MeOH 10:1). **^{1}H NMR ($CDCl_3$, 400 MHz):** δ 8.36 (d, 1H, J = 8.6 Hz), 7.82 (d, 1H, J = 8.7 Hz), 7.77 (d, 1H, J = 8.1 Hz), 7.63 (d, 1H, J = 8.8 Hz), 7.53 (t, 1H, J = 7.3 Hz), 7.44 (t, 1H, J = 7.4 Hz), 7.05 (s, 1H), 3.58 (s, 3H), 1.78 (s, 6H). **^{13}C NMR ($CDCl_3$, 100 MHz):** δ 144.9, 142.7, 133.2, 132.6, 128.0, 127.7, 127.4, 127.1, 125.9, 125.1, 120.7, 52.2, 41.7, 24.8. **IR (CH_2Cl_2, cm^{-1})** ν_{max}: 3396, 3058, 2969, 2945, 2873, 1596, 1408, 1185, 957, 812, 749. **HRMS (ESI$^+$)** *m/z*: [M + H]$^+$ Calcd for $C_{15}H_{17}NOBr$ 306.0494; Found 306.0486.

(*Z*)-1-(1-(2-Bromophenyl)cyclopentyl)-*N*-methylmethanimine oxide (1k)

Following **GP4** from 1-(2-bromophenyl)cyclopentane-1-carbaldehyde.[10] The crude was purified by column chromatography to afford the title compound as a pale-yellow solid in quantitative yield (564 mg, 2 mmol).

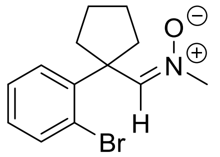

mp 79-81 °C. ***Rf:*** 0.31 (EtOAc/MeOH 10:1). **^{1}H NMR ($CDCl_3$, 400 MHz):** δ 7.49 (m, 2H), 7.27 (m, 1H), 7.04 (m, 1H), 7.01 (d, 1H, J = 0.6 Hz), 3.60 (d, 3H, J = 0.6 Hz), 2.55 (m, 2H), 2.20 (m, 2H), 1.74 (m, 4H). **^{13}C NMR ($CDCl_3$, 100 MHz):** δ 144.5, 143.6, 134.0, 129.1, 127.8, 126.8, 121.8, 52.8, 52.6, 35.0, 23.9. **IR (CH_2Cl_2, cm^{-1})** ν_{max}: 3397, 3060, 2952, 2873, 1600, 1465, 1409, 1173, 1019, 757, 542. **HRMS (ESI$^+$)** *m/z*: [M + H]$^+$ Calcd for $C_{13}H_{17}NOBr$ 282.0494; Found 282.0491.

(*Z*)-1-(1-(2-Bromophenyl)cyclopent-3-en-1-yl)-*N*-methylmethanimine oxide (1l)

Following **GP4** from 1-(2-bromophenyl)cyclopent-3-ene-1-carbaldehyde.[11] The crude was purified by column chromatography to afford the title compound as a yellow oil in 90% yield (504 mg, 1.8 mmol).

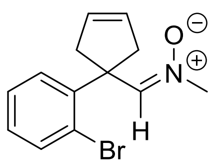

Rf: 0.30 (EtOAc/MeOH 10:1). **^{1}H NMR ($CDCl_3$, 400 MHz):** δ 7.51 (dd, 1H, J = 7.9 Hz, 1.2 Hz), 7.44 (dd, 1H, J = 8.0 Hz, 1.5 Hz), 7.26 (m, 1H), 7.11 (s, 1H), 7.05 (td, 1H, J = 7.7 Hz, 1.4 Hz), 5.74 (s, 2H), 3.61 (s, 3H), 3.20 (d, 2H, J = 15.1 Hz), 3.02 (d, 2H, J = 15.1 Hz). **^{13}C NMR ($CDCl_3$, 100 MHz):** δ 145.2, 143.2, 134.1, 129.3, 128.9, 127.9, 126.9, 121.6, 52.4, 52.2, 41.8. **IR (CH_2Cl_2, cm^{-1})** ν_{max}: 3396, 3056, 2945, 2855, 1599, 1468, 1408, 1183, 1064, 1023, 756. **MS (EI 70 eV, *m/z*, %)** 279 (M$^+$, 7), 200 (90), 159 (69), 154 (60), 43 (100); **HRMS (EI$^+$)** *m/z*: [M]$^+$ Calcd for $C_{13}H_{14}NOBr$ 279.0259; Found 279.0247.

(*Z*)-1-(4-(2-Bromophenyl)tetrahydro-2*H*-pyran-4-yl)-*N*-methylmethanimine oxide (1m)

Following **GP4** from aldehyde **S3m**. The crude was purified by column chromatography to afford the title compound as a white solid in 87% yield (519 mg, 1.74 mmol).

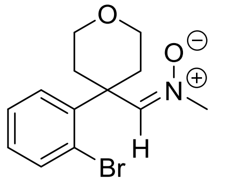

mp 137-139 °C. ***Rf:*** 0.33 (EtOAc/MeOH 5:1). **^{1}H NMR ($CDCl_3$, 400 MHz):** δ 7.49 (m, 2H), 7.33 (t, 1H, J = 7.5 Hz), 7.24 (s, 1H), 7.07 (t, 1H, J = 7.5 Hz), 3.94 (m, 2H), 3.67 (s, 3H), 3.65 (m, 2H), 2.86 (m, 2H), 2.02 (m, 2H). **^{13}C NMR ($CDCl_3$, 100 MHz):** δ 143.1, 140.9, 134.5, 128.9, 128.1, 127.5, 120.4, 65.0, 53.0, 42.9, 32.5. **IR (CH_2Cl_2, cm^{-1})** ν_{max}: 3401, 3052, 2956, 2852, 1598, 1467, 1413, 1183, 1103, 1015, 748. **MS (EI 70 eV, *m/z*, %)** 297 (M^+, 3), 218 (100), 147 (44), 128 (47), 115 (50), 96 (66); **HRMS (EI$^+$)** *m/z*: $[M]^+$ Calcd for $C_{13}H_{16}NO_2Br$ 297.0364; Found 297.0372.

(*Z*)-2-(2-Bromophenyl)-*N*,2-dimethyl-3-phenylpropan-1-imine oxide (1n)

Following **GP4** using aldehyde **S3n**. The crude was purified by column chromatography to afford the title compound as a white solid in 61% yield (405 mg, 1.22 mmol).

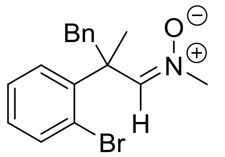

mp 139-141 °C. ***Rf:*** 0.33 (EtOAc/MeOH 20:1). **^{1}H NMR ($CDCl_3$, 400 MHz):** δ 7.57 (d, 1H, J = 7.7 Hz), 7.22 (m, 5H), 7.08 (m, 1H), 6.95 (m, 2H), 6.88 (s, 1H), 3.62 (s, 3H), 3.55 (d, 1H, J = 12.6 Hz), 3.33 (d, 1H, J = 12.6 Hz), 1.66 (s, 3H). **^{13}C NMR ($CDCl_3$, 100 MHz):** δ 143.3, 143.1, 136.8, 134.2, 130.6, 128.9, 127.9, 127.8, 127.3, 126.5, 120.9, 52.6, 44.9, 42.3, 20.3. **IR (CH_2Cl_2, cm^{-1})** ν_{max}: 3395, 3060, 3028, 2943, 2878, 1602, 1467, 1410, 1179, 1016, 704. **MS (EI 70 eV, *m/z*, %)** 331 (M^+, 3), 252 (100), 161 (99), 91 (99), 43 (85); **HRMS (EI$^+$)** *m/z*: $[M]^+$ Calcd for $C_{17}H_{18}NOBr$ 331.0572; Found 331.0570.

(*Z*)-2-(2-Bromophenyl)-*N*-methylhexan-1-imine oxide (1o)

Following **GP4** from aldehyde **S3o**. The crude was purified by column chromatography to afford the title compound as a colorless oil in 80% yield (455 mg, 1.6 mmol).

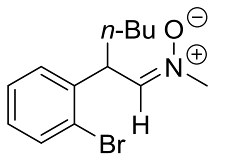

Rf: 0.30 (EtOAc/MeOH 20:1). **^{1}H NMR ($CDCl_3$, 400 MHz):** δ 7.52 (dd, 1H, J = 8.0 Hz, 1.1 Hz), 7.24 (m, 1H), 7.20 (dd, 1H, J = 7.7 Hz, 2.0 Hz), 7.06 (m, 1H), 6.85 (d, 1H, J = 7.0 Hz), 4.54 (m, 1H), 3.65 (s, 3H), 1.92 (m, 1H), 1.78 (m, 1H), 1.28 (m, 4H), 0.85 (t, 3H, J = 7.1 Hz). **^{13}C NMR ($CDCl_3$, 100 MHz):** δ 140.9, 140.2, 133.3, 129.1, 128.2, 127.5, 124.5, 65.2, 52.5, 42.7, 31.8, 29.2, 22.5, 13.8. **IR (CH_2Cl_2, cm^{-1})** ν_{max}: 3401, 3057, 2955, 2859, 1599, 1469, 1437, 1409, 1177, 1024, 756. **HRMS (ESI$^+$)** *m/z*: $[M + H]^+$ Calcd for $C_{13}H_{19}NOBr$ 284.0650; Found 284.0652.

(*Z*)-2-(2-Bromophenyl)-*N*-methyl-2-(4-nitrophenyl)hexan-1-imine oxide (1p)

Following **GP4** from aldehyde **S3p**. The crude was purified by column chromatography to afford the title compound as a pale-yellow solid in 35% yield (284 mg, 0.7 mmol).

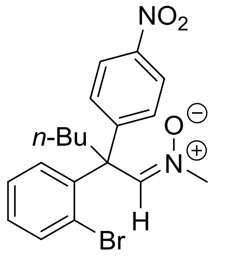

mp 159-161 °C. ***Rf:*** 0.33 (EtOAc). **^{1}H NMR ($CDCl_3$, 400 MHz):** δ 8.13 (d, 2H, J = 9.0 Hz), 7.71 (dd, 1H, J = 8.0 Hz, 1.5 Hz), 7.56 (s, 1H), 7.48 (dd, 1H, J = 7.9 Hz, 1.4 Hz), 7.42 (m, 1H), 7.27 (d, 2H, J = 9.0 Hz), 7.17 (td, 1H, J = 7.6 Hz, 1.6 Hz), 3.74 (s, 3H), 3.20 (m, 1H), 2.19 (m, 1H), 1.39 - 1.17 (m, 2H), 1.01 (m, 2H), 0.82 (t, 3H, J = 7.3 Hz). **^{13}C NMR ($CDCl_3$, 100 MHz):** δ 150.8, 146.4, 141.7, 139.8, 134.8, 130.9, 129.0, 128.0, 127.0, 123.2, 122.8, 53.84, 53.76, 35.6, 27.5, 23.0, 13.9. **IR (CH_2Cl_2, cm^{-1})** ν_{max}: 3061, 2957, 2930, 2871, 1595, 1517, 1423, 1347, 1206, 856, 752. **HRMS (ESI$^+$)** *m/z*: $[M + Na]^+$ Calcd for $C_{19}H_{21}N_2O_3NaBr$ 427.0633; Found 427.0634.

(*Z*)-3-(2-Bromophenyl)-*N*,2,2-trimethylpropan-1-imine oxide (1q)

Following **GP4** from 3-(2-bromophenyl)-2,2-dimethylpropanal.[1] The crude was purified by column chromatography to afford the title compound as a white solid in 78% yield (422 mg, 1.56 mmol).

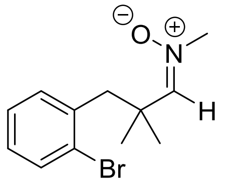

mp 82-84 °C. ***Rf:*** 0.28 (EtOAc/MeOH 10:1). **^{1}H NMR (CDCl$_3$, 400 MHz):** δ 7.50 (dd, 1H, $^3J_{HH}$ = 8.0 Hz, ^4J = 1.2 Hz), 7.18 (m, 1H), 7.12 (dd, 1H, $^3J_{HH}$ = 7.7 Hz, ^4J = 1.9 Hz), 7.01 (m, 1H), 6.33 (q, 1H, $^4J_{HH}$ = 0.7 Hz), 3.65 (d, 3H, $^4J_{HH}$ = 0.7 Hz), 3.30 (s, 2H), 1.27 (s, 6H). **^{13}C NMR (CDCl$_3$, 100 MHz):** δ 144.6, 138.6, 132.8, 131.5, 127.7, 127.1, 125.8, 53.9, 40.8, 38.5, 24.7. **IR (CH$_2$Cl$_2$, cm^{-1})** ν_{max}: 3408, 3062, 2962, 2870, 2053, 1598, 1469, 1413, 1156, 1023, 755. **MS (ESI$^+$)** *m/z*: [M + Na]$^+$ Calcd for $C_{12}H_{16}NONaBr$ 292.0313; Found 292.0315.

(*Z*)-*N*-Benzyl-2-(2-bromophenyl)-2-methylpropan-1-imine oxide (1r)

Following **GP4** from 2-(2-bromophenyl)-2-methylpropanal and *N*-benzylhydroxylamine hydrochloride as substrates. The crude was purified by column chromatography to afford the title compound as a white crystals in 84% yield (558 mg, 1.68 mmol).

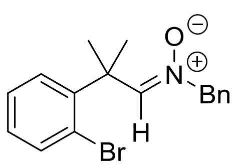

mp 124-126 °C (recrystallized from hexane/EtOAc). ***Rf:*** 0.30 (Hexane/EtOAc 1:1). **^{1}H NMR (CDCl$_3$, 400 MHz):** δ 7.50 (dd, 1H, J = 7.9 Hz, 1.0 Hz), 7.45 (dd, 1H, J = 8.0 Hz, 1.3 Hz), 7.41 (m, 2H), 7.37 (m, 3H), 7.29 (m, 1H), 7.05 (m, 1H), 6.91 (s, 1H), 4.83 (s, 2H), 1.65 (s, 6H). **^{13}C NMR (CDCl$_3$, 100 MHz):** δ 144.6, 143.3, 134.1, 132.9, 129.5, 128.7, 128.7, 127.8, 127.8, 127.5, 121.0, 69.5, 41.0, 24.4. **IR (CH$_2$Cl$_2$, cm^{-1})** ν_{max}: 3384, 3063, 2969, 1588, 1467, 1426, 1356, 1173, 1020, 754, 703. **HRMS (ESI$^+$)** *m/z*: [M + Na]$^+$ Calcd for $C_{17}H_{18}NONaBr$ 354.0469; Found 354.0464.

Synthesis of nitrones 1s, 1t, 1u

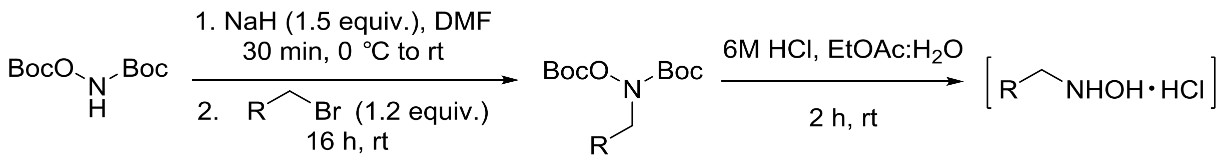

General procedure for the alkylation of *N*,*O*-di-Boc-protected hydroxylamines and the following deprotection (GP5):

Under argon atmosphere, a round-bottom flask was equipped with a stirring bar and loaded with DMF (40 mL) and *N*,*O*-di-Boc-hydroxylamine (2.333 g, 10 mmol, 1 equiv.). The solution was cooled down to 0 °C and sodium hydride (60% in oil, 600 mg, 15 mmol, 1.5 equiv.) was added in portions. After completion of the addition, the ice bath was removed and the reaction mixture was stirred for 30 min at room temperature. Then, the appropriate alkyl bromide (12 mmol, 1.2 equiv.) was added dropwise. The resulting mixture was stirred at room temperature for 16 h before quenching by the addition of saturated aqueous solution of NH_4Cl. The solution was transferred into a separatory funnel and extracted with EtOAc (3 x 20 mL). The combined organic phases were dried over anhydrous Na_2SO_4 and the solvent was removed under reduced pressure. The crude material was purified by column chromatography (silica gel, hexanes/EtOAc).

***Tert*-butyl ((*tert*-butoxycarbonyl)oxy)(phenethyl)carbamate (S4)**

Following **GP5** from (2-bromoethyl)benzene. The crude was purified by column chromatography to afford the title compound as a white solid in 82% yield (2.77 g, 8.20 mmol).

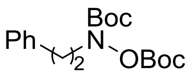

mp 66-68 °C. ***Rf:*** 0.27 (Hexane/EtOAc 20:1). **^{1}H NMR ($CDCl_3$, 400 MHz):** δ 7.29 (m, 2H), 7.21 (m, 3H), 3.81 (m, 2H), 2.92 (t, 2H, J = 7.6 Hz), 1.53 (s, 9H), 1.43 (s, 9H). **^{13}C NMR ($CDCl_3$, 100 MHz):** δ 154.5, 152.2, 138.4, 128.8, 128.5, 126.4, 84.7, 82.2, 51.6, 33.5, 28.0, 27.6. **IR (CH_2Cl_2, cm^{-1})** ν_{max}: 2980, 2934, 1786, 1720, 1369, 1250, 1146, 1099, 1074, 747, 700. **HRMS (ESI$^+$)** *m/z*: $[M + Na]^+$ Calcd for $C_{18}H_{27}NO_5Na$ 360.1787; Found 360.1788.

Methyl 4-((*tert*-butoxycarbonyl)((*tert*-butoxycarbonyl)oxy)amino)butanoate (S5)

Following **GP5** from methyl 4-bromobutanoate. The crude was purified by column chromatography to afford the title compound as a colorless oil in 72% yield (2.40 g, 7.20 mmol).

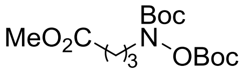

Rf: 0.36 (Hexane/EtOAc 5:1). **^{1}H NMR ($CDCl_3$, 400 MHz):** δ 3.58 (s, 3H), 3.56 (m, 2H), 2.33 (t, 2H, J = 7.4 Hz), 1.82 (pent, 2H, J = 7.2 Hz), 1.44 (s, 9H), 1.38 (s, 9H). **^{13}C NMR ($CDCl_3$, 100 MHz):** δ 173.1, 154.6, 152.1, 84.6, 82.1, 51.3, 49.1, 30.7, 27.9, 27.4, 22.3. **IR (CH_2Cl_2, cm^{-1})** ν_{max}: 2981, 2938, 1785, 1739, 1438, 1370, 1255, 1147, 1106, 836, 781. **HRMS (ESI$^+$)** *m/z*: $[M + Na]^+$ Calcd for $C_{15}H_{27}NO_7Na$ 356.1685; Found 356.1670.

***Tert*-butyl but-3-en-1-yl((*tert*-butoxycarbonyl)oxy)carbamate (S6)**

Following **GP5** from 4-bromo-1-butene. The crude was purified by column chromatography to afford the title compound as a colorless oil in 79% yield (2.27 g, 7.90 mmol).

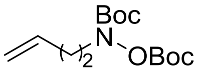

Rf: 0.41 (Hexane/EtOAc 20:1). **^{1}H NMR ($CDCl_3$, 400 MHz):** δ 5.75 (ddt, 1H, J = 17.1 Hz, 10.3 Hz, 6.8 Hz), 5.06 (dm, 1H, J = 17.2 Hz), 5.00 (dm, 1H, 10.2 Hz), 3.60 (t, 2H, J = 6.7 Hz), 2.32 (m, 2H), 1.48 (s, 9H), 1.43 (s, 9H). **^{13}C NMR ($CDCl_3$, 100 MHz):** δ 154.7, 152.2, 134.7, 116.8, 84.6, 82.1, 49.6, 31.4, 28.0, 27.5. **IR (CH_2Cl_2, cm^{-1})** ν_{max}: 3080, 2980, 2935, 1786, 1719, 1457, 1369, 1253, 1137, 916, 756. **HRMS (ESI$^+$)** *m/z*: $[M + Na]^+$ Calcd for $C_{14}H_{25}NO_5Na$ 310.1630; Found 310.1632.

Deprotection of hydroxylamines S4, S5, S6: Hydroxylamine derivative (5 mmol, 1 equiv.) was dissolved in EtOAc (20 mL) following by the addition of 6M HCl solution (20 mL). The reaction mixture was stirred at room temperature for 2 h and concentrated under reduced pressure giving the desired hydroxylamine hydrochloride in quantitative yield. Deprotected hydroxylamines were used directly for the synthesis of nitrones **1s, 1t, 1u** without further purification.

(*Z*)-2-(2-Bromophenyl)-2-methyl-*N*-phenethylpropan-1-imine oxide (1s)

Following **GP4** from 2-(2-bromophenyl)-2-methylpropanal and *N*-phenethylhydroxylamine hydrochloride as substrates. The crude purified by column chromatography to afford the title compound as a colorless oil in 82% yield (568 mg, 1.64 mmol).

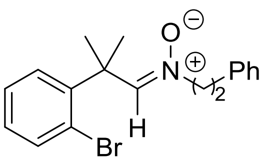

Rf: 0.40 (EtOAc). **^{1}H NMR ($CDCl_3$, 400 MHz):** δ 7.50 (dd, 1H, ^3J = 7.9 Hz, ^4J = 1.4 Hz), 7.45 (dd, 1H, ^3J = 8.0 Hz, ^4J = 1.6 Hz), 7.30 (m, 3H), 7.23 (m, 3H), 7.05 (m, 1H), 6.76 (s, 1H), 3.92 (t, 2H, J = 7.1 Hz), 3.18 (t, 2H, J = 7.1 Hz), 1.59 (s, 6H). **^{13}C NMR ($CDCl_3$, 100 MHz):** δ 144.6, 143.9, 137.8, 134.1, 128.9, 128.5, 127.7, 127.7, 127.5, 126.6, 121.0, 66.4, 40.9, 33.0, 24.3. **IR (CH_2Cl_2, cm^{-1})** ν_{max}: 3399, 3061, 2969, 2871, 1722, 1592, 1468, 1176, 1020, 754, 701. **HRMS (ESI$^+$)** *m/z*: $[M + Na]^+$ Calcd for $C_{18}H_{20}NONaBr$ 368.0626; Found 368.0631.

(*Z*)-2-(2-Bromophenyl)-*N*-(4-methoxy-4-oxobutyl)-2-methylpropan-1-imine oxide (1t)

Following **GP4** from 2-(2-bromophenyl)-2-methylpropanal and methyl 4-(hydroxyamino)butanoate hydrochloride as substrates. The crude was purified by column chromatography to afford the title compound as a colorless oil in 68% yield (465 mg, 1.36 mmol).

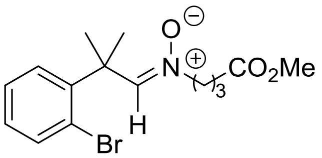

Rf: 0.20 (EtOAc). **^{1}H NMR ($CDCl_3$, 400 MHz):** δ 7.50 (dd, 1H, ^{3}J = 7.9 Hz, ^{4}J = 1.2 Hz), 7.46 (dd, 1H, ^{3}J = 7.9 Hz, ^{4}J = 1.5 Hz), 7.29 (m, 1H), 7.05 (m, 1H), 6.96 (s, 1H), 3.80 (t, 2H, J = 6.7 Hz), 3.66 (s, 3H), 2.39 (t, 2H, J = 7.0 Hz), 2.16 (m, 2H), 1.67 (s, 6H). **^{13}C NMR ($CDCl_3$, 100 MHz):** δ 173.2, 144.5, 144.1, 134.2, 127.8, 127.7, 127.6, 120.9, 63.6, 51.6, 41.0, 30.4, 24.4, 22.0. **IR (CH_2Cl_2, cm^{-1})** ν_{max}: 3442, 3065, 2969, 2874, 1736, 1591, 1436, 1173, 1020, 758, 723. **HRMS (ESI^{+})** *m/z*: [M + Na]$^{+}$ Calcd for $C_{15}H_{20}NO_3NaBr$ 364.0524; Found 364.0508.

(*Z*)-2-(2-Bromophenyl)-*N*-(but-3-en-1-yl)-2-methylpropan-1-imine oxide (1u)

Following **GP4** from 2-(2-bromophenyl)-2-methylpropanal and *N*-(but-3-en-1-yl)hydroxylamine hydrochloride as substrates. The crude was purified by column chromatography to afford the title compound as a white solid in 82% yield (486 mg, 1.64 mmol).

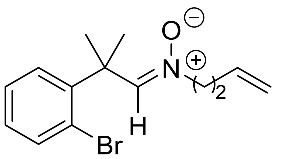

mp 46-48 °C. ***Rf:*** 0.37 (EtOAc). **^{1}H NMR ($CDCl_3$, 400 MHz):** δ 7.50 (d, 1H, J = 7.9 Hz), 7.46 (d, 1H, J = 7.9 Hz), 7.30 (m, 1H), 7.05 (m, 1H), 6.93 (s, 1H), 5.76 (m, 1H), 5.12 (dm, 1H, J = 17.1 Hz), 5.07 (d, 1H, J = 10.2 Hz), 3.74 (t, 2H, J = 7.1 Hz), 2.61 (m, 2H), 1.67 (s, 6H). **^{13}C NMR ($CDCl_3$, 100 MHz):** δ 144.6, 143.6, 134.1, 133.8, 127.8, 127.8, 127.5, 120.9, 117.6, 64.4, 41.0, 31.1, 24.5. **IR (CH_2Cl_2, cm^{-1})** ν_{max}: 3391, 3074, 2972, 1723, 1591, 1468, 1424, 1178, 1019, 917, 756. **HRMS (ESI^{+})** *m/z*: [M + Na]$^{+}$ Calcd for $C_{14}H_{18}NONaBr$ 318.0469; Found 318.0455.

(*Z*)-2-(2-bromo-3-(but-3-en-1-yl)phenyl)-*N*,2-dimethylpropan-1-imine oxide (1v)

Following **GP4** from aldehyde **S3v**. The crude was purified by column chromatography to afford the title compound as a colorless oil in quantitative yield (621 mg, 2 mmol).

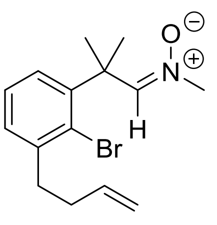

Rf: 0.31 (EtOAc/MeOH 10:1). **^{1}H NMR ($CDCl_3$, 400 MHz):** δ 7.30 (dd, 1H, J = 7.9 Hz, 1.6 Hz), 7.22 (t, 1H, J = 7.7 Hz), 7.09 (dd, 1H, J = 7.4 Hz, 1.6 Hz), 6.95 (s, 1H), 5.88 (ddt, 1H, J = 17.0 Hz, 10.3 Hz, 6.6 Hz), 5.06 (dm, 1H, J = 17.1 Hz), 4.98 (dm, 1H, J = 10.2 Hz), 3.59 (s, 3H), 2.84 (m, 2H), 2.35 (m, 2H), 1.69 (s, 6H). **^{13}C NMR ($CDCl_3$, 100 MHz):** δ 145.3, 144.7, 142.0, 137.8, 128.4, 127.3, 125.6, 123.4, 114.8, 52.5, 41.6, 36.9, 33.7, 24.7. **IR (CH_2Cl_2, cm^{-1})** ν_{max}: 3394, 3075, 2970, 2871, 1602, 1456, 1409, 1194, 1015, 913, 726. **HRMS (ESI^{+})** *m/z*: [M + H]$^{+}$ Calcd for $C_{15}H_{21}NOBr$ 310.0807; Found 310.0810.

3.2 Palladium catalyzed nitrone C–H cyclization

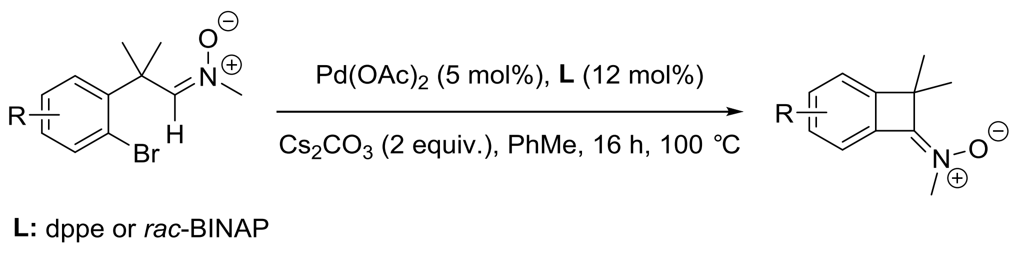

L: dppe or *rac*-BINAP

General procedure for the palladium-catalyzed C–H cyclization of aldonitrones (GP6):

Under argon atmosphere, a flame-dried Schlenk flask was loaded with anhydrous PhMe (2 mL), Cs_2CO_3 (326 mg, 1 mmol, 2 equiv.), dppe or *rac*-BINAP (23.9 mg or 37.4 mg respectively, 0.06 mmol, 0.12 equiv.), $Pd(OAc)_2$ (5.6 mg, 0.025 mmol, 0.05 equiv.) and aldonitrone (0.5 mmol, 1 equiv.). The mixture was stirred at 100 °C for 16 h (oil bath). After the reaction was complete, the reaction mixture was cooled down to room temperature, diluted with DCM (ca. 5 mL), filtered through a pad of Celite and concentrated under reduced pressure. The resulting crude material was purified by column chromatography (silica gel, hexanes/EtOAc) to afford cyclobutenitrone as a mixture inseparable *E* and *Z* isomers.

Nearly all cyclic ketonitrones **2** were obtained as mixtures of inseparable *E/Z* isomers (with major *E* isomer as determined by NOE experiments), even though the starting materials exist exclusively as *E* isomers. Isomerization of BCBn **2** takes place spontaneously even at room temperature and their *E/Z* isomeric composition may vary with time.

(*E*)-*N*,8,8-Trimethylbicyclo[4.2.0]octa-1,3,5-trien-7-imine oxide (2a)

Following **GP6** from aldonitrone **1a** with dppe as ligand. The crude was purified by column chromatography to afford the title compound as a yellow oil in 94% yield (82 mg, 0.47 mmol, *E:Z* 2.3:1, based on NOE NMR spectra).

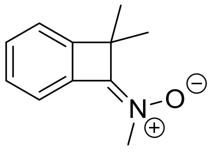

Rf: 0.29 (EtOAc/MeOH 5:1). *Major isomer:* **^{1}H NMR (CDCl$_3$, 500 MHz):** δ 7.25 (m, 2H), 7.19 (m, 1H), 7.09 (m, 1H), 3.77 (s, 3H), 1.55 (s, 6H). **^{13}C NMR (CDCl$_3$, 125 MHz):** δ 153.4, 150.5, 134.6, 130.7, 128.7, 120.5, 119.2, 54.3, 49.0, 22.7. *Minor isomer:* **^{1}H NMR (CDCl$_3$, 500 MHz):** δ 7.59 (d, 1H, J = 6.8 Hz), 7.29 (m, 2H), 7.14 (m, 1H), 3.60 (s, 3H), 1.52 (s, 6H). **^{13}C NMR (CDCl$_3$, 125 MHz):** δ 152.9, 150.1, 135.8, 131.9, 129.0, 122.4, 119.3, 55.1, 46.5, 23.9.**IR (CH_2Cl_2, cm^{-1})** ν_{max}: 3407, 3064, 2964, 2926, 1658, 1460, 1345, 1231, 1171, 1019, 755. **MS (EI 70 eV, *m/z*, %)** 175 (M$^+$, 66), 158 (100), 115 (58), 84 (63), 49 (76); **HRMS (EI$^+$)** *m/z*: [M]$^+$ Calcd for $C_{11}H_{13}NO$ 175.0997; Found 175.0999.

(*E*)-4-Fluoro-*N*,8,8-trimethylbicyclo[4.2.0]octa-1,3,5-trien-7-imine oxide (2b)

Following **GP6** from aldonitrone **1b** and dppe as ligand. The crude was purified by column chromatography to afford the title compound as a pale-yellow oil in 78% yield (75 mg, 0.39 mmol, *E:Z* 1.1:1, based on NOE NMR spectra).

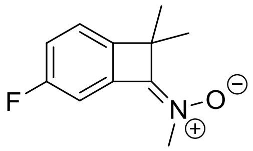

Rf: 0.29 (EtOAc/MeOH 10:1). *Major isomer:* **^{1}H NMR (CDCl$_3$, 400 MHz):** δ 7.19 (ddd, 1H, $^3J_{HH}$ = 8.0 Hz, $^4J_{HF}$ = 4.4 Hz, $^5J_{HH}$ = 0.7 Hz), 6.99 (m, 1H), 6.86 (ddd, 1H, $^3J_{HF}$ = 7.5 Hz, $^4J_{HH}$ = 2.0 Hz, $^5J_{HH}$ = 0.6 Hz), 3.80 (s, 3H), 1.56 (s, 6H). **^{19}F NMR (CDCl$_3$, 376 MHz):** δ -110.55 (m). *Minor isomer:* **^{1}H NMR (CDCl$_3$, 400 MHz):** δ 7.34 (ddd, 1H, $^3J_{HF}$ = 7.2 Hz, $^4J_{HH}$ = 2.1 Hz, $^5J_{HH}$ = 0.5 Hz), 7.14 (ddd, 1H, $^3J_{HH}$ = 8.7 Hz, $^4J_{HF}$ = 4.4 Hz, $^5J_{HH}$ = 0.6 Hz), 7.03 (m, 1H), 3.66 (s, 3H), 1.56 (s, 6H). **^{19}F NMR (CDCl$_3$, 376 MHz):** δ -110.10 (m). *Both isomers:* **^{13}C NMR (CDCl$_3$, 100 MHz):** δ 163.22 (d, $^1J_{CF}$ = 247.6 Hz), 163.19 (d, $^1J_{CF}$ = 246.6 Hz), 149.4 (d, $^4J_{CF}$ = 4.3 Hz), 149.0 (d, $^4J_{CF}$ = 4.3 Hz), 148.6 (d, $^4J_{CF}$ = 2.7 Hz), 148.1 (d, $^4J_{CF}$ = 2.5 Hz), 136.8 (d, $^3J_{CF}$ = 9.8 Hz), 135.3 (d, $^3J_{CF}$ = 9.3 Hz), 122.4 (d, $^3J_{CF}$ = 9.3 Hz), 121.3 (d, $^3J_{CF}$ = 8.9 Hz), 119.4 (d, $^2J_{CF}$ = 24.4 Hz), 118.0 (d, $^2J_{CF}$ = 24.2 Hz), 109.7 (d, $^2J_{CF}$ = 24.2 Hz), 106.9 (d, $^2J_{CF}$ = 24.6 Hz), 54.4, 53.5, 49.1, 46.8, 24.0, 22.8. **IR (CH_2Cl_2, cm^{-1})** ν_{max}: 3414, 2965, 2866, 1767, 1657, 1587, 1468, 1329, 1238, 1020, 839. **HRMS (ESI$^+$)** *m/z*: [M + H]$^+$ Calcd for $C_{11}H_{13}NOF$ 194.0981; Found 194.0972.

(*E*)-4-(*Tert*-butyl)-*N*,8,8-trimethylbicyclo[4.2.0]octa-1,3,5-trien-7-imine oxide (2c)

Following **GP6** from aldonitrone **1c** and dppe as a ligand. The crude was purified by column chromatography to afford the title compound as a orange oil in quantitative yield (115 mg, 0.5 mmol, *E:Z* 1:1.2, based on NOE NMR spectra).

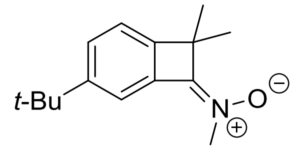

Rf: 0.24 (EtOAc/MeOH 10:1). *Major isomer:* **^{1}H NMR ($CDCl_3$, 400 MHz):** δ 7.65 (m, 2H), 7.49 - 7.38 (m, 1H), 7.11 (dd, 1H, J = 7. Hz, 0.9 Hz), 3.62 (s, 3H), 1.53 (s, 6H), 1.27 (s, 9H). **^{13}C NMR ($CDCl_3$, 100 MHz):** δ 152.8, 151.1, 150.3, 135.6, 129.8, 119.8, 119.0, 54.7, 46.3, 35.3, 31.4, 24.1. *Minor isomer:* **^{1}H NMR ($CDCl_3$, 400 MHz):** δ 7.49 - 7.38 (m, 1H), 7.36 (dd, 1H, J = 7.8 Hz, 1.4 Hz), 7.15 (m, 2H), 3.82 (s, 3H), 1.56 (s, 6H), 1.28 (s, 9H). **^{13}C NMR ($CDCl_3$, 100 MHz):** δ 152.4, 151.1, 150.9, 134.4, 128.8, 120.3, 116.2, 53.9, 49.0, 35.2, 31.4, 23.0. **IR (CH_2Cl_2, cm^{-1})** ν_{max}: 3410, 3055, 2961, 2868, 1759, 1653, 1478, 1364, 1231, 1016, 741. **MS (EI 70 eV, *m/z*, %)** 231 (M^+, 52), 214 (100), 187 (34), 159 (53), 58 (25); **HRMS (EI$^+$)** *m/z*: $[M]^+$ Calcd for $C_{15}H_{21}NO$ 231.1623; Found 231.1624.

(*E*)-4-(Methoxycarbonyl)-*N*,8,8-trimethylbicyclo[4.2.0]octa-1,3,5-trien-7-imine oxide (2d)

Following **GP6** from aldonitrone **1d** and dppe as a ligand. The crude was purified by column chromatography to afford the title compound as a yellow oil in quantitative yield (117 mg, 0.5 mmol, *E:Z* 1.1:1, based on NOE NMR spectra).

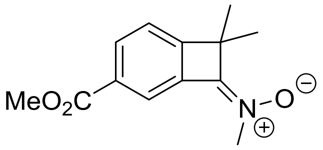

Rf: 0.47 (EtOAc/MeOH 5:1). *Major isomer:* **^{1}H NMR ($CDCl_3$, 400 MHz):** δ 8.05 (dd, 1H, J = 7.7 Hz, 1.2 Hz), 7.83 (m, 1H), 7.32 (dd, 1H, J = 7.7 Hz, 1.0 Hz), 3.91 (s, 3H), 3.88 (s, 3H), 1.61 (s, 6H). **^{13}C NMR ($CDCl_3$, 100 MHz):** δ 166.4, 158.0, 149.3, 134.9, 132.3, 130.9, 120.6, 120.2, 54.5, 52.4, 49.4, 22.4. *Minor isomer:* **^{1}H NMR ($CDCl_3$, 400 MHz):** δ 8.28 (m, 1H), 8.12 (dd, 1H, J = 7.7 Hz, 1.3 Hz), 7.28 (dd, 1H, J = 7.7 Hz, 1.0 Hz), 3.89 (s, 3H), 3.67 (s, 3H), 1.60 (s, 6H). **^{13}C NMR ($CDCl_3$, 100 MHz):** δ 166.5, 157.3, 148.9, 136.1, 133.6, 131.3, 123.4, 119.5, 55.3, 52.2, 46.9, 23.7. **IR (CH_2Cl_2, cm^{-1})** ν_{max}: 3419, 2959, 2867, 1721, 1573, 1435, 1300, 1236, 1096, 1021, 753. **MS (EI 70 eV, *m/z*, %)** 233 (M^+, 49), 216 (100), 189 (24), 145 (45), 128 (34), 115 (40); **HRMS (EI$^+$)** *m/z*: $[M]^+$ Calcd for $C_{13}H_{15}NO_3$ 233.1052; Found 233.1054.

(*E*)-3-Chloro-*N*,8,8-trimethylbicyclo[4.2.0]octa-1,3,5-trien-7-imine oxide (2e)

Following **GP6** from aldonitrone **1e** and *rac*-BINAP as a ligand. The crude was purified by column chromatography to afford the title compound as a orange oil in 54% yield (57 mg, 0.27 mmol, *E:Z* 1.4:1, based on NOE NMR spectra).

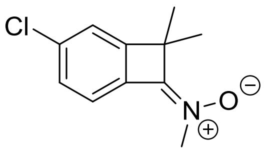

Rf: 0.27 (EtOAc/MeOH 5:1). *Major isomer:* **^{1}H NMR ($CDCl_3$, 400 MHz):** δ 7.27 (m, 1H), 7.20 (m, 1H), 7.05 (d, 1H, J = 8.0 Hz), 3.77 (s, 3H), 1.54 (s, 6H). **^{13}C NMR ($CDCl_3$, 100 MHz):** δ 154.3, 149.1, 136.3, 132.6, 129.3, 121.5, 120.5, 54.3, 48.9, 22.5. *Minor isomer:* **^{1}H NMR ($CDCl_3$, 400 MHz):** δ 7.53 (d, 1H, J = 7.9 Hz), 7.27 (m, 1H), 7.16 (m, 1H), 3.61 (s, 3H), 1.54 (s, 6H). **^{13}C NMR ($CDCl_3$, 100 MHz):** δ 153.8, 148.8, 137.3, 133.9, 129.8, 123.7, 120.4, 55.2, 46.6, 213.7. **IR (CH_2Cl_2, cm^{-1})** ν_{max}: 3408, 3058, 2965, 2866, 1654, 1436, 1297, 1238, 1092, 1019, 822. **MS (EI 70 eV, *m/z*, %)** 209 (M^+, 72), 192 (100), 180 (45), 165 (71), 128 (37), 115 (70); **HRMS (EI$^+$)** *m/z*: $[M]^+$ Calcd for $C_{11}H_{12}NOCl$ 209.0607; Found 209.0607.

(*E*)-*N*,8,8-Trimethyl-3-(trifluoromethyl)bicyclo[4.2.0]octa-1,3,5-trien-7-imine oxide (2f)

Following **GP6** from aldonitrone **1f** and *rac*-BINAP as a ligand. The crude was purified by column chromatography to afford the title compound as a brown solid in 72% yield (88 mg, 0.36 mmol, *E:Z* 1.2:1, based on NOE NMR spectra).

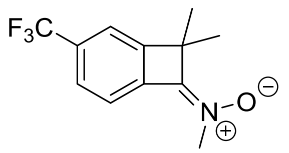

mp 58-60 °C. ***Rf:*** 0.23 (EtOAc/MeOH 10:1). *Major isomer:* **^{1}H NMR ($CDCl_3$, 400 MHz):** δ 7.23 (d, 1H, J = 7.9 Hz), 7.59 (m, 1H), 7.48 (s, 1H), 3.87 (s, 3H), 1.60 (s, 6H). **^{13}C NMR ($CDCl_3$, 100 MHz):** δ 153.5, 149.0, 137.6, 131.9 (q, $^2J_{CF}$ = 32.0 Hz), 126.2 (q, $^3J_{CF}$ = 4.2 Hz), 123.8 (q, $^1J_{CF}$ = 272.9 Hz), 119.2, 117.9 (q, $^3J_{CF}$ = 3.9 Hz), 54.4, 49.5, 22.5. **^{19}F NMR ($CDCl_3$, 376 MHz):** δ -62.39 (s). *Minor isomer:* **^{1}H NMR ($CDCl_3$, 400 MHz):** δ 7.72 (d, 1H, J = 7.8 Hz), 7.59 (m, 1H), 7.44 (s, 1H), 3.69 (s, 3H), 1.61 (s, 6H). **^{13}C NMR ($CDCl_3$, 125 MHz):** δ 152.9, 148.6, 139.1, 133.0 (q, $^2J_{CF}$ = 30.8 Hz), 126.5 (q, $^3J_{CF}$ = 4.2 Hz), 123.8 (q, $^1J_{CF}$ = 272.9 Hz), 122.4, 116.8 (q, $^3J_{CF}$ = 4.1 Hz), 55.4, 47.1, 23.8. **^{19}F NMR ($CDCl_3$, 376 MHz):** δ -62.40 (s). **IR (CH_2Cl_2, cm^{-1})** ν_{max}: 3419, 2969, 2928, 2867, 1755, 1657, 1581, 1295, 1166, 1123, 1044. **HRMS (ESI$^+$)** *m/z*: $[M + H]^+$ Calcd for $C_{12}H_{13}NOF_3$ 244.0949; Found 244.0943.

(*E*)-3-(Diethylamino)-*N*,8,8-trimethylbicyclo[4.2.0]octa-1,3,5-trien-7-imine oxide (2g)

Following **GP6** from aldonitrone **1g** and dppe as a ligand. The crude was purified by column chromatography to afford the title compound as a brown oil in 81% yield (100 mg, 0.41 mmol, *E:Z* 1.9:1, based on NOE NMR spectra).

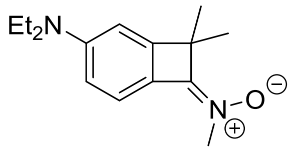

Rf: 0.32 (EtOAc/MeOH 2:1). *Major isomer:* **^{1}H NMR ($CDCl_3$, 400 MHz):** δ 6.95 (d, 1H, J = 8.5 Hz), 6.53 (m, 1H), 6.54 (s, 1H), 3.65 (s, 3H), 3.34 (m, 4H), 1.54 (s, 6H), 1.13 (t, 6H, J = 7.1 Hz). **^{13}C NMR ($CDCl_3$, 100 MHz):** δ 155.6, 151.3, 150.5, 121.1, 120.6, 112.1, 101.8, 53.7, 47.6, 44.6, 22.8, 12.3. *Minor isomer:* **^{1}H NMR ($CDCl_3$, 400 MHz):** δ 7.43 (d, 1H, J = 8.5 Hz), 6.53 (m, 1H), 6.37 (s, 1H), 3.53 (s, 3H), 3.34 (m, 4H), 1.49 (s, 6H), 1.13 (t, 6H, J = 7.1 Hz). **^{13}C NMR ($CDCl_3$, 100 MHz):** δ 155.3, 151.5, 150.7, 124.6, 121.7, 112.5, 100.2, 54.2, 45.2, 44.6, 23.7, 12.3. **IR (CH_2Cl_2, cm^{-1})** ν_{max}: 3382, 2970, 2899, 1731, 1591, 1502, 1352, 1191, 1052, 1011, 805. **MS (EI 70 eV, *m/z*, %)** 246 (M^+, 100), 231 (28), 229 (91), 202 (69), 199 (43), 185 (28); **HRMS (EI$^+$)** *m/z*: $[M]^+$ Calcd for $C_{15}H_{22}N_2O$ 246.1732; Found 246.1737.

(*E*)-2-Fluoro-*N*,8,8-trimethylbicyclo[4.2.0]octa-1,3,5-trien-7-imine oxide (2h)

Following **GP6** from aldonitrone **1h** with dppe as a ligand. The crude was purified by column chromatography to afford the title compound as a orange oil in 79% yield (76 mg, 0.40 mmol, *E:Z* 2.7:1, based on NOE NMR spectra).

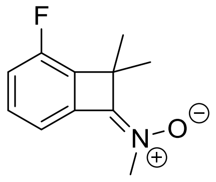

Rf: 0.26 (EtOAc/MeOH 10:1). *Major isomer:* **^{1}H NMR ($CDCl_3$, 400 MHz):** δ 7.27 (m, 1H), 6.93 (m, 2H), 3.80 (s, 3H), 1.63 (s, 6H). **^{13}C NMR ($CDCl_3$, 100 MHz):** δ 155.7 ($^1J_{CF}$ = 255.7 Hz), 137.2 ($^3J_{CF}$ = 9.5 Hz), 136.5 ($^2J_{CF}$ = 19.1 Hz), 131.0 (5.9 Hz), 117.8 (21.0 Hz), 115.6 (4.4 Hz), 54.1, 49.2, 22.4. **^{19}F NMR ($CDCl_3$, 376 MHz):** δ -120.24 (m). *Minor isomer:* **^{1}H NMR ($CDCl_3$, 400 MHz):** δ 7.65 (m, 1H), 7.45 (m, 1H), 7.39 (m, 1H), 3.79 (s, 3H), 1.63 (s, 6H). **^{13}C NMR ($CDCl_3$, 100 MHz):** δ 155.0 ($^1J_{CF}$ = 254.4 Hz), 138.3 ($^3J_{CF}$ = 8.8 Hz), 135.9 ($^2J_{CF}$ = 17.5 Hz), 131.3 (5.9 Hz), 118.9 (20.5 Hz), 118.7 (4.6 Hz), 55.0, 46.8, 23.6. **^{19}F NMR ($CDCl_3$, 376 MHz):** δ -121.88 (m). **IR (CH_2Cl_2, cm^{-1})** ν_{max}: 3411, 3073, 2967, 2927, 2866, 1659, 1580, 1478, 1244, 1057, 791. **MS (EI 70 eV, *m/z*, %)** 193 (M^+, 79), 176 (100), 147 (38), 133 (43), 109 (20); **HRMS (EI$^+$)** *m/z*: $[M]^+$ Calcd for $C_{11}H_{12}NOF$ 193.0903; Found 193.0905.

(*E*)-*N*,6,6-Trimethylcyclobuta[4,5]benzo[1,2-*d*][1,3]dioxol-5(6*H*)-imine oxide (2i)

Following **GP6** from aldonitrone **1i** with dppe as a ligand. The crude was purified by column chromatography to afford the title compound as a orange oil in 95% yield (104 mg, 0.48 mmol, *E:Z* 1:1.4, based on NOE NMR spectra).

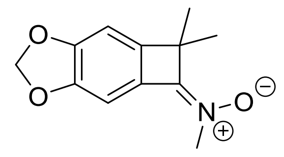

Rf: 0.22 (EtOAc/MeOH 5:1). *Major isomer:* **^{1}H NMR ($CDCl_3$, 400 MHz):** δ 7.07 (s, 1H), 6.64 (s, 1H), 5.90 (s, 2H), 3.54 (s, 3H), 1.46 (s, 6H). **^{13}C NMR ($CDCl_3$, 100 MHz):** δ 151.1, 149.2, 148.6, 148.5, 128.5, 103.6, 101.4, 101.0, 53.1, 45.9, 23.9. *Minor isomer:* **^{1}H NMR ($CDCl_3$, 400 MHz):** δ 6.69 (s, 1H), 6.59 (s, 1H), 5.90 (s, 2H), 3.68 (s, 3H), 1.48 (s, 6H). **^{13}C NMR ($CDCl_3$, 100 MHz):** δ 150.1, 149.7, 148.7, 148.3, 126.9, 102.6, 101.0, 100.7, 52.4, 48.0, 22.9. **IR (CH_2Cl_2, cm^{-1})** ν_{max}: 3378, 2962, 2924, 2865, 1740, 1460, 1308, 1228, 1165, 1040, 987. **MS (EI 70 eV, *m/z*, %)** 219 (M^+, 64), 202 (79), 190 (96), 162 (100), 147 (80), 84 (72); **HRMS (EI$^+$)** *m/z*: $[M]^+$ Calcd for $C_{12}H_{13}NO_3$ 219.0895; Found 219.0891.

(*E*)-*N*,2,2-Trimethylcyclobuta[*a*]naphthalen-1(2*H*)-imine oxide (2j)

Following slightly modified **GP6** from aldonitrone **1j** with *rac*-BINAP as a ligand at 120 °C. The crude was purified by column chromatography to afford the title compound as a orange oil in 59% yield (66 mg, 0.30 mmol, only *E* isomer, based on NOE NMR spectra).

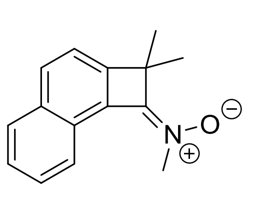

Rf: 0.21 (EtOAc/MeOH 10:1). **^{1}H NMR ($CDCl_3$, 400 MHz):** 9.02 (d, 1H, J = 8.3 Hz), 7.89 (d, 1H, J = 8.1 Hz), 7.80 (d, 1H, J = 8.2 Hz), 7.56 (t, 1H, J = 7.6 Hz), 7.48 (1H, m), 7.32 (d, 1H, J = 8.1 Hz), 3.74 (s, 3H), 1.64 (s, 6H). **^{13}C NMR ($CDCl_3$, 100 MHz):** δ 152.5, 150.9, 134.2, 134.0, 132.9, 128.9, 127.5, 127.4, 127.0, 126.5, 117.0, 54.0, 46.6, 23.9. **IR (CH_2Cl_2, cm^{-1})** ν_{max}: 3382, 3053, 2960, 2925, 2862, 1755, 1515, 1236, 1007, 824, 756. **MS (EI 70 eV, *m/z*, %)** 225 (M^+, 91), 208 (100), 181 (30), 165 (42), 152 (35); **HRMS (EI$^+$)** *m/z*: $[M]^+$ Calcd for $C_{15}H_{15}NO$ 225.1154; Found 225.1156.

(*E*)-*N*-Methylspiro[bicyclo[4.2.0]octane-7,1'-cyclopentane]-1,3,5-trien-8-imine oxide (2k)

Following **GP6** from aldonitrone **1k** with dppe as a ligand. The crude was purified by column chromatography to afford the title compound as a orange oil in 77% yield (81 mg, 0.39 mmol, *E:Z* 10:1, based on NOE NMR spectra).

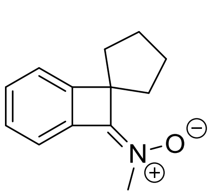

Rf: 0.20 (EtOAc/MeOH 10:1). *Major isomer:* **^{1}H NMR ($CDCl_3$, 400 MHz):** δ 7.27 (m, 2H), 7.16 (m, 1H), 7.08 (m, 1H), 3.80 (s, 3H), 2.45 (m, 2H), 1.98 (m, 2H), 1.80 (m, 4H). **^{13}C NMR ($CDCl_3$, 100 MHz):** δ 153.7, 149.3, 135.7, 131.0, 128.3, 120.3, 118.4, 63.2, 48.9, 34.2, 25.8. *Minor isomer:* **^{1}H NMR ($CDCl_3$, 400 MHz):** δ 7.65 (m, 1H), 7.46 (m, 1H), 7.49 (m, 2H), 3.61 (s, 3H), 2.45 (m, 2H), 1.98 (m, 2H), 1.80 (m, 4H).**IR (CH_2Cl_2, cm^{-1})** ν_{max}: 3396, 3065, 2952, 2866, 1746, 1659, 1441, 1220, 1096, 976, 751. **MS (EI 70 eV, *m/z*, %)** 201 (M^+, 43), 184 (88), 160 (100), 153 (51), 115 (46); **HRMS (EI$^+$)** *m/z*: $[M]^+$ Calcd for $C_{13}H_{15}NO$ 201.1154; Found 201.1156.

(*E*)-*N*-Methylspiro[bicyclo[4.2.0]octane-7,1'-cyclopentane]-1,3,3',5-tetraen-8-imine oxide (2l)

Following **GP6** from aldonitrone **1l** with dppe as a ligand. The crude was purified by column chromatography to afford the title compound as a brown oil in 67% yield (67 mg, 0.34 mmol, *E:Z* 1.7:1, based on NOE NMR spectra).

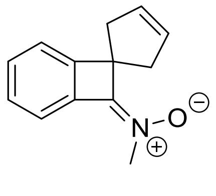

Rf: 0.29 (EtOAc/MeOH 5:1). *Major isomer:* **^{1}H NMR ($CDCl_3$, 400 MHz):** δ 7.28 (m, 2H), 7.23 - 7.15 (m, 1H), 7.11 (m, 1H), 5.80 (s, 1H), 3.83 (s, 3H), 3.24 (d, 2H, J = 14.8 Hz), 2.54 (d, 2H, J = 14.8 Hz). **^{13}C NMR ($CDCl_3$, 100 MHz):** δ 153.2, 148.4,

135.9, 131.2, 129.1, 128.7, 120.1, 118.5, 60.8, 48.9, 40.4. *Minor isomer:* **^{1}H NMR ($CDCl_3$, 400 MHz):** δ 7.60 (m, 1H), 7.39 - 7.30 (m, 2H), 7.23 - 7.15 (m, 1H), 5.83 (s, 1H), 3.58 (s, 3H), 2.93 (AB, 2H, J = 16.5 Hz). **^{13}C NMR ($CDCl_3$, 100 MHz):** δ 151.7, 150.1, 137.3, 132.5, 129.5, 129.1, 121.8, 119.3, 61.2, 46.6, 42.1. **IR (CH_2Cl_2, cm^{-1})** ν_{max}: 3398, 3059, 2921, 2839, 1746, 1685, 1346, 1265, 1168, 967, 745. **MS (EI 70 eV, *m/z*, %)** 199 (M^+, 14), 182 (100), 167 (32), 153 (21), 141 (22), 115 (21); **HRMS (EI^+)** *m/z*: $[M]^+$ Calcd for $C_{13}H_{13}NO$ 199.0997; Found 199.1000.

(*E*)-*N*-Methyl-2',3',5',6'-tetrahydrospiro[bicyclo[4.2.0]octane-7,4'-pyran]-1,3,5-trien-8-imine oxide (2m)

Following **GP6** from aldonitrone **1m** with dppe as a ligand. The crude was purified by column chromatography to afford the title compound as a brown solid in 98% yield (106 mg, 0.49 mmol, *E:Z* 2.2:1, based on NOE NMR spectra).

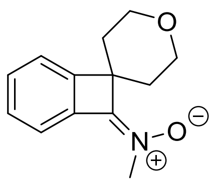

mp 84-86 °C. ***Rf:*** 0.23 (EtOAc/MeOH 5:1). *Major isomer:* **^{1}H NMR ($CDCl_3$, 400 MHz):** δ 7.42 (m, 1H), 7.40 - 7.23 (m, 2H), 7.16 (d, 1H, J = 7.1 Hz), 4.04 (m, 2H), 3.79 (s, 3H), 3.65 (m, 2H), 2.61 (td, 2H, J = 12.8 Hz, 4.6 Hz), 1.58 (d, 2H, J = 13.1 Hz). **^{13}C NMR ($CDCl_3$, 100 MHz):** δ 151.2, 148.4, 134.9, 130.6, 129.0, 122.3, 119.2, 65.1, 56.9, 49.1, 31.8. *Minor isomer:* **^{1}H NMR ($CDCl_3$, 400 MHz):** δ 7.64 (m, 1H), 7.50 (m, 1H), 7.42 (m, 1H), 7.40 - 7.23 (m, 1H), 4.04 (m, 2H), 3.65 (m, 5H), 2.24 (td, 2H, J = 12.8 Hz, 4.7 Hz), 1.73 (d, 2H, J = 13.4 Hz). **^{13}C NMR ($CDCl_3$, 100 MHz):** δ 50.9, 148.1, 136.3, 131.7, 129.3, 122.2, 121.7, 65.3, 58.0, 46.7, 33.1. **IR (CH_2Cl_2, cm^{-1})** ν_{max}: 3399, 3054, 2950, 2848, 1753, 1655, 1441, 1314, 1252, 1101, 753. **MS (EI 70 eV, *m/z*, %)** 217 (M^+, 16), 200 (78), 170 (73), 141 (100), 128 (73), 115 (71); **HRMS (EI^+)** *m/z*: $[M]^+$ Calcd for $C_{13}H_{15}NO_2$ 217.1103; Found 217.1096.

(*E*)-8-Benzyl-*N*,8-dimethylbicyclo[4.2.0]octa-1,3,5-trien-7-imine oxide (2n)

Following **GP6** from aldonitrone **1n** with *rac*-BINAP as a ligand. The crude was purified by column chromatography to afford the title compound as a orange oil in 71% yield (89 mg, 0.36 mmol, *E:Z* 6.6:1, based on NOE NMR spectra).

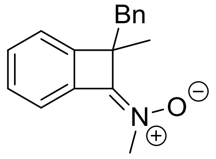

Rf: 0.31 (EtOAc/MeOH 10:1). *Major isomer:* **^{1}H NMR ($CDCl_3$, 400 MHz):** δ 7.27 - 7.16 (m, 2H), 7.16 - 7.01 (m, 6H), 6.94 (d, 2H, J = 7.20 Hz), 3.72 (s, 3H), 3.32 (d, 1H, J = 13.3 Hz), 3.18 (d, 1H, J = 13.3 Hz), 1.65 (s, 3H). **^{13}C NMR ($CDCl_3$, 100 MHz):** δ 150.6, 149.4, 137.5, 134.9, 130.4, 129.6, 128.7, 127.6, 126.1, 122.1, 118.7, 59.1, 48.8, 41.8, 21.4. *Minor isomer:* **^{1}H NMR ($CDCl_3$, 400 MHz):** δ 7.60 (m, 1H), 7.29 (m, 2H), 7.27 - 7.01 (m, 6H), 3.60 (s, 3H), 3.12 (AB, 2H, J = 13.4 Hz), 1.58 (s, 3H). **^{13}C NMR ($CDCl_3$, 100 MHz):** δ 131.5, 129.8, 129.1, 128.1, 126.8, 122.2, 121.1, 59.6, 47.0, 43.6, 21.8 *(signals of quaternary aromatic carbon atoms invisible)*. **IR (CH_2Cl_2, cm^{-1})** ν_{max}: 3404, 3028, 2924, 2863, 1657, 1451, 1347, 1268, 1113, 979, 756. **MS (EI 70 eV, *m/z*, %)** 251 (M^+, 22), 234 (100), 218 (95), 205 (50), 91 (26); **HRMS (EI^+)** *m/z*: $[M]^+$ Calcd for $C_{17}H_{17}NO$ 251.1310; Found 251.1317.

(*E*)-8-Butyl-*N*-methylbicyclo[4.2.0]octa-1,3,5-trien-7-imine oxide (2o)

Following **GP6** from aldonitrone **1o** with dppe as a ligand. The crude was purified by column chromatography to afford the title compound as a orange oil in 27% yield (27 mg, 0.14 mmol, *E:Z* 10:1, based on NOE NMR spectra).

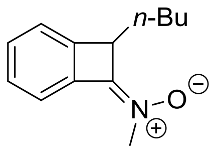

Rf: 0.18 (EtOAc/MeOH 10:1). *Major isomer:* **^{1}H NMR ($CDCl_3$, 400 MHz):** δ 7.67 (m, 1H), 7.38 (m, 1H), 7.33 (m, 2H), 4.26 (dd, 1H, J = 9.5 Hz, 4.2 Hz), 3.83 (s, 3H),

2.26 (m, 1H), 2.01 (m, 1H), 1.38 (m, 4H), 0.89 (m, 3H). *Minor isomer:* **^{1}H NMR ($CDCl_3$, 400 MHz):** δ 7.62 (d, 1H, J = 7.2 Hz), 7.48 (m, 1H), 7.28 (m, 1H), 7.13 (m, 1H), 4.23 (dd, 1H, J = 9.7 Hz, 4.4 Hz), 3.69 (s, 3H), 1.71 (m, 2H), 1.38 (m, 4H), 0.89 (m, 3H). **^{13}C NMR ($CDCl_3$, 100 MHz):** δ 147.2, 146.7, 137.4, 136.3, 132.3, 132.1, 131.3, 130.8, 129.1, 128.8, 123.0, 122.3, 122.0, 119.0, 53.0, 52.2, 48.6, 46.6, 30.6, 29.7, 29.2, 28.9, 22.5, 22.5, 13.9, 13.8. **IR (CH_2Cl_2, cm^{-1})** ν_{max}: 3197, 3063, 2956, 2928, 2858, 1764, 1582, 1463, 1251, 1160, 758. **MS (EI 70 eV, *m/z*, %)** 203 (M^+, 52), 186 (57), 147 (96), 131 (85), 115 (81), 89 (100); **HRMS (EI$^+$)** *m/z*: $[M]^+$ Calcd for $C_{13}H_{17}NO$ 203.1310; Found 203.1309.

(*Z*)-8-Butyl-*N*-methyl-8-(4-nitrophenyl)bicyclo[4.2.0]octa-1,3,5-trien-7-imine oxide (2p)

Following **GP6** from aldonitrone **1p** with dppe as a ligand. The crude was purified by column chromatography to afford the title compound as a brown oil in 75% yield (122 mg, 0.38 mmol, only *Z* isomer, based on NOE NMR spectra).

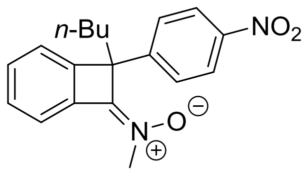

Rf: 0.36 (EtOAc). **^{1}H NMR ($CDCl_3$, 400 MHz):** δ 8.10 (d, 2H, J = 8.8 Hz), 7.88 (d, 2H, J = 8.8 Hz), 7.54 (m, 1H), 7.43 (m, 2H), 7.23 (m, 1H), 3.89 (s, 3H), 2.35 (m, 1H), 2.25 (m, 1H), 1.24 (m, 4H), 0.78 (t, 3H, J = 7.1 Hz). **^{13}C NMR ($CDCl_3$, 100 MHz):** δ 148.8, 147.6, 146.8, 146.7, 136.1, 131.0, 129.7, 128.2, 123.4, 123.0, 119.3, 66.6, 49.4, 36.4, 27.5, 22.6, 13.7. **IR (CH_2Cl_2, cm^{-1})** ν_{max}: 3410, 3060, 2956, 2930, 2858, 1600, 1518, 1346, 1255, 1099, 753. **MS (EI 70 eV, *m/z*, %)** 324 (M^+, 21), 307 (100), 279 (79), 265 (74), 251 (62), 210 (54); **HRMS (EI$^+$)** *m/z*: $[M]^+$ Calcd for $C_{19}H_{20}N_2O_3$ 324.1474; Found 324.1476.

(*E*)-*N*,2,2-Trimethyl-2,3-dihydro-1*H*-inden-1-imine oxide (2q)

Following **GP6** from aldonitrone **1q** with dppe as a ligand. The crude was purified by column chromatography to afford the title compound as a yellow oil in 87% yield (82 mg, 0.44 mmol, *E:Z* 2.5:1, based on NOE NMR spectra).

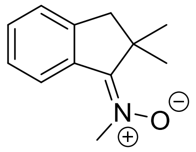

Rf: 0.29 (EtOAc/MeOH 50:1). *Major isomer:* **^{1}H NMR ($CDCl_3$, 400 MHz):** δ 7.41 (m, 1H), 7.29 - 7.20 (m, 3H), 4.01 (s, 3H), 2.90 (s, 2H), 1.52 (s, 6H). **^{13}C NMR ($CDCl_3$, 100 MHz):** δ 156.6, 145.7, 132.4, 129.4, 127.2, 126.1, 124.5, 52.7, 47.0, 44.3, 23.7. *Minor isomer:* **^{1}H NMR ($CDCl_3$, 400 MHz):** δ 8.94 (d, 1H, ^{3}J = 7.9 Hz), 7.34 (dd, 1H, ^{3}J = 7.4 Hz, ^{4}J = 1.2 Hz), 7.29 - 7.20 (m, 1H), 7.17 (m, 1H), 3.89 (s, 3H), 3.04 (s, 2H), 1.43 (s, 6H). **^{13}C NMR ($CDCl_3$, 100 MHz):** δ 155.9, 144.0, 134.5, 131.2, 128.0, 127.0, 124.0, 49.4, 49.2, 41.7, 26.7. **IR (CH_2Cl_2, cm^{-1})** ν_{max}: 3398, 2960, 2929, 1712, 1606, 1466, 1347, 1246, 1036, 1015, 771. **HRMS (ESI$^+$)** *m/z*: $[M + H]^+$ Calcd for $C_{12}H_{16}NO$ 190.1232; Found 190.1240.

(*E*)-*N*-Benzyl-8,8-dimethylbicyclo[4.2.0]octa-1,3,5-trien-7-imine oxide (2r)

Following **GP6** from aldonitrone **1r** with *rac*-BINAP as a ligand. The crude was purified by column chromatography to afford the title compound as a brown oil in 41% yield (52 mg, 0.21 mmol, *E:Z* 4.8:1, based on NOE NMR spectra).

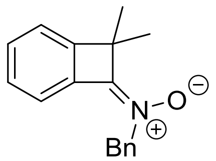

Rf: 0.22 (EtOAc). *Major isomer:* **^{1}H NMR ($CDCl_3$, 400 MHz):** δ 7.47 (m, 2H), 7.34 (m, 5H), 7.26 (m, 1H), 7.21 (m, 1H), 5.11 (s, 2H), 1.62 (s, 6H). **^{13}C NMR ($CDCl_3$, 100 MHz):** δ 153.6, 150.1, 134.6, 133.4, 130.7, 128.8, 128.6, 128.5, 128.3, 120.6, 119.1, 65.7, 54.3, 22.8. *Minor isomer:* **^{1}H NMR ($CDCl_3$, 400 MHz):** δ 7.69 (d, 1H, J = 6.8 Hz), 7.47 (m, 2H), 7.34 (m, 6H), 7.21 (m, 1H), 4.93 (s, 2H), 1.65 (s, 6H). **^{13}C NMR ($CDCl_3$, 100 MHz):** δ 153.0, 150.0, 136.0, 132.9, 132.0, 129.1, 128.8, 128.4, 128.3, 122.7, 119.2, 62.7, 55.5, 24.6.

IR (CH_2Cl_2, cm^{-1}) ν_{max}: 3397, 3061, 2962, 2862, 1647, 1572, 1456, 1345, 1173, 987, 751. **MS (EI 70 eV, *m/z*, %)** 251 (M^+, 39), 144 (53), 129 (18), 116 (34), 91 (100), 66 (19); **HRMS (EI^+)** *m/z*: $[M]^+$ Calcd for $C_{17}H_{17}NO$ 251.1310; Found 251.1307.

(*E*)-8,8-dimethyl-*N*-phenethylbicyclo[4.2.0]octa-1,3,5-trien-7-imine oxide (2s)

Following **GP6** from aldonitrone **1s** with dppe as a ligand. The crude was purified by column chromatography to afford the title compound as a pale-yellow oil in 66% yield (87 mg, 0.33 mmol, *E:Z* 3.2:1, based on NOE NMR spectra).

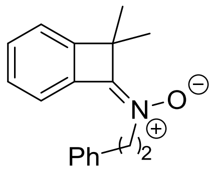

Rf: 0.32 (EtOAc/MeOH 20:1). *Major isomer:* **^{1}H NMR ($CDCl_3$, 400 MHz):** δ 7.24 (m, 3H), 7.17 (m, 4H), 7.11 (m, 1H), 6.89 (d, 1H, J = 7.2 Hz), 4.14 (t, 2H, J = 6.9 Hz), 3.29 (t, 2H, J = 6.9 Hz), 1.54 (s, 6H). **^{13}C NMR ($CDCl_3$, 100 MHz):** δ 153.3, 150.5, 137.4, 134.2, 130.5, 128.8, 128.6, 128.4, 126.7, 120.3, 118.9, 62.6, 54.0, 33.1, 22.7. *Minor isomer:* **^{1}H NMR ($CDCl_3$, 400 MHz):** δ 7.69 (m, 1H), 7.33 (m, 2H), 7.24 (2H, m), 7.17 (m, 3H), 7.11 (m, 1H), 3.90 (t, 2H, J = 7.2 Hz), 3.32 (t, 2H, J = 7.2 Hz), 1.33 (s, 6H). **^{13}C NMR ($CDCl_3$, 100 MHz):** δ 153.1, 150.0, 137.7, 135.8, 131.9, 129.0, 128.9, 128.4, 126.8, 122.5, 119.2, 60.3, 55.2, 33.2, 24.0. **IR (CH_2Cl_2, cm^{-1})** ν_{max}: 3406, 3061, 2961, 2863, 1746, 1650, 1574, 1456, 1223, 1146, 750. **MS (EI 70 eV, *m/z*, %)** 265 (M^+, 24), 144 (100), 116 (38), 104 (49), 91 (21), 77 (23); **HRMS (EI^+)** *m/z*: $[M]^+$ Calcd for $C_{18}H_{19}NO$ 265.1467; Found 265.1463.

(*E*)-*N*-(4-Methoxy-4-oxobutyl)-8,8-dimethylbicyclo[4.2.0]octa-1,3,5-trien-7-imine oxide (2t)

Following **GP6** from aldonitrone **1t** with dppe as a ligand. The crude was purified by column chromatography to afford the title compound as a pale-yellow oil in 78% yield (102 mg, 0.39 mmol, *E:Z* 7.2:1, based on NOE NMR spectra).

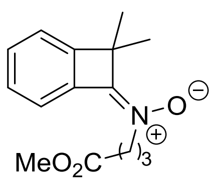

Rf: 0.29 (EtOAc/MeOH 20:1). *Major isomer:* **^{1}H NMR ($CDCl_3$, 400 MHz):** δ 7.29 (m, 2H), 7.23 (m, 1H), 7.17 (m, 1H), 4.05 (t, 2H, J = 6.6 Hz), 3.55 (s, 3H), 2.44 (t, 2H, J = 6.7 Hz), 2.30 (m, 2H), 1.59 (s, 6H). **^{13}C NMR ($CDCl_3$, 100 MHz):** δ 173.1, 153.5, 150.6, 134.4, 130.7, 128.7, 120.5, 119.5, 59.8, 54.2, 51.5, 30.0, 22.8, 21.9. *Minor isomer:* **^{1}H NMR ($CDCl_3$, 400 MHz):** δ 7.29 (m, 2H), 7.23 (m, 1H), 7.17 (m, 1H), 3.82 (t, 2H, J = 7.1 Hz), 3.65 (s, 3H), 2.50 (t, 2H, J = 6.8 Hz), 2.30 (m, 2H), 1.59 (s, 6H). **IR (CH_2Cl_2, cm^{-1})** ν_{max}: 3406, 2959, 2864, 1734, 1647, 1576, 1439, 1248, 1150, 1022, 754. **MS (EI 70 eV, *m/z*, %)** 261 (M^+, 27), 244 (18), 158 (100), 144 (43), 129 (85), 60 (48); **HRMS (EI^+)** *m/z*: $[M]^+$ Calcd for $C_{15}H_{19}NO_3$ 261.1365; Found 261.1369.

8',8'-Dimethyl-7-oxa-1-azaspiro[bicyclo[2.2.1]heptane-2,7'-bicyclo[4.2.0]octane]-1',3',5'-triene (2u)

Following **GP6** from aldonitrone **1u**. The crude was purified by column chromatography to afford the title compound as a single diastereoisomer, orange solid in 15% yield (16 mg, 0.08 mmol) when dppe was used as a ligand and in 55% yield (60 mg, 0.28 mmol) when *rac*-BINAP was used as a ligand.

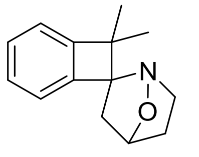

mp 47-49 °C. ***Rf:*** 0.26 (Hexane/EtOAc 2:1). **^{1}H NMR ($CDCl_3$, 400 MHz):** δ 7.27 (m, 1H), 7.22 (m, 1H), 7.12 (m, 2H), 4.93 (t, 1H, ^3J = 5.2 Hz), 3.47 (ddd, 1H, ^2J = 11.8 Hz, ^3J = 8.6 Hz, 4.4 Hz), 3.09 (ddd, 1H, ^2J = 11.8 Hz, ^3J = 10.9 Hz, 5.1 Hz), 2.45 (ddd, 1H, ^2J = 12.4 Hz, ^3J = 5.3 Hz, ^4J = 2.6 Hz), 2.03 (m, 1H), 1.78 (ddd, 1H, ^2J = 11.1 Hz, ^3J = 8.6 Hz, 5.1 Hz), 1.64 (d, 1H, ^2J = 12.4 Hz), 1.52 (s, 3H), 1.22 (s, 3H). **^{13}C NMR ($CDCl_3$, 100 MHz):** δ 154.5, 142.1, 129.1, 127.4, 122.6, 120.5, 81.2, 80.1, 52.8, 51.0, 40.7, 32.9, 25.5, 23.2. **IR (CH_2Cl_2, cm^{-1})** ν_{max}: 2957, 2862, 1775, 1601, 1455, 1345, 1283, 1253, 1040, 876, 747. **HRMS (ESI^+)** *m/z*: $[M + H]^+$

Calcd for $C_{14}H_{18}NO$ 216.1388; Found 216.1393. **Anal. calcd for $C_{14}H_{17}NO$:** C, 78.10; H, 7.96; N, 6.51;. Found: C, 77.92; H, 7.86; N, 6.35.

9,9-Dimethyl-3,3a,4,9-tetrahydro-2*H*-benzo[*f*]indole 1-oxide (2u')

Following **GP6** from aldonitrone **1u** with *rac*-BINAP as a ligand. The crude was purified by column chromatography to afford the title compound as a pale-brown solid in 29% yield (32 mg, 0.15 mmol).

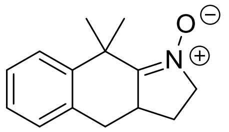

mp 125-127 °C. ***Rf:*** 0.17 (EtOAc/MeOH 10:1). **^{1}H NMR ($CDCl_3$, 400 MHz):** δ 7.39 (d, 1H, J = 7.8 Hz), 7.26 (t, 1H, J = 7.5 Hz), 7.14 (td, 1H, J = 7.5 Hz, 1.0 Hz), 7.09 (d, 1H, J = 7.5 Hz), 4.15 (dddd, 1H, J = 13.2 Hz, 10.8 Hz, 8.2 Hz, 2.5 Hz), 3.99 (m, 1H), 3.14 (m, 1H), 3.03 (dd, 1H, J = 14.7 Hz, 5.2 Hz), 2.67 (m, 1H), 2.40 (dtd, 1H, J = 12.7 Hz, 8.1 Hz, 1.8 Hz), 1.84 (m, 1H), 1.76 (s, 3H), 1.68 (s, 3H). **^{13}C NMR ($CDCl_3$, 100 MHz):** δ 151.6, 144.7, 133.8, 128.5, 127.5, 126.2, 125.6, 77.4, 77.1, 76.8, 63.6, 41.3, 38.2, 36.9, 25.8, 24.9, 21.7. **IR (CH_2Cl_2, cm^{-1})** ν_{max}: 3389, 2966, 2869, 1753, 1595, 1444, 1281, 1219, 1086, 996, 768. **MS (EI 70 eV, *m/z*, %)** 215 (M^+, 71), 200 (79), 170 (34), 157 (100), 142 (54), 115 (38); **HRMS (EI^+)** *m/z*: $[M]^+$ Calcd for $C_{14}H_{17}NO$ 215.1310; Found 215.1310.

2,11,11-trimethyl-5,6-dihydro-2*H*,4*H*-1,4:1,10-dimethanobenzo[*d*][1,2]oxazocine (2v)

Following **GP6** from aldonitrone **1v** with *rac*-BINAP as a ligand. The crude was purified by column chromatography to afford the title compound as a mixture of two diastereoisomers in a ratio about 2.5:1, pale-yellow crystals in 24% yield (28 mg, 0.12 mmol).

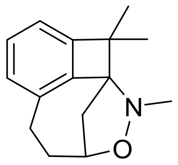

mp 113-115 °C (recrystallized from DCM/Heptane). ***Rf:*** 0.33 (Hexane/EtOAc 2:1). *Major diastereoisomer:* **^{1}H NMR ($CDCl_3$, 400 MHz):** δ 7.09 (t, 1H, J = 7.5 Hz), 6.96 (d, 1H, J = 7.7 Hz), 6.88 (d, 1H, J = 7.3 Hz), 4.90 (t, 1H, J = 8.2 Hz), 3.28 (m, 1H), 2.86 (m, 1H), 2.72 (s, 3H), 2.38 (m, 1H), 2.24 (m, 1H), 1.84 (d, 1H, J = 11.6 Hz), 1.64 (m, 1H), 1.56 (s, 3H), 1.39 (s, 3H). **^{13}C NMR ($CDCl_3$, 100 MHz):** δ 148.1, 146.5, 136.0, 128.4, 126.0, 117.3, 76.7, 75.6, 50.2, 42.6, 38.8, 32.6, 25.9, 25.2, 24.7.

Minor diastereoisomer: **^{1}H NMR ($CDCl_3$, 400 MHz):** δ 7.15 (t, 1H, J = 7.5 Hz), 7.02 (d, 1H, J = 7.6 Hz), 6.92 (d, 1H, J = 7.3 Hz), 4.68 (t, 1H, J = 7.5 Hz), 3.20 (m, 1H), 2.69 (s, 3H), 2.64 (m, 1H), 2.54 (m, 1H), 2.15 (m, 1H), 1.94 (d, 1H, J = 11.2 Hz), 1.58 (m, 1H), 1.51 (s, 3H), 1.39 (s, 3H). **^{13}C NMR ($CDCl_3$, 100 MHz):** δ 150.2, 149.4, 137.4, 129.0, 126.0, 118.0, 76.7, 74.9, 52.7, 45.3, 42.6, 31.6, 27.5, 26.0, 23.1. **IR (CH_2Cl_2, cm^{-1})** ν_{max}: 3039, 2955, 2923, 2863, 1668, 1461, 1362, 1244, 845, 769, 733. **MS (EI 70 eV, *m/z*, %)** 229 (M^+, 49), 200 (69), 186 (86), 155 (77), 141 (100), 128 (61); **HRMS (EI^+)** *m/z*: $[M]^+$ Calcd for $C_{15}H_{19}NO$ 229.1467; Found 229.1458. **Anal. calcd for $C_{15}H_{19}NO$:** C, 78.56; H, 8.35; N, 6.11;. Found: C, 78.58; H, 8.31; N, 6.04.

1,9,9-trimethyl-3,3a,4,5-tetrahydro-1*H*,9*H*-cyclobuta[8,1]naphtho[1,2-*c*]isoxazole (2v')

Following **GP6** from aldonitrone **1v** with *rac*-BINAP as a ligand. The crude was purified by column chromatography to afford the title compound as a white solid in 28% yield (32 mg, 0.14 mmol).

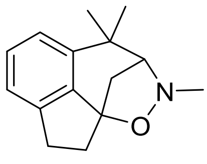

mp 87-89 °C. ***Rf:*** 0.28 (Hexane/EtOAc 10:1). **^{1}H NMR ($CDCl_3$, 400 MHz):** δ 7.23 (t, 1H, J = 7.5 Hz), 7.09 (d, 1H, J = 7.6 Hz), 7.04 (d, 1H, J = 7.4 Hz), 3.20 (m, 1H), 2.86 (m, 2H), 2.78 (s, 3H), 2.38 (m, 3H), 2.20 (d, 1H, J = 11.1 Hz), 1.46 (s, 3H), 1.23 (s, 3H). **^{13}C NMR ($CDCl_3$, 100 MHz):** δ 142.1, 140.8, 140.6, 129.7, 123.3, 122.2, 88.1, 75.3, 47.7, 41.0, 34.0, 30.6, 30.0, 28.8, 26.2. **IR (CH_2Cl_2, cm^{-1})** ν_{max}: 3028, 2947,

2864, 2771, 1610, 1477, 1456, 1315, 1162, 915, 767. **MS (EI 70 eV, *m/z*, %)** 229 (M^+, 3), 183 (100), 168 (42), 153 (15), 128 (8); **HRMS (EI^+)** *m/z*: $[M]^+$ Calcd for $C_{15}H_{19}NO$ 229.1467; Found 229.1459.

Deuterated substrates:

2-(2-Bromophenyl)-2-methylpropan-1-*d*-1-ol (S7)

A 100 mL round-bottom flask was charged with the solution of 2-(2-bromophenyl)-2-methylpropanal (1.136 g, 5 mmol, 1 equiv.) in MeOH (20 mL). Then $NaBD_4$ (209 mg, 5 mmol, 1 equiv.) was added in portions and the resulting suspension was stirred for 2 h at room temperature. After that time, the reaction mixture was diluted with H_2O (ca. 50 mL), transferred into a separatory funnel and extracted with EtOAc (2 x 50 mL). The combined organic phases were washed with brine and dried over anhydrous Na_2SO_4. The solution was filtered, concentrated under reduced pressure and purified by column chromatography (silica gel, hexanes/EtOAc) to afford the title compound as a colorless oil in 77% yield (898 mg, 3.9 mmol, 90 atom% D).

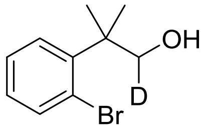

Rf: 0.16 (Hexane/EtOAc 10:1). **^{1}H NMR ($CDCl_3$, 400 MHz):** δ 7.59 (dd, 1H, ^3J = 7.9 Hz, ^4J = 1.4 Hz), 7.46 (dd, 1H, ^3J = 8.0 Hz, ^4J = 1.6 Hz), 7.26 (m, 1H), 7.05 (ddd, 1H, ^3J = 7.8 Hz, 7.3 Hz, ^4J = 1.7 Hz), 3.99 (s, 1H) (*H*), 3.96 (m, 1H) (*D*), 1.76 (s, 1H), 1.49 (s, 6H). **^{13}C NMR ($CDCl_3$, 100 MHz):** δ 143.7, 135.7, 130.0, 127.9, 127.3, 122.2, 69.0 (t, $^1J_{CD}$ = 22.0), 42.0, 25.02, 25.00. **IR (CH_2Cl_2, cm^{-1})** ν_{max}: 3371, 2966, 2877, 2166, 1562, 1468, 1422, 1263, 1055, 1017, 754. **MS (EI 70 eV, *m/z*, %)** 244 (9), 229 (M^+, 1), 199 (84), 169 (100), 150 (65), 115 (39); **HRMS (EI^+)** *m/z*: $[M]^+$ Calcd for $C_{12}H_{12}DOBr$ 229.0213; Found 229.0203.

2-(2-Bromophenyl)-2-methylpropanal-1-*d* (S3a-*d*$_1$)

To a mixture of PCC (1.509 g, 7 mmol, 2 equiv.) and silica gel (1 g) in DCM (7 mL) was added dropwise the solution of 2-(2-bromophenyl)-2-methylpropan-1-*d*-1-ol **S7** (805 mg, 3.5 mmol, 1 equiv.) in DCM (7 mL) and stirred at room temperature for 2 h. Then, the reaction was filtered through a Celite® pad and the filtrate was concentrated under reduced pressure. The crude was purified by column chromatography to afford the title compound as a colorless oil in 86% yield (684 mg, 3.0 mmol, 86 atom% D).

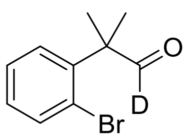

Rf: 0.56 (Hexane/EtOAc 20:1). **^{1}H NMR ($CDCl_3$, 400 MHz):** δ 9.75 (s, 0.14H), 7.56 (dd, 1H, J = 7.9 Hz, 1.4 Hz), 7.39 (dd, 1H, J = 7.9 Hz, 1.8 Hz), 7.34 (ddd, 1H, J = 7.9 Hz, 7.2 Hz, 1.3 Hz), 7.14 (ddd, 1H, J = 7.9 Hz, 7.2 Hz, 1.8 Hz), 1.48 (s, 6H). **^{13}C NMR ($CDCl_3$, 100 MHz):** δ 202.7 (H), 202.5 (t, $^1J_{CD}$ = 27.3 Hz; D), 142.2, 134.2, 129.0, 128.5, 127.7, 123.2, 51.6 (H), 51.4 (t, $^2J_{CD}$ = 3.2 Hz; D), 23.0. **IR (CH_2Cl_2, cm^{-1})** ν_{max}: 3409, 3065, 2977, 2121, 2053, 1712, 1468, 1427, 1021, 950, 757. **HRMS ($APCI^+$)** *m/z*: $[M + H]^+$ Calcd for $C_{10}H_{11}DOBr$ 228.0134; Found 228.0133.

(*Z*)-2-(2-Bromophenyl)-*N*,2-dimethylpropan-1-imine oxide-1-*d* (1a-*d*$_1$)

Following **GP4** from **S3a-*d*$_1$**. The crude was purified by column chromatography to afford the title compound as a white solid in 87% yield (450 mg, 1.75 mmol, 87 atom% D).

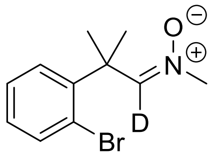

mp 76-78 °C. ***Rf:*** 0.29 (EtOAc/MeOH 10:1). **^{1}H NMR ($CDCl_3$, 500 MHz):** δ 7.50 (dd, J = 7.9, 1.2 Hz, 1H), 7.45 (dd, J = 8.0, 1.5 Hz, 1H), 7.29 (m, 1H), 7.04 (td, J = 7.7, 1.6 Hz, 1H), 3.60 (s, 3H), 1.66 (s, 6H). **^{13}C NMR ($CDCl_3$, 100 MHz):** δ 144.4, 144.3 (H), 144.0 (t, $^1J_{CD}$ = 27.8 Hz; D), 134.0, 127.7, 127.7, 127.5, 120.8, 52.4, 40.8, 24.4. **IR (CH_2Cl_2, cm^{-1})** ν_{max}: 3395, 3062, 2970, 2278, 1596, 1468, 1428, 1312, 1198,

1020, 757. **HRMS (ESI$^+$)** *m/z*: [M + Na]$^+$ Calcd for $C_{11}H_{13}DNONaBr$ 279.0219; Found 279.0221.

3.3 Scale-up procedure

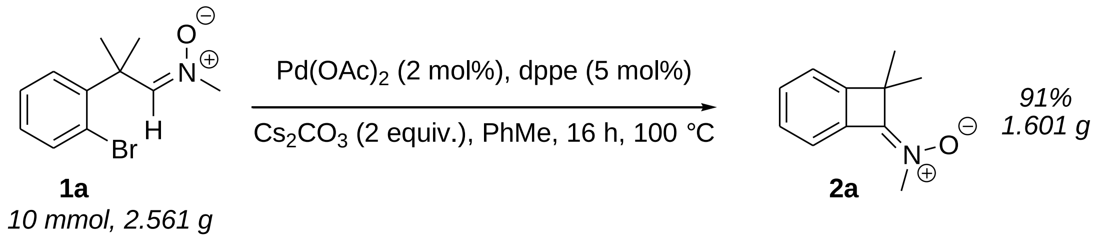

Under argon atmosphere, a flame-dried Schlenk flask was charged with anhydrous PhMe (20 mL), Cs_2CO_3 (6.516 g, 20 mmol, 2 equiv.), dppe (199 mg, 0.5 mmol, 0.05 equiv.), $Pd(OAc)_2$ (44.9 mg, 0.2 mmol, 0.02 equiv.) and aldonitrone **1a** (2.561 g, 10 mmol, 1 equiv.). The mixture was stirred at 100 °C for 16 h (oil bath). After the reaction was complete, the crude was cooled down to room temperature, diluted with DCM (ca. 30 mL), filtered through a pad of Celite® and concentrated under reduced pressure. The resulting crude material was purified by column chromatography (silica gel, hexanes/EtOAc) to afford nitrone **2a** as inseparable mixture of *E*, *Z* isomers in 91% yield (1.601 g, 9.1 mmol).

3.4 Application of ketonitrones

2',5',8,8-Tetramethyl-3a',6a'-dihydro-2'*H*,4'*H*-spiro[bicyclo[4.2.0]octane-7,3'-pyrrolo[3,4-*d*]isoxazole]-1,3,5-triene-4',6'(5'*H*)-dione (3)

An oven-dried sealed tube was charged with ketonitrone **2a** (88 mg, 0.5 mmol, 1.0 equiv.), *N*-methylmaleimide (61 mg, 0.55 mmol, 1.1 equiv.) and anhydrous toluene (2 mL). The reaction mixture was stirred at 80 °C for 2 h (oil bath). After cooling to room temperature, the crude mixture was concentrated under reduced pressure and purified by column chromatography to afford the title compound **3** as a single diastereoisomer, white crystals in 81% yield (117 mg, 0.41 mmol).

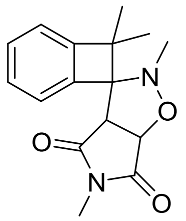

mp 175-177 °C (recrystallized from EtOH). ***Rf:*** 0.37 (Hexane/EtOAc 2:1). **^{1}H NMR (CDCl$_3$, 400 MHz):** δ 7.34 (td, 1H, J = 7.4 Hz, 0.9 Hz), 7.24 (td, 1H, J = 7.5 Hz, 1.0 Hz), 7.12 (dm, 1H, J = 7.4 Hz), 7.08 (dm, 1H, J = 7.4 Hz), 4.84 (1H, d, J = 7.7 Hz), 3.97 (1H, d, J = 7.7 Hz), 2.99 (s, 3H), 2.70 (s, 3H), 1.50 (s, 3H), 1.41 (s, 3H). **^{13}C NMR (CDCl$_3$, 100 MHz):** δ 175.0, 174.5, 152.5, 136.9, 130.3, 127.7, 125.2, 120.3, 81.9, 77.0, 53.9, 52.3, 43.2, 25.5, 25.2, 23.3. **IR (CH_2Cl_2, cm^{-1})** ν_{max}: 2958, 2868, 1785, 1709, 1433, 1381, 1281, 1136, 1026, 758, 734. **MS (EI 70 eV, *m/z*, %)** 283 (M$^+$, 3), 271 (66), 243 (48), 186 (85), 158 (100), 115 (46); **HRMS (EI$^+$)** *m/z*: [M]$^+$ Calcd for $C_{16}H_{18}N_2O_3$ 286.1317; Found 286.1311.

2,8',8'-Trimethyl-2*H*-spiro[benzo[*d*]isoxazole-3,7'-bicyclo[4.2.0]octane]-1',3',5'-triene (4)

A modified procedure adopted from literature was used.[12] A flame-dried Schlenk flask was charged with 2-(trimethylsilyl)phenyl trifluoromethanesulfonate (298 mg, 1.0 mmol, 2.0 equiv.) and 18-crown-6 (396 mg, 1.5 mmol, 3.0 equiv.). The reactants were dissolved in THF (5 mL) and allowed to stir at 25 °C for 5 min. CsF (228 mg, 1.5 mmol, 3.0 equiv.) was then added as a solid and the reaction mixture was stirred for another 5 min at 25 °C. After generation of the benzyne reagent, a solution of ketonitrone **2a** (88 mg, 0.5 mmol, 1 equiv.) in 2.5 mL of THF was added dropwise and the reaction

mixture was stirred for 2 h at room temperature. Then, it was concentrated under reduced pressure and the product purified by column chromatography to afford the title compound **4** as a pale-orange solid in 88% yield (111 mg, 0.44 mmol).

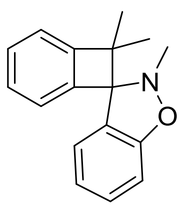

mp 84-86 °C. ***Rf:*** 0.24 (Hexane). **^{1}H NMR ($CDCl_3$, 400 MHz):** δ 7.39 (m, 1H), 7.31 (m, 1H), 7.23 (m, 1H), 7.19 (m, 2H), 6.90 (m, 2H), 6.79 (dd, 1H, J = 7.7 Hz, 1.3 Hz), 2.97 (s, 3H), 1.56 (s, 3H), 1.04 (s, 3H). **^{13}C NMR ($CDCl_3$, 100 MHz):** δ 156.7, 154.3, 140.3, 129.8, 128.9, 128.1, 128.0, 125.6, 125.0, 120.8, 120.7, 108.5, 83.9, 56.1, 44.0, 25.3, 23.5. **IR (CH_2Cl_2, cm^{-1})** ν_{max}: 3066, 2968, 2918, 2856, 1595, 1455, 1219, 1069, 1015, 850, 747. **HRMS (ESI$^+$)** *m/z*: $[M + H]^+$ Calcd for $C_{17}H_{18}NO$ 252.1388; Found 252.1383.

2-((Methylimino)(2-(prop-1-en-2-yl)phenyl)methyl)phenol (5)

A flame-dried Schlenk flask was charged with cesium fluoride (152 mg, 1 mmol, 2 equiv.), 2-(Trimethylsilyl)phenyl trifluoromethanesulfonate (179 mg, 0.6 mmol, 1.2 equiv.) and solution of ketonitrone **2a** (88 mg, 0.5 mmol, 1 equiv.) in anhydrous toluene (2mL). The Schlenk flask was tightly closed and the reaction mixture was stirred at 120 °C for 12 h (oil bath). After cooling to room temperature, the crude was filtered through a short Celite pad, concentrated under reduced pressure and purified by column chromatography to afford the title compound **5** as a yellow solid in 50% yield (63 mg, 0.25 mmol).

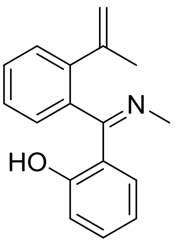

mp 72-74 °C. ***Rf:*** 0.42 (Hexane/EtOAc 5:1). **^{1}H NMR ($CDCl_3$, 400 MHz):** 15.8 (bs, 1H), 7.49 - 7.34 (m, 3H), 7.24 (m, 1H), 7.07 (dm, 1H, J = 7.5 Hz), 6.97 (dm, 1H, J = 8.3 Hz), 6.80 (dd, 1H, J = 8.0 Hz, 1.7 Hz), 6.61 (m, 1H), 5.00 (m, 1H), 4.86 (m, 1H), 3.12 (s, 3H), 1.92 (m, 3H). **^{13}C NMR ($CDCl_3$, 100 MHz):** δ 175.8, 163.7, 143.3, 142.0, 132.2, 131.2, 131.1, 129.2, 128.4, 127.9, 127.2, 120.0, 118.2, 116.9, 116.2, 38.1, 23.3. **IR (CH_2Cl_2, cm^{-1})** ν_{max}: 3060, 2969, 2919, 2569, 1608, 1499, 1449, 1305, 1256, 903, 756. **HRMS (ESI$^+$)** *m/z*: $[M + H]^+$ Calcd for $C_{17}H_{18}NO$ 252.1388; Found 252.1390.

7-(Hydroxy(methyl)amino)-8,8-dimethylbicyclo[4.2.0]octa-1,3,5-triene-7-carbonitrile (6)

A flame-dried Schlenk flask was charged with a solution of ketonitrone **2a** (88 mg, 0.5 mmol, 1 equiv.) in anhydrous DCM (2mL). Then the stirred solution was cooled down to 0 °C, TMSCN (125 µL, 1 mmol, 2 equiv.) was added and the resulting mixture was allowed to warm to room temperature over the course of 2 h. After that time, 2M HCl solution (2 mL) and MeOH (2 mL) were added and stirred for another 1 h at room temperature. The reaction mixture was diluted with DCM (10 mL), transferred into a separatory funnel and extracted with saturated aqueous sodium bicarbonate solution (10 mL). The organic phase was dried over anhydrous Na_2SO_4, filtered and concentrated under reduced pressure. The crude was purified by column chromatography to afford the title compound **6** as a white solid in 75% yield (77 mg, 0.38 mmol).

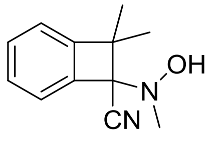

mp 103-105 °C. ***Rf:*** 0.19 (Hexane/EtOAc 5:1). **^{1}H NMR ($CDCl_3$, 400 MHz):** δ 7.37 (m, 1H), 7.27 (m, 1H), 7.23 (d, 1H, J = 7.4 Hz), 7.13 (dm, 1H, J = 7.4 Hz), 5.60 (bs, 1H), 2.89 (s, 3H), 1.60 (s, 3H), 1.51 (s, 3H). **^{13}C NMR ($CDCl_3$, 100 MHz):** δ 151.7, 130.9, 128.4, 123.2, 123.2, 120.9, 116.2, 75.2, 54.2, 44.5, 25.4, 22.6. **IR (CH_2Cl_2, cm^{-1})** ν_{max}: 3422, 2968, 2926, 2882, 2235, 1456, 1246, 1096, 1024, 856, 766. **HRMS (APCI$^+$)** *m/z*: $[M + H]^+$ Calcd for $C_{12}H_{15}N_2O$ 203.1184; Found 203.1180.

8-Benzyl-5',5'-difluoro-2',8-dimethyl-4'-(trifluoromethyl)spiro[bicyclo[4.2.0]octane-7,3'-isoxazolidine]-1,3,5-triene (7)

A modified procedure adopted from literature was used.[13] 2*H*-Pentafluoropropene (ca. 0.5 mL, 5 mmol) was condensed in a glass pressure tube at -78 °C under argon atmosphere. DMF (1.5 mL) and ketonitrone **2n** (126 mg, 0.5 mmol, 1 equiv.) were introduced and the pressure tube was closed with a Teflon valve. The reaction mixture was stirred at room temperature for 7 days. After that time, the tube was cooled down to -78 °C, carefully opened and slowly warmed to room temperature. The reaction mixture was diluted with EtOAc (10 mL), transferred to separatory funnel and washed with brine (2 x 10mL) and the combined organic phases were dried over anhydrous Na_2SO_4. Volatiles were removed under reduced pressure and the crude was purified by column chromatography to afford the title compound as a mixture of two diastereoisomers in 6.3:1 ratio, colorless oil, 72% yield (138 mg, 0.36 mmol).

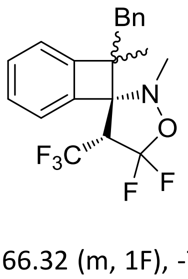

Rf: 0.70 (Hexane/EtOAc 100:1). *Major diastereoisomer:* **^{1}H NMR ($CDCl_3$, 400 MHz):** δ 7.33 (m, 6H), 7.22 (m, 2H), 6.79 (m, 1H), 3.99 (sextet, 1H, $^3J_{HF}$ = 10.4 Hz), 3.47 (d, 1H, $^2J_{HH}$ = 13.6 Hz), 3.18 (m, 3H), 2.91 (d, 1H, $^2J_{HH}$ = 13.6 Hz), 1.31 (s, 3H). **^{13}C NMR ($CDCl_3$, 100 MHz):** δ 151.7, 138.9, 135.6, 131.3 (dd, $^1J_{CF}$ = 283.2 Hz, 257.5 Hz), 130.64, 130.63, 130.57, 128.3, 128.1, 126.3, 123.2 (q, $^1J_{CF}$ = 278.5 Hz), 122.9, 80.7 (m), 59.2, 55.4 (m), 44.0, 41.5, 23.3. **^{19}F NMR ($CDCl_3$, 376 MHz):** δ -62.85 (m, 3F), -66.32 (m, 1F), -72.05 (dd, 1F, $^2J_{FF}$ = 145.8 Hz, $^3J_{FH}$ = 12.4 Hz). *Minor diastereoisomer:* **^{1}H NMR ($CDCl_3$, 400 MHz):** δ 7.33 (m, 6H), 7.16 (m, 2H), 6.74 (m, 1H), 4.11 (sextet, 1H, $^3J_{HF}$ = 10.3 Hz), 3.06 (m, 3H), 2.88 (m, 1H), 2.83 (d, 1H, $^2J_{HH}$ = 11.8 Hz), 1.44 (s, 3H). **^{13}C NMR ($CDCl_3$, 100 MHz):** δ 151.6, 137.1, 135.8, 130.3, 126.8, 123.1, 80.9 (m), 58.8, 45.1, 41.5, 19.9 *(other signals invisible)*. **^{19}F NMR ($CDCl_3$, 376 MHz):** δ -62.55 (m, 3F), -67.44 (m, 1F), -71.60 (dd, 1F, $^2J_{FF}$ = 145.8 Hz, $^3J_{FH}$ = 10.8 Hz). **IR (CH_2Cl_2, cm^{-1})** ν_{max}: 3064, 3030, 2870, 1604, 1456, 1375, 1299, 1192, 1068, 748, 704. **HRMS (APCI$^+$)** *m/z*: [M + H]$^+$ Calcd for $C_{20}H_{19}NOF_5$ 384.1387; Found 384.1384.

Reduction of isoxazolidine 7:

Under an argon atmosphere, palladium on charcoal (120 mg, 10 mol%) was added to a solution of isoxazolidine **7** (115 mg, 0.3 mmol, 1 equiv.) in EtOH (3 mL) in a round-bottom flask. Argon was then replaced by hydrogen and the reaction mixture was stirred vigorously at room temperature at 1 atm of H_2 for 16 h. The flask was again filled with argon and the reaction mixture was filtered through a pad of Celite under reduced pressure. After evaporation of EtOH, the products were purified by column chromatography on silica gel.

(3*S*,8'*R*)-8'-Benzyl-1,8'-dimethyl-3-(trifluoromethyl)spiro[azetidine-2,7'-bicyclo[4.2.0]octane]-1',3',5'-trien-4-one) (8)

The title compound was obtained as a colorless crystals in 75% yield (79 mg, 0.23 mmol).

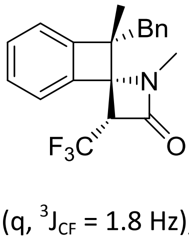

mp 125-127 °C (recrystallized from DCM/Heptane). ***Rf:*** 0.25 (Hexane/EtOAc 10:1). **^{1}H NMR ($CDCl_3$, 400 MHz):** δ 7.42 - 7.28 (m, 6H), 7.21 (m, 2H), 6.96 (m, 1H), 4.04 (q, 1H, $^3J_{HF}$ = 9.4 Hz), 3.00 (d (AB), 1H, $^2J_{HH}$ = 13.0 Hz), 2.94 (m, 4H), 1.29 (s, 3H). **^{13}C NMR ($CDCl_3$, 100 MHz):** δ 161.1 (q, $^3J_{CF}$ = 4.4 Hz), 152.0, 137.4, 136.6, 130.9, 130.0, 128.7, 128.3, 126.8, 125.2 (q, $^4J_{CF}$ = 2.9 Hz), 123.5 (q, $^1J_{CF}$ = 276.6 Hz), 122.3, 73.1 (q, $^3J_{CF}$ = 1.8 Hz), 57.7 (q, $^2J_{CF}$ = 29.6 Hz), 57.5, 42.5, 28.1, 23.5. **^{19}F NMR ($CDCl_3$, 376 MHz):** δ -65.38 (d, $^3J_{FH}$ = 9.5 Hz). **IR (CH_2Cl_2, cm^{-1})** ν_{max}: 3064, 3030, 2967, 1779, 1454, 1344, 1258, 1177, 1133, 763, 706. **MS (EI 70 eV, *m/z*, %)** 345 (M$^+$, 31), 288 (33), 226 (62), 204 (100), 158 (68), 91 (37); **HRMS (EI$^+$)** *m/z*:

$[M]^+$ Calcd for $C_{20}H_{18}NOF_3$ 345.1340; Found 345.1337. **Anal. calcd for $C_{20}H_{18}NOF_3$:** C, 69.56; H, 5.25; N, 4.06; F, 16.50. Found: C, 69.65; H, 5.21; N, 4.01; F, 16.44.

(3*R*,8'*R*)-8'-benzyl-1,8'-dimethyl-3-(trifluoromethyl)spiro[azetidine-2,7'-bicyclo[4.2.0]octane]-1',3',5'-trien-4-one (8')

The title compound was obtained as a white solid in 10% yield (10 mg, 0.03 mmol).

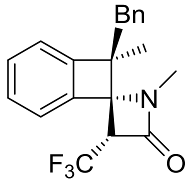

mp 176-178 °C. ***Rf:*** 0.21 (Hexane/EtOAc 10:1). **^{1}H NMR (CDCl$_3$, 400 MHz):** δ 7.40 - 7.22 (m, 6H), 7.13 (m, 2H), 6.74 (m, 1H), 4.15 (q, 1H, $^3J_{HF}$ = 9.4 Hz), 3.00 (s, 2H), 2.80 (s, 3H), 1.35 (s, 3H). **^{13}C NMR (CDCl$_3$, 100 MHz):** δ 161.0 (q, $^3J_{CF}$ = 4.1 Hz), 151.7, 136.8, 136.7, 130.7, 130.5, 128.8, 128.1, 126.8, 125.1 (q, $^4J_{CF}$ = 2.9 Hz), 123.7 (q, $^1J_{CF}$ = 281.0 Hz), 122.9, 73.4 (m), 57.0, 56.9 (q, $^2J_{CF}$ = 29.6 Hz), 45.6, 27.8, 20.1. **^{19}F NMR (CDCl$_3$, 376 MHz):** δ -65.13 (d, $^3J_{FH}$ = 9.5 Hz). **IR (CH$_2$Cl$_2$, cm^{-1})** ν_{max}: 3064, 3029, 2926, 1780, 1455, 1347, 1256, 1174, 1080, 762, 707. MS (EI 70 eV, *m/z*, %) 345 (M^+, 31), 288 (32), 226 (60), 204 (100), 158 (66), 91 (40); **HRMS (EI$^+$)** *m/z*: $[M]^+$ Calcd for $C_{20}H_{18}NOF_3$ 345.1340; Found 345.1339.

4. Unsuccessful and low-yielding substrates

Scheme S1. Unsuccessful and low yielding substrates[a]

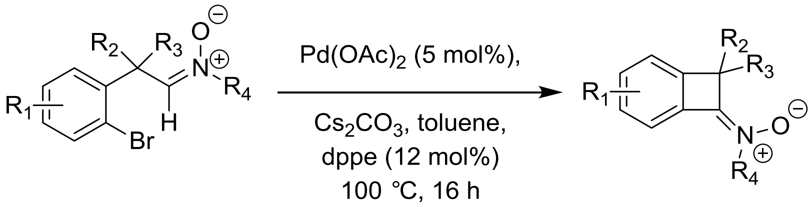

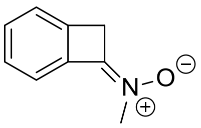

0%, decomposition

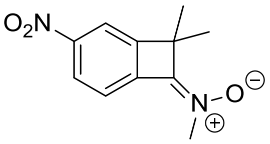

30%

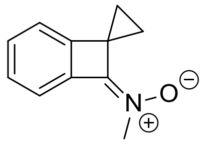

0%, only starting material

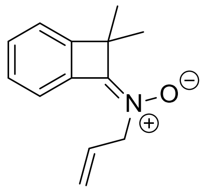

0%, decomposition

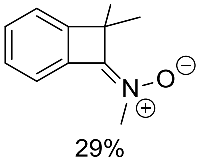

29%

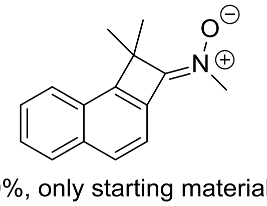

0%, only starting material

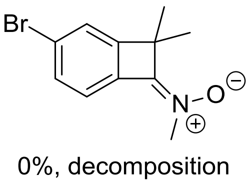

0%, decomposition

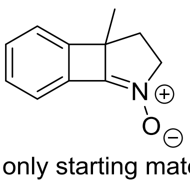

0%, only starting material

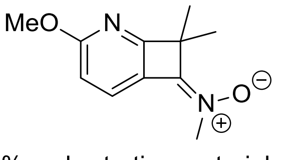

0%, only starting material

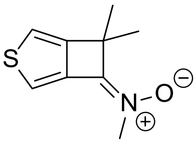

0%, only starting material

[a]*Reaction conditions: aldonitrone (0.5 mmol), Pd(OAc)$_2$ (5 mol%), dppe (12 mol%), Cs$_2$CO$_3$ (1 mmol), toluene (2.0 mL), 100 °C, 16 h, under an argon atmosphere.*

N-Phenyl aldonitrone similar to **1a** could not be prepared because condensation between 2-(2-bromophenyl)-2-methylpropanal and *N*-phenylhydroxylamine failed, probably due to sterical reasons (substrates were recovered); the same for *N*-*tert*-butyl aldonitrone.

5. Mechanistic considerations

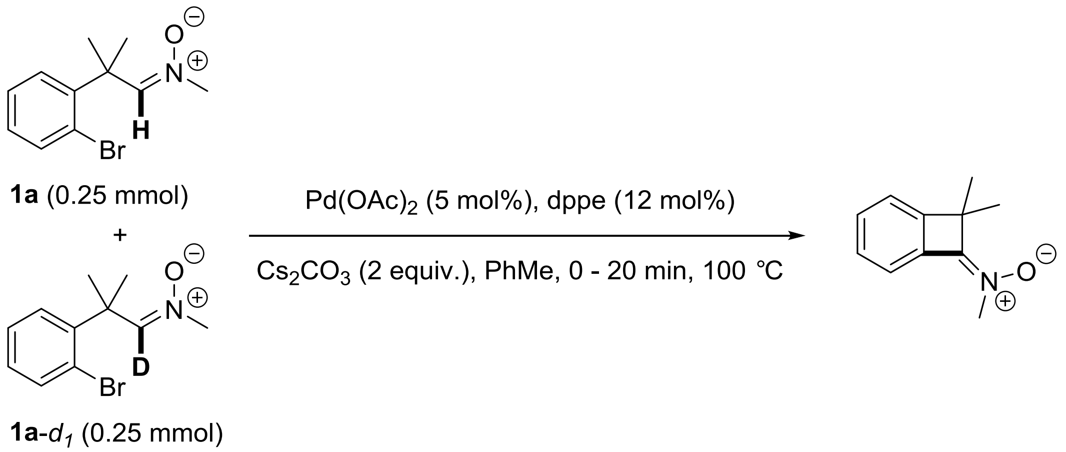

Under argon atmosphere, a flame-dried Schlenk flask was loaded with anhydrous PhMe (2 mL, 0.25 M), Cs_2CO_3 (326 mg, 1 mmol, 2 equiv.), dppe (23.9 mg, 0.06 mmol, 0.12 equiv.), Pd(OAc)$_2$ (5.6 mg, 0.025 mmol, 0.05 equiv.) and mixture of aldonitrone **1a** and aldonitrone **1a**-*d$_1$* in a 1:1 ratio (128 mg, 0.5 mmol, 1 equiv.). The mixture was stirred at 100 °C (oil bath) for the time indicated in **Table S2**. All the reactions were set up independently and the product yield was determined by ^{1}H NMR. The kinetic isotope effect calculated on the basis of initial rates was **k_H/k_D = 1.06**.

Table S2. KIE measurements

Entry	Time (min)	Total product concentration (%)	H-Nitrone concentration (%)	Conc. (M)	D-Nitrone concentration (%)	Conc. (M)
**1.**	0	0	0	0	0	0
**2.**	5	0,065	0,033	0,008	0,032	0,008
**3.**	10	0,100	0,053	0,013	0,047	0,012
**4.**	15	0,207	0,109	0,027	0,098	0,025
**5.**	20	0,260	0,131	0,033	0,129	0,032

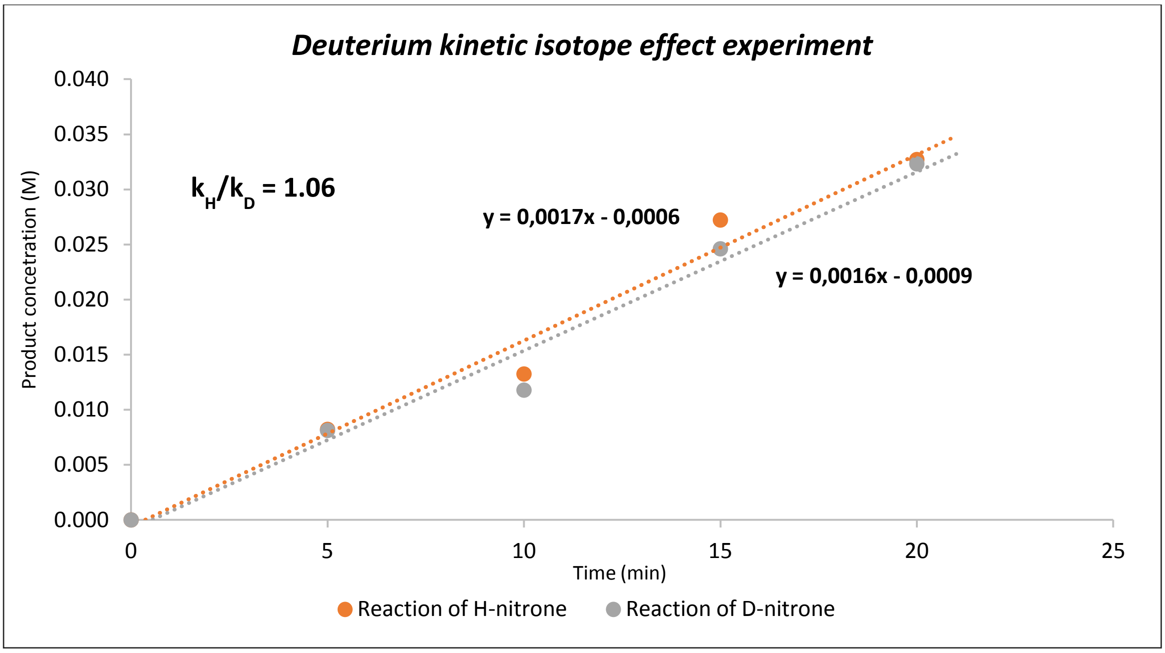
Deuterium kinetic isotope effect experiment
0.040
0.035
0.030
0.025
0.020
0.015
0.010
0.005
0.000
Product concetration (M)
kH/kD = 1.06
y = 0,0017x - 0,0006
y = 0,0016x - 0,0009
0
5
10
15
20
25
Time (min)
Reaction of H-nitrone
Reaction of D-nitrone

6. Crystallographic data

The crystalline sample of isoxazolidine **3** was prepared by dissolving in boiling EtOH and slow cooling to room temperature. The obtained white crystals of **3** were washed three times with EtOH and dried using an oil vacuum pump.

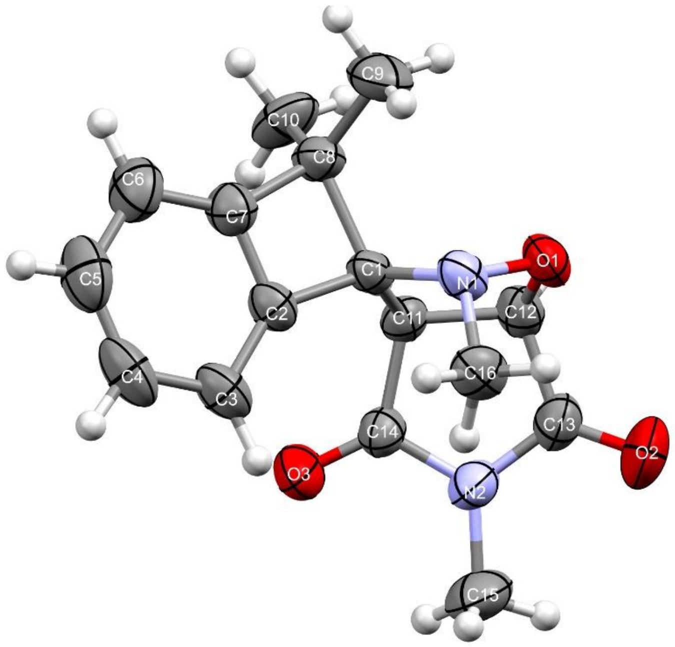

Fig. S1. Crystal structure of isoxazolidine 3 (CCDC 2164461) drawn with 50% displacement ellipsoid.

A total of 1148 frames were collected. The total exposure time was 38.27 hours. The frames were integrated with the Bruker SAINT software package using a narrow-frame algorithm. The integration of the data using an orthorhombic unit cell yielded a total of 13641 reflections to a maximum θ angle of 68.58° (0.83 Å resolution), of which 2711 were independent (average redundancy 5.032, completeness = 99.1%, R_{int} = 4.66%, R_{sig} = 3.98%) and 2560 (94.43%) were greater than $2\sigma(F^2)$. The final cell constants of a = 8.7040(10) Å, b = 9.1894(10) Å, c = 18.585(2) Å, volume = 1486.5(3) $Å^3$, are based upon the refinement of the XYZ-centroids of 8563 reflections above 20 σ(I) with 4.754° < 2θ < 135.9°. Data were corrected for absorption effects using the numerical method (SADABS). The ratio of minimum to maximum apparent transmission was 0.816. The calculated minimum and maximum transmission coefficients (based on crystal size) are 0.7200 and 0.7870.

The structure was solved and refined using the Bruker SHELXTL Software Package, using the space group P 21 21 21, with Z = 4 for the formula unit, $C_{16}H_{18}N_2O_3$. The final anisotropic full-matrix least-squares refinement on F^2 with 230 variables converged at R1 = 3.54%, for the observed data and wR2 = 9.99% for all data. The goodness-of-fit was 1.049. The largest peak in the final difference electron density synthesis was 0.119 $e^-/Å^3$ and the largest hole was -0.165 $e^-/Å^3$ with an RMS deviation of 0.035 $e^-/Å^3$. On the basis of the final model, the calculated density was 1.279 g/cm^3 and F(000), 608 e^-.

Table S3. Sample and crystal data for isoxazolidine 3 (CCDC 2164461)

**Identification code**	JB1101_A	
**Chemical formula**	$C_{16}H_{18}N_2O_3$	
**Formula weight**	286.32 g/mol	
**Temperature**	296(2) K	
**Wavelength**	1.54178 Å	
**Crystal size**	0.346 x 0.387 x 0.482 mm	
**Crystal habit**	colorless cubes	
**Crystal system**	orthorhombic	
**Space group**	P 21 21 21	
**Unit cell dimensions**	a = 8.7040(10) Å	α = 90°
	b = 9.1894(10) Å	β = 90°
	c = 18.585(2) Å	γ = 90°
**Volume**	1486.5(3) $Å^3$	
**Z**	4	
**Density (calculated)**	1.279 g/cm^3	
**Absorption coefficient**	0.729 mm^{-1}	
**F(000)**	608	

Table S4. Data collection and structure refinement for isoxazolidine 3 (CCDC 2164461)

**Theta range for data collection**	4.76 to 68.58°	
**Index ranges**	-9<=h<=10, -11<=k<=10, -22<=l<=22	
**Reflections collected**	13641	
**Independent reflections**	2711 [R(int) = 0.0466]	
**Coverage of independent reflections**	99.1%	
**Absorption correction**	numerical	
**Max. and min. transmission**	0.7870 and 0.7200	
**Structure solution technique**	direct methods	
**Structure solution program**	SHELXL-2014 (Sheldrick, 2014)	
**Refinement method**	Full-matrix least-squares on F^2	
**Refinement program**	SHELXL-2014 (Sheldrick, 2014)	
**Function minimized**	$\Sigma\ w(F_o^2 - F_c^2)^2$	
**Data / restraints / parameters**	2711 / 0 / 230	
**Goodness-of-fit on F^2**	1.049	
**Final R indices**	2560 data; I>2σ(I)	R1 = 0.0354, wR2 = 0.0978
	all data	R1 = 0.0372, wR2 = 0.0999
**Weighting scheme**	$w=1/[\sigma^2(F_o^2)+(0.0598P)^2+0.1326P]$ where $P=(F_o^2+2F_c^2)/3$	
**Absolute structure parameter**	-0.1(3)	
**Largest diff. peak and hole**	0.119 and -0.165 $eÅ^{-3}$	
**R.M.S. deviation from mean**	0.035 $eÅ^{-3}$	

Table S5. Atomic coordinates and equivalent isotropic atomic displacement parameters (Å^2) for isoxazolidine 3 (CCDC 2164461)

U(eq) is defined as one third of the trace of the orthogonalized U_{ij} tensor.

	x/a	y/b	z/c	U(eq)
O1	0.74003(19)	0.72554(16)	0.13581(9)	0.0519(4)
O2	0.9210(3)	0.6705(2)	0.27277(15)	0.0844(7)
O3	0.6418(2)	0.0834(2)	0.29594(9)	0.0641(5)
N1	0.7508(2)	0.8704(2)	0.10548(10)	0.0455(4)
N2	0.7955(2)	0.8827(2)	0.29968(10)	0.0522(5)
C1	0.6189(2)	0.9479(2)	0.13660(10)	0.0380(4)
C2	0.6220(3)	0.1113(2)	0.12401(11)	0.0431(4)
C3	0.6986(3)	0.2380(3)	0.14263(14)	0.0559(6)
C4	0.6442(4)	0.3638(3)	0.10995(17)	0.0694(8)
C5	0.5218(4)	0.3617(3)	0.06225(18)	0.0749(8)
C6	0.4454(4)	0.2343(3)	0.04467(16)	0.0678(7)
C7	0.5008(3)	0.1086(3)	0.07669(12)	0.0497(5)
C8	0.4705(3)	0.9464(3)	0.08417(12)	0.0472(5)
C9	0.4914(4)	0.8552(3)	0.01611(15)	0.0714(8)
C10	0.3174(3)	0.9144(3)	0.11992(17)	0.0680(7)
C11	0.5995(2)	0.8824(2)	0.21270(11)	0.0398(4)
C12	0.6863(2)	0.7400(2)	0.20818(13)	0.0471(5)
C13	0.8157(3)	0.7539(3)	0.26271(13)	0.0539(5)
C14	0.6760(2)	0.9650(2)	0.27292(11)	0.0450(5)
C15	0.8960(4)	0.9339(4)	0.35682(18)	0.0804(9)
C16	0.9008(3)	0.9306(3)	0.12316(15)	0.0557(6)

Table S6. Bond lengths (Å) for isoxazolidine 3 (CCDC 2164461)

O1-C12	1.430(3)	O1-N1	1.449(2)
O2-C13	1.210(3)	O3-C14	1.207(3)
N1-C16	1.455(3)	N1-C1	1.469(3)
N2-C13	1.380(3)	N2-C14	1.378(3)
N2-C15	1.454(3)	C1-C2	1.520(3)
C1-C11	1.546(3)	C1-C8	1.619(3)
C2-C7	1.374(3)	C2-C3	1.385(3)
C3-C4	1.389(4)	C3-H3	1.01(3)
C4-C5	1.386(5)	C4-H4	0.91(4)
C5-C6	1.385(5)	C5-H5	0.91(4)
C6-C7	1.386(4)	C6-H6	0.97(4)
C7-C8	1.520(3)	C8-C10	1.517(3)
C8-C9	1.528(4)	C9-H9A	0.96

C9-H9B	0.96	C9-H9C	0.96
C10-H10A	0.96	C10-H10B	0.96
C10-H10C	0.96	C11-C14	1.508(3)
C11-C12	1.513(3)	C11-H11	0.97(3)
C12-C13	1.520(3)	C12-H12	0.96(3)
C15-H15A	0.96	C15-H15B	0.96
C15-H15C	0.96	C16-H16C	1.01(4)
C16-H16A	0.96(4)	C16-H16B	1.04(3)

Table S7. Bond angles (°) for isoxazolidine 3 (CCDC 2164461)

C12-O1-N1	107.55(14)	O1-N1-C16	108.65(19)
O1-N1-C1	103.96(15)	C16-N1-C1	115.32(17)
C13-N2-C14	112.78(19)	C13-N2-C15	124.4(2)
C14-N2-C15	122.7(2)	N1-C1-C2	113.84(16)
N1-C1-C11	104.90(17)	C2-C1-C11	121.82(17)
N1-C1-C8	112.50(16)	C2-C1-C8	85.98(16)
C11-C1-C8	117.37(16)	C7-C2-C3	123.0(2)
C7-C2-C1	93.83(17)	C3-C2-C1	143.1(2)
C2-C3-C4	115.2(3)	C2-C3-H3	121.4(19)
C4-C3-H3	123.4(19)	C5-C4-C3	122.0(3)
C5-C4-H4	119.(3)	C3-C4-H4	119.(3)
C6-C5-C4	122.1(3)	C6-C5-H5	118.(3)
C4-C5-H5	120.(2)	C5-C6-C7	115.9(3)
C5-C6-H6	120.(2)	C7-C6-H6	124.(2)
C2-C7-C6	121.8(2)	C2-C7-C8	95.34(18)
C6-C7-C8	142.8(2)	C10-C8-C7	112.5(2)
C10-C8-C9	111.2(2)	C7-C8-C9	116.2(2)
C10-C8-C1	116.04(18)	C7-C8-C1	84.72(16)
C9-C8-C1	114.1(2)	C8-C9-H9A	109.5
C8-C9-H9B	109.5	H9A-C9-H9B	109.5
C8-C9-H9C	109.5	H9A-C9-H9C	109.5
H9B-C9-H9C	109.5	C8-C10-H10A	109.5
C8-C10-H10B	109.5	H10A-C10-H10B	109.5
C8-C10-H10C	109.5	H10A-C10-H10C	109.5
H10B-C10-H10C	109.5	C14-C11-C12	104.81(17)
C14-C11-C1	115.76(17)	C12-C11-C1	103.35(17)
C14-C11-H11	109.8(14)	C12-C11-H11	110.6(15)
C1-C11-H11	112.0(14)	O1-C12-C11	107.21(17)
O1-C12-C13	113.13(18)	C11-C12-C13	105.11(18)
O1-C12-H12	102.2(16)	C11-C12-H12	117.4(17)
C13-C12-H12	112.0(16)	O2-C13-N2	124.3(2)

O2-C13-C12	127.7(2)	N2-C13-C12	108.01(18)
O3-C14-N2	123.6(2)	O3-C14-C11	127.4(2)
N2-C14-C11	108.97(19)	N2-C15-H15A	109.5
N2-C15-H15B	109.5	H15A-C15-H15B	109.5
N2-C15-H15C	109.5	H15A-C15-H15C	109.5
H15B-C15-H15C	109.5	N1-C16-H16C	109.(2)
N1-C16-H16A	107.(2)	H16C-C16-H16A	106.(3)
N1-C16-H16B	114.7(18)	H16C-C16-H16B	103.(3)
H16A-C16-H16B	117.(3)		

Table S8. Torsion angles (°) for isoxazolidine 3 (CCDC 2164461)

C12-O1-N1-C16	86.75(19)	C12-O1-N1-C1	-36.54(19)
O1-N1-C1-C2	169.00(16)	C16-N1-C1-C2	50.2(3)
O1-N1-C1-C11	33.43(18)	C16-N1-C1-C11	-85.4(2)
O1-N1-C1-C8	-95.26(19)	C16-N1-C1-C8	145.9(2)
N1-C1-C2-C7	110.00(19)	C11-C1-C2-C7	-122.77(19)
C8-C1-C2-C7	-2.85(17)	N1-C1-C2-C3	-69.3(4)
C11-C1-C2-C3	57.9(4)	C8-C1-C2-C3	177.9(3)
C7-C2-C3-C4	0.0(3)	C1-C2-C3-C4	179.2(3)
C2-C3-C4-C5	0.1(4)	C3-C4-C5-C6	0.5(5)
C4-C5-C6-C7	-1.2(5)	C3-C2-C7-C6	-0.7(4)
C1-C2-C7-C6	179.8(2)	C3-C2-C7-C8	-177.5(2)
C1-C2-C7-C8	3.04(18)	C5-C6-C7-C2	1.3(4)
C5-C6-C7-C8	175.9(3)	C2-C7-C8-C10	113.2(2)
C6-C7-C8-C10	-62.2(4)	C2-C7-C8-C9	-117.1(2)
C6-C7-C8-C9	67.5(4)	C2-C7-C8-C1	-2.86(17)
C6-C7-C8-C1	-178.3(4)	N1-C1-C8-C10	135.9(2)
C2-C1-C8-C10	-109.9(2)	C11-C1-C8-C10	14.1(3)
N1-C1-C8-C7	-111.59(19)	C2-C1-C8-C7	2.58(15)
C11-C1-C8-C7	126.55(18)	N1-C1-C8-C9	4.8(3)
C2-C1-C8-C9	118.9(2)	C11-C1-C8-C9	-117.1(2)
N1-C1-C11-C14	95.21(19)	C2-C1-C11-C14	-35.9(3)
C8-C1-C11-C14	-139.08(18)	N1-C1-C11-C12	-18.75(19)
C2-C1-C11-C12	-149.85(19)	C8-C1-C11-C12	106.96(19)
N1-O1-C12-C11	24.4(2)	N1-O1-C12-C13	-91.0(2)
C14-C11-C12-O1	-124.65(18)	C1-C11-C12-O1	-3.0(2)
C14-C11-C12-C13	-4.0(2)	C1-C11-C12-C13	117.63(18)
C14-N2-C13-O2	173.6(3)	C15-N2-C13-O2	-1.5(4)
C14-N2-C13-C12	-5.4(3)	C15-N2-C13-C12	179.6(3)
O1-C12-C13-O2	-56.5(3)	C11-C12-C13-O2	-173.2(3)
O1-C12-C13-N2	122.4(2)	C11-C12-C13-N2	5.7(2)

C13-N2-C14-O3	-177.6(2)	C15-N2-C14-O3	-2.5(4)
C13-N2-C14-C11	2.7(3)	C15-N2-C14-C11	177.9(2)
C12-C11-C14-O3	-178.5(2)	C1-C11-C14-O3	68.3(3)
C12-C11-C14-N2	1.1(2)	C1-C11-C14-N2	-112.0(2)

Table S9. Anisotropic atomic displacement parameters (Å^2) for isoxazolidine 3 (CCDC 2164461)

The anisotropic atomic displacement factor exponent takes the form: $-2\pi^2[h^2 a^{*2} U_{11} + ... + 2 h k a^* b^* U_{12}]$

	U_{11}	U_{22}	U_{33}	U_{23}	U_{13}	U_{12}
O1	0.0530(8)	0.0375(7)	0.0652(9)	-0.0137(7)	0.0038(7)	-0.0004(7)
O2	0.0777(14)	0.0679(13)	0.1076(17)	0.0067(12)	-0.0204(12)	0.0333(11)
O3	0.0741(12)	0.0605(11)	0.0578(9)	-0.0182(8)	-0.0085(8)	0.0201(9)
N1	0.0412(8)	0.0439(9)	0.0515(9)	-0.0091(7)	0.0073(7)	-0.0044(8)
N2	0.0476(10)	0.0596(11)	0.0493(9)	-0.0014(9)	-0.0100(8)	0.0095(9)
C1	0.0342(9)	0.0368(10)	0.0430(9)	-0.0056(8)	0.0006(8)	-0.0050(8)
C2	0.0454(10)	0.0369(10)	0.0469(10)	-0.0017(8)	0.0048(8)	-0.0058(8)
C3	0.0633(14)	0.0419(12)	0.0626(13)	-0.0080(10)	0.0094(12)	-0.0131(11)
C4	0.096(2)	0.0360(12)	0.0757(16)	-0.0063(11)	0.0225(16)	-0.0123(13)
C5	0.103(2)	0.0466(15)	0.0756(17)	0.0138(13)	0.0098(17)	0.0127(16)
C6	0.0818(18)	0.0575(16)	0.0641(14)	0.0117(13)	-0.0067(14)	0.0107(14)
C7	0.0536(12)	0.0449(12)	0.0507(11)	0.0022(9)	-0.0019(9)	-0.0014(10)
C8	0.0464(11)	0.0442(12)	0.0510(11)	-0.0001(9)	-0.0099(9)	-0.0076(9)
C9	0.091(2)	0.0658(17)	0.0569(13)	-0.0140(12)	-0.0235(14)	-0.0068(16)
C10	0.0383(11)	0.0823(19)	0.0833(17)	0.0181(14)	-0.0159(11)	-0.0126(12)
C11	0.0302(9)	0.0428(10)	0.0464(10)	-0.0003(9)	0.0013(7)	-0.0005(8)
C12	0.0444(10)	0.0348(10)	0.0620(12)	0.0009(9)	0.0015(9)	-0.0041(9)
C13	0.0489(11)	0.0475(13)	0.0653(13)	0.0080(10)	-0.0043(10)	0.0073(11)
C14	0.0427(10)	0.0489(12)	0.0434(10)	-0.0018(9)	0.0001(8)	0.0080(10)
C15	0.0709(17)	0.101(2)	0.0691(16)	-0.0090(16)	-0.0298(14)	0.0108(16)
C16	0.0374(11)	0.0609(15)	0.0687(15)	-0.0075(12)	0.0097(10)	-0.0074(10)

Table S10. Hydrogen atomic coordinates and isotropic atomic displacement parameters (Å^2) for isoxazolidine 3 (CCDC 2164461)

	x/a	y/b	z/c	U(eq)
H9A	0.4119	0.8787	-0.0177	0.107
H9B	0.4856	0.7537	0.0282	0.107
H9C	0.5898	0.8759	-0.0048	0.107
H10A	0.3092	0.9695	0.1636	0.102
H10B	0.3109	0.8124	0.1308	0.102
H10C	0.2354	0.9409	0.0880	0.102
H15A	0.9740	0.9957	0.3368	0.121

H15B	0.9434	0.8521	0.3800	0.121
H15C	0.8369	0.9878	0.3914	0.121
H3	0.790(4)	1.236(4)	0.1755(17)	0.066(8)
H11	0.492(3)	0.865(3)	0.2245(13)	0.042(6)
H4	0.687(5)	1.451(5)	0.122(2)	0.095(12)
H16C	0.915(4)	1.025(5)	0.096(2)	0.084(10)
H16A	0.977(4)	0.864(4)	0.1048(19)	0.077(9)
H16B	0.913(4)	0.963(3)	0.1766(18)	0.065(8)
H5	0.484(5)	1.447(4)	0.045(2)	0.083(10)
H12	0.629(3)	0.652(3)	0.2147(14)	0.051(7)
H6	0.355(4)	1.237(4)	0.014(2)	0.077(9)

7. References

1. Álvarez-Bercedo, P.; Flores-Gaspar, A.; Correa, A.; Martin, R. *J. Am. Chem. Soc.* **2010**, *132*, 466–467.
2. Hsieh, J.-C.; Cheng, A.-Y.; Fu, J.-H.; Kang, T.-W. *Org. Biomol. Chem.* **2012**, *10*, 6404–6409.
3. Li, S.; Eleya, N.; Staubitz, A. *Org. Lett.* **2020**, *22*, 1624–1627.
4. Hisano, N.; Kamei, Y.; Kansaku, Y.; Yamanaka, M.; Mori, K. *Org. Lett.* **2018**, *20*, 4223–4226.
5. Chaumontet, M.; Piccardi, R.; Audic, N.; Hitce, J.; Peglion, J.-L.; Clot, E.; Baudoin, O. *J. Am. Chem. Soc.* **2008**, *130*, 15157–15166.
6. Zhang, W.; Tao, S.; Ge, H.; Li, Q.; Ai, Z.; Li, X.; Zhang, B.; Sun, F.; Xu, X.; Du, Y. *Org. Lett.* **2020**, *22*, 448–452.
7. Xin, H.; Xin, M.; Feng, R.; Zehong, W. Patent WO2018205948. **2018**, A1.
8. Guin, J.; Varseev, G.; List, B. *J. Am. Chem. Soc.* **2013**, *135*, 2100–2103.
9. Culkin, D. A.; Hartwig, J. F. *J. Am. Chem. Soc.* **2002**, *124*, 9330–9331.
10. Kesharwani, T.; Verma, A. K.; Emrich, D.; Ward, J. A.; Larock, R. C. *Org. Lett.* **2009**, *11*, 2591–2593.
11. Fujita, T.; Takahashi, I.; Hayashi, M.; Wang, J.; Fuchibe, K.; Ichikawa, J. *European J. Org. Chem.* **2017**, *2017*, 262–265.
12. Son, J.; Kim, K. H.; Mo, D.-L.; Wink, D. J.; Anderson, L. L. *Angew. Chemie Int. Ed.* **2017**, *56*, 3059–3063.
13. Jakowiecki, J.; Loska, R.; Makosza, M. *J. Org. Chem.* **2008**, *73*, 5436–5441.

8. NMR Spectra

S1c

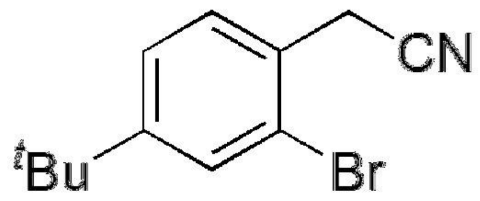

^{1}H NMR ($CDCl_3$, 400 MHz)

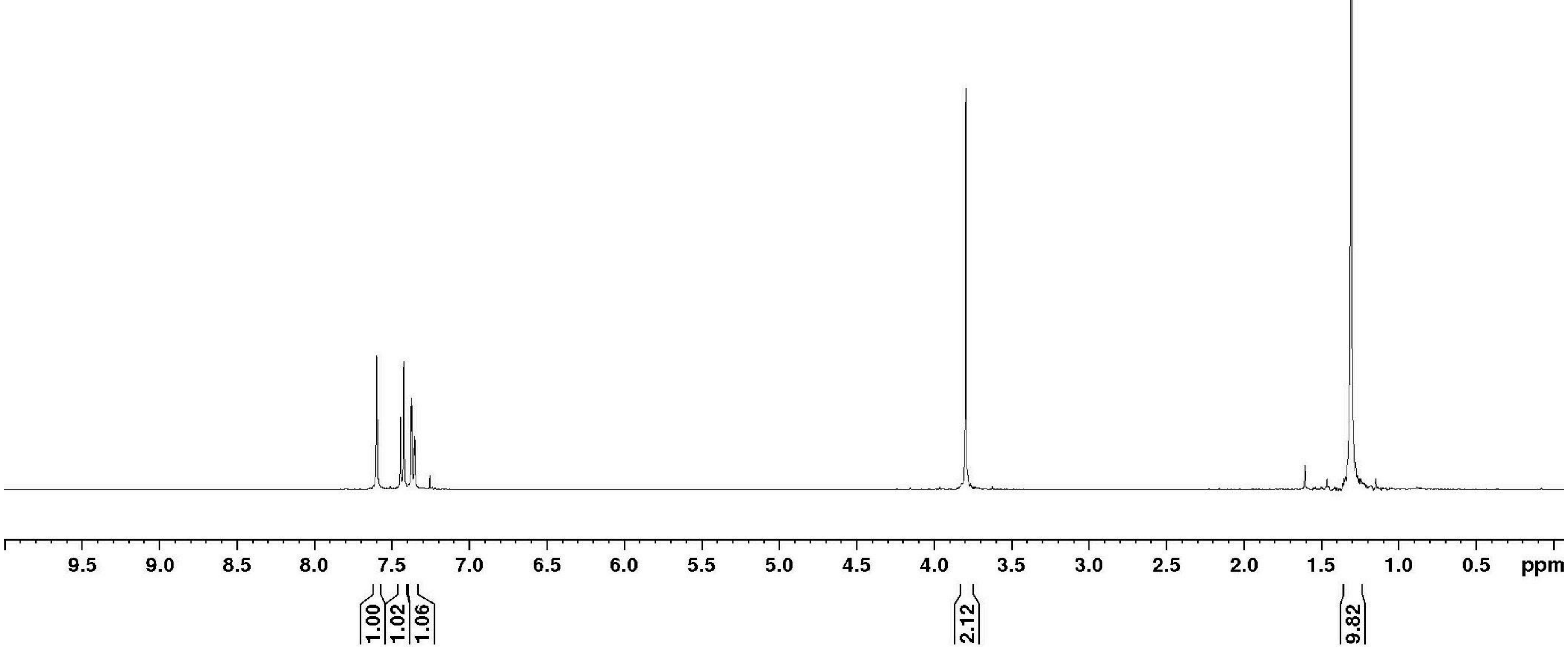

S1c

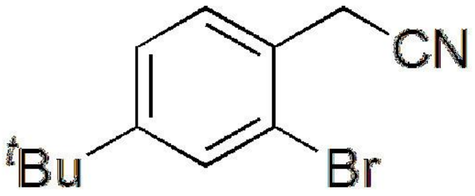

^{13}C NMR ($CDCl_3$, 100 MHz)

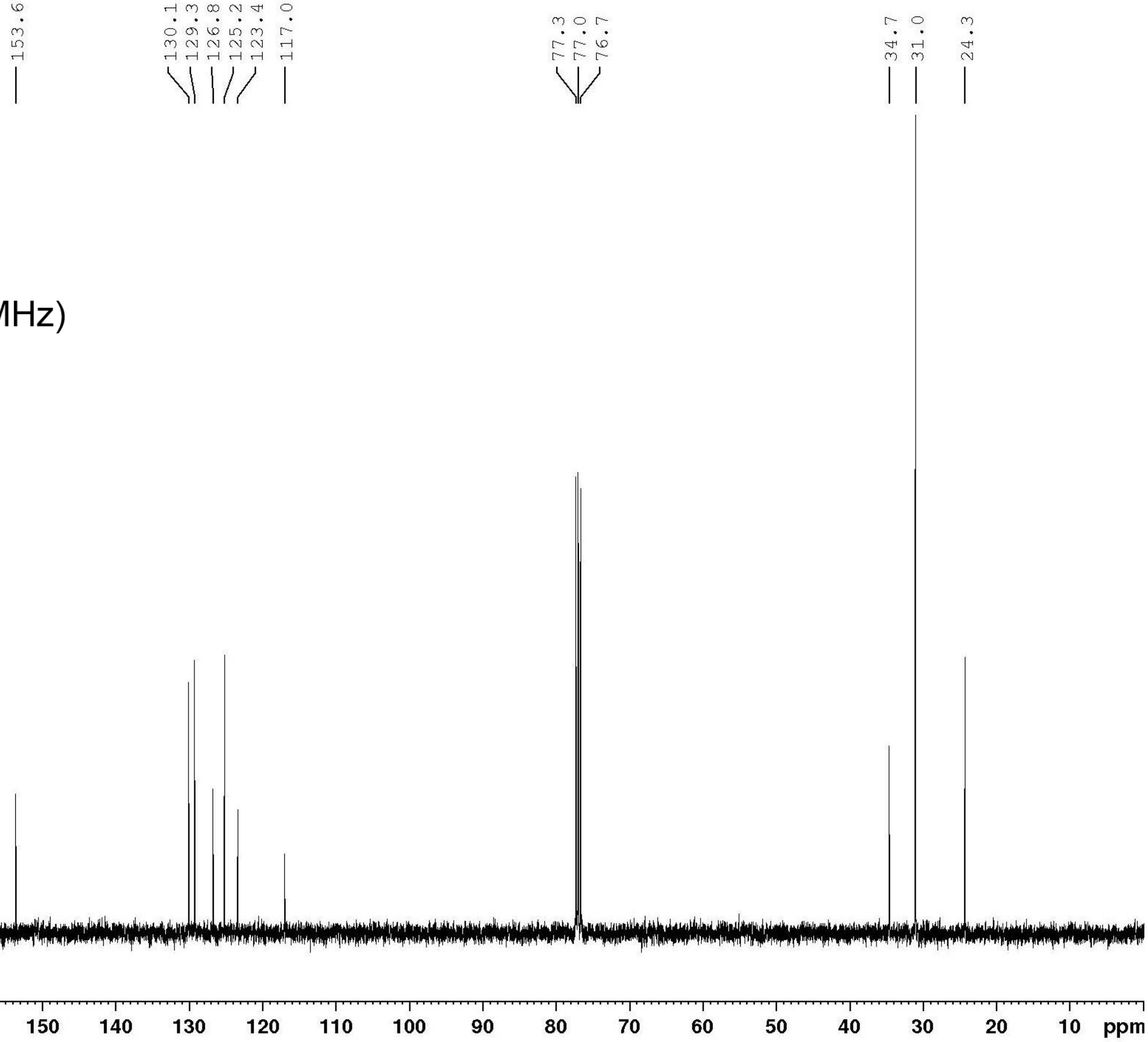

S1v

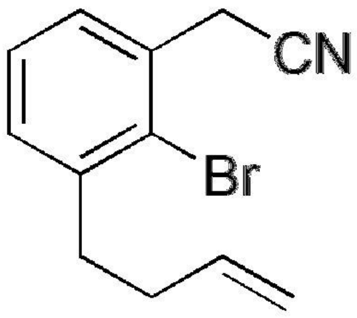

^{1}H NMR ($CDCl_3$, 400 MHz)

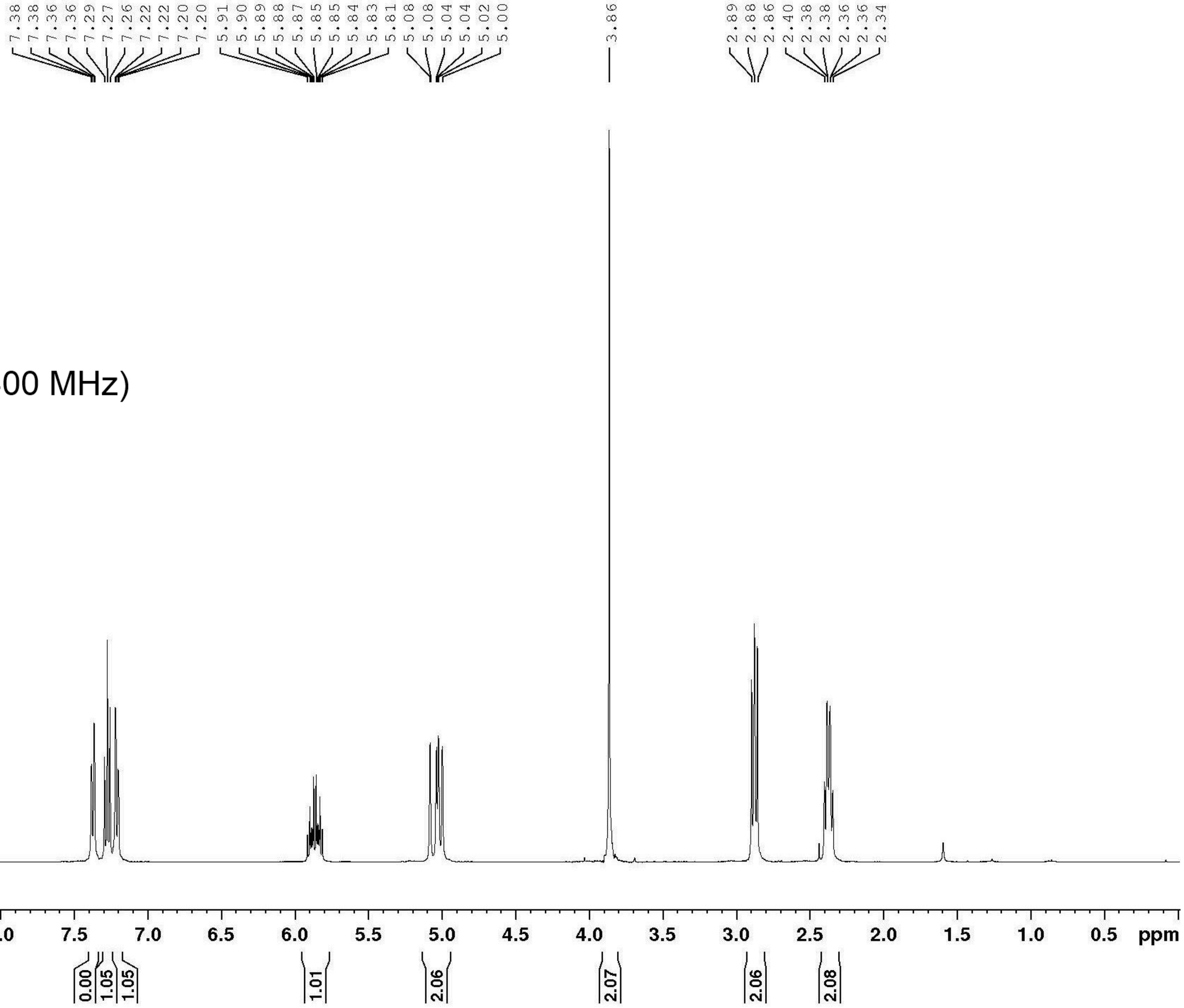

S1v

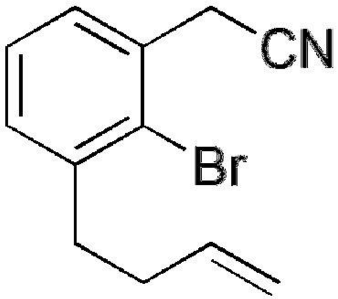

^{13}C NMR ($CDCl_3$, 100 MHz)

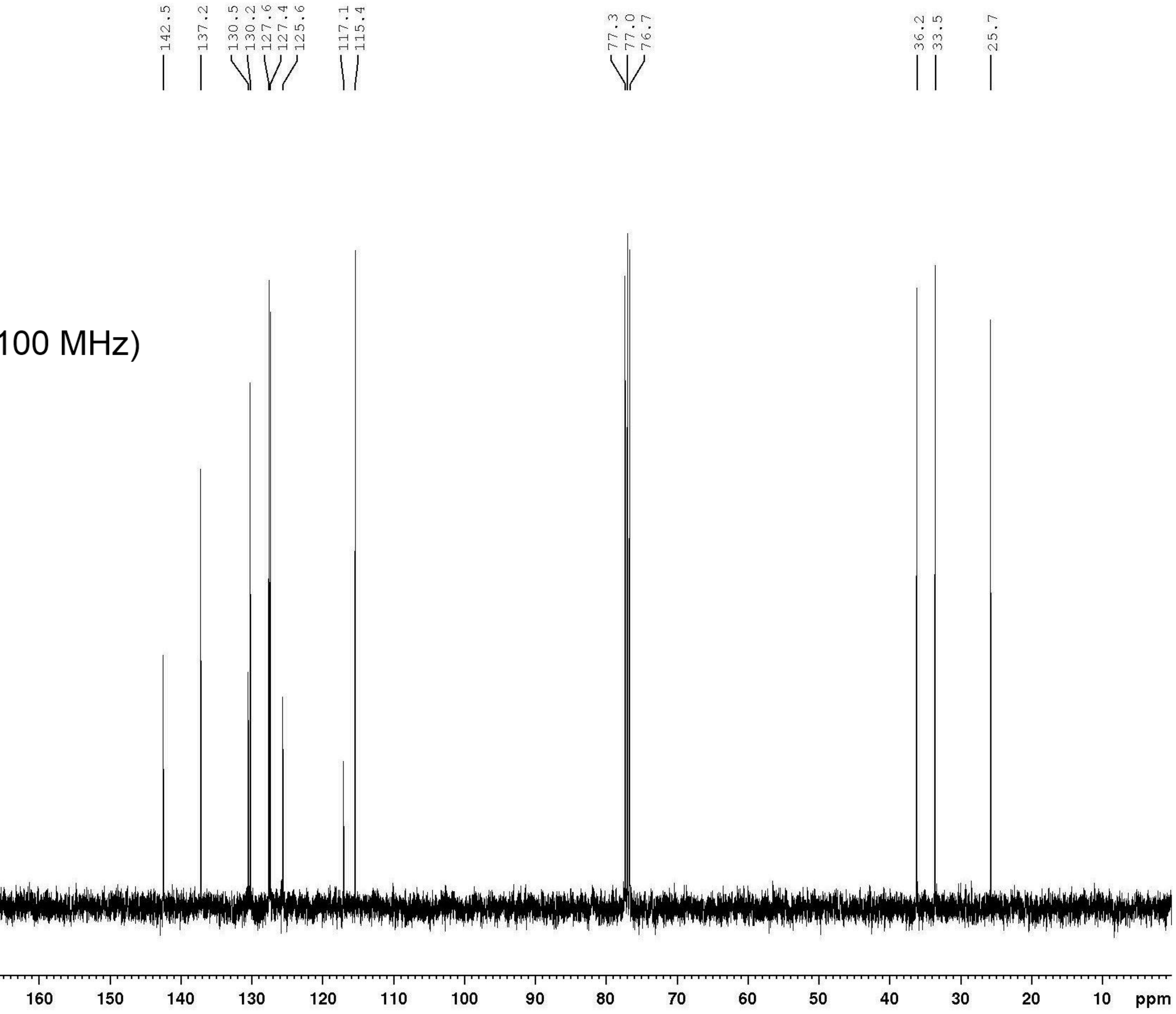

S2b

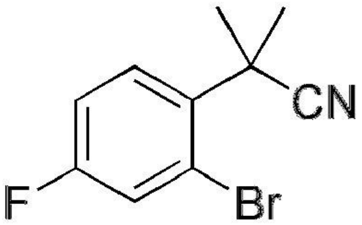

^{1}H NMR ($CDCl_3$, 400 MHz)

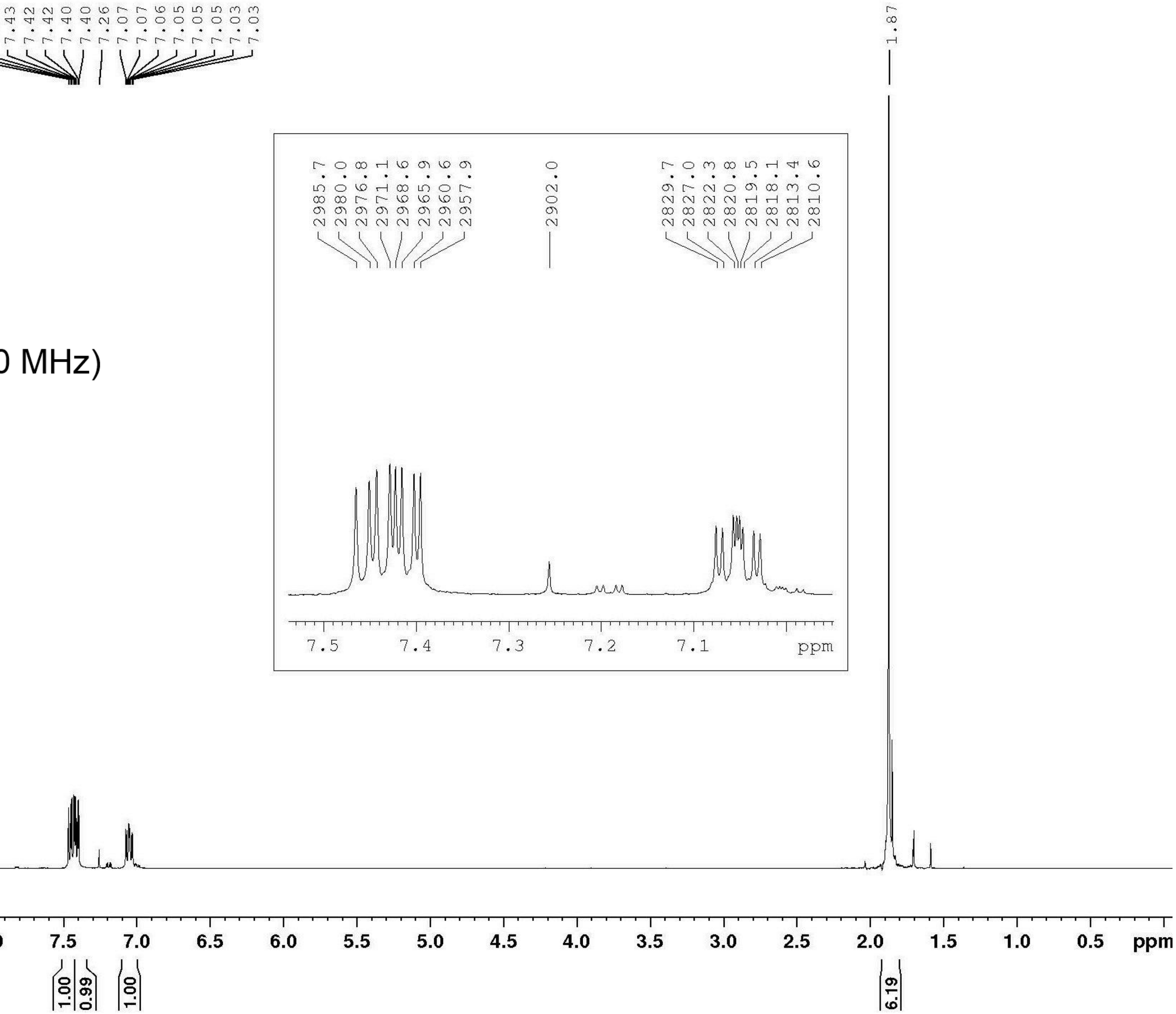

S2b

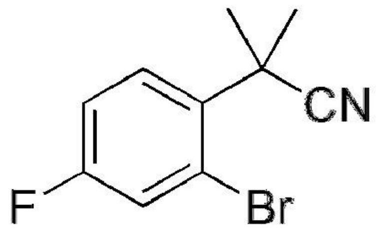

^{13}C NMR ($CDCl_3$, 100 MHz)

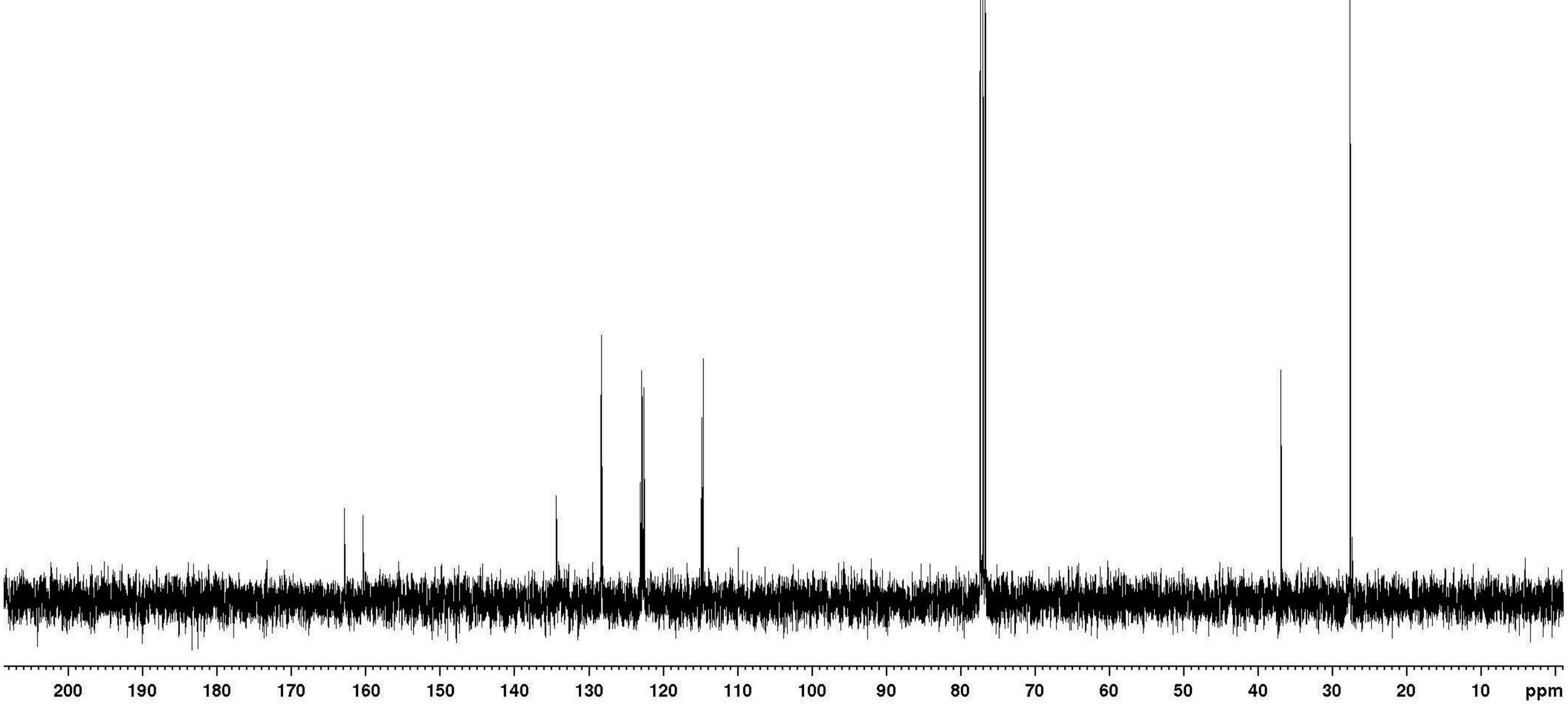

S2b

^{19}F NMR ($CDCl_3$, 376 MHz)

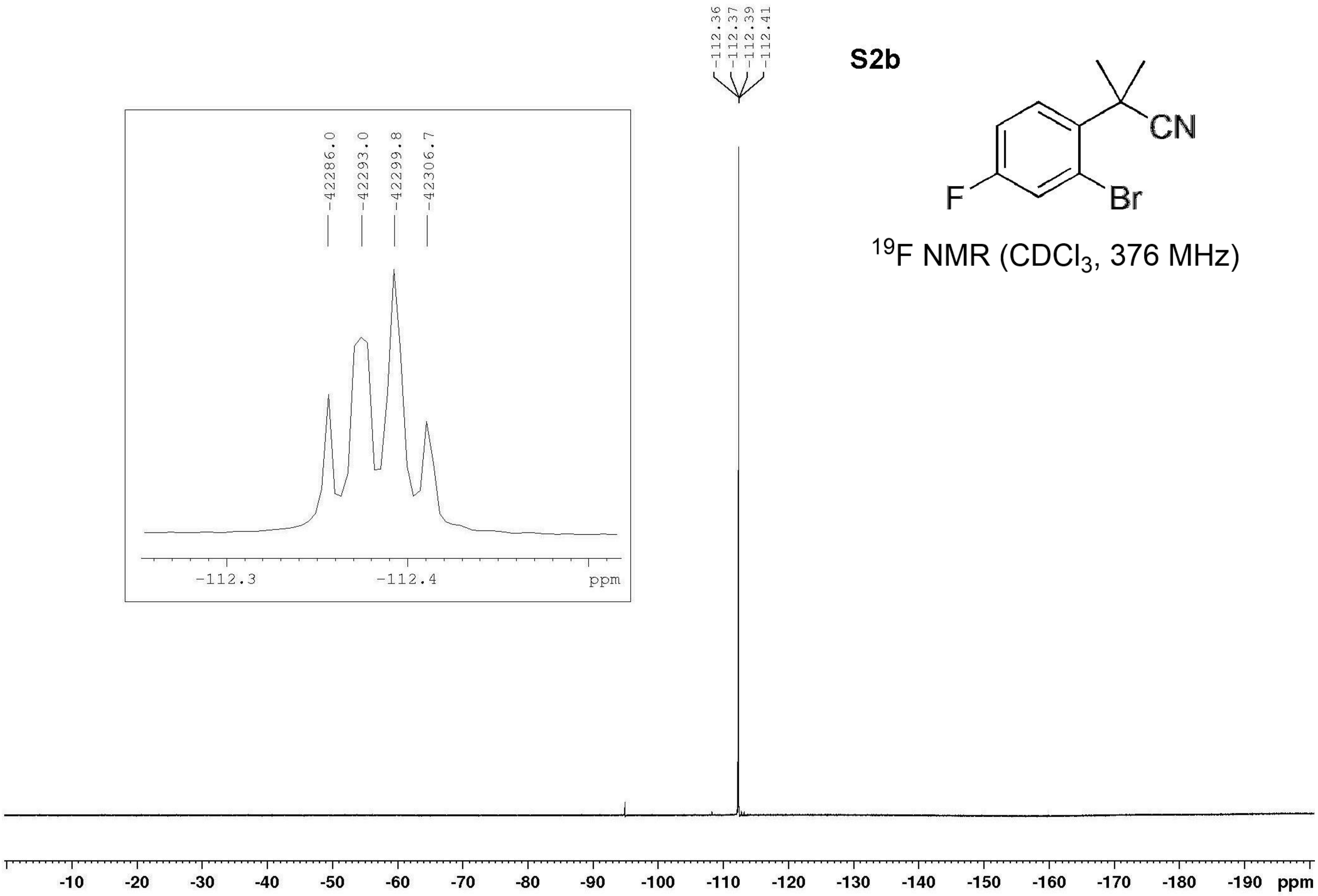

S2c

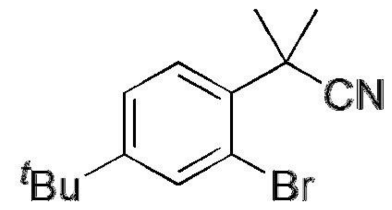

^{1}H NMR ($CDCl_3$, 400 MHz)

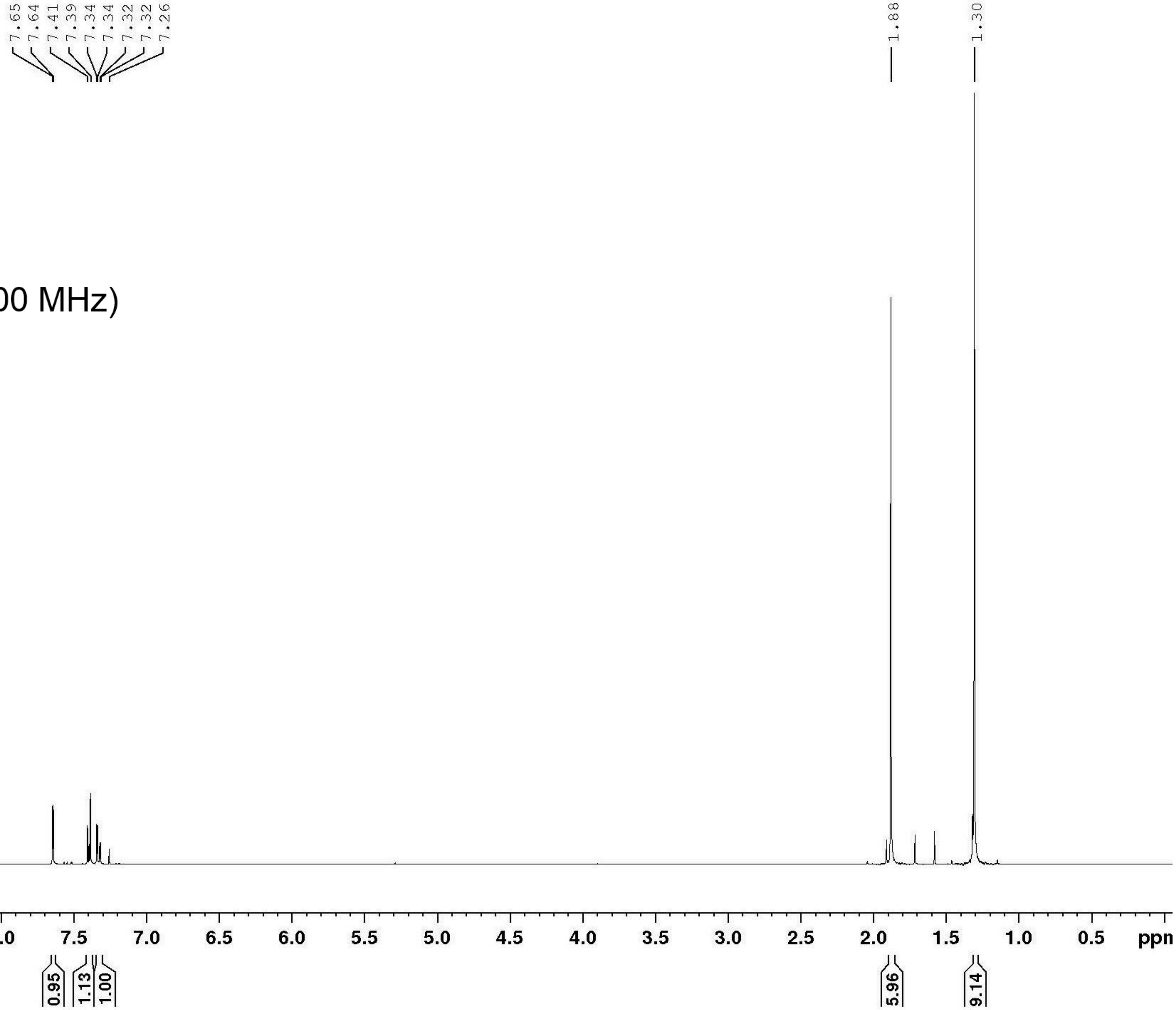

S2c

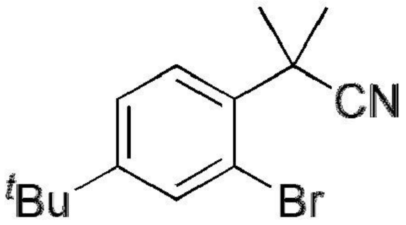

^{13}C NMR ($CDCl_3$, 100 MHz)

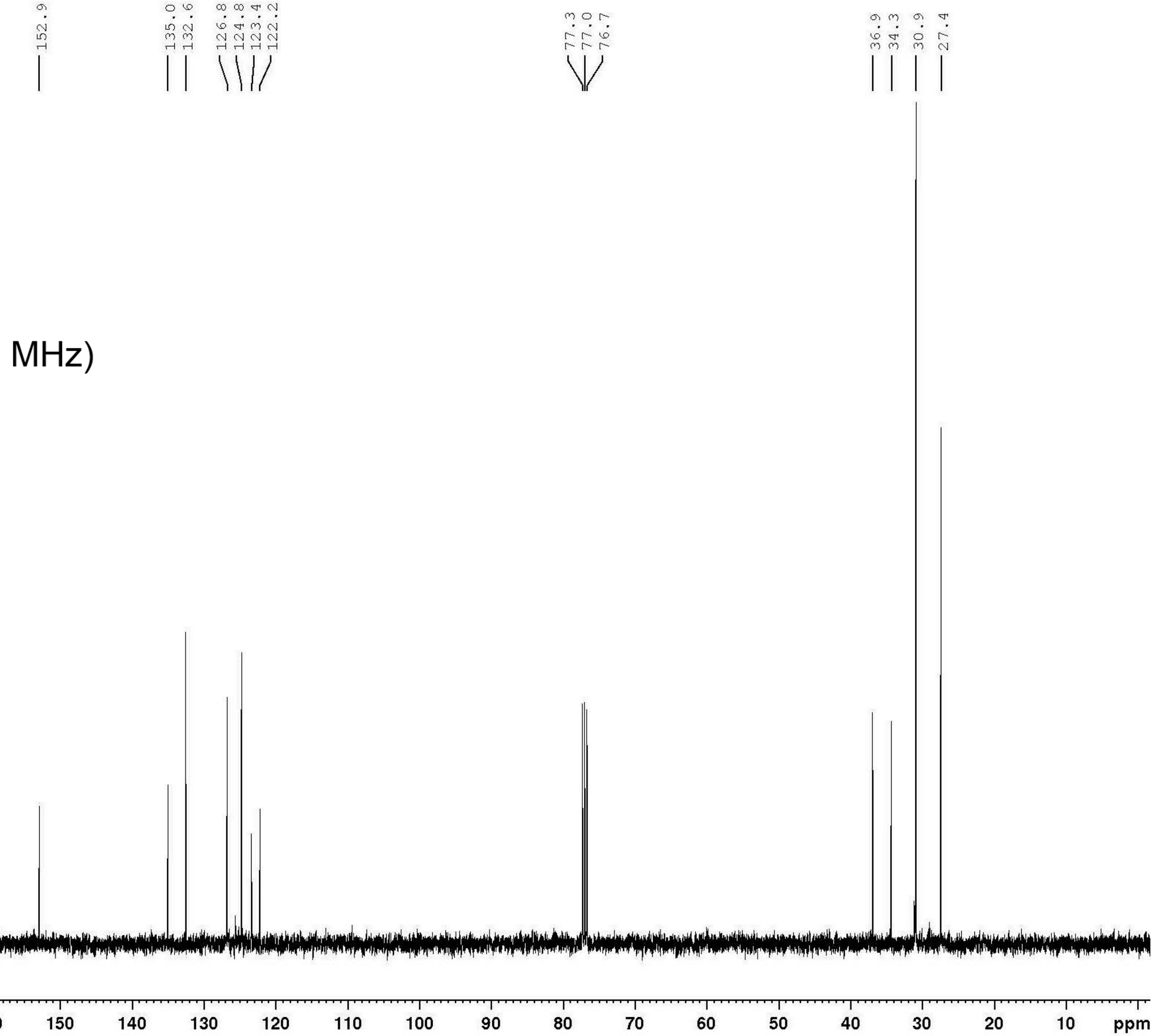

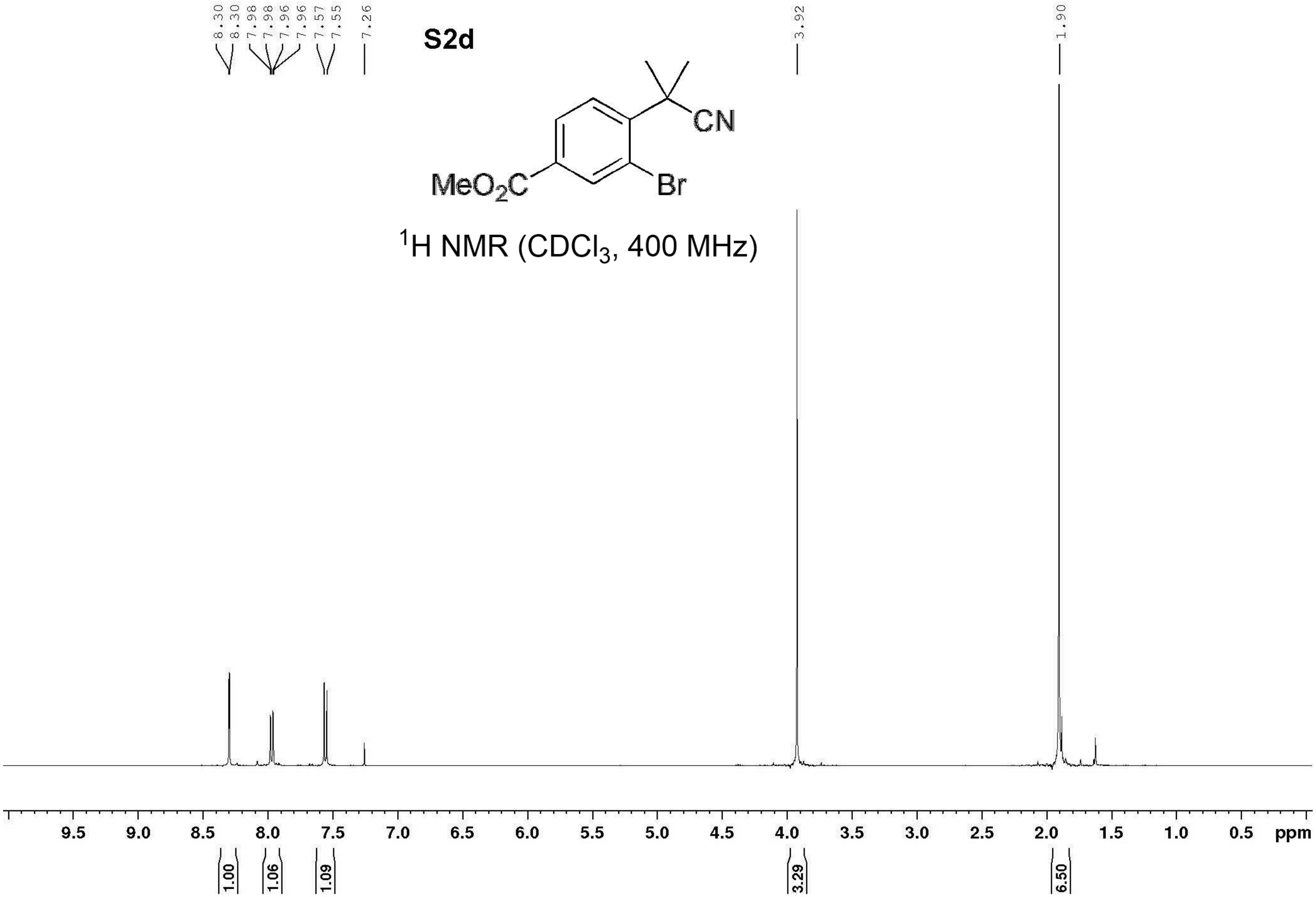
S2d
MeO2C
Br
CN
1H NMR (CDCl3, 400 MHz)
8.30
8.30
7.98
7.98
7.96
7.96
7.57
7.55
7.26
3.92
1.90
9.5
9.0
8.5
8.0
7.5
7.0
6.5
6.0
5.5
5.0
4.5
4.0
3.5
3.0
2.5
2.0
1.5
1.0
0.5
ppm
1.00
1.06
1.09
3.29
6.50

S2d

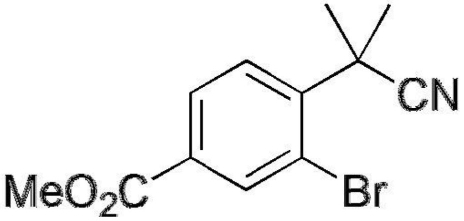

^{13}C NMR ($CDCl_3$, 100 MHz)

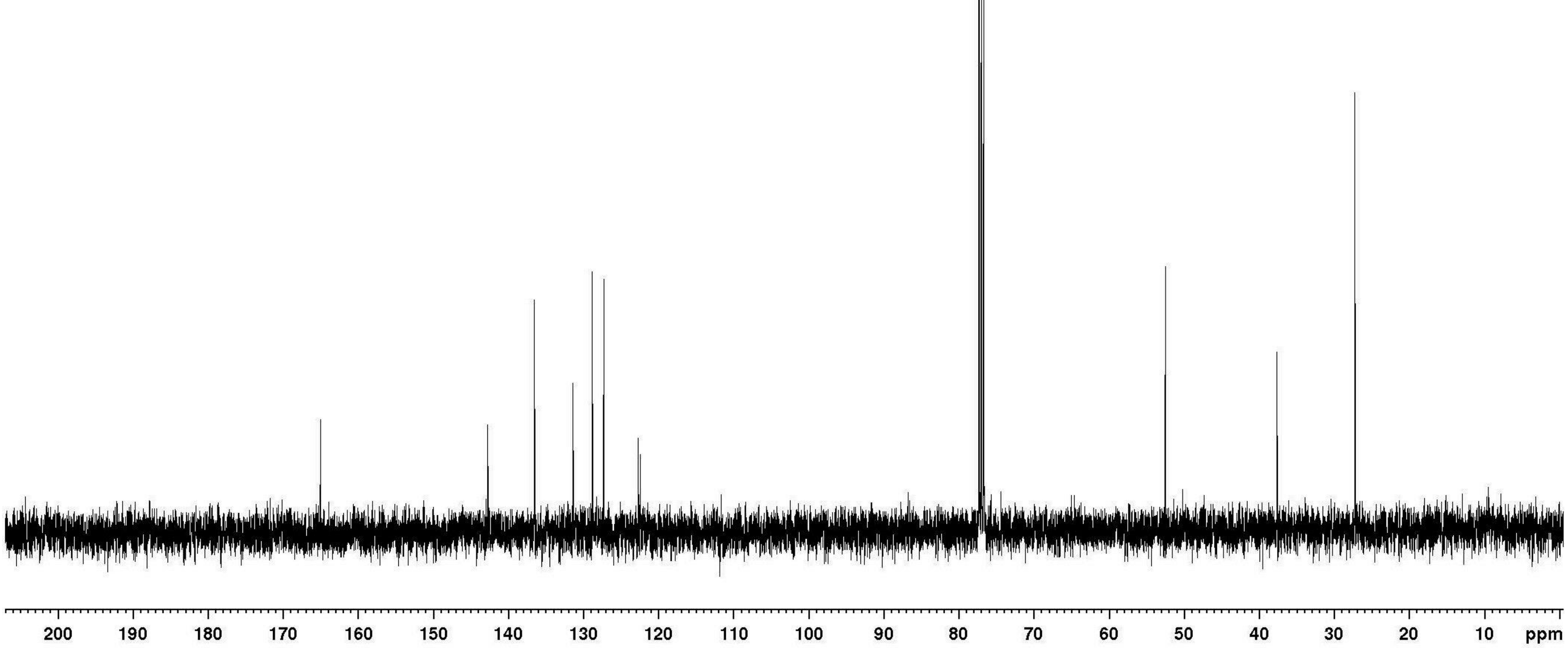

S2f

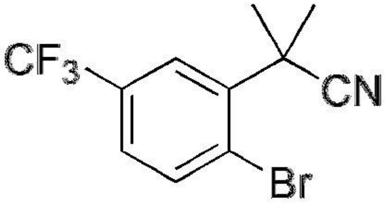

^1H NMR ($CDCl_3$, 400 MHz)

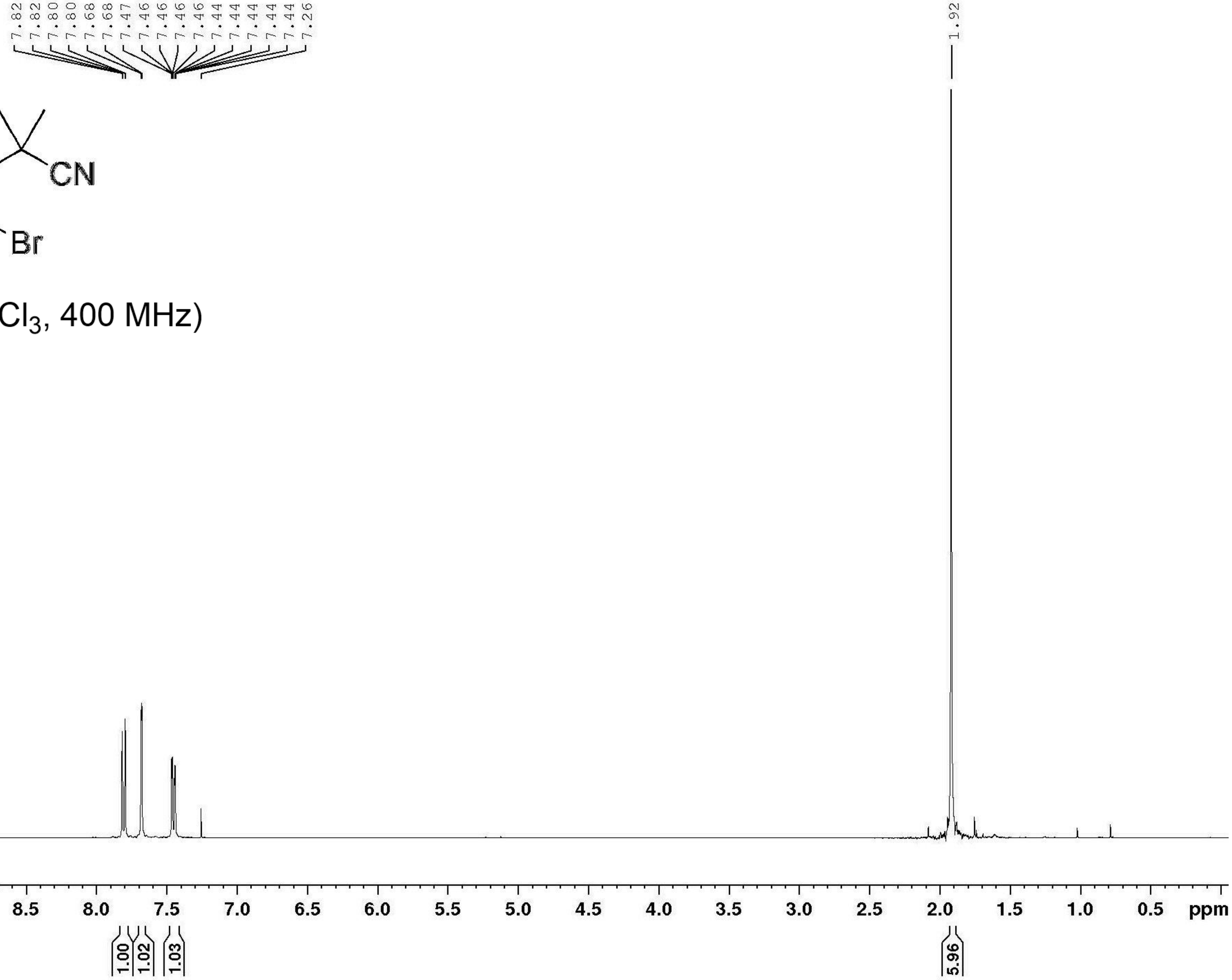

S2f

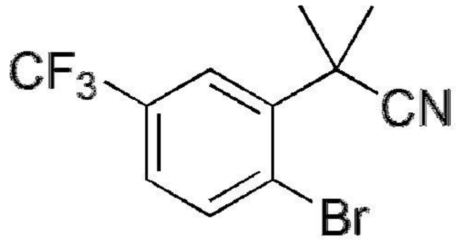

^{13}C NMR ($CDCl_3$, 100 MHz)

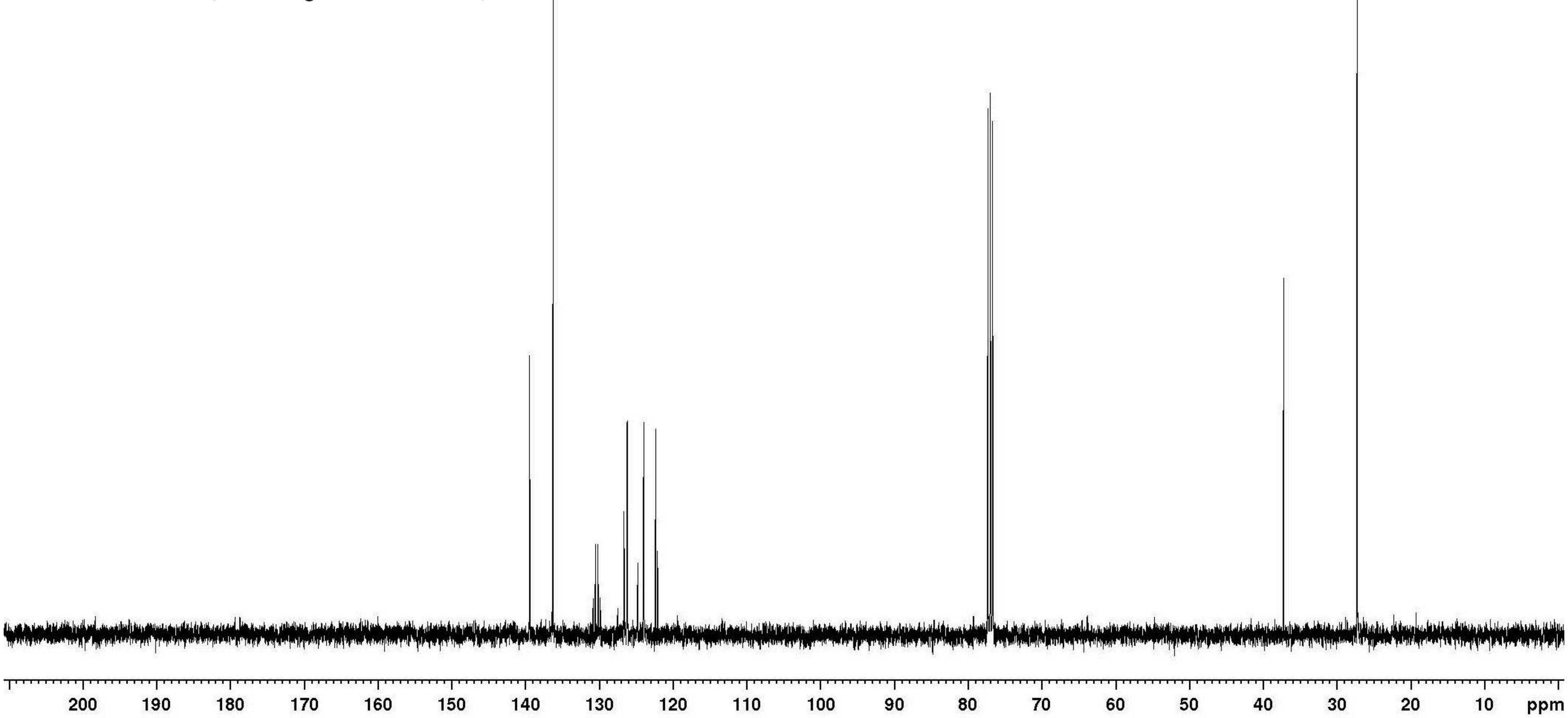

S2f

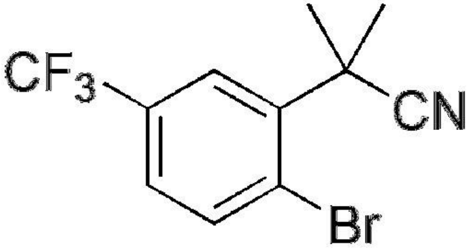

^{19}F NMR ($CDCl_3$, 376 MHz)

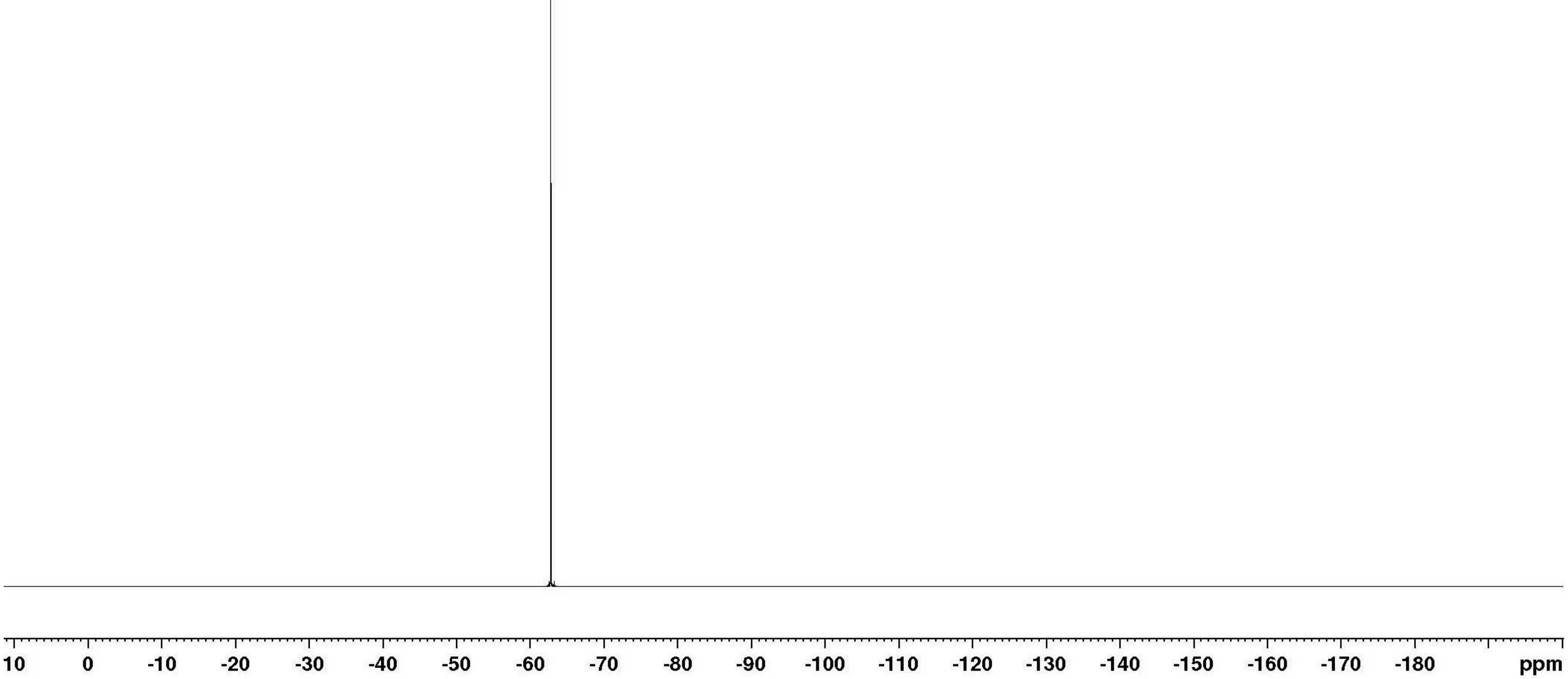

S2g

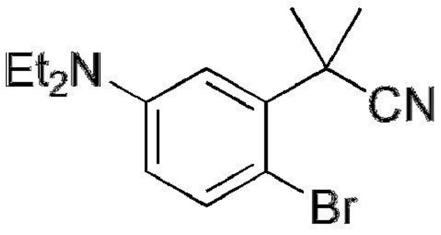

^{1}H NMR ($CDCl_3$, 400 MHz)

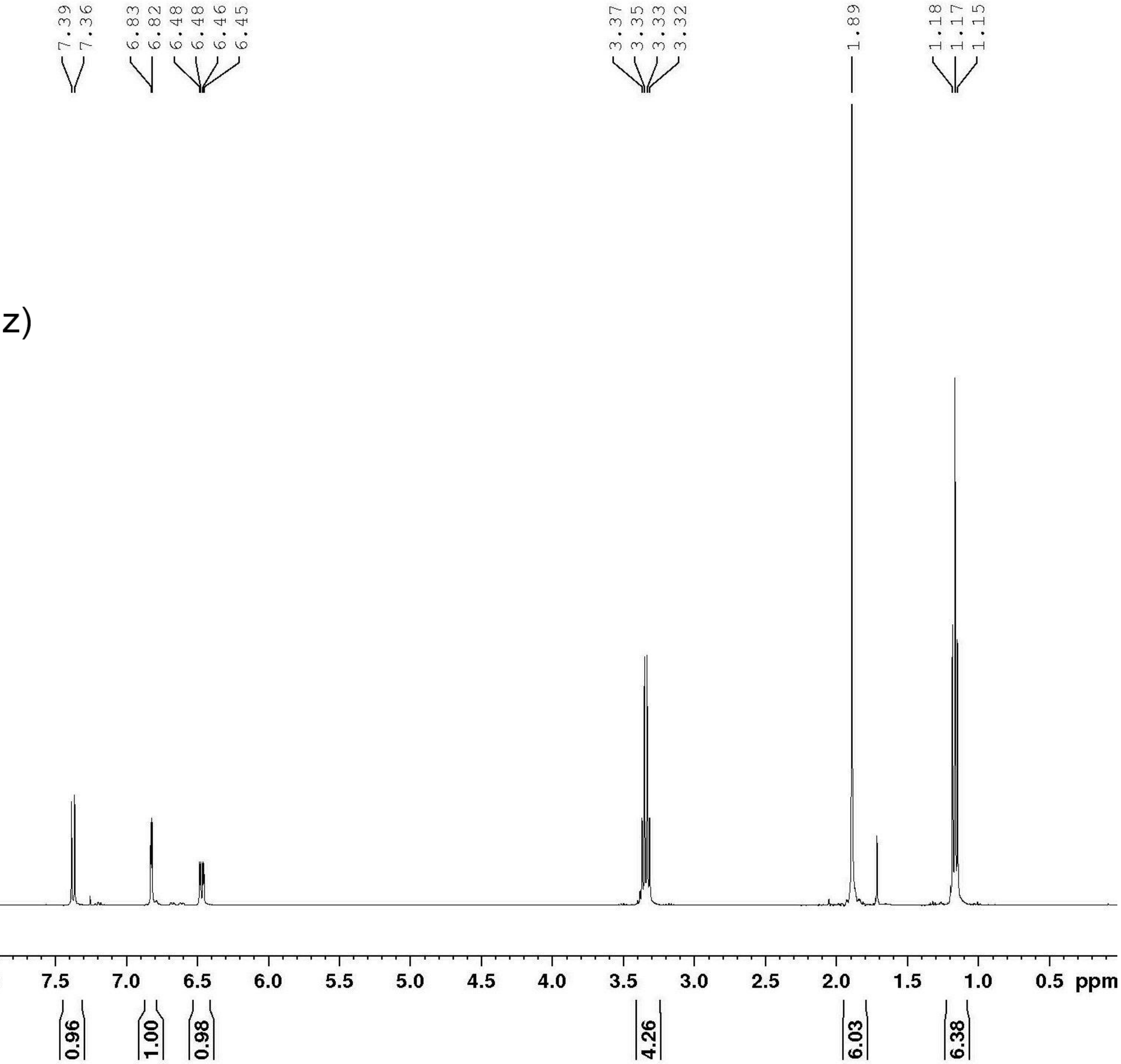

S2g

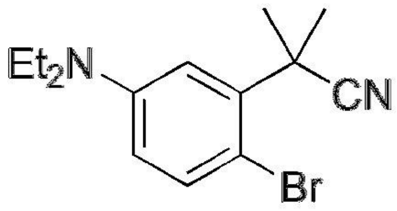

^{13}C NMR ($CDCl_3$, 100 MHz)

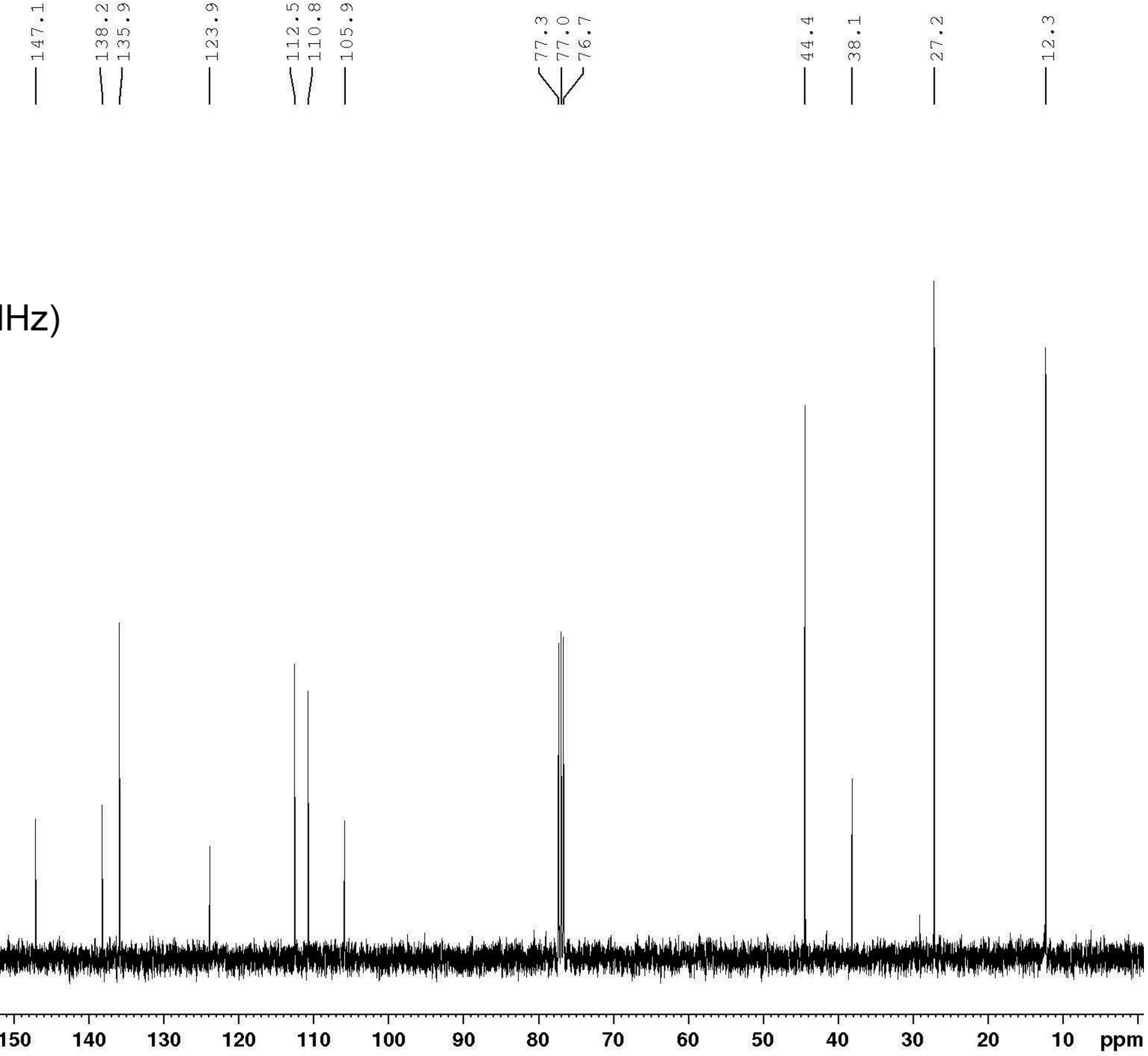

S2h

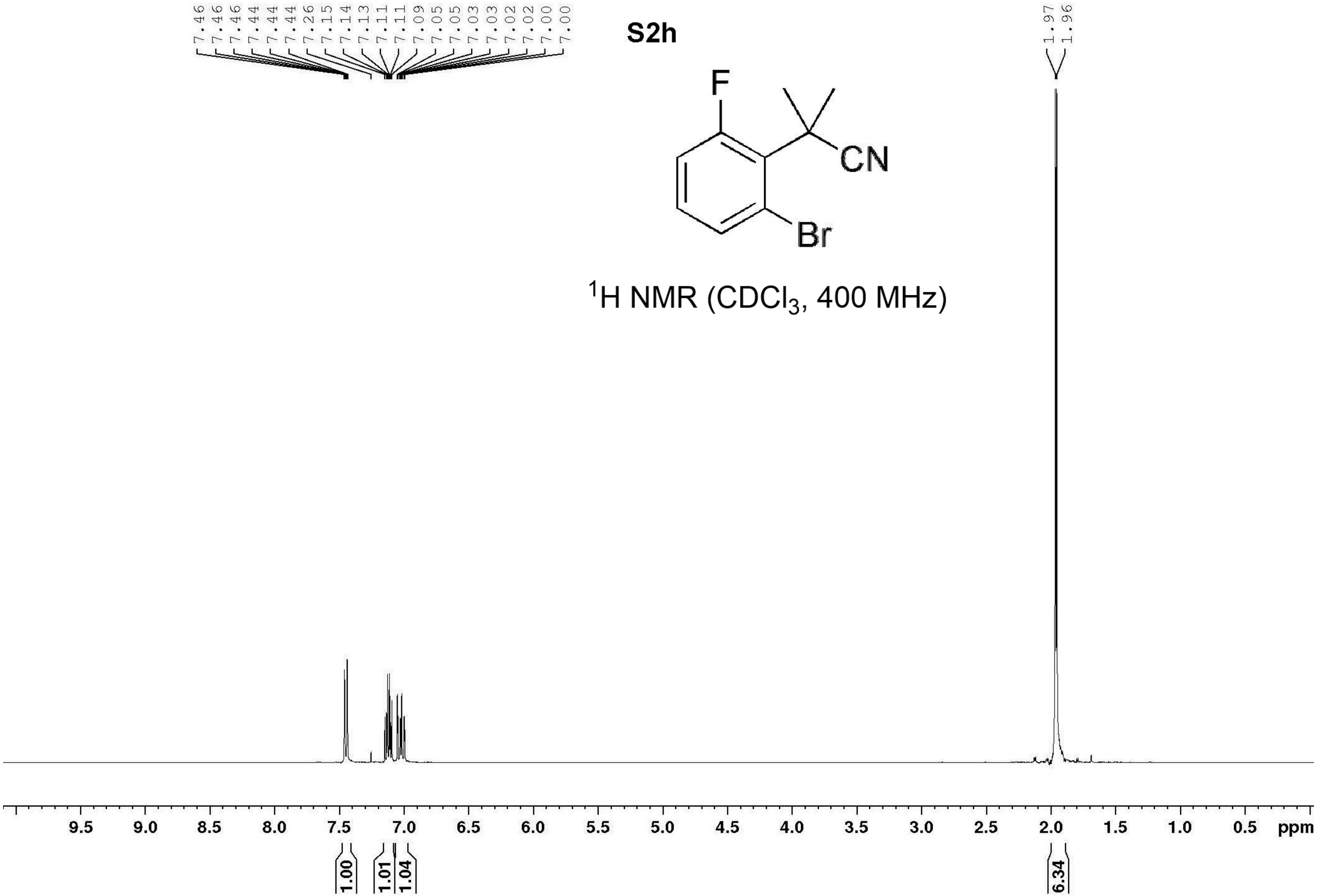

F
CN
Br
^{1}H NMR (CDCl$_3$, 400 MHz)
7.46
7.46
7.46
7.44
7.44
7.44
7.26
7.15
7.14
7.13
7.11
7.11
7.09
7.05
7.05
7.03
7.03
7.02
7.02
7.00
7.00
1.97
1.96
9.5
9.0
8.5
8.0
7.5
7.0
6.5
6.0
5.5
5.0
4.5
4.0
3.5
3.0
2.5
2.0
1.5
1.0
0.5
ppm
1.00
1.01
1.04
6.34

S2h

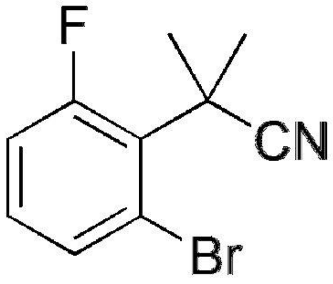

^{13}C NMR ($CDCl_3$, 100 MHz)

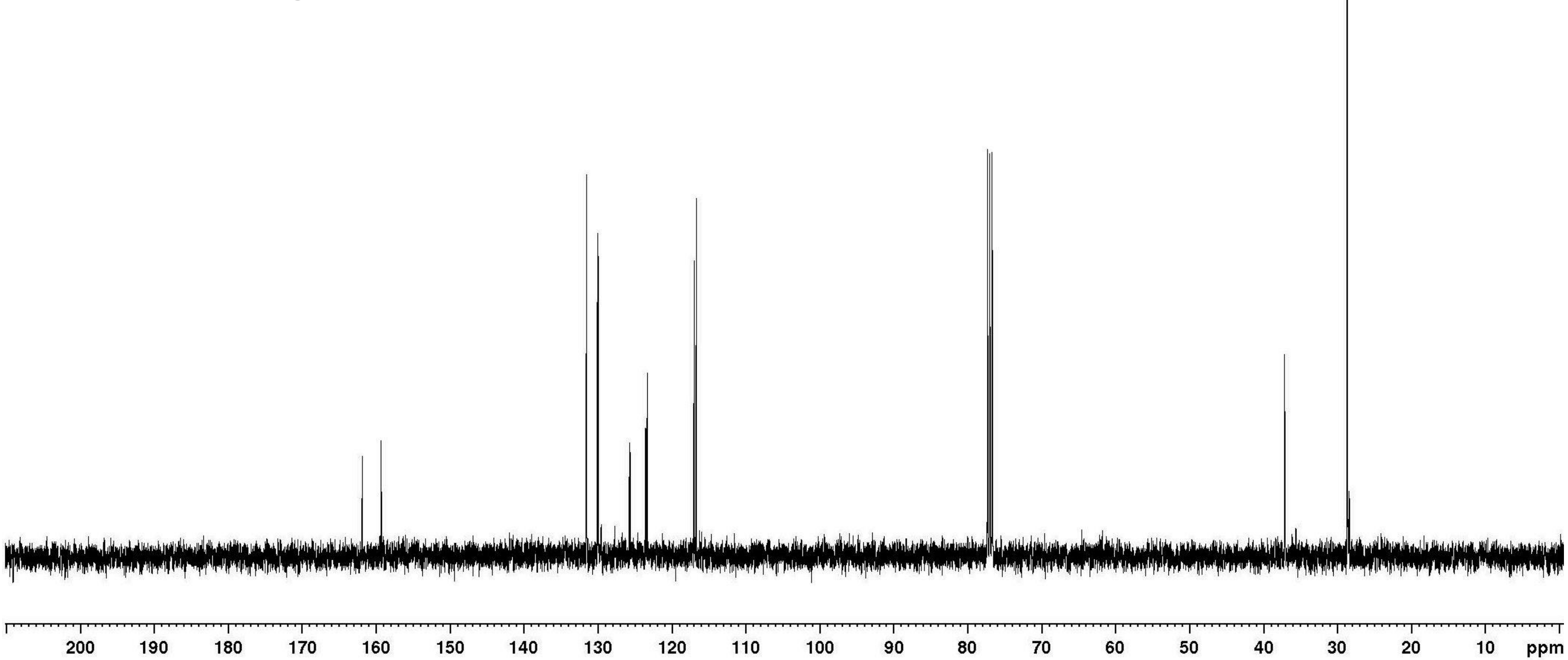

S2h

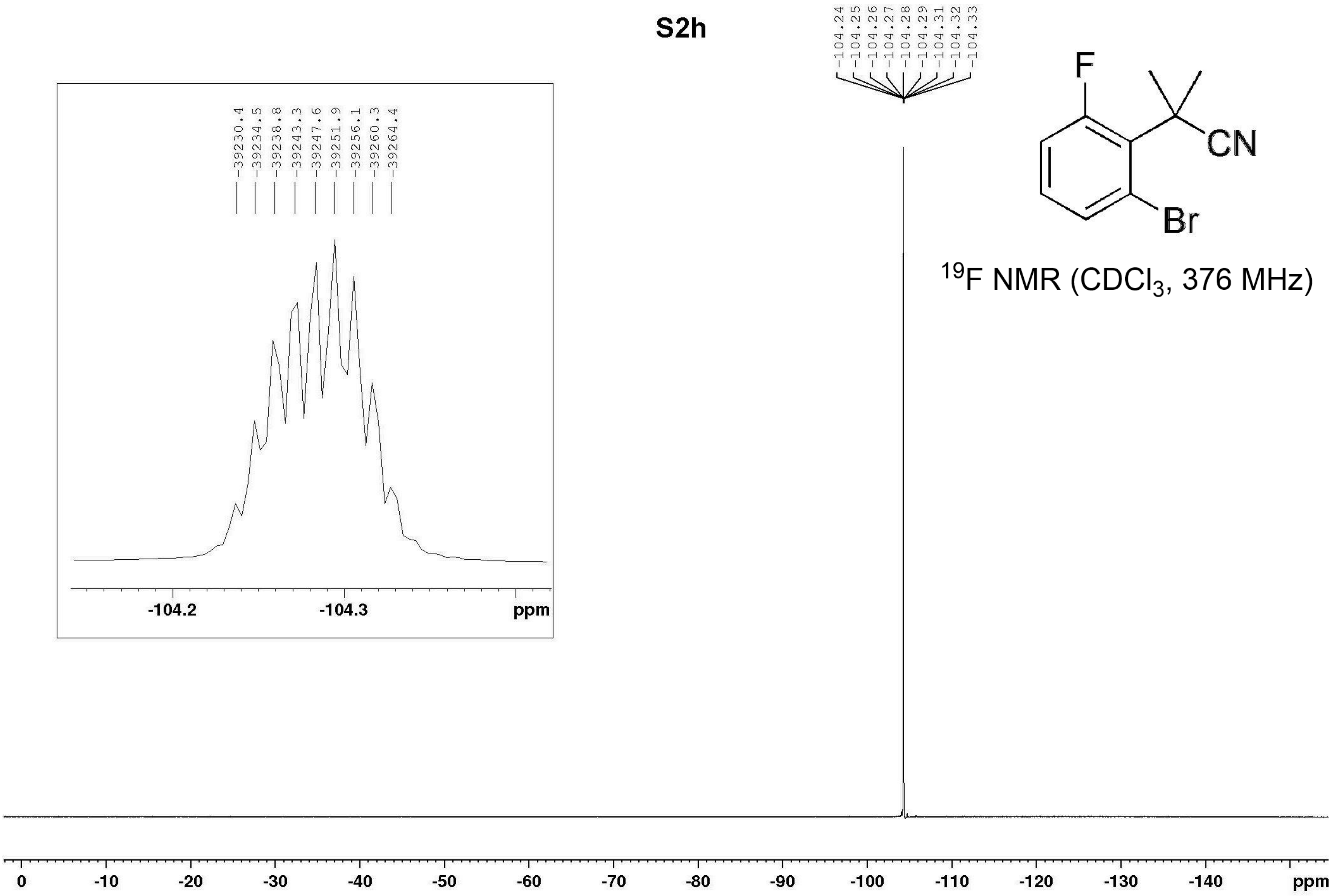

^{19}F NMR ($CDCl_3$, 376 MHz)

S2n

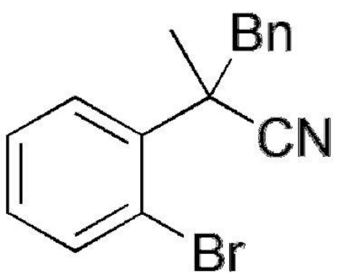

^1H NMR ($CDCl_3$, 400 MHz)

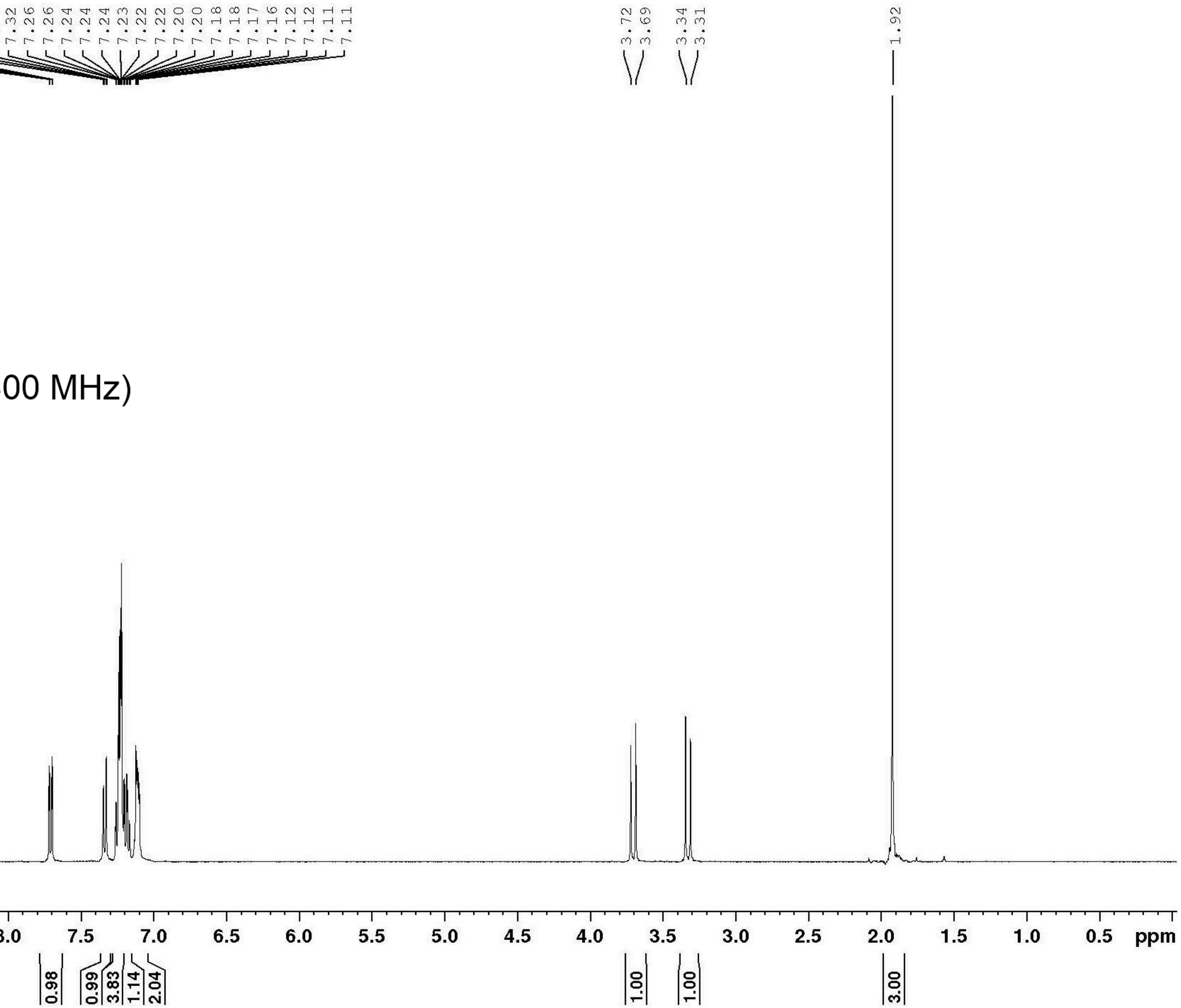

S2n

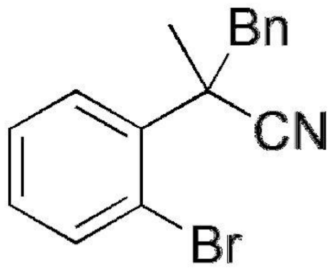

^{13}C NMR ($CDCl_3$, 100 MHz)

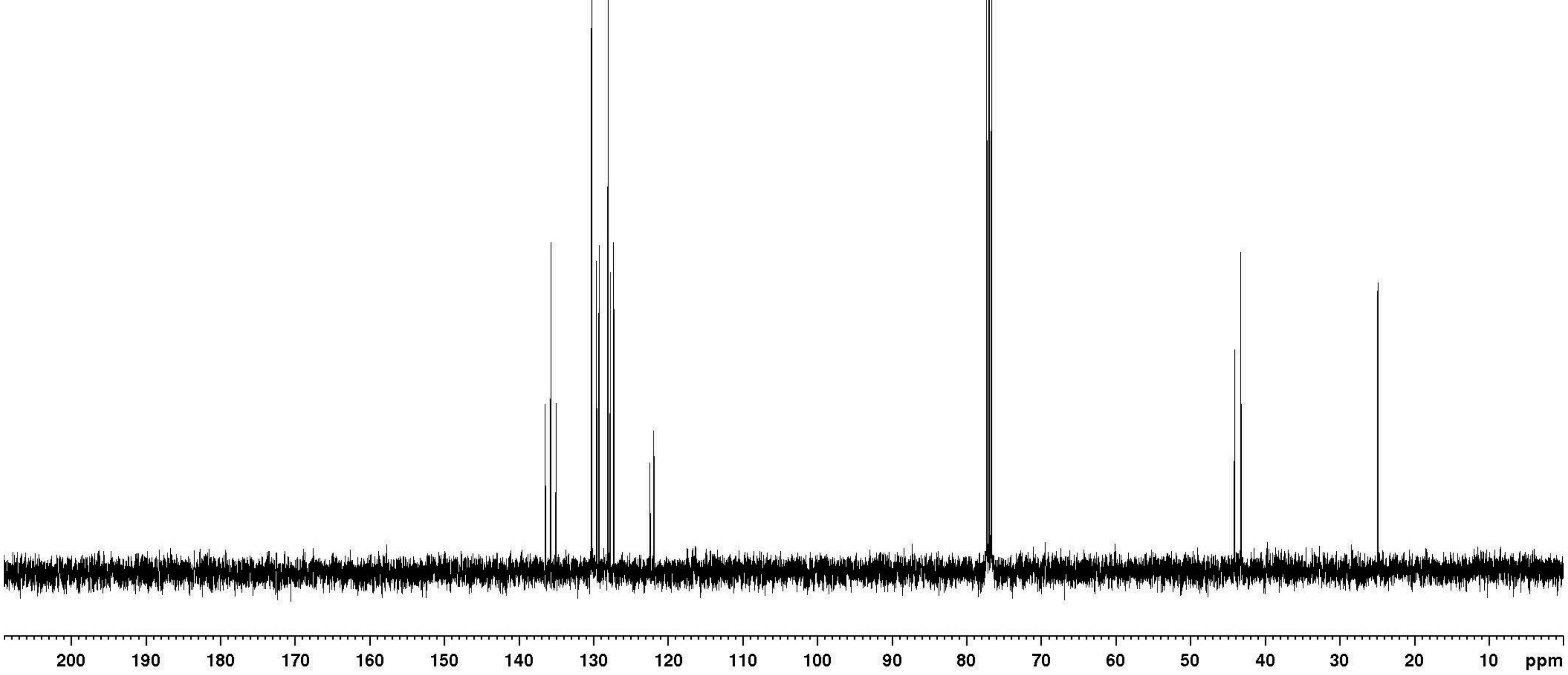

S2o

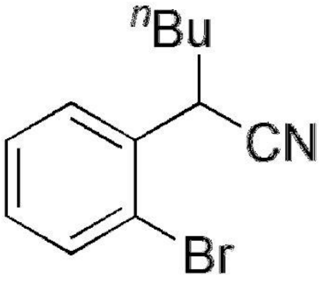

^{1}H NMR ($CDCl_3$, 400 MHz)

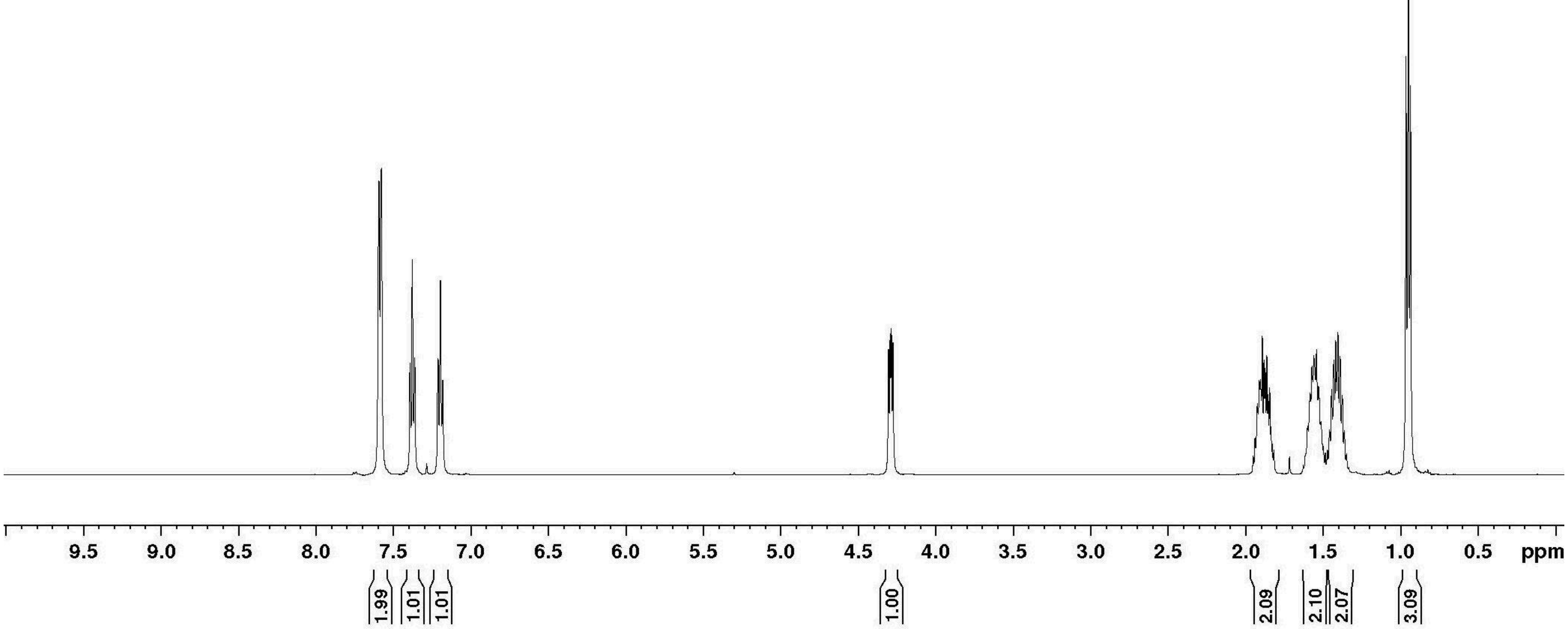

S2o

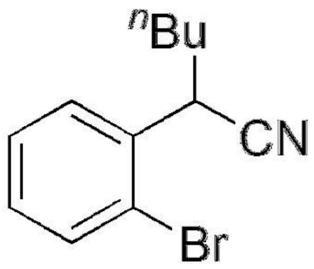

^{13}C NMR ($CDCl_3$, 100 MHz)

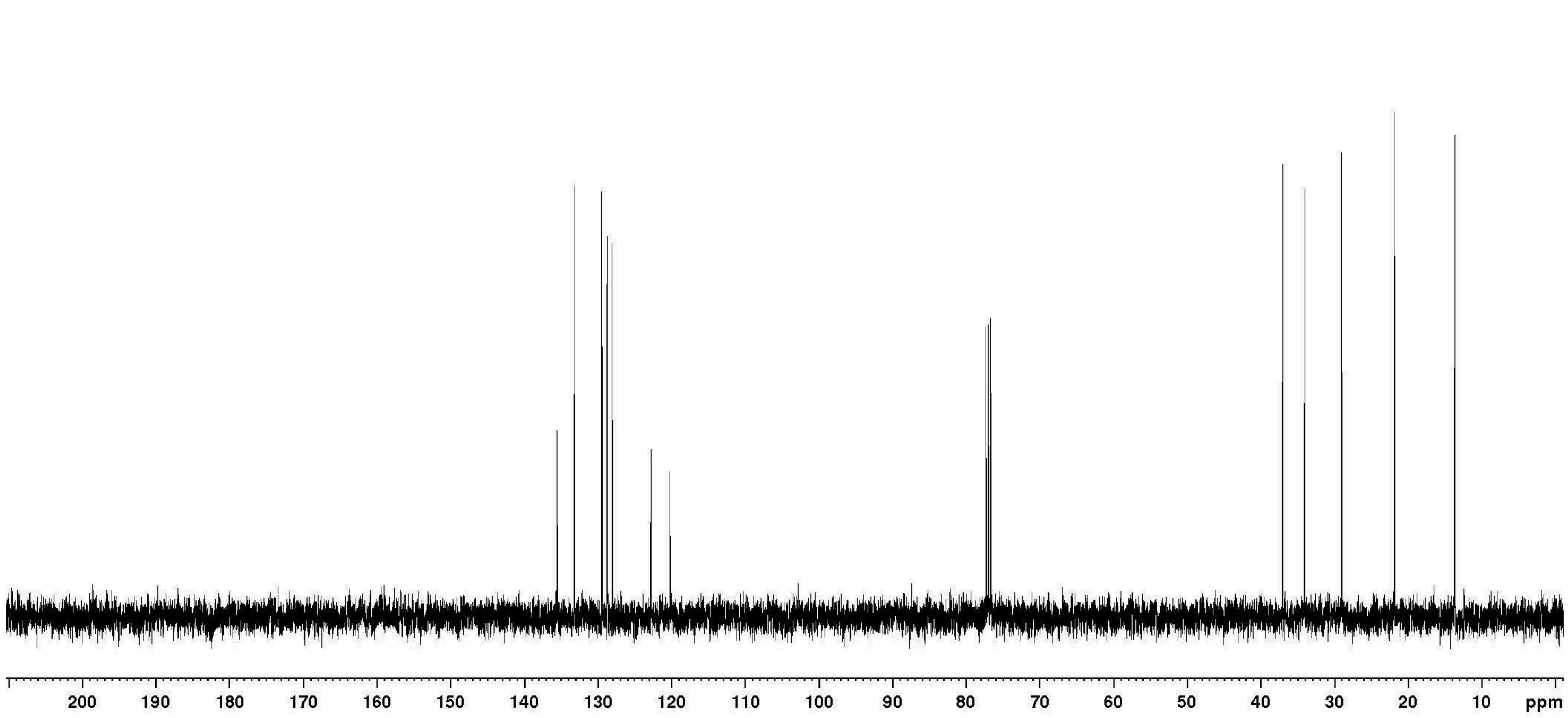

S2p

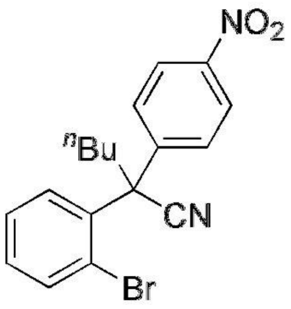

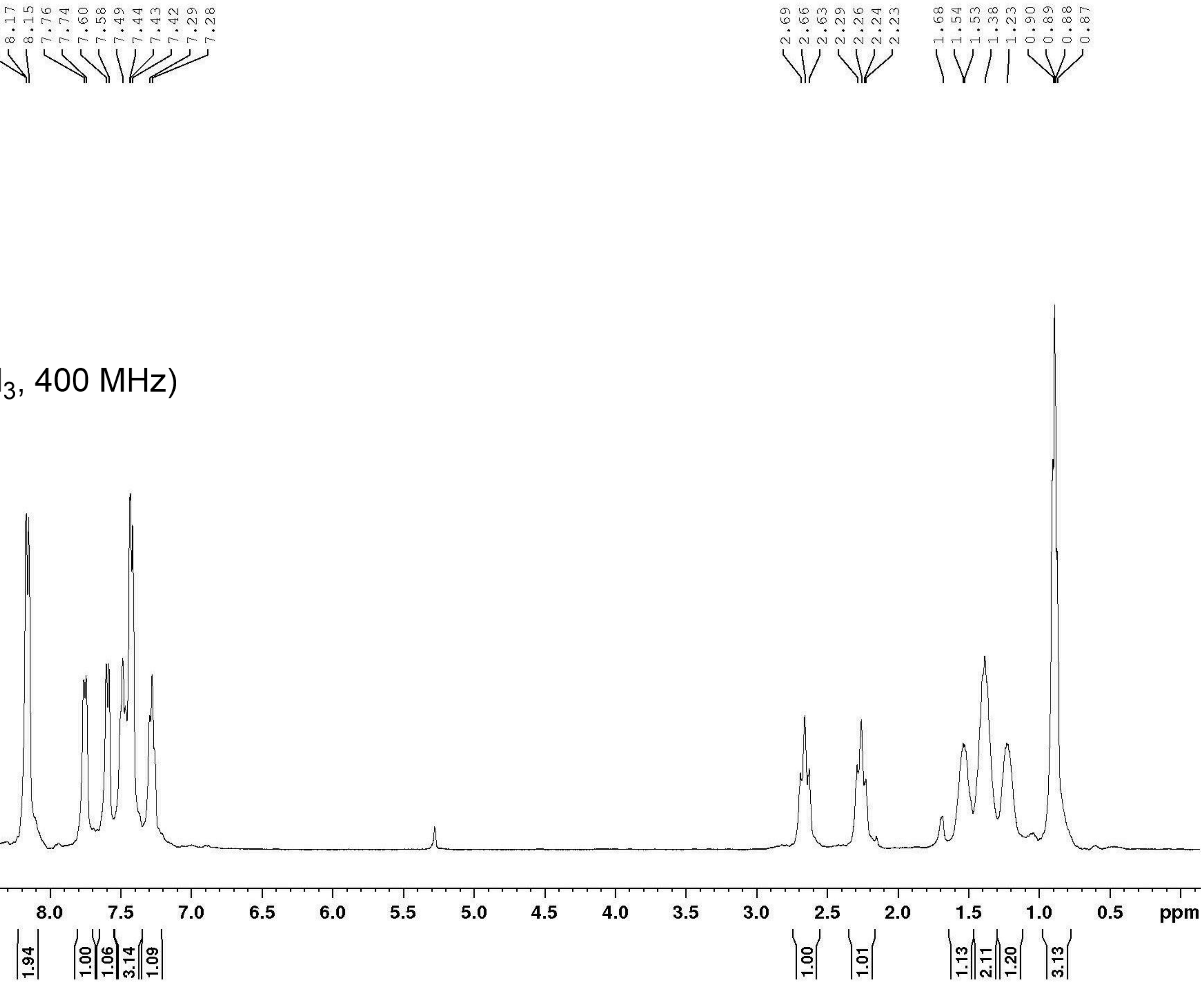

8.17
8.17
8.15
7.76
7.74
7.60
7.58
7.49
7.44
7.43
7.42
7.29
7.28
2.69
2.66
2.63
2.29
2.26
2.24
2.23
1.68
1.54
1.53
1.38
1.23
0.90
0.89
0.88
0.87
^{1}H NMR ($CDCl_3$, 400 MHz)
9.5
9.0
8.5
8.0
7.5
7.0
6.5
6.0
5.5
5.0
4.5
4.0
3.5
3.0
2.5
2.0
1.5
1.0
0.5
ppm
1.94
1.00
1.06
3.14
1.09
1.00
1.01
1.13
2.11
1.20
3.13

S2p

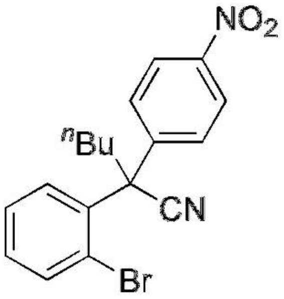

^{13}C NMR ($CDCl_3$, 100 MHz)

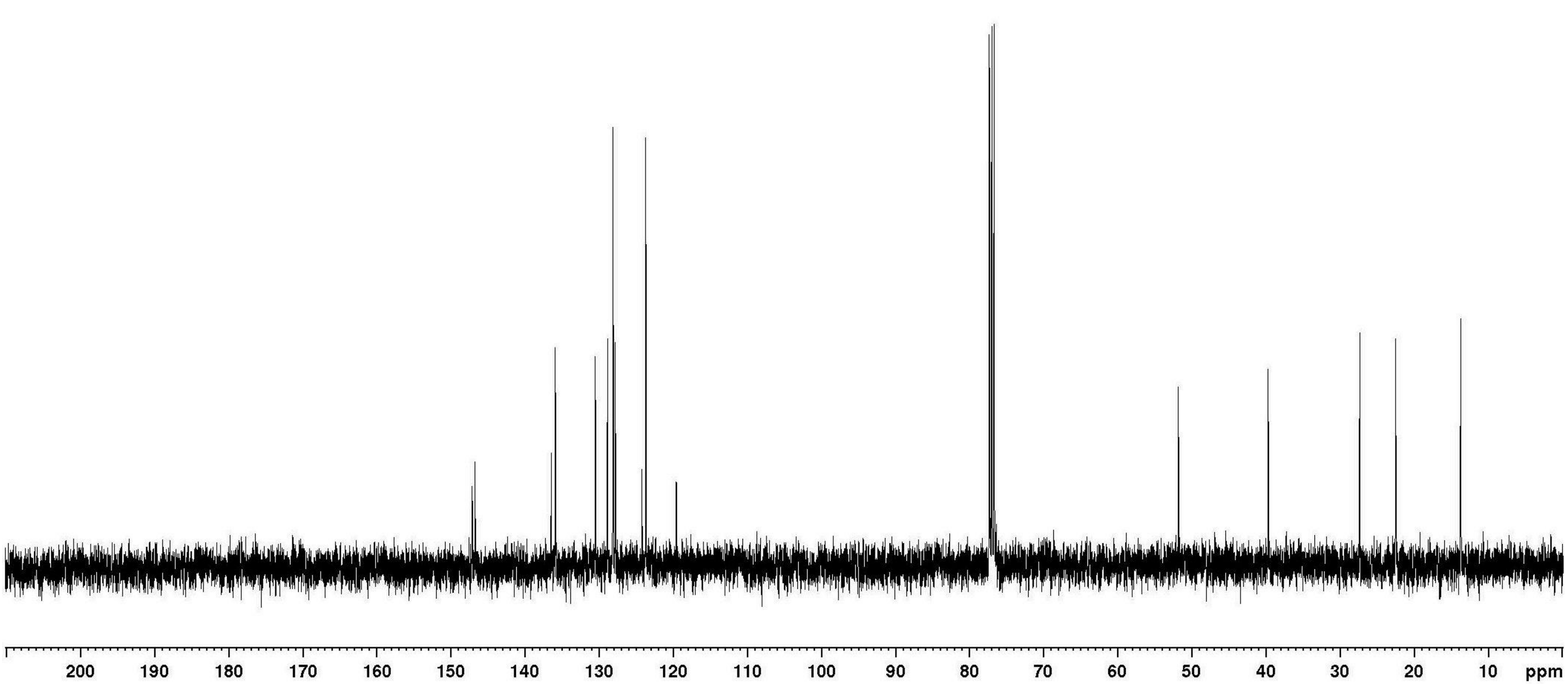

S2v

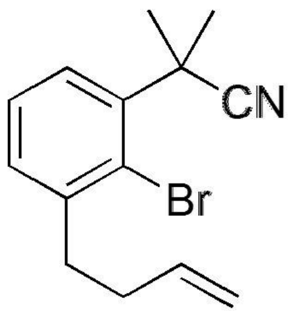

^{1}H NMR ($CDCl_3$, 400 MHz)

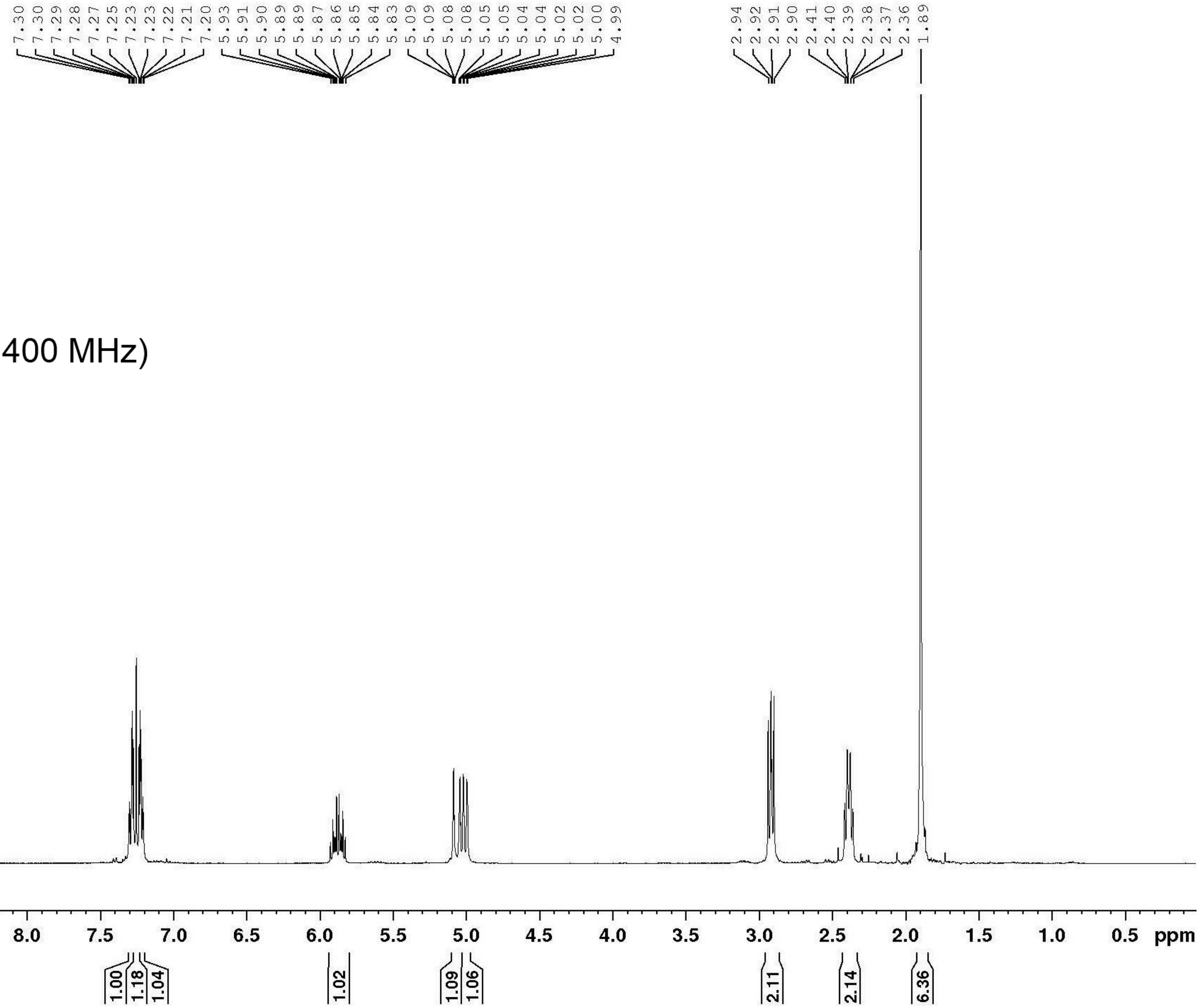

S2v

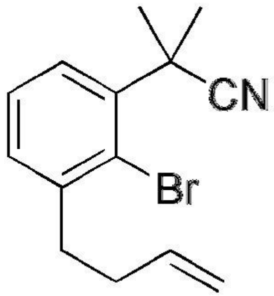

^{13}C NMR (CDCl$_3$, 100 MHz)

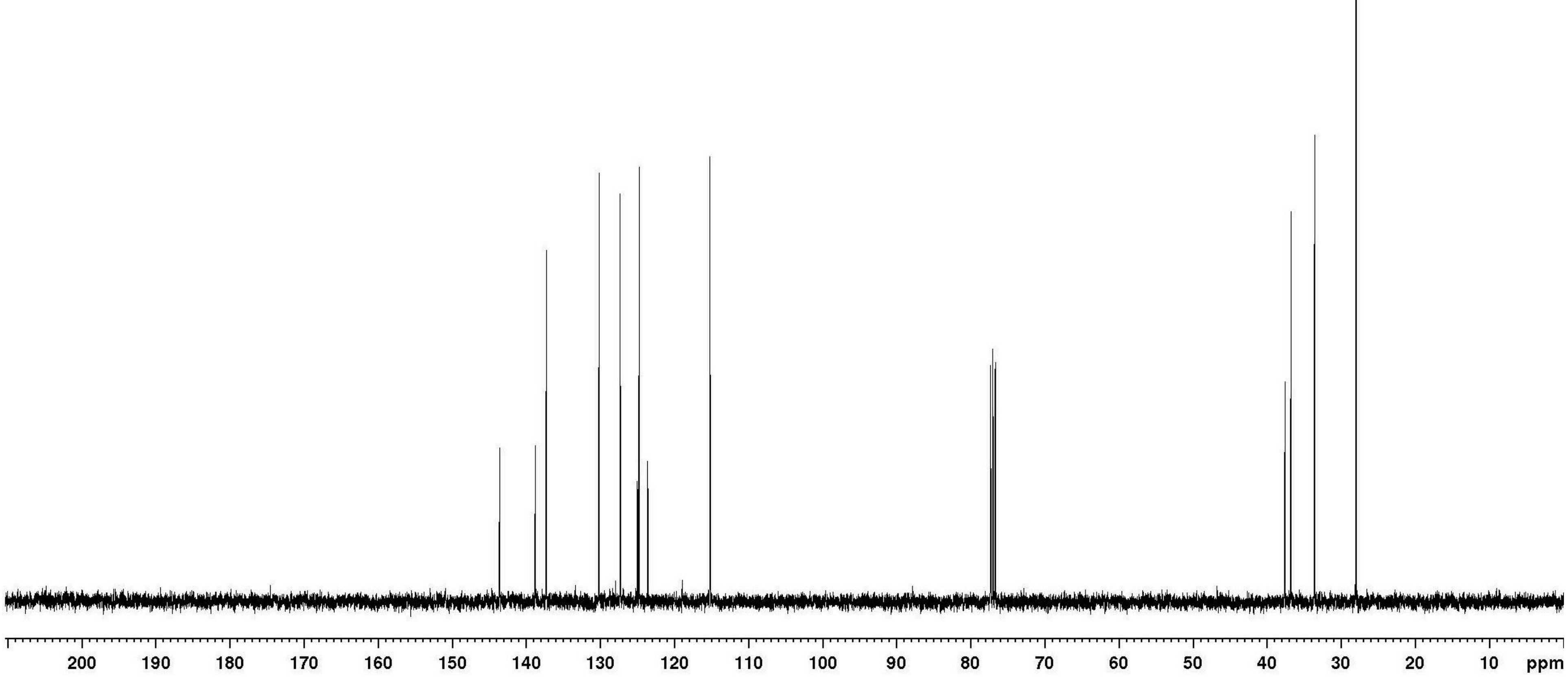

S3b

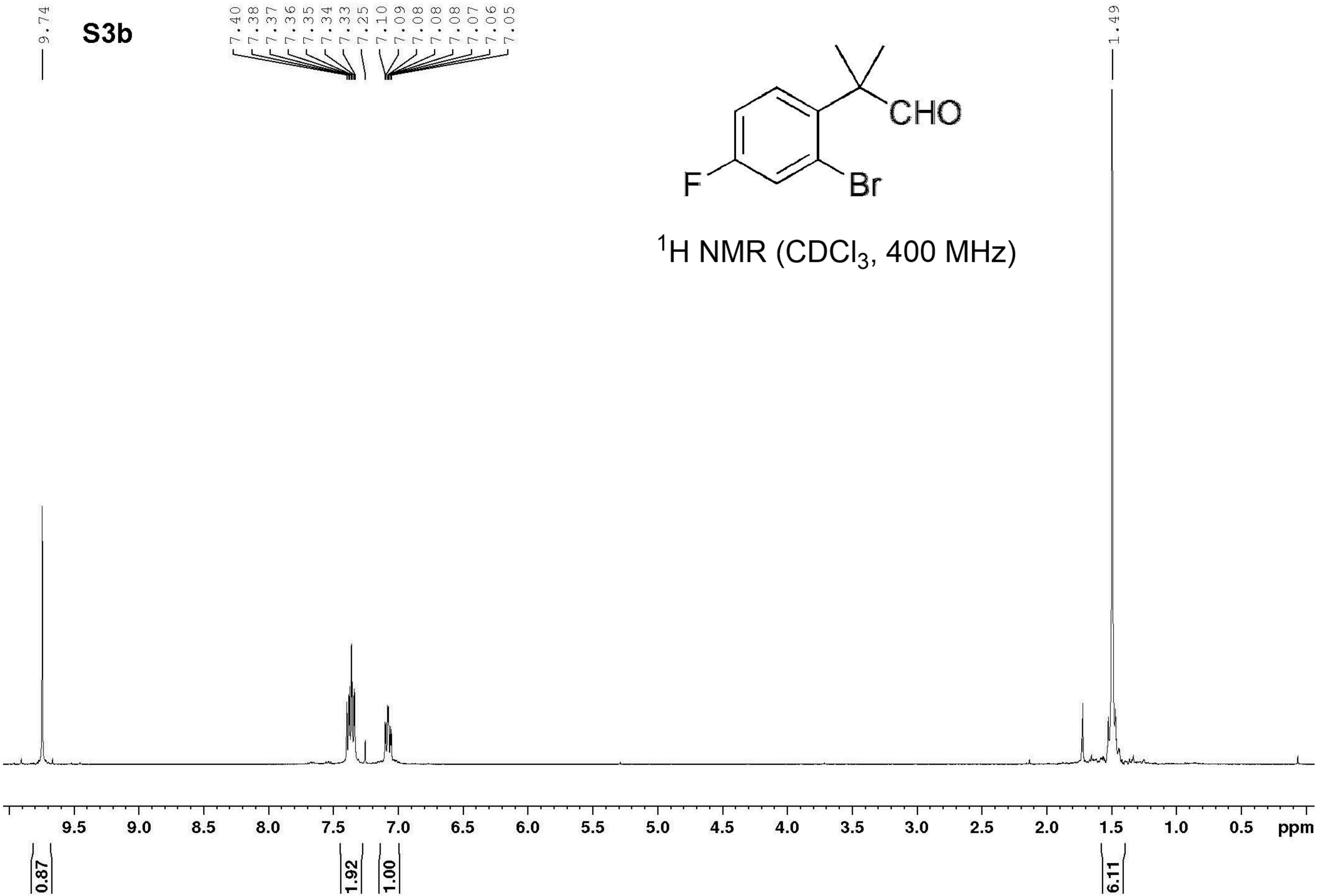

^1H NMR ($CDCl_3$, 400 MHz)

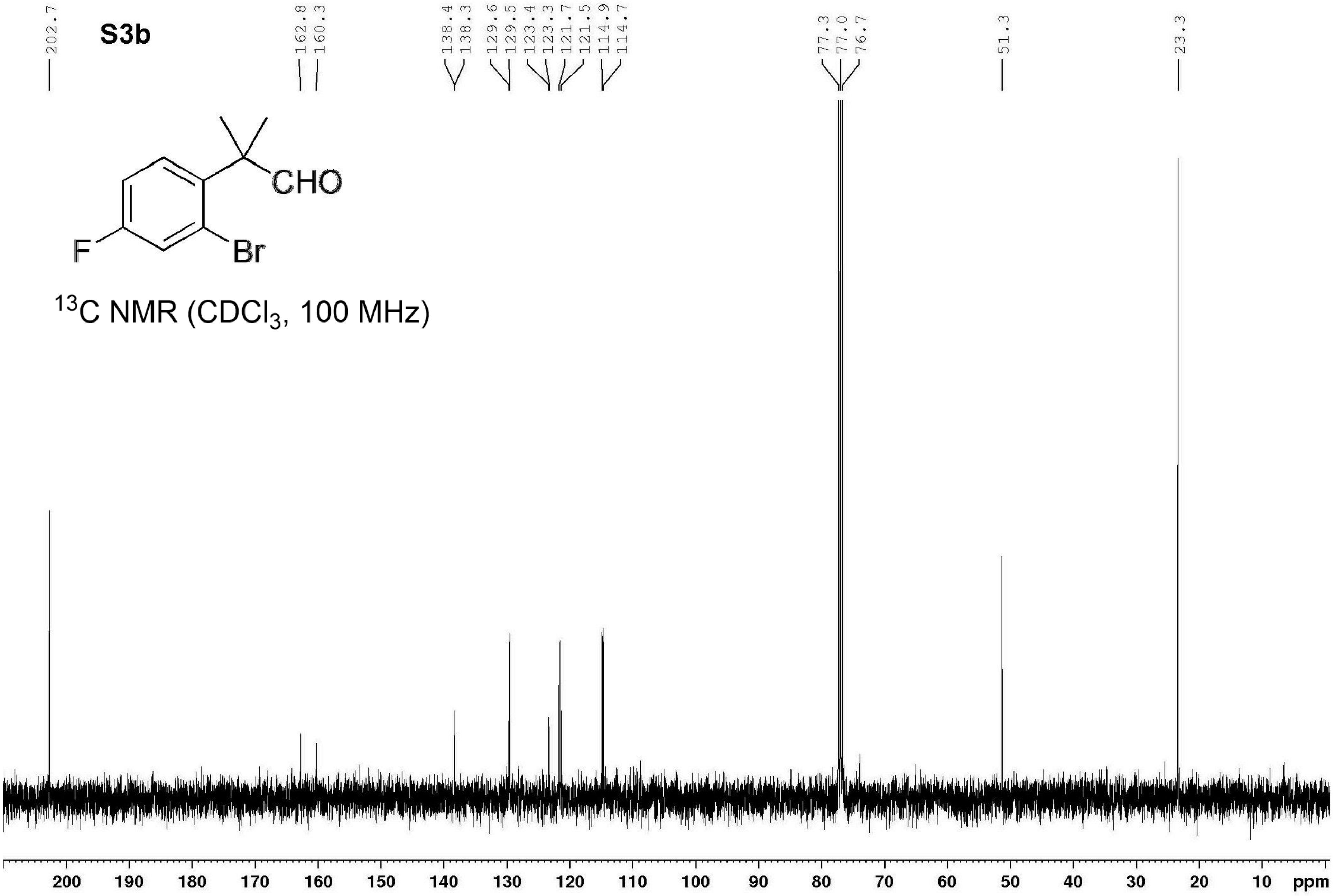
202.7
S3b
162.8
160.3
138.4
138.3
129.6
129.5
123.4
123.3
121.7
121.5
114.9
114.7
77.3
77.0
76.7
51.3
23.3
CHO
F
Br
^{13}C NMR ($CDCl_3$, 100 MHz)
200 190 180 170 160 150 140 130 120 110 100 90 80 70 60 50 40 30 20 10 ppm

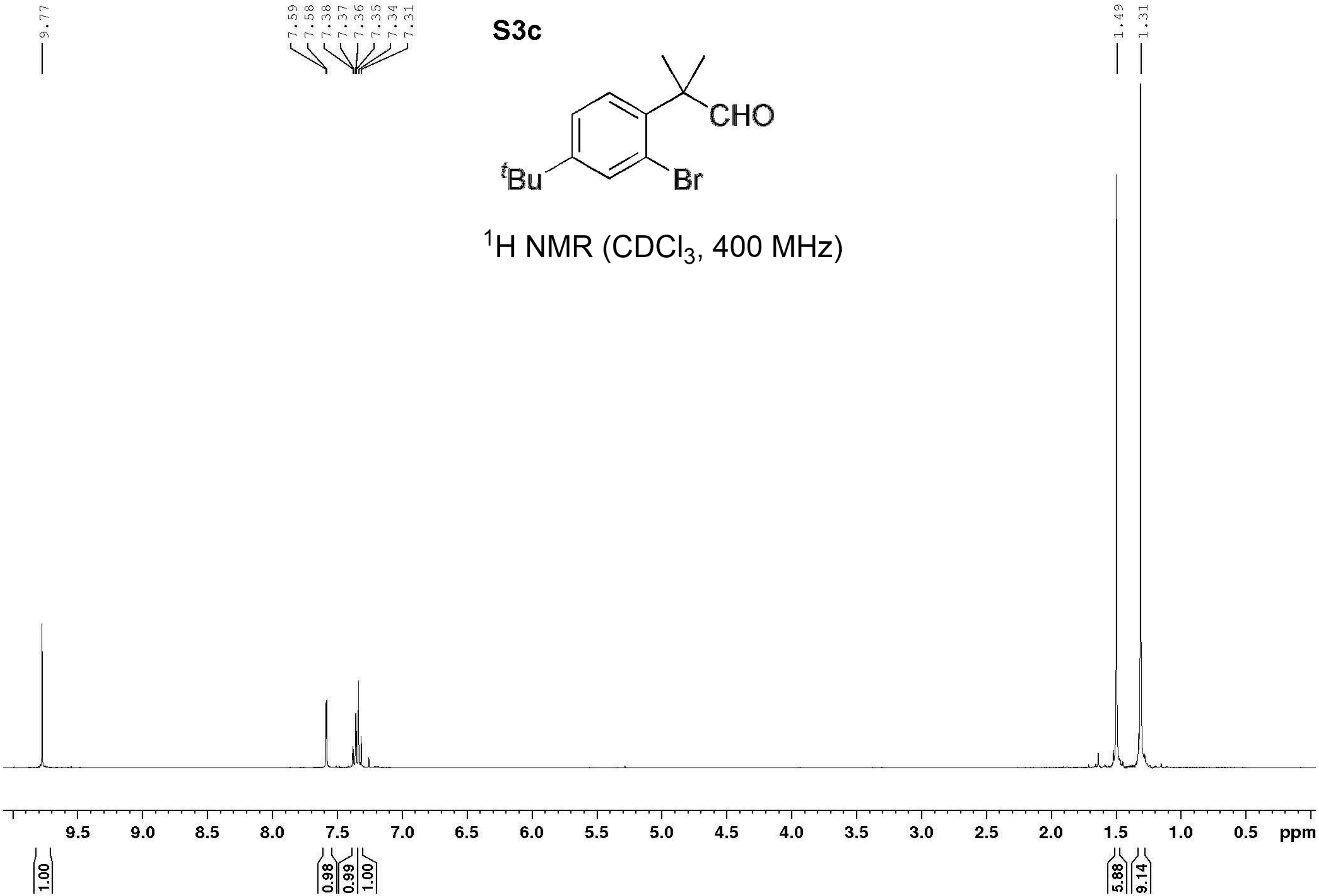

S3c
CHO
tBu
Br
1H NMR (CDCl3, 400 MHz)
9.77
7.59
7.58
7.38
7.37
7.36
7.35
7.34
7.31
1.49
1.31
9.5
9.0
8.5
8.0
7.5
7.0
6.5
6.0
5.5
5.0
4.5
4.0
3.5
3.0
2.5
2.0
1.5
1.0
0.5
ppm
1.00
0.98
0.99
1.00
5.88
9.14

S3c

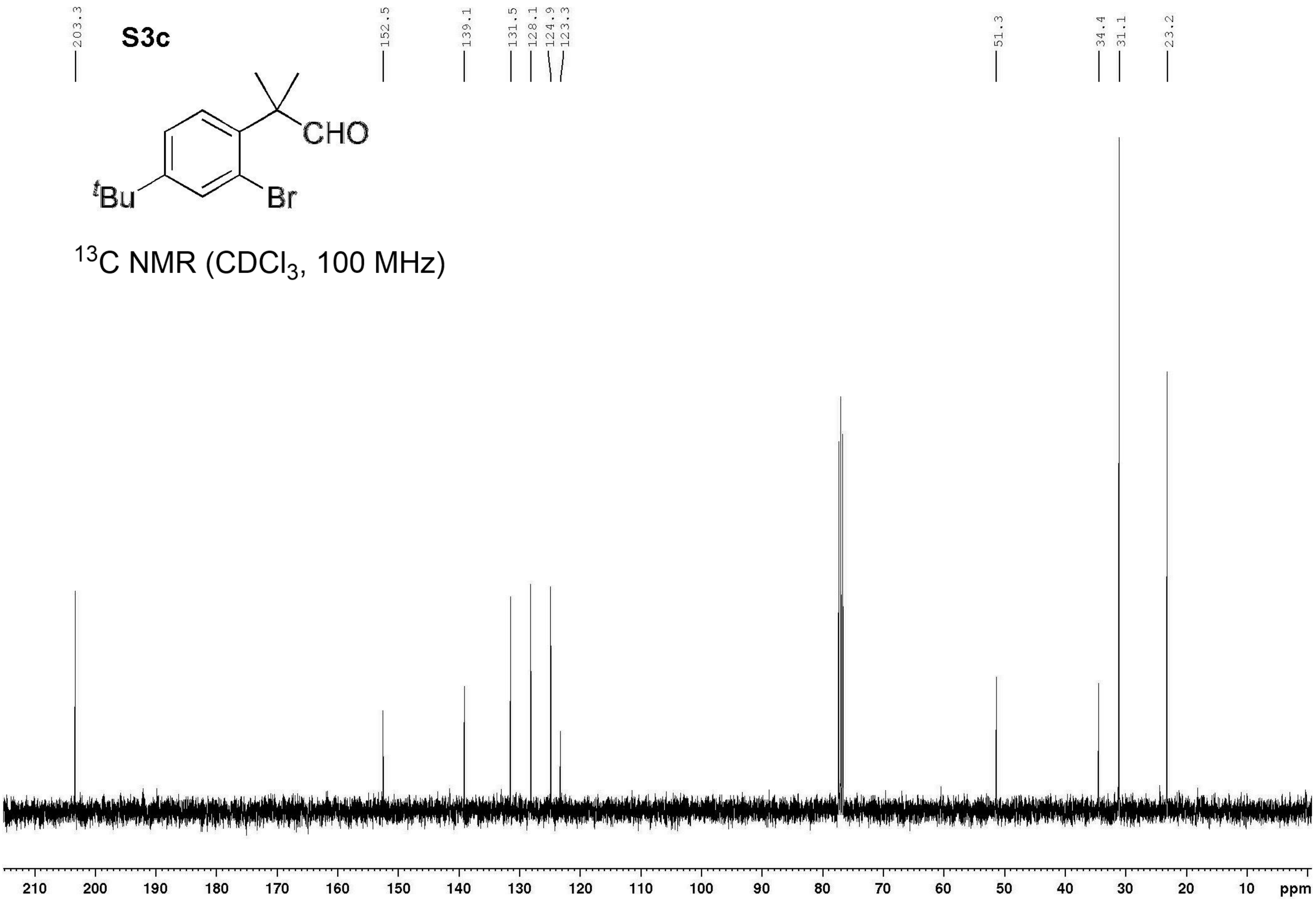

^{13}C NMR ($CDCl_3$, 100 MHz)

S3d

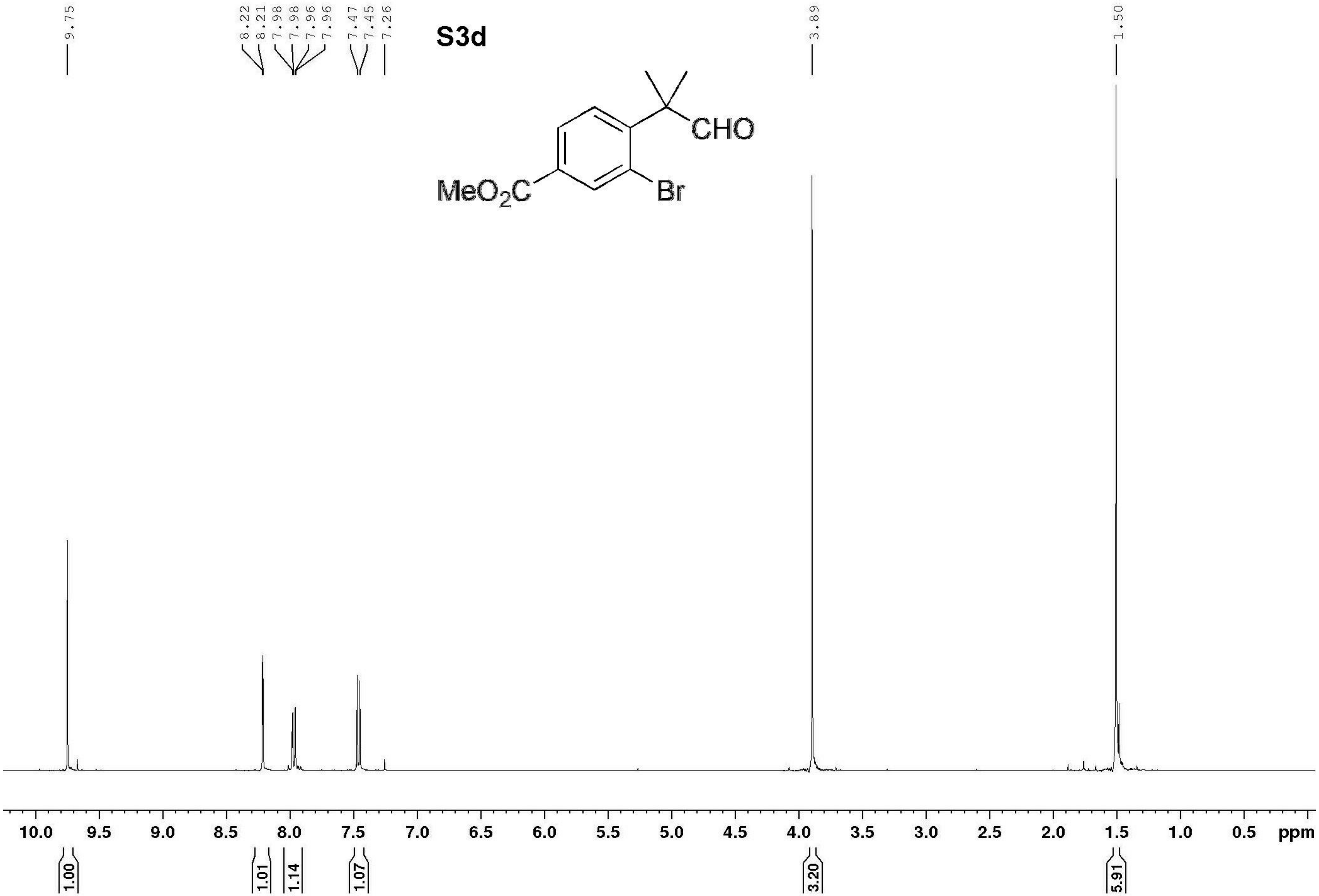
9.75
8.22
8.21
7.98
7.98
7.96
7.96
7.47
7.45
7.26
3.89
1.50
CHO
MeO2C
Br
10.0
9.5
9.0
8.5
8.0
7.5
7.0
6.5
6.0
5.5
5.0
4.5
4.0
3.5
3.0
2.5
2.0
1.5
1.0
0.5
ppm
1.00
1.01
1.14
1.07
3.20
5.91

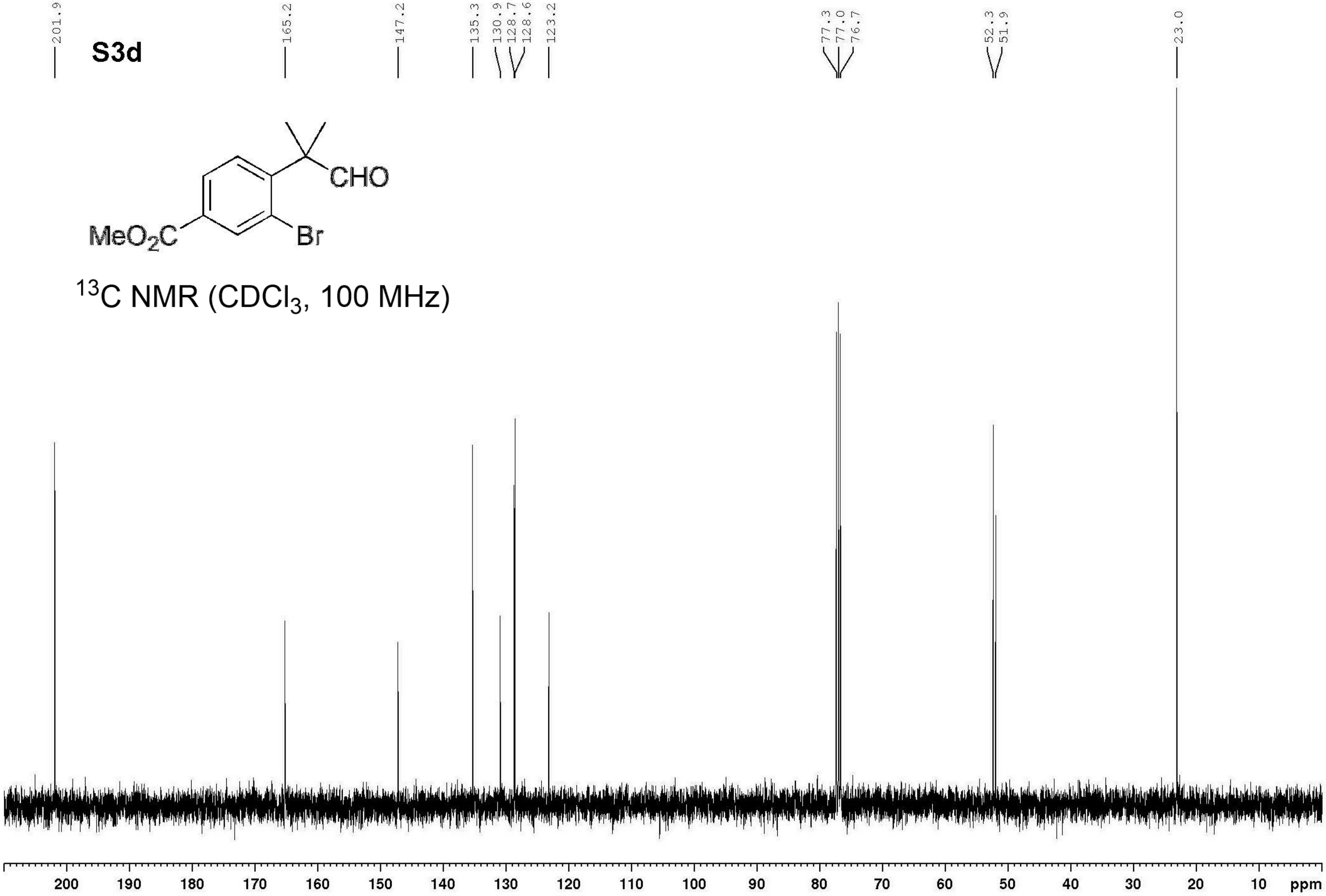
S3d
MeO2C
CHO
Br
13C NMR (CDCl3, 100 MHz)
201.9
165.2
147.2
135.3
130.9
128.7
128.6
123.2
77.3
77.0
76.7
52.3
51.9
23.0
200
190
180
170
160
150
140
130
120
110
100
90
80
70
60
50
40
30
20
10
ppm

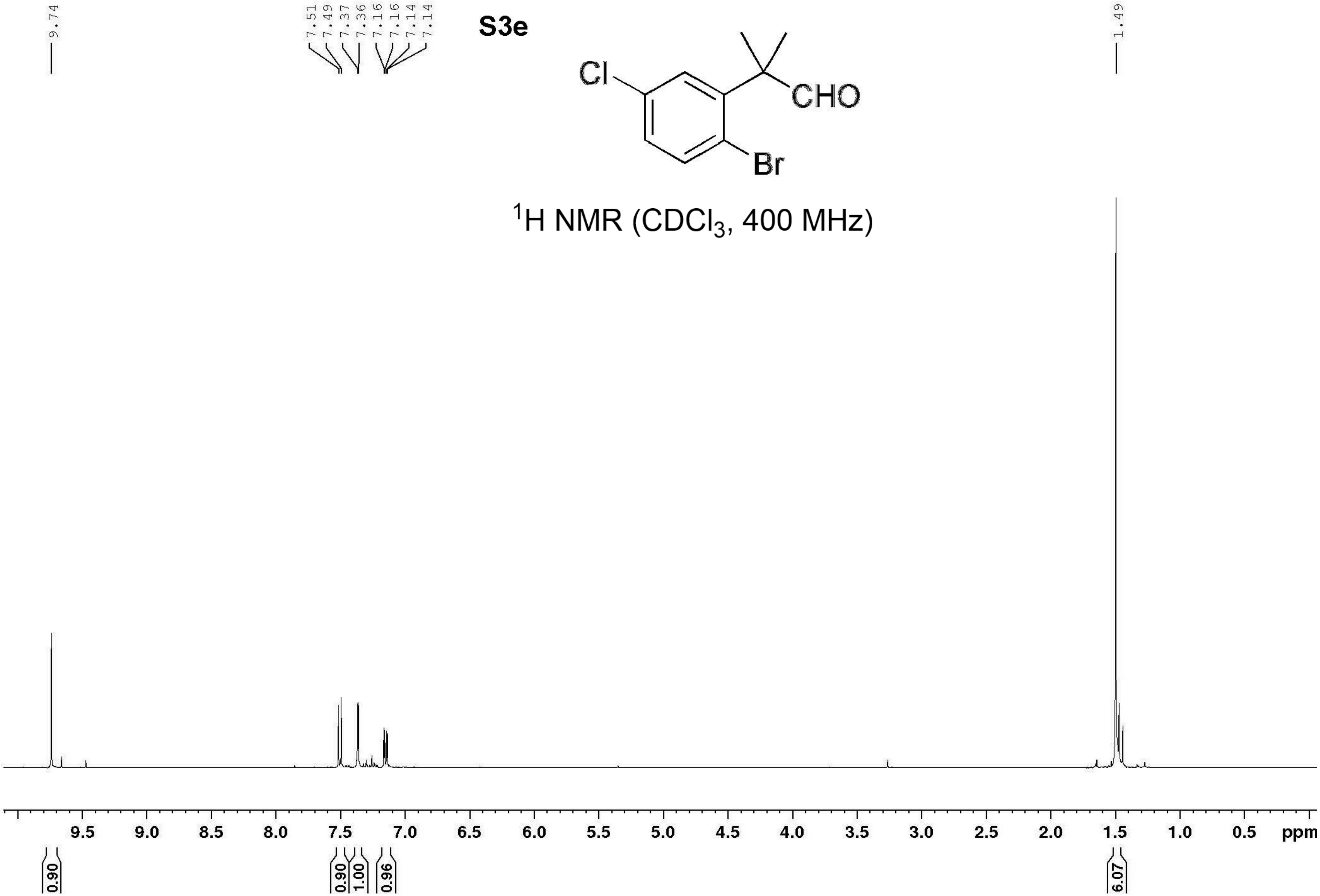
S3e
Cl
CHO
Br
^{1}H NMR ($CDCl_3$, 400 MHz)
9.74
7.51
7.49
7.37
7.36
7.16
7.16
7.14
7.14
1.49
9.5
9.0
8.5
8.0
7.5
7.0
6.5
6.0
5.5
5.0
4.5
4.0
3.5
3.0
2.5
2.0
1.5
1.0
0.5
ppm
0.90
0.90
1.00
0.96
6.07

S3e

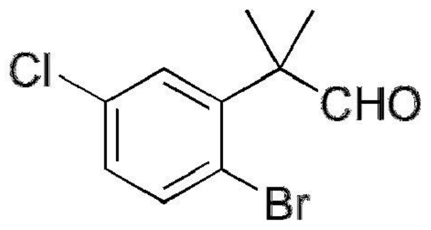

^{13}C NMR ($CDCl_3$, 100 MHz)

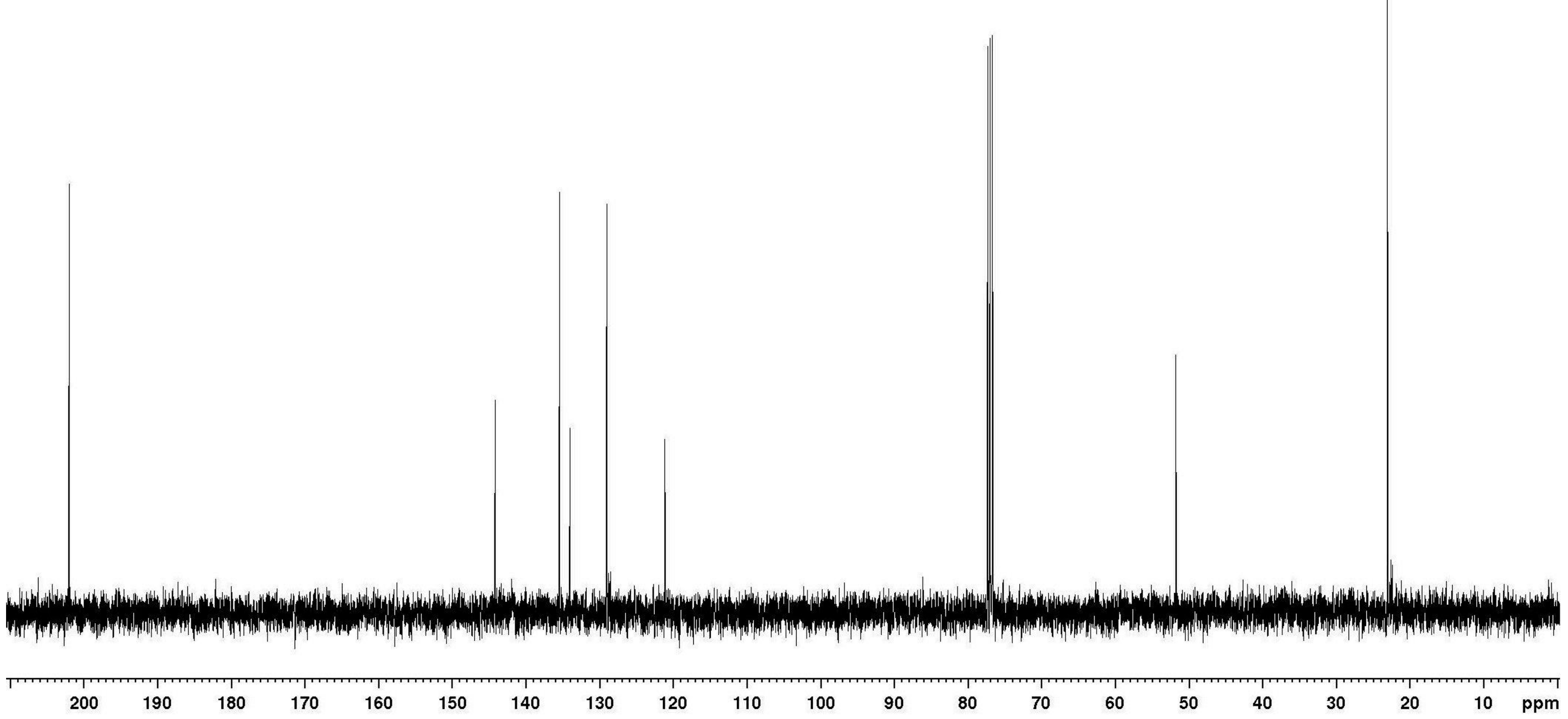

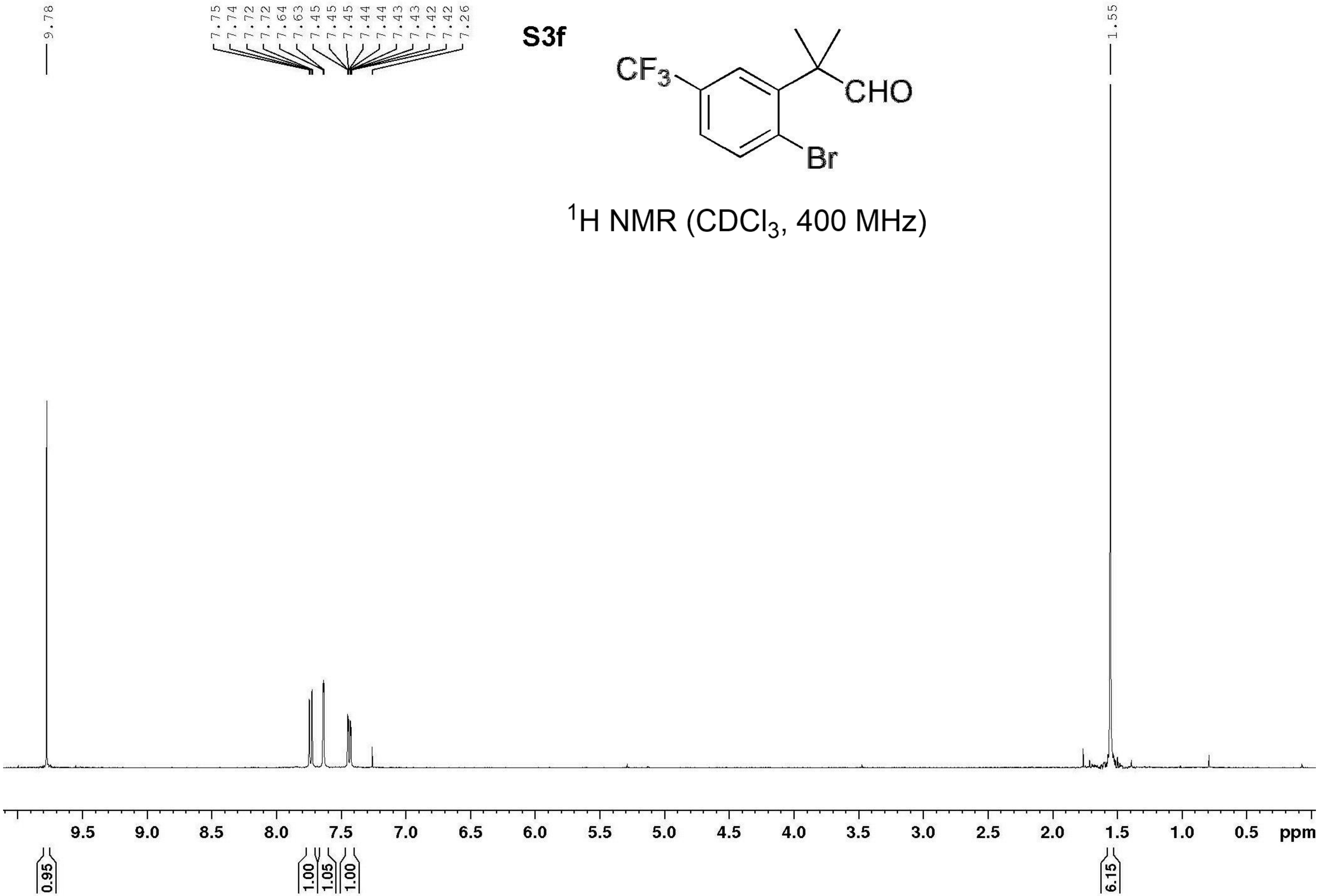

S3f
CF3
CHO
Br
1H NMR (CDCl3, 400 MHz)
9.78
7.75 7.74 7.72 7.72 7.64 7.63 7.45 7.45 7.45 7.44 7.44 7.43 7.43 7.42 7.42 7.26
1.55
9.5 9.0 8.5 8.0 7.5 7.0 6.5 6.0 5.5 5.0 4.5 4.0 3.5 3.0 2.5 2.0 1.5 1.0 0.5 ppm
0.95
1.00 1.05 1.00
6.15

S3f

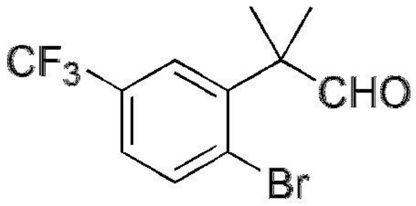

^{13}C NMR ($CDCl_3$, 100 MHz)

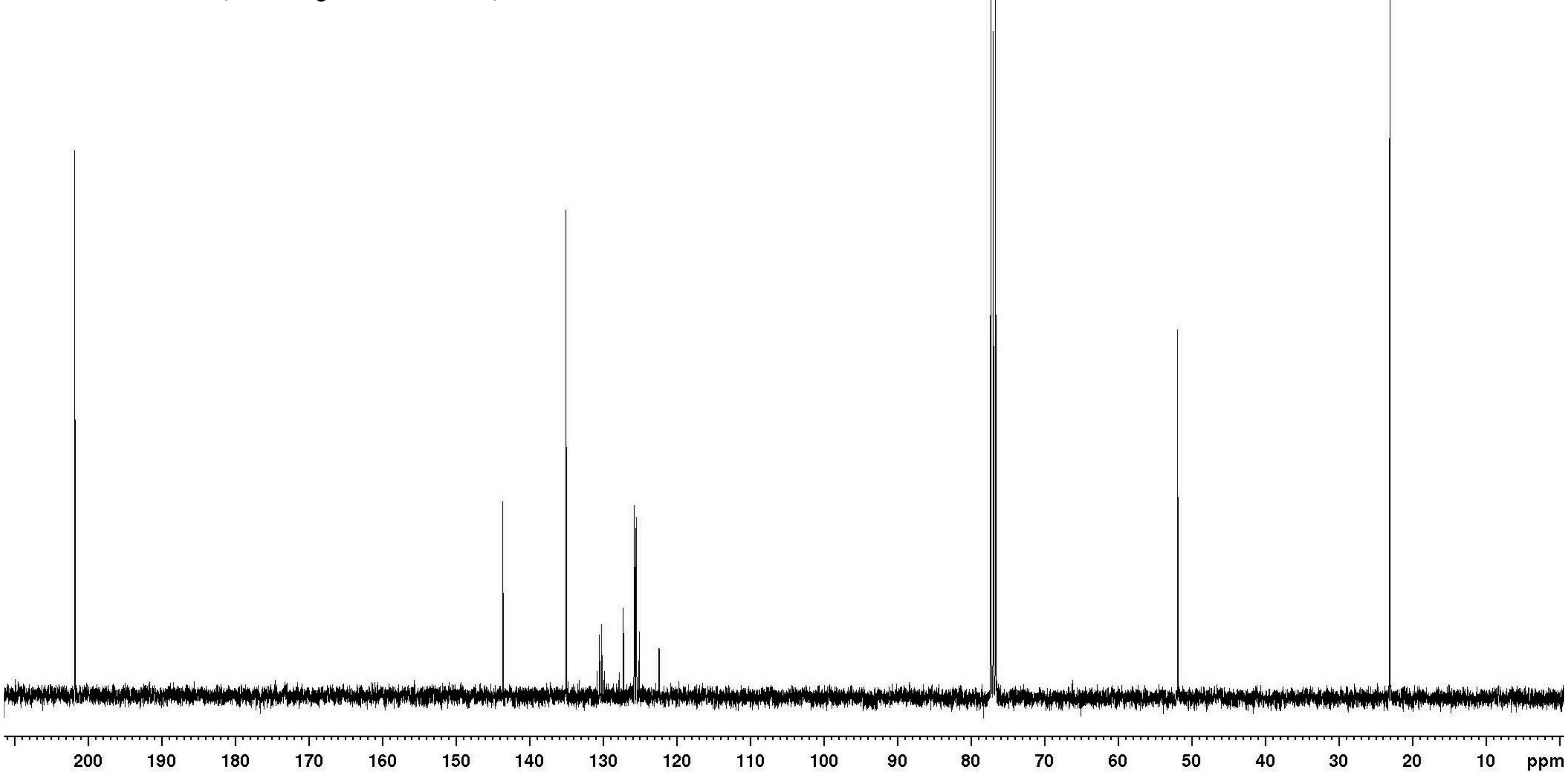

S3f

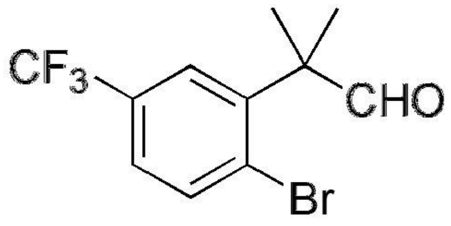

^{19}F NMR ($CDCl_3$, 376 MHz)

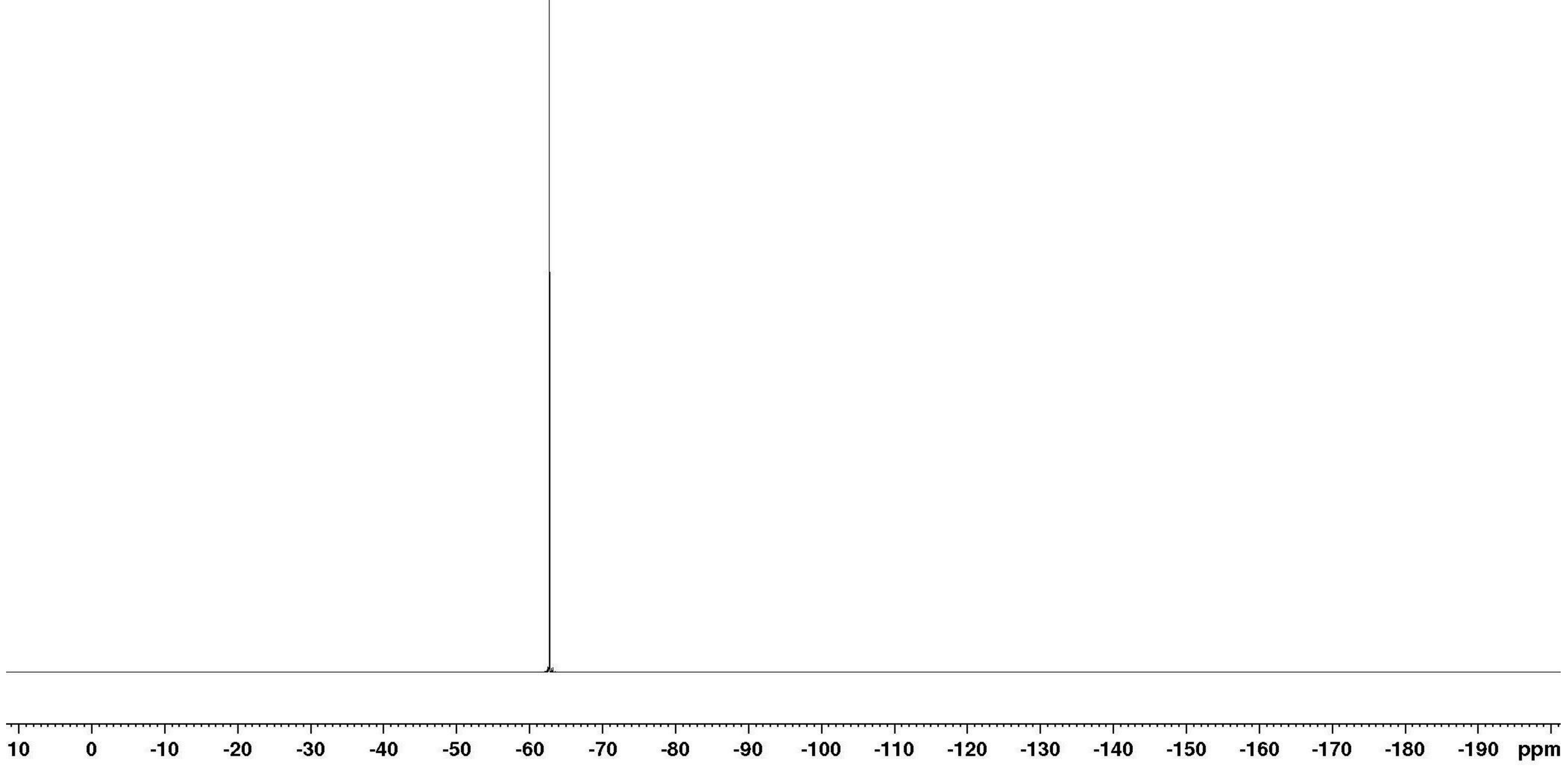

S3g

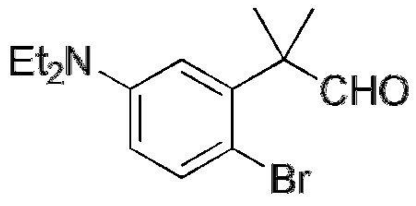

^{1}H NMR ($CDCl_3$, 400 MHz)

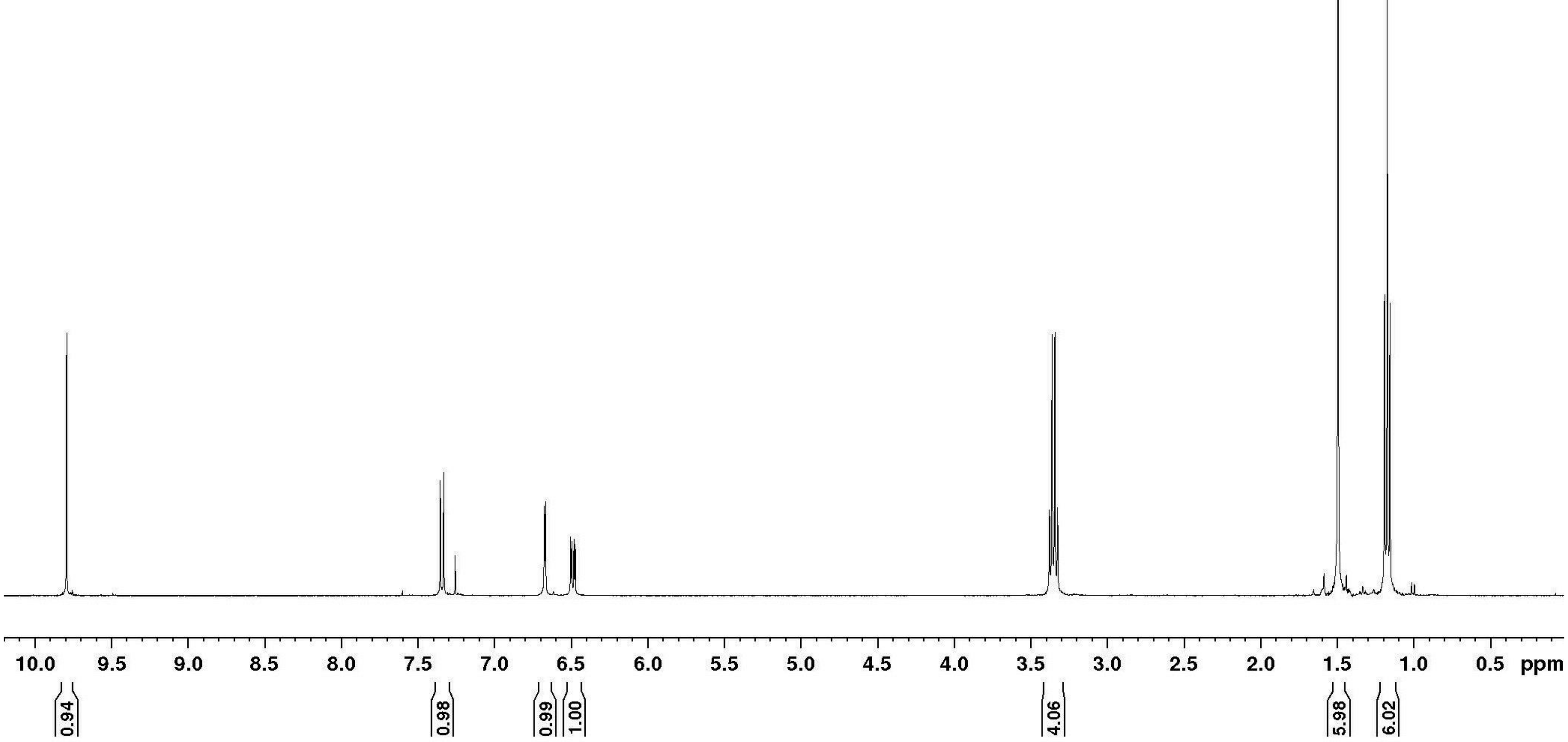

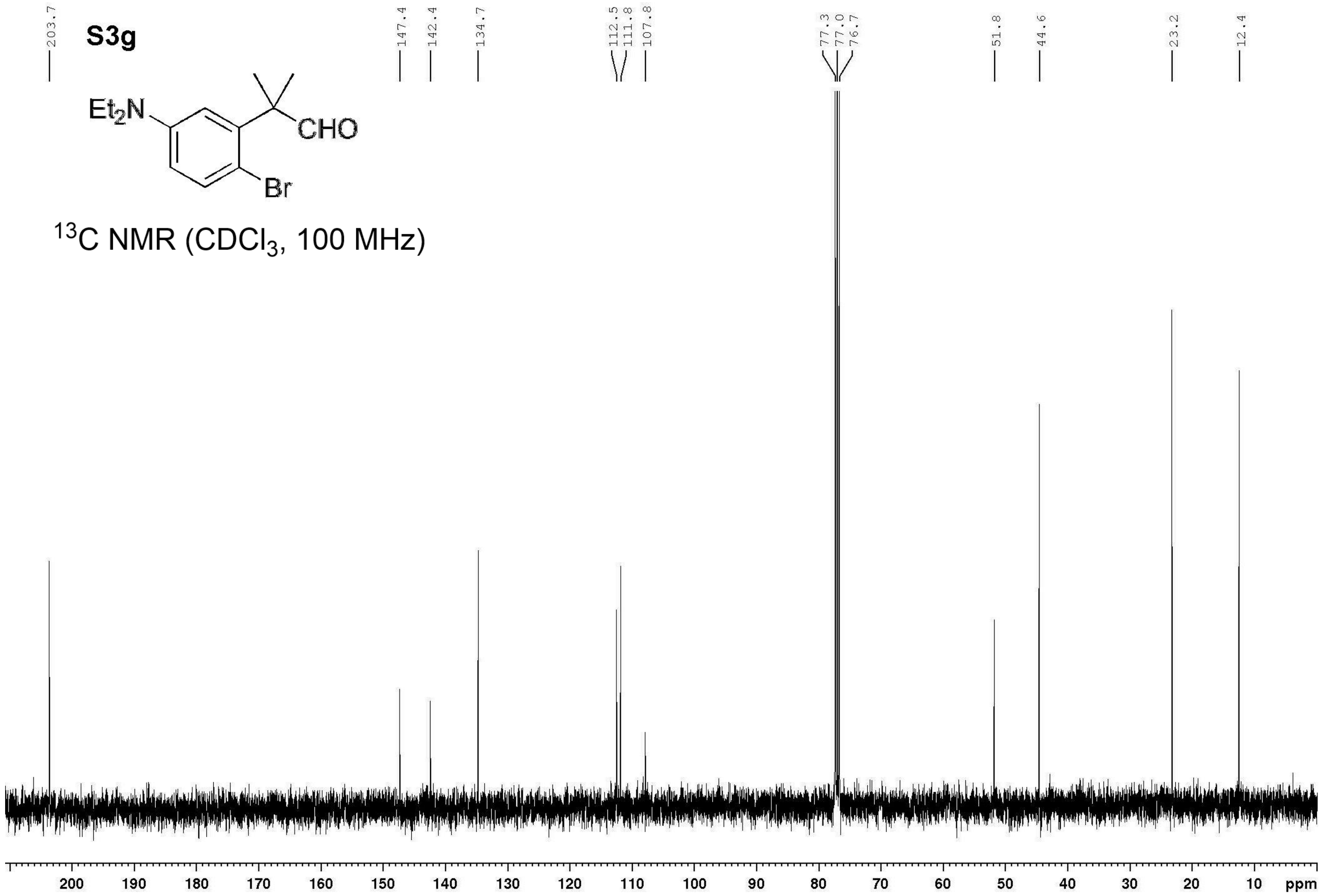
S3g
Et$_2$N
CHO
Br
^{13}C NMR (CDCl$_3$, 100 MHz)
203.7
147.4
142.4
134.7
112.5
111.8
107.8
77.3
77.0
76.7
51.8
44.6
23.2
12.4
200 190 180 170 160 150 140 130 120 110 100 90 80 70 60 50 40 30 20 10 ppm

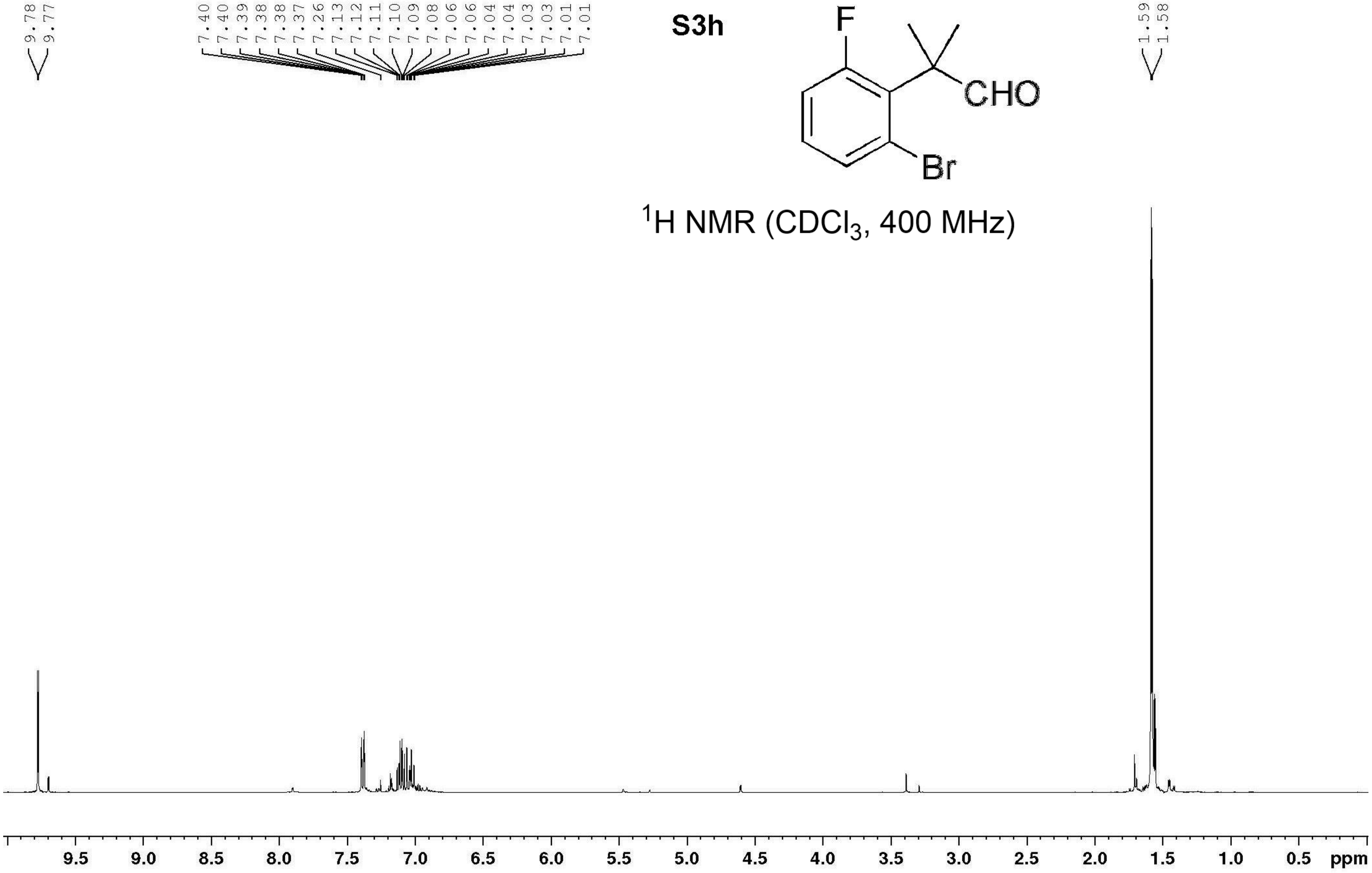
S3h
F
CHO
Br
^{1}H NMR ($CDCl_3$, 400 MHz)
9.78
9.77
7.40
7.40
7.39
7.38
7.38
7.37
7.26
7.13
7.12
7.11
7.10
7.09
7.08
7.06
7.06
7.04
7.04
7.03
7.03
7.01
7.01
1.59
1.58
9.5 9.0 8.5 8.0 7.5 7.0 6.5 6.0 5.5 5.0 4.5 4.0 3.5 3.0 2.5 2.0 1.5 1.0 0.5 ppm

S3h

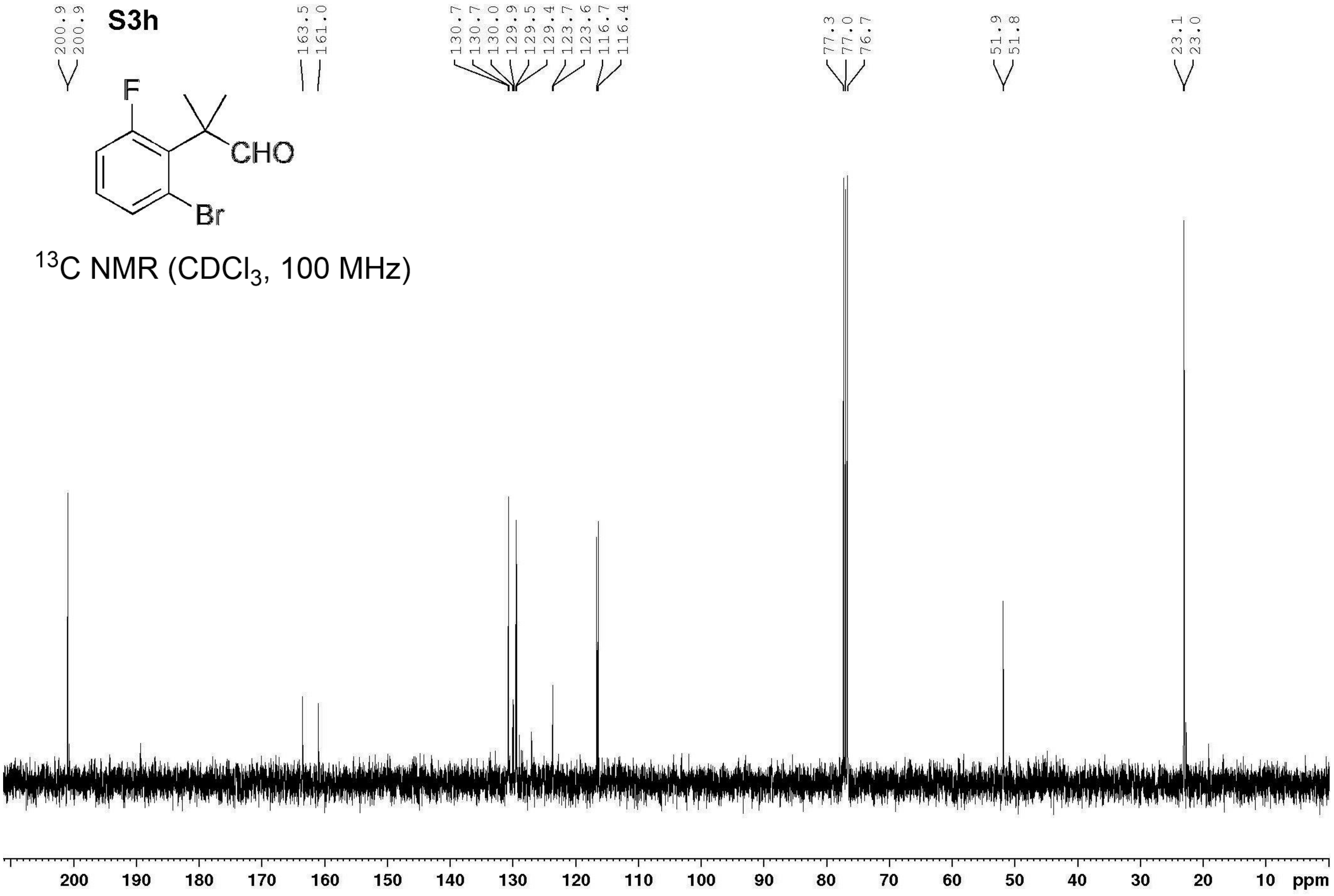

^{13}C NMR ($CDCl_3$, 100 MHz)

S3h

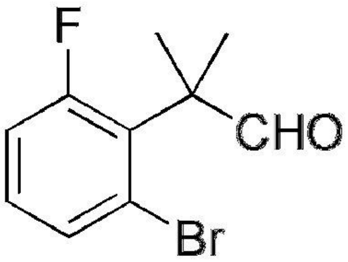

^{19}F NMR ($CDCl_3$, 376 MHz)

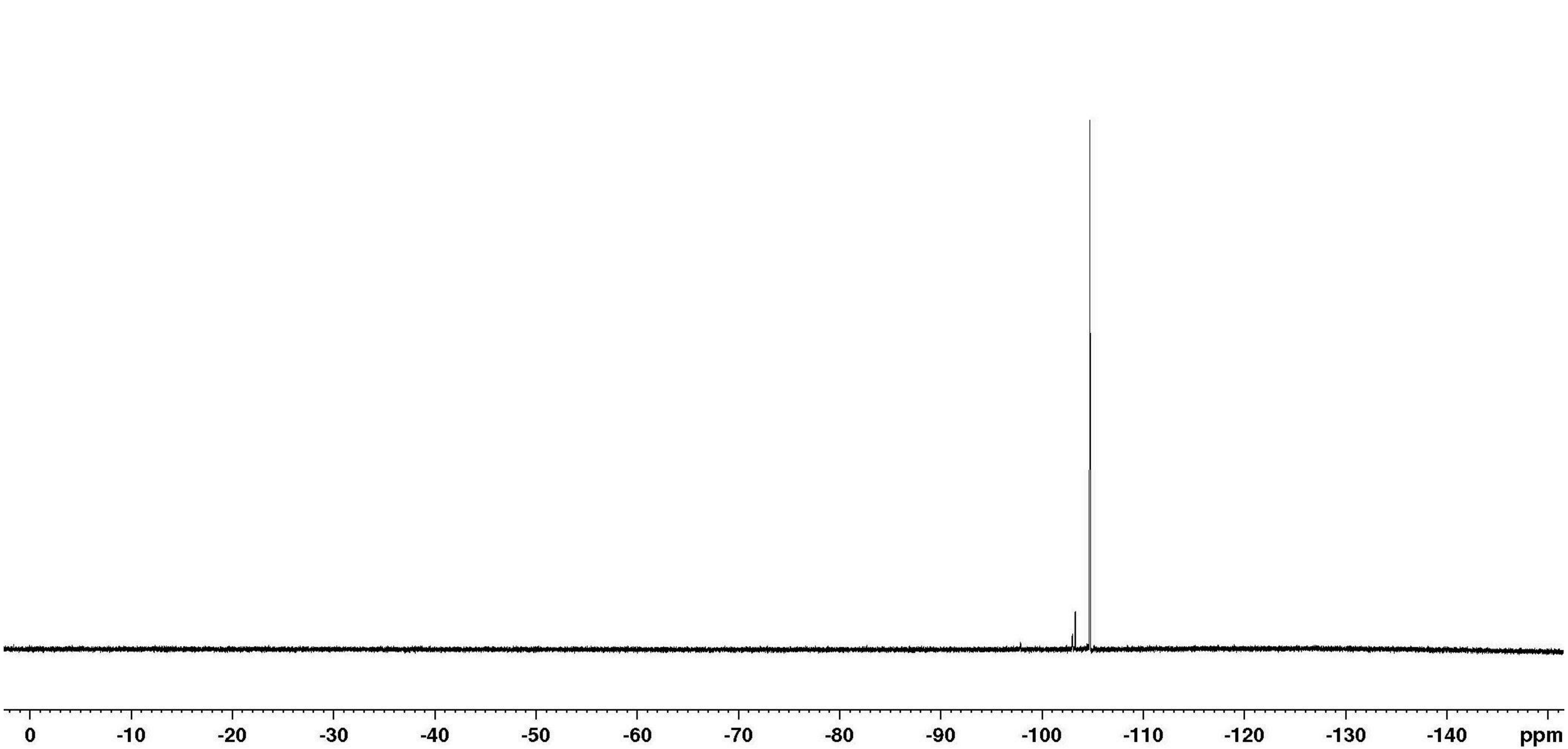

S3i

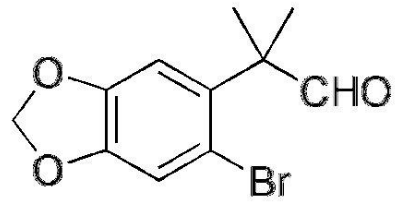

^1H NMR ($CDCl_3$, 400 MHz)

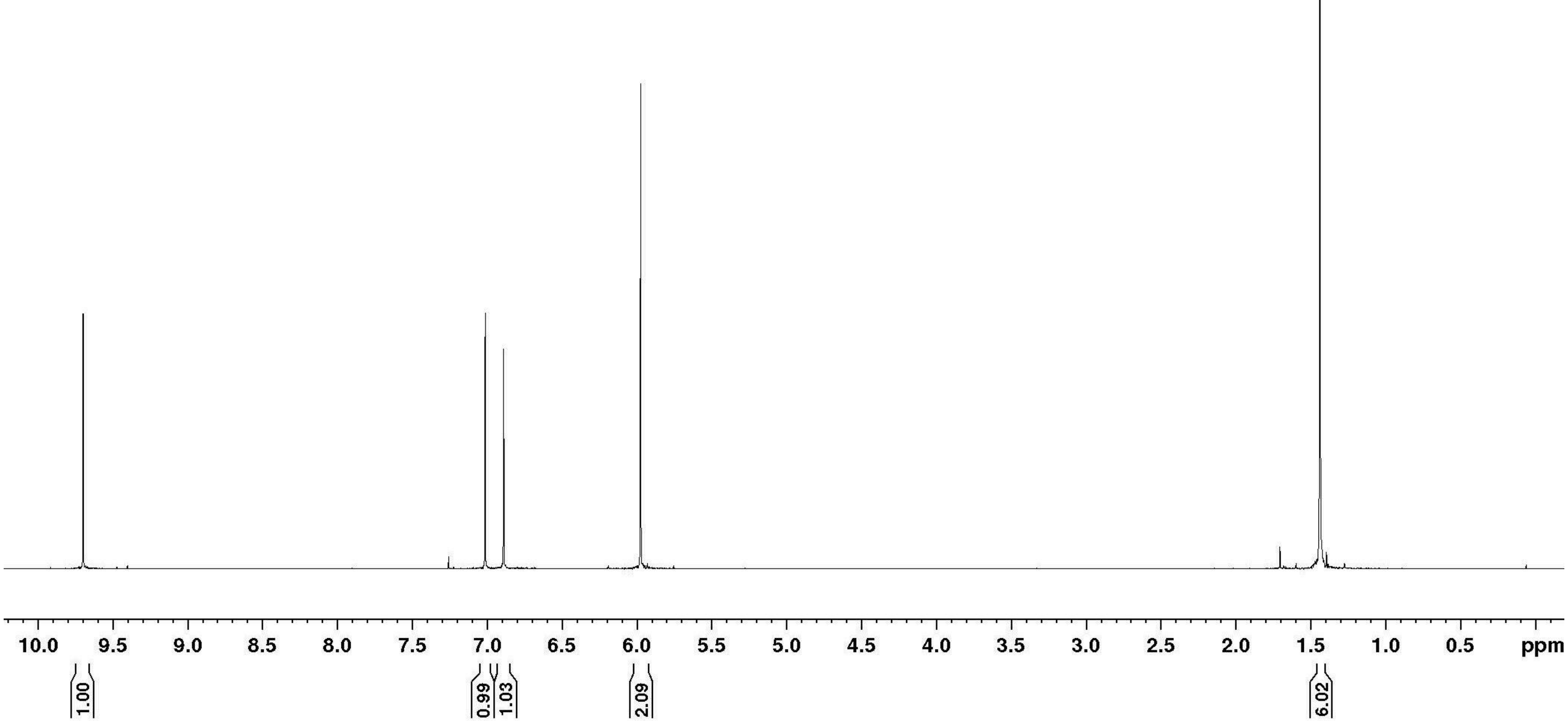

S3i

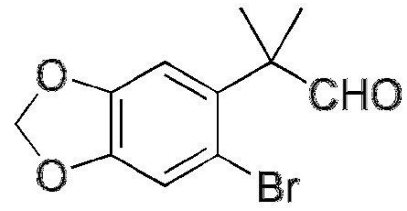

^{13}C NMR ($CDCl_3$, 100 MHz)

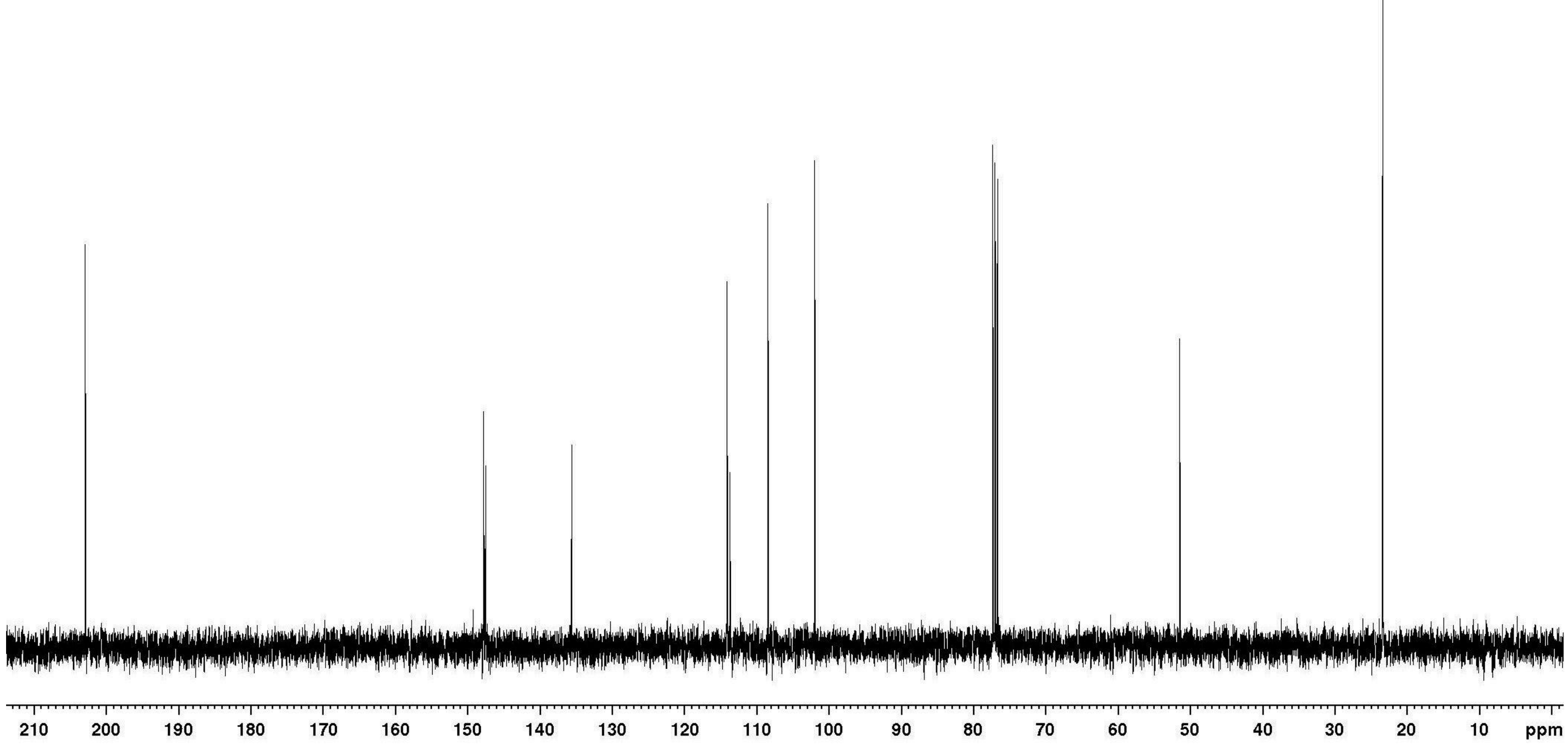

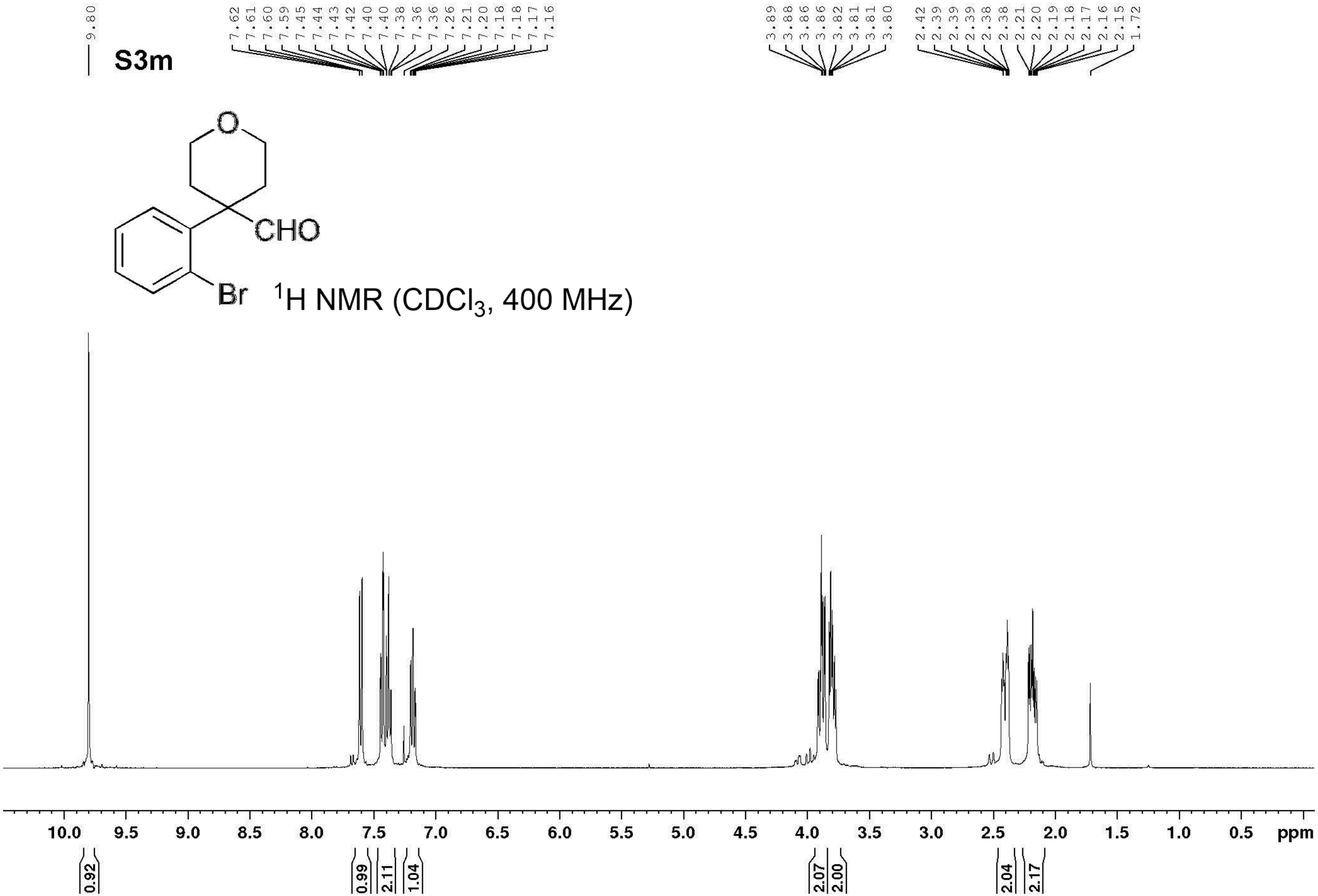
S3m
O
CHO
Br
1H NMR (CDCl3, 400 MHz)
9.80
7.62 7.61 7.60 7.59 7.45 7.44 7.43 7.42 7.40 7.40 7.38 7.36 7.36 7.26 7.21 7.20 7.18 7.18 7.17 7.16
3.89 3.88 3.86 3.86 3.82 3.81 3.81 3.80
2.42 2.39 2.39 2.39 2.38 2.38 2.21 2.20 2.19 2.18 2.17 2.16 2.15 1.72
10.0 9.5 9.0 8.5 8.0 7.5 7.0 6.5 6.0 5.5 5.0 4.5 4.0 3.5 3.0 2.5 2.0 1.5 1.0 0.5 ppm
0.92 0.99 2.11 1.04 2.07 2.00 2.04 2.17

S3m

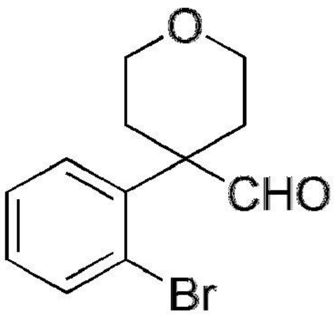

^{13}C NMR ($CDCl_3$, 100 MHz)

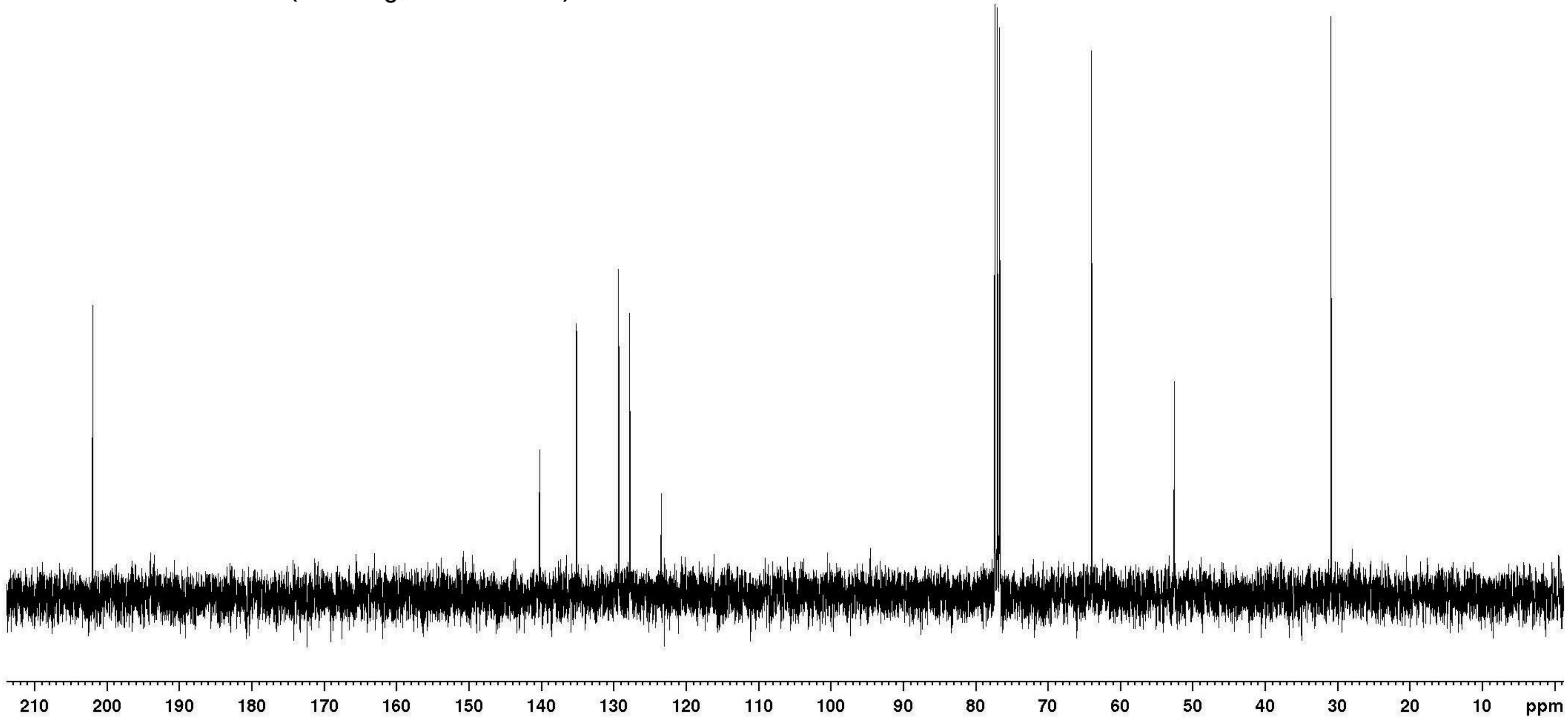

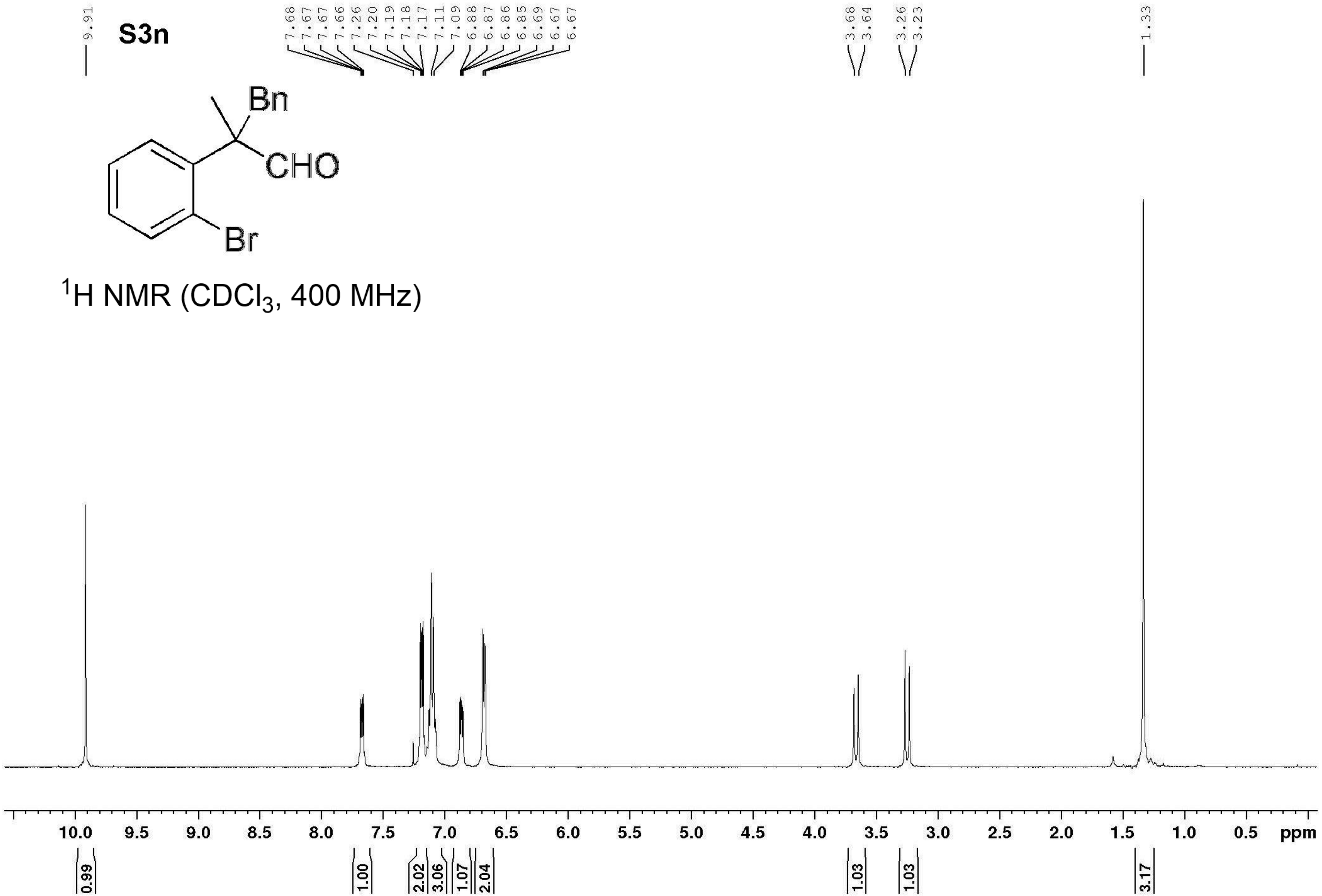
S3n
Bn
CHO
Br
^{1}H NMR (CDCl$_3$, 400 MHz)
9.91
7.68
7.67
7.67
7.66
7.26
7.20
7.19
7.18
7.17
7.11
7.09
6.88
6.87
6.86
6.85
6.69
6.67
6.67
3.68
3.64
3.26
3.23
1.33
10.0
9.5
9.0
8.5
8.0
7.5
7.0
6.5
6.0
5.5
5.0
4.5
4.0
3.5
3.0
2.5
2.0
1.5
1.0
0.5
ppm
0.99
1.00
2.02
3.06
1.07
2.04
1.03
1.03
3.17

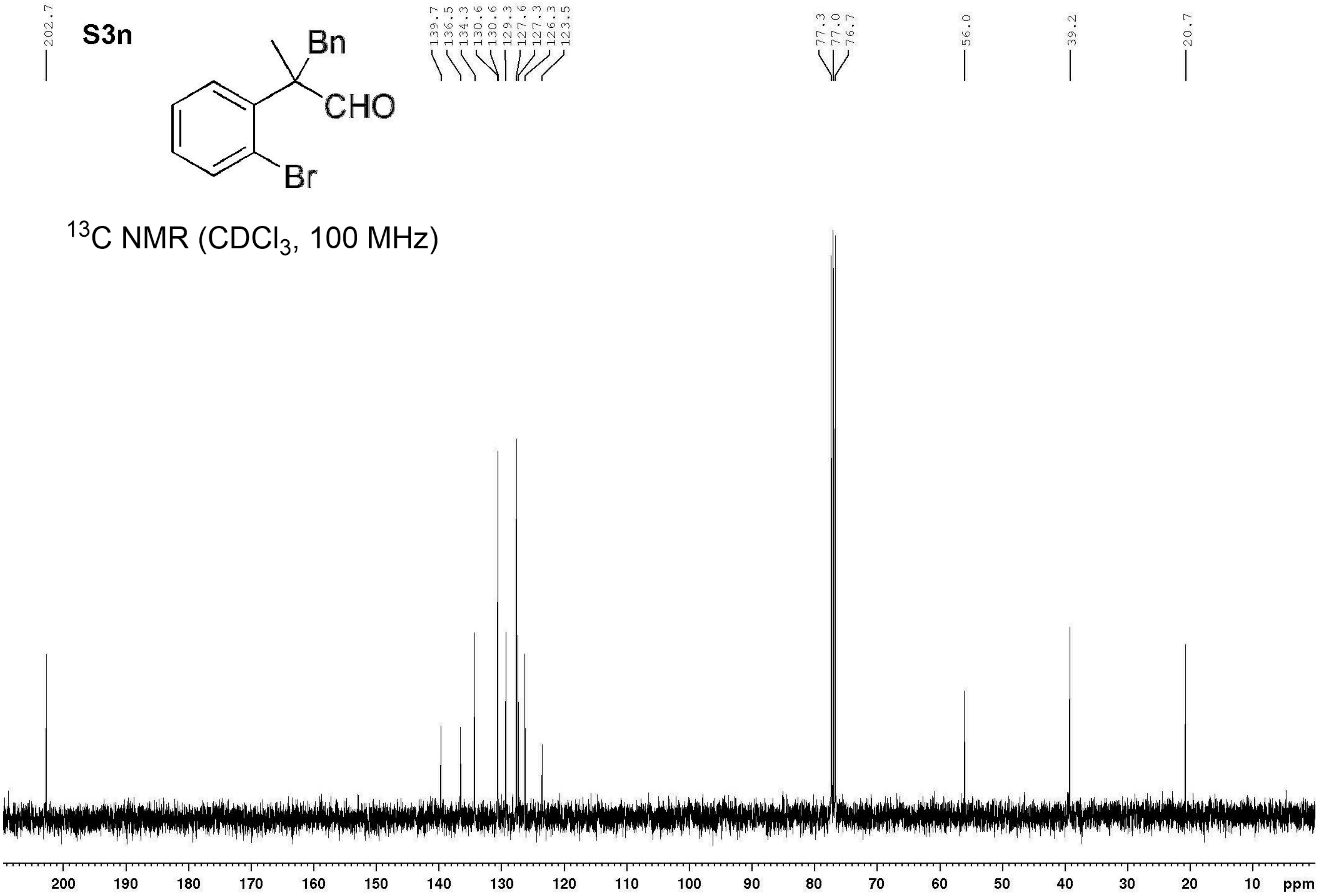
S3n
Bn
CHO
Br
^{13}C NMR ($CDCl_3$, 100 MHz)
202.7
139.7
136.5
134.3
130.6
130.6
129.3
127.6
127.3
126.3
123.5
77.3
77.0
76.7
56.0
39.2
20.7
200 190 180 170 160 150 140 130 120 110 100 90 80 70 60 50 40 30 20 10 ppm

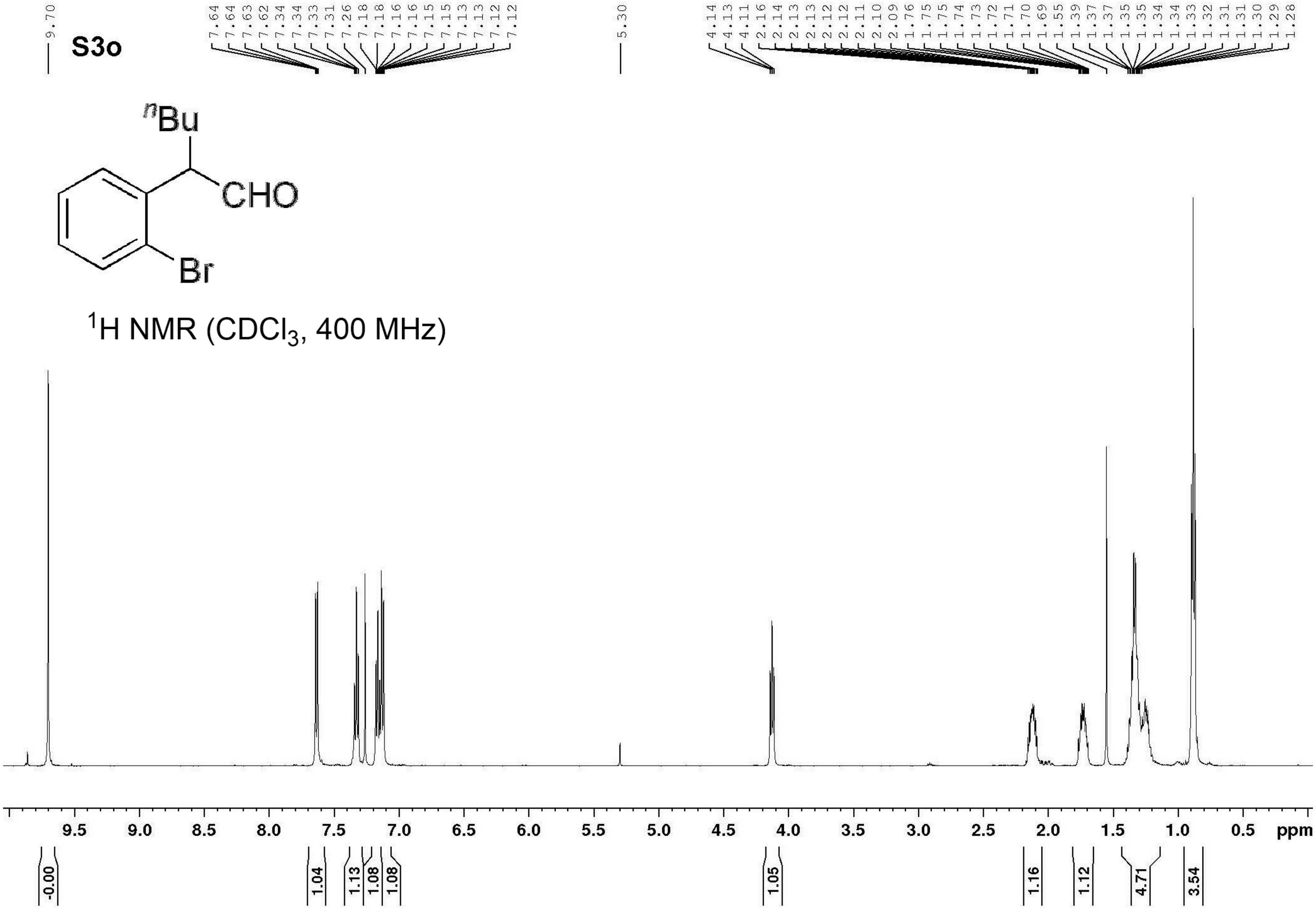
S3o
nBu
CHO
Br
1H NMR (CDCl3, 400 MHz)
9.70
7.64 7.64 7.63 7.62 7.34 7.34 7.33 7.31 7.26 7.18 7.18 7.16 7.16 7.15 7.15 7.13 7.13 7.12 7.12
5.30
4.14 4.13 4.11 2.16 2.14 2.13 2.13 2.12 2.12 2.11 2.10 2.09 1.76 1.75 1.75 1.74 1.73 1.72 1.71 1.70 1.69 1.55 1.39 1.37 1.37 1.35 1.35 1.34 1.34 1.33 1.32 1.31 1.31 1.30 1.29 1.28
9.5 9.0 8.5 8.0 7.5 7.0 6.5 6.0 5.5 5.0 4.5 4.0 3.5 3.0 2.5 2.0 1.5 1.0 0.5 ppm
-0.00 1.04 1.13 1.08 1.08 1.05 1.16 1.12 4.71 3.54

S3o

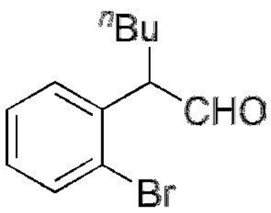

^{13}C NMR ($CDCl_3$, 100 MHz)

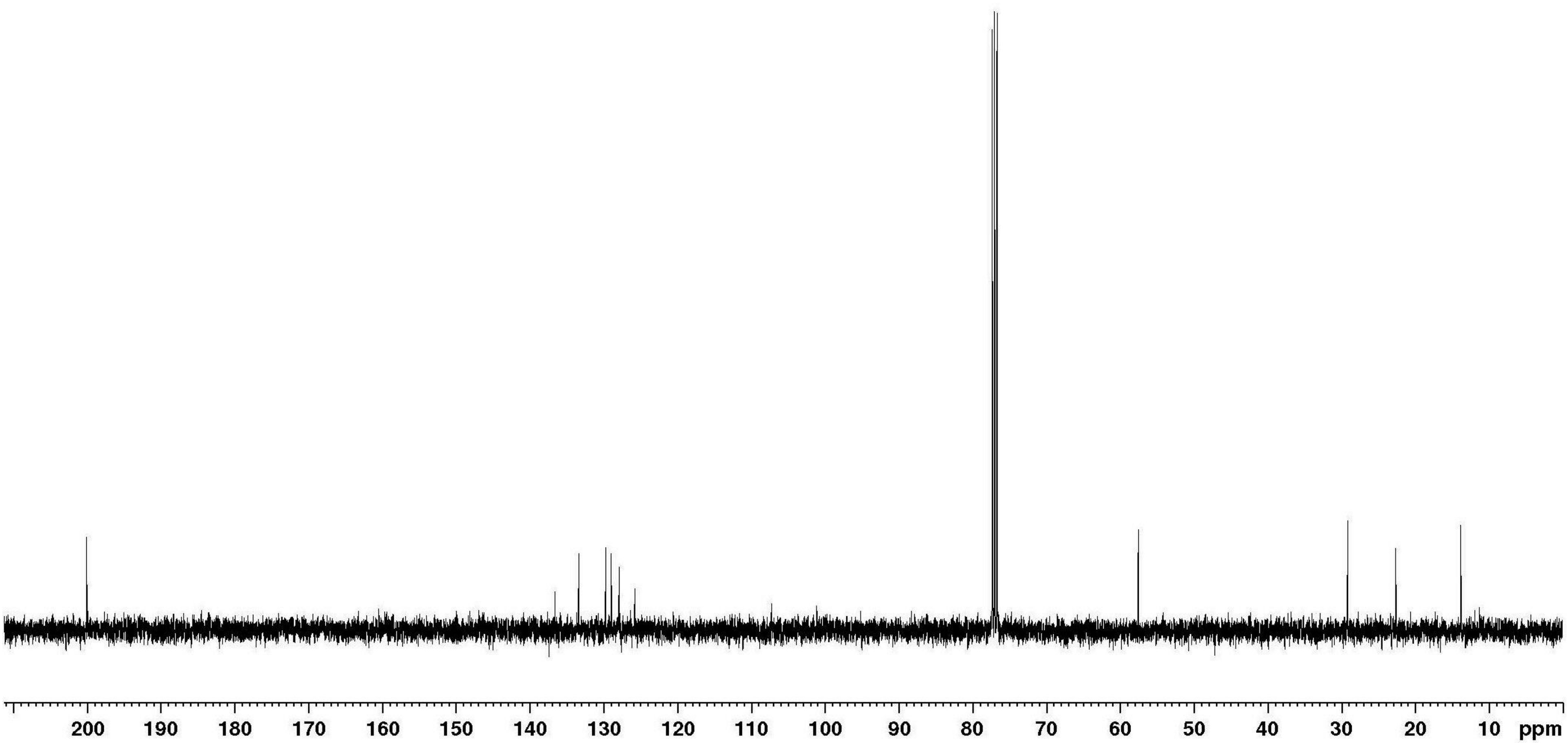

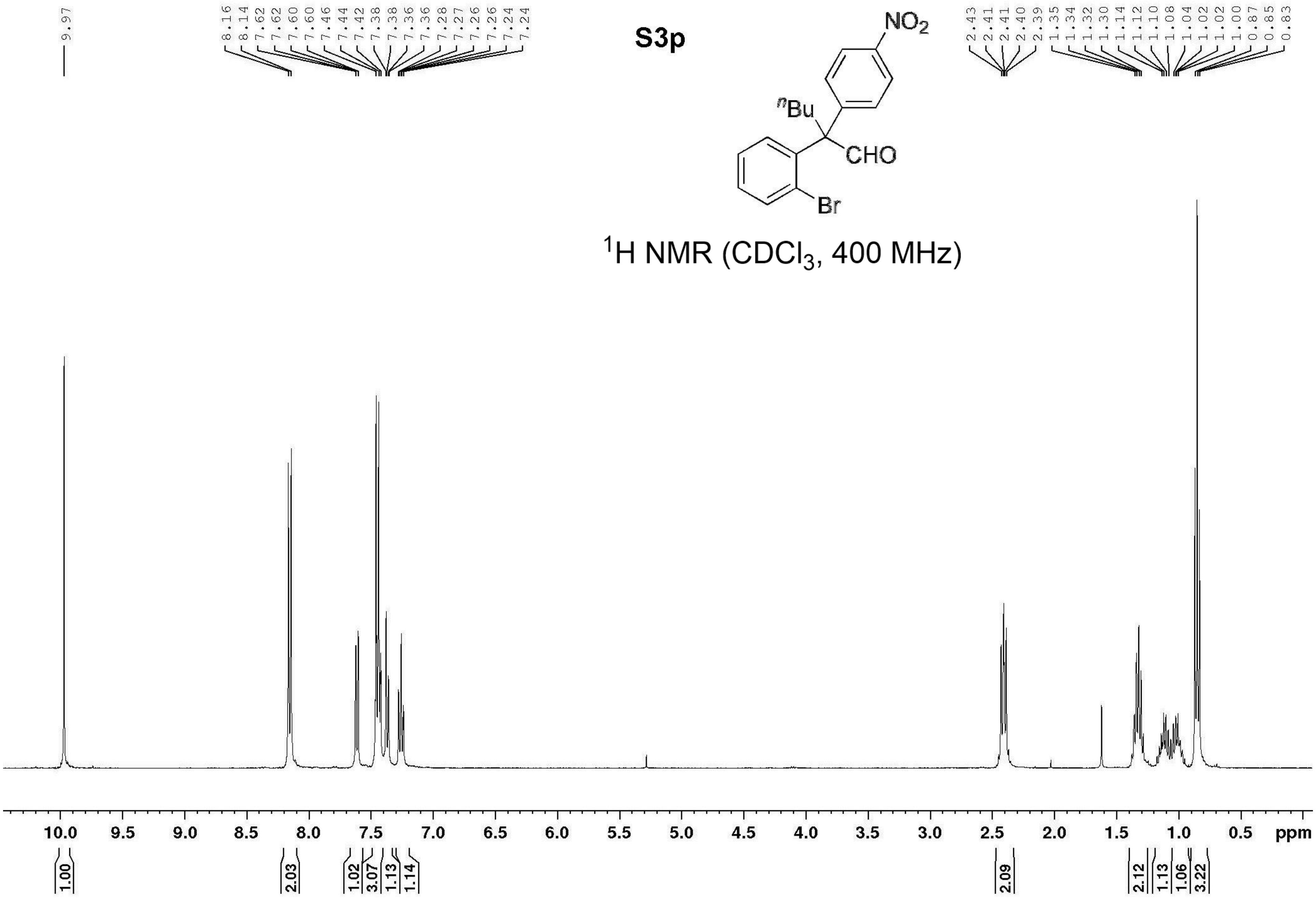
S3p
NO2
nBu
CHO
Br
1H NMR (CDCl3, 400 MHz)
9.97
8.16 8.14 7.62 7.62 7.60 7.60 7.46 7.44 7.42 7.38 7.38 7.36 7.36 7.28 7.27 7.26 7.26 7.24 7.24
2.43 2.41 2.41 2.40 2.39 1.35 1.34 1.32 1.30 1.14 1.12 1.10 1.08 1.04 1.02 1.00 0.87 0.85 0.83
10.0 9.5 9.0 8.5 8.0 7.5 7.0 6.5 6.0 5.5 5.0 4.5 4.0 3.5 3.0 2.5 2.0 1.5 1.0 0.5 ppm
1.00
2.03
1.02
3.07
1.13
1.14
2.09
2.12
1.13
1.06
3.22

S3p

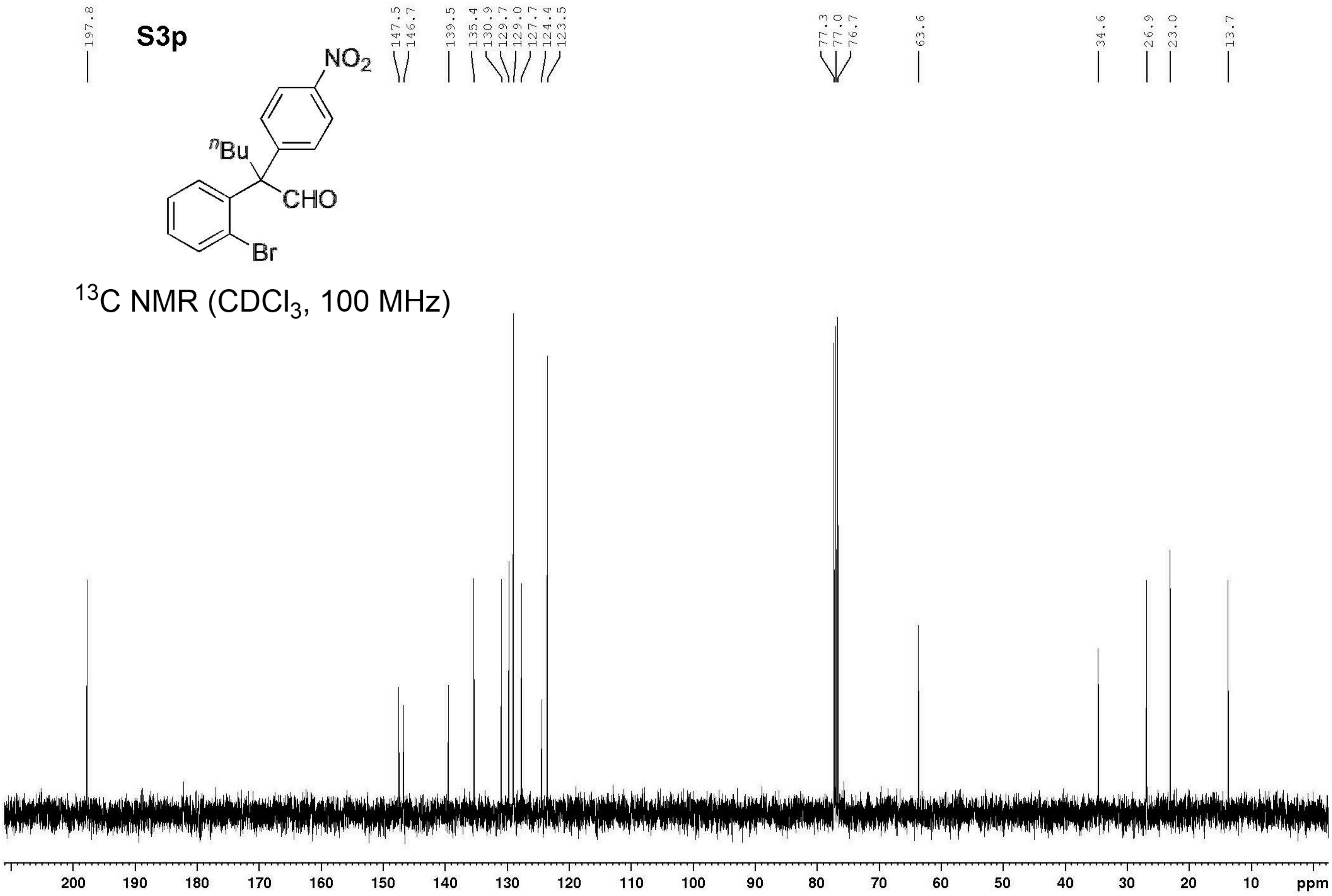

^{13}C NMR ($CDCl_3$, 100 MHz)

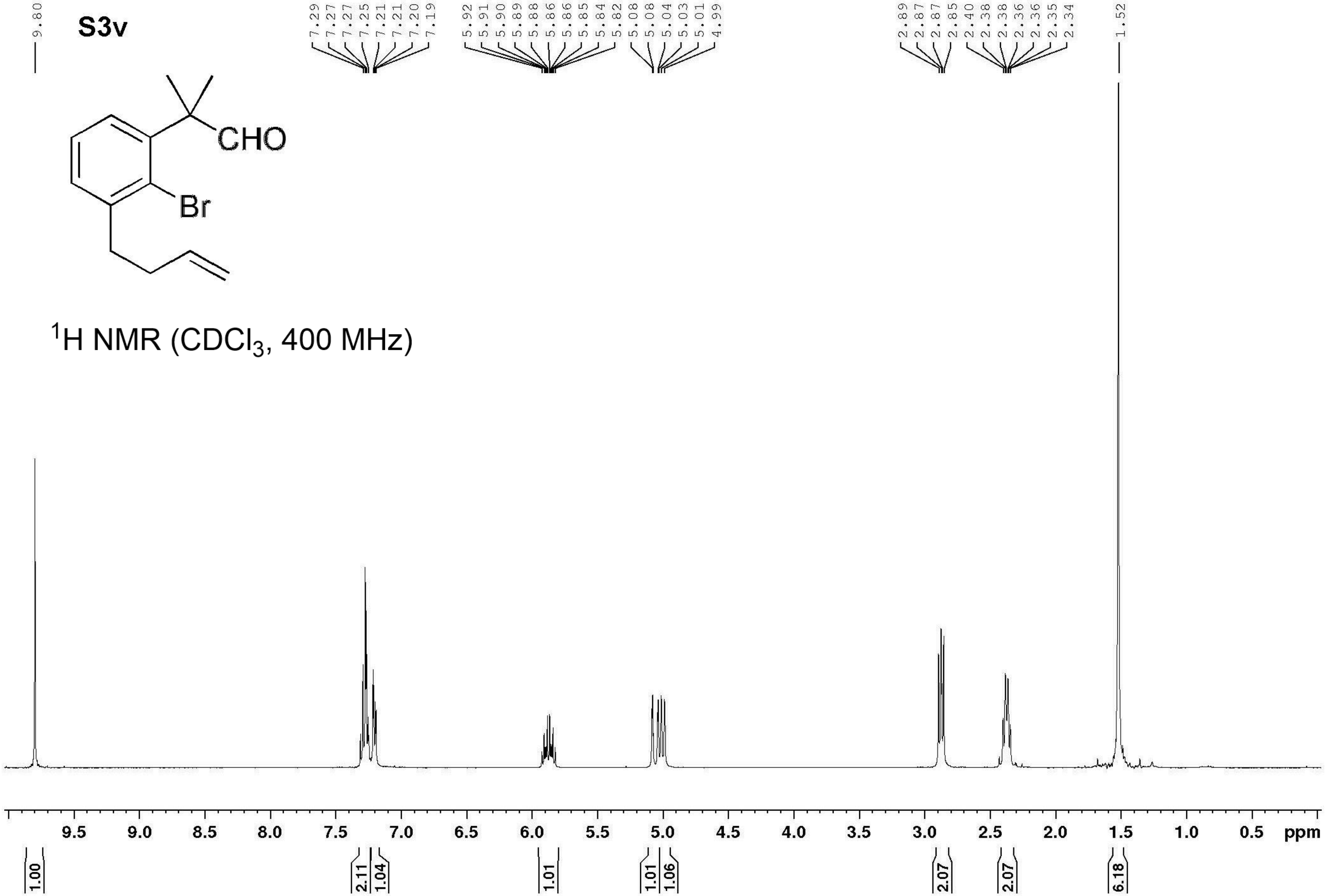
S3v
CHO
Br
^{1}H NMR ($CDCl_3$, 400 MHz)
9.80
7.29 7.27 7.27 7.25 7.21 7.21 7.20 7.19
5.92 5.91 5.90 5.89 5.88 5.86 5.86 5.85 5.84 5.82
5.08 5.08 5.04 5.03 5.01 4.99
2.89 2.87 2.87 2.85
2.40 2.38 2.38 2.36 2.36 2.35 2.34
1.52
9.5 9.0 8.5 8.0 7.5 7.0 6.5 6.0 5.5 5.0 4.5 4.0 3.5 3.0 2.5 2.0 1.5 1.0 0.5 ppm
1.00 2.11 1.04 1.01 1.01 1.06 2.07 2.07 6.18

S3v

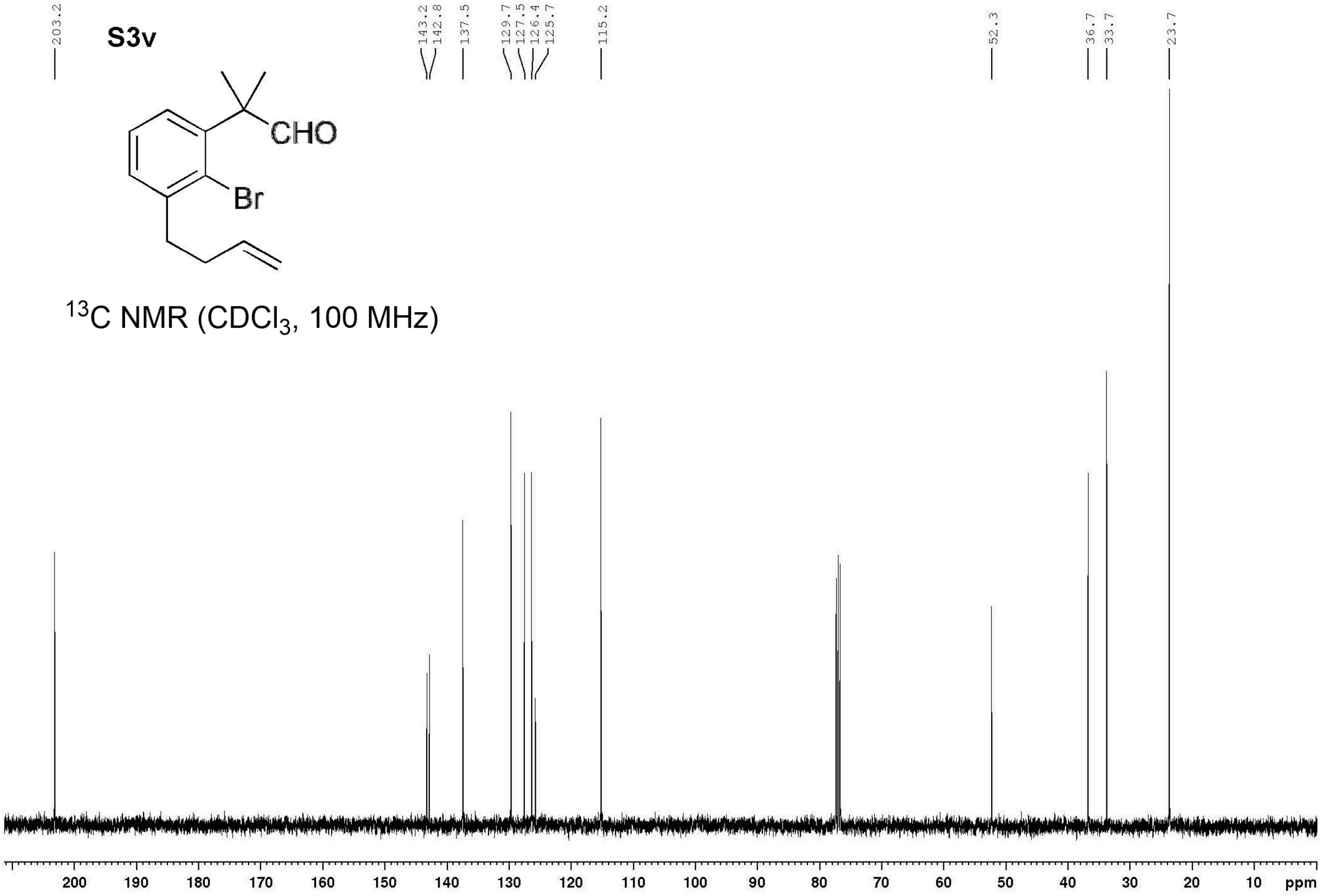

^{13}C NMR ($CDCl_3$, 100 MHz)

S4

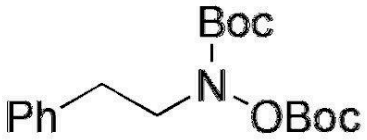

^1H NMR ($CDCl_3$, 400 MHz)

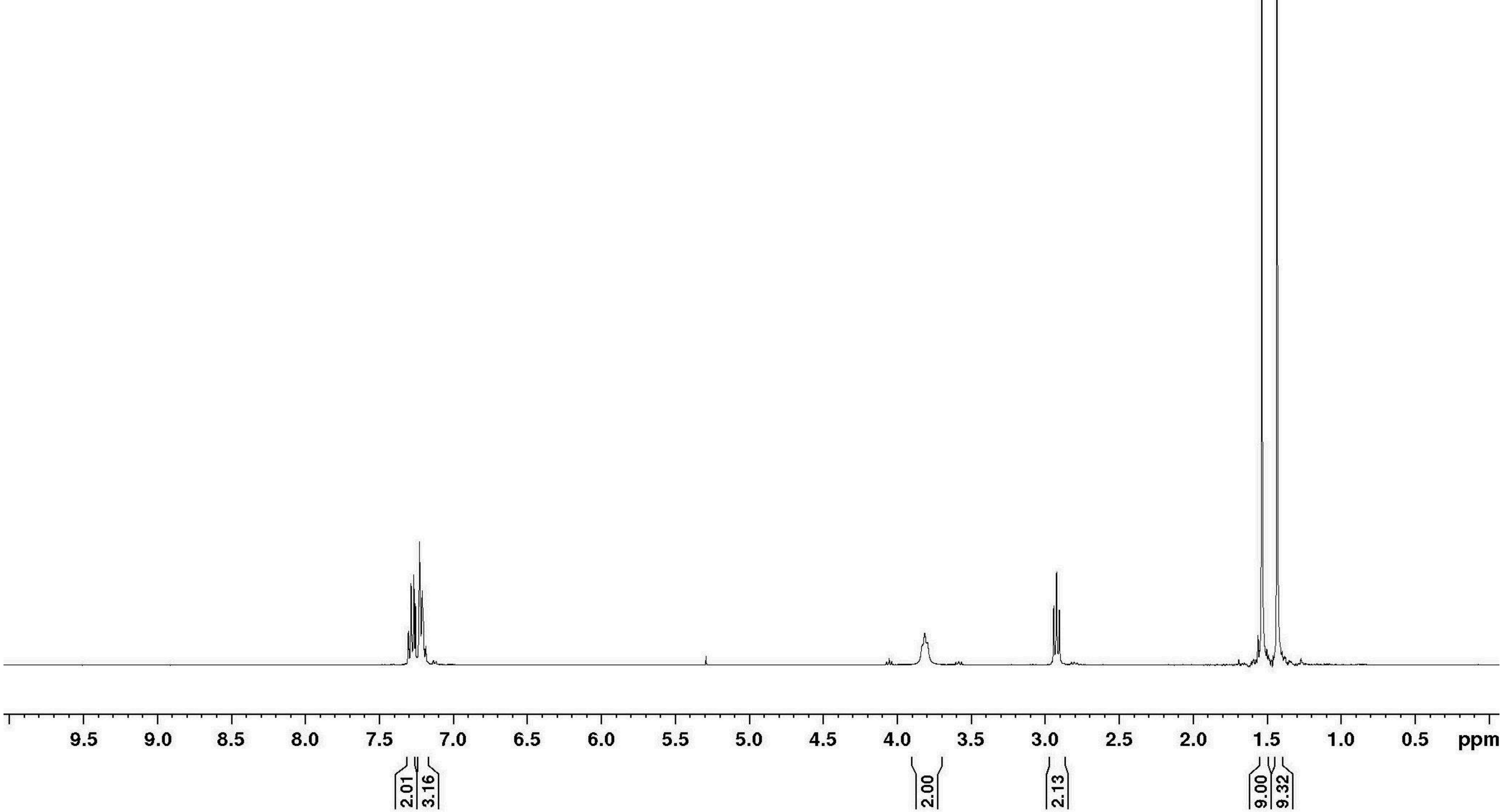

S4

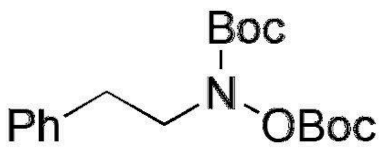

^{13}C NMR ($CDCl_3$, 100 MHz)

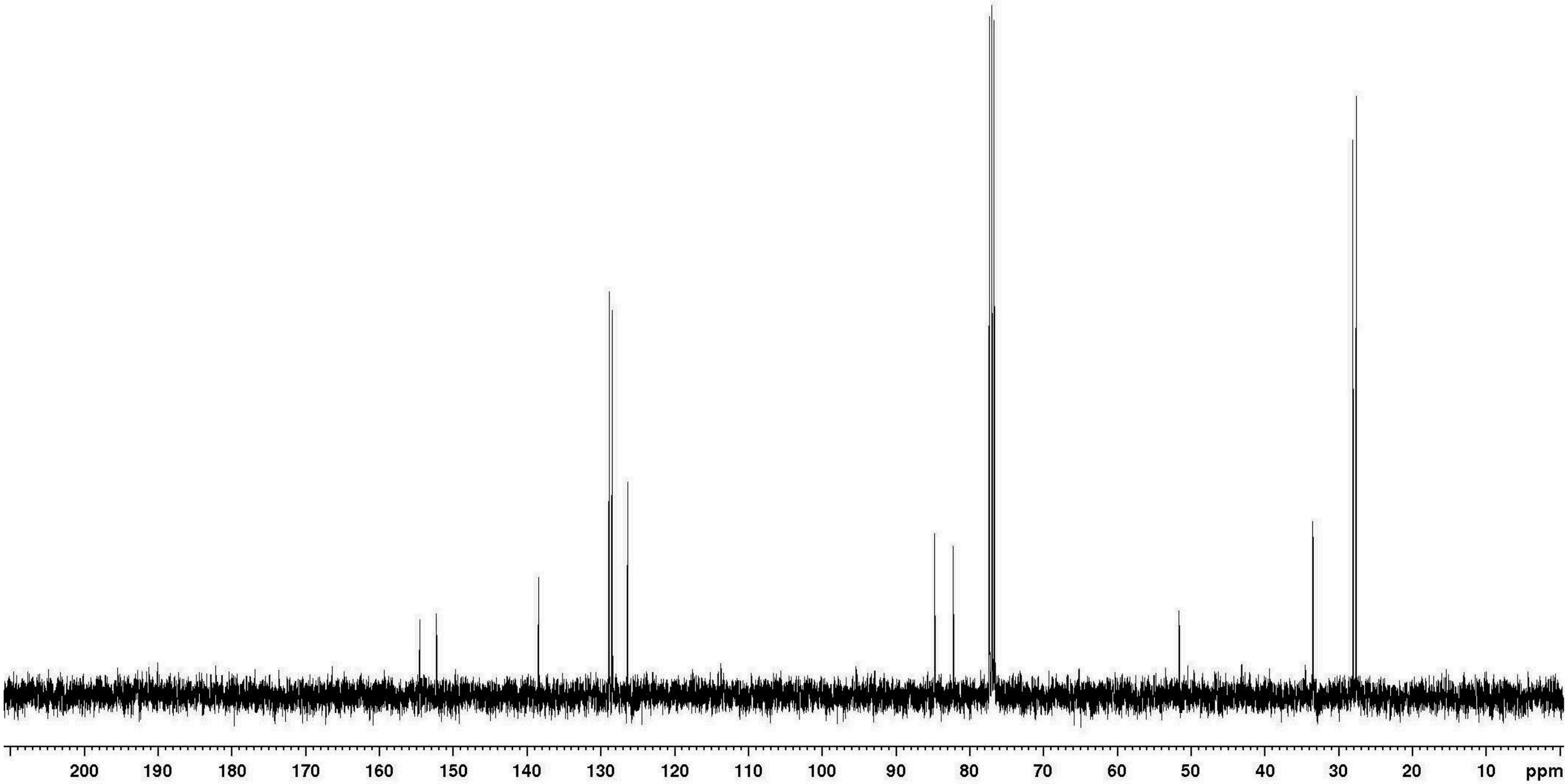

S5

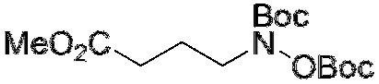

^{1}H NMR ($CDCl_3$, 400 MHz)

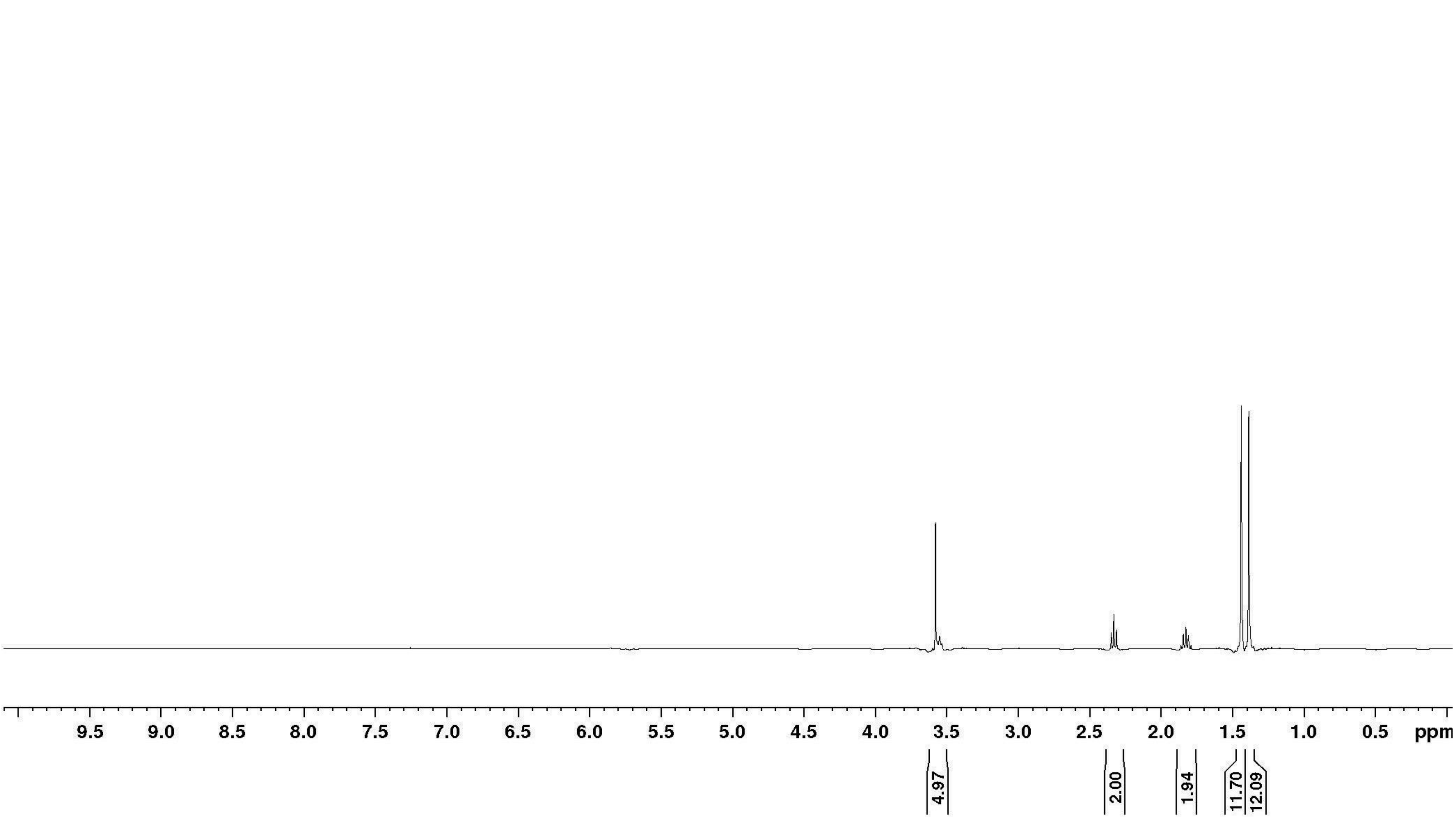

S5

^{13}C NMR ($CDCl_3$, 100 MHz)

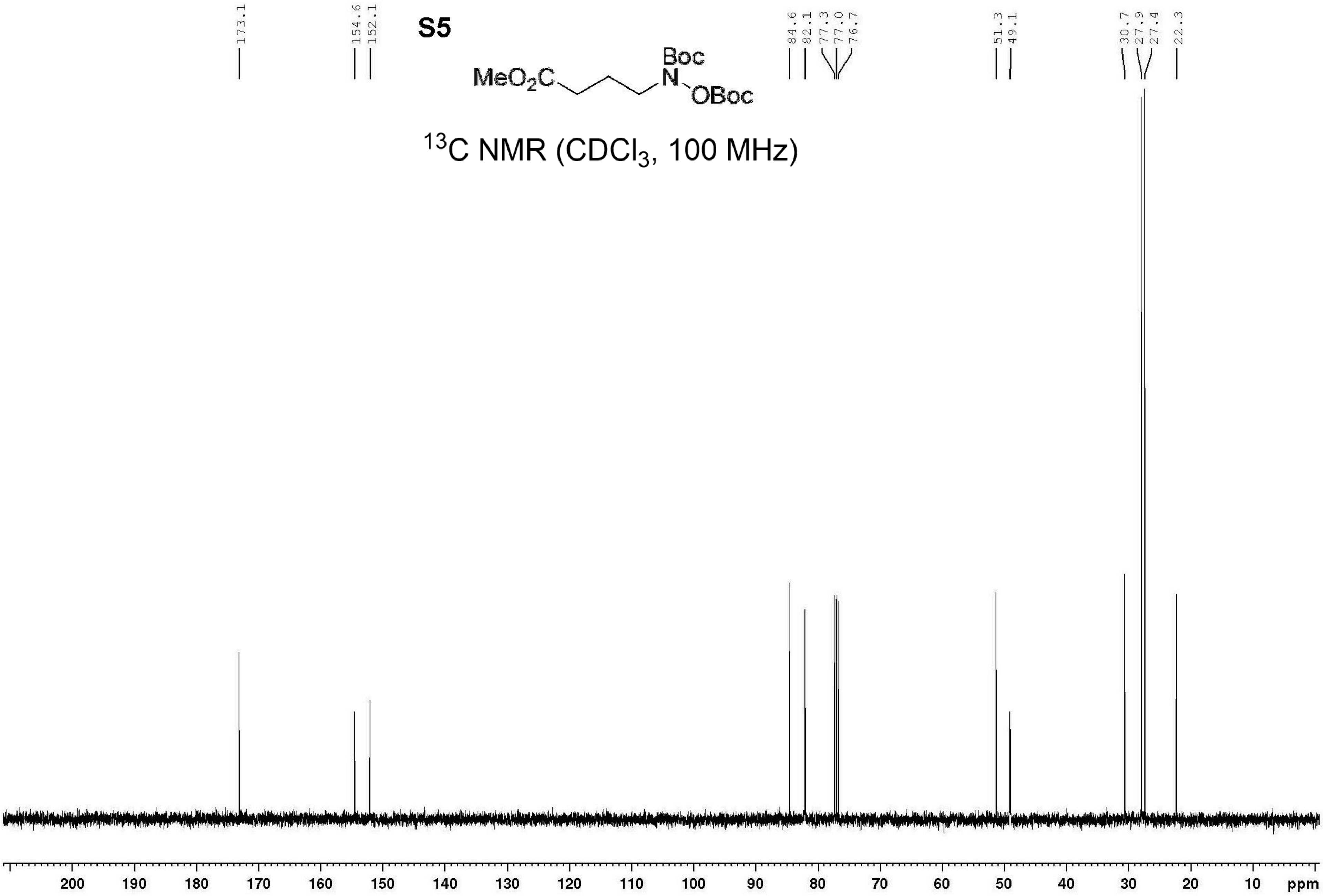

S6

^{1}H NMR ($CDCl_3$, 400 MHz)

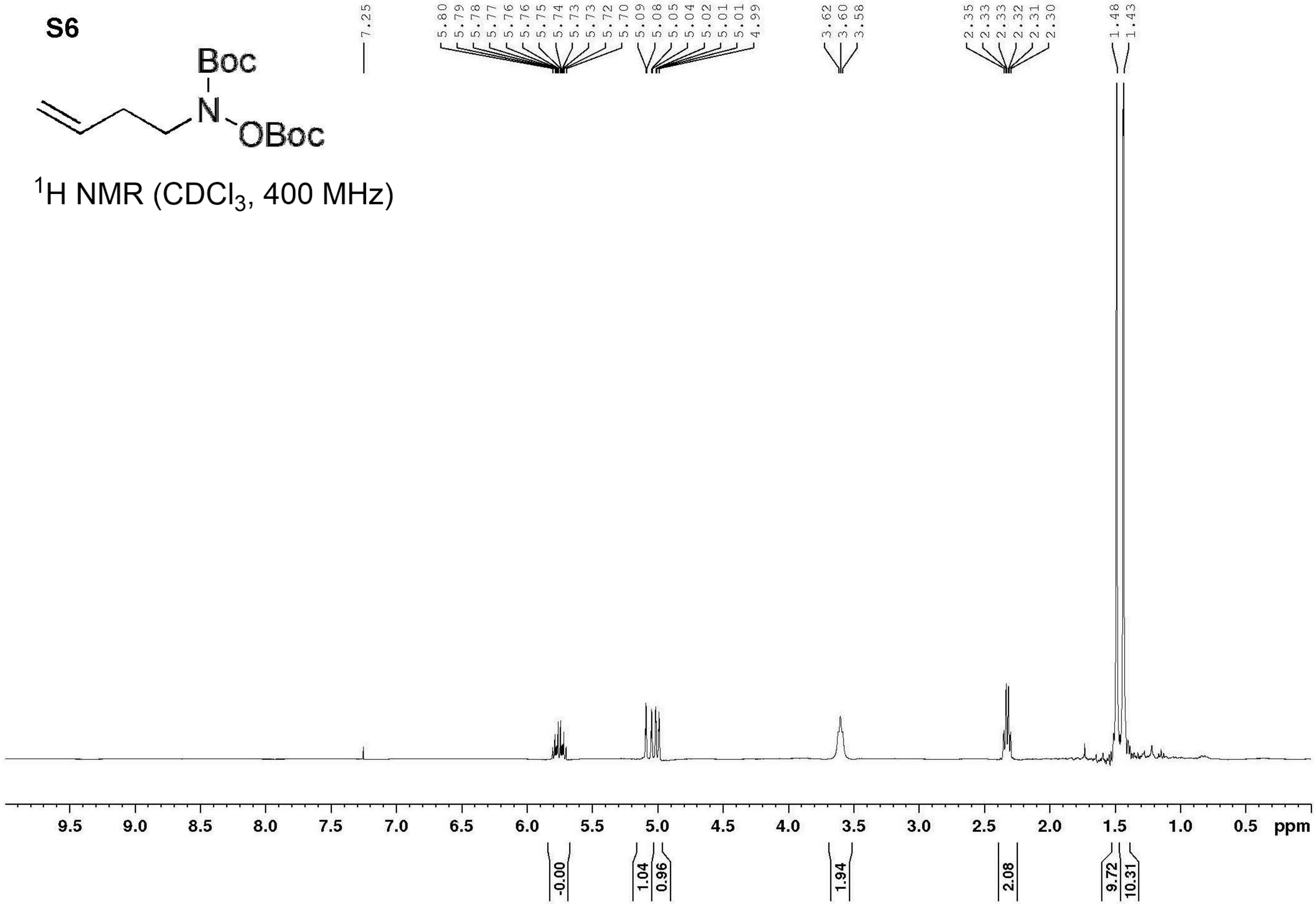

S6

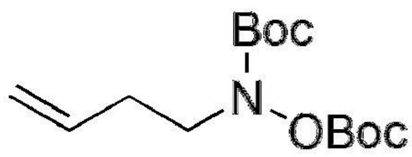

^{13}C NMR ($CDCl_3$, 100 MHz)

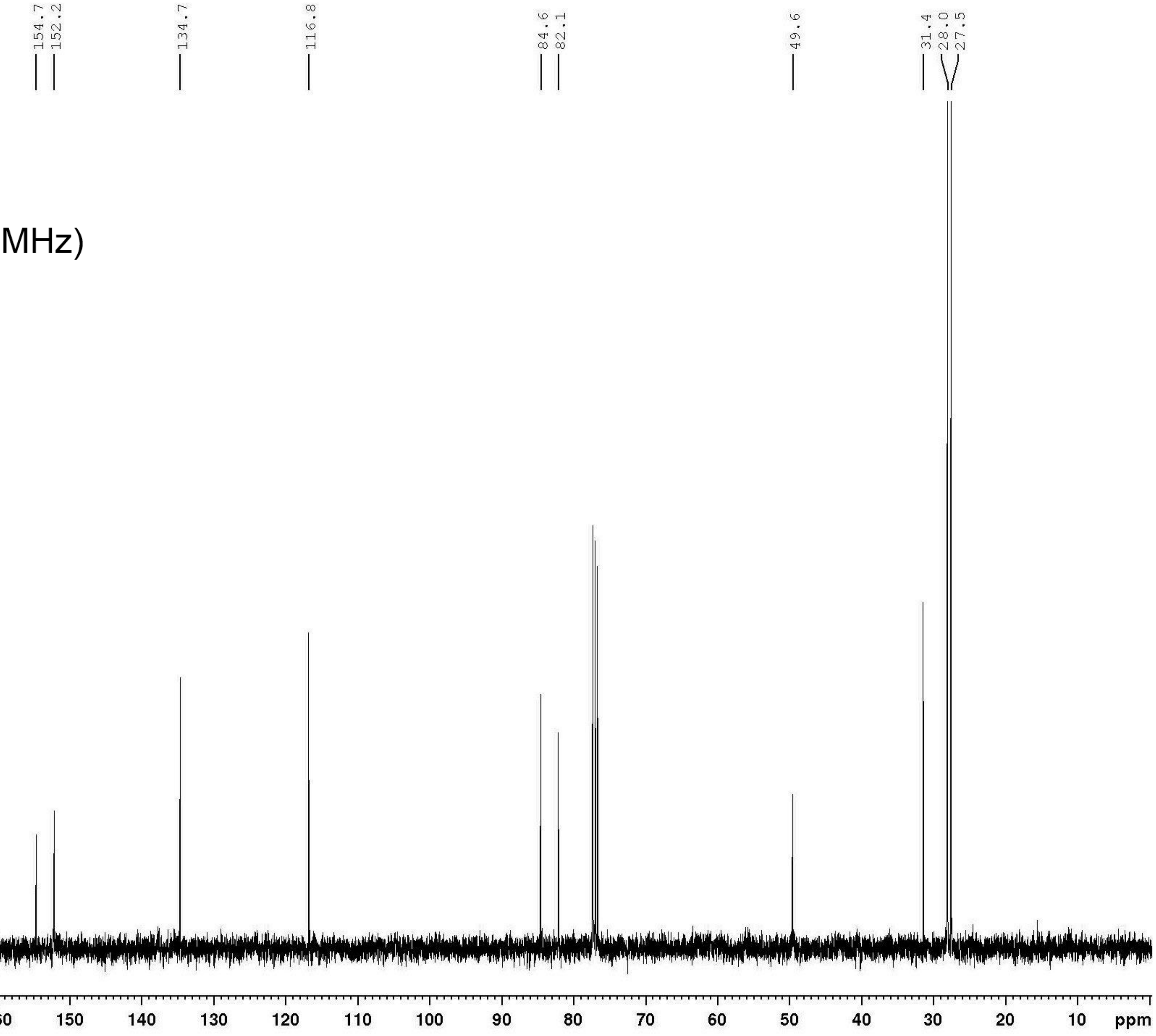

S7

90 atom% D

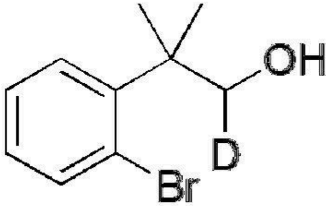

^{1}H NMR ($CDCl_3$, 400 MHz)

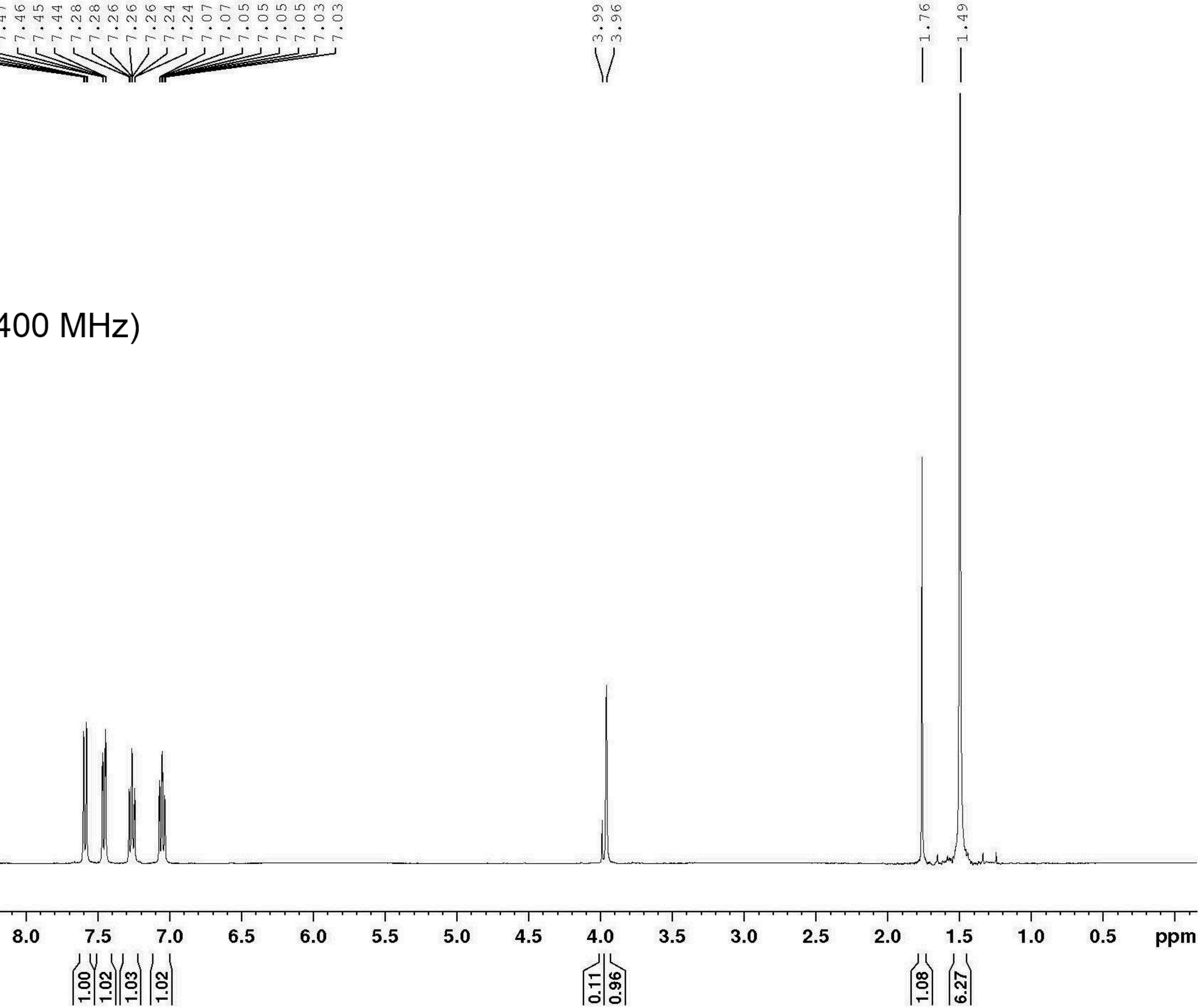

S7

90 atom% D

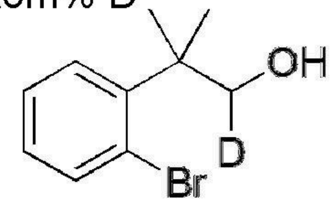

^{13}C NMR ($CDCl_3$, 100 MHz)

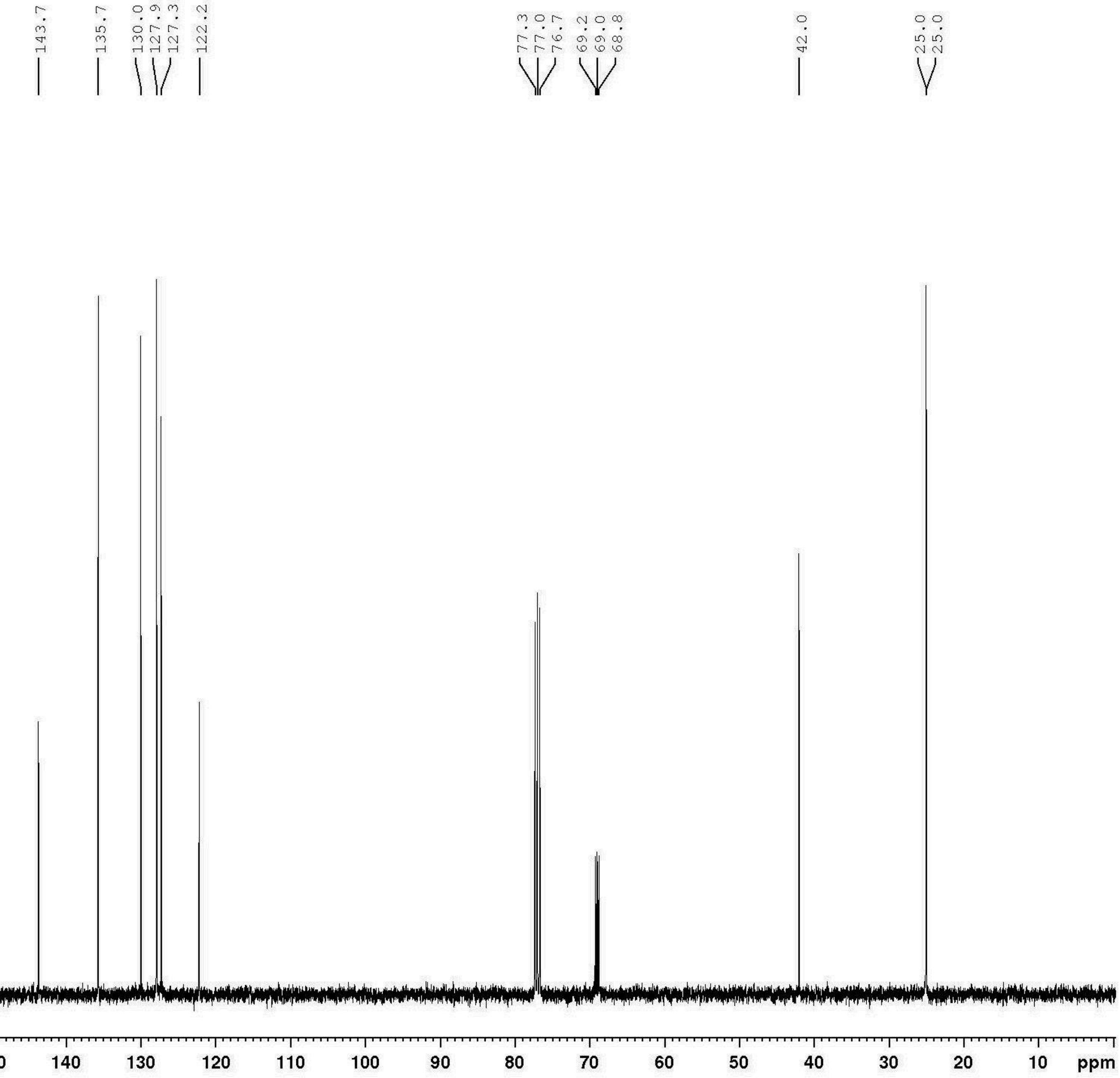

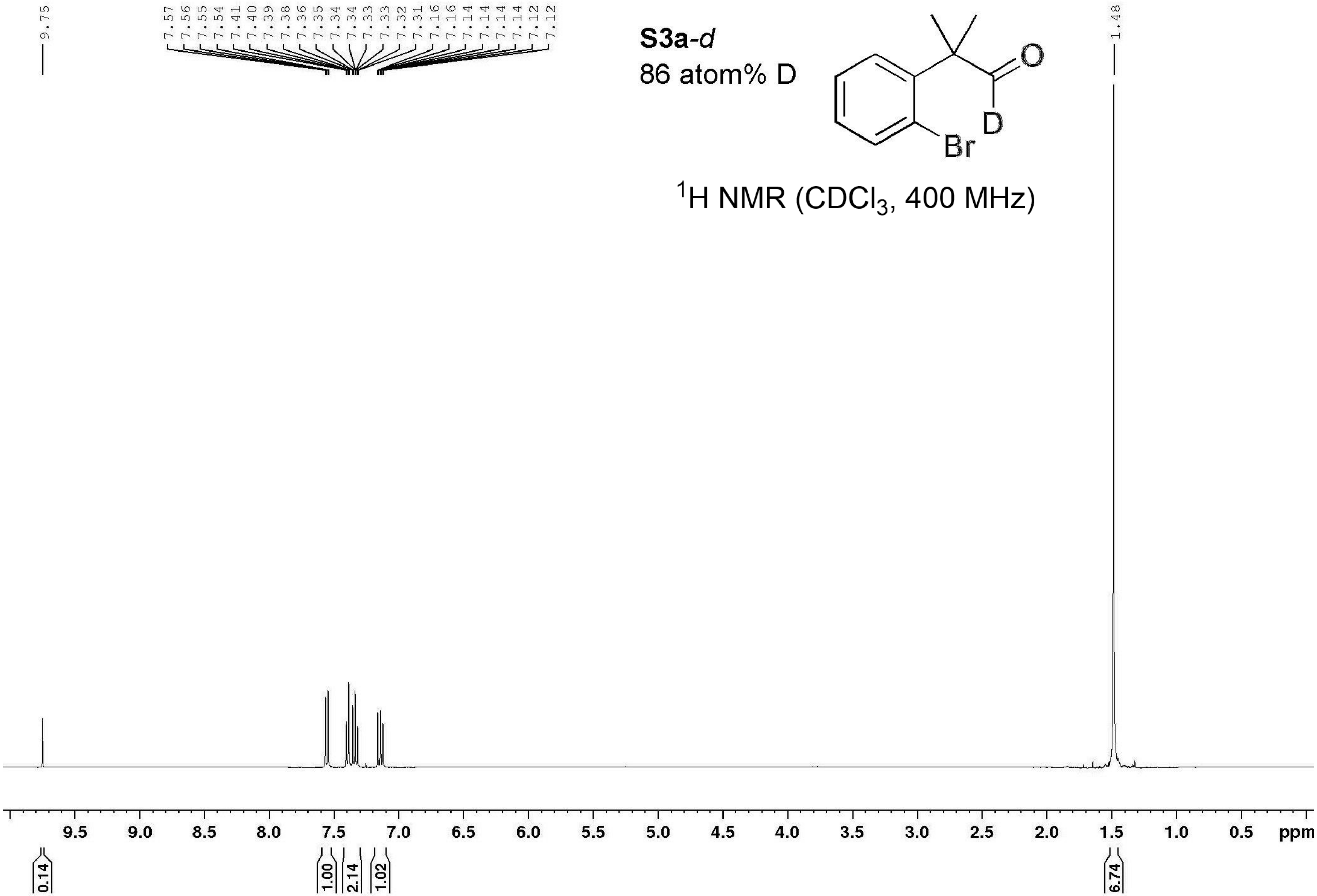
S3a-d
86 atom% D
D
Br
O
^{1}H NMR ($CDCl_3$, 400 MHz)
9.75
7.57 7.56 7.55 7.54 7.41 7.40 7.39 7.38 7.36 7.35 7.34 7.34 7.33 7.33 7.32 7.31 7.16 7.16 7.14 7.14 7.14 7.14 7.12 7.12
1.48
9.5 9.0 8.5 8.0 7.5 7.0 6.5 6.0 5.5 5.0 4.5 4.0 3.5 3.0 2.5 2.0 1.5 1.0 0.5 ppm
0.14
1.00
2.14
1.02
6.74

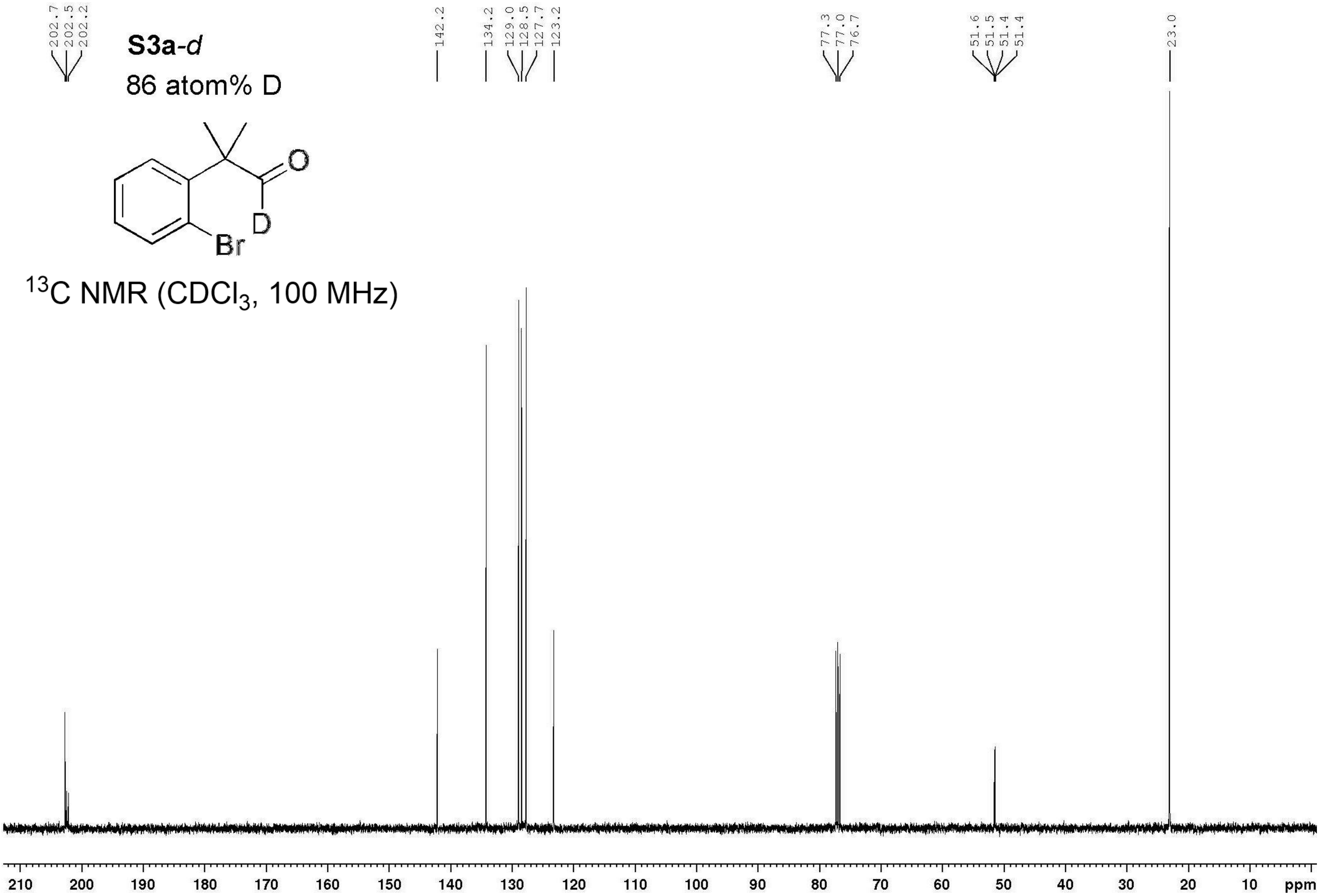
S3a-d
86 atom% D
D
Br
O
13C NMR (CDCl3, 100 MHz)
202.7
202.5
202.2
142.2
134.2
129.0
128.5
127.7
123.2
77.3
77.0
76.7
51.6
51.5
51.4
51.4
23.0
210 200 190 180 170 160 150 140 130 120 110 100 90 80 70 60 50 40 30 20 10 ppm

1a-*d*

87 atom% D

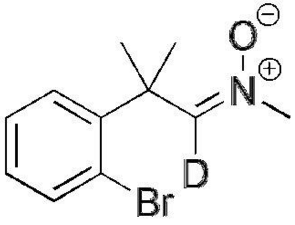

^{1}H NMR ($CDCl_3$, 500 MHz)

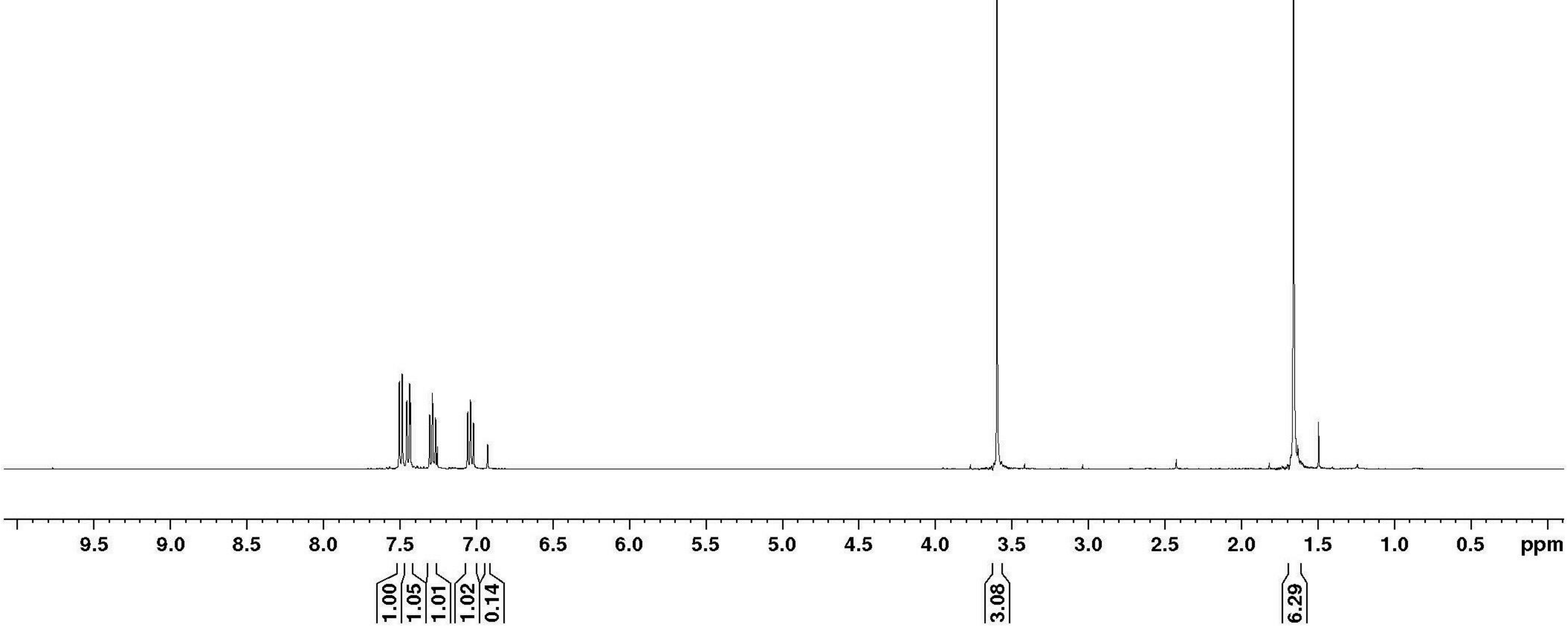

1a-*d*

87 atom% D

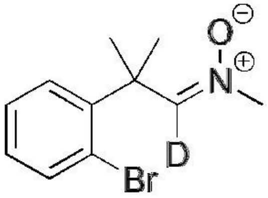

^{13}C NMR ($CDCl_3$, 125 MHz)

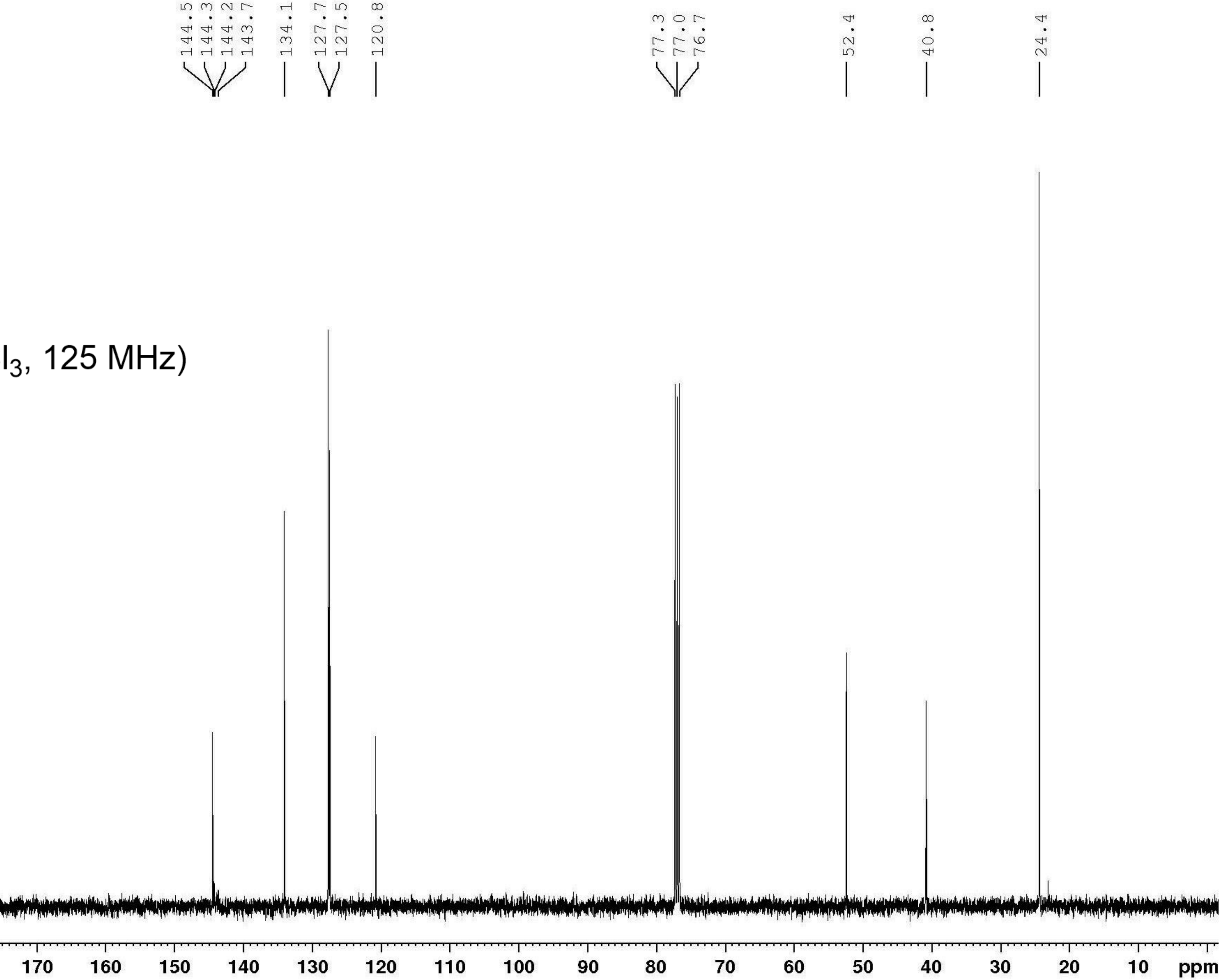

1a

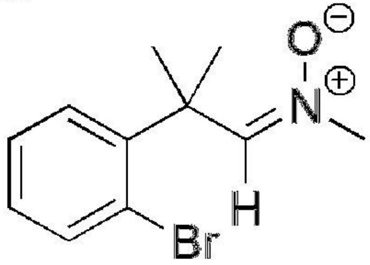

^{1}H NMR ($CDCl_3$, 400 MHz)

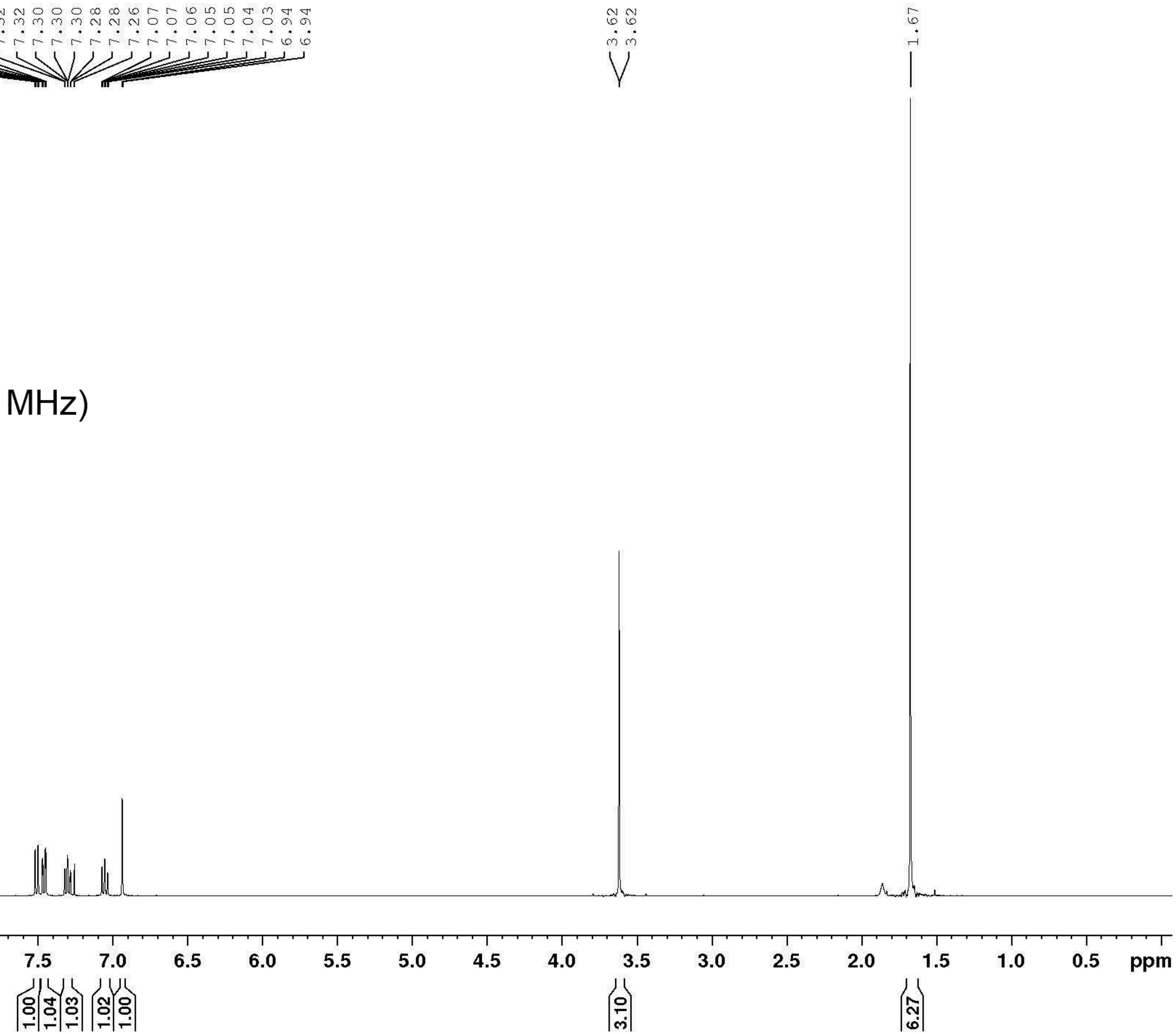

1a

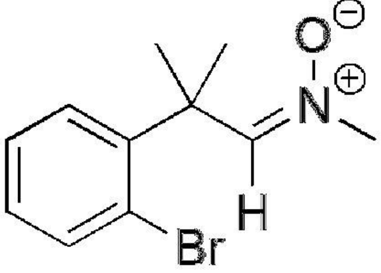

^{13}C NMR ($CDCl_3$, 100 MHz)

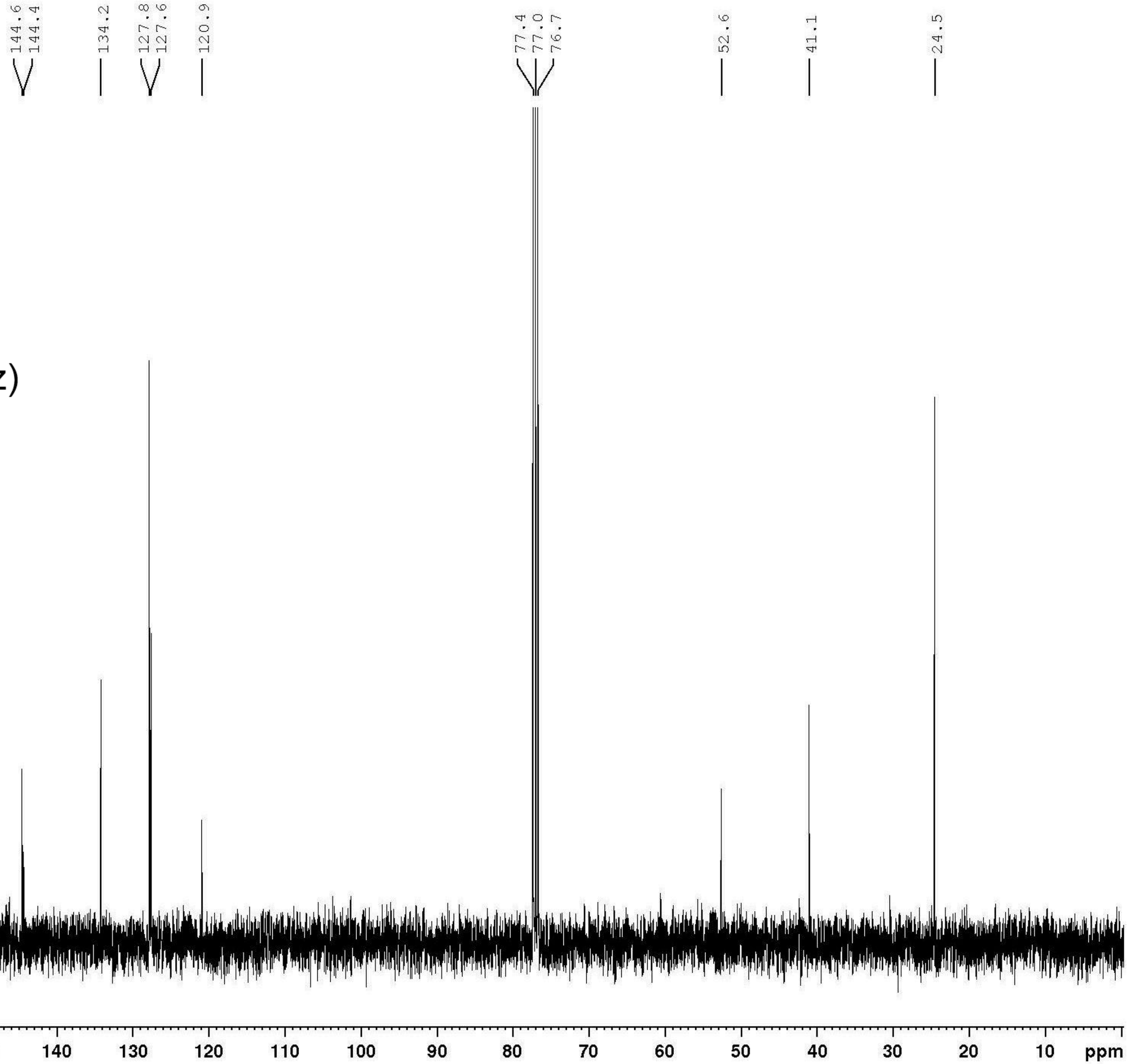

1b

^1H NMR ($CDCl_3$, 400 MHz)

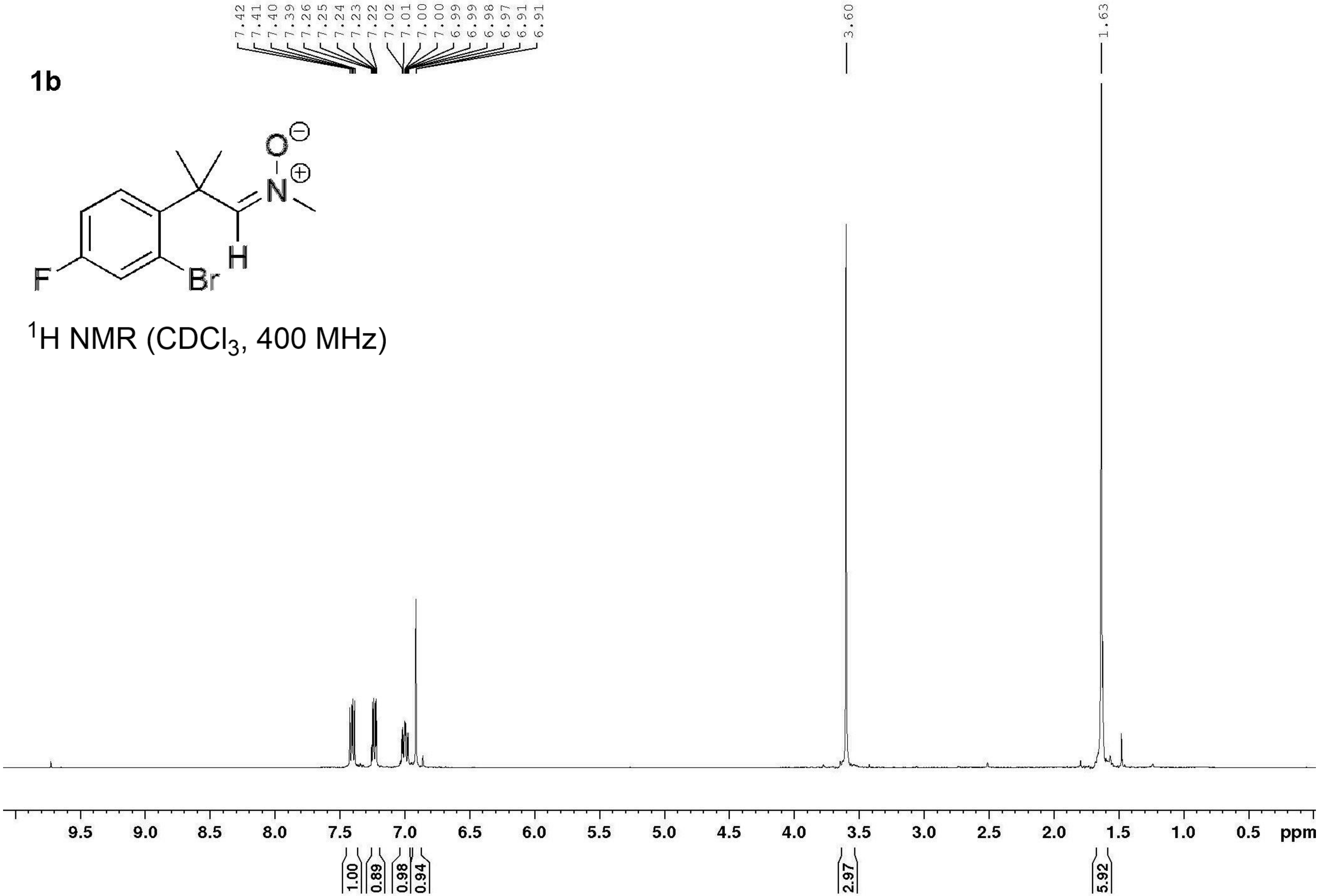

1b

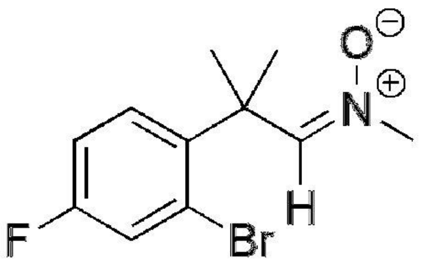

^{13}C NMR ($CDCl_3$, 100 MHz)

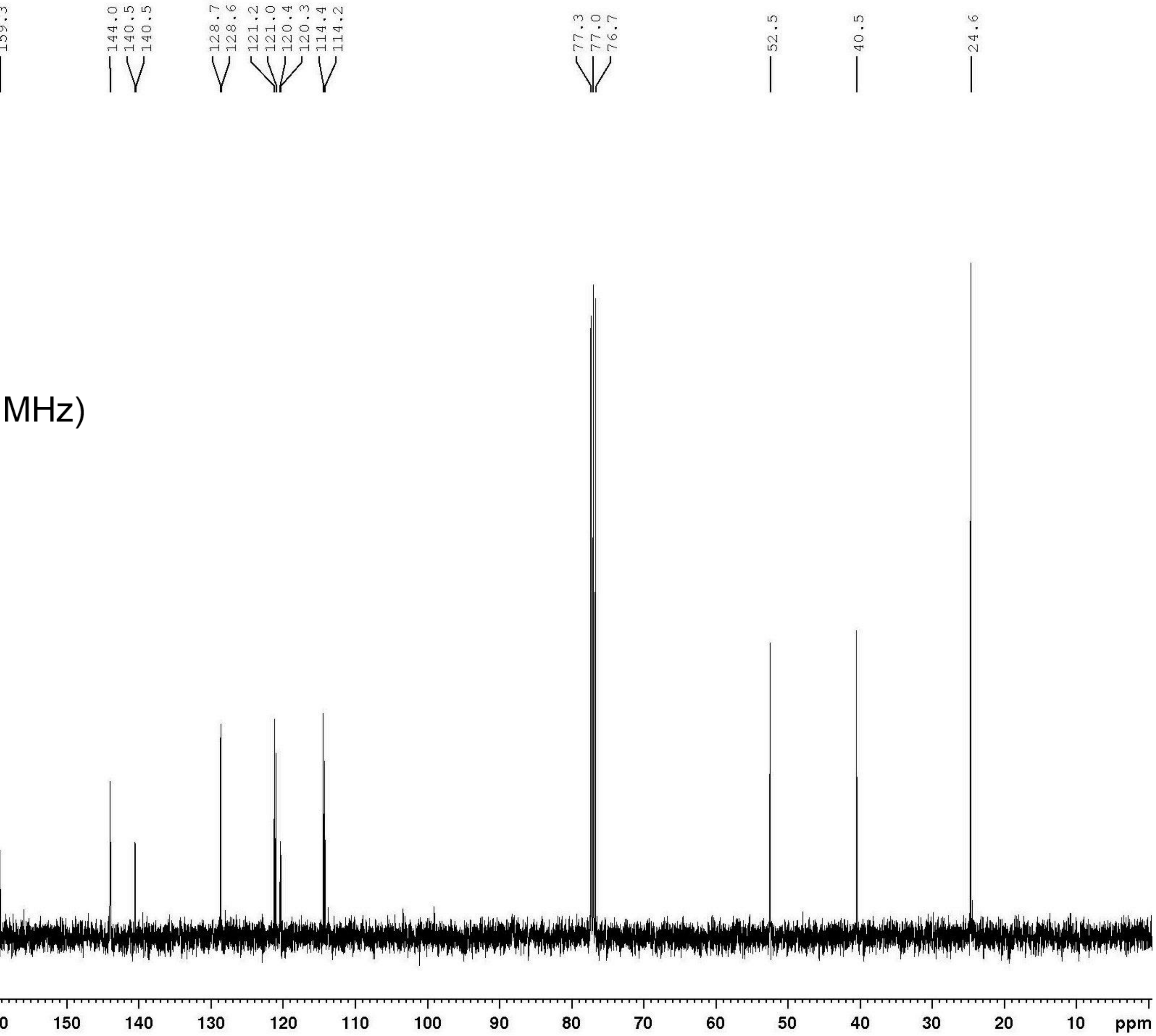

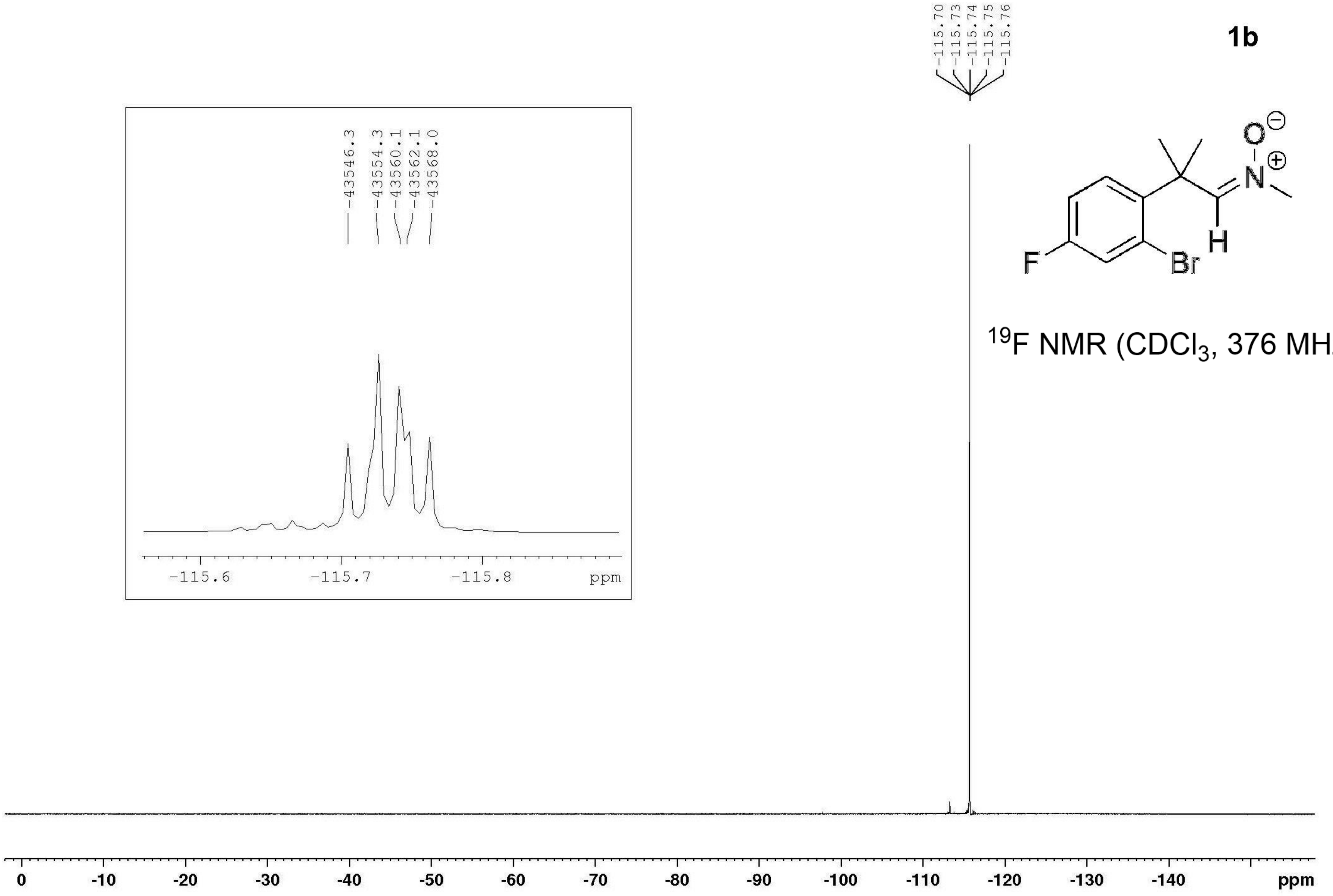

1b

^{19}F NMR ($CDCl_3$, 376 MHz)

1c

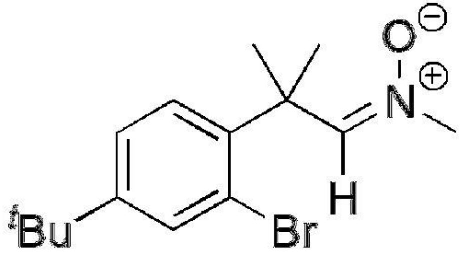

^1H NMR ($CDCl_3$, 400 MHz)

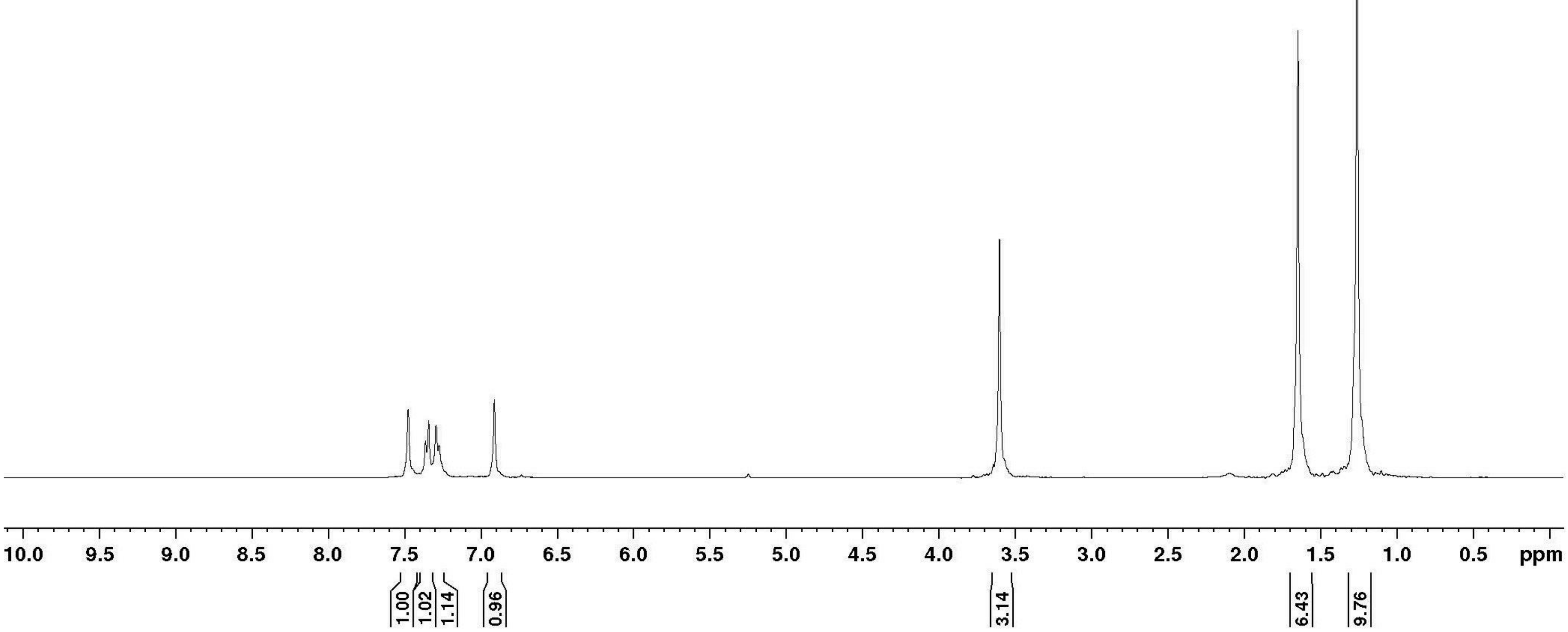

1c

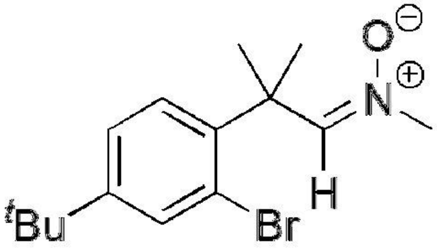

^{13}C NMR ($CDCl_3$, 100 MHz)

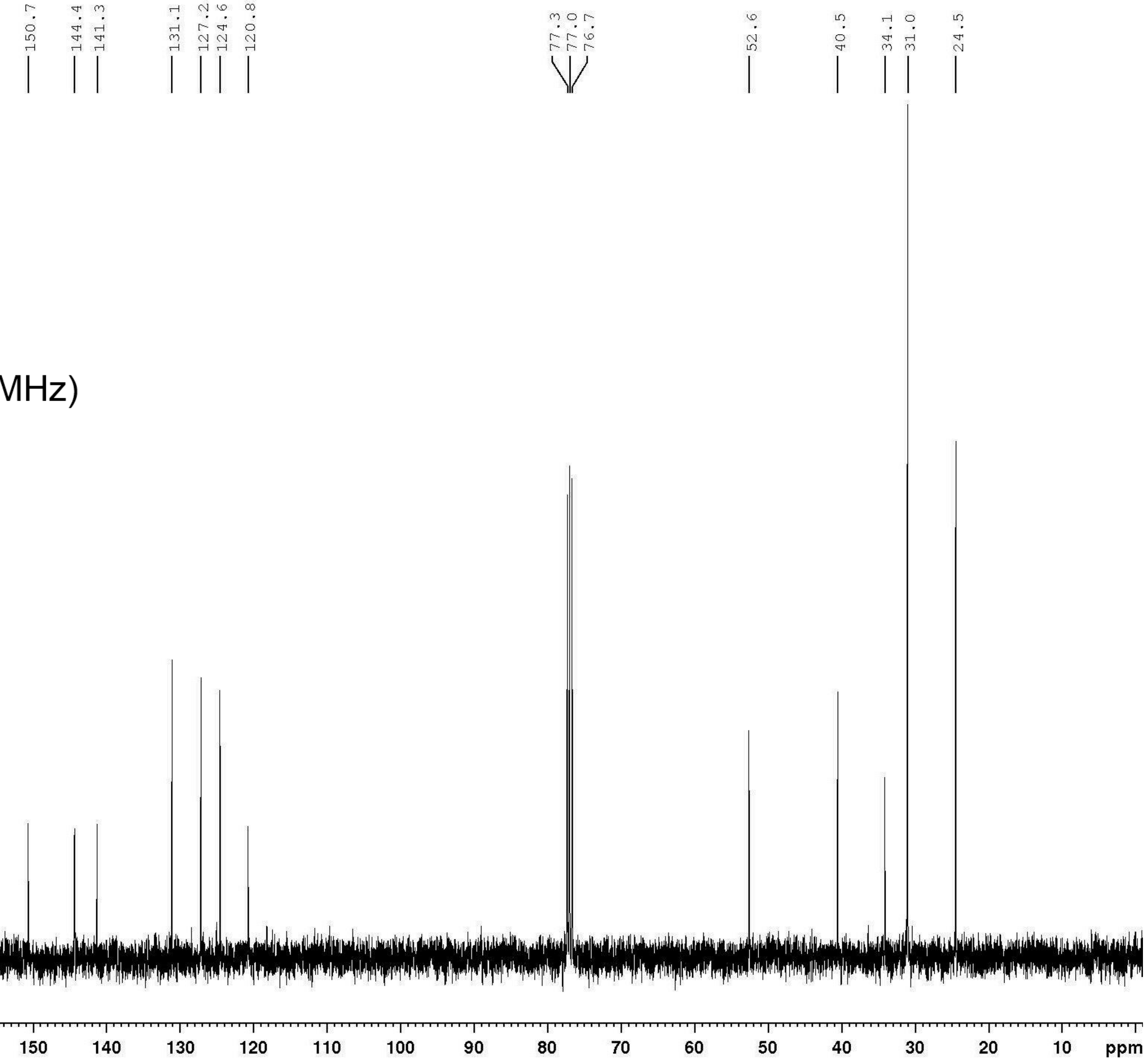

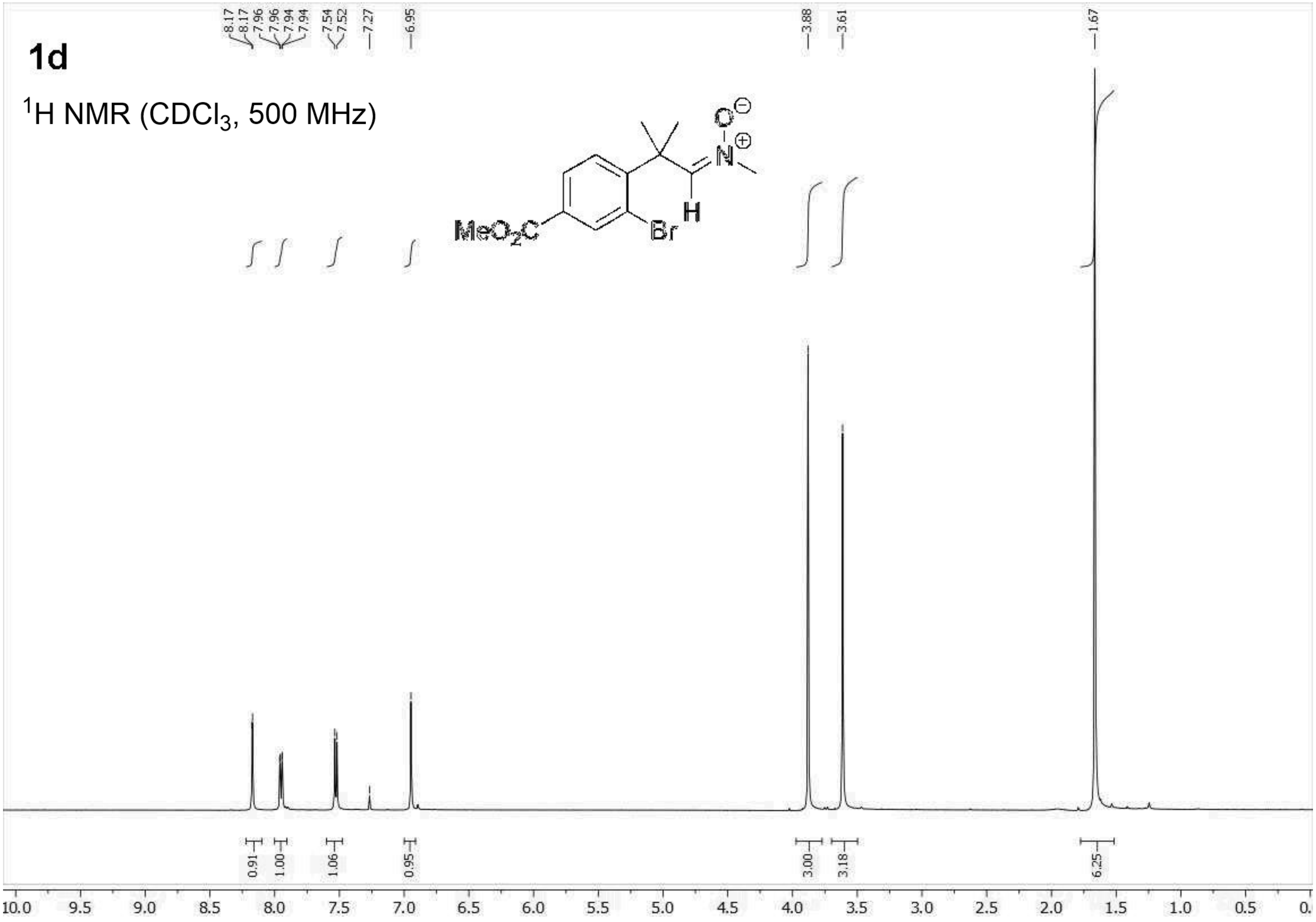
1d
^{1}H NMR (CDCl$_3$, 500 MHz)
8.17
8.17
7.96
7.96
7.94
7.94
7.54
7.52
7.27
6.95
3.88
3.61
1.67
MeO2C
Br
H
O
N
0.91
1.00
1.06
0.95
3.00
3.18
6.25
10.0
9.5
9.0
8.5
8.0
7.5
7.0
6.5
6.0
5.5
5.0
4.5
4.0
3.5
3.0
2.5
2.0
1.5
1.0
0.5

1d

^{13}C NMR ($CDCl_3$, 125 MHz)

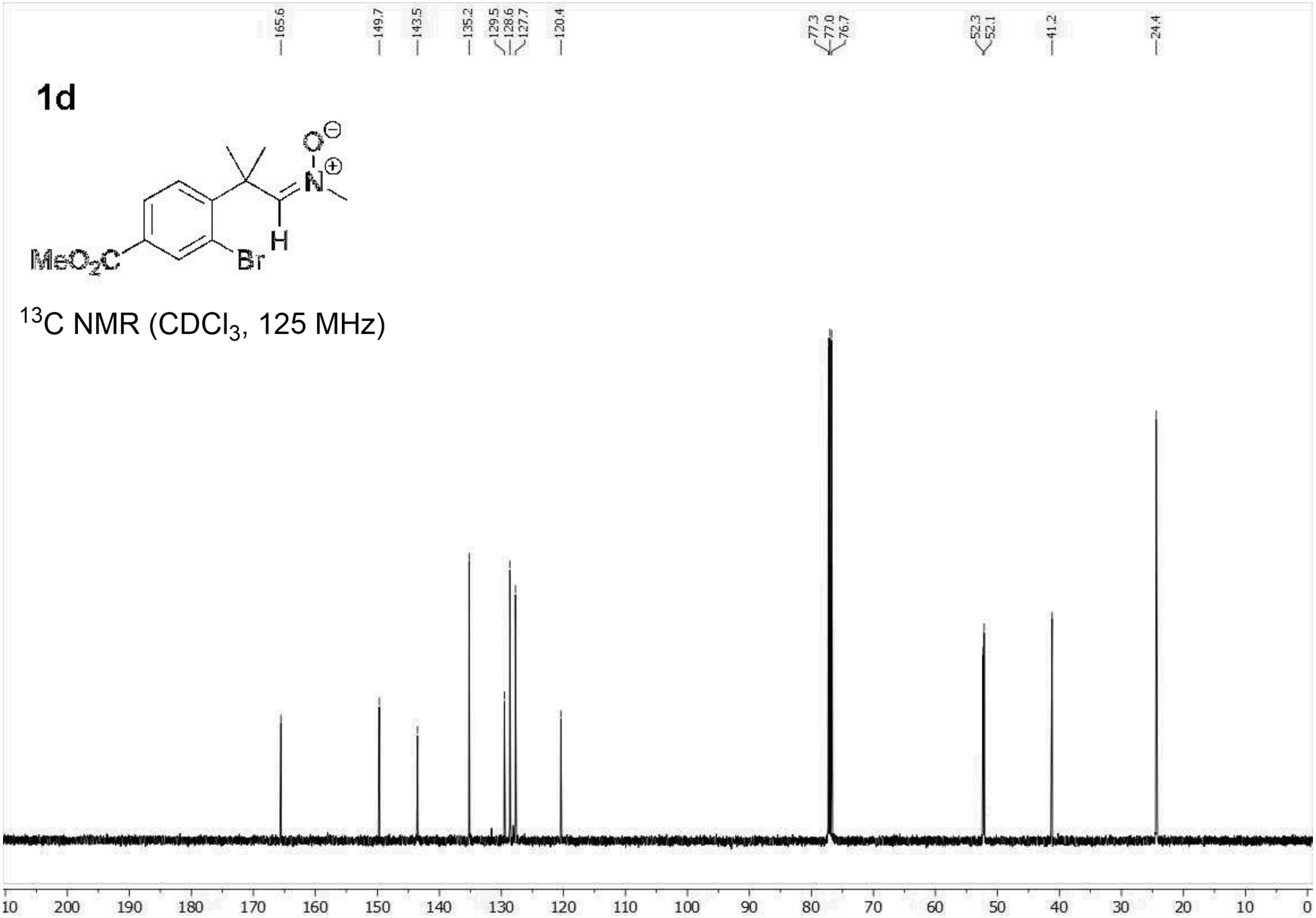

1e

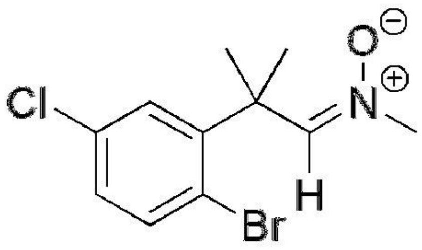

^{1}H NMR ($CDCl_3$, 400 MHz)

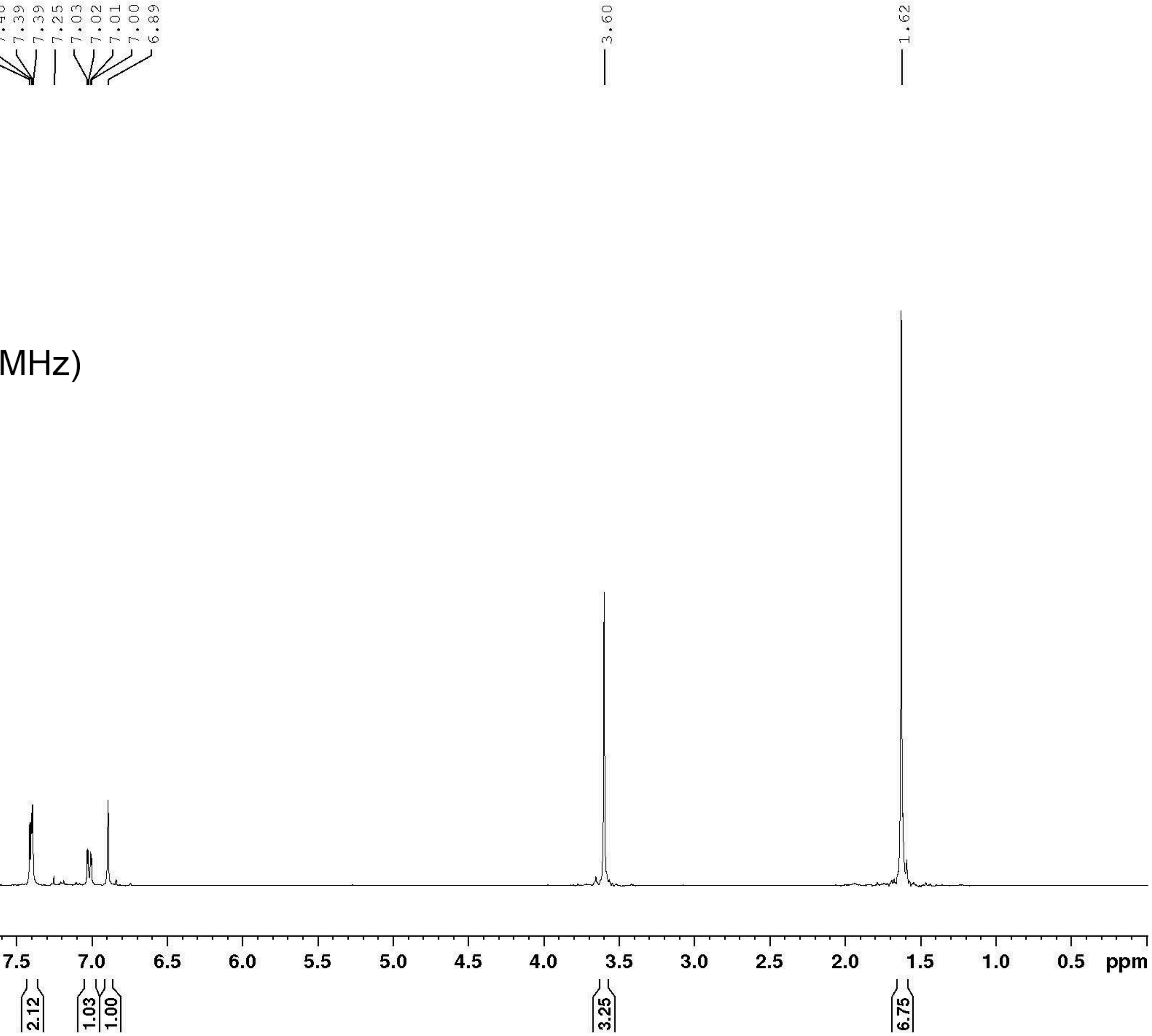

1e

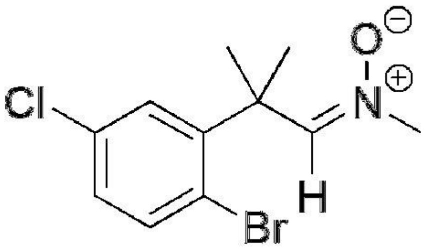

^{13}C NMR ($CDCl_3$, 100 MHz)

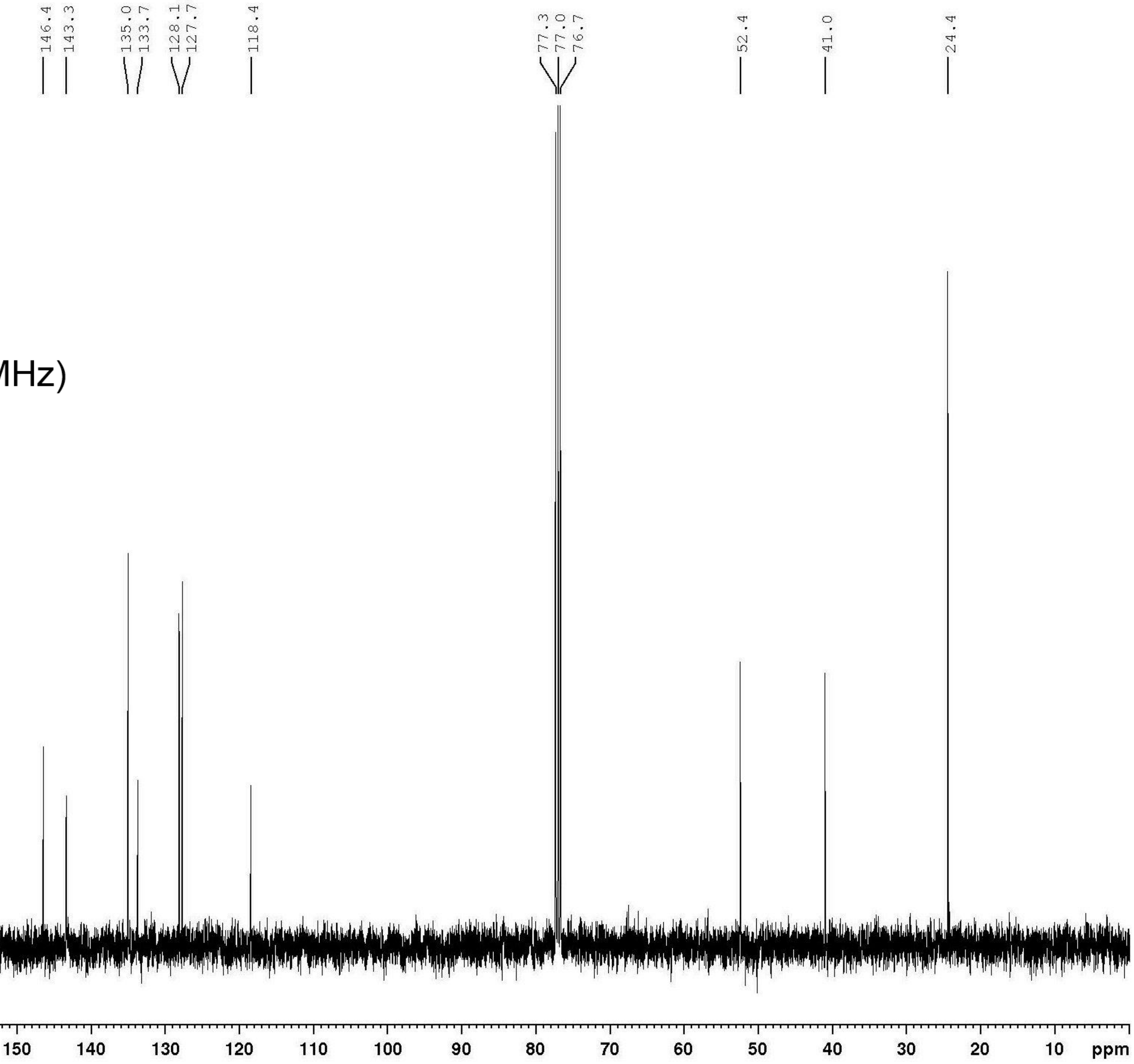

1f

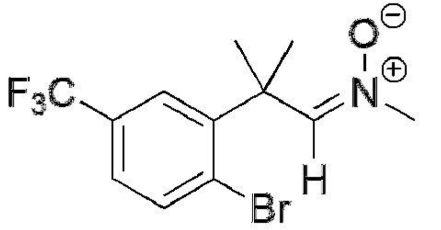

^1H NMR ($CDCl_3$, 500 MHz)

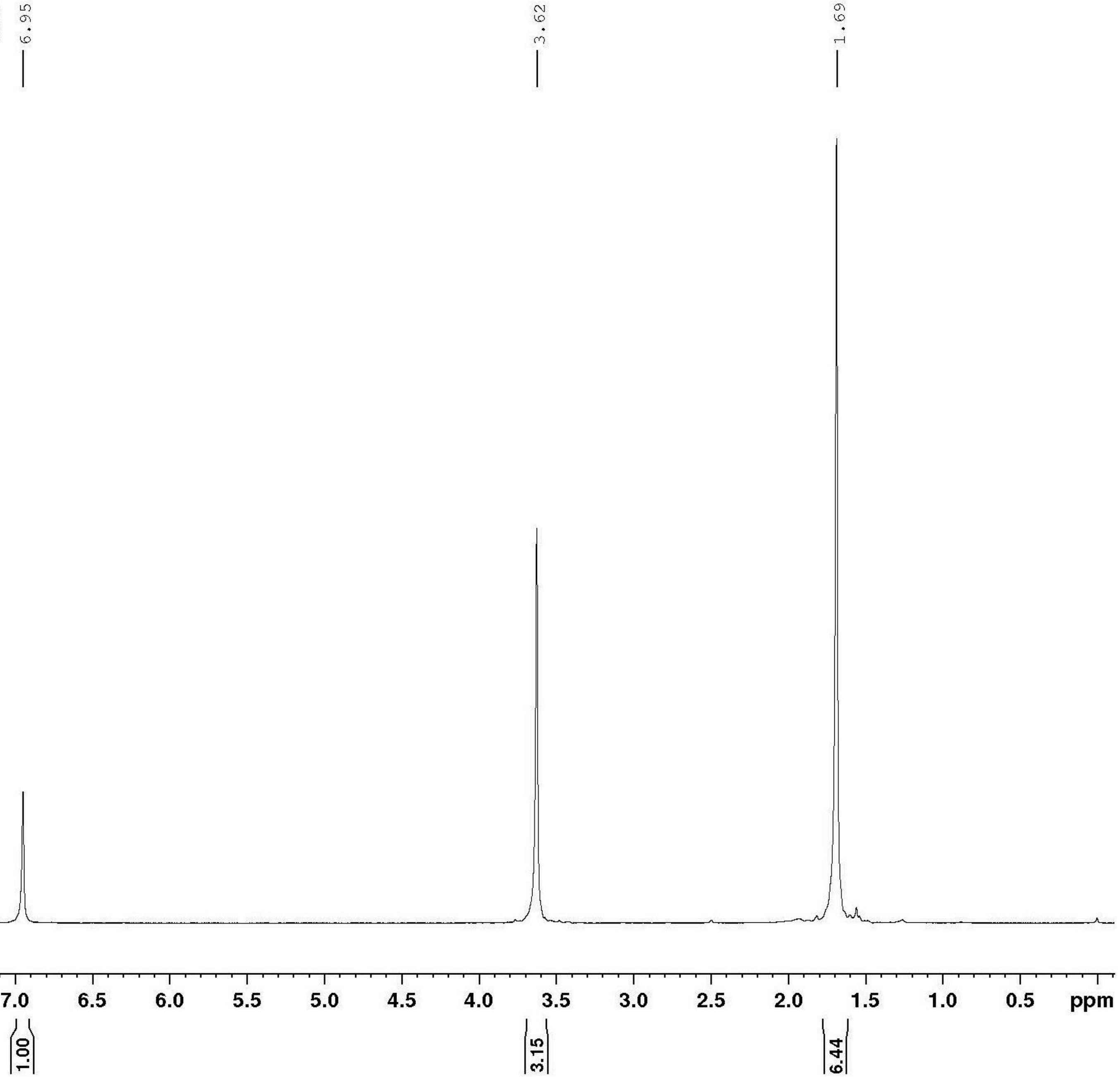

1f

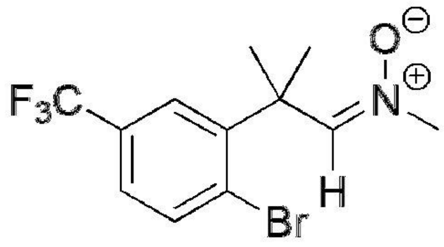

^{13}C NMR ($CDCl_3$, 100 MHz)

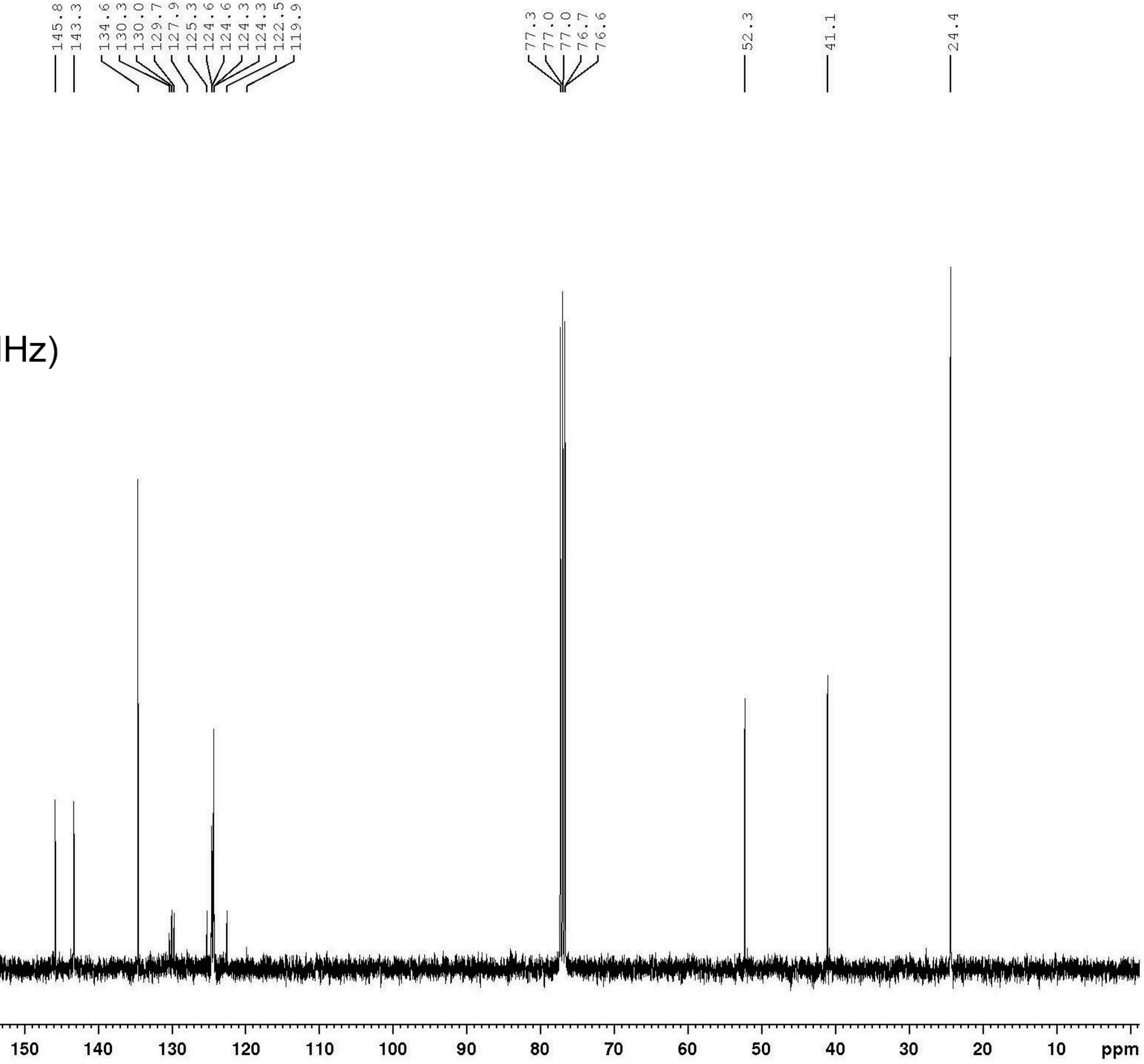

1f

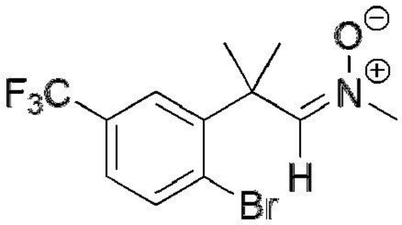

^{19}F NMR ($CDCl_3$, 376 MHz)

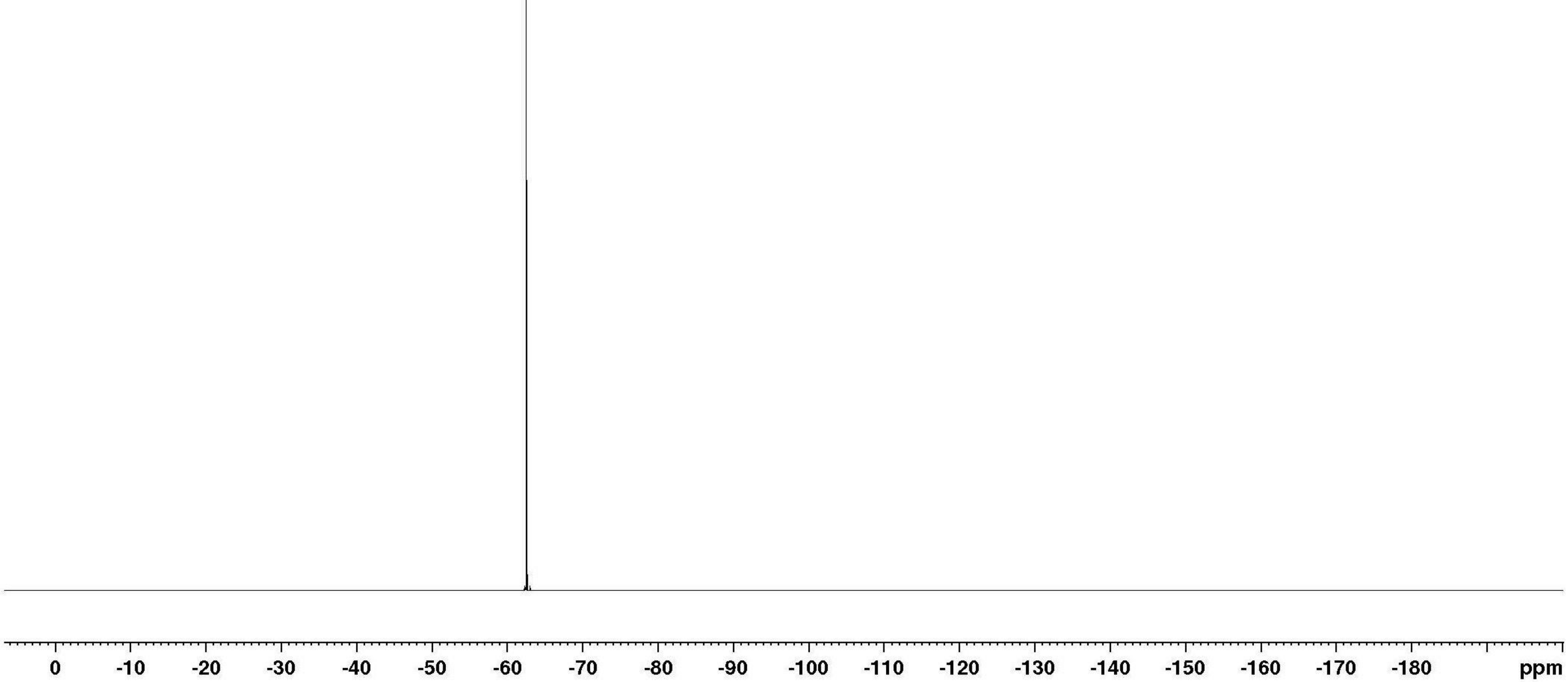

1g

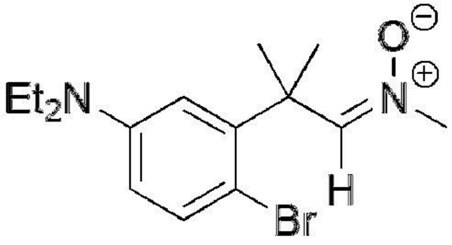

^{1}H NMR ($CDCl_3$, 400 MHz)

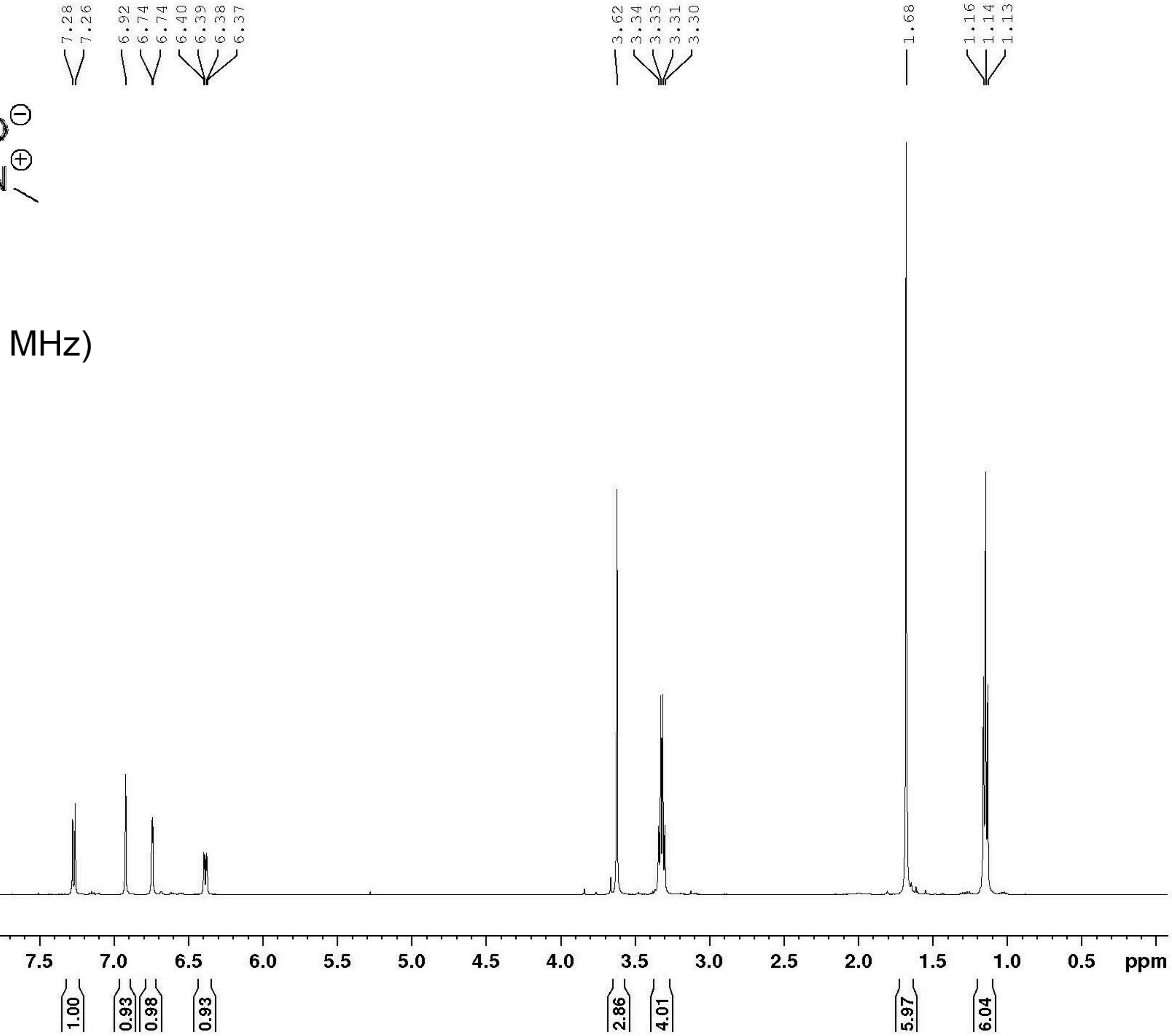

1g

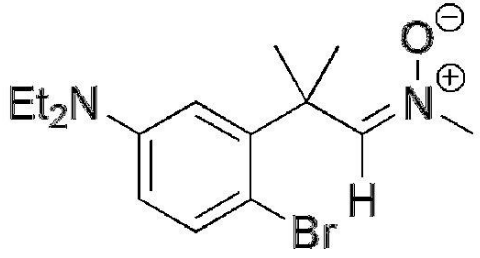

^{13}C NMR ($CDCl_3$, 100 MHz)

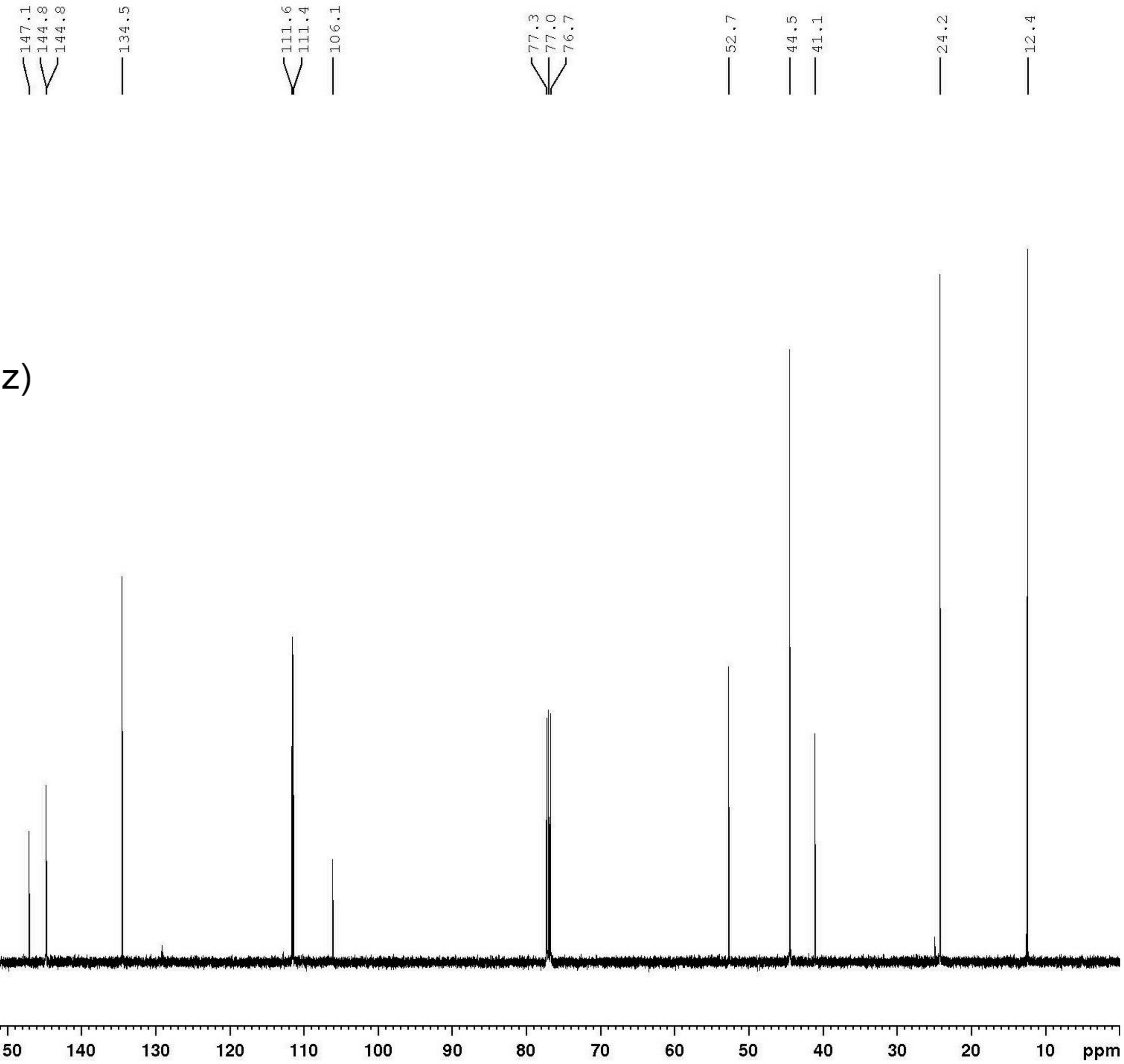

1h

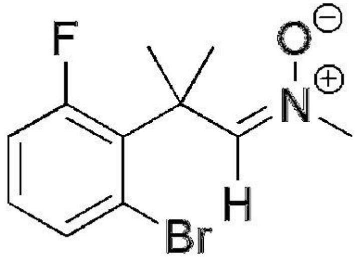

^{1}H NMR (CDCl$_3$, 400 MHz)

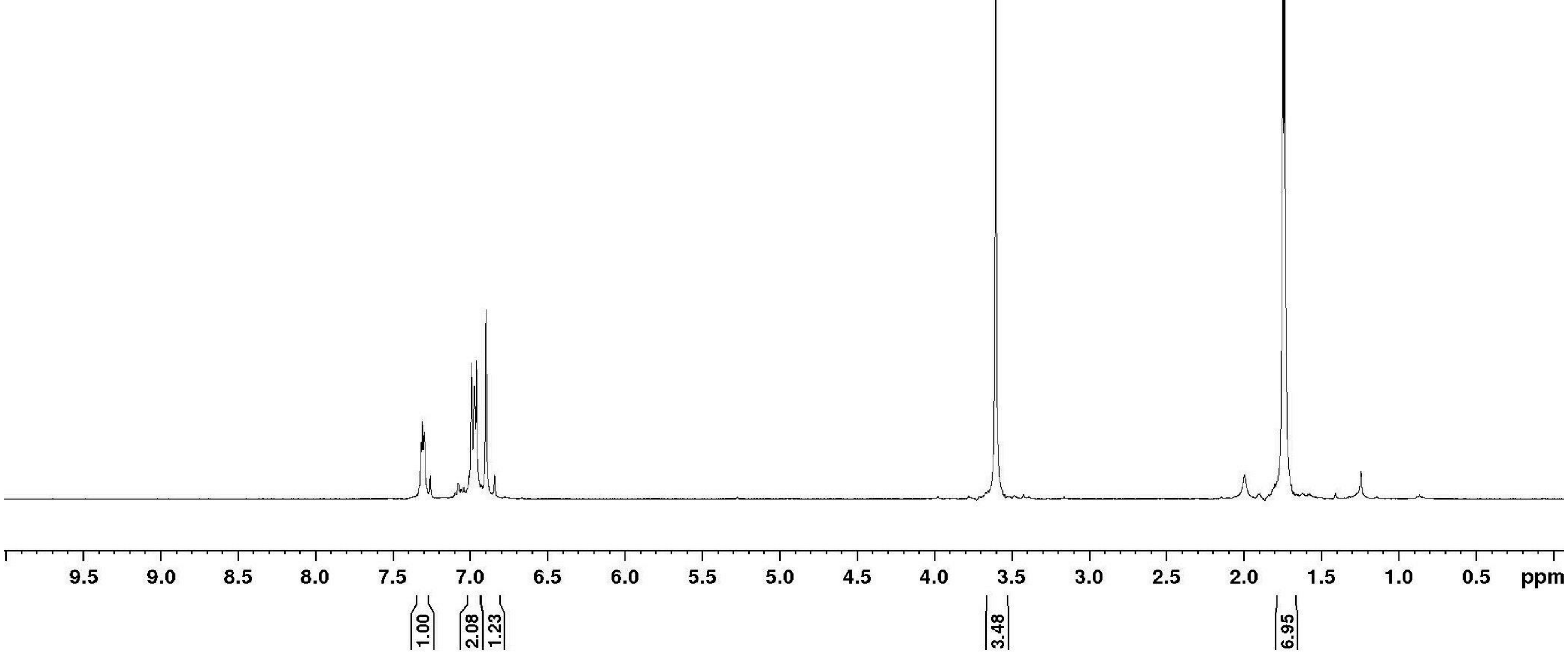

1h

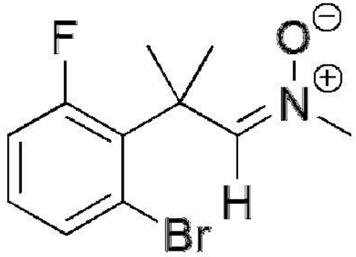

^{13}C NMR ($CDCl_3$, 100 MHz)

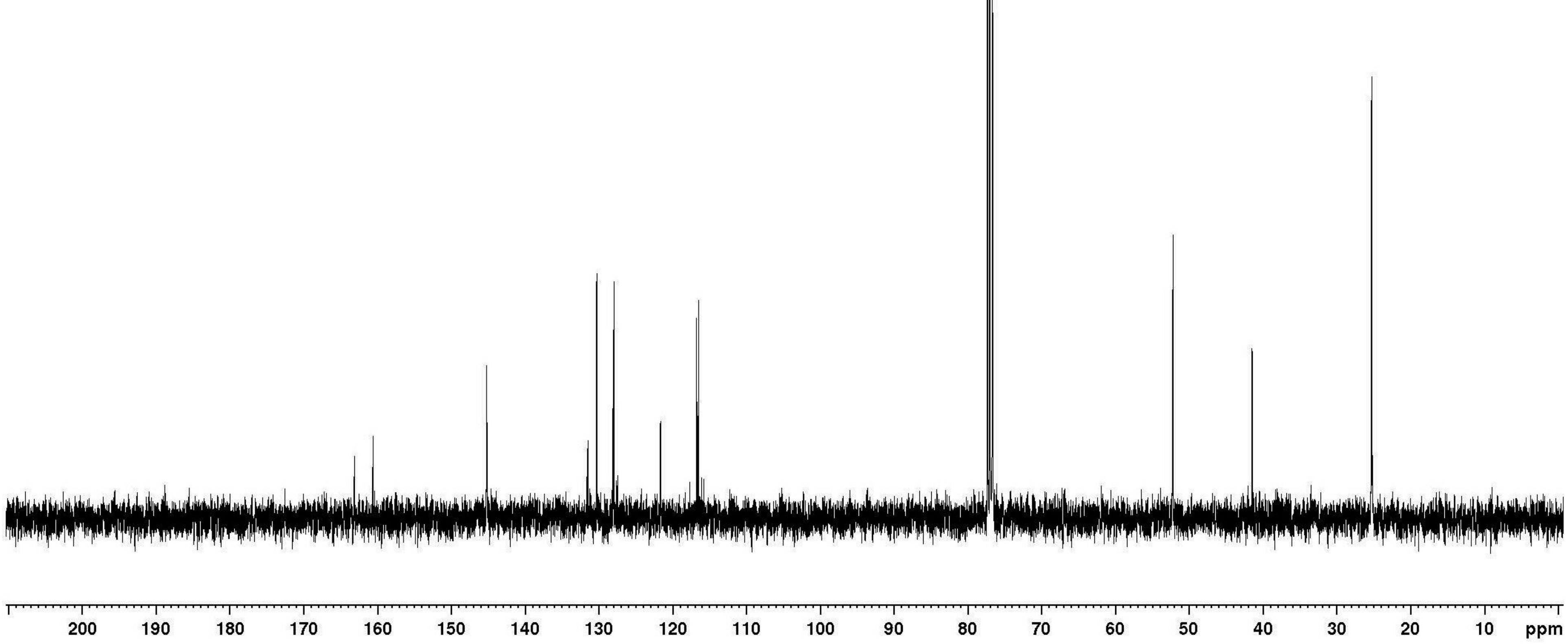

1i

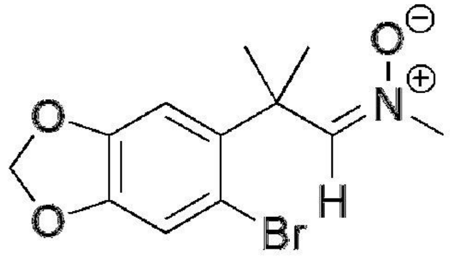

^{1}H NMR ($CDCl_3$, 400 MHz)

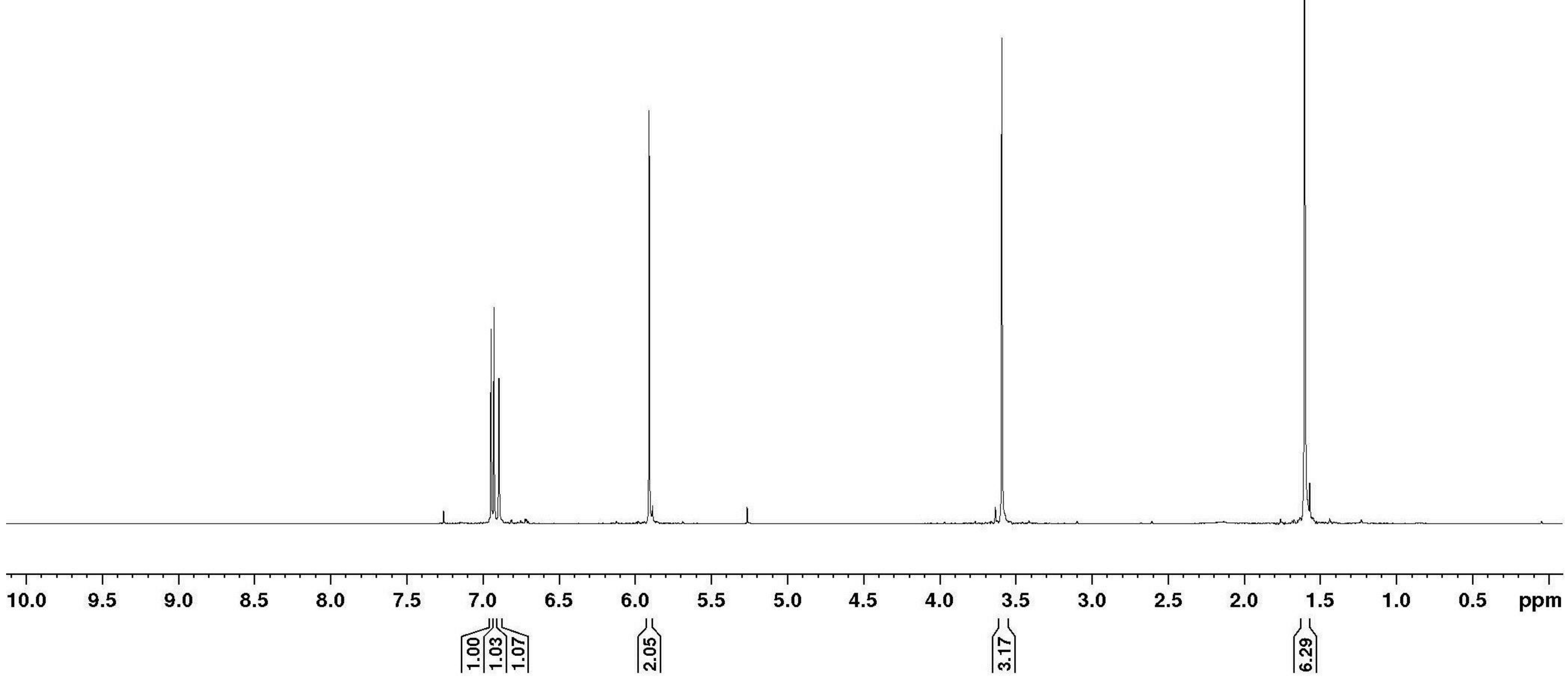

1i

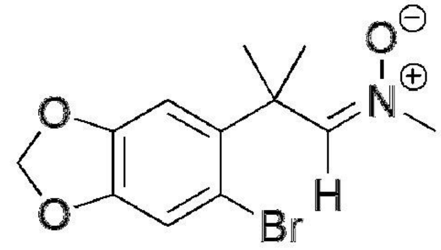

^{13}C NMR ($CDCl_3$, 100 MHz)

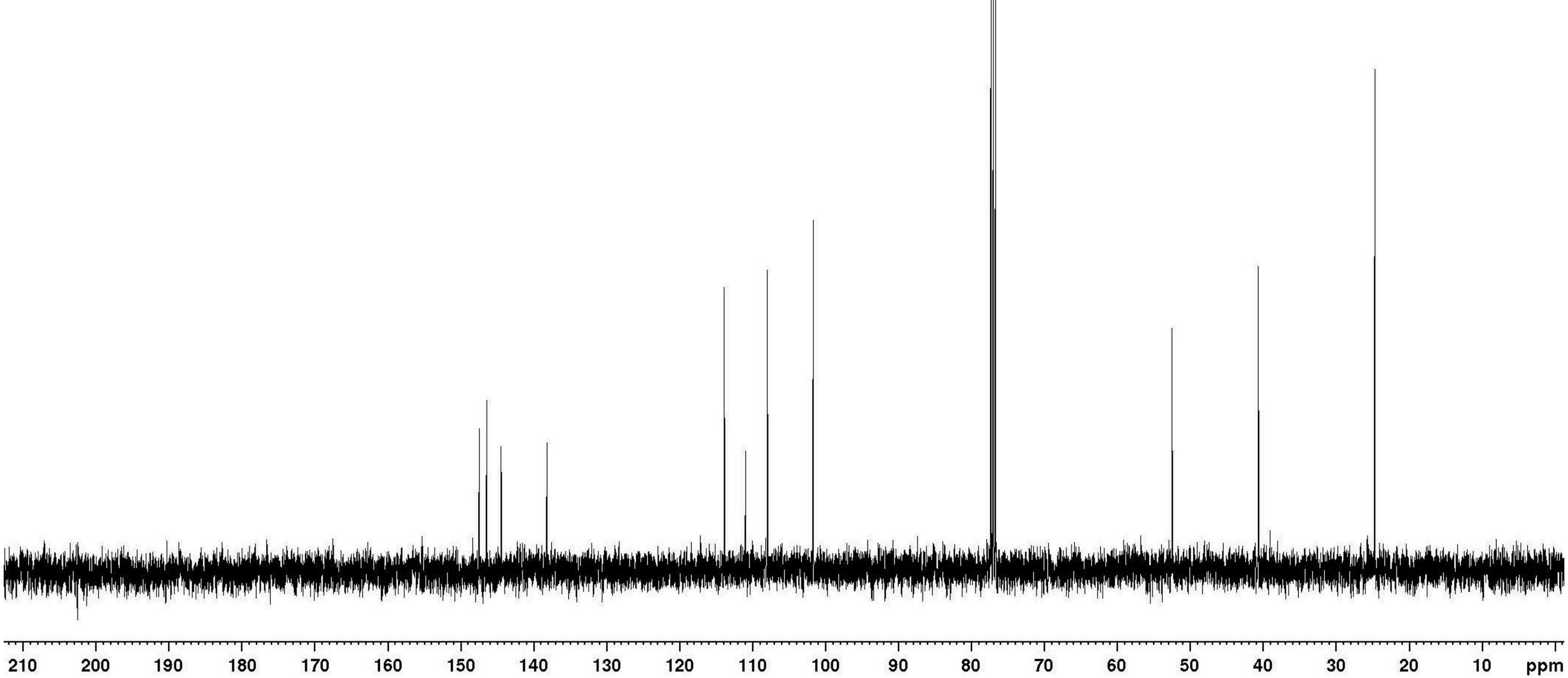

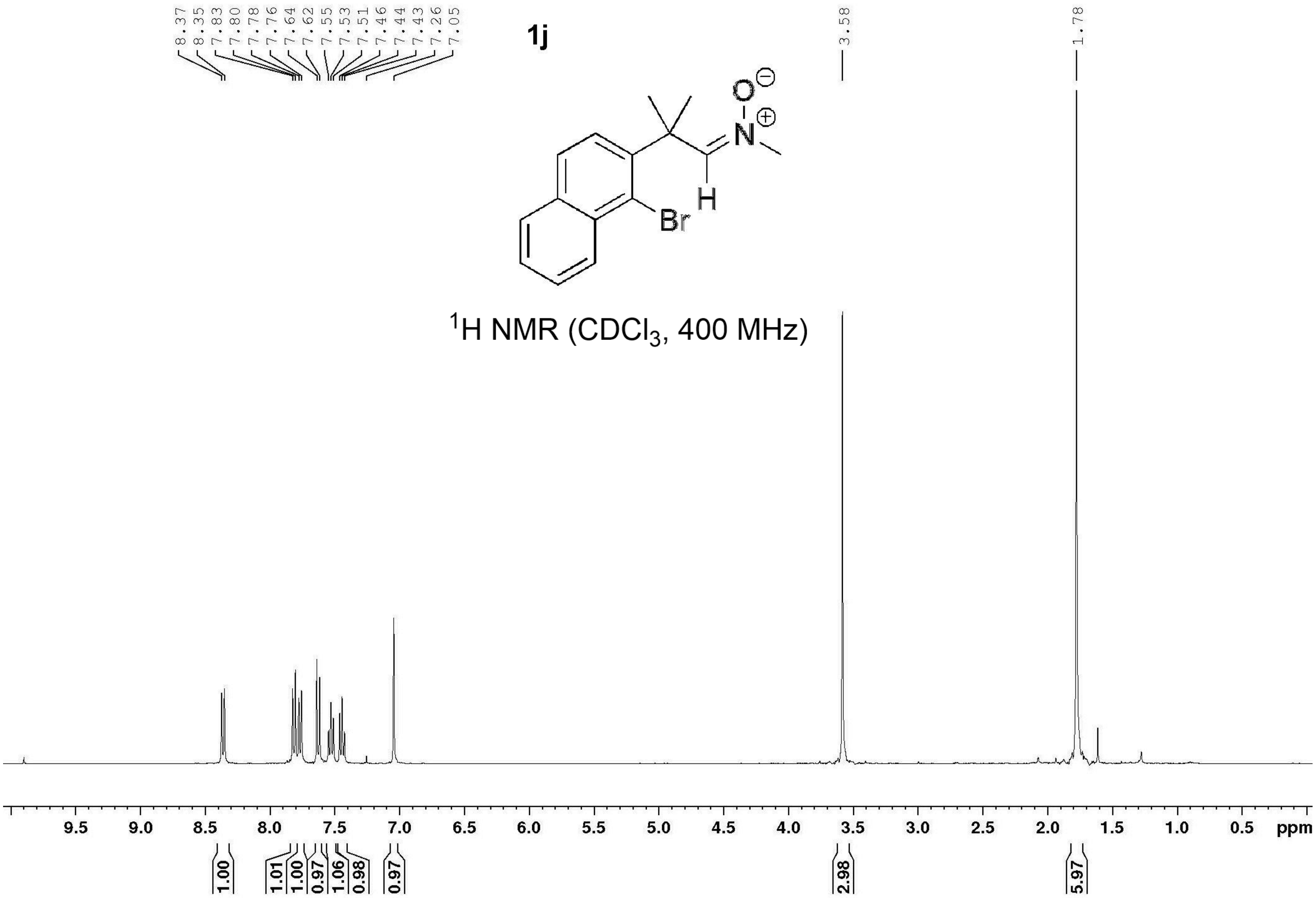
1j
8.37 8.35 7.83 7.80 7.78 7.76 7.64 7.62 7.55 7.53 7.51 7.46 7.44 7.43 7.26 7.05
3.58
1.78
Br
H
O
N
1H NMR (CDCl3, 400 MHz)
9.5 9.0 8.5 8.0 7.5 7.0 6.5 6.0 5.5 5.0 4.5 4.0 3.5 3.0 2.5 2.0 1.5 1.0 0.5 ppm
1.00 1.01 1.00 0.97 1.06 0.98 0.97 2.98 5.97

1j

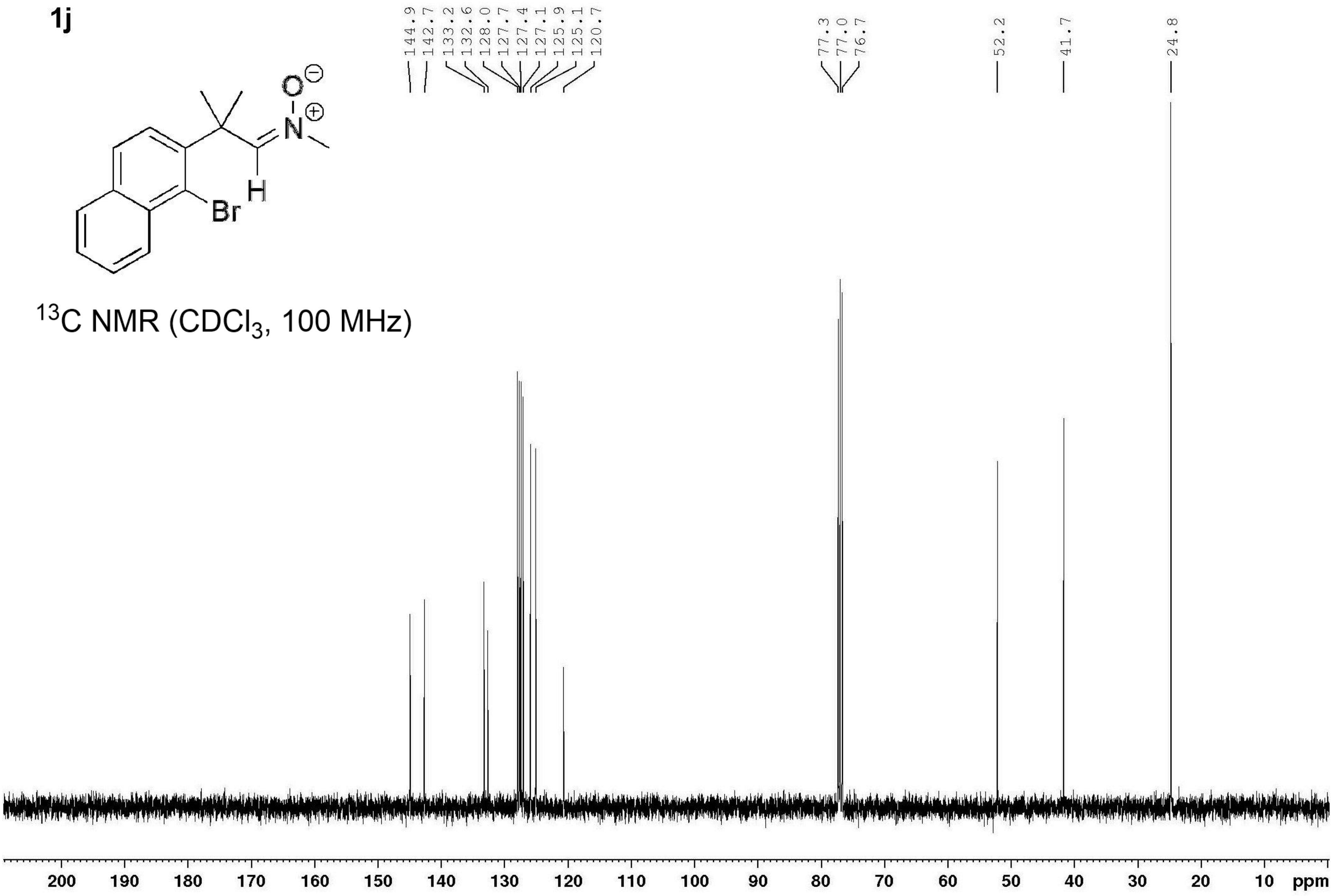

^{13}C NMR ($CDCl_3$, 100 MHz)

1k

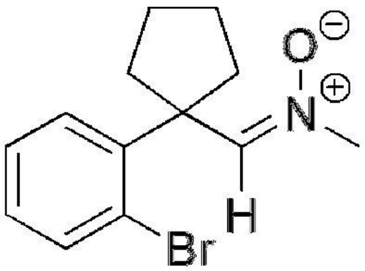

^1H NMR ($CDCl_3$, 400 MHz)

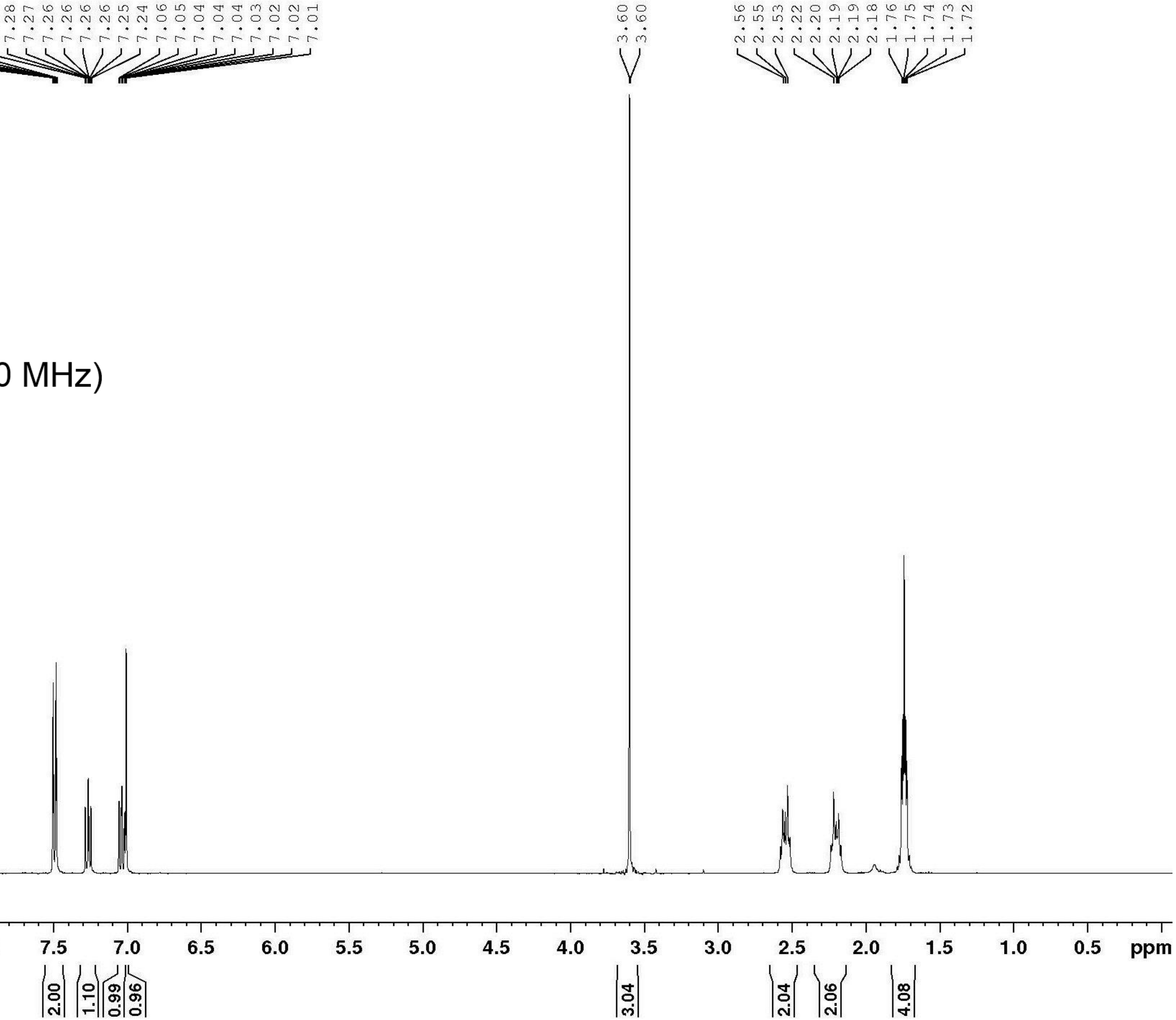

1k

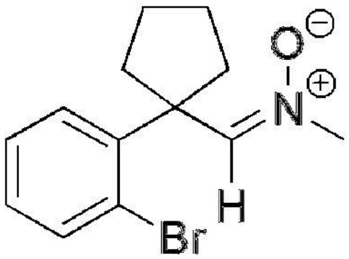

^{13}C NMR ($CDCl_3$, 100 MHz)

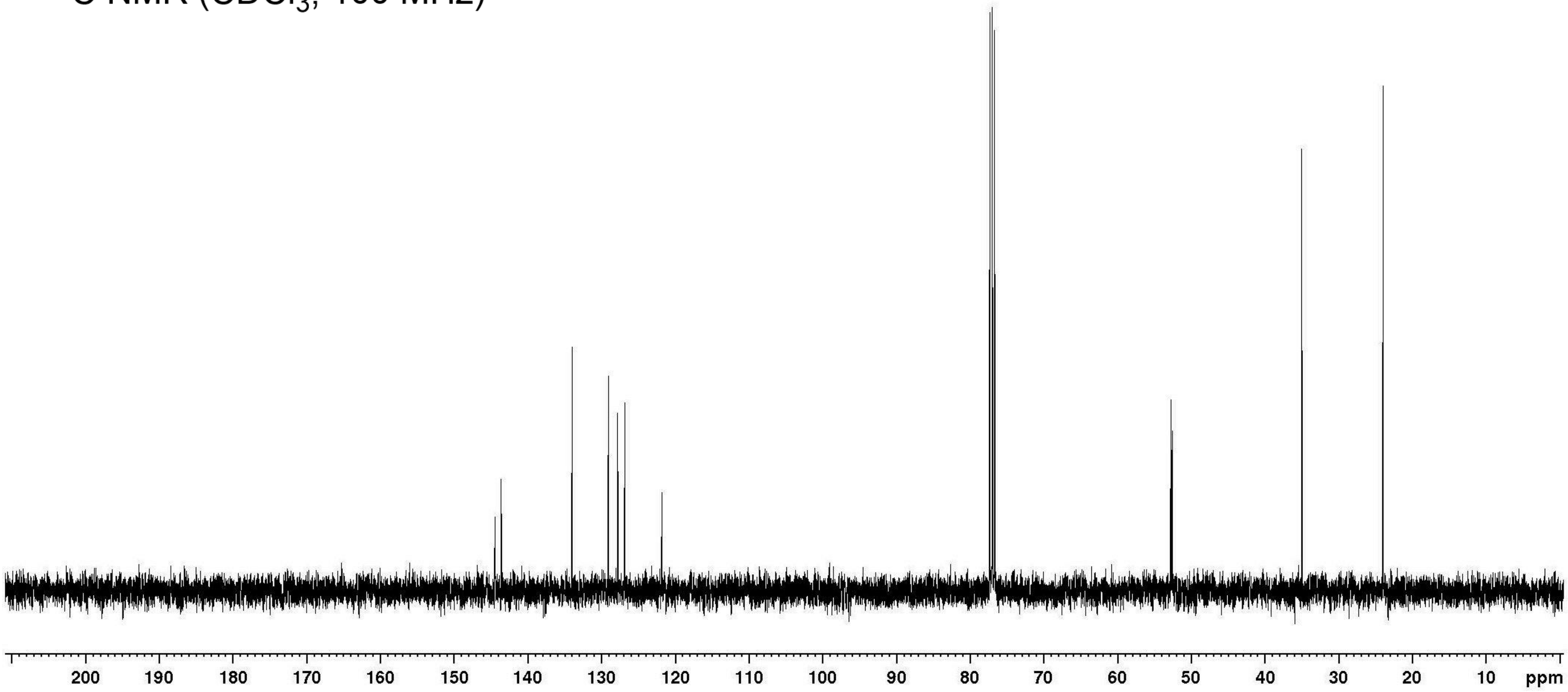

1l

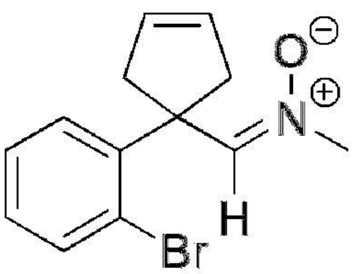

^{1}H NMR ($CDCl_3$, 400 MHz)

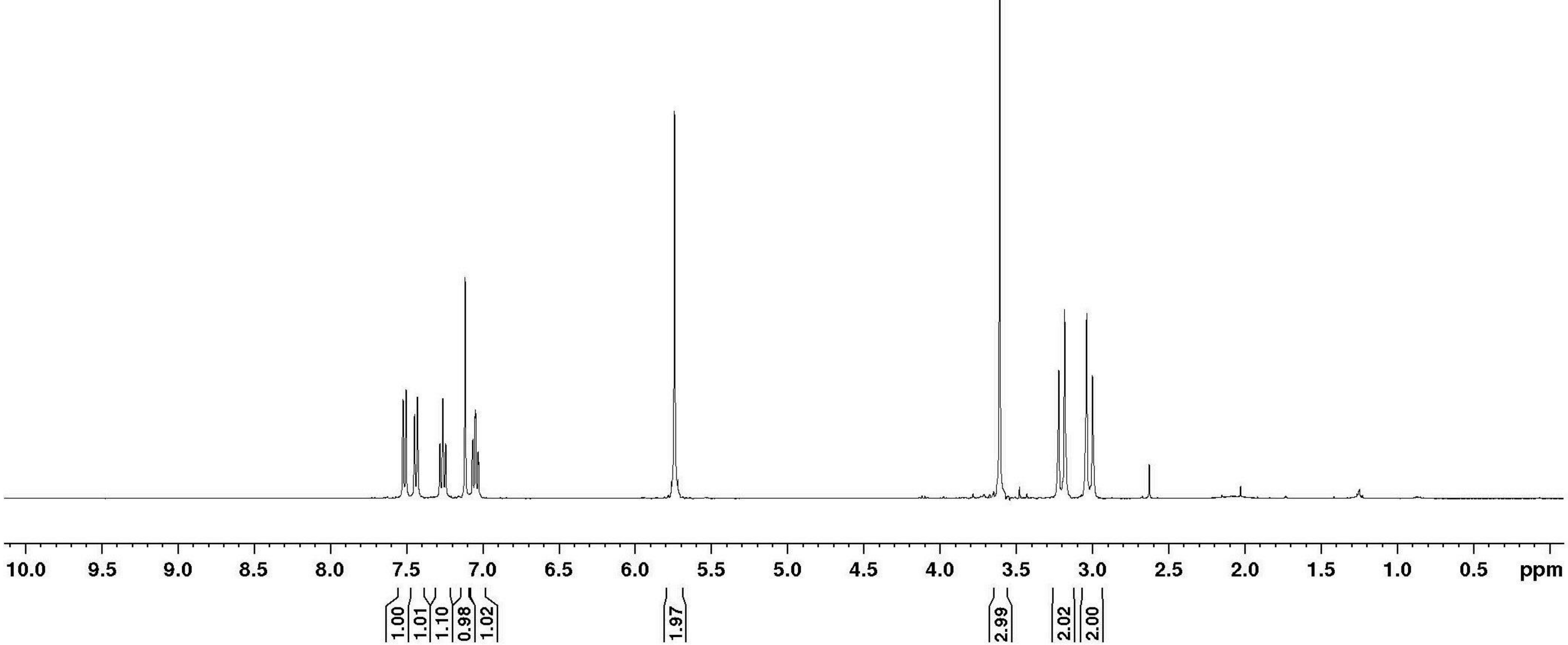

1l

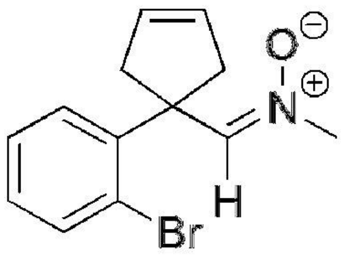

^{13}C NMR ($CDCl_3$, 100 MHz)

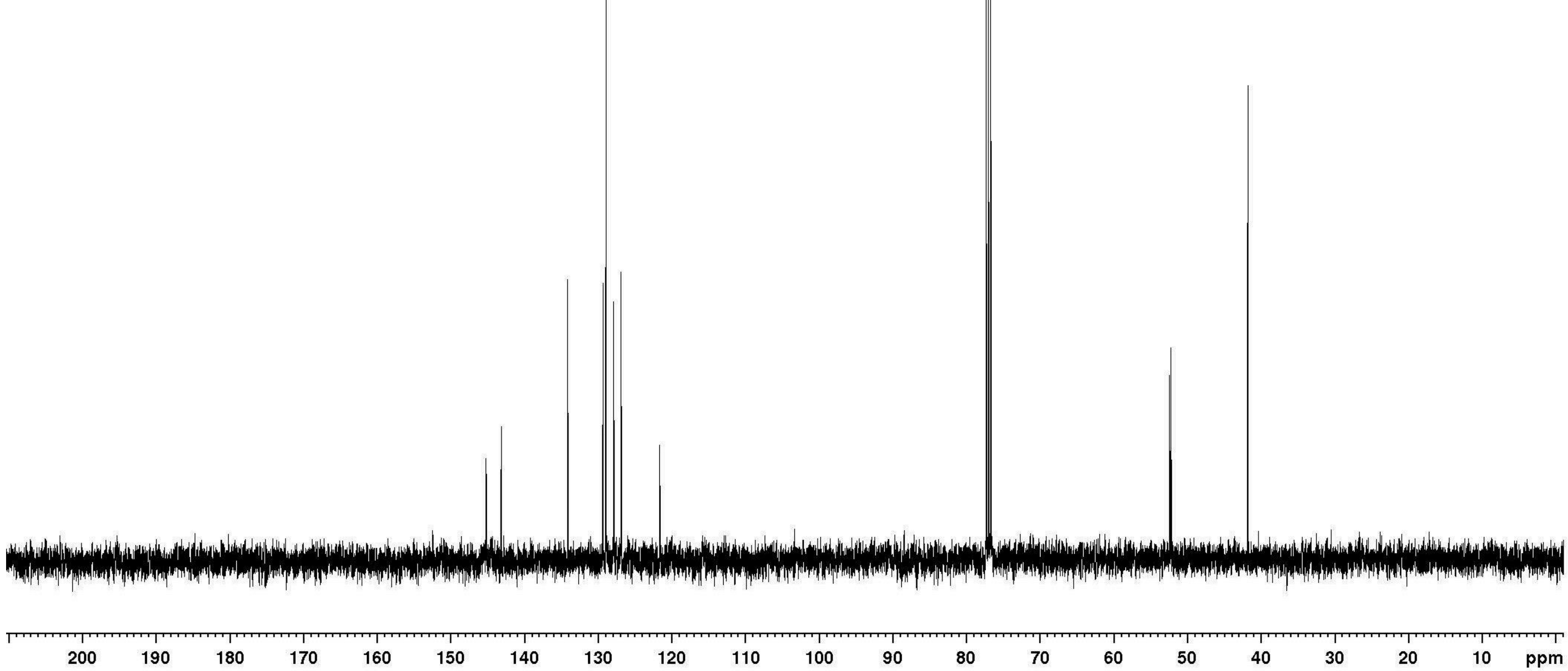

1m

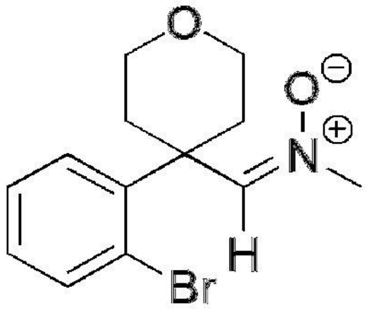

^{1}H NMR ($CDCl_3$, 400 MHz)

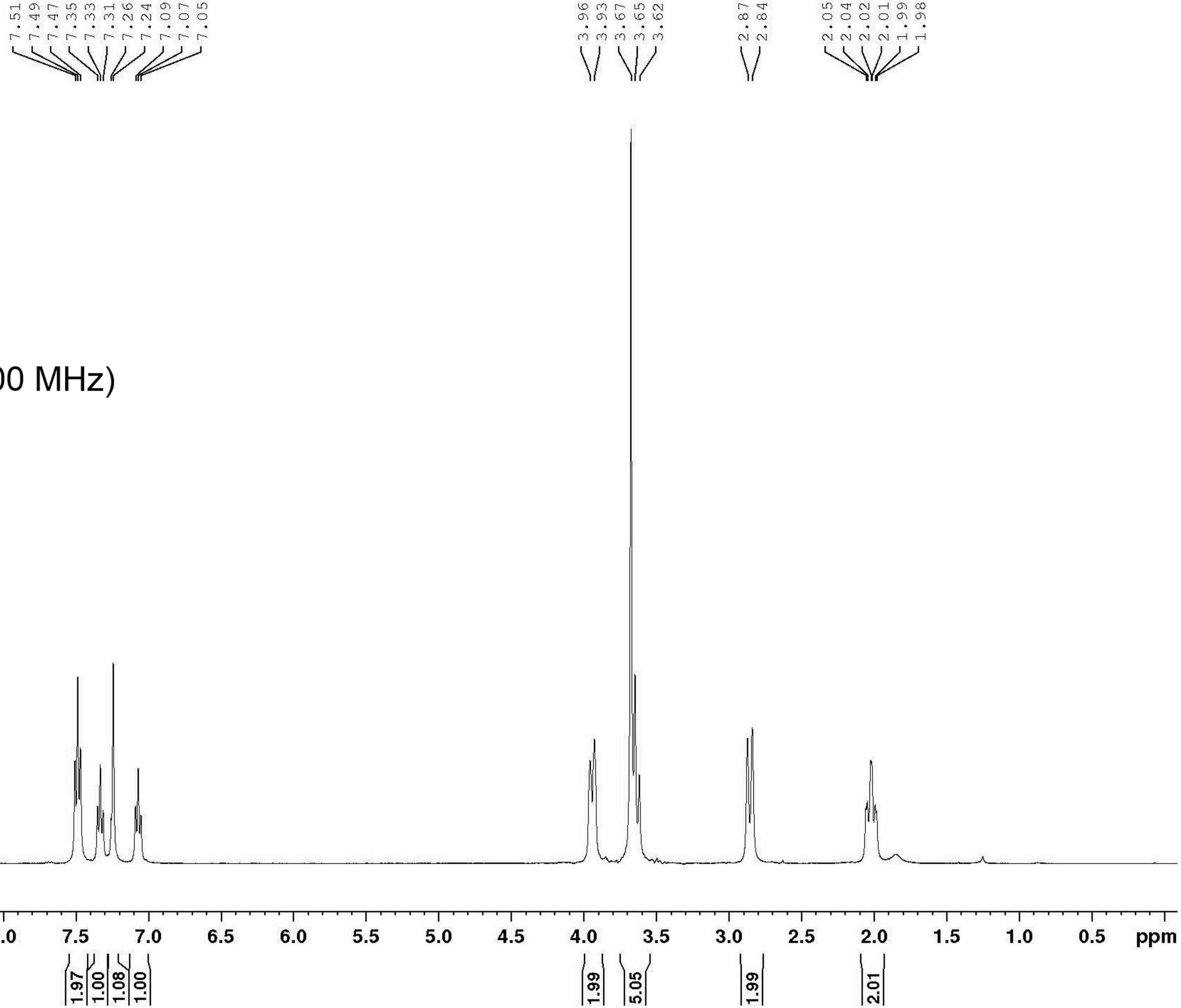

1m

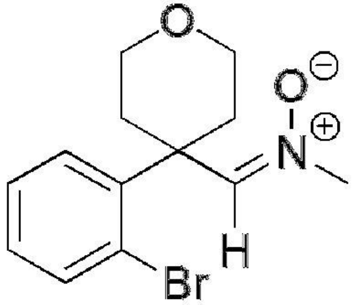

^{13}C NMR ($CDCl_3$, 100 MHz)

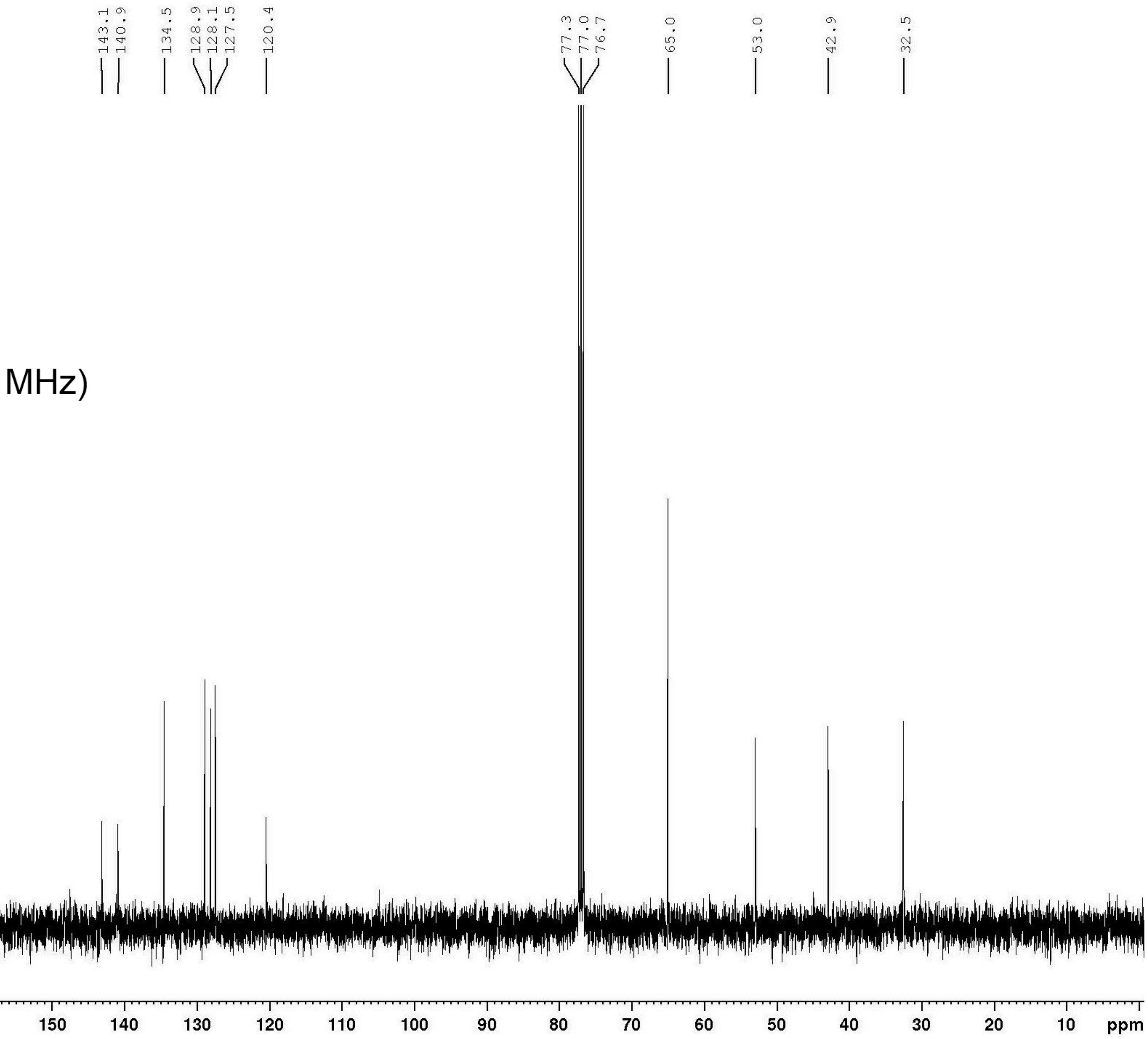

1n

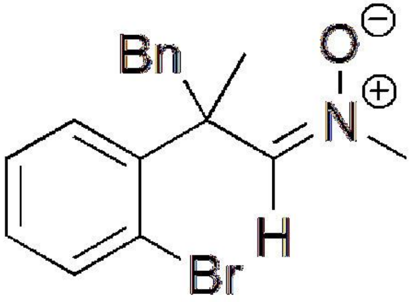

^1H NMR ($CDCl_3$, 400 MHz)

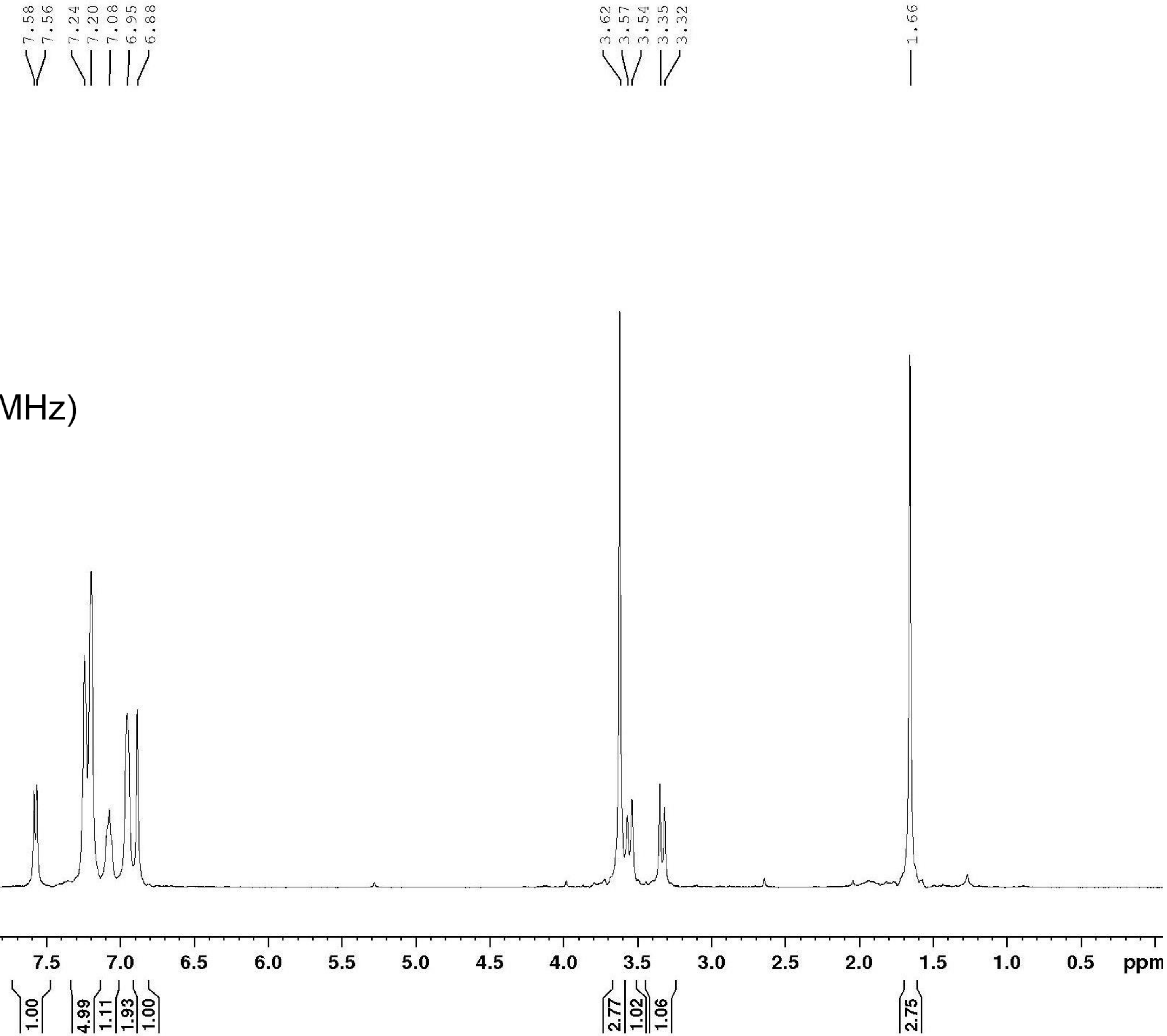

1n

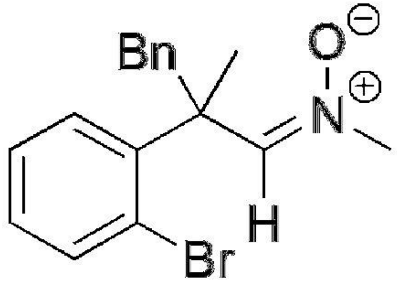

^{13}C NMR ($CDCl_3$, 100 MHz)

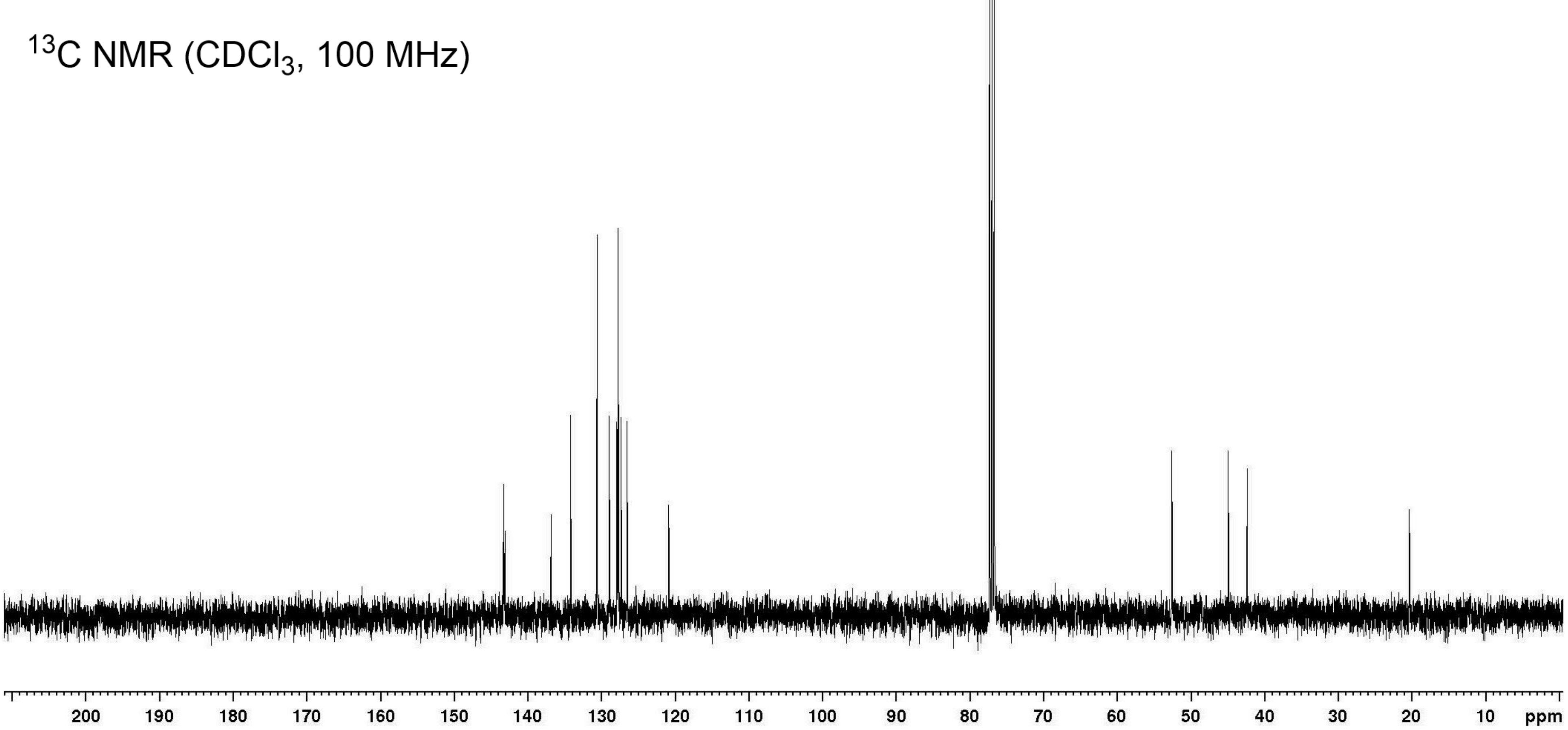

1o

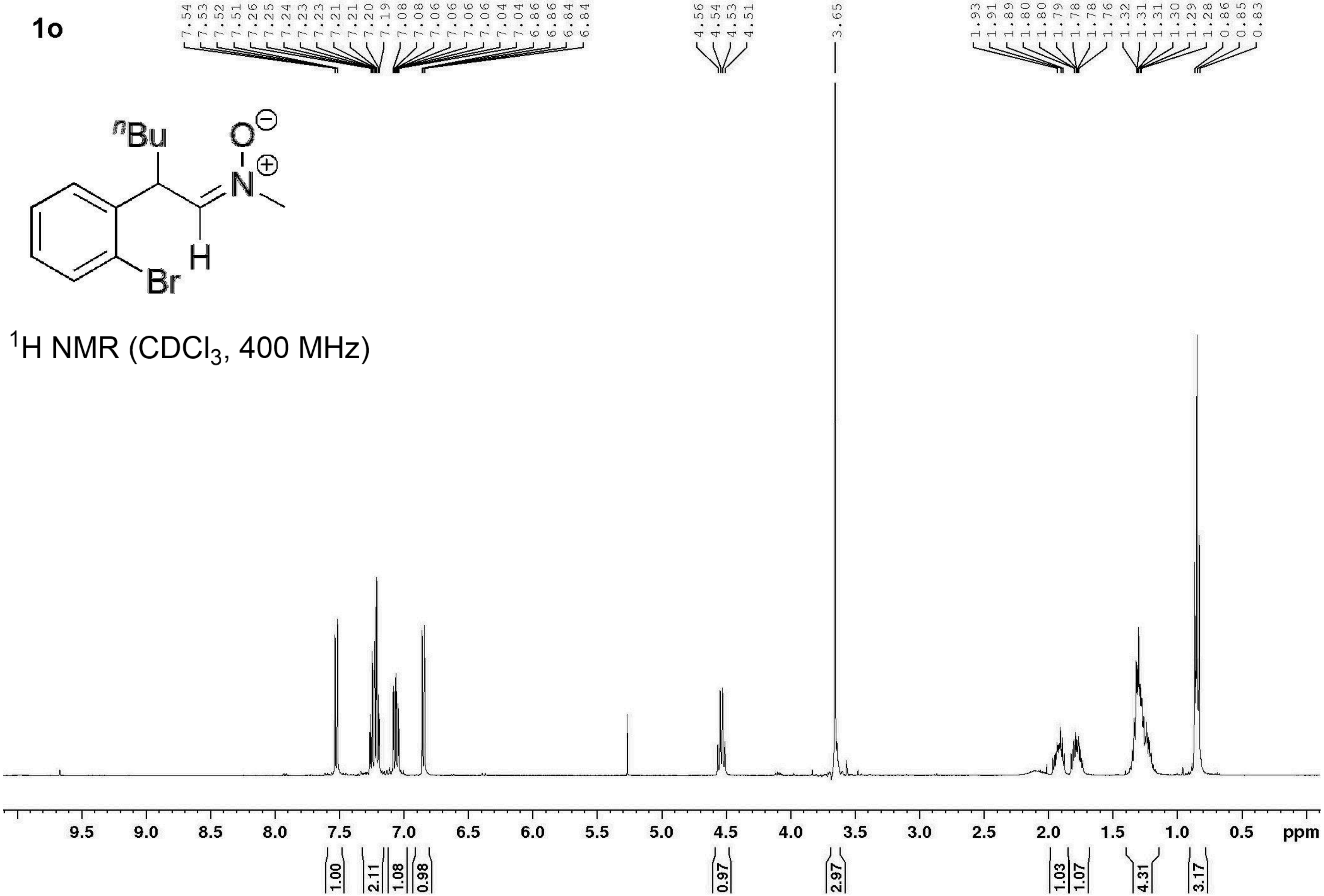

^{1}H NMR ($CDCl_3$, 400 MHz)

1o

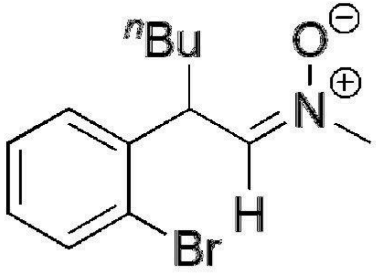

^{13}C NMR ($CDCl_3$, 100 MHz)

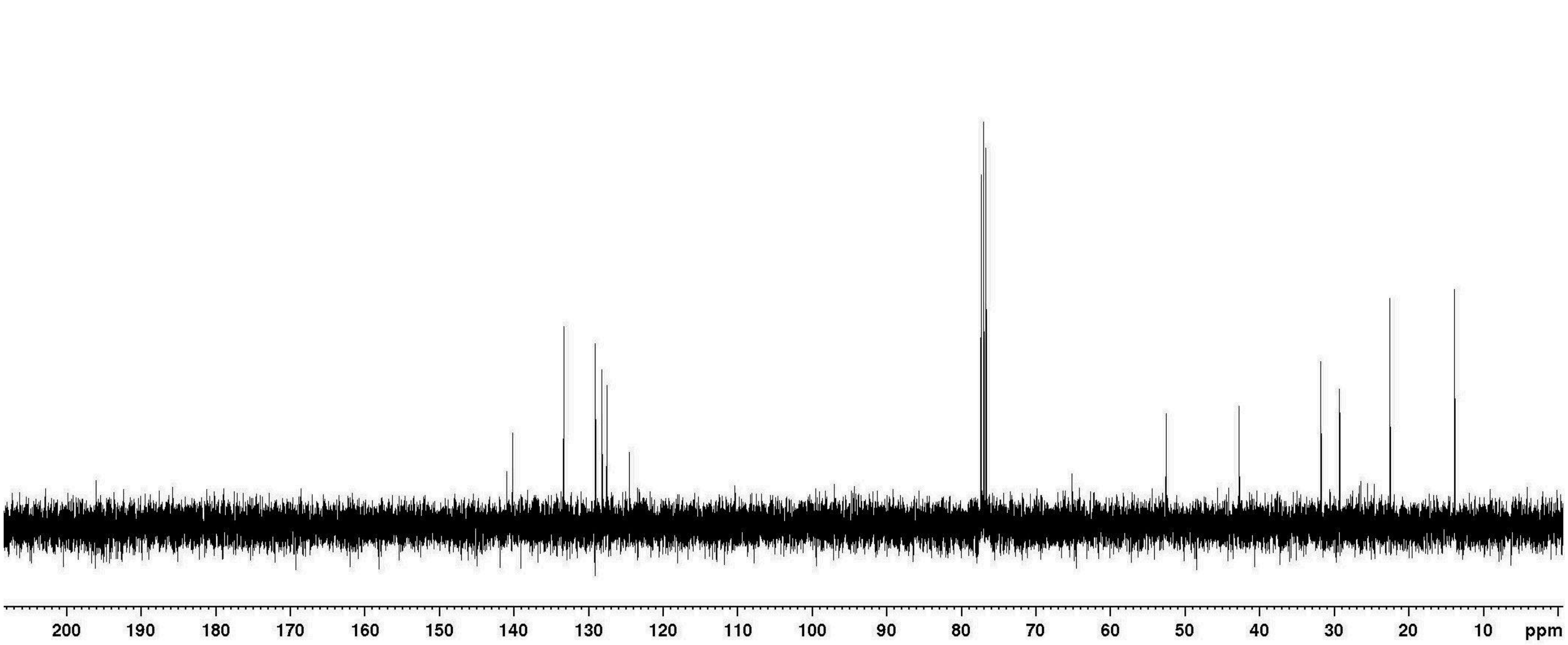

1p

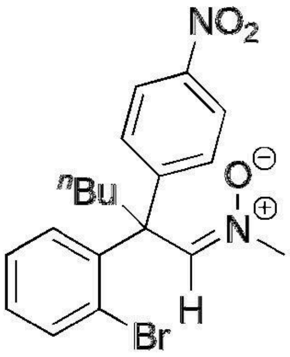

^{1}H NMR ($CDCl_3$, 400 MHz)

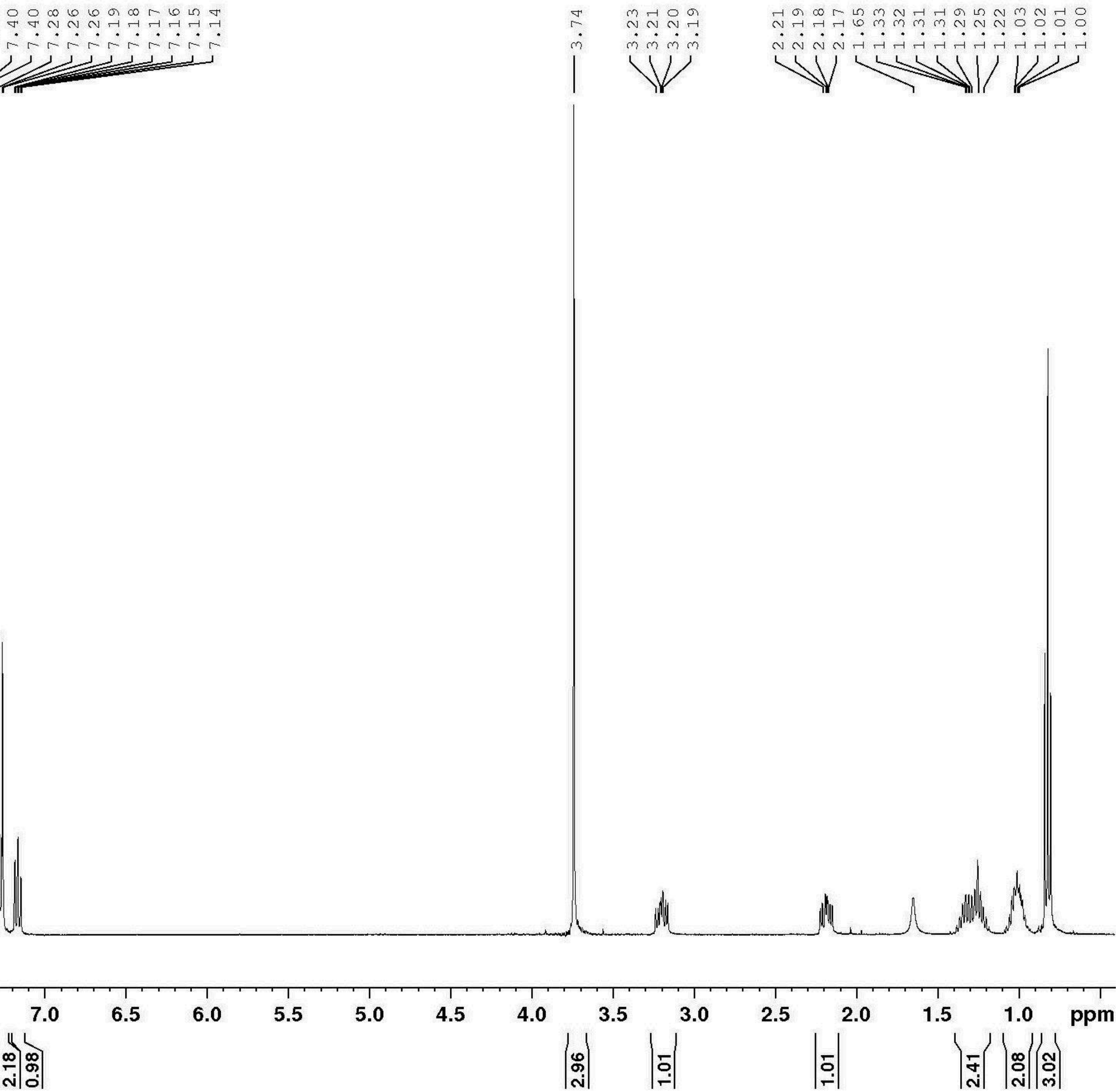

1p

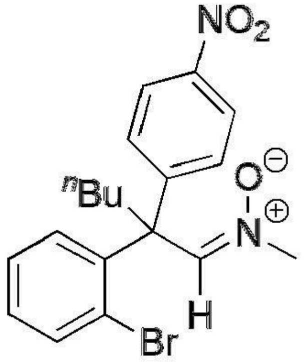

^{13}C NMR ($CDCl_3$, 100 MHz)

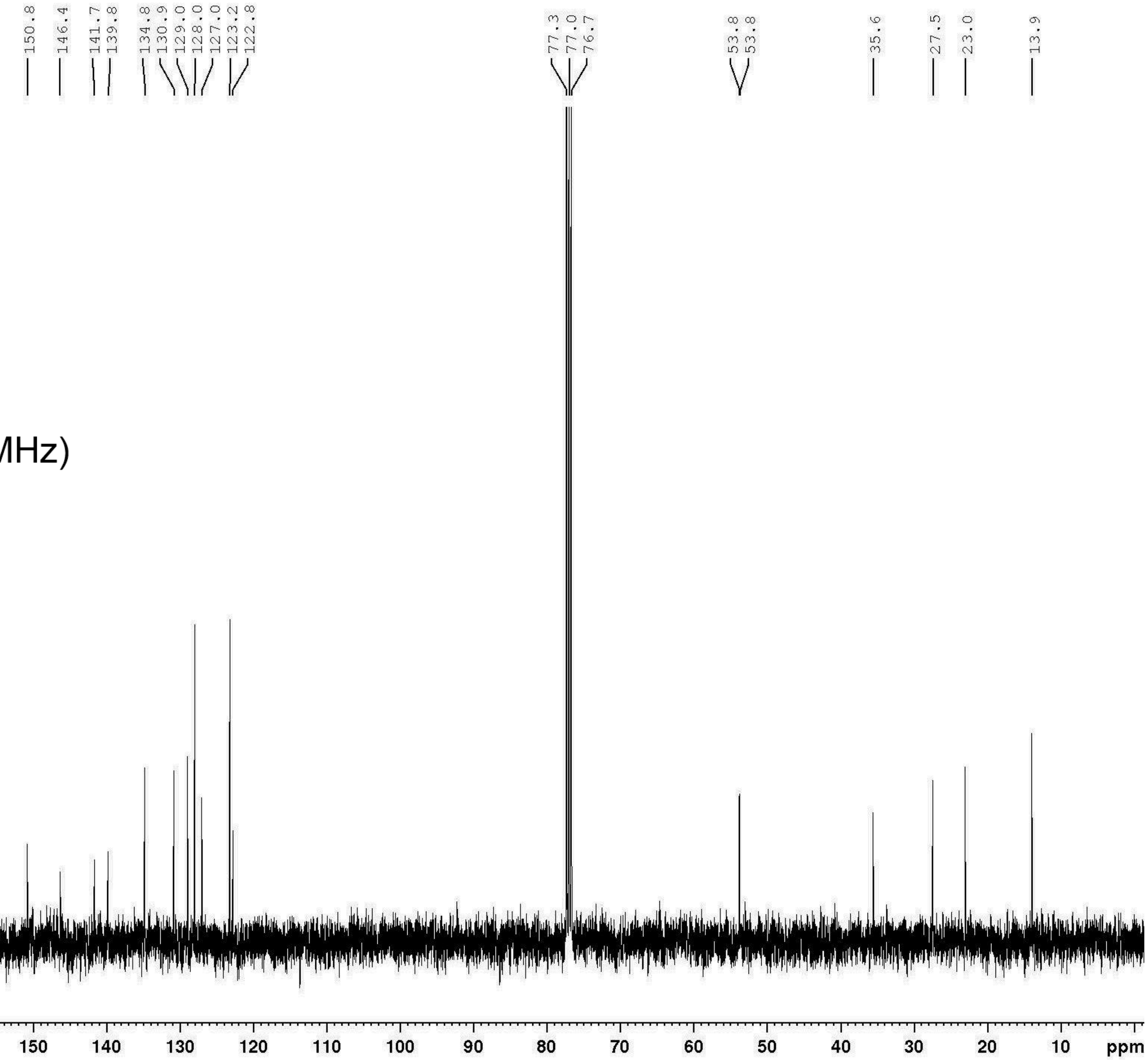

1q

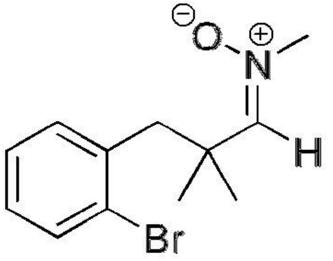

^1H NMR ($CDCl_3$, 400 MHz)

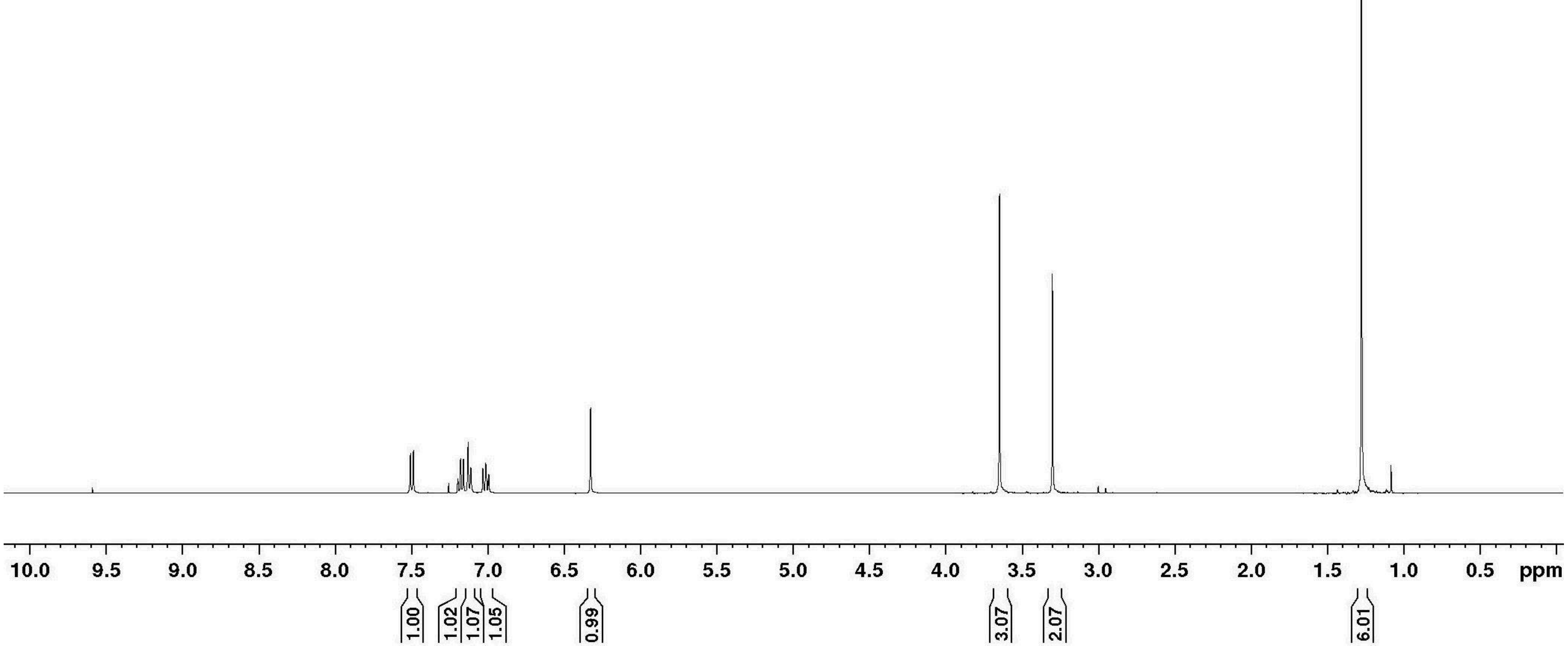

1q

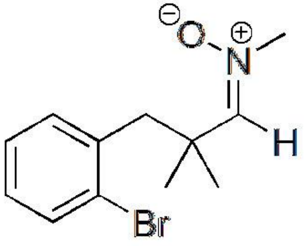

^{13}C NMR ($CDCl_3$, 100 MHz)

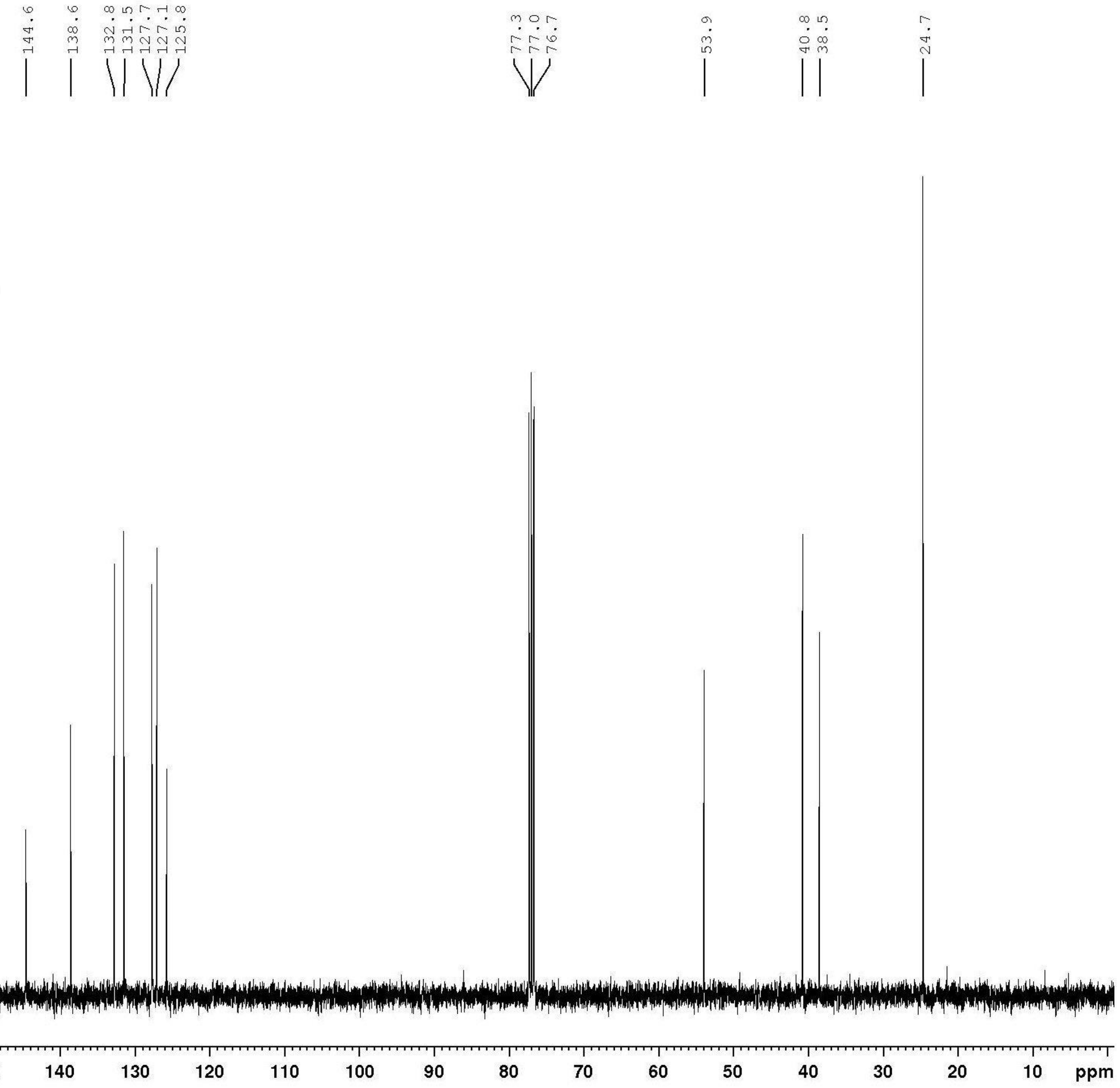

1r

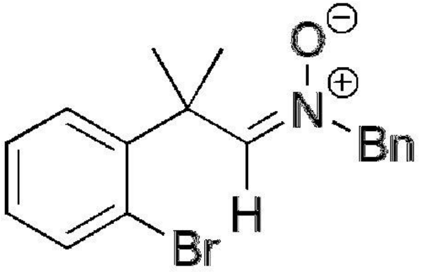

^{1}H NMR ($CDCl_3$, 400 MHz)

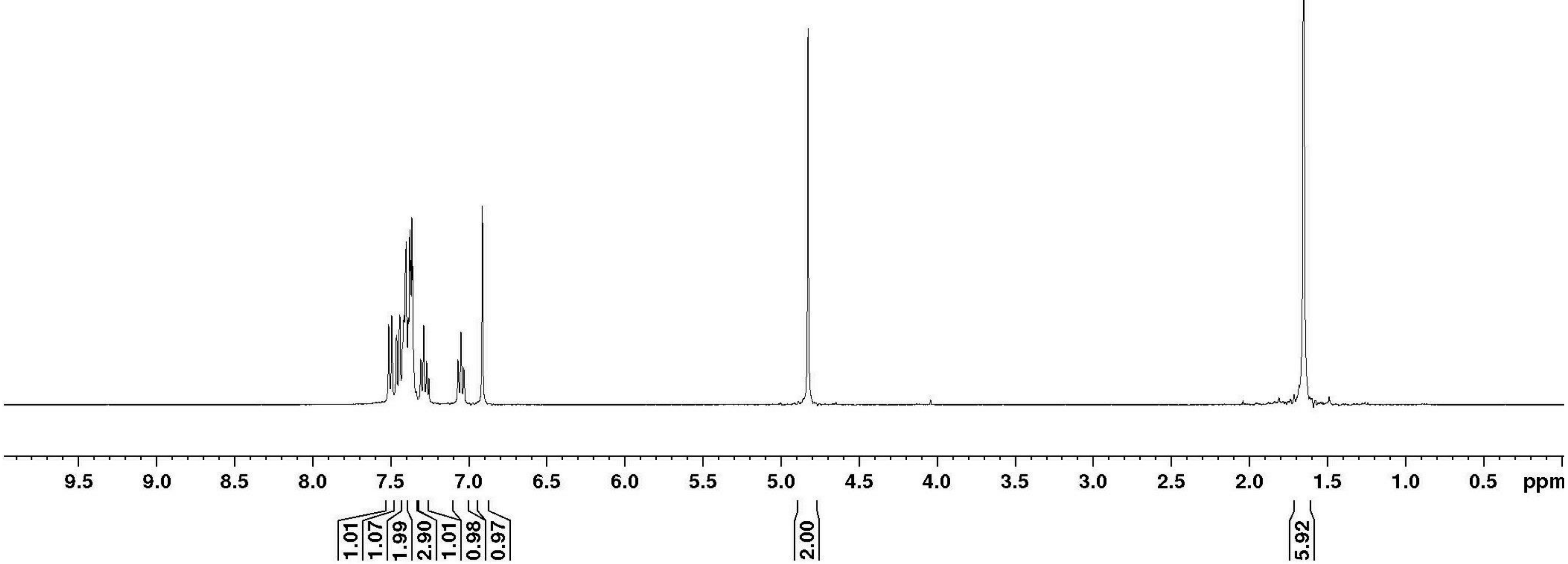

1r

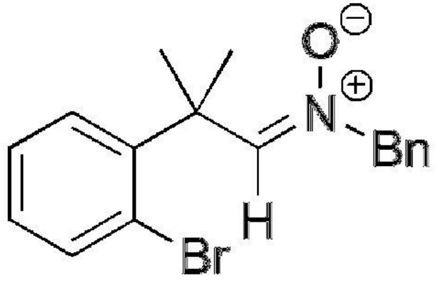

^{13}C NMR ($CDCl_3$, 100 MHz)

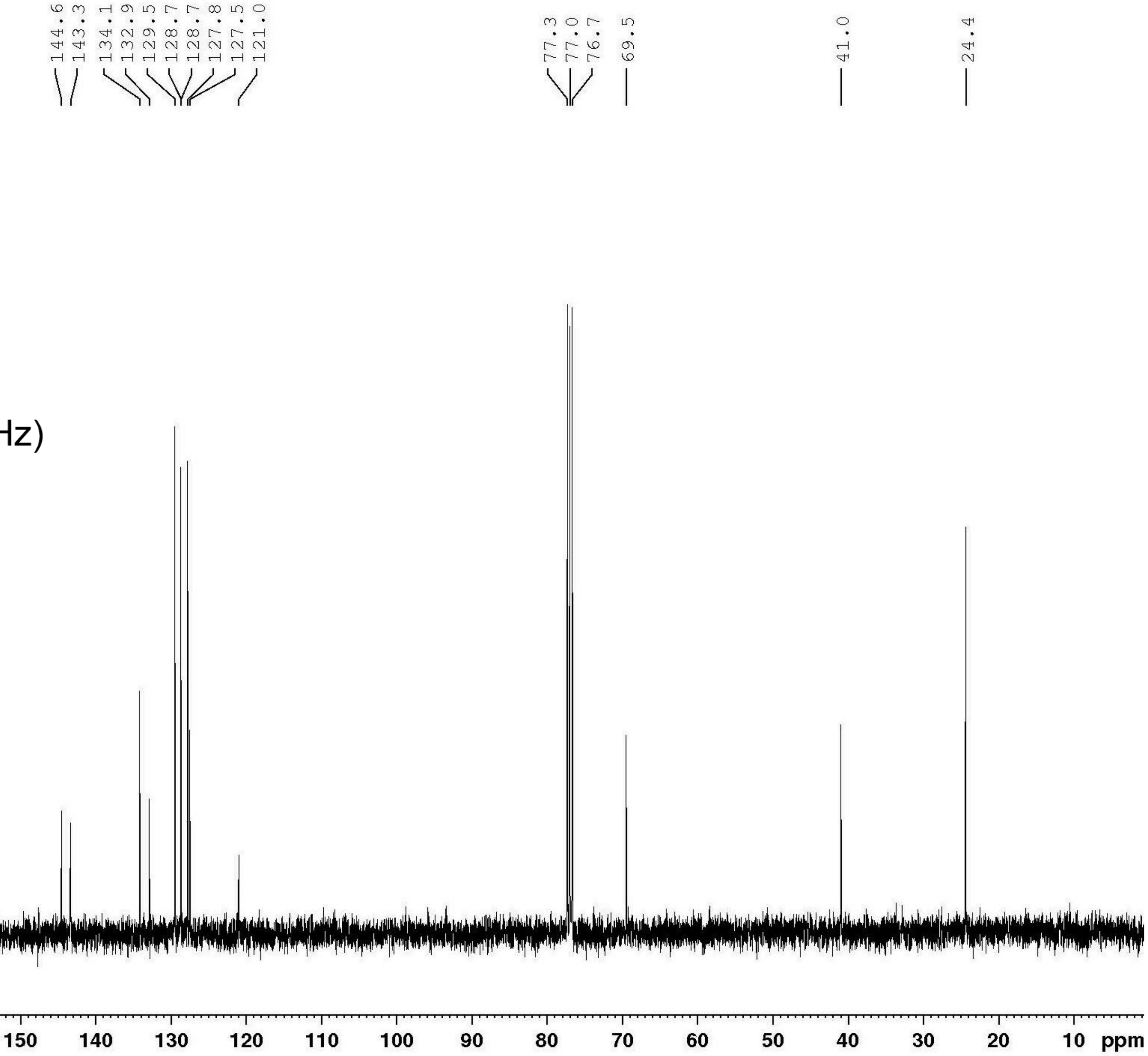

1s

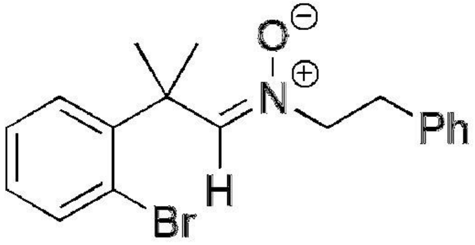

^{1}H NMR ($CDCl_3$, 400 MHz)

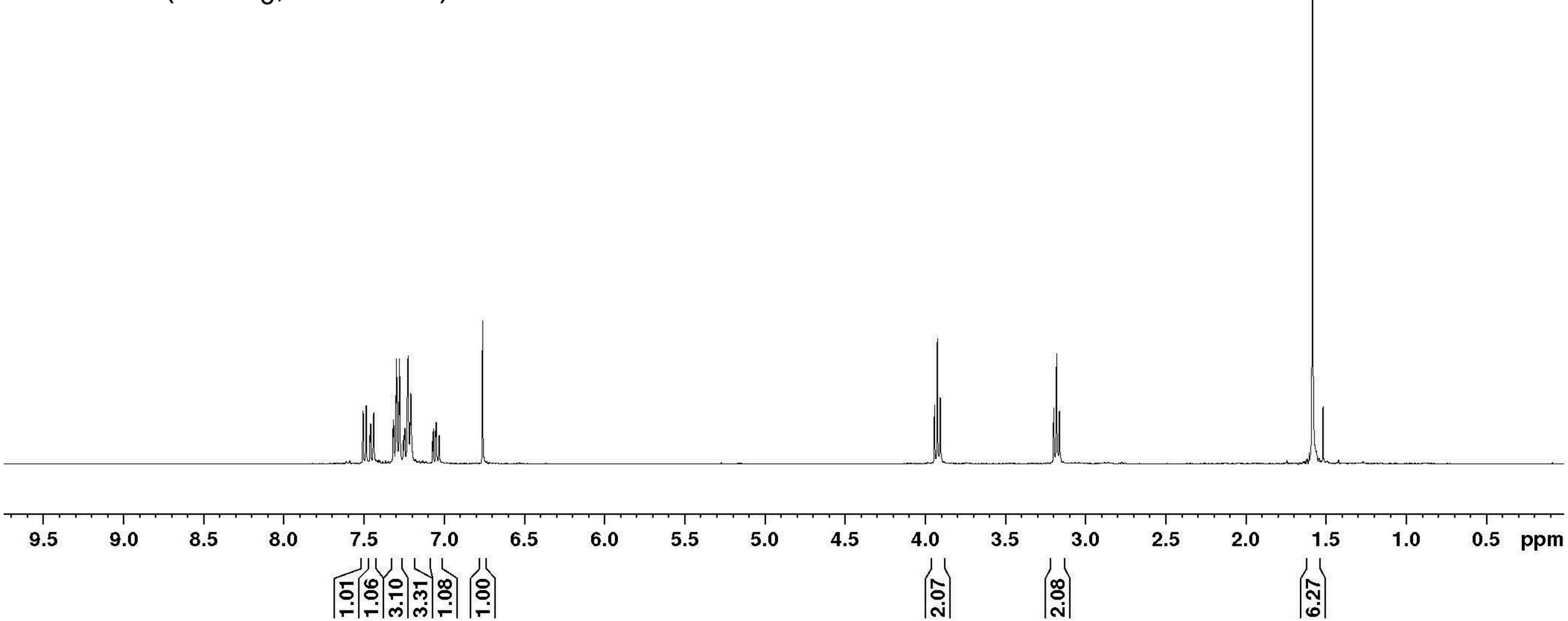

1s

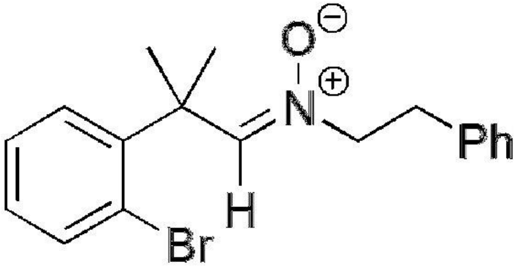

^{13}C NMR ($CDCl_3$, 100 MHz)

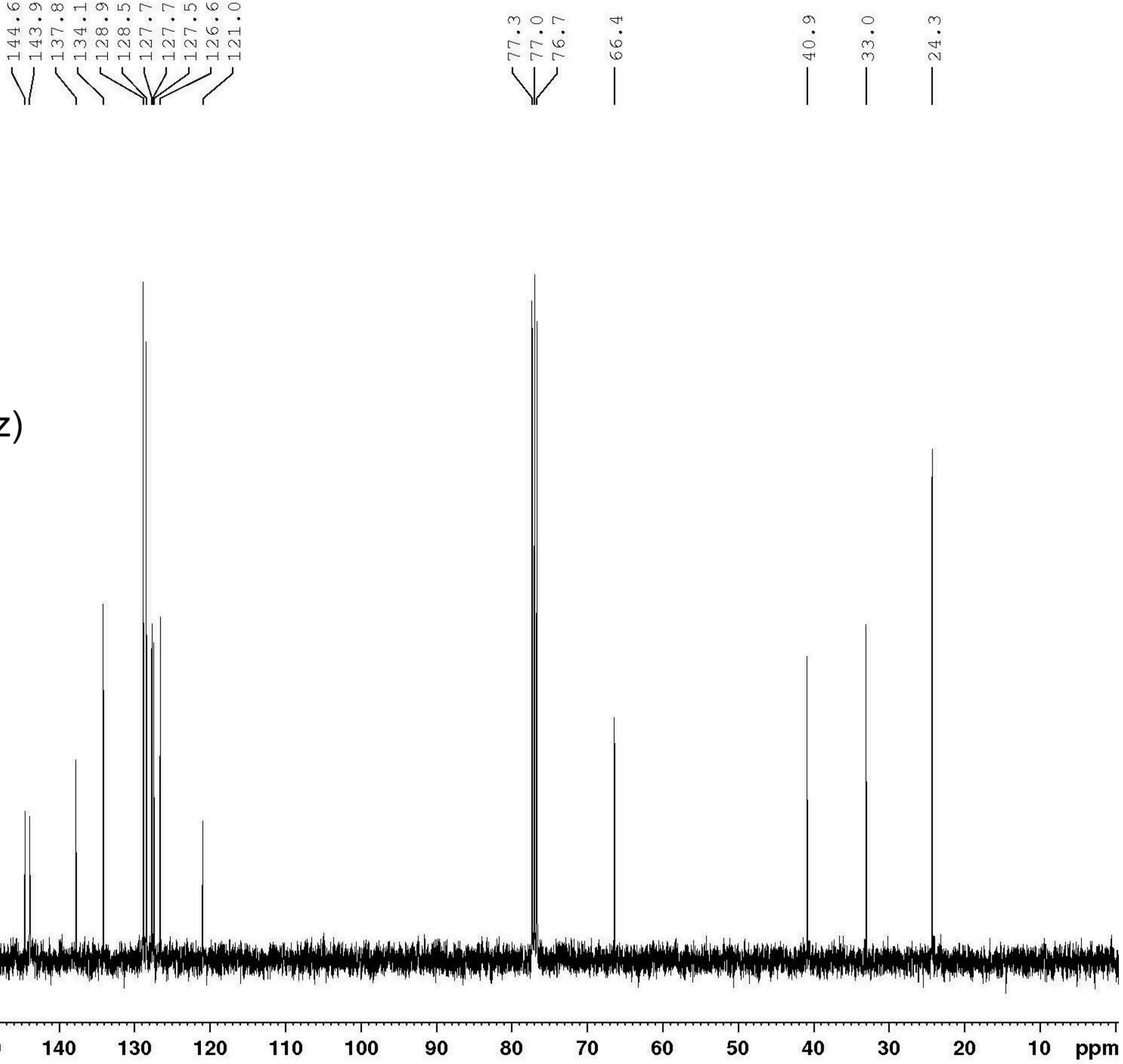

1t

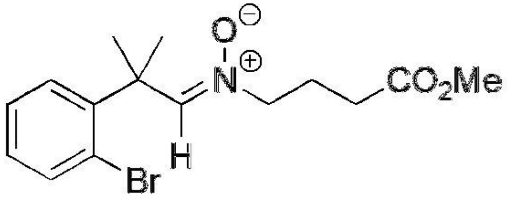

^1H NMR ($CDCl_3$, 400 MHz)

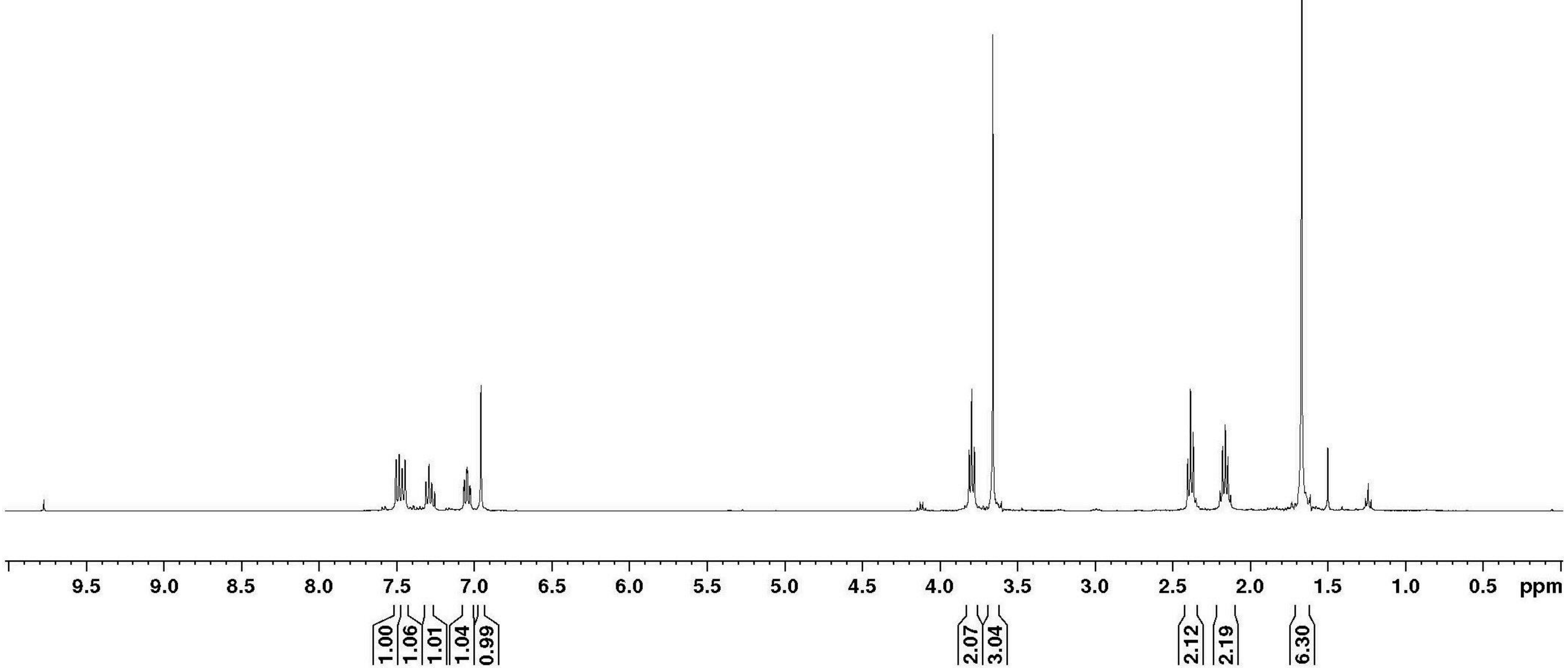

1t

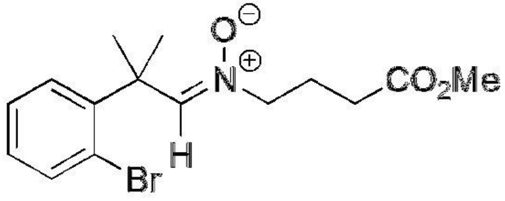

^{13}C NMR ($CDCl_3$, 100 MHz)

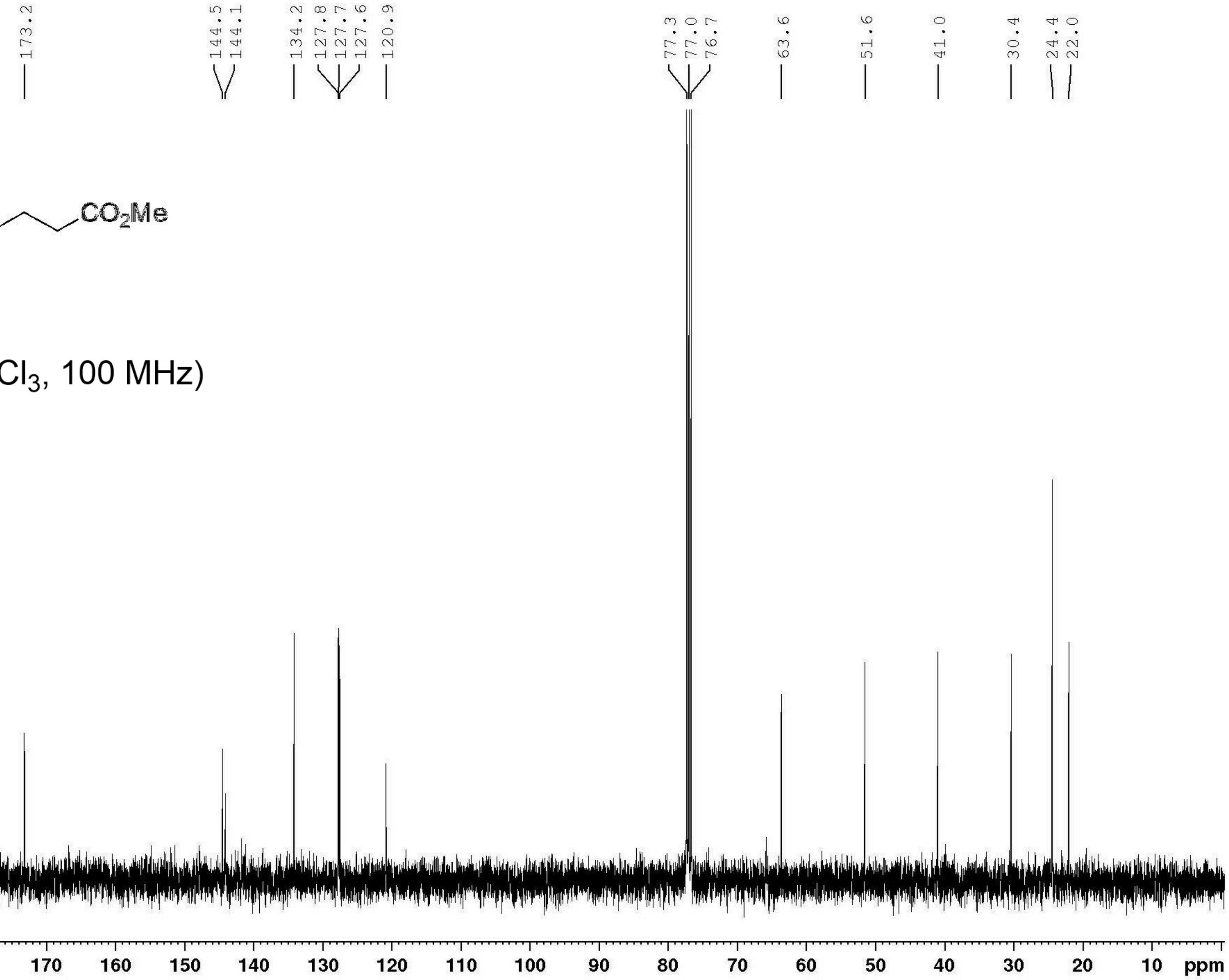

1u

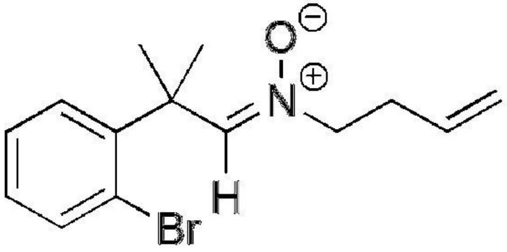

^{1}H NMR ($CDCl_3$, 400 MHz)

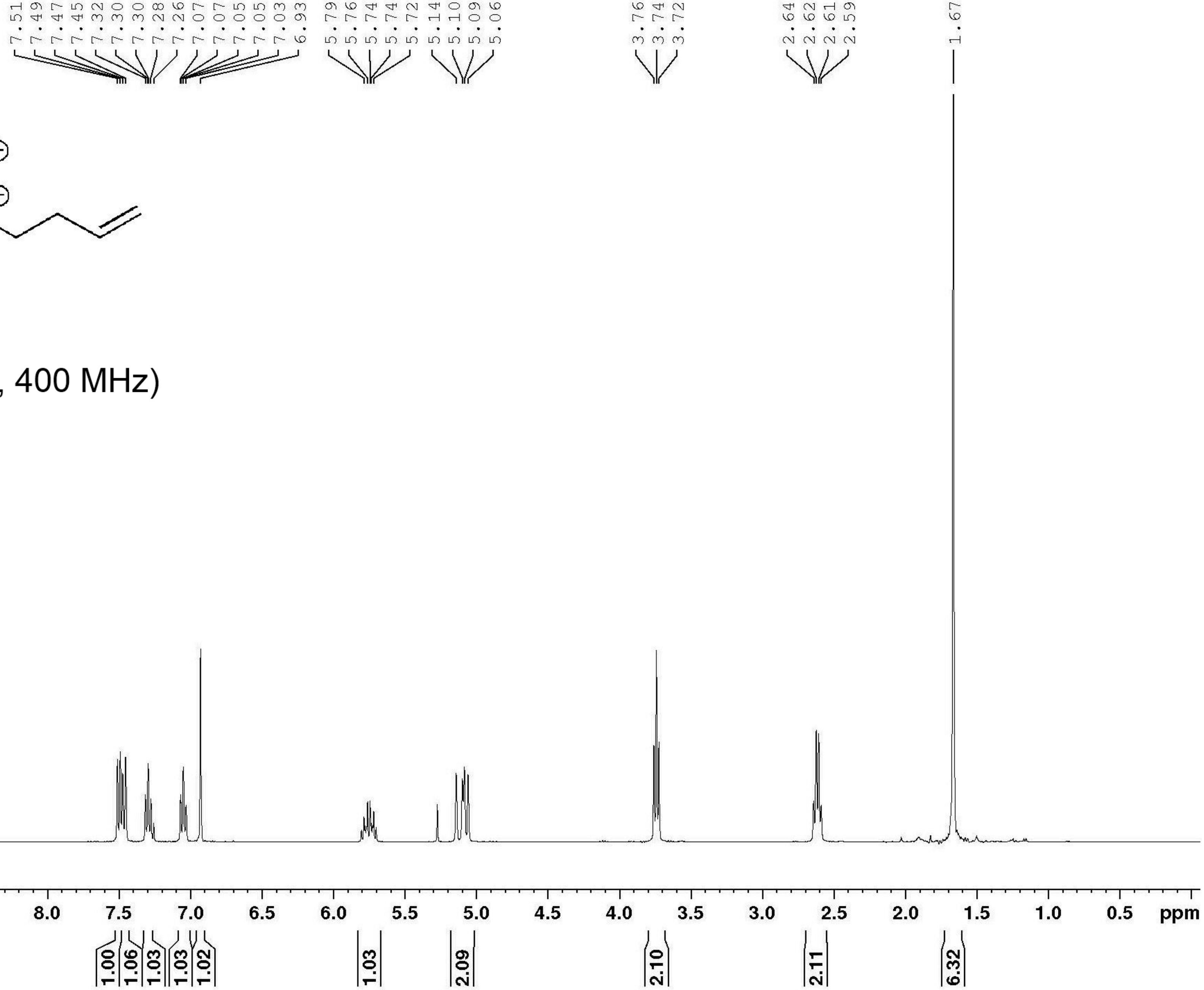

1u

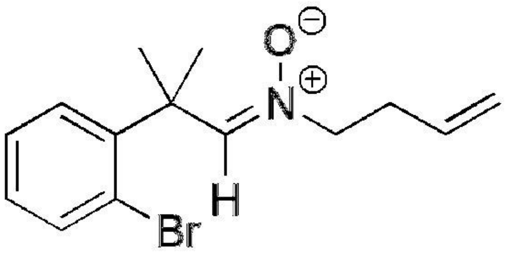

^{13}C NMR ($CDCl_3$, 100 MHz)

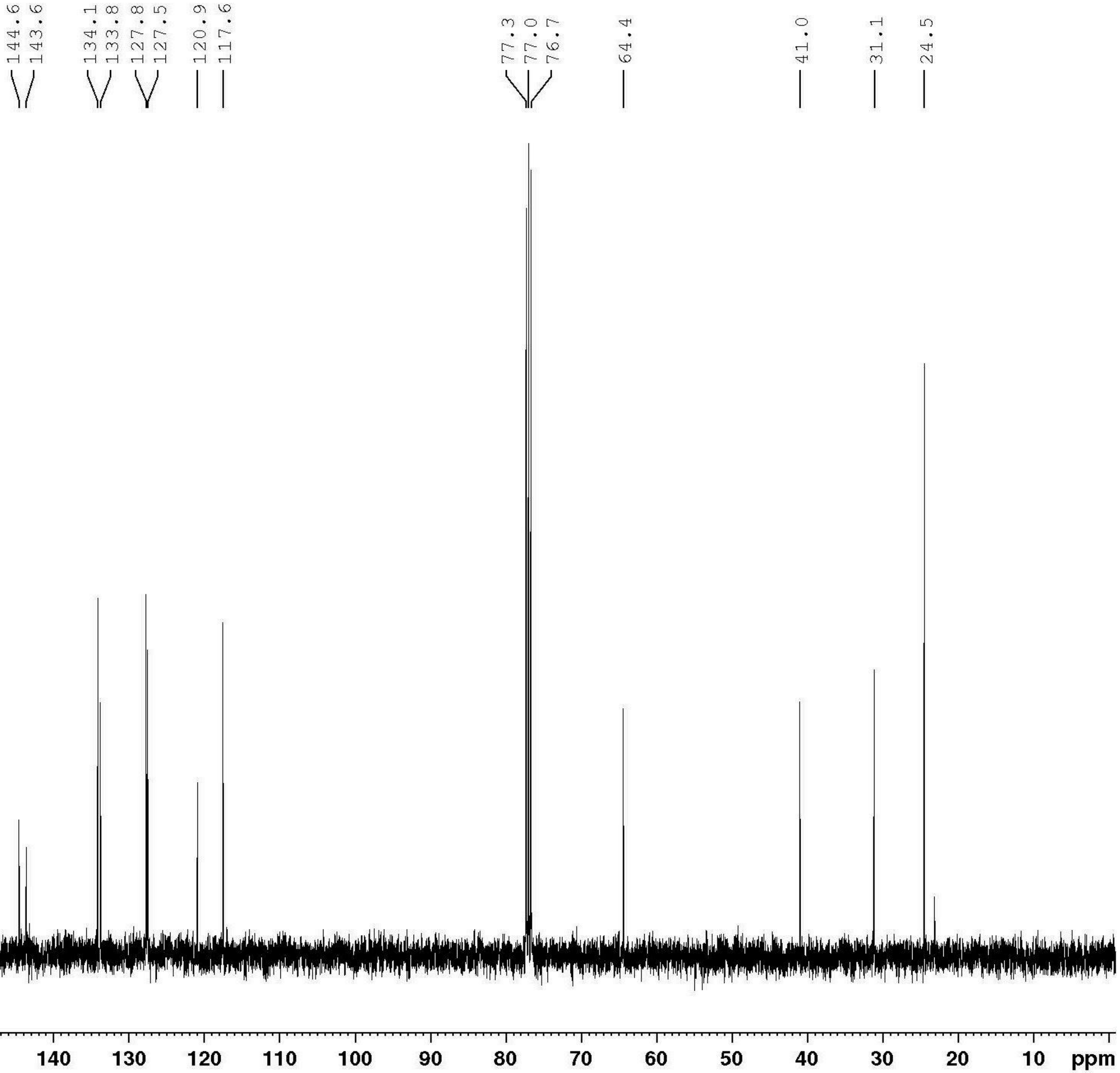

1v

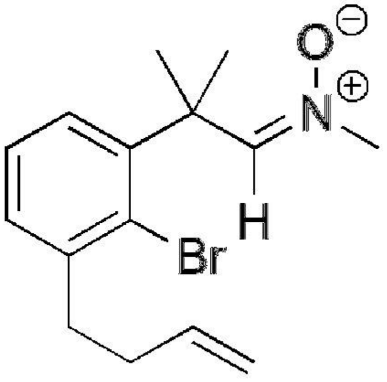

^{1}H NMR ($CDCl_3$, 400 MHz)

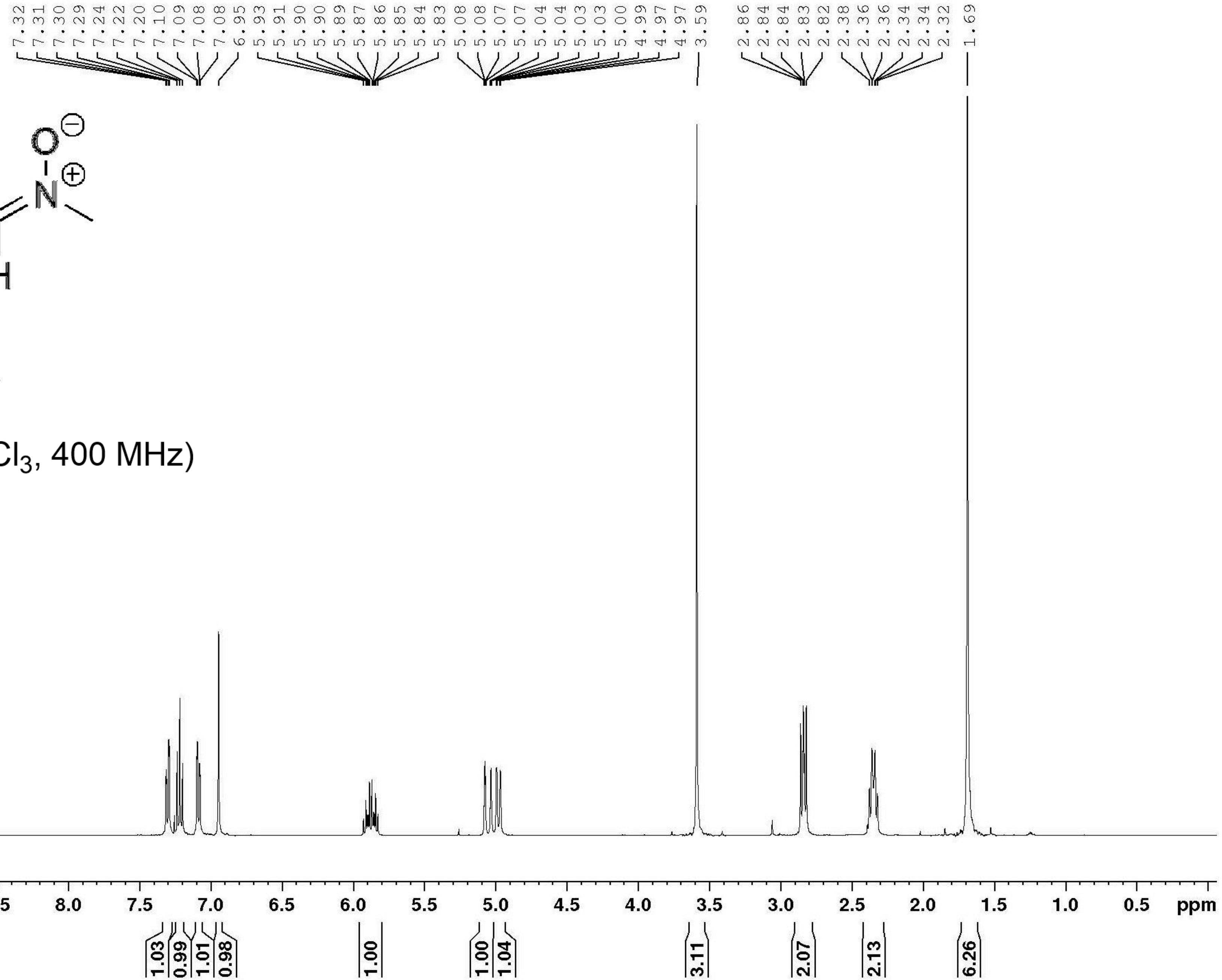

1v

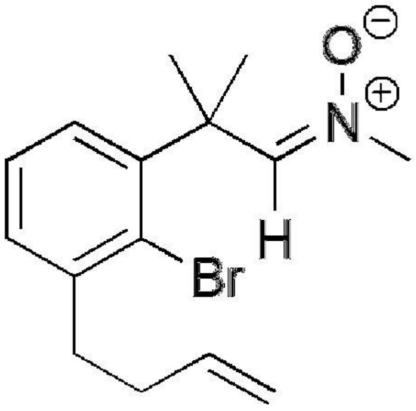

^{13}C NMR ($CDCl_3$, 100 MHz)

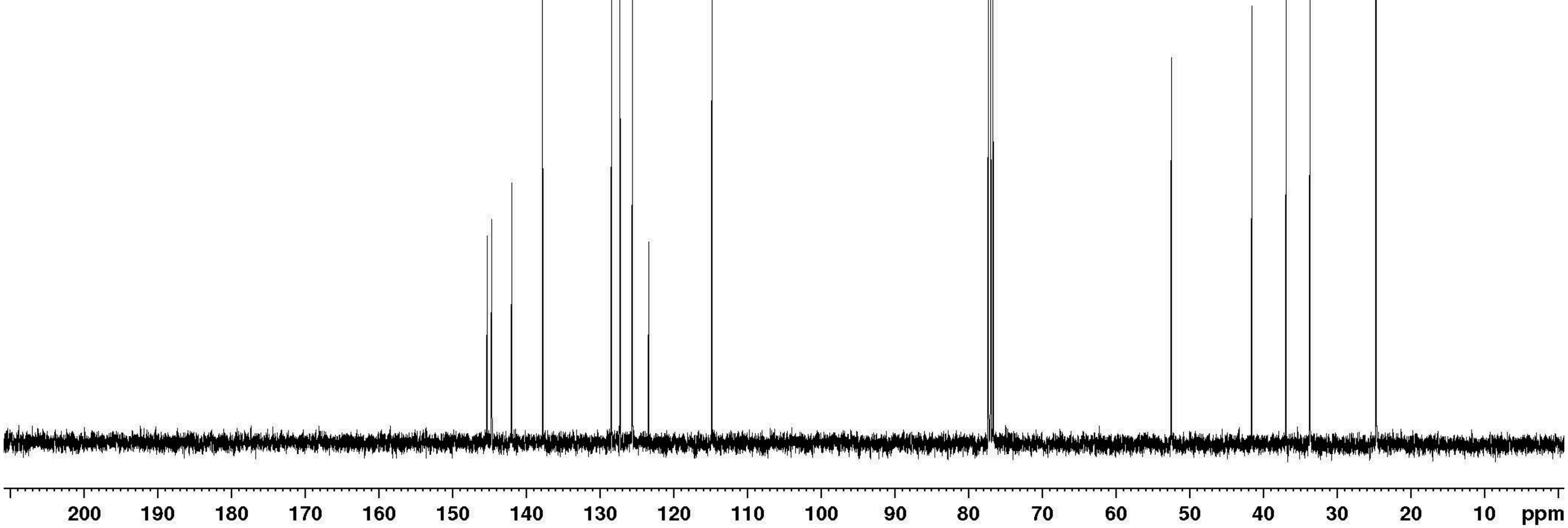

2a

E:Z 2.3:1

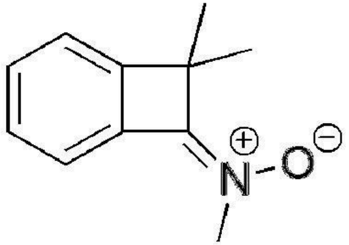

^{1}H NMR ($CDCl_3$, 500 MHz)

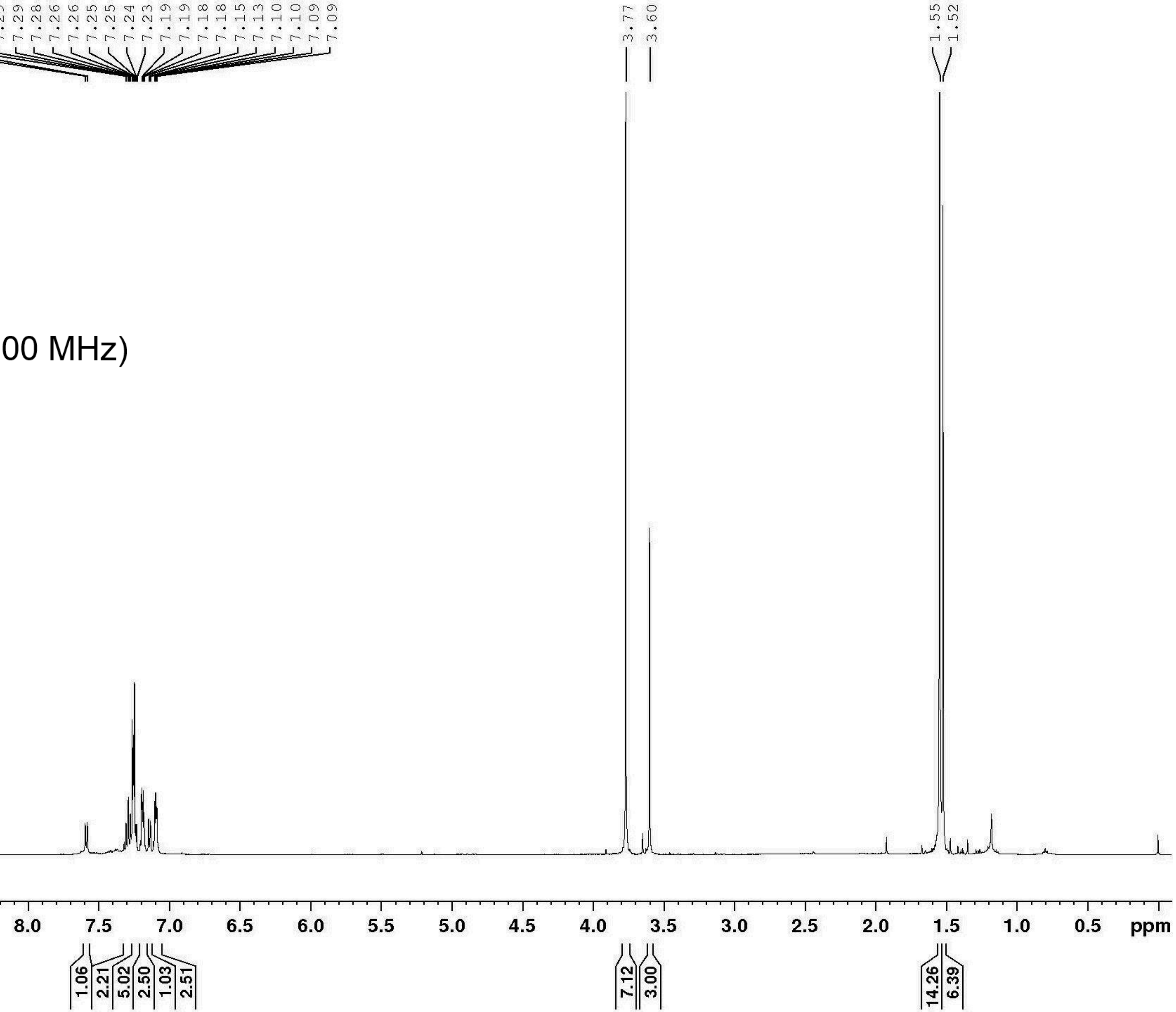

2a *E:Z* 2.3:1

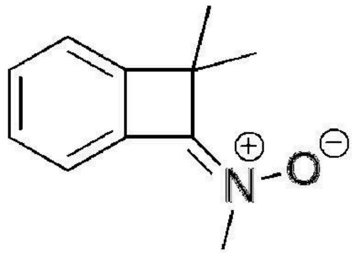

^{13}C NMR ($CDCl_3$, 125 MHz)

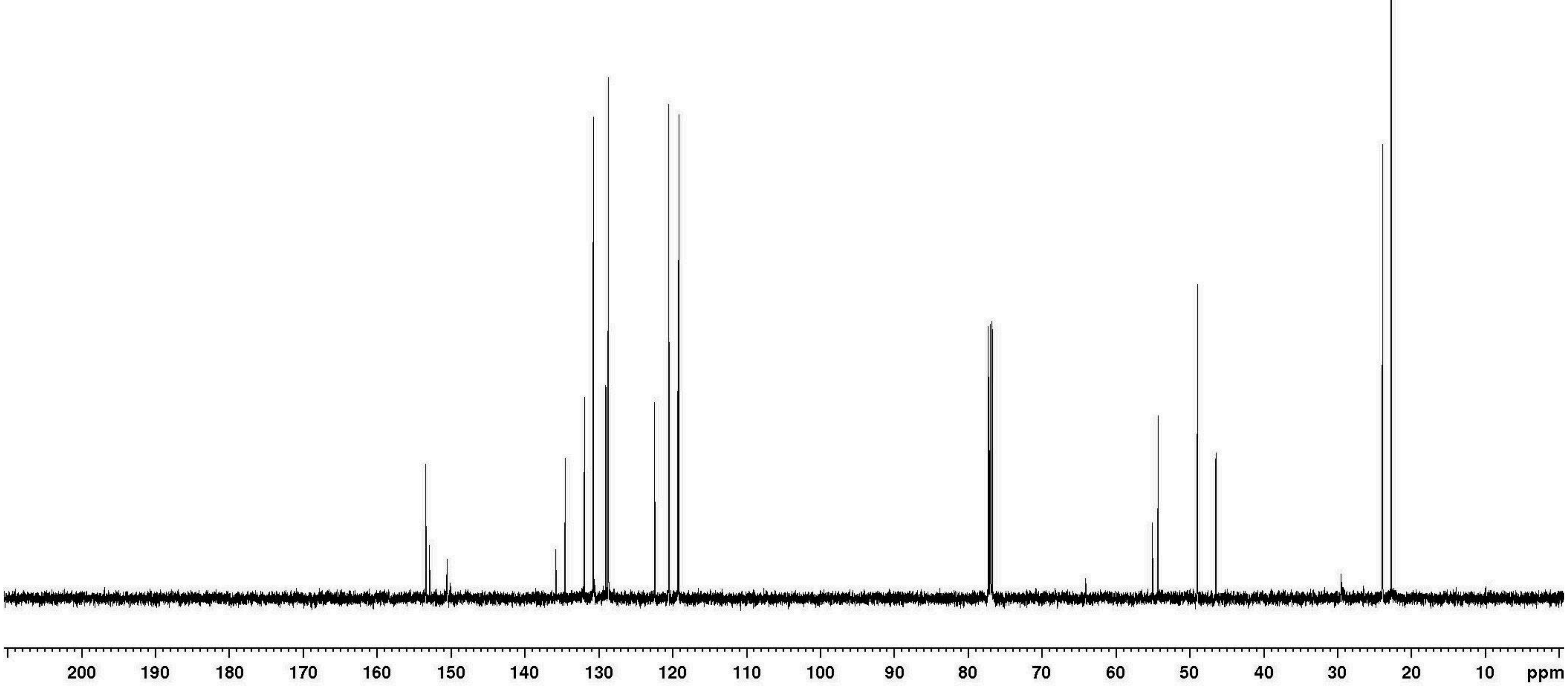

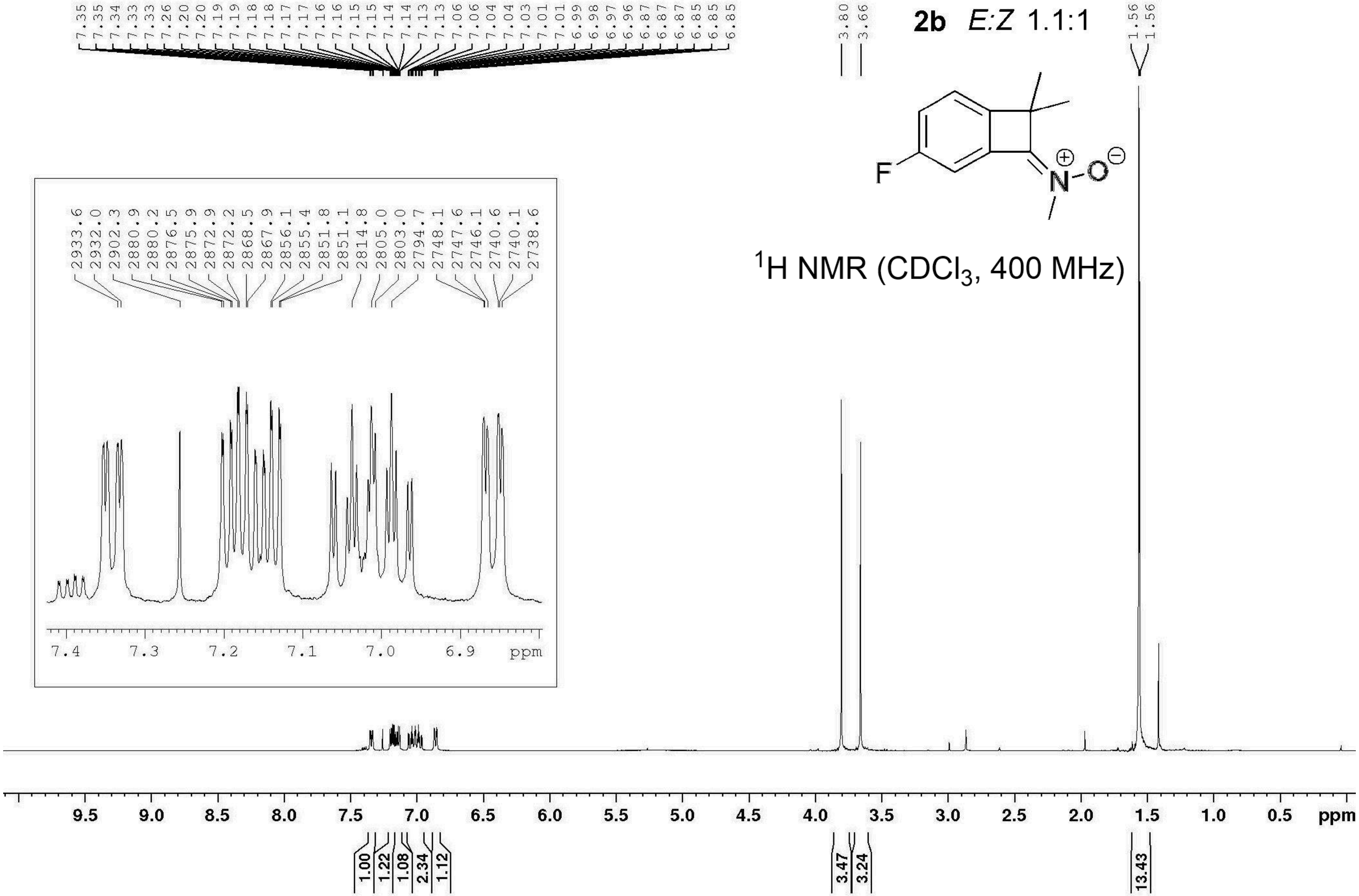
7.35 7.35 7.34 7.33 7.33 7.26 7.20 7.20 7.19 7.19 7.18 7.18 7.17 7.17 7.16 7.16 7.15 7.15 7.14 7.14 7.13 7.13 7.06 7.06 7.04 7.04 7.03 7.01 7.01 6.99 6.98 6.97 6.96 6.87 6.87 6.85 6.85 6.85
3.80 3.66
2b E:Z 1.1:1
1.56 1.56
F
N
O
1H NMR (CDCl3, 400 MHz)
2933.6 2932.0 2902.3 2880.9 2880.2 2876.5 2875.9 2872.9 2872.2 2868.5 2867.9 2856.1 2855.4 2851.8 2851.1 2814.8 2805.0 2803.0 2794.7 2748.1 2747.6 2746.1 2740.6 2740.1 2738.6
7.4 7.3 7.2 7.1 7.0 6.9 ppm
9.5 9.0 8.5 8.0 7.5 7.0 6.5 6.0 5.5 5.0 4.5 4.0 3.5 3.0 2.5 2.0 1.5 1.0 0.5 ppm
1.00 1.22 1.08 2.34 1.12 3.47 3.24 13.43

2b *E*:*Z* 1.1:1

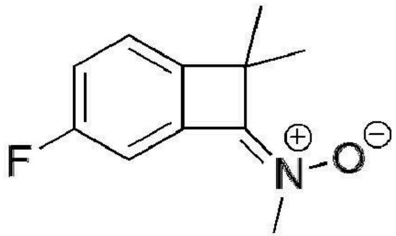

^{13}C NMR ($CDCl_3$, 100 MHz)

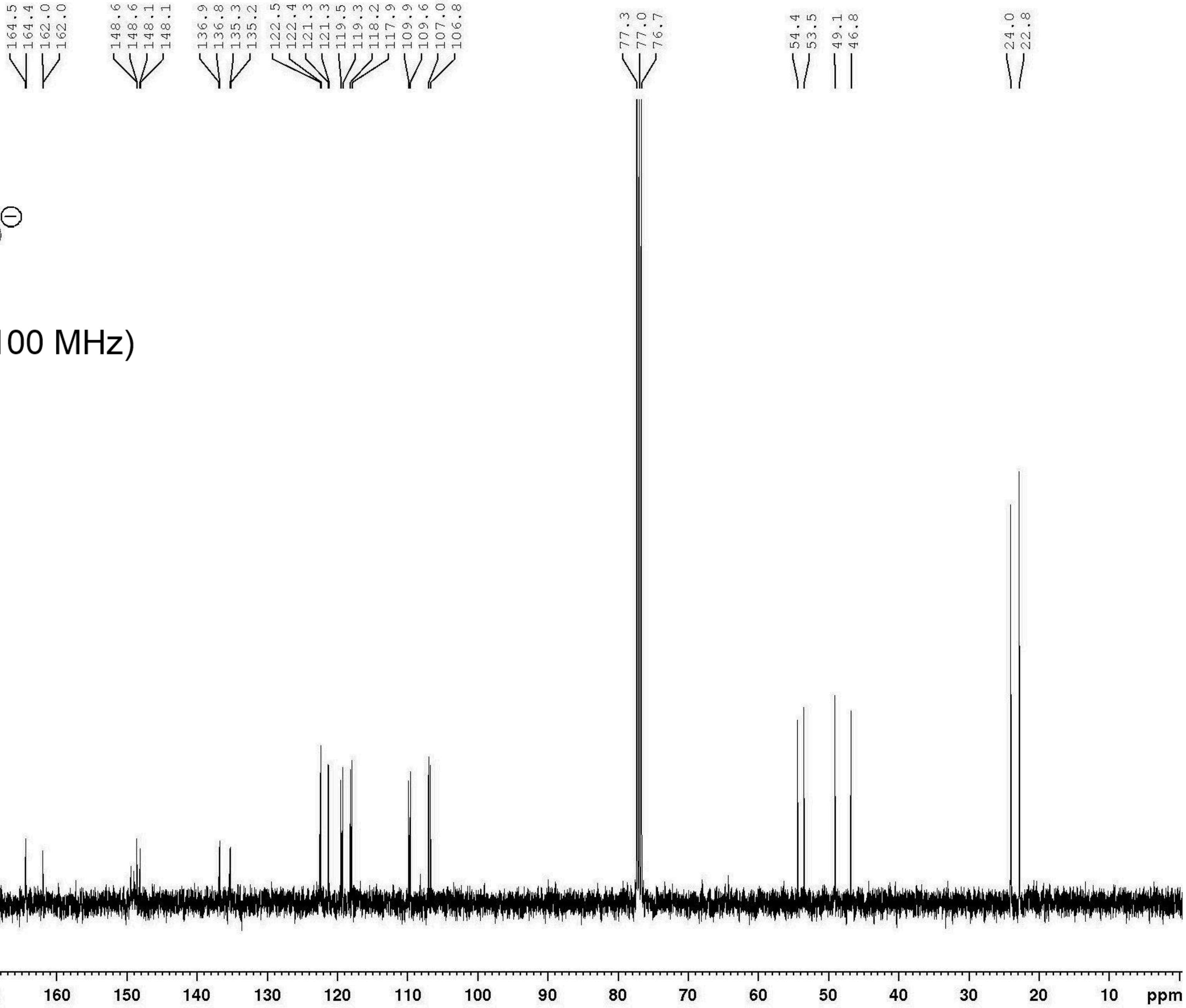

2b

E:Z 1.1:1

^{19}F NMR ($CDCl_3$, 376 MHz)

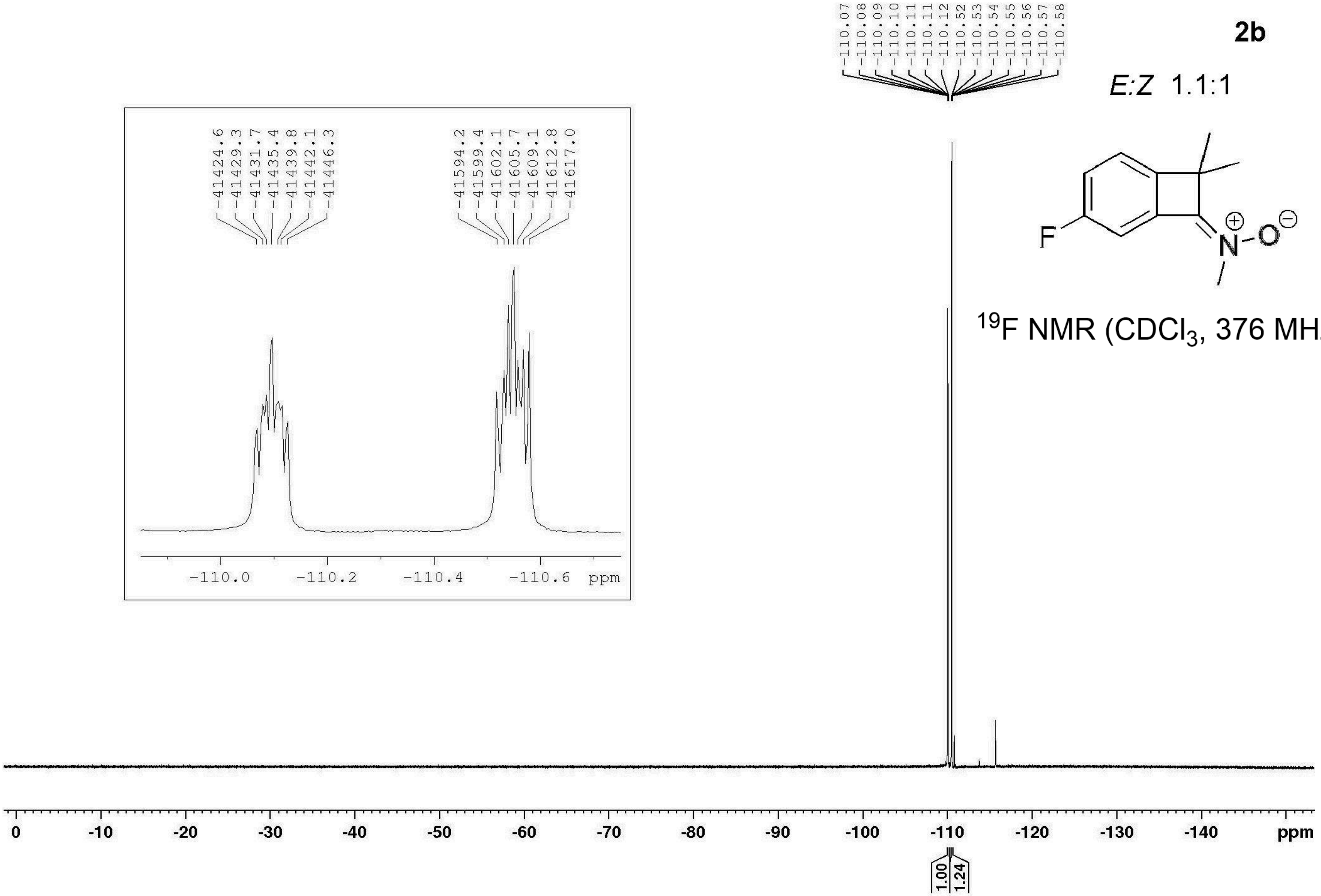

2c

E:*Z* 1:1.2

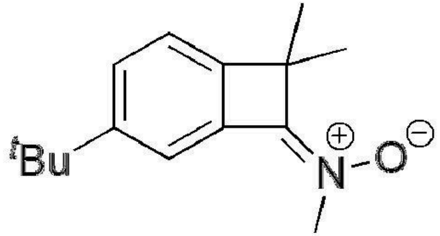

^{1}H NMR ($CDCl_3$, 400 MHz)

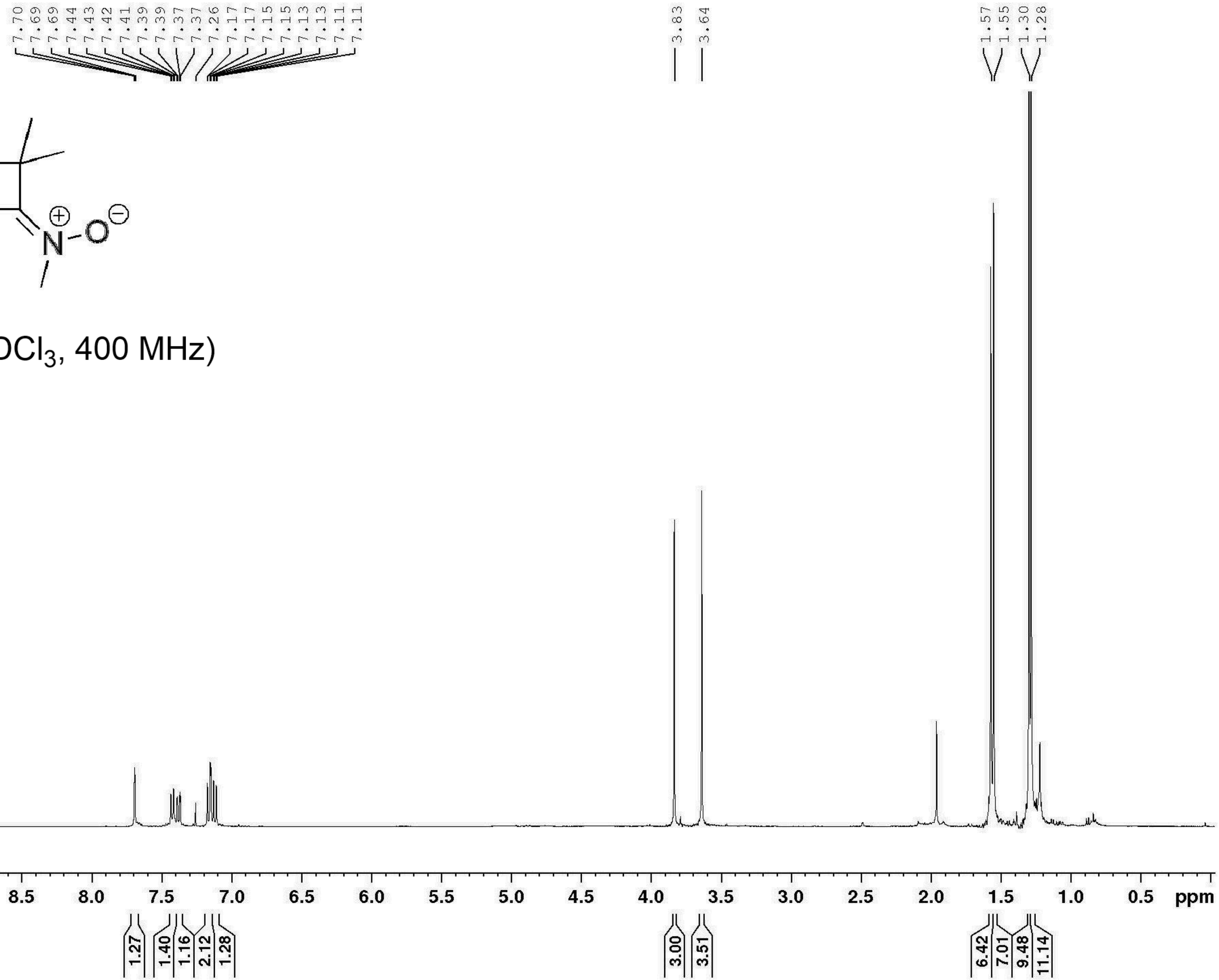

2c *E*:*Z* 1:1.2

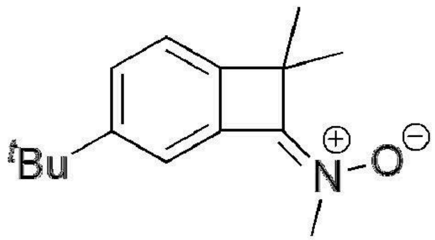

^{13}C NMR ($CDCl_3$, 100 MHz)

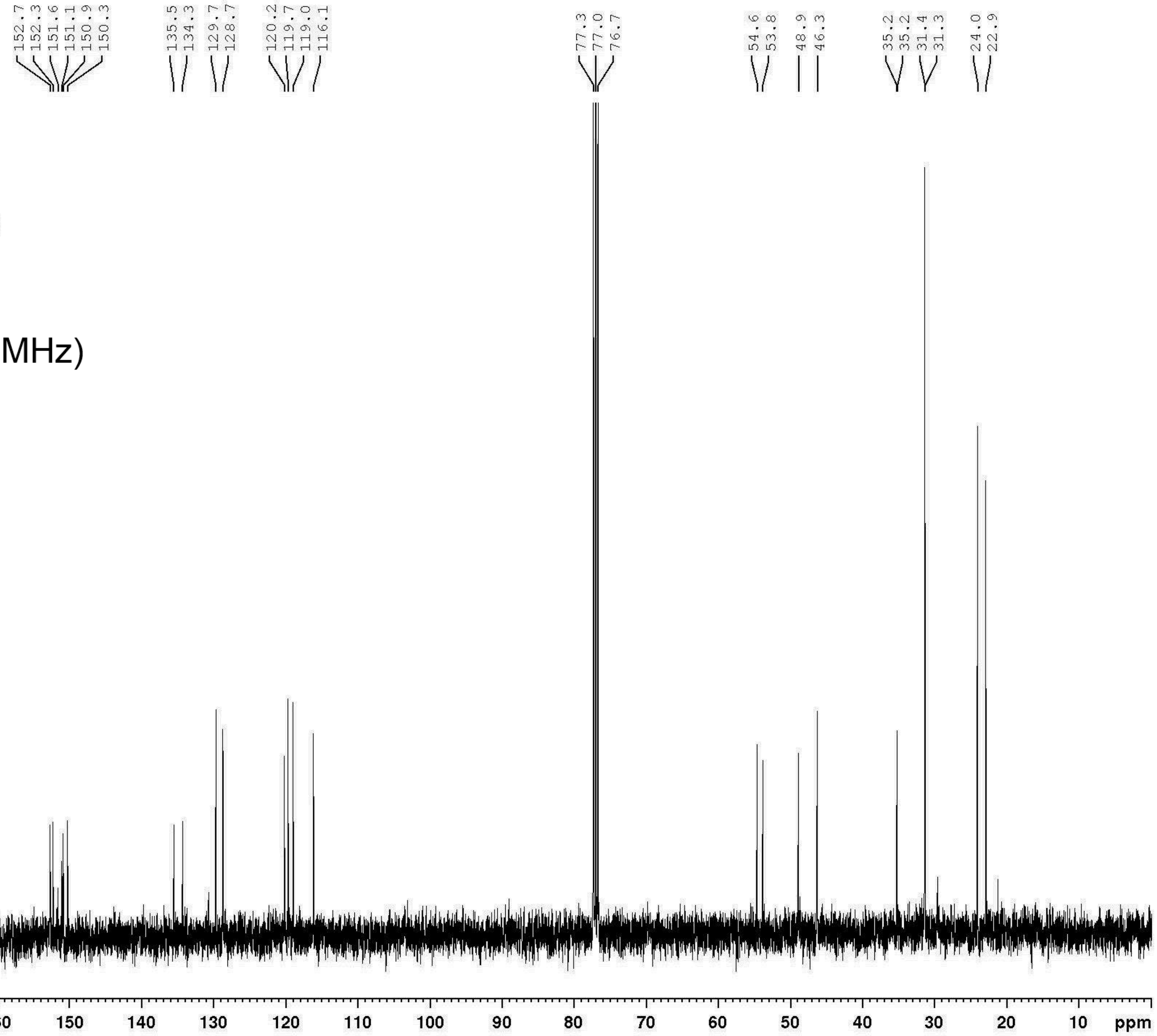

2d

E:Z 1.1:1

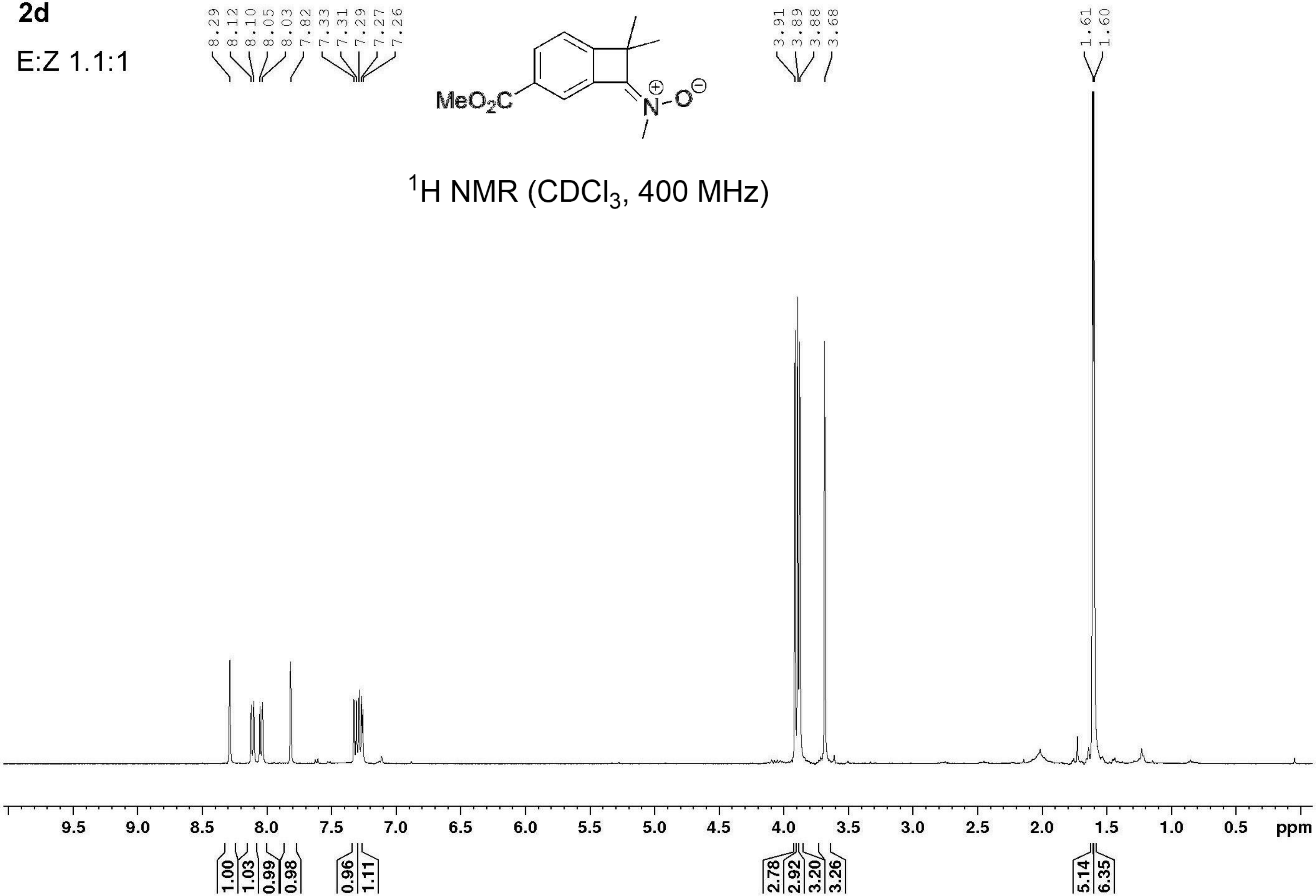

8.29
8.12
8.10
8.05
8.03
7.82
7.33
7.31
7.29
7.27
7.26
3.91
3.89
3.88
3.68
1.61
1.60
MeO2C
N
O
1H NMR (CDCl3, 400 MHz)
9.5 9.0 8.5 8.0 7.5 7.0 6.5 6.0 5.5 5.0 4.5 4.0 3.5 3.0 2.5 2.0 1.5 1.0 0.5 ppm
1.00
1.03
0.99
0.98
0.96
1.11
2.78
2.92
3.20
3.26
5.14
6.35

2d

E:Z 1.1:1

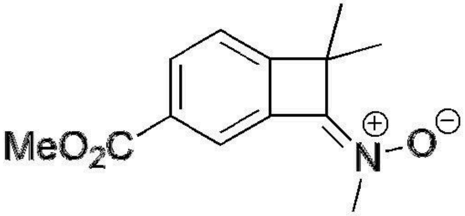

^{13}C NMR ($CDCl_3$, 100 MHz)

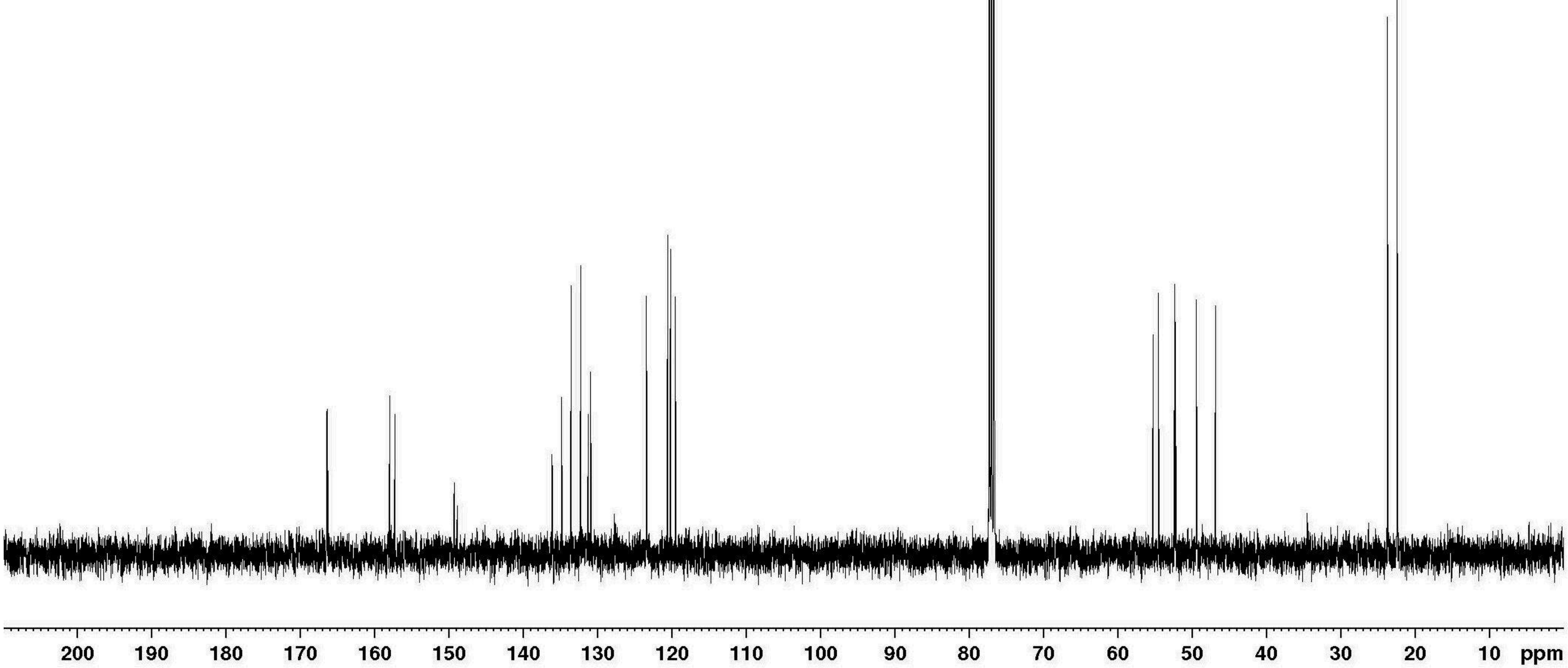

2e

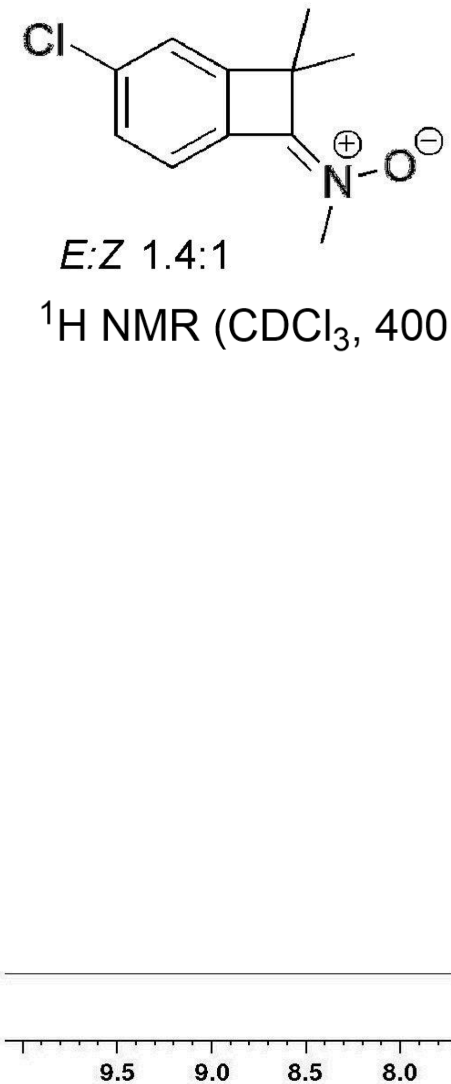

E:Z 1.4:1

^{1}H NMR ($CDCl_3$, 400 MHz)

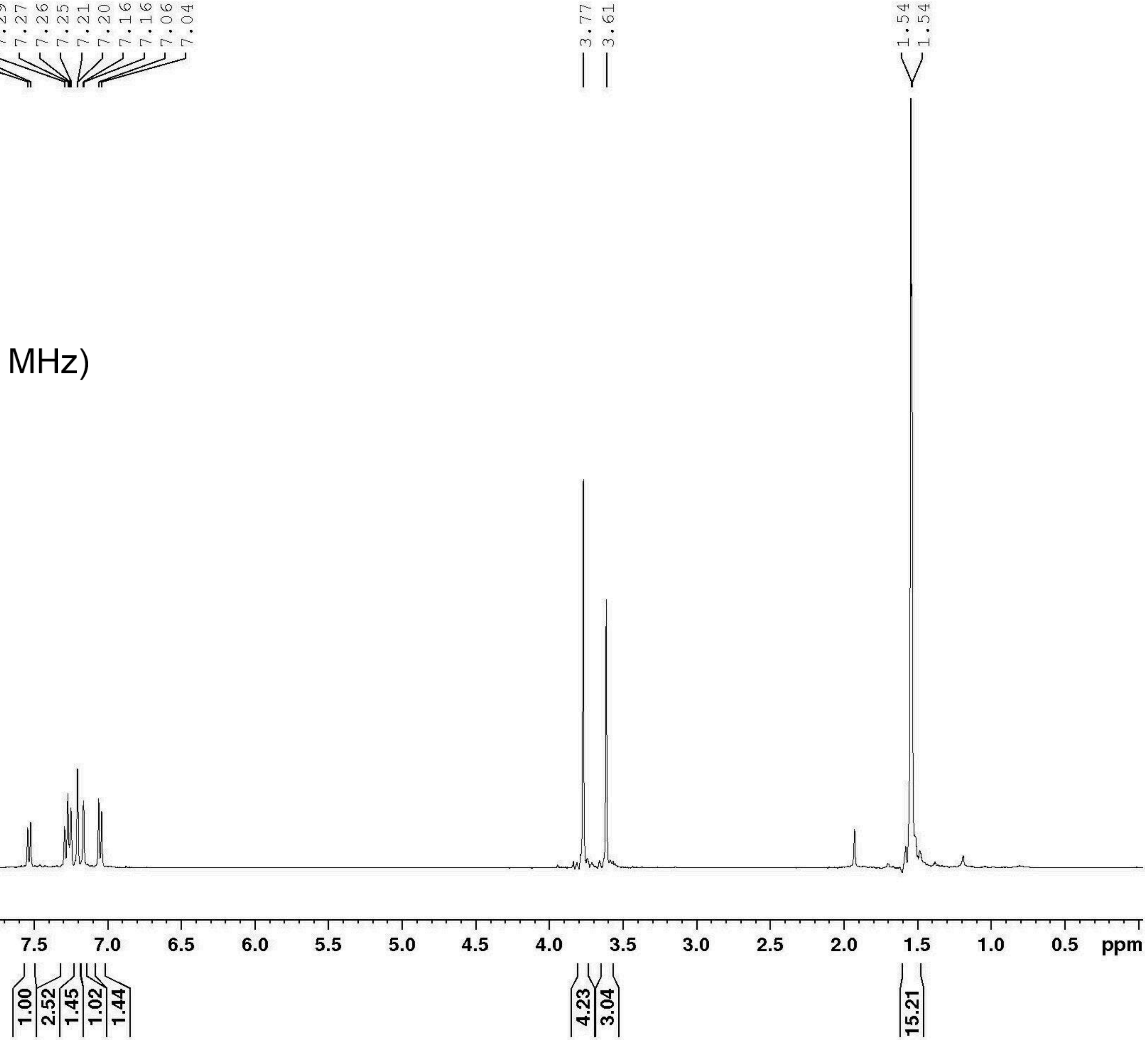

2e

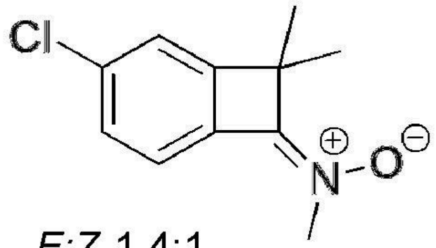

E:Z 1.4:1

^{13}C NMR ($CDCl_3$, 100 MHz)

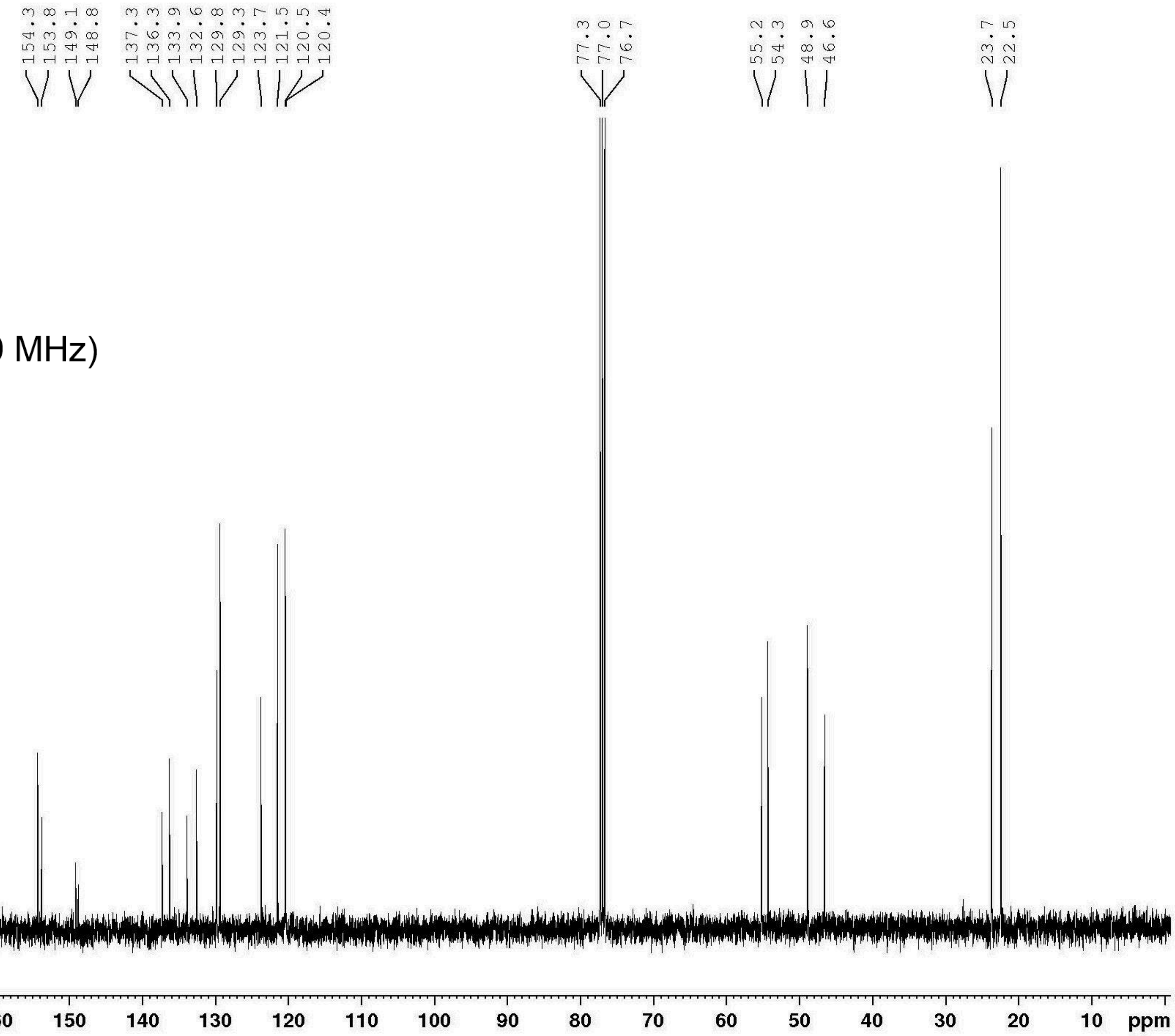

2f

E:*Z* 1.2:1

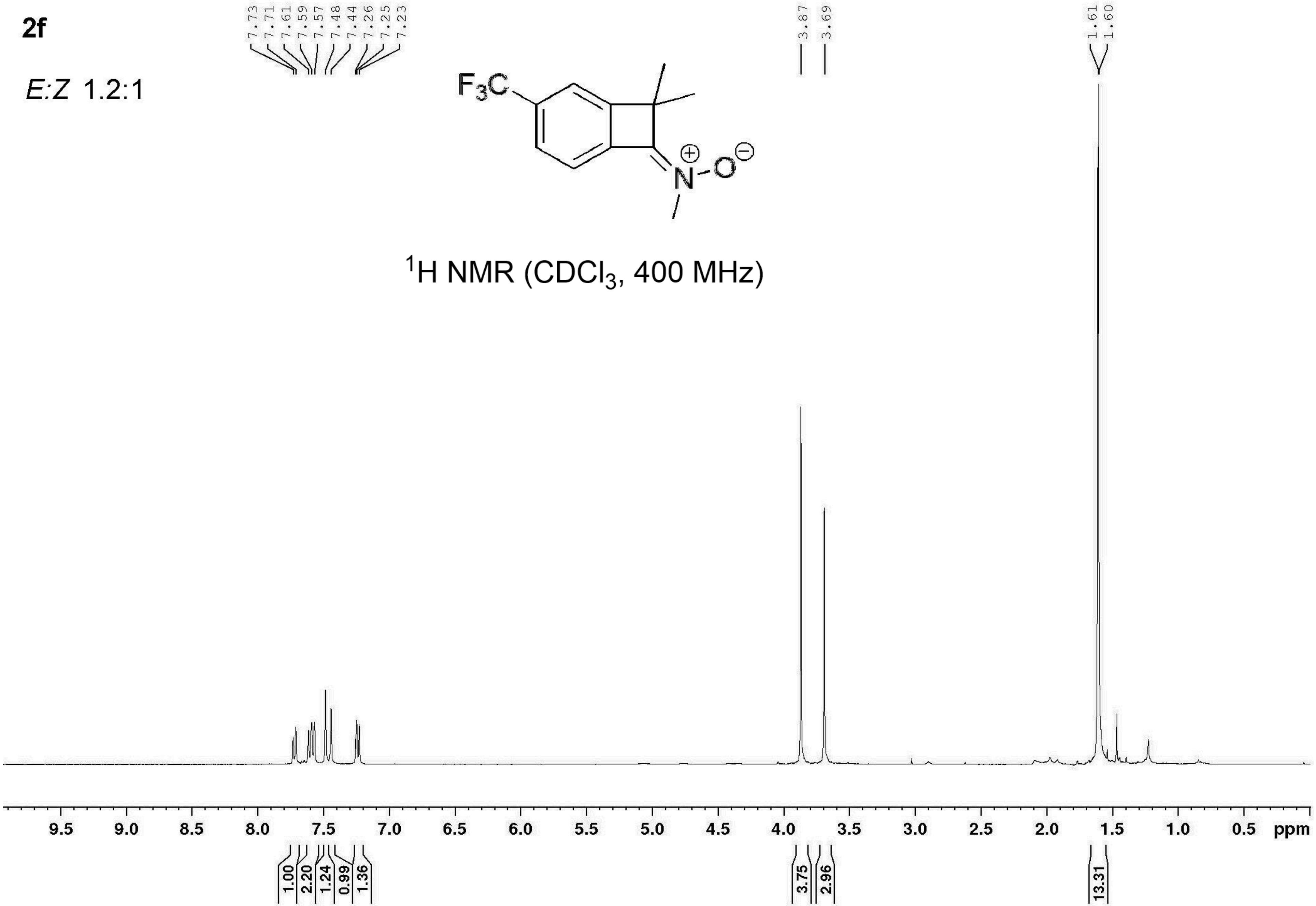
7.73
7.71
7.61
7.59
7.57
7.48
7.44
7.26
7.25
7.23
3.87
3.69
1.61
1.60
F3C
1H NMR (CDCl3, 400 MHz)
9.5
9.0
8.5
8.0
7.5
7.0
6.5
6.0
5.5
5.0
4.5
4.0
3.5
3.0
2.5
2.0
1.5
1.0
0.5
ppm
1.00
2.20
1.24
0.99
1.36
3.75
2.96
13.31

2f *E*:*Z* 1.2:1

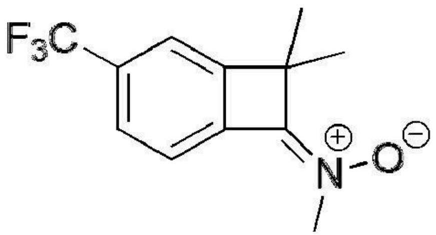

^{13}C NMR ($CDCl_3$, 100 MHz)

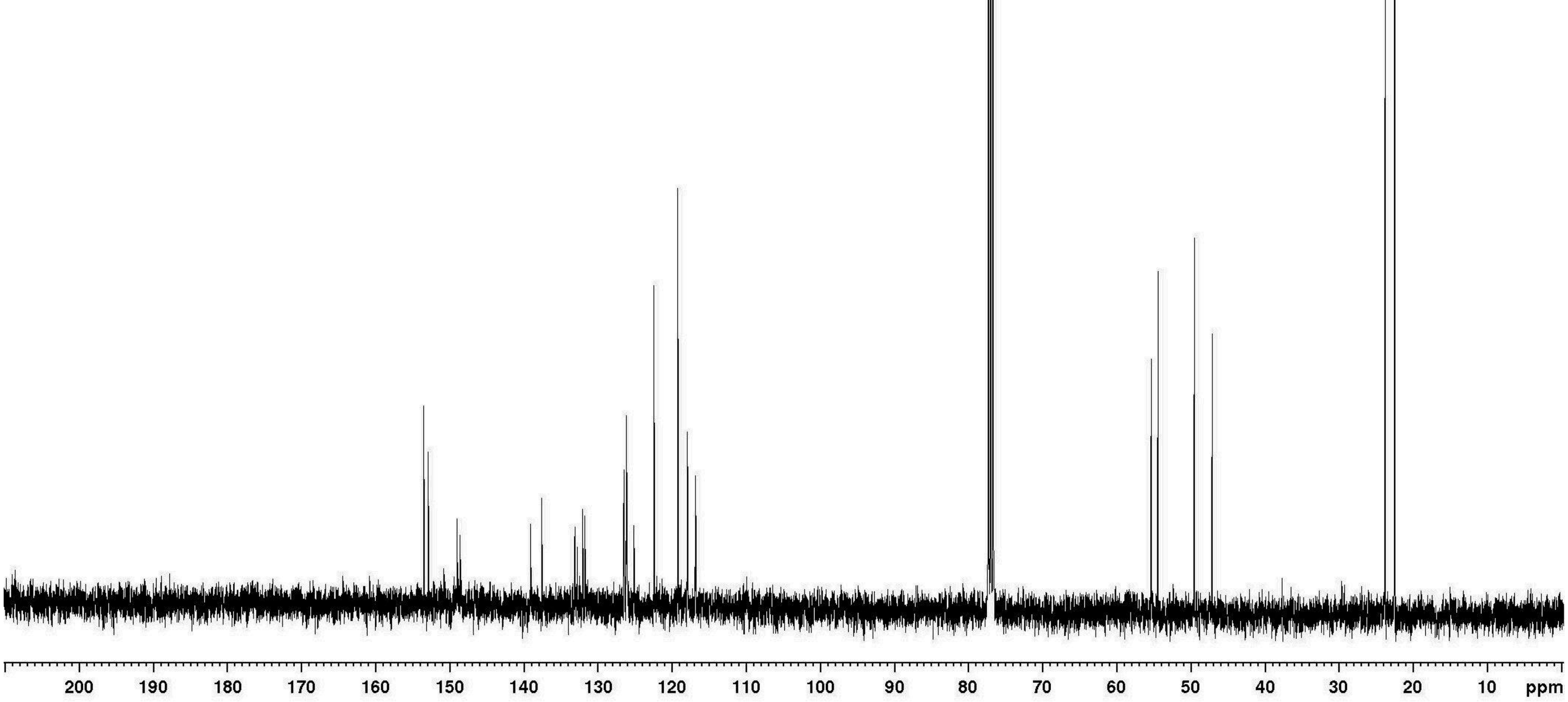

2f *E:Z* 1.2:1

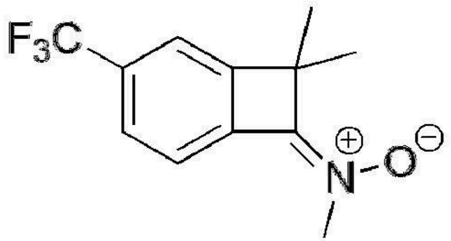

^{19}F NMR ($CDCl_3$, 376 MHz)

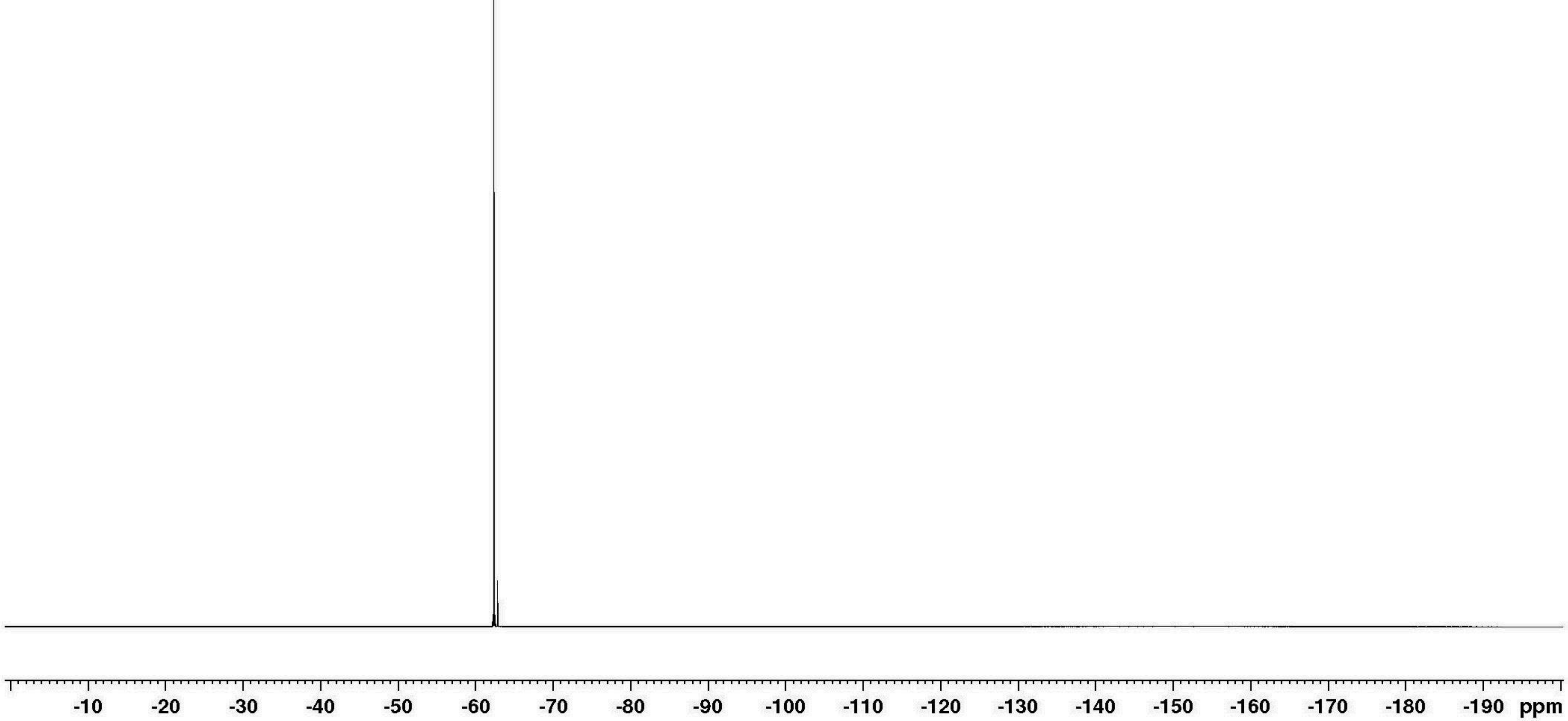

2g

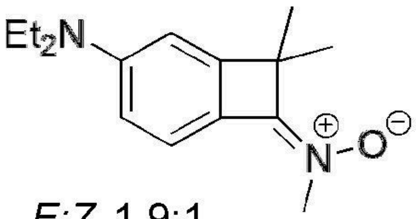

E:Z 1.9:1

^{1}H NMR ($CDCl_3$, 400 MHz)

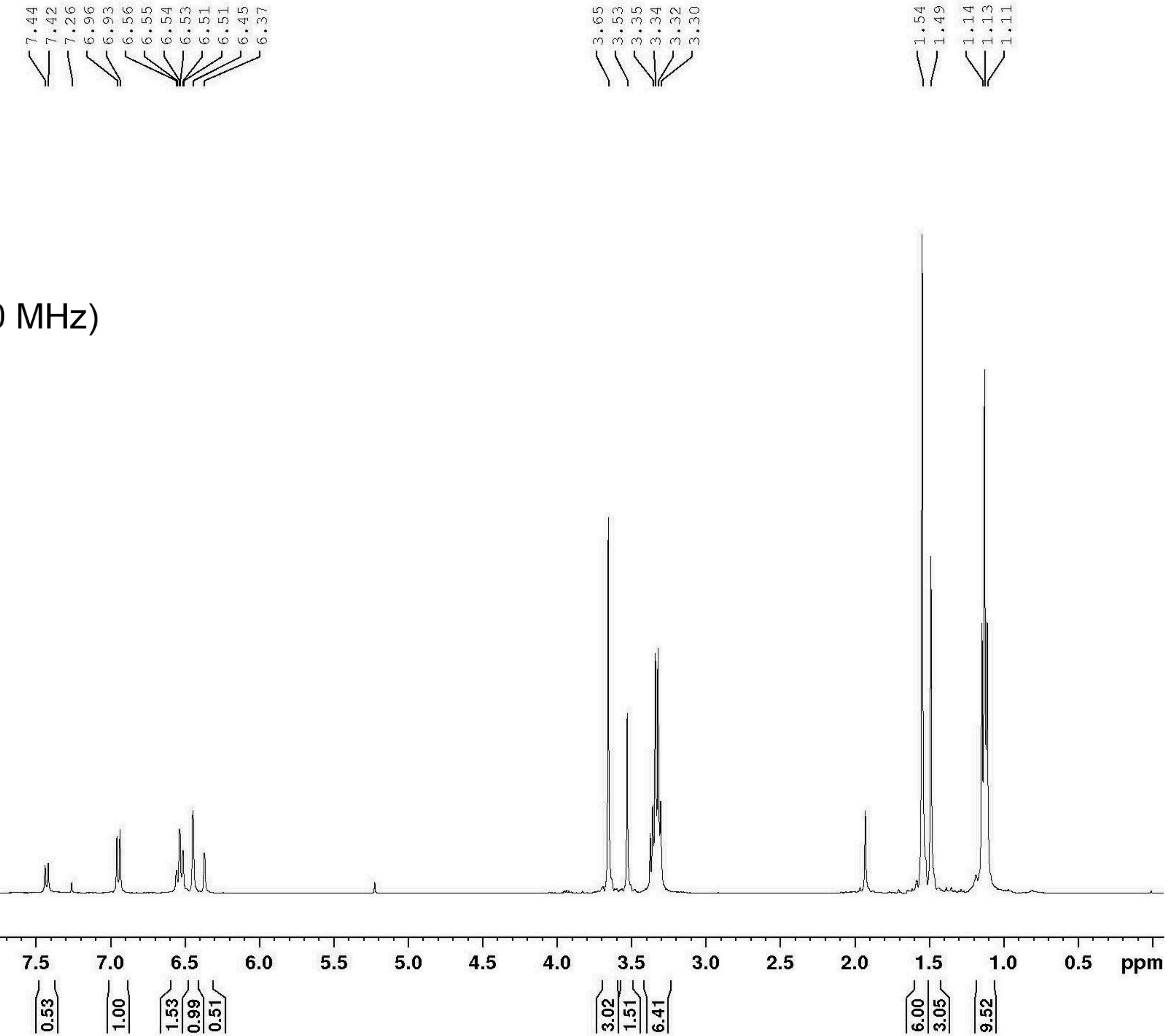

2g

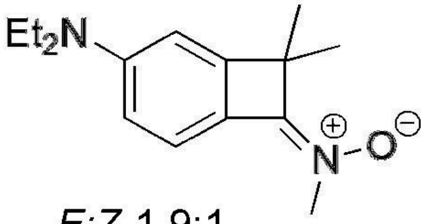

E:Z 1.9:1

^{13}C NMR ($CDCl_3$, 100 MHz)

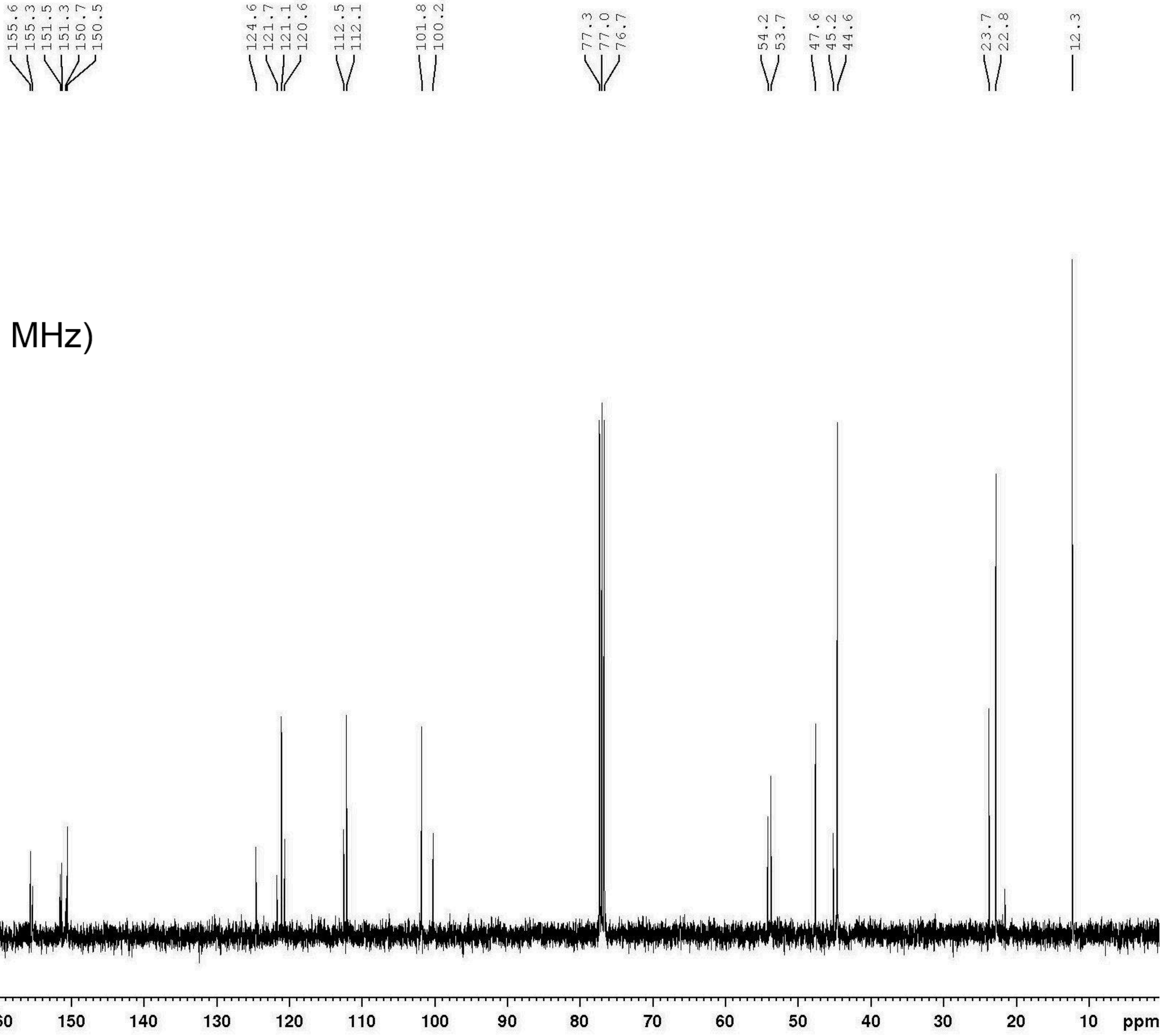

2h *E:Z* 2.7:1

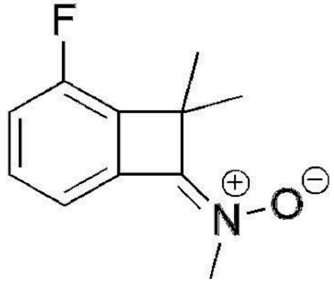

^{1}H NMR ($CDCl_3$, 400 MHz)

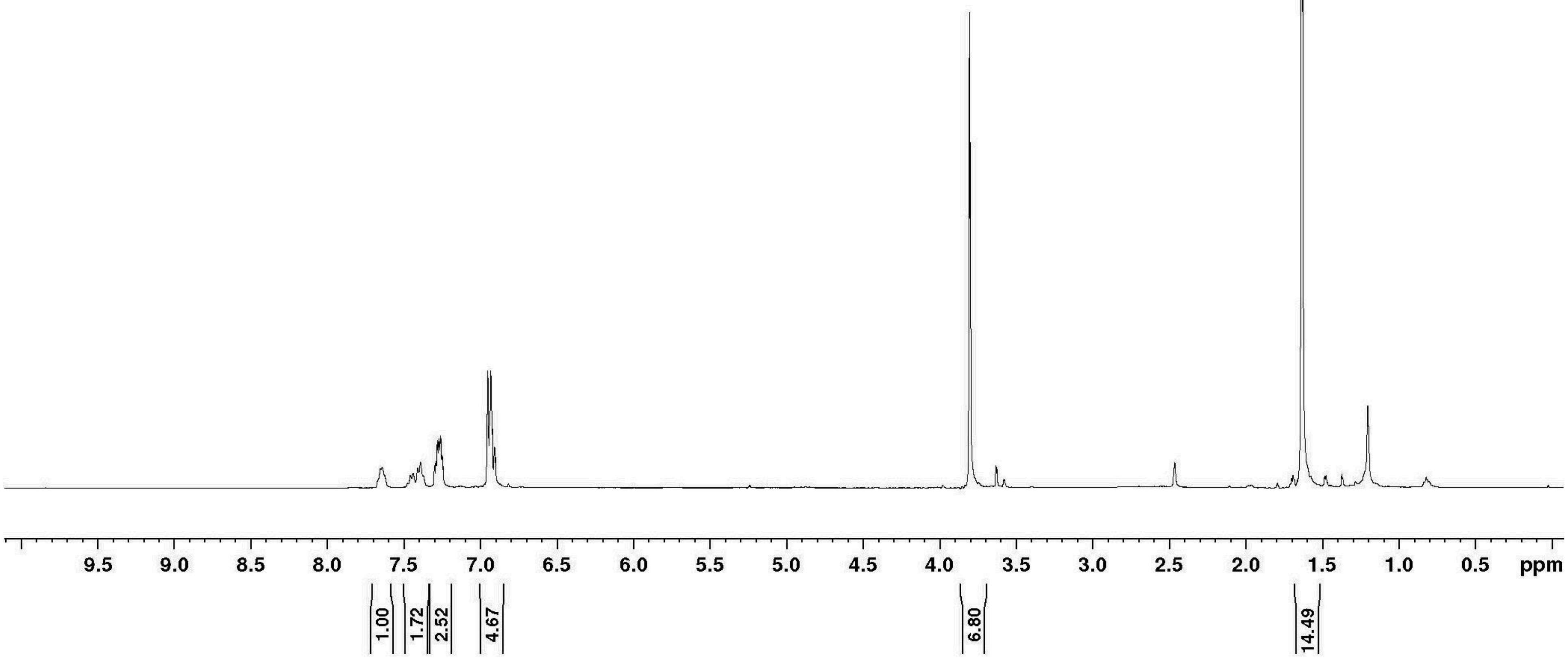

2h *E*:*Z* 2.7:1

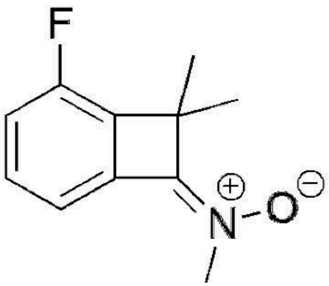

^{13}C NMR ($CDCl_3$, 100 MHz)

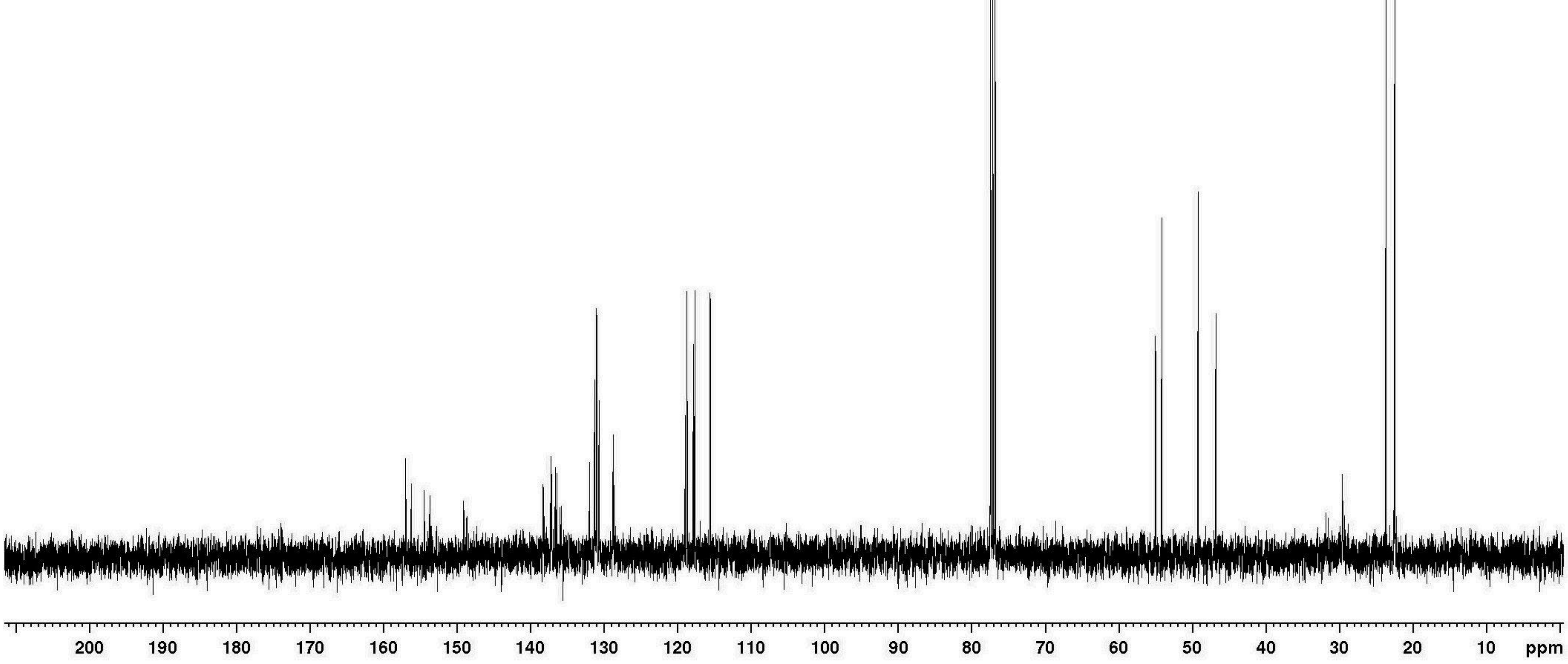

2h *E:Z* 2.7:1

^{19}F NMR ($CDCl_3$, 376 MHz)

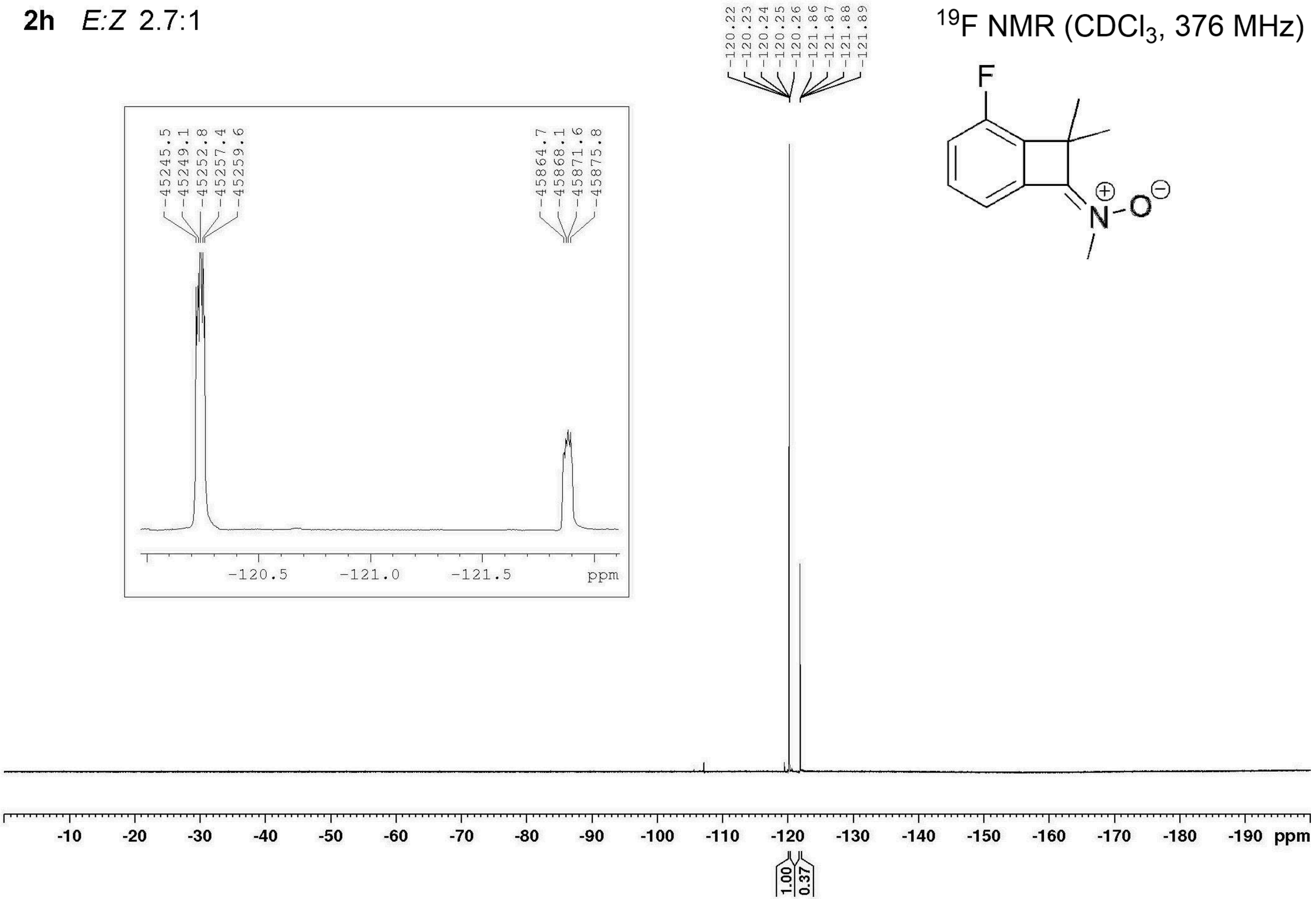

2i *E:Z* 1:1.4

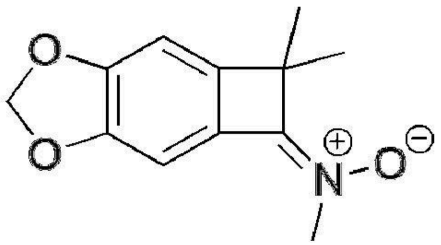

^{1}H NMR ($CDCl_3$, 400 MHz)

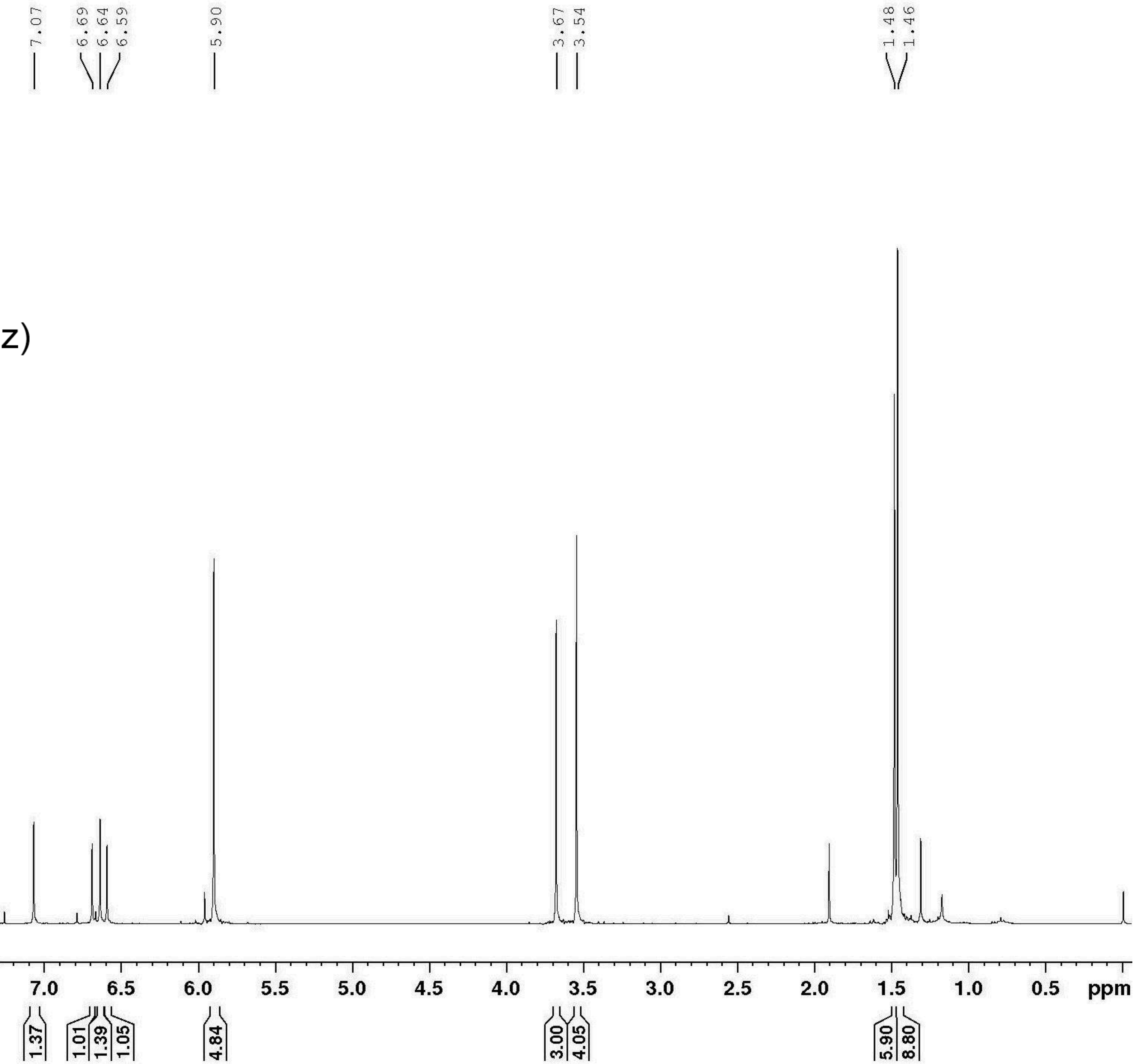

2i *E:Z* 1:1.4

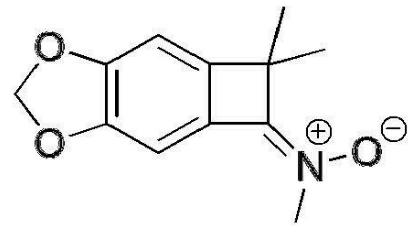

^{13}C NMR ($CDCl_3$, 100 MHz)

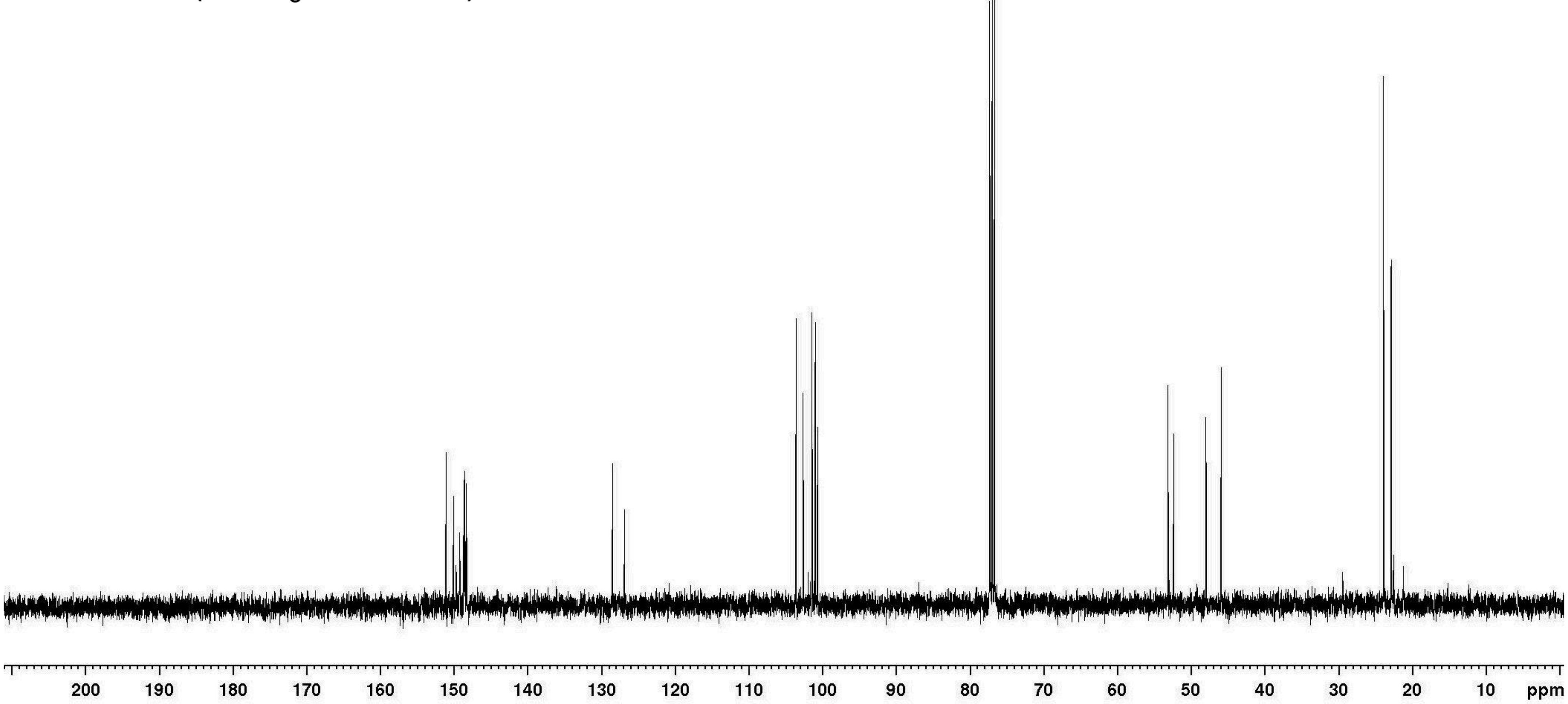

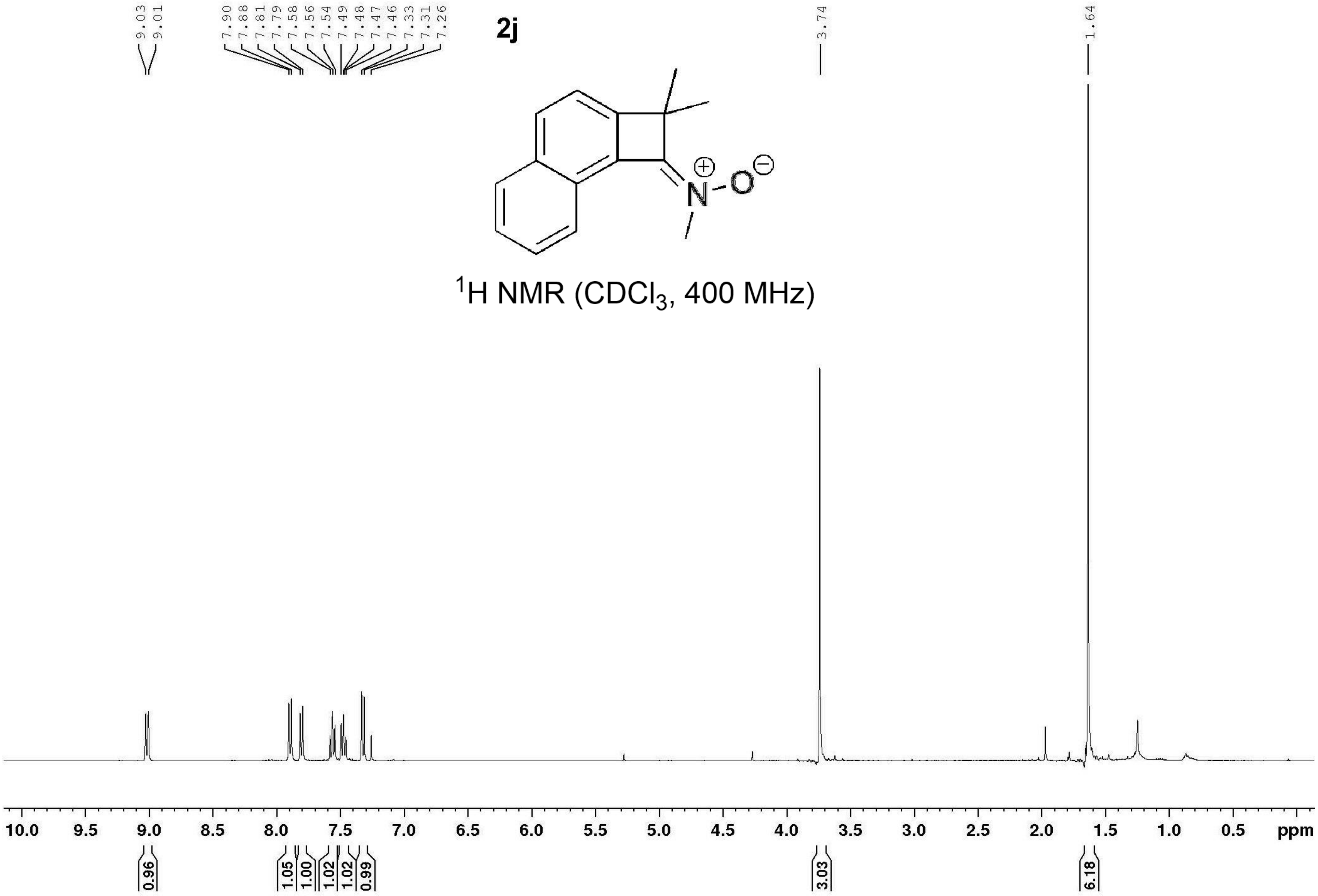
9.03
9.01
7.90
7.88
7.81
7.79
7.58
7.56
7.54
7.49
7.48
7.47
7.46
7.33
7.31
7.26
2j
3.74
1.64
^{1}H NMR (CDCl$_3$, 400 MHz)
10.0 9.5 9.0 8.5 8.0 7.5 7.0 6.5 6.0 5.5 5.0 4.5 4.0 3.5 3.0 2.5 2.0 1.5 1.0 0.5 ppm
0.96
1.05
1.00
1.02
1.02
0.99
3.03
6.18

2j

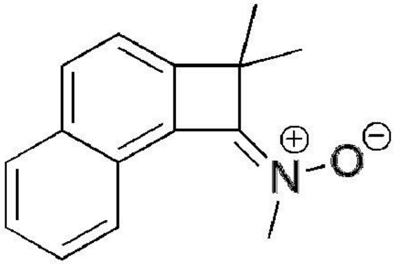

^{13}C NMR ($CDCl_3$, 100 MHz)

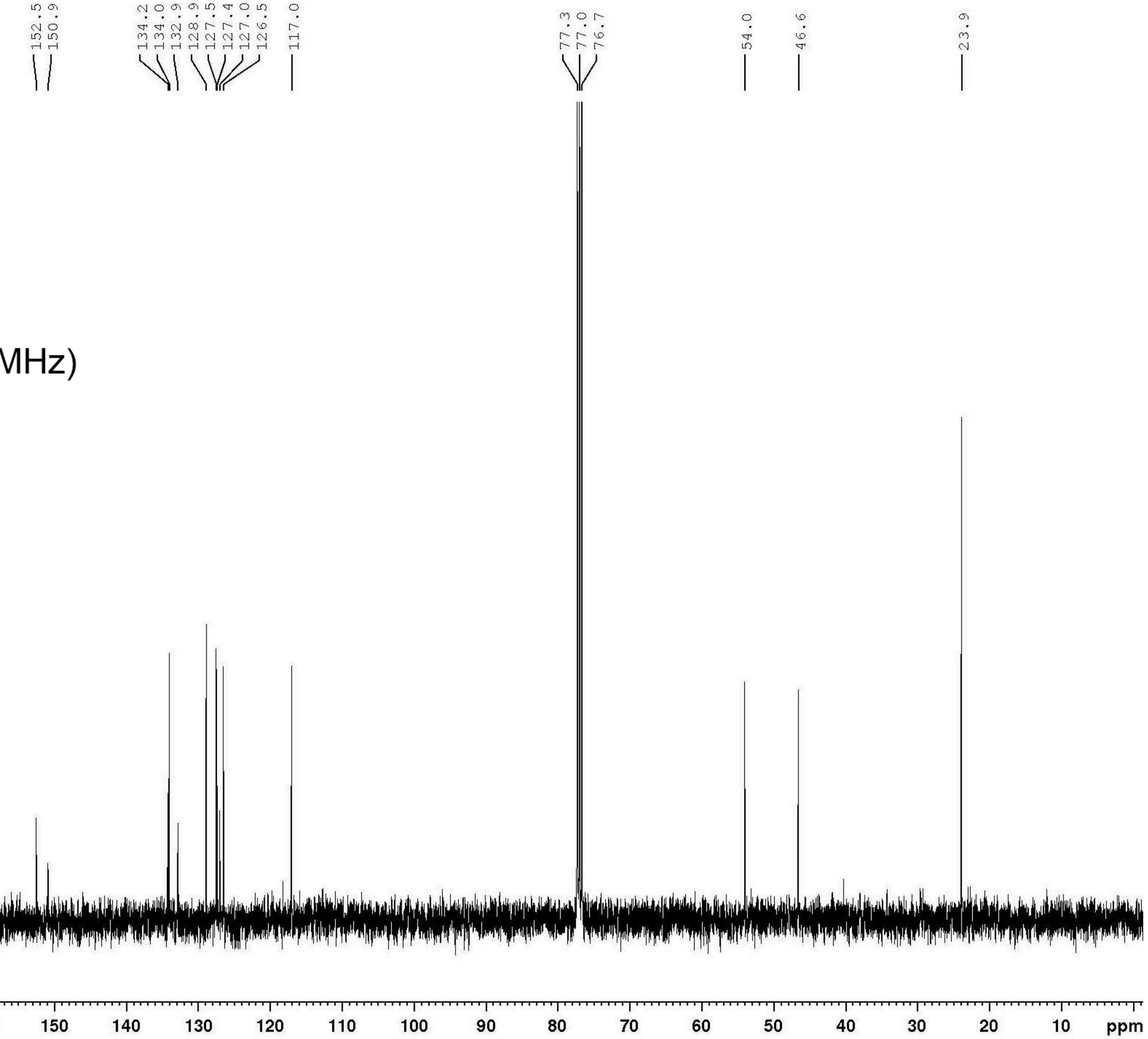

2k

E:*Z* 10:1

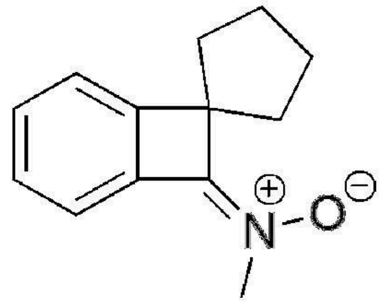

^1H NMR ($CDCl_3$, 400 MHz)

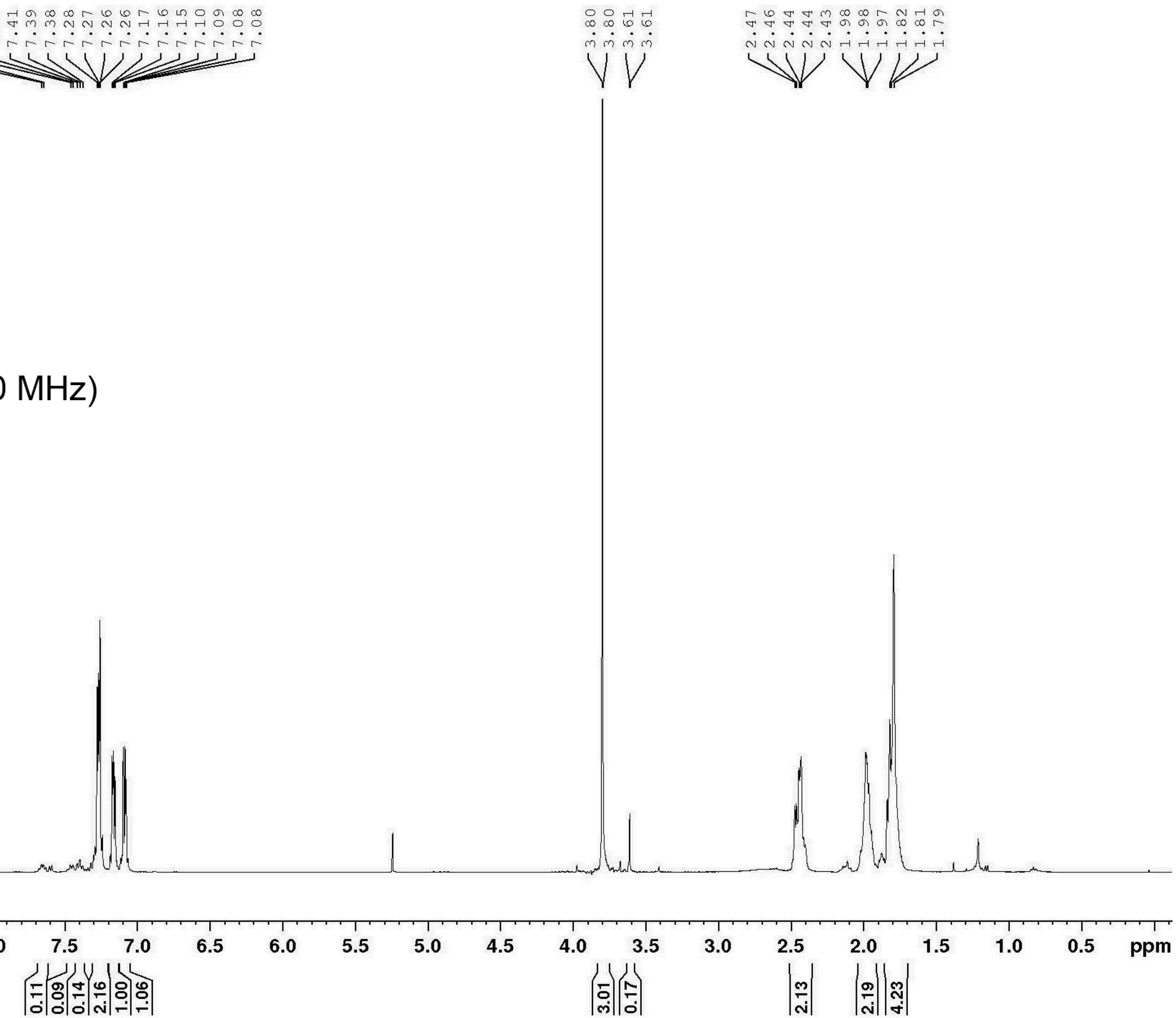

2k *E:Z* 10:1

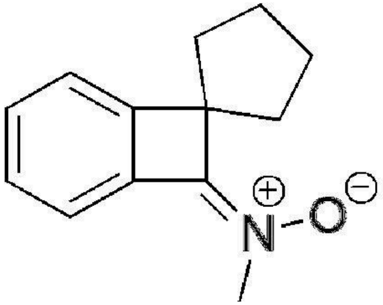

^{13}C NMR ($CDCl_3$, 100 MHz)

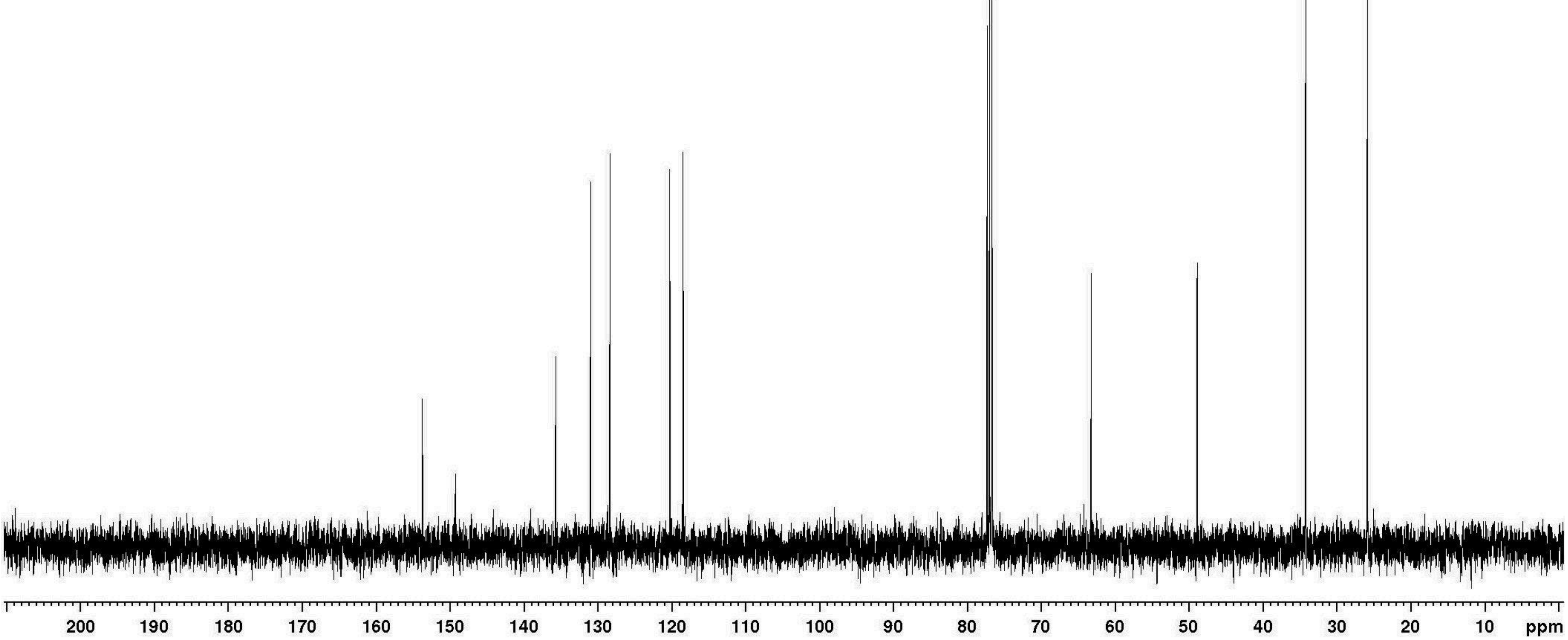

2l

E:*Z* 1.7:1

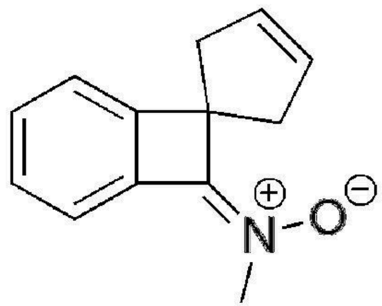

^{1}H NMR ($CDCl_3$, 400 MHz)

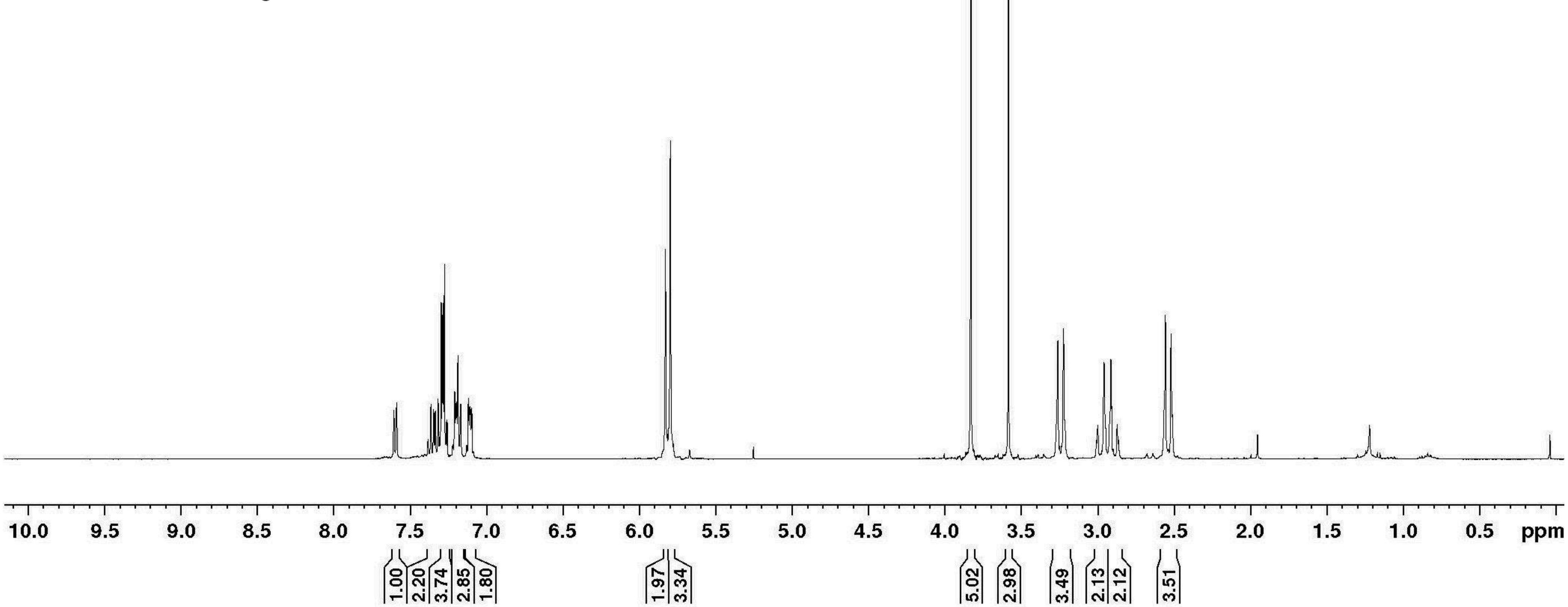

2l

E:*Z* 1.7:1

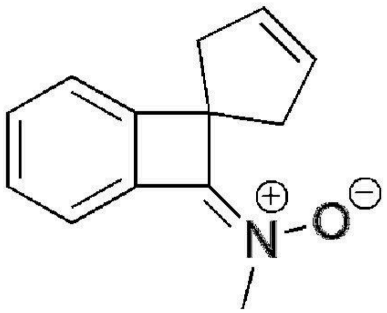

^{13}C NMR ($CDCl_3$, 100 MHz)

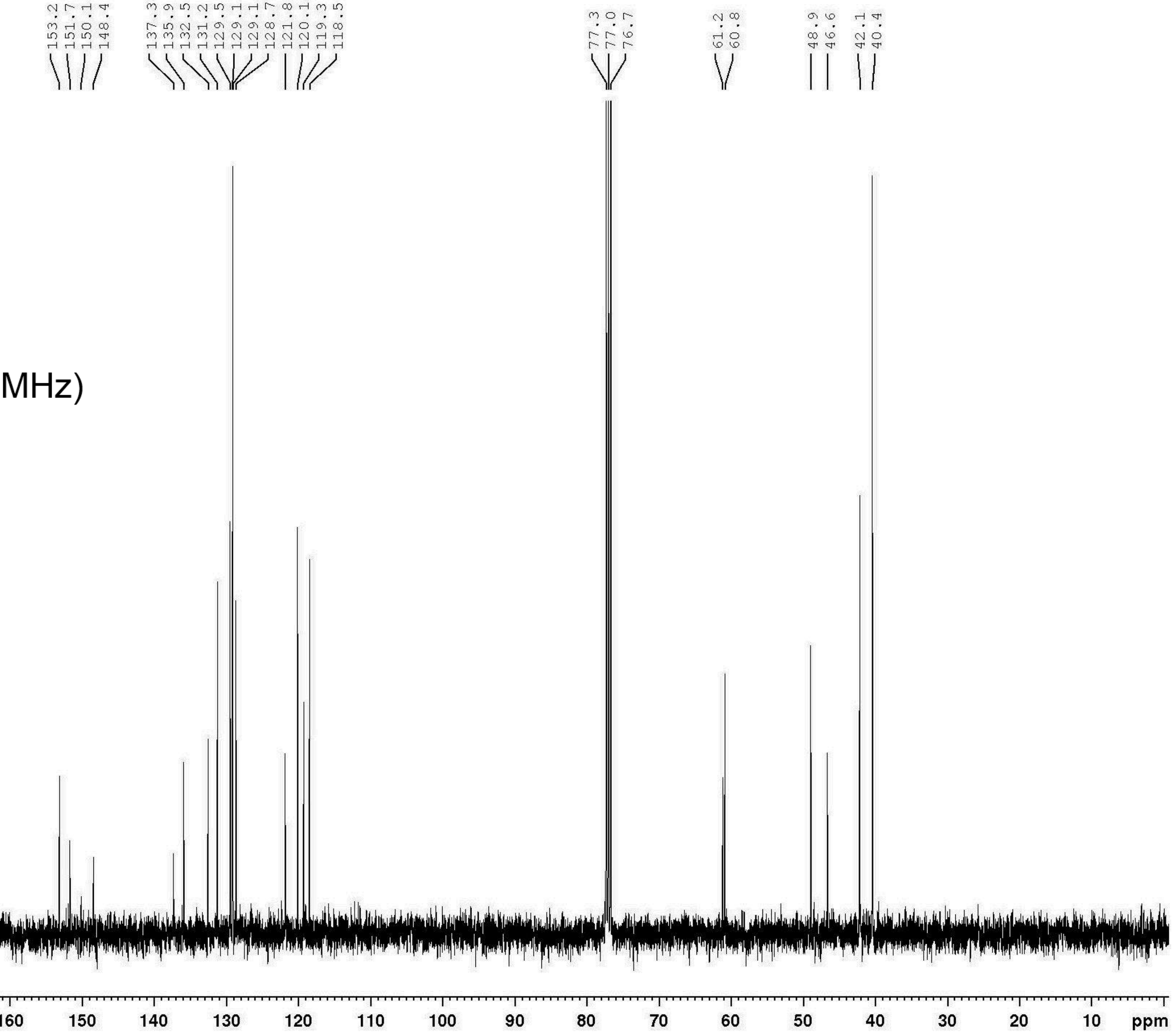

2m

E:*Z* 2.2:1

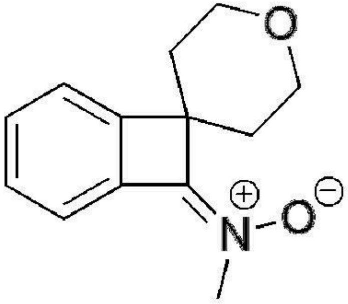

^{1}H NMR ($CDCl_3$, 400 MHz)

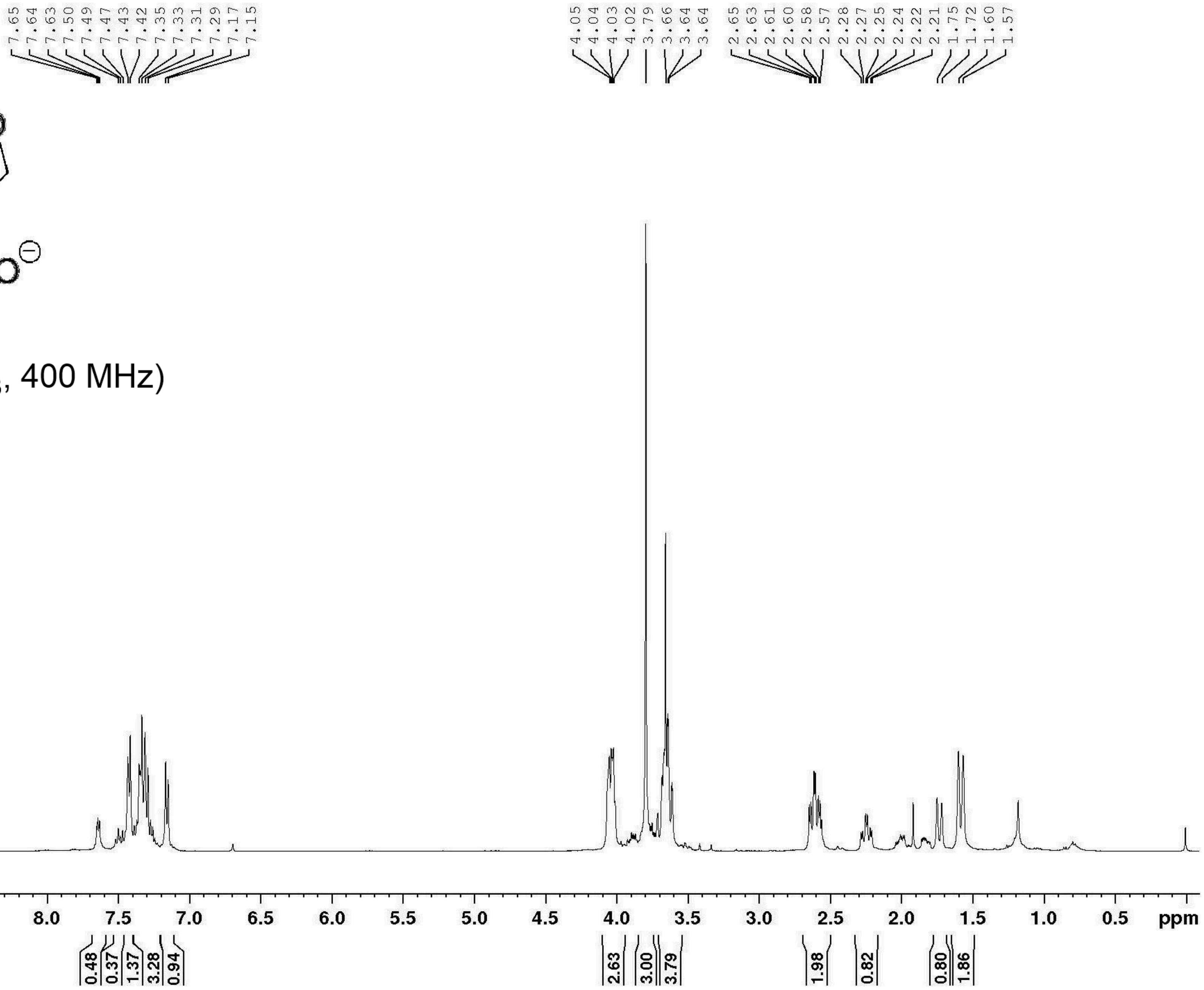

2m *E:Z* 2.2:1

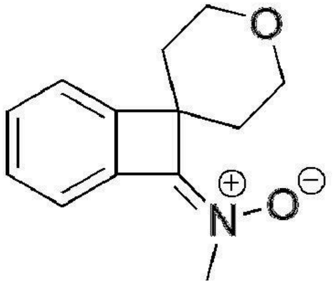

^{13}C NMR ($CDCl_3$, 100 MHz)

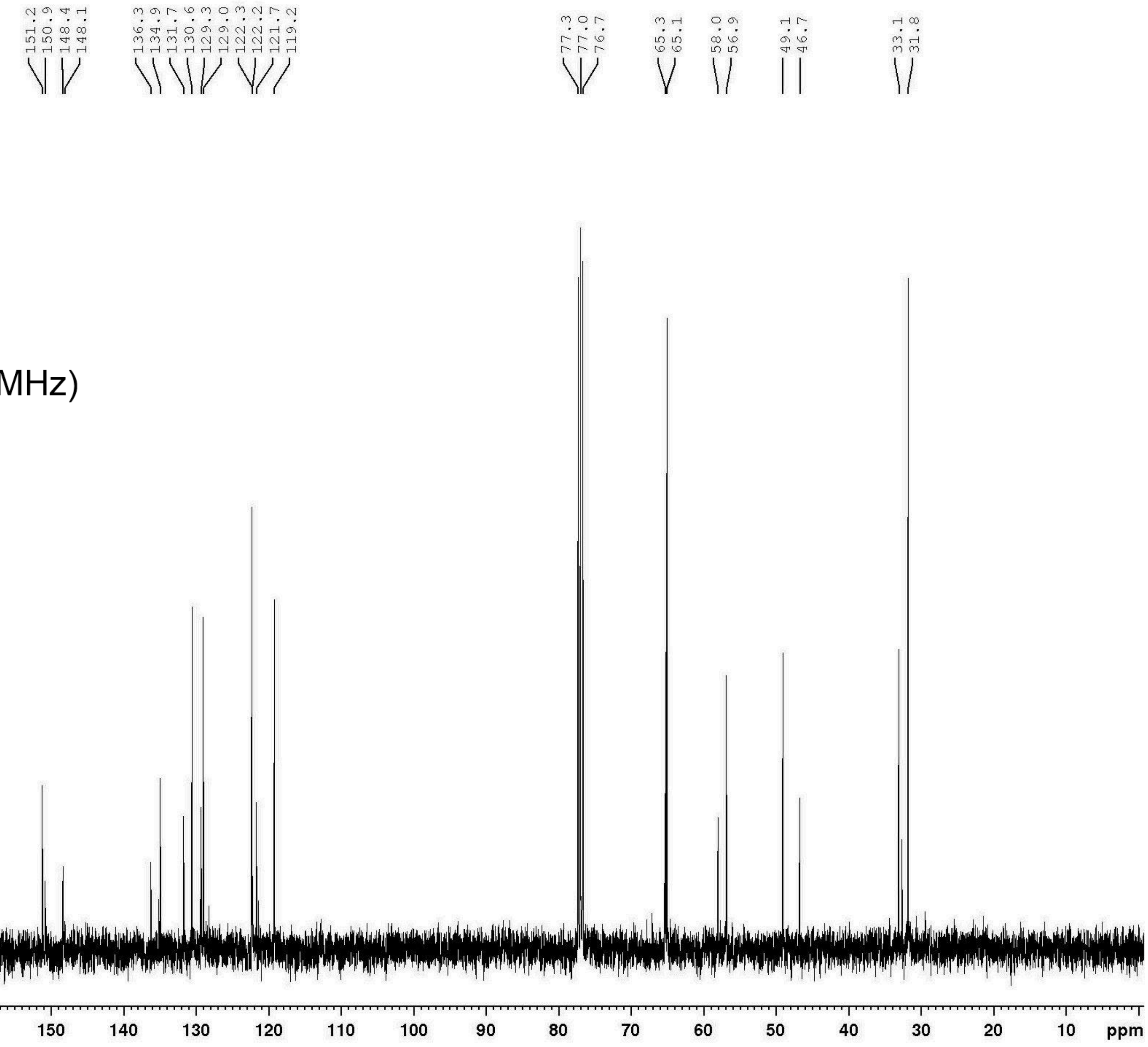

2n *E:Z* 6.6:1

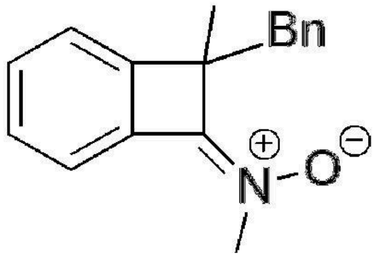

^1H NMR ($CDCl_3$, 400 MHz)

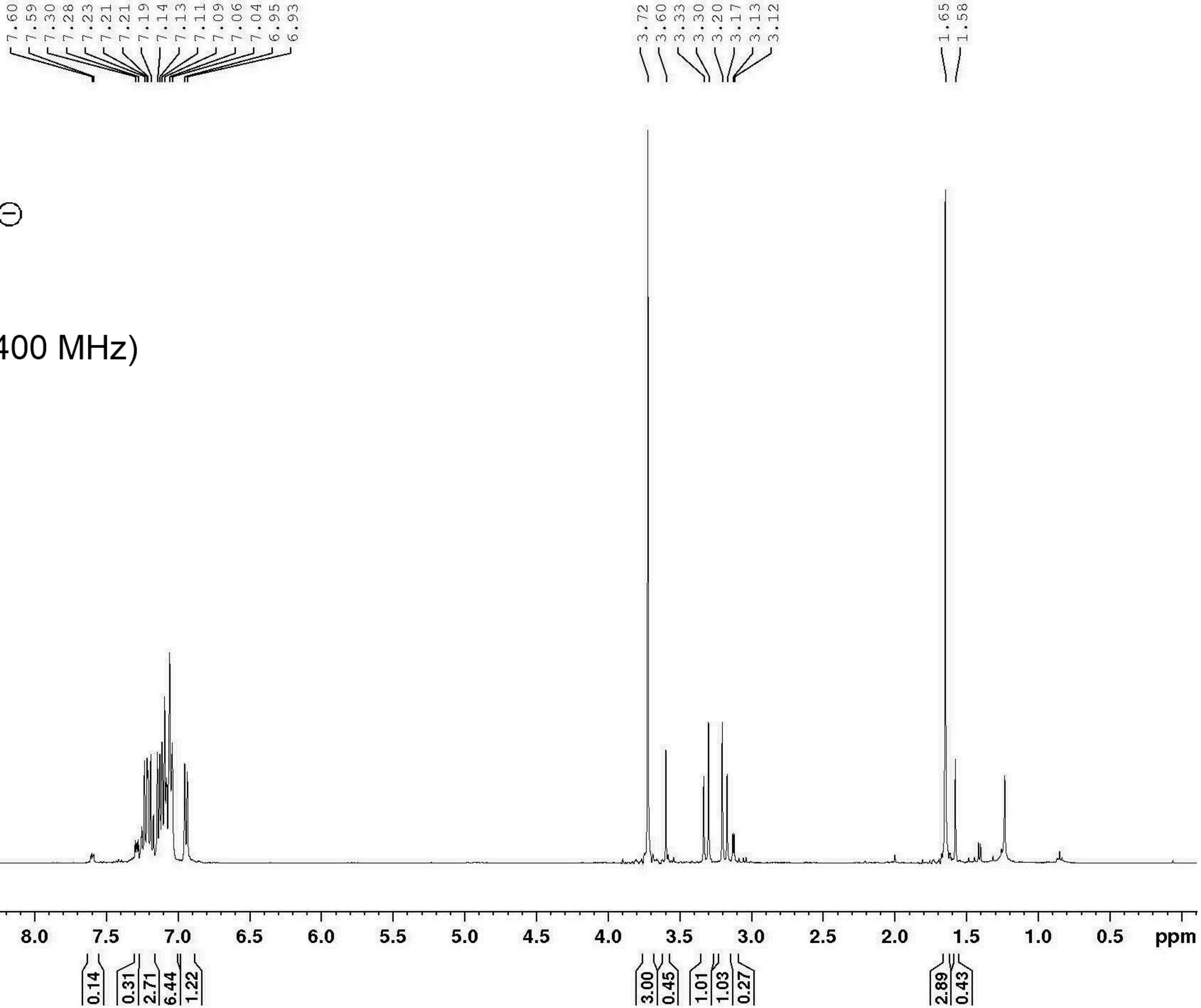

2n *E:Z* 6.6:1

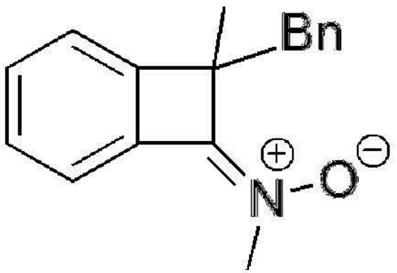

^{13}C NMR (CDCl$_3$, 100 MHz)

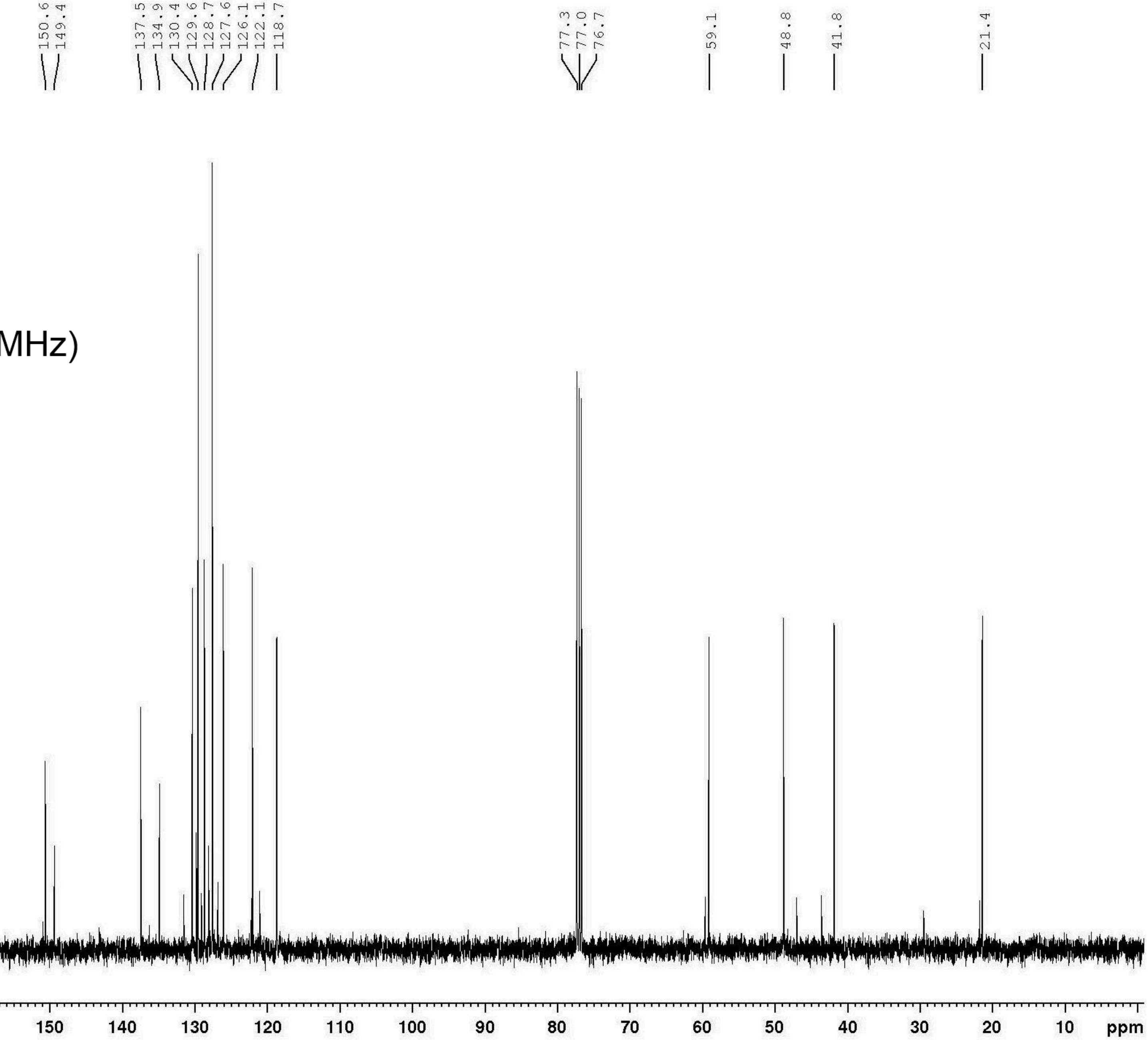

2o

E:*Z* 1.2:1

^{1}H NMR ($CDCl_3$, 400 MHz)

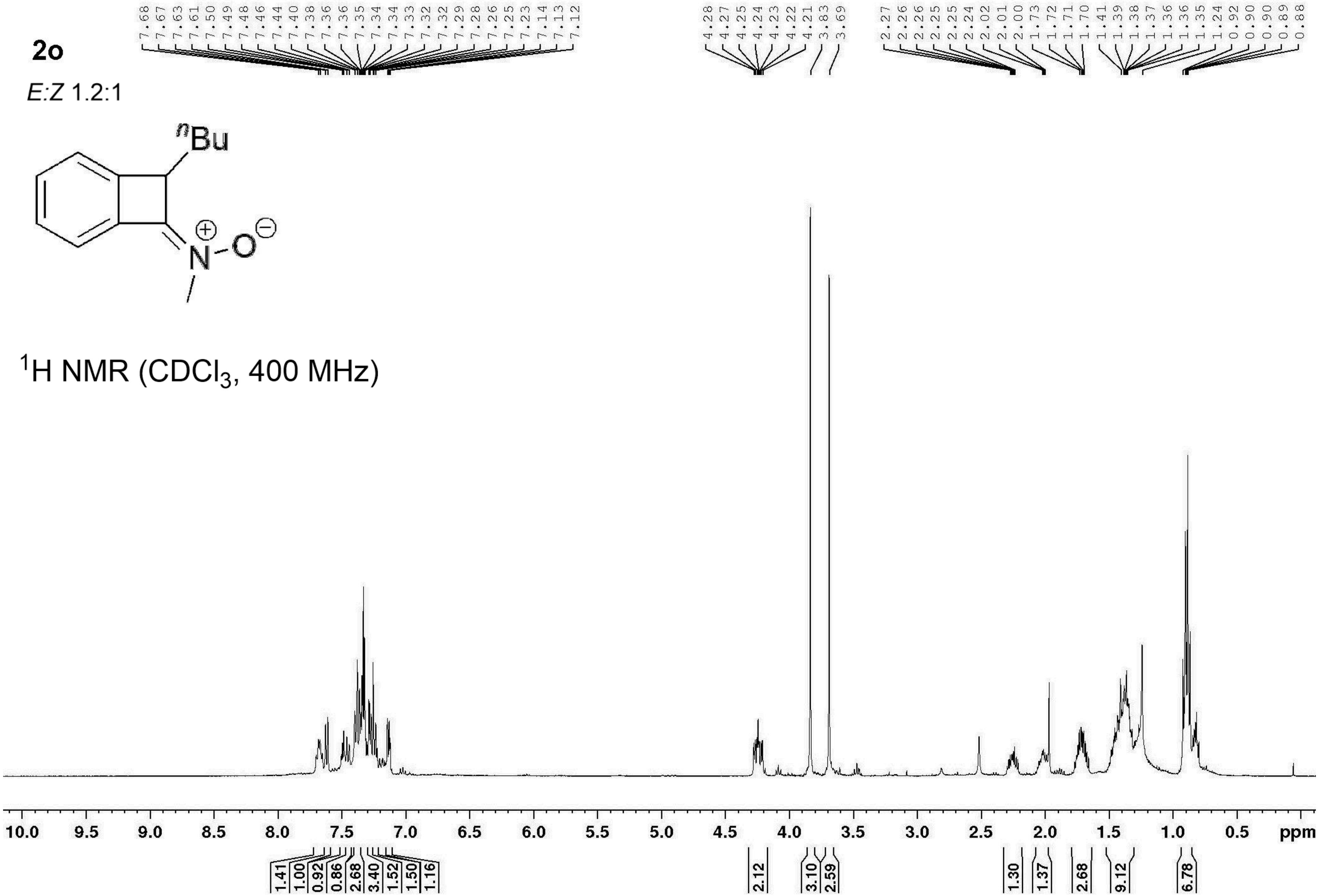

2o *E*:*Z* 1.2:1

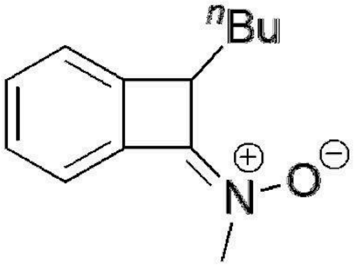

^{13}C NMR ($CDCl_3$, 100 MHz)

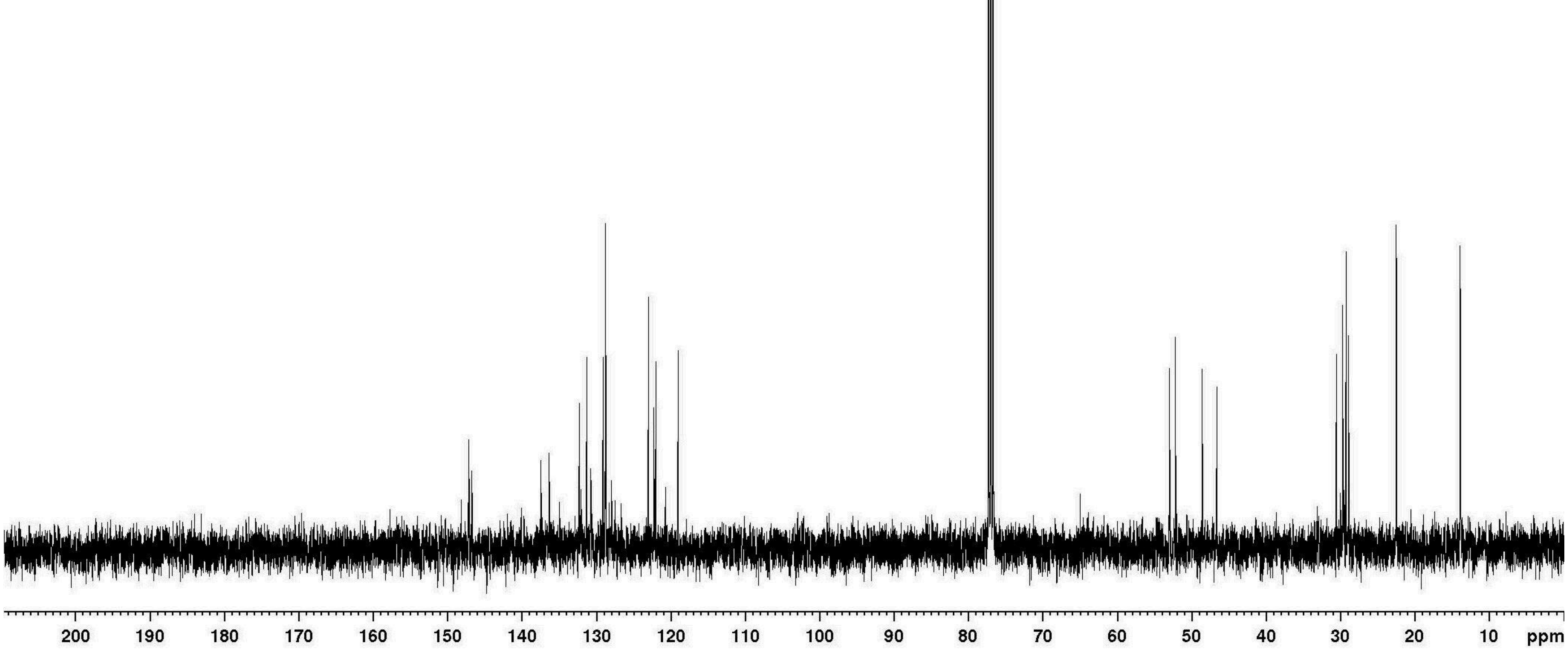

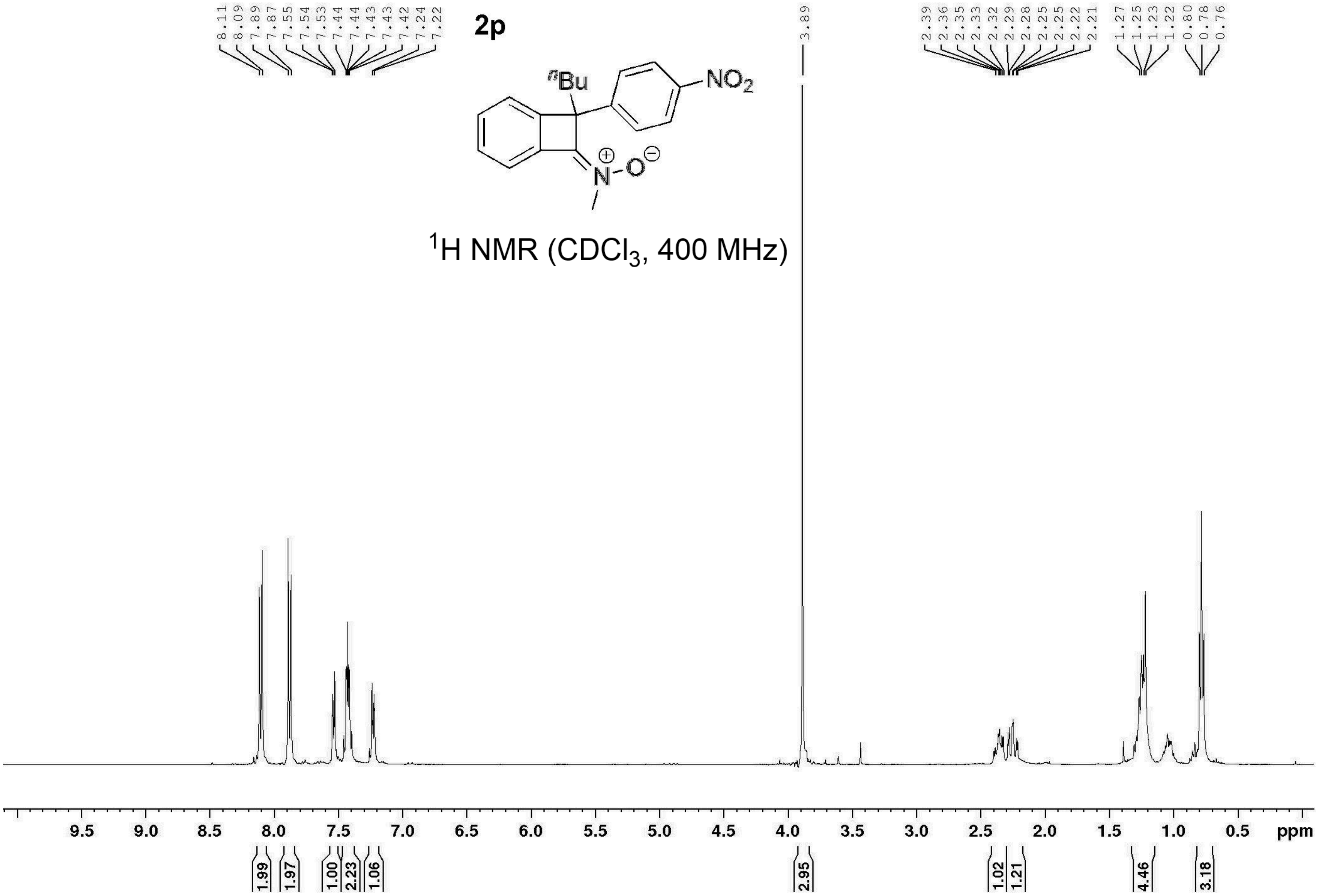
2p
nBu
NO2
1H NMR (CDCl3, 400 MHz)
8.11 8.09 7.89 7.87 7.55 7.54 7.53 7.44 7.44 7.43 7.43 7.42 7.24 7.22
3.89
2.39 2.36 2.35 2.33 2.32 2.29 2.28 2.25 2.25 2.22 2.21
1.27 1.25 1.23 1.22
0.80 0.78 0.76
9.5 9.0 8.5 8.0 7.5 7.0 6.5 6.0 5.5 5.0 4.5 4.0 3.5 3.0 2.5 2.0 1.5 1.0 0.5 ppm
1.99 1.97 1.00 2.23 1.06 2.95 1.02 1.21 4.46 3.18

2p

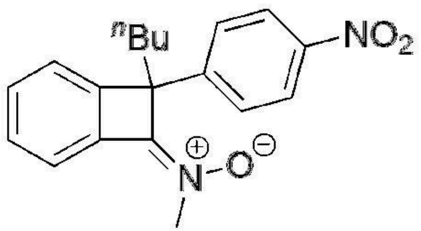

^{13}C NMR ($CDCl_3$, 100 MHz)

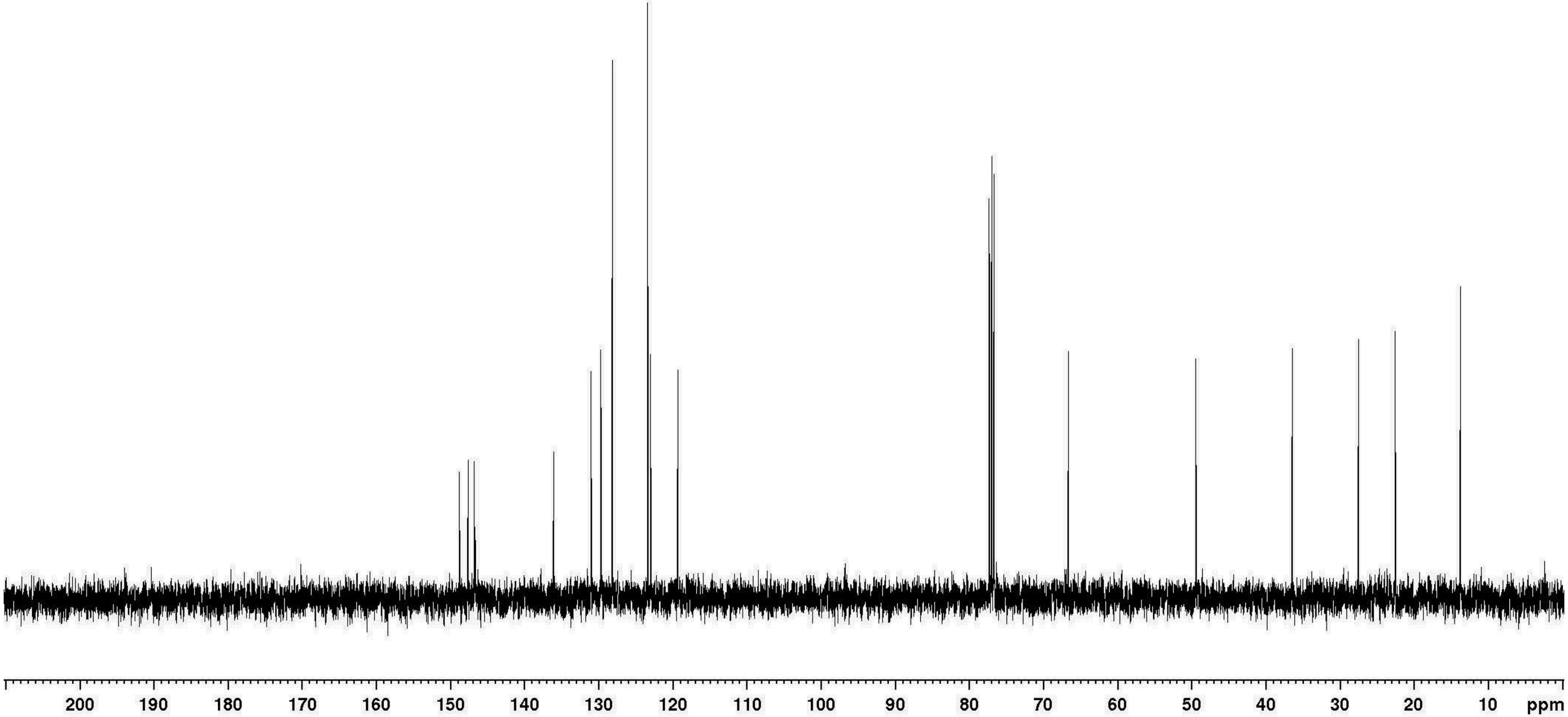

2q

E:*Z* 2.5:1

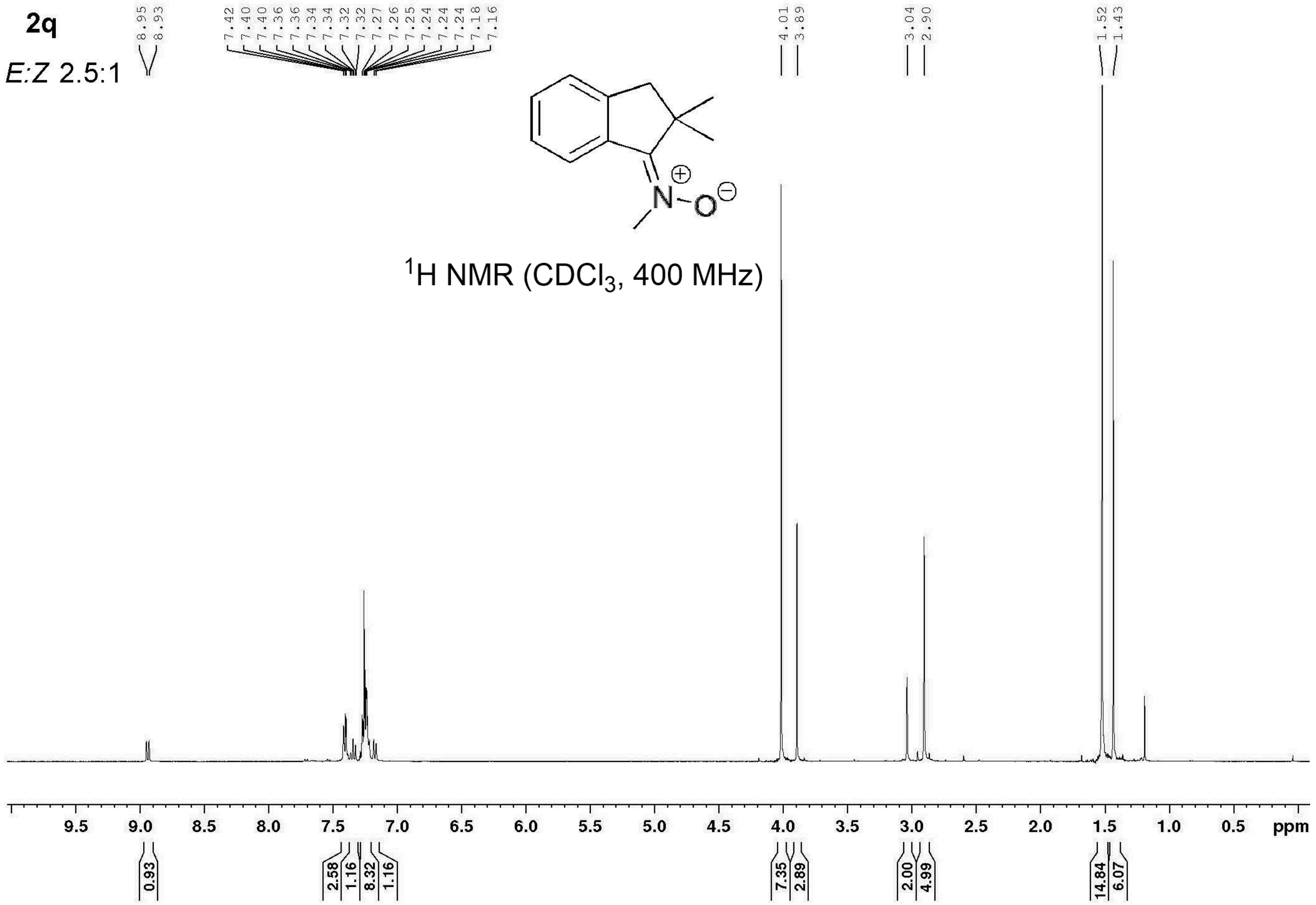
8.95
8.93
7.42
7.40
7.40
7.36
7.36
7.34
7.34
7.32
7.32
7.27
7.26
7.25
7.24
7.24
7.24
7.18
7.16
4.01
3.89
3.04
2.90
1.52
1.43
N
O
^{1}H NMR ($CDCl_3$, 400 MHz)
9.5
9.0
8.5
8.0
7.5
7.0
6.5
6.0
5.5
5.0
4.5
4.0
3.5
3.0
2.5
2.0
1.5
1.0
0.5
ppm
0.93
2.58
1.16
8.32
1.16
7.35
2.89
2.00
4.99
14.84
6.07

2q *E*:*Z* 2.5:1

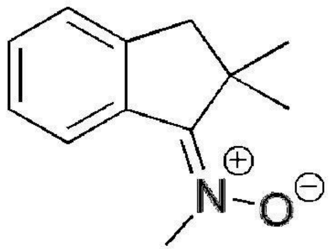

^{13}C NMR ($CDCl_3$, 100 MHz)

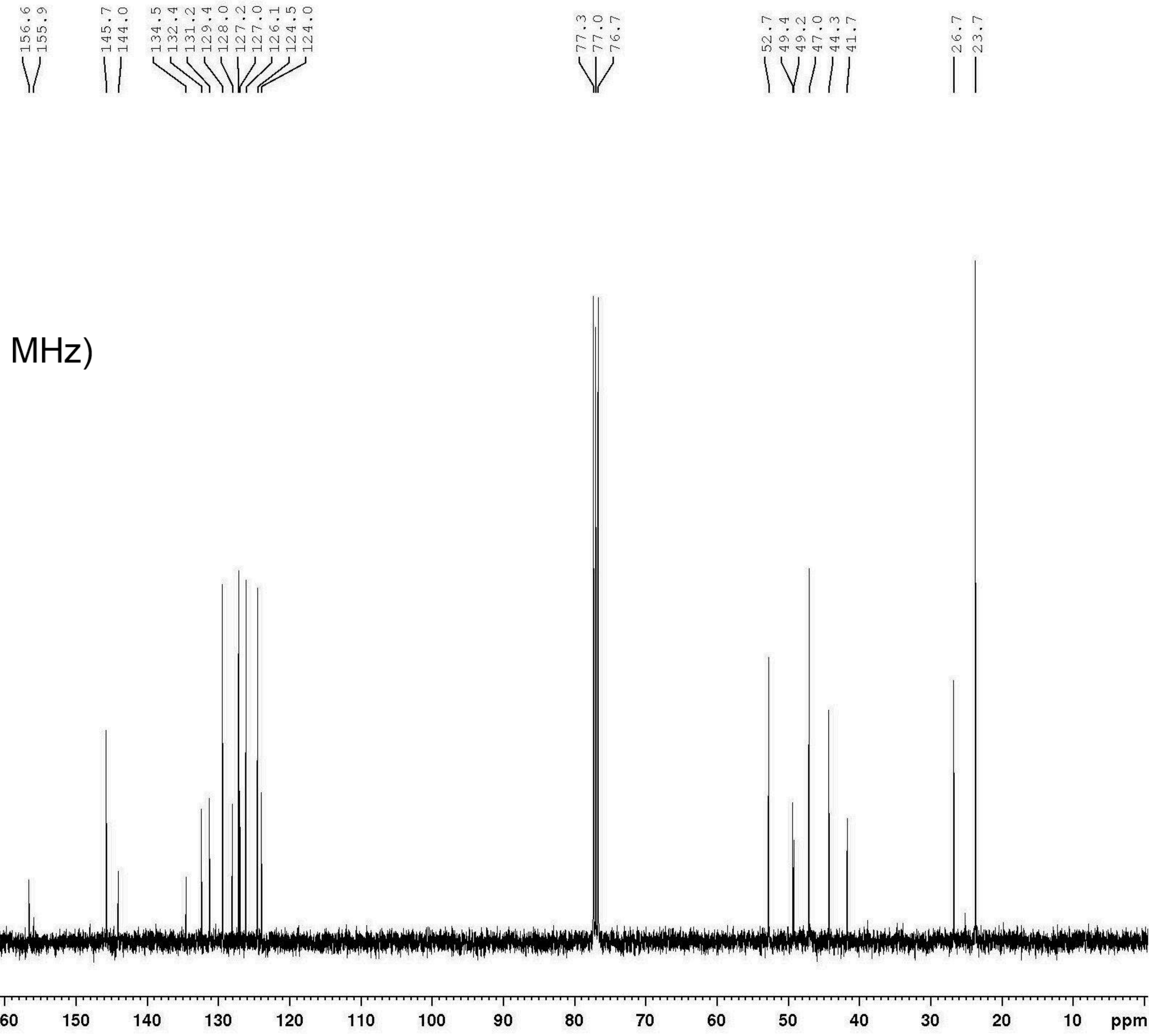

2r

E:*Z* 4.8:1

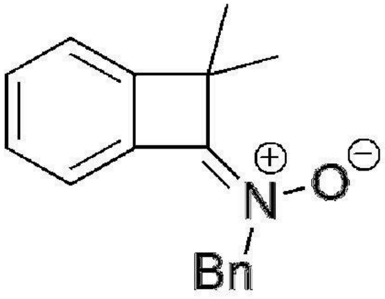

^{1}H NMR ($CDCl_3$, 400 MHz)

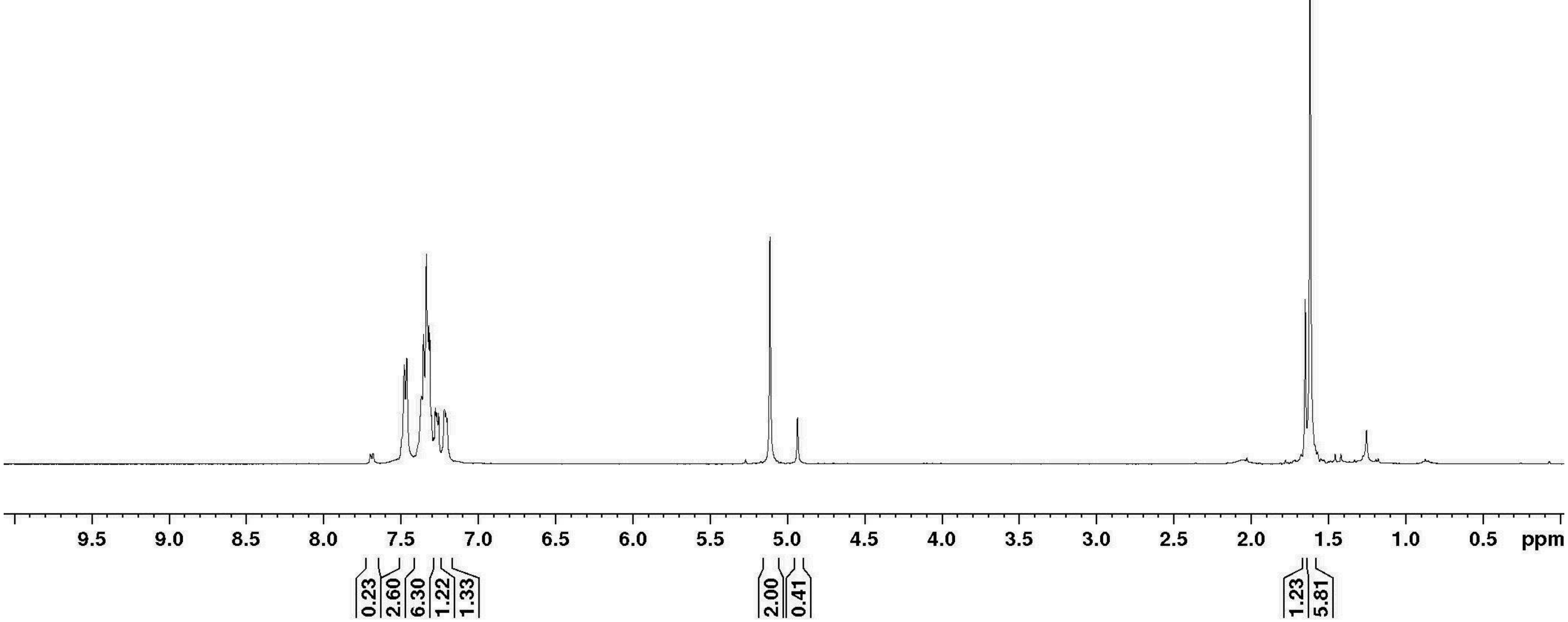

2r

E:Z 4.8:1

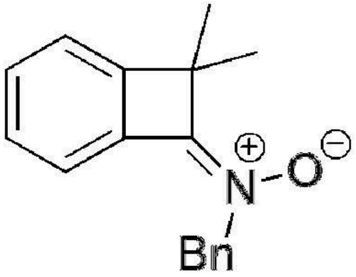

^{13}C NMR ($CDCl_3$, 100 MHz)

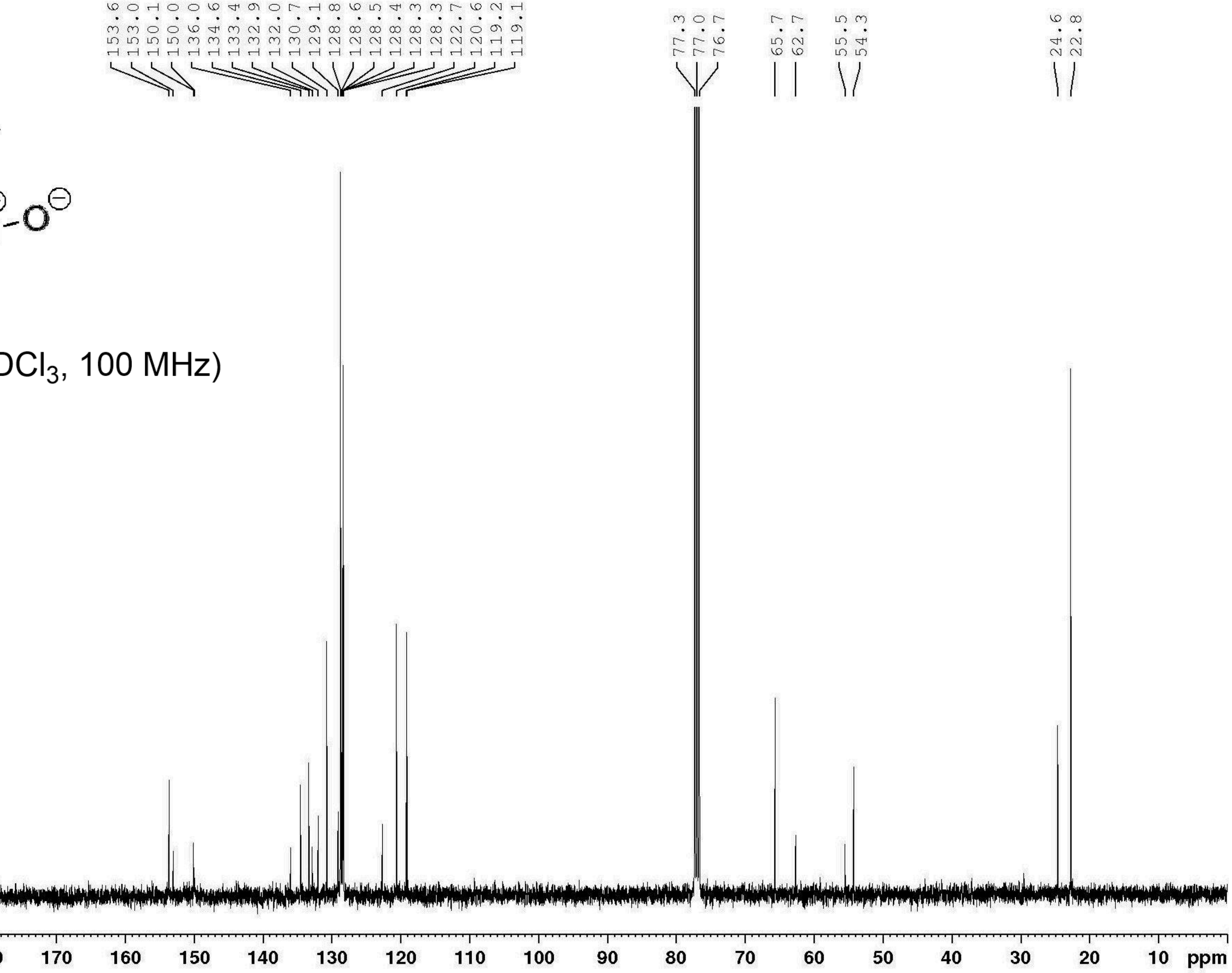

2s

E:*Z* 3.2:1

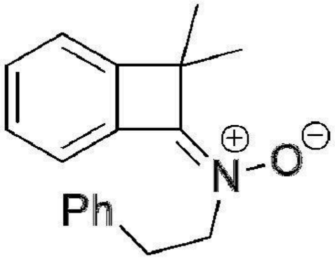

^{1}H NMR ($CDCl_3$, 400 MHz)

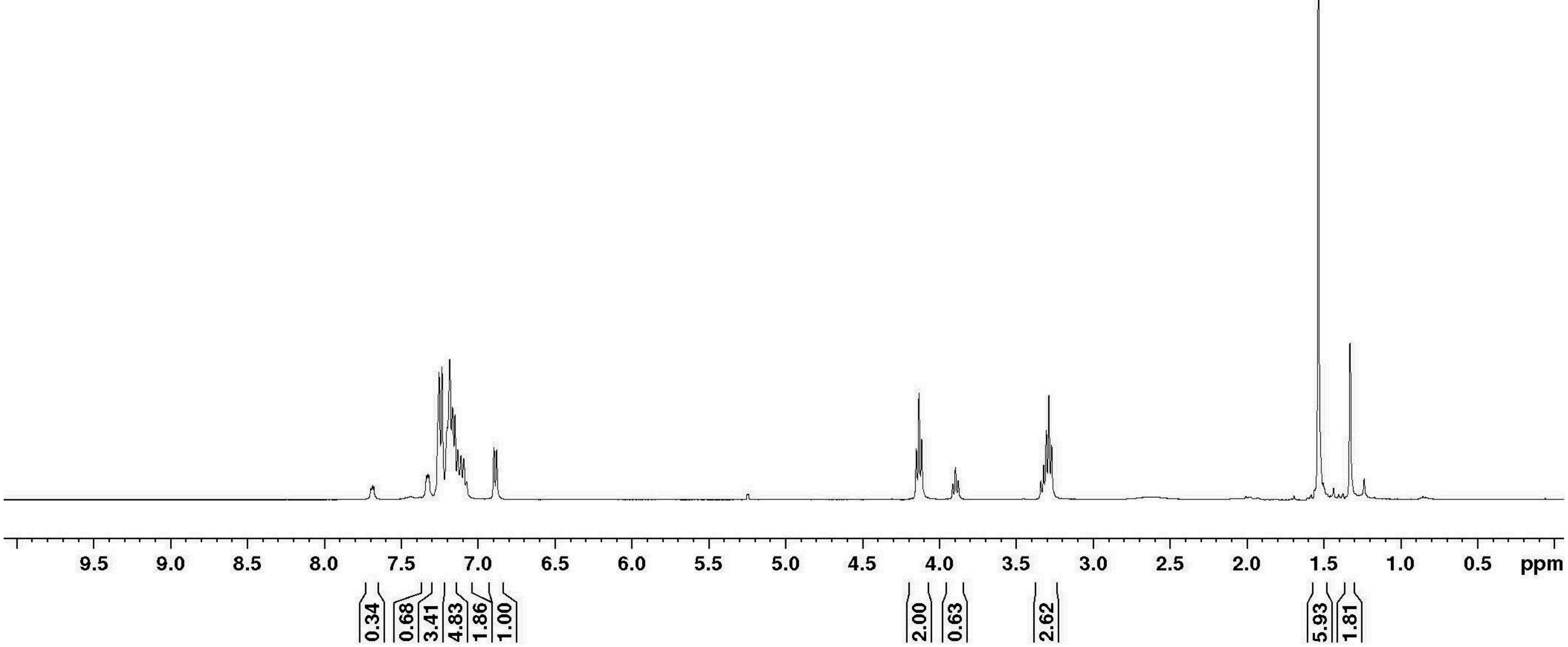

2s *E*:*Z* 3.2:1

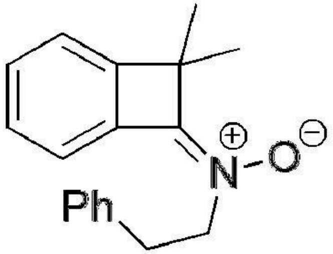

^{13}C NMR ($CDCl_3$, 100 MHz)

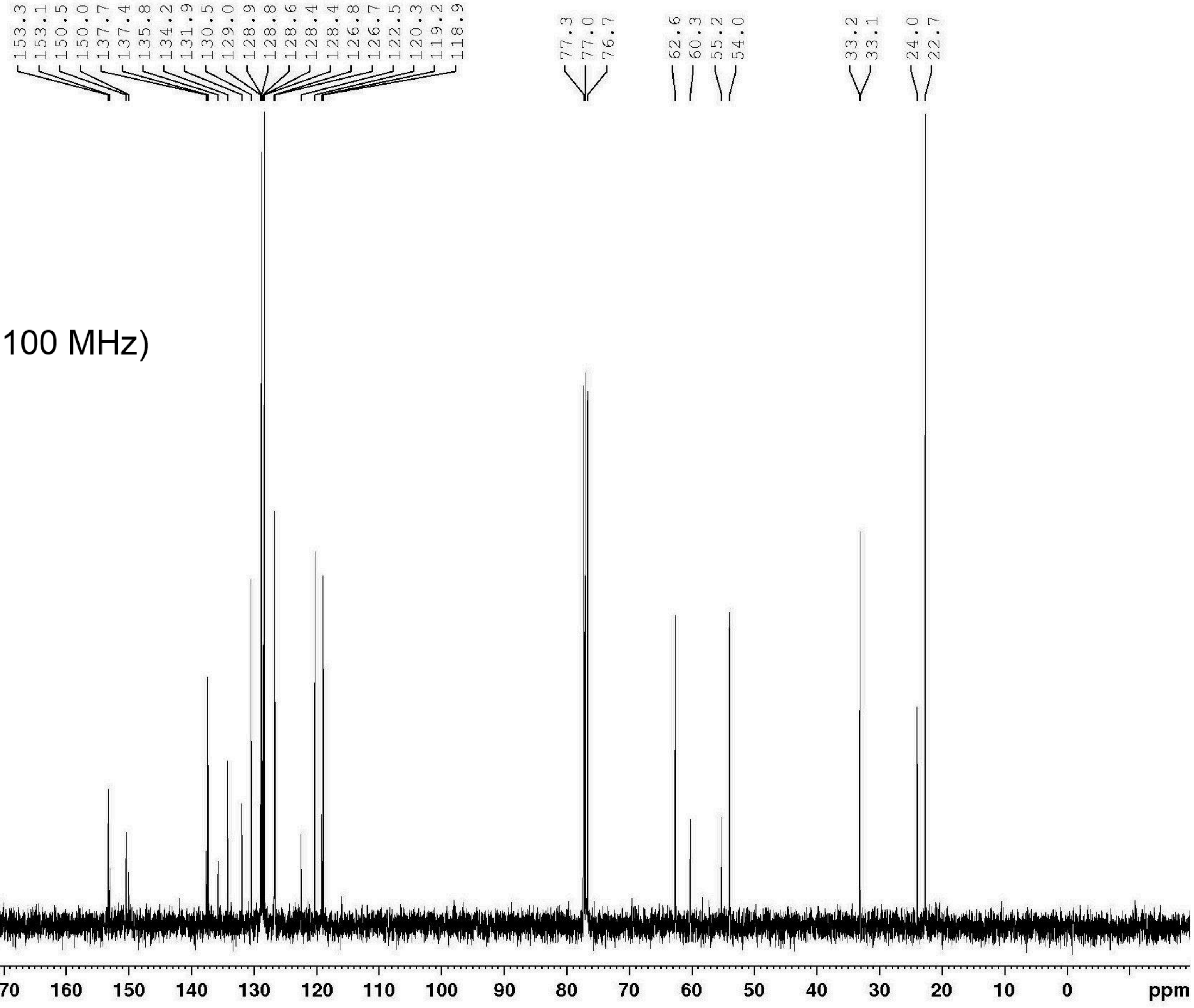

2t

E:*Z* 7.2:1

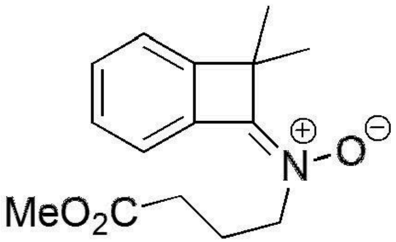

^{1}H NMR ($CDCl_3$, 400 MHz)

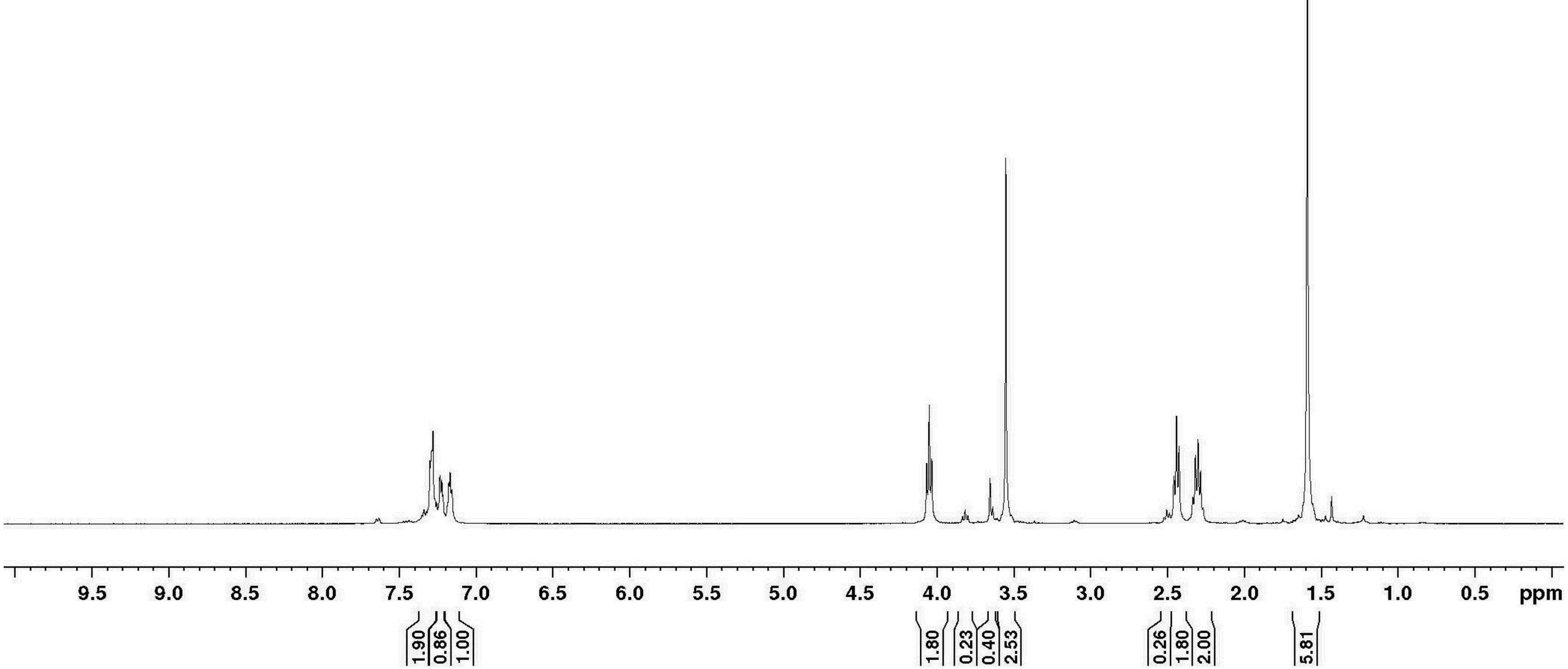

2t

E:*Z* 7.2:1

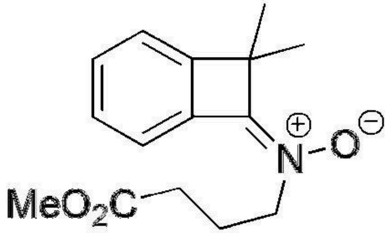

^{13}C NMR ($CDCl_3$, 100 MHz)

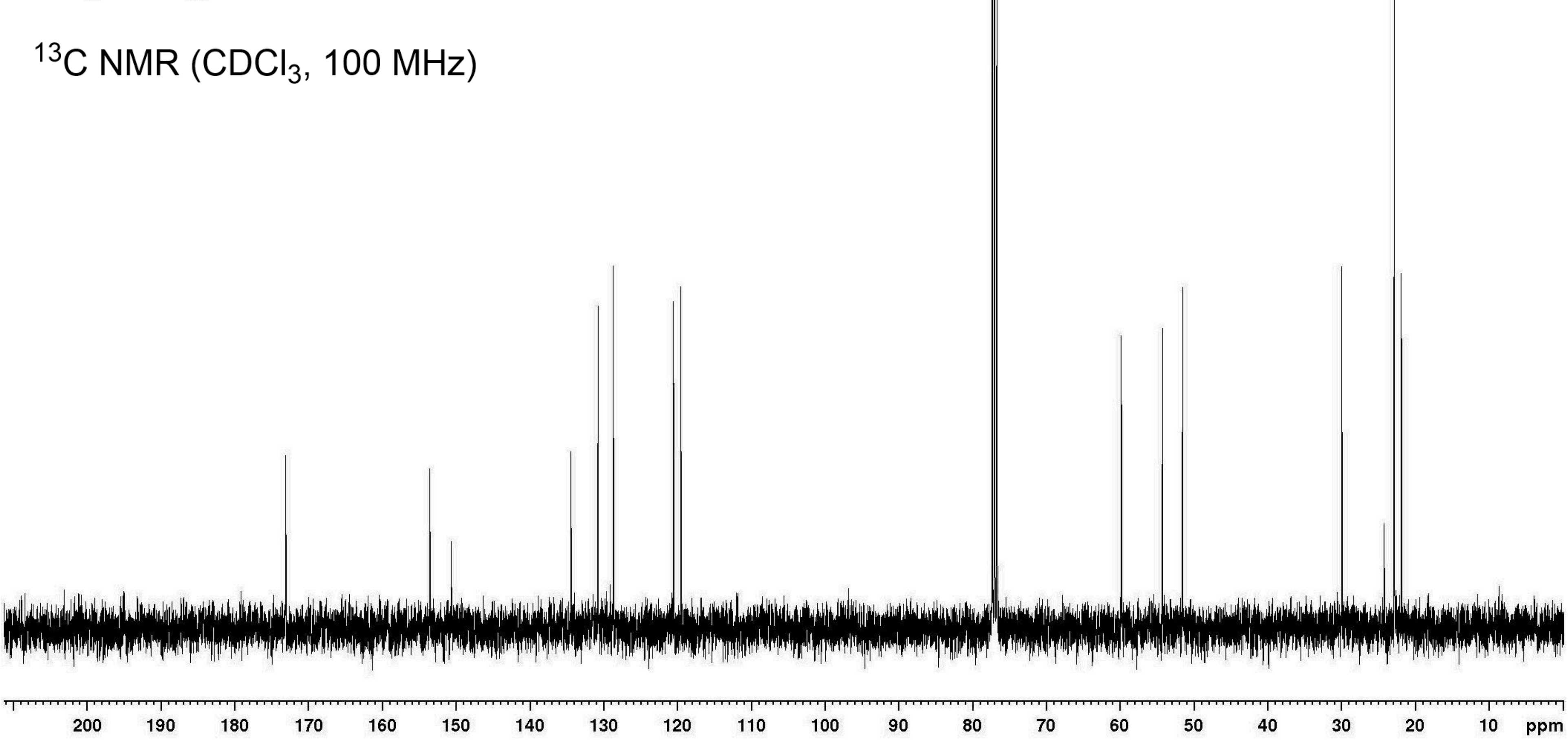

2u

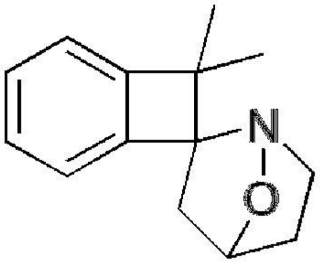

^{1}H NMR ($CDCl_3$, 400 MHz)

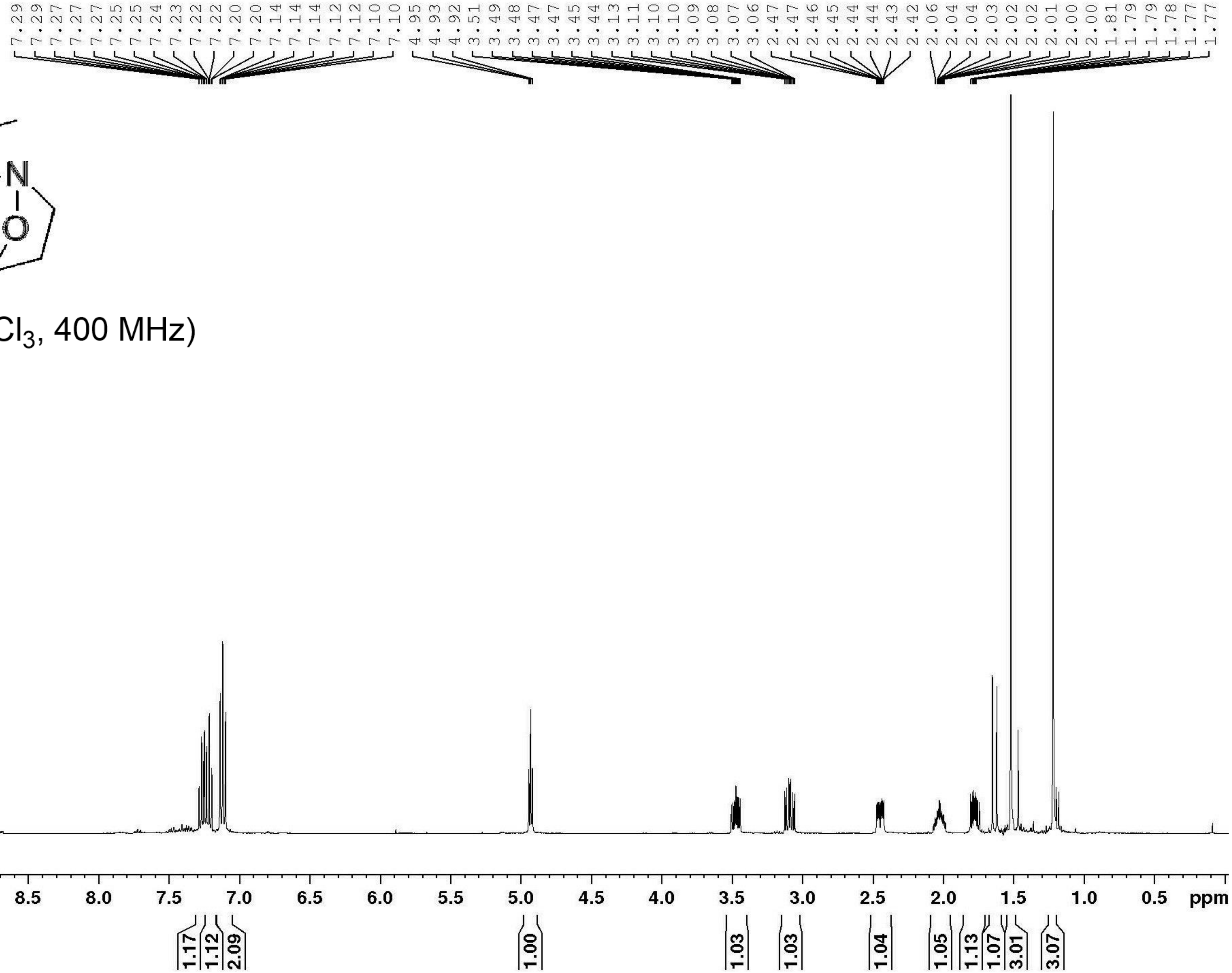

2u

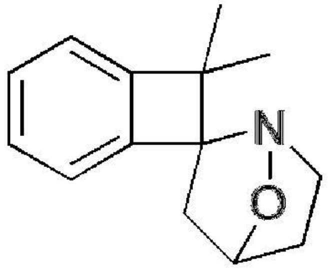

^{13}C NMR ($CDCl_3$, 100 MHz)

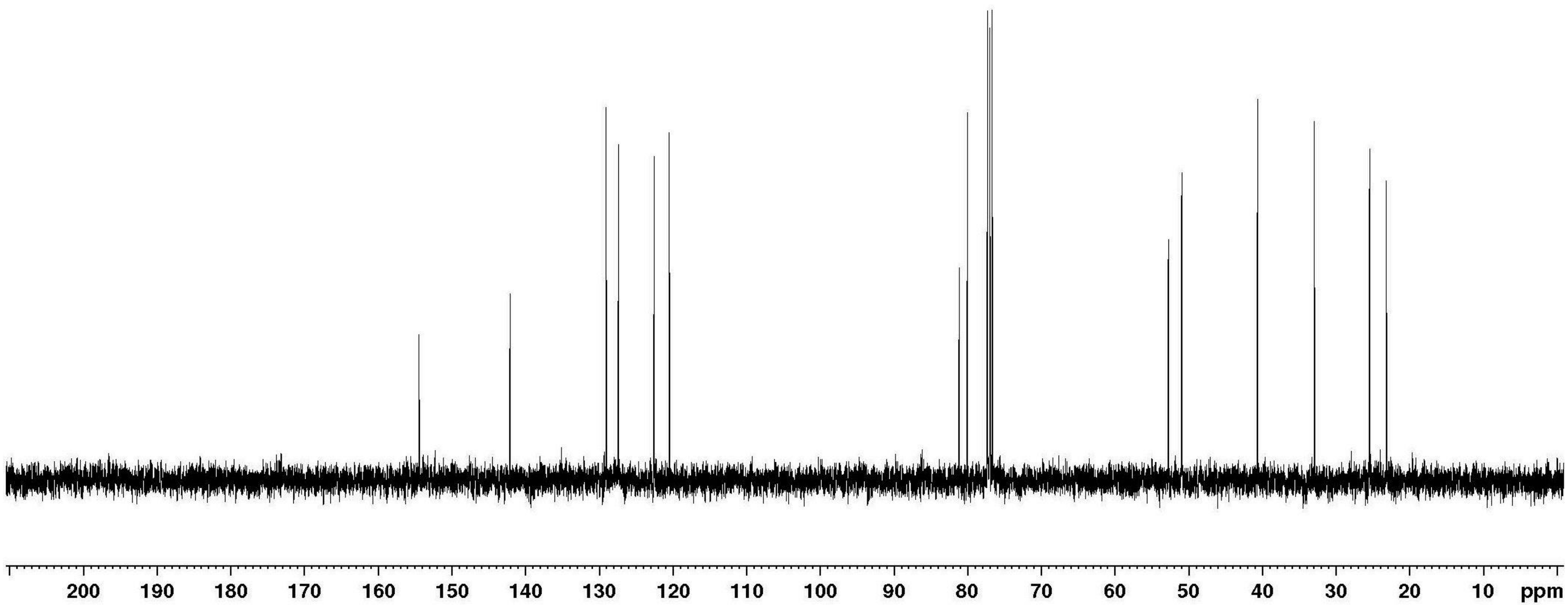

2u'

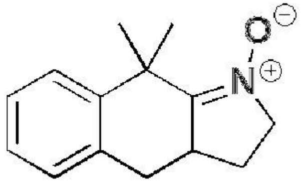

^{1}H NMR ($CDCl_3$, 400 MHz)

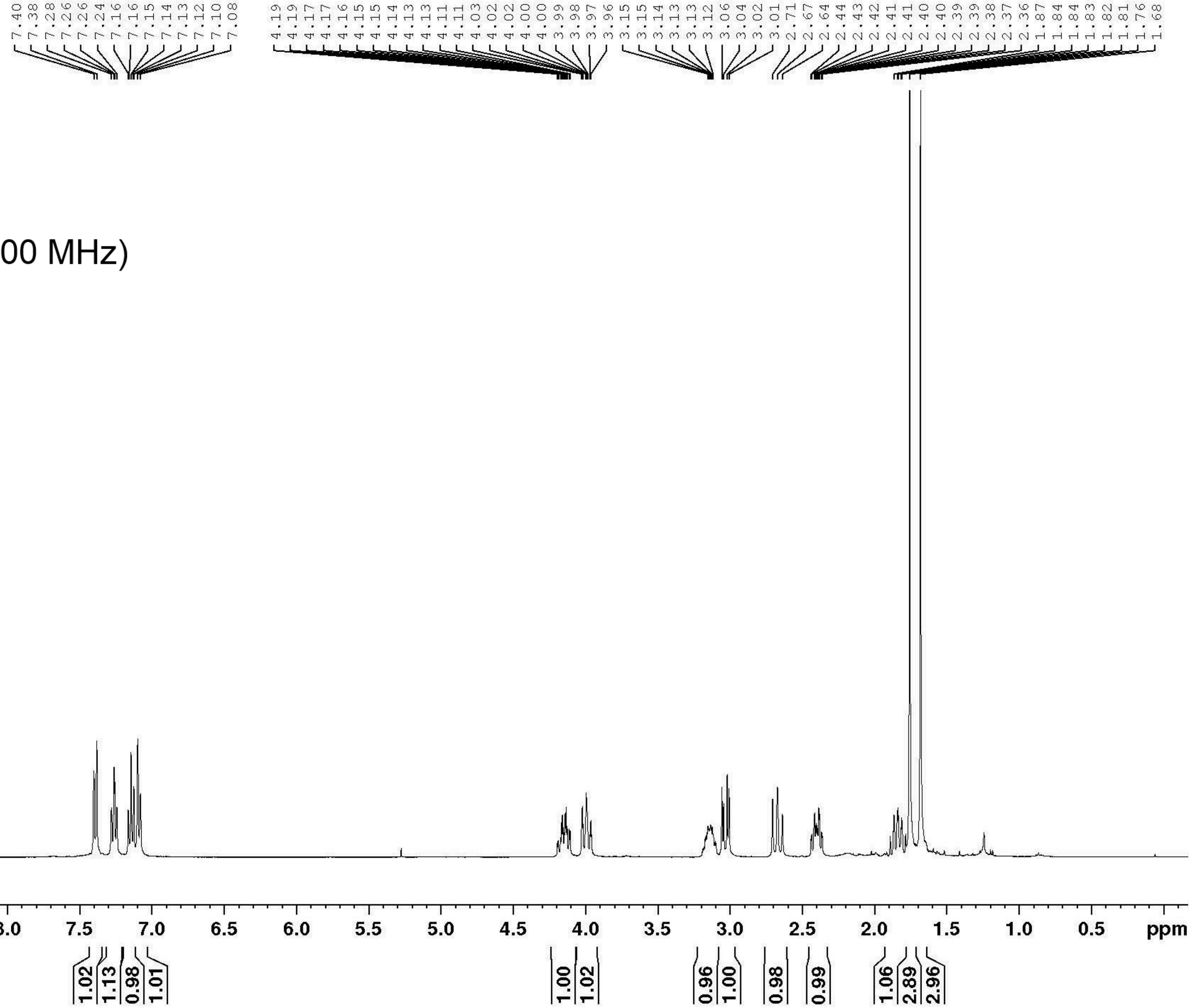

2u'

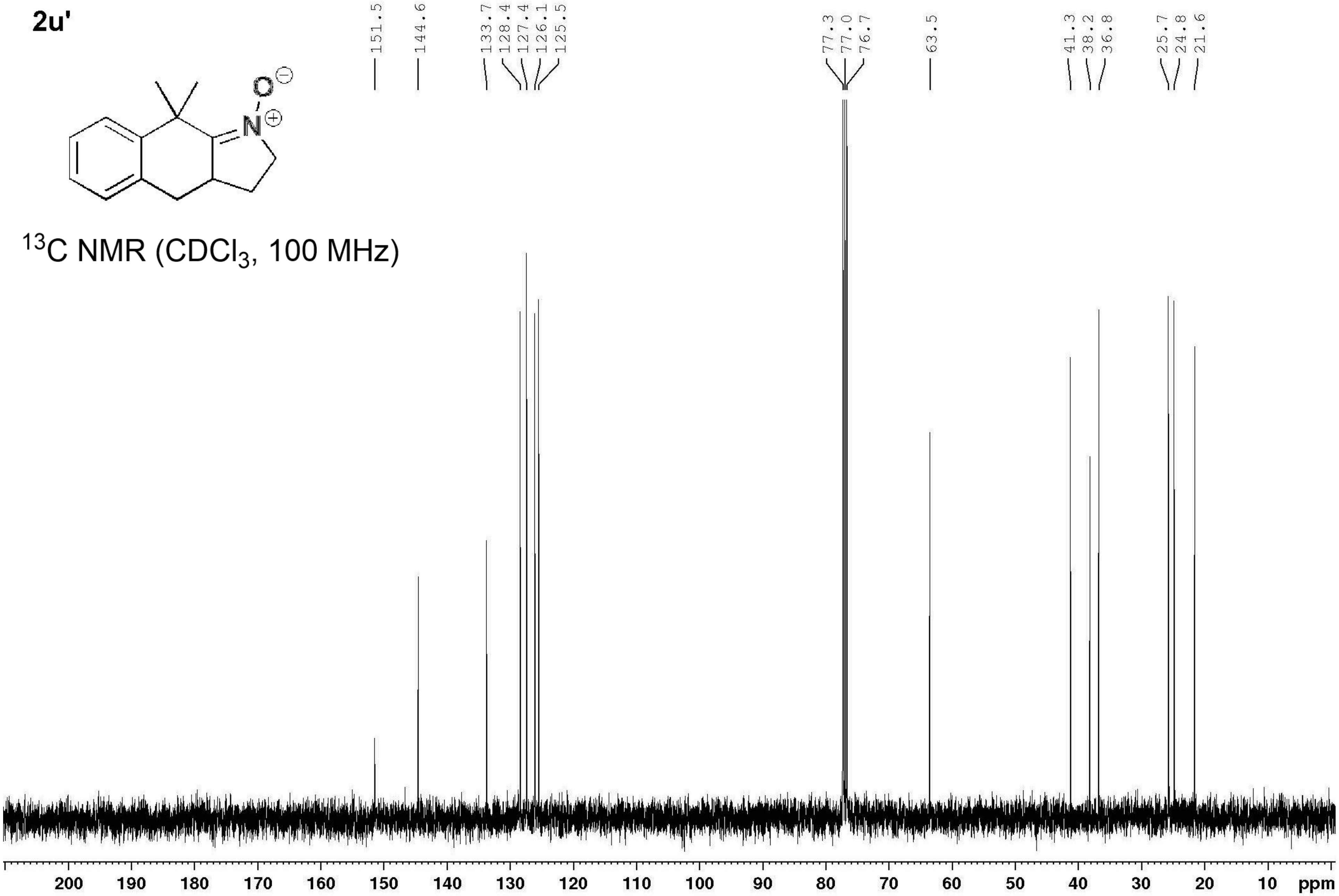

^{13}C NMR ($CDCl_3$, 100 MHz)

2v

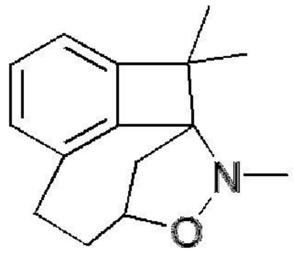

^{1}H NMR ($CDCl_3$, 400 MHz)

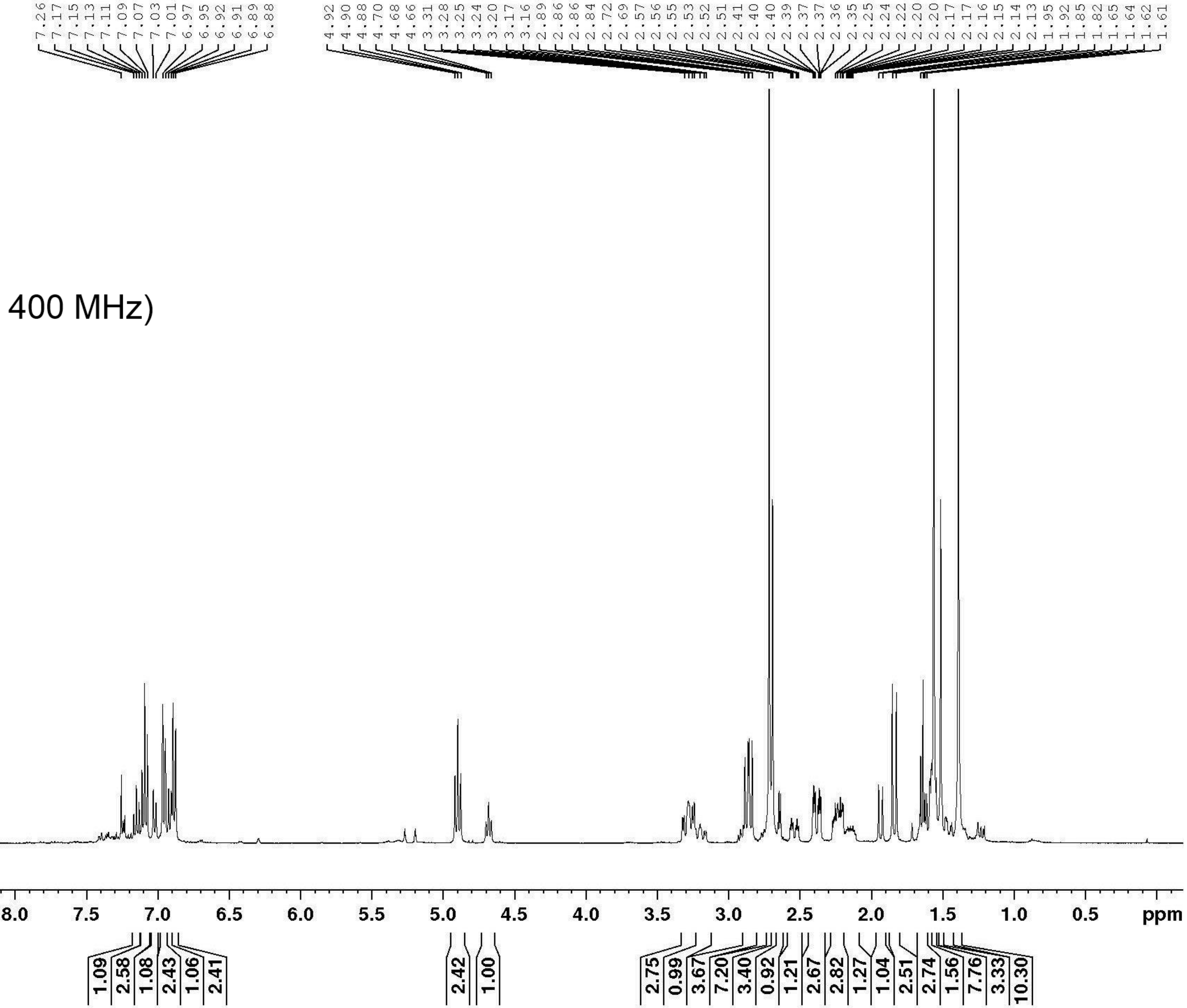

2v

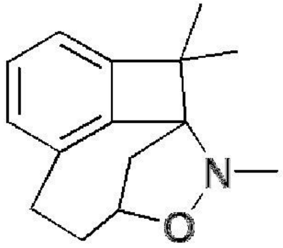

^{13}C NMR ($CDCl_3$, 100 MHz)

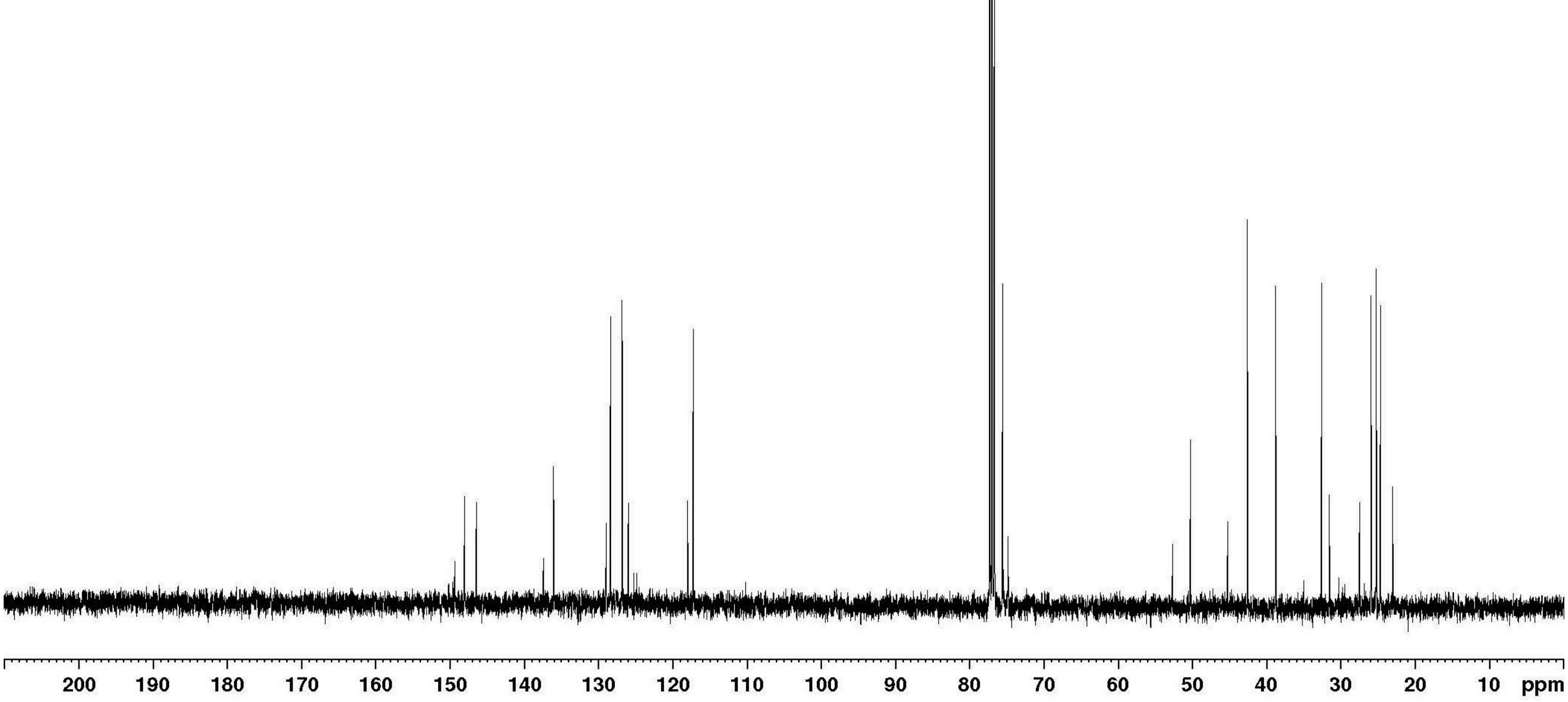

2v'

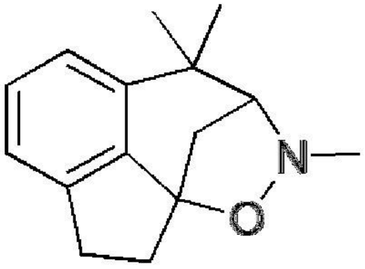

^{1}H NMR ($CDCl_3$, 400 MHz)

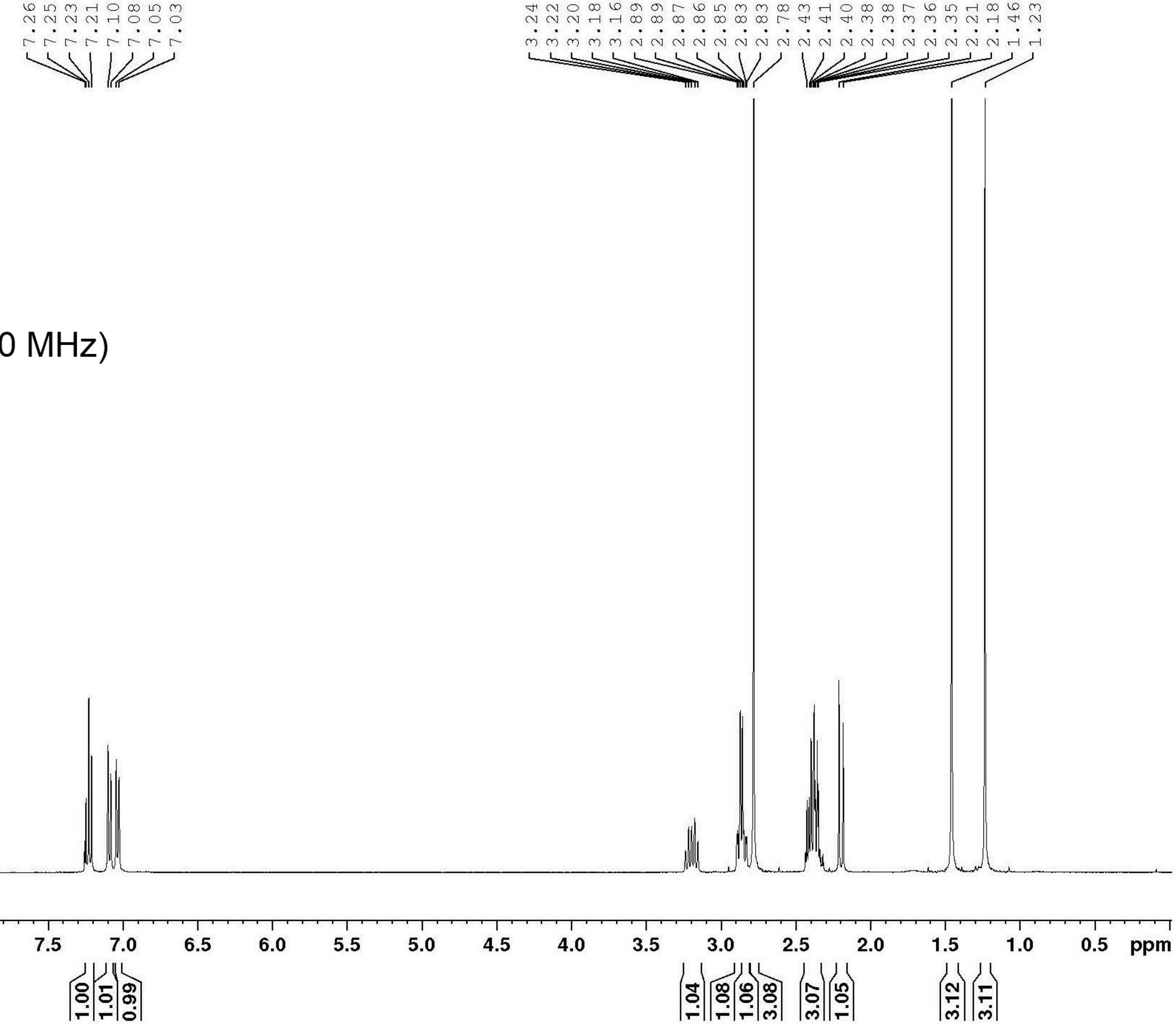

2v'

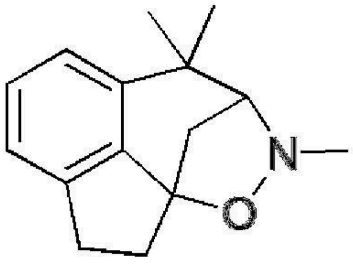

^{13}C NMR ($CDCl_3$, 100 MHz)

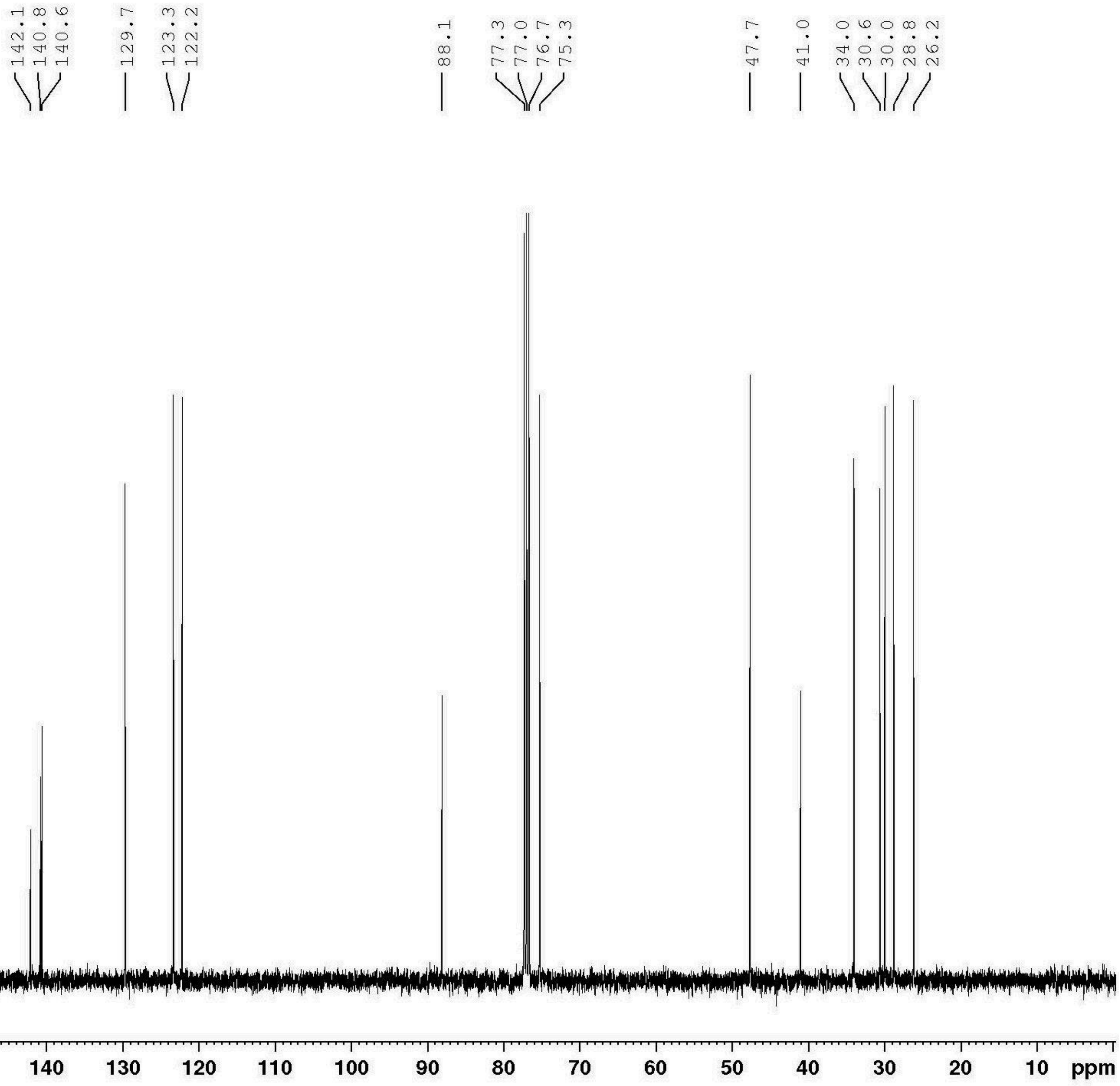

3

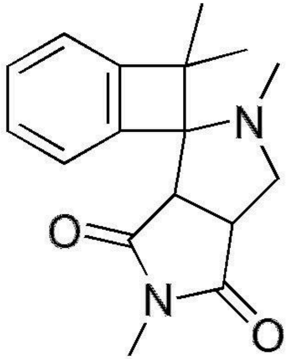

^{1}H NMR (CDCl$_3$, 400 MHz)

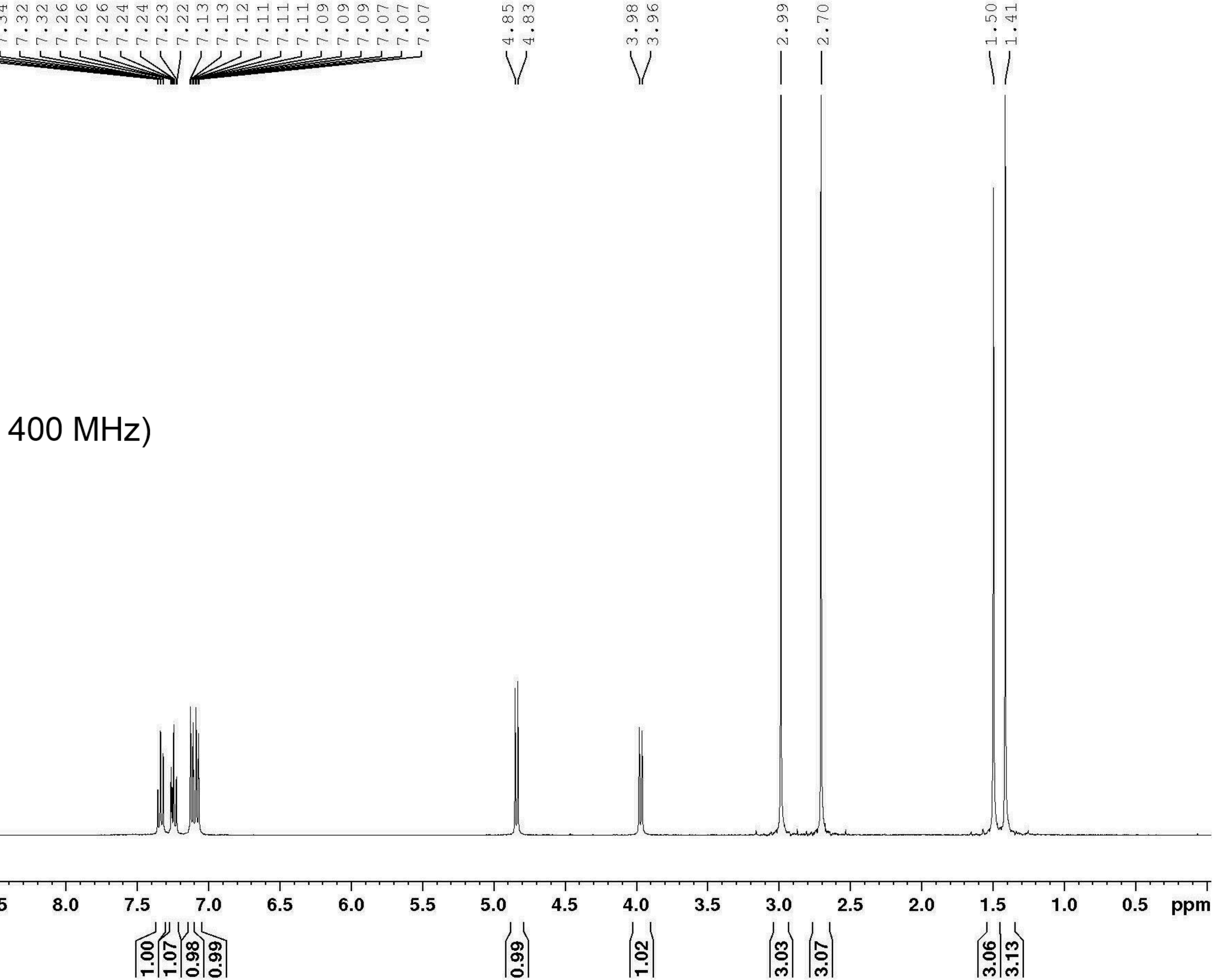

3

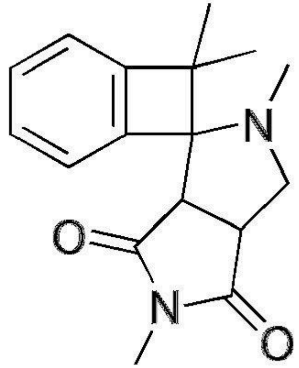

^{13}C NMR ($CDCl_3$, 100 MHz)

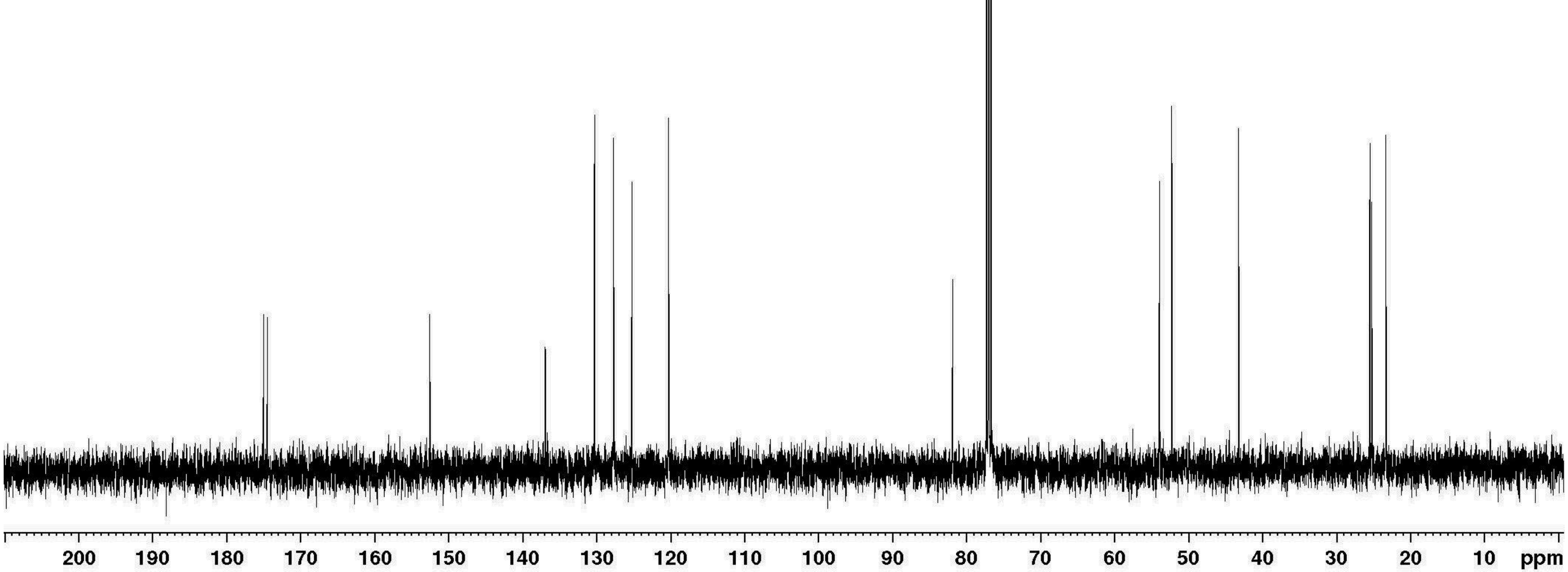

4

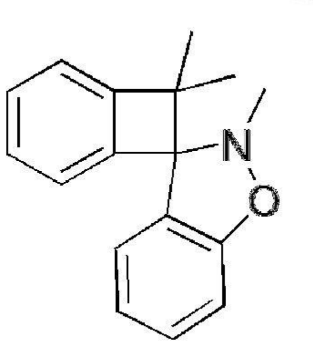

^{1}H NMR ($CDCl_3$, 400 MHz)

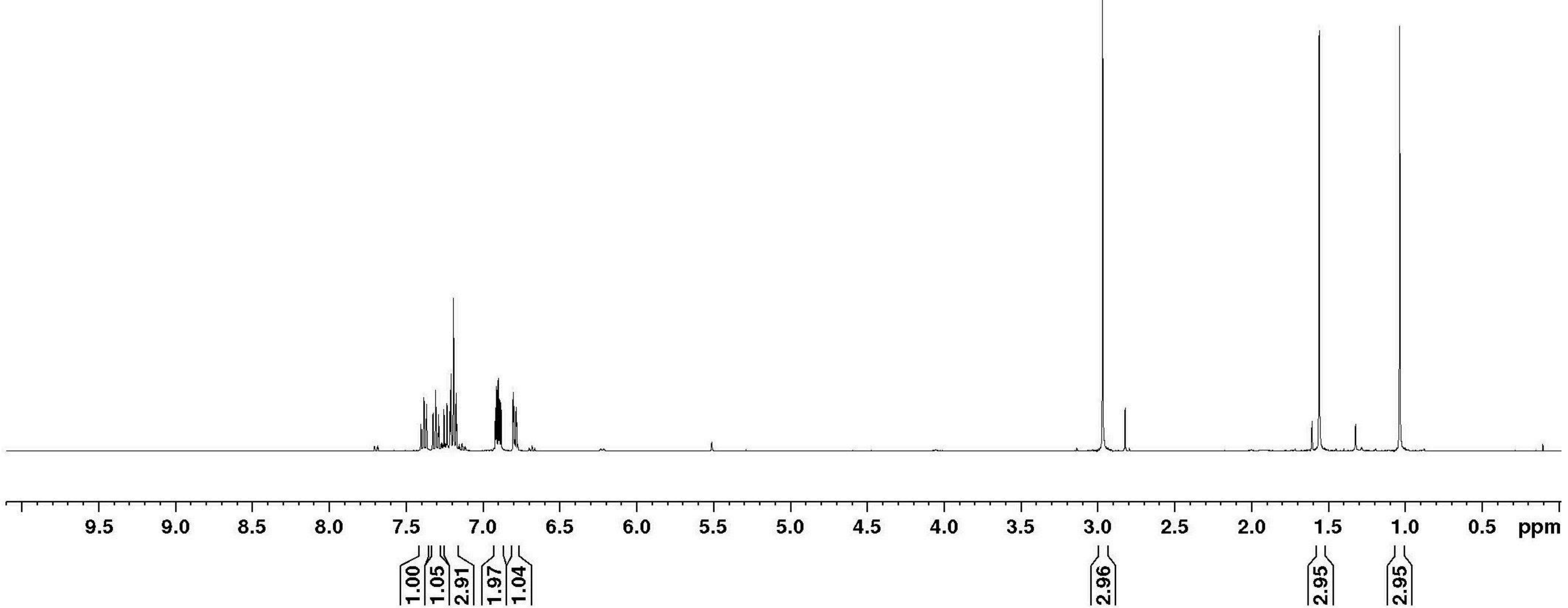

4

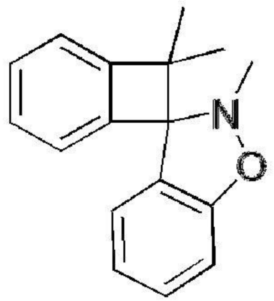

^{13}C NMR ($CDCl_3$, 100 MHz)

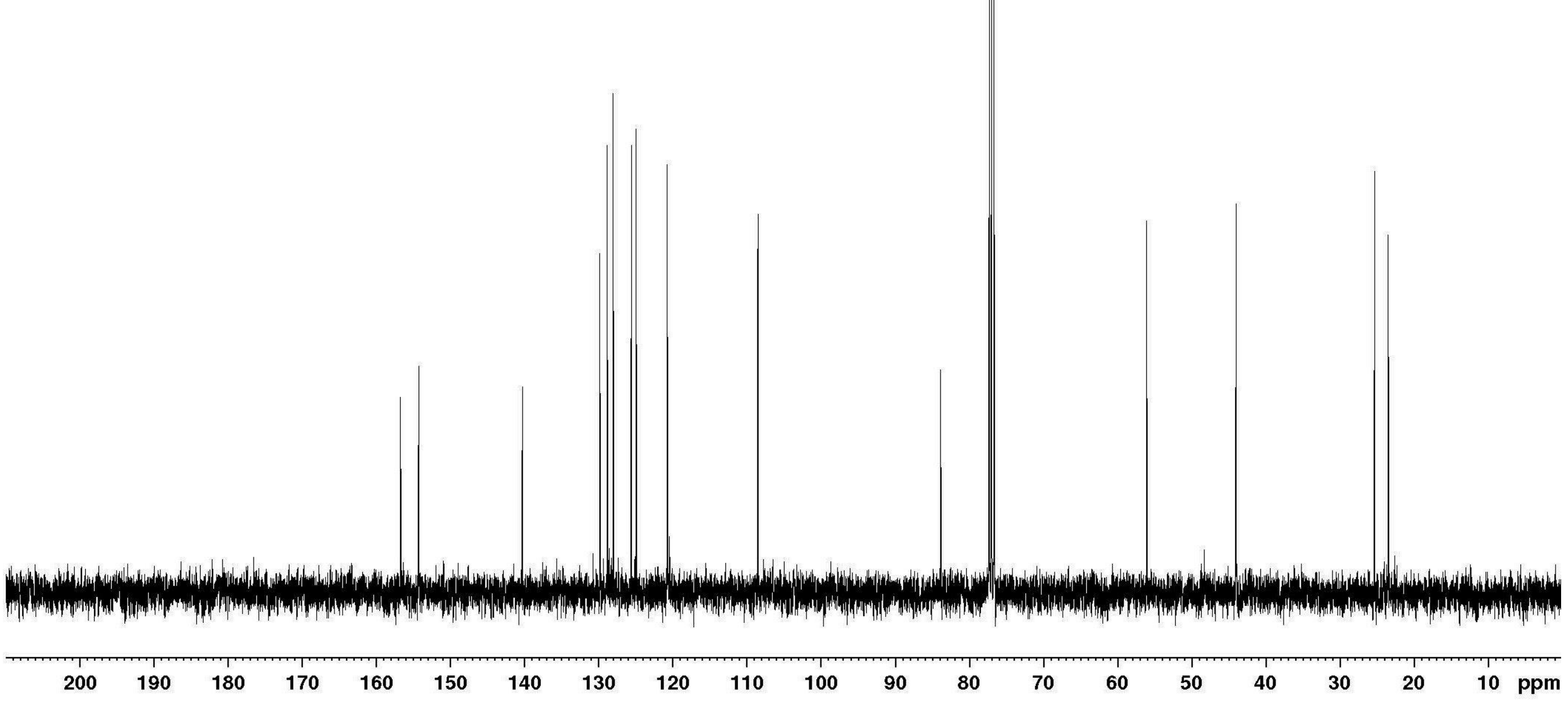

5

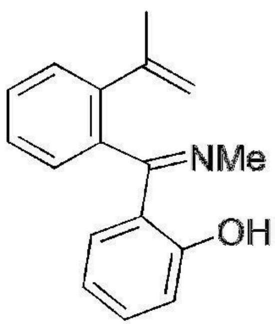

^{1}H NMR ($CDCl_3$, 400 MHz)

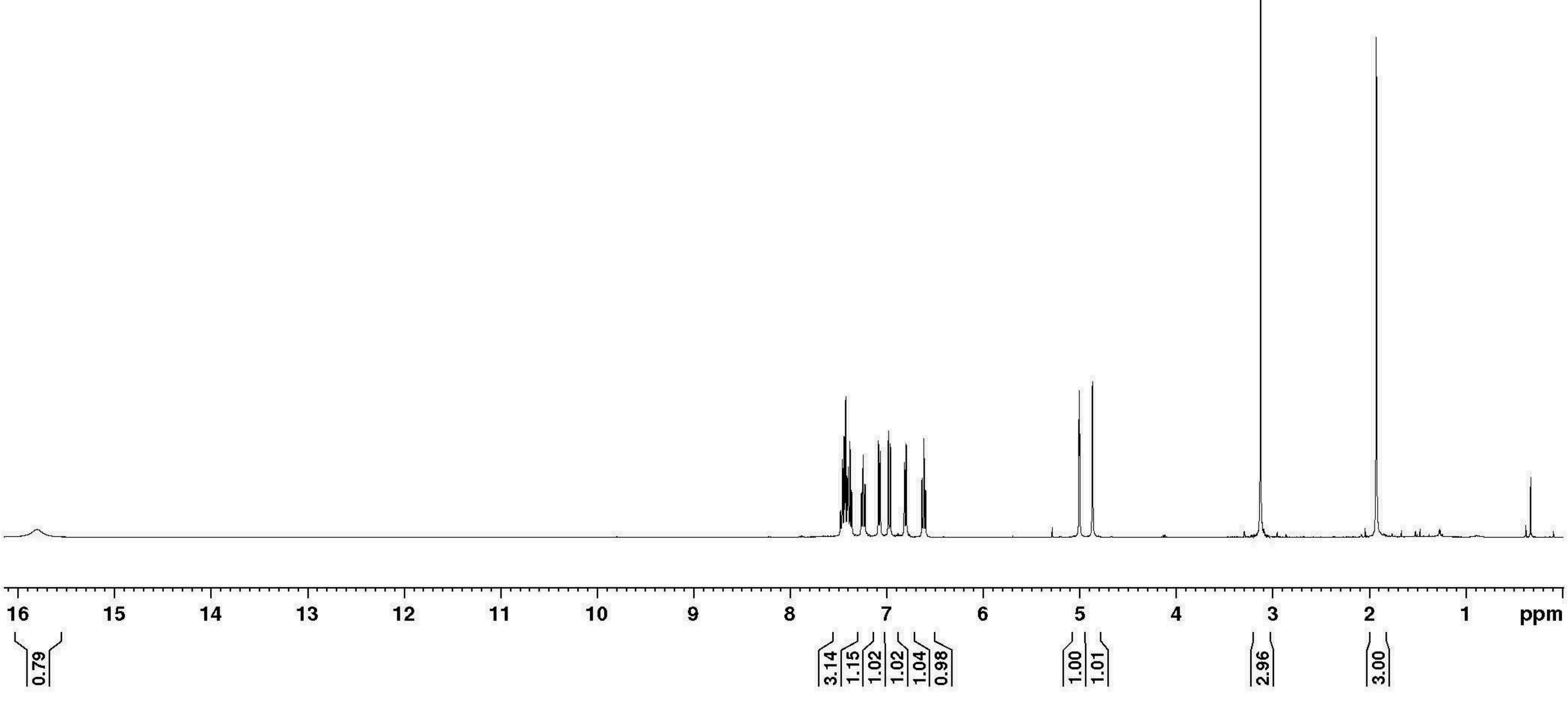

5

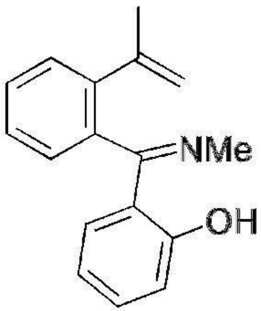

^{13}C NMR ($CDCl_3$, 100 MHz)

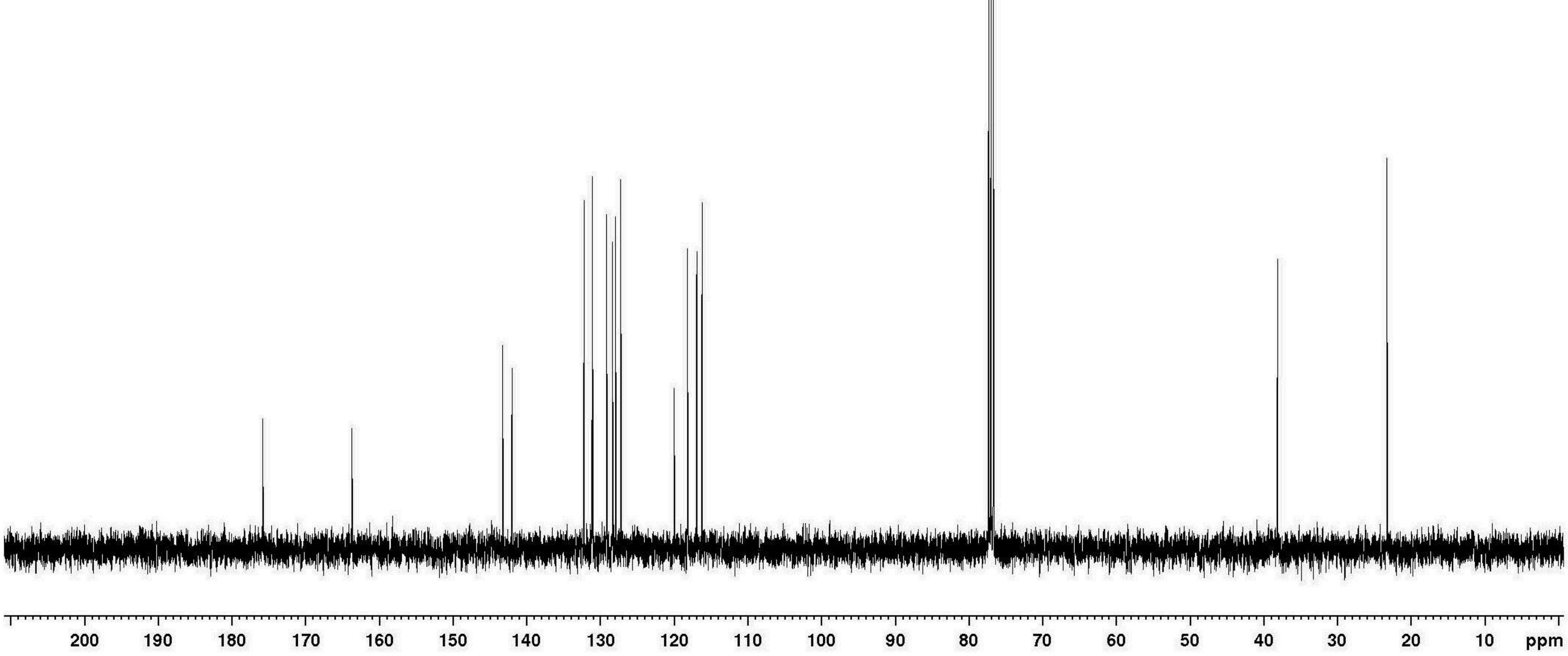

6

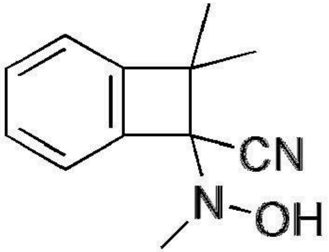

^{1}H NMR ($CDCl_3$, 400 MHz)

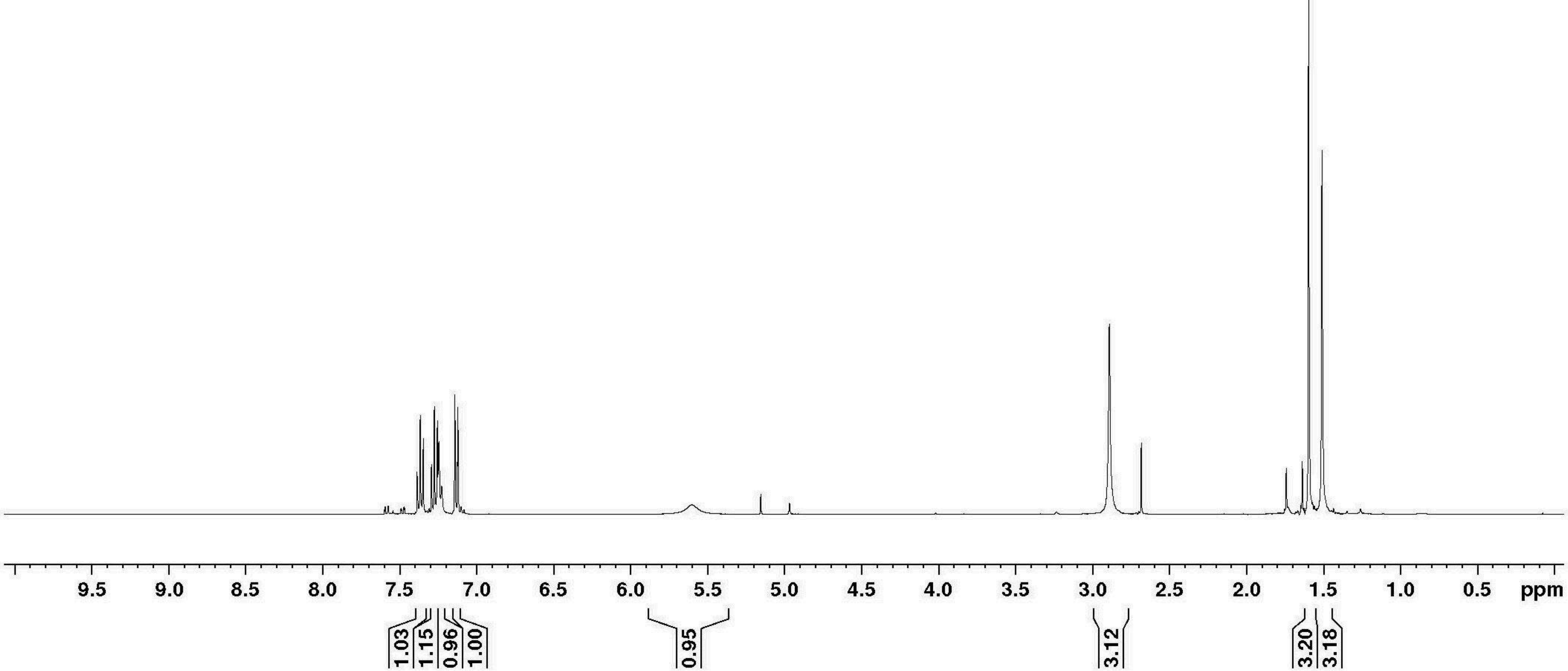

6

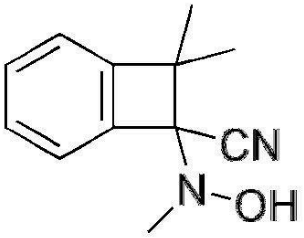

^{13}C NMR ($CDCl_3$, 100 MHz)

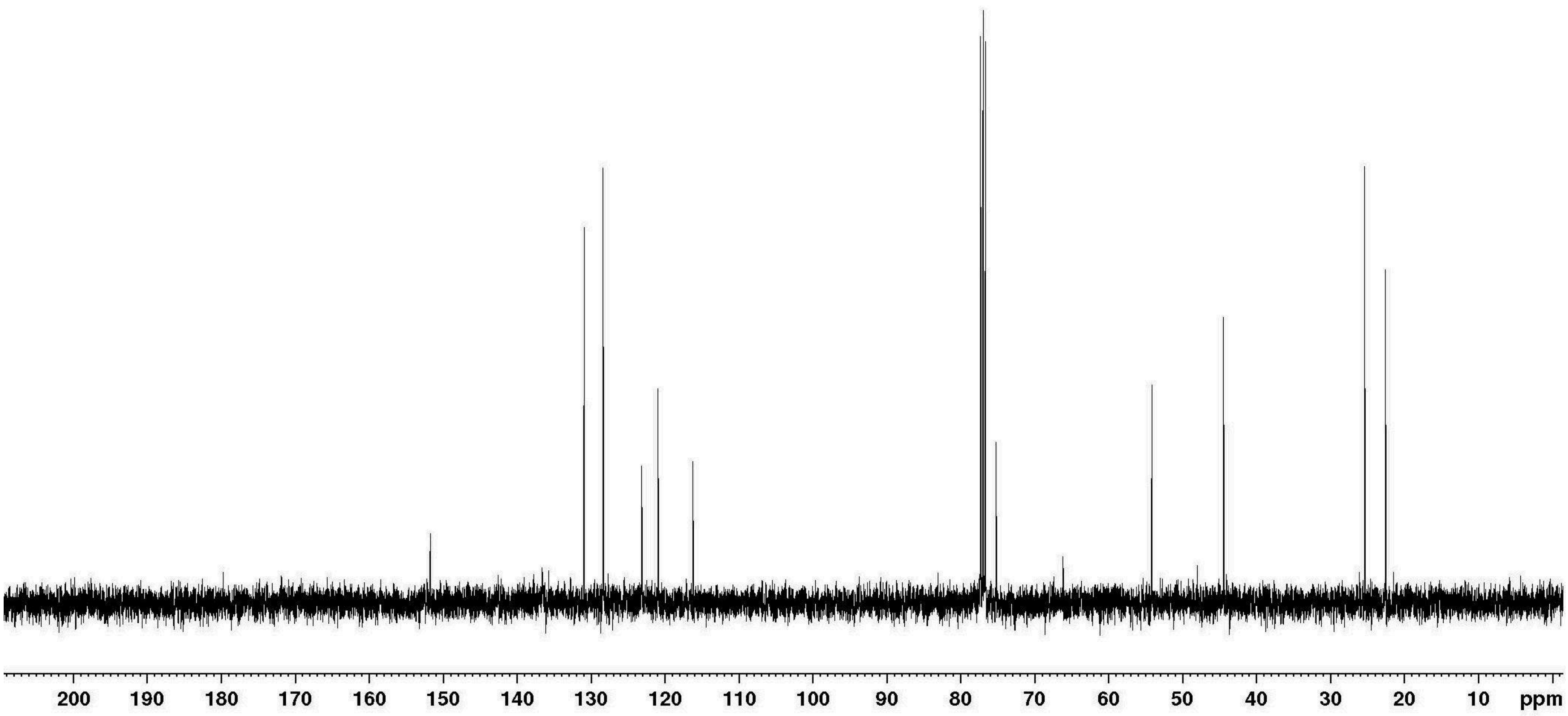

7

dr 6.3:1

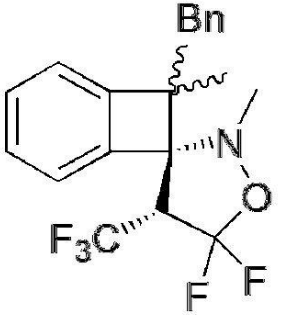

^1H NMR ($CDCl_3$, 400 MHz)

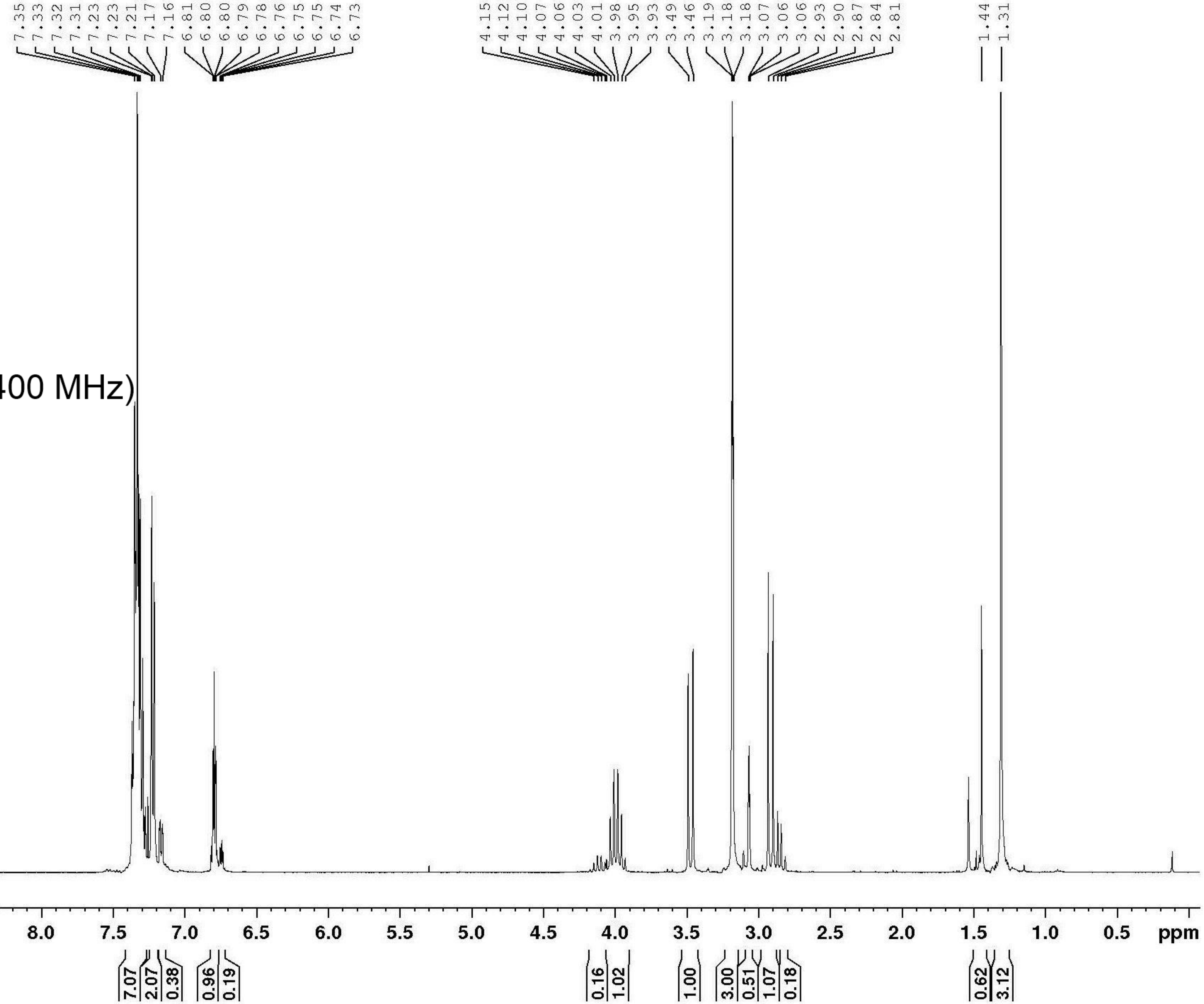

7
dr 6.3:1

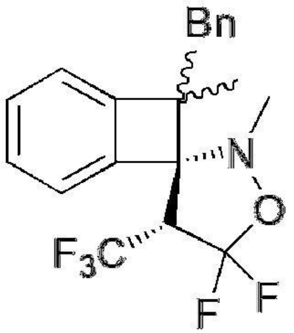

^{13}C NMR ($CDCl_3$, 100 MHz)

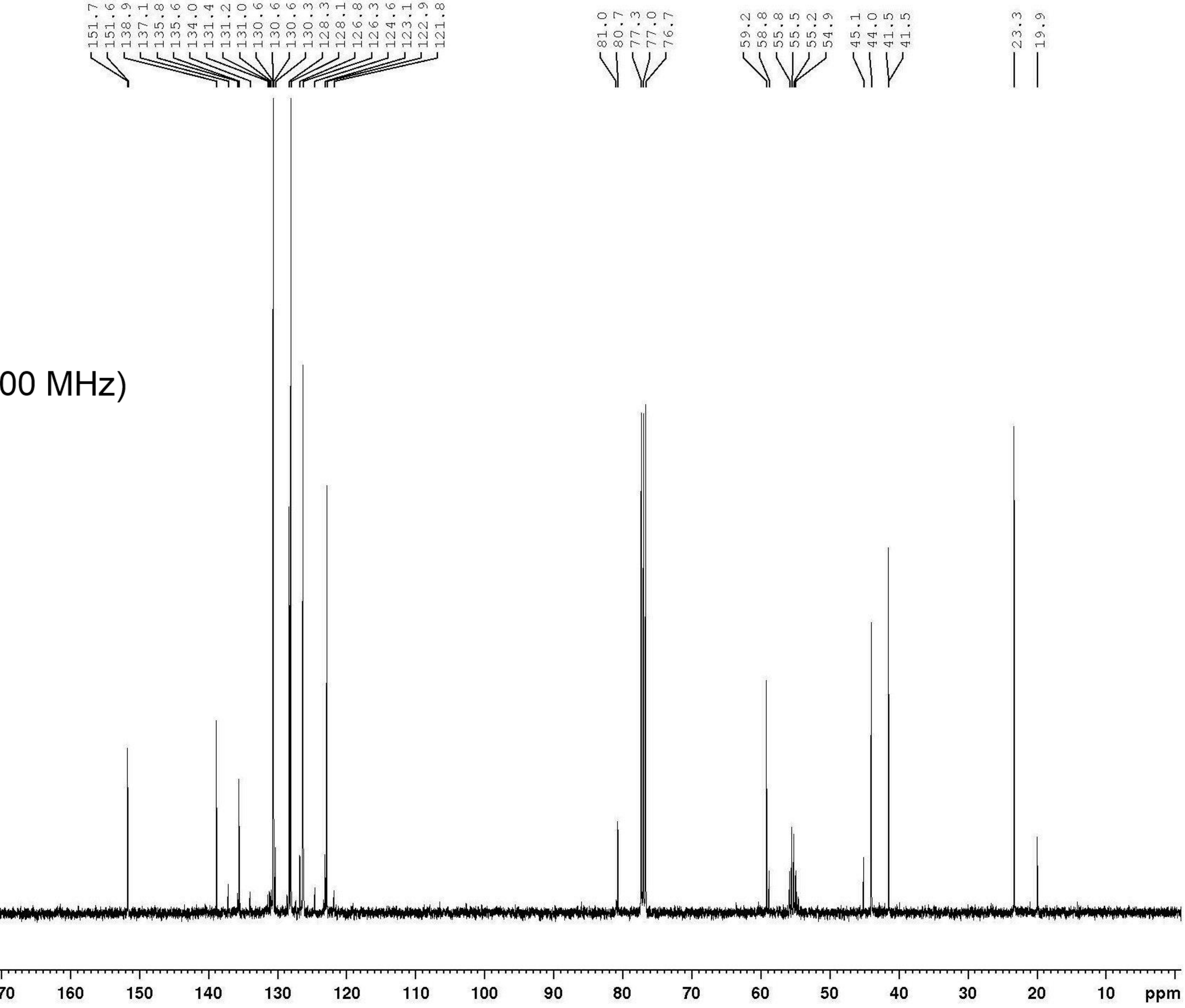

7
dr 6.3:1

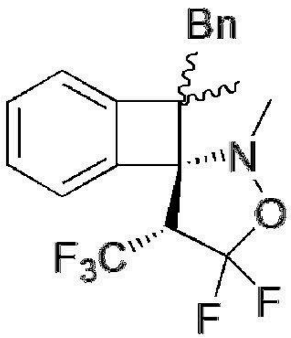

^{19}F NMR ($CDCl_3$, 376 MHz)

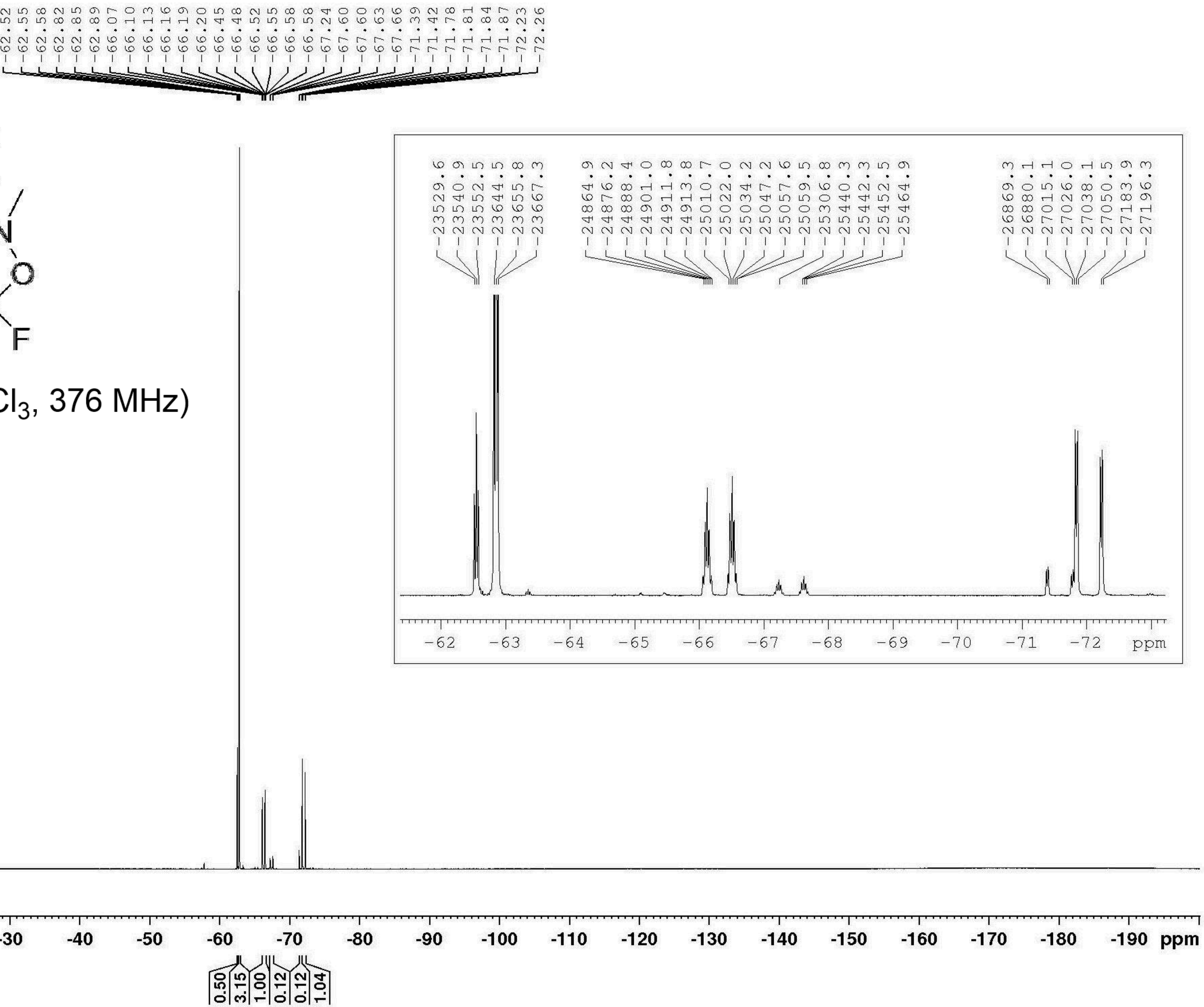

8

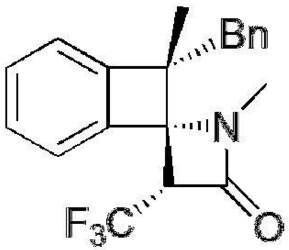

^1H NMR ($CDCl_3$, 400 MHz)

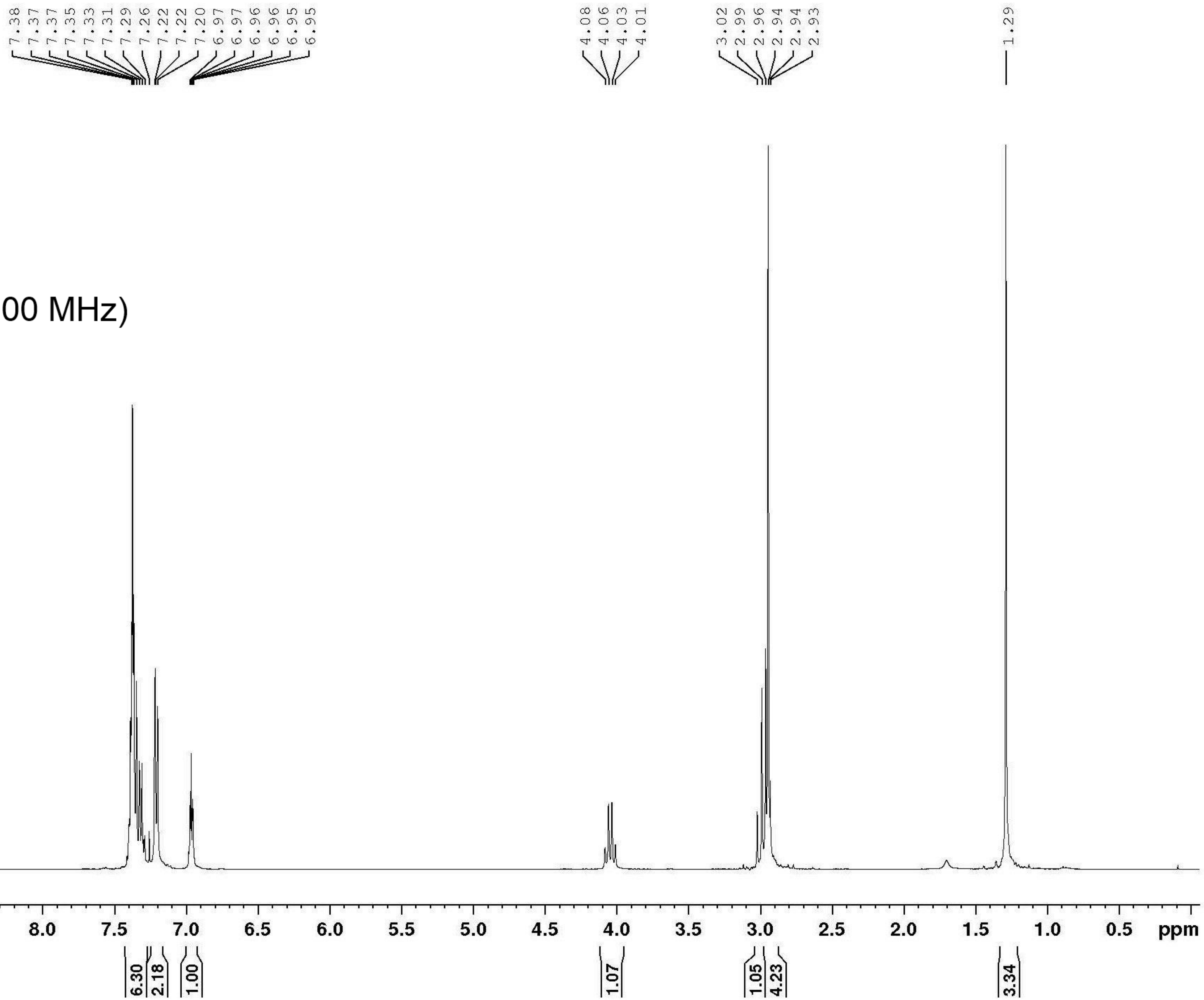

8

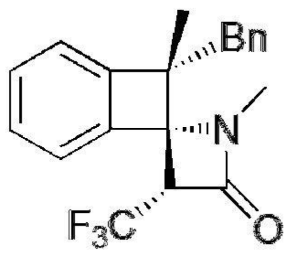

^{13}C NMR ($CDCl_3$, 100 MHz)

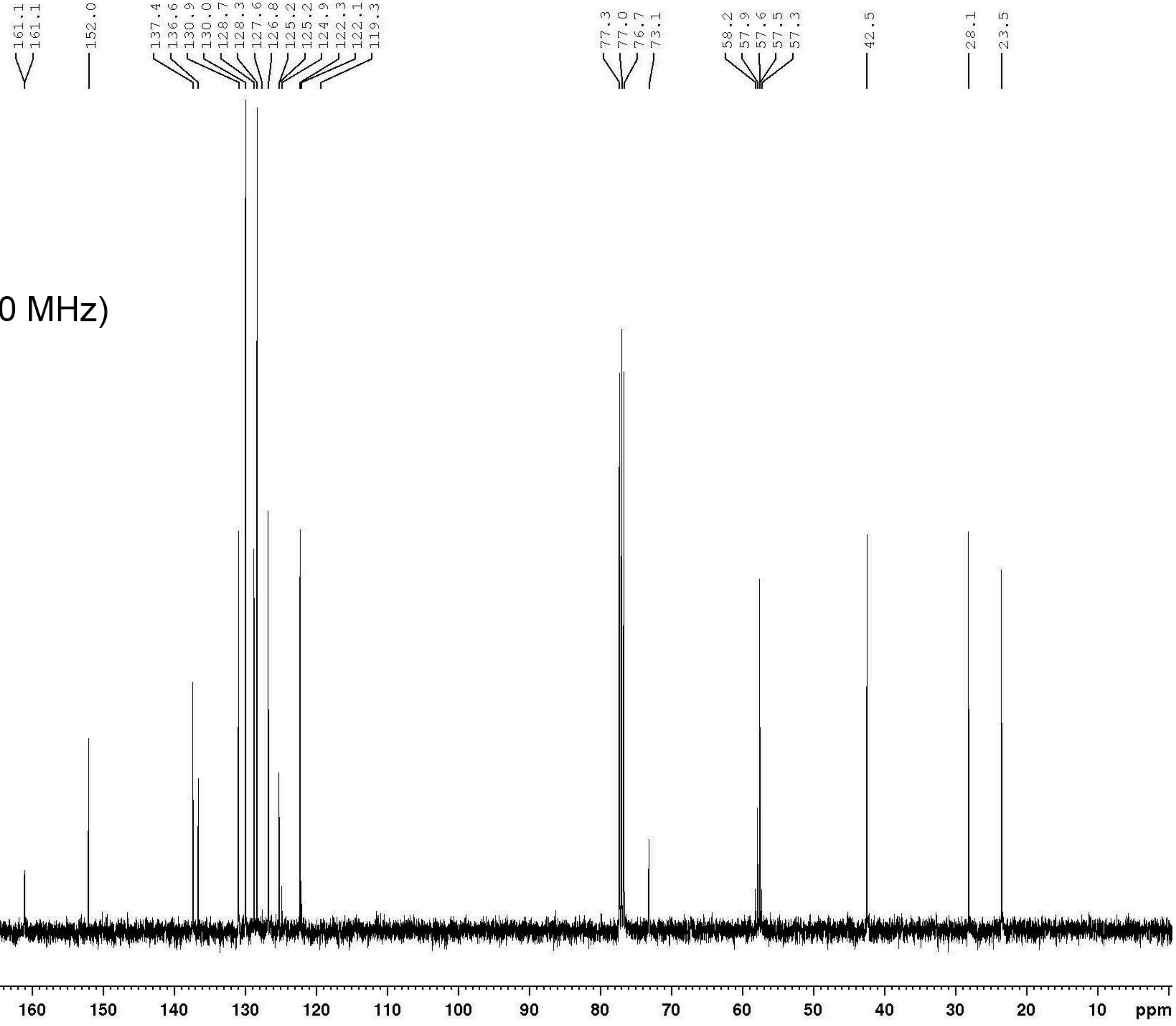

8

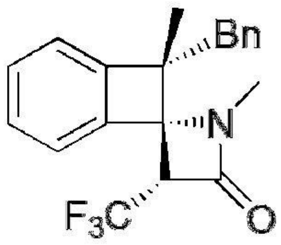

^{19}F NMR ($CDCl_3$, 376 MHz)

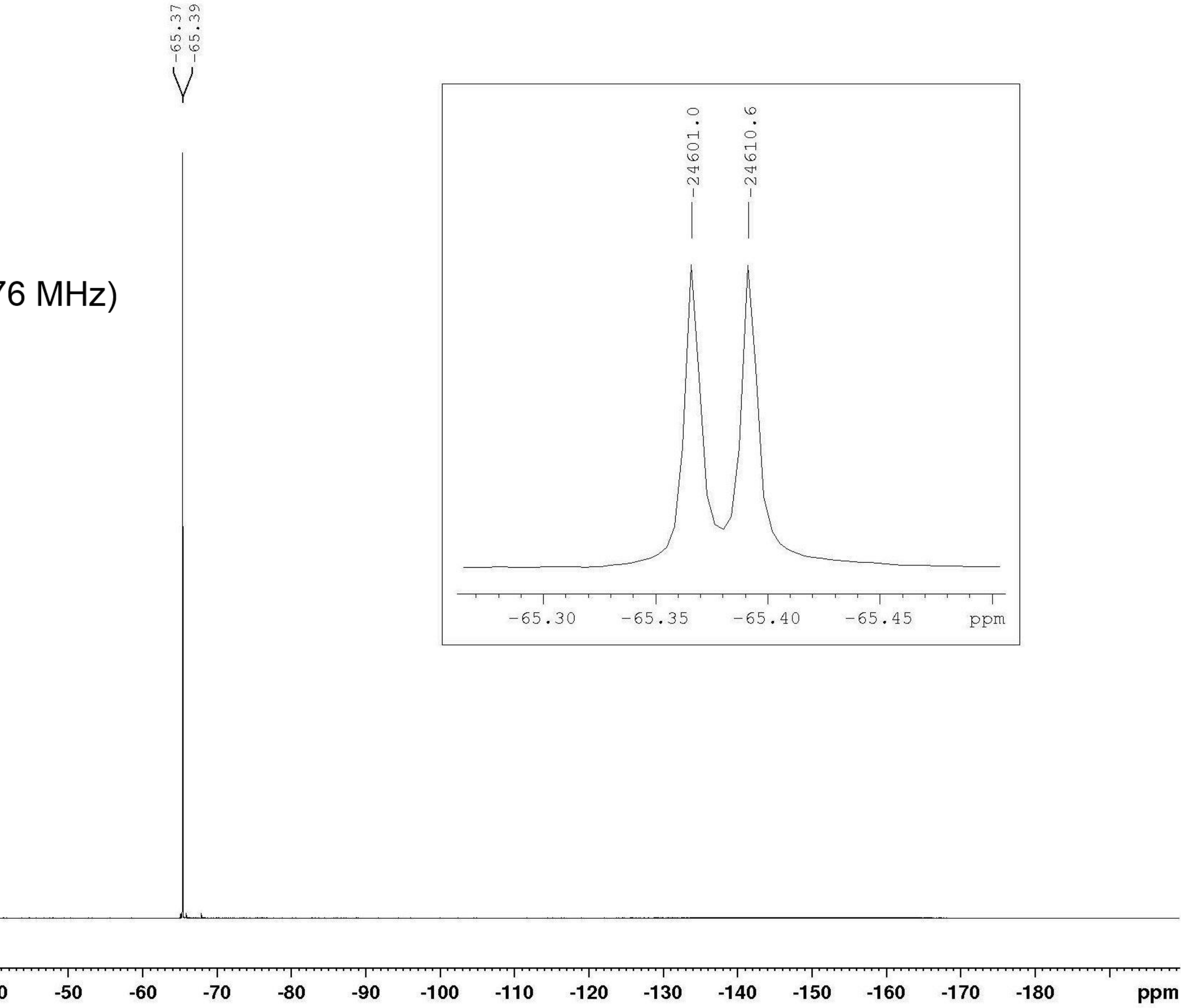

8'

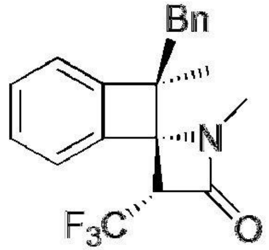

^{1}H NMR ($CDCl_3$, 400 MHz)

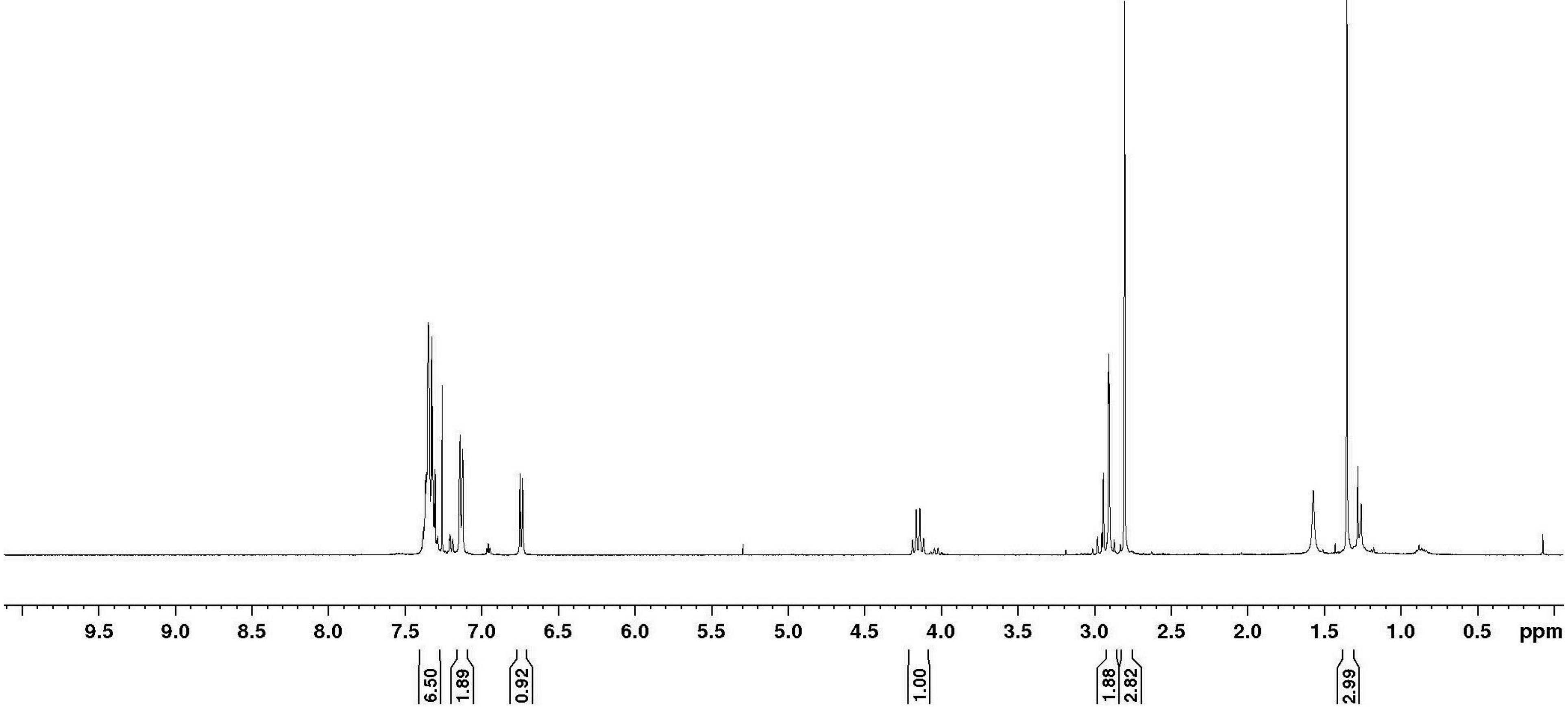

8'

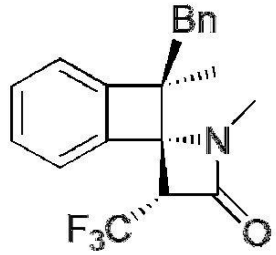

^{13}C NMR (CDCl$_3$, 100 MHz)

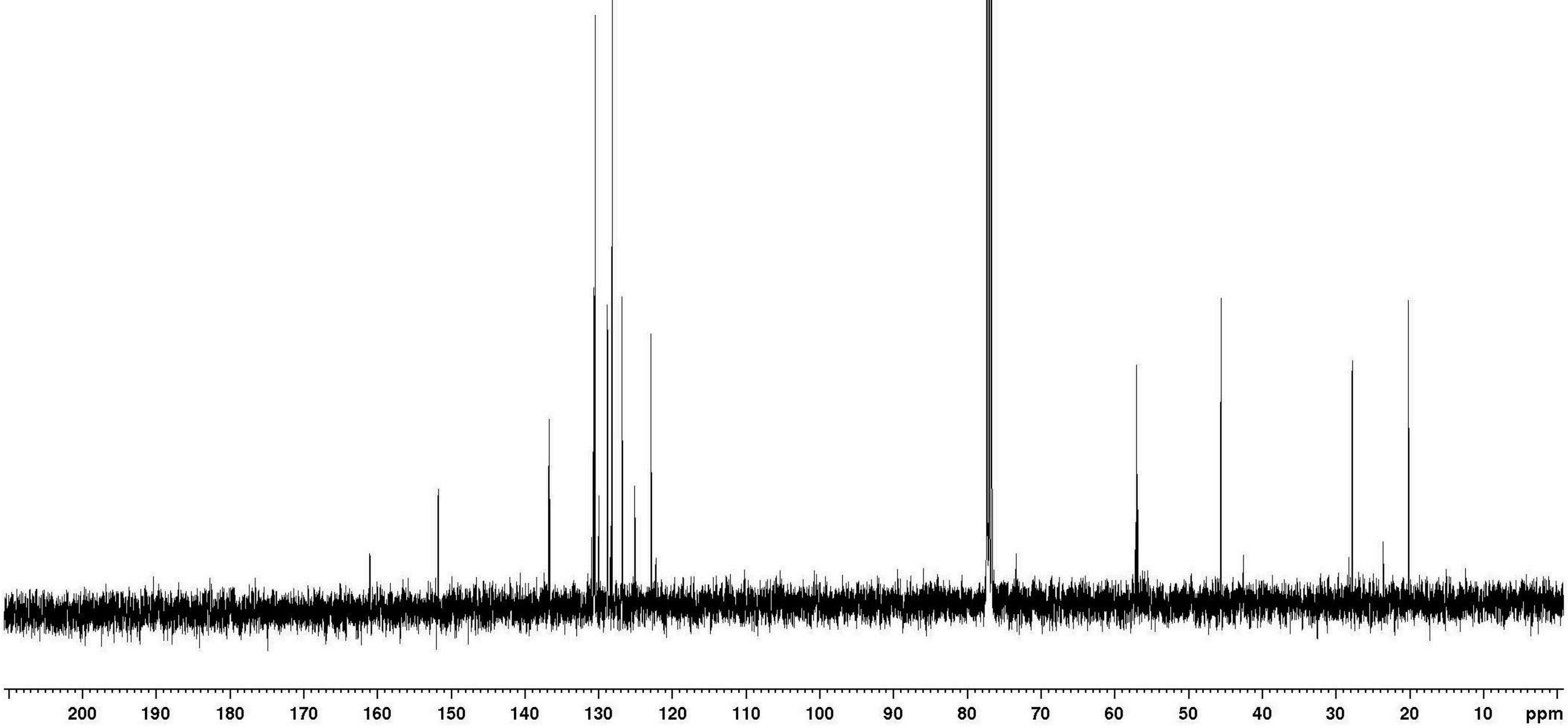

8'

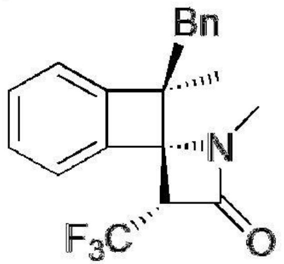

^{19}F NMR ($CDCl_3$, 376 MHz)

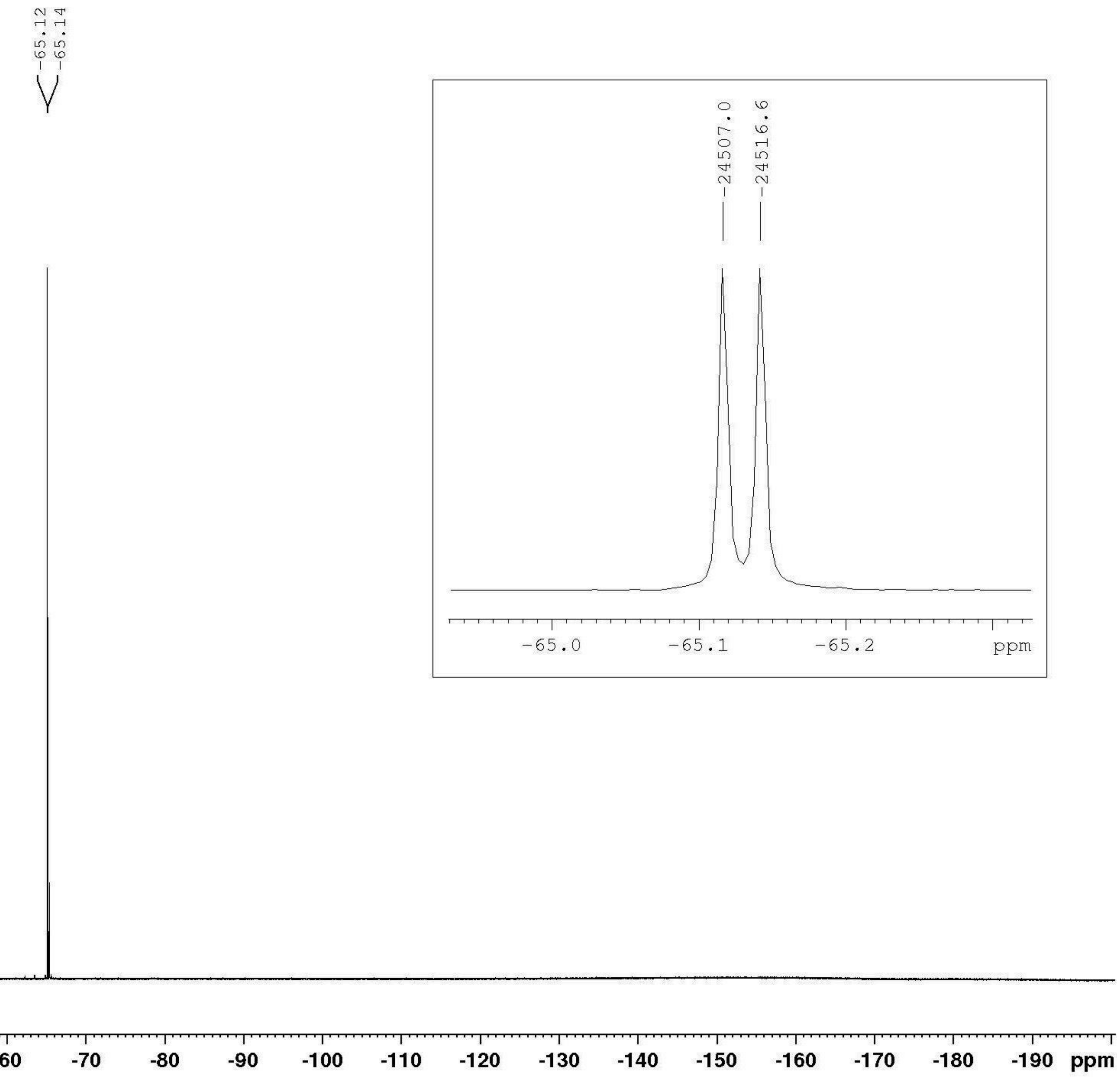

Supplement: Supplementary file 1 — ol2c01317_si_001.pdf [file ol2c01317_si_001.pdf]
